# Inhibitors of Apoptosis Protein Antagonists (Smac Mimetic Compounds) Control Polarization of Macrophages during Microbial Challenge and Sterile Inflammatory Responses

**DOI:** 10.3389/fimmu.2017.01792

**Published:** 2018-01-09

**Authors:** Vinod Nadella, Aparna Mohanty, Lalita Sharma, Sailu Yellaboina, Hans-Joachim Mollenkopf, Varadendra Balaji Mazumdar, Ramesh Palaparthi, Madhavi B. Mylavarapu, Radheshyam Maurya, Sreenivasulu Kurukuti, Thomas Rudel, Hridayesh Prakash

**Affiliations:** ^1^Laboratory of Translational Medicine, School of Life Sciences, University of Hyderabad, Telangana, India; ^2^YU-IOB Centre for Systems Biology and Molecular Medicine, Yenepoya University, Mangalore, India; ^3^Core Facility Genomics and Microarray, Max Planck Institute for Infection Biology, Berlin, Germany; ^4^Centre for Cellular and Molecular Biology, Hyderabad, India; ^5^Department of Animal Biology, School of Life Sciences, University of Hyderabad, Hyderabad, India; ^6^Biocentre, Department of Microbiology, University of Würzburg, Würzburg, Germany

**Keywords:** apoptosis, macrophages immunobiology, inflammation mediators, polarization, infection, hypothalamus

## Abstract

Apoptosis is a physiological cell death process essential for development, tissue homeostasis, and for immune defense of multicellular animals. Inhibitors of apoptosis proteins (IAPs) regulate apoptosis in response to various cellular assaults. Using both genetic and pharmacological approaches we demonstrate here that the IAPs not only support opportunistic survival of intracellular human pathogens like *Chlamydia pneumoniae* but also control plasticity of iNOS+ M1 macrophage during the course of infection and render them refractory for immune stimulation. Treatment of Th1 primed macrophages with birinapant (IAP-specific antagonist) inhibited NO generation and relevant proteins involved in innate immune signaling. Accordingly, birinapant promoted hypoxia, angiogenesis, and tumor-induced M2 polarization of iNOS+ M1 macrophages. Interestingly, birinapant-driven changes in immune signaling were accompanied with changes in the expression of various proteins involved in the metabolism, and thus revealing the new role of IAPs in immune metabolic reprogramming in committed macrophages. Taken together, our study reveals the significance of IAP targeting approaches (Smac mimetic compounds) for the management of infectious and inflammatory diseases relying on macrophage plasticity.

## Introduction

Deregulated host cell apoptosis leads to destructive inflammatory responses which are deleterious in nature as seen in septic shock, auto-immunity, cancer, and episode of chronic bacterial infections. Resistance to apoptosis relies on the increased expression and stability of anti-apoptotic proteins namely inhibitors of apoptosis proteins (IAPs). *Chlamydia pneumoniae*, a Gram-negative obligate intracellular human pathogen which is associated with many chronic inflammatory diseases (1) including chronic obstructive pulmonary disorders, chronic heart disease, asthma, and other idiopathic diseases (2–4), exploits host apoptosis pathways to escape eradication by immune effector cells (6, 7). Among all the cells with which these bacteria interact macrophages have gained particular importance, for their role in bacterial clearance and for acting as bacterial carriers during persistency. During acute infection, iNOS+ and pro-inflammatory macrophages are sufficient to clear off the infection from the lungs (8, 9). However, under chronic disease conditions such as bacterial persistency, particularly in the case of *C. pneumoniae* infection, inflammation can lead to vascular remodeling and tumor-like phenotype in the infected area, which is manifested by polarization of M1 effector macrophages toward M2 or refractory macrophages. It is anticipated that this mechanism could be decisive for neoplastic transformation of infected tissue and thus leading to the development of lung adenocarcinoma. We have previously shown that deregulated host cell apoptosis in *C. pneumoniae*-infected IAP-deficient mice helps these bacteria to replicate more efficiently as a result of weak immune response of macrophages and T cells (10, 11). In line with our previous data, here we demonstrate that IAPs are important for the phenotypical and functional plasticity of macrophages during the course of *C. pneumoniae* pulmonary infection. Furthermore, we also demonstrate here that targeting IAPs pharmacologically by Smac mimetic-based approaches (12, 13) led to metabolic programming of iNOS+ macrophages and enhancement of non-immunogenic and sterile inflammatory response in Th1-primed iNOS+. Altogether, our results provide new regulatory role of IAPs in immune-metabolic reprogramming and phenotypical plasticity of committed/activated macrophages that are important for chronic inflammatory diseases.

## Materials and Methods

### Antibodies and Reagents

The general reagents were purchased from Sigma-Aldrich (UK), unless stated otherwise. RPMI 1640, lipopolysaccharide (LPS), Gentamicin, MTT and NaNO_2_, sodium nitroprusside (SNP), penicillin–streptomycin solution, and metformin were procured from Sigma-Aldrich. Recombinant mouse IFNγ cytokine is from eBiosciences (San Diego, CA, USA). CD11b+ human and Mouse MACS Microbeads and LC Columns are from Miltenyi Biotec. Primary antibodies including rabbit polyclonal NOS-2, rabbit polyclonal xIAP, cIAP-1, cIAP-2, LAMP-2, CD-206, rabbit polyclonal β-actin, and mouse monoclonal β-actin are from Santa Cruz biotechnology. Mouse monoclonal arginase-1 is from BD Biosciences. Rabbit monoclonal STAT1 and 3, pp38MAPK, and pNF-kB p65 are from Cell Signalling Technology. Ym-1 antibody was purchased from Stem cell technologies; Fizz-1 antibody was from Abcam. HRP-linked anti-mouse IgG and anti-rabbit IgG are from Cell Signalling Technology. TNFα and IL-10 ELISA kits were purchased from R&D system (Darmstadt, Germany).

### Cell Lines, Cell Isolation, and Cell Culture

Standard murine macrophage cell line RAW264.7A, procured from ATCC, was cultured in RPMI 1640 medium containing 2 mM l-glutamine supplemented with 10% FCS, 100 IU/ml penicillin, and 100 µg/ml streptomycin. Both wild-type and well-described Ciap-1 knockdown He La cells (14, 15) were cultured in DMEM medium supplemented with 10% serum and antibiotics. Mouse peritoneal macrophages were elicited by 4% Brewer’s thioglycolate medium and purified by MACS bead-based method (Miltenyi Biotec). Briefly, C57BL/6j mice were injected with 4% Brewer’s thioglycolate medium (Fluka, Sigma-Aldrich) into the peritoneal cavity. Peritoneal exudates were obtained 72 h after injection by flushing the peritoneal cavity with ice-cold serum-free RPMI 1640 medium using a 22 G needle. Peritoneal lavage was pooled and centrifuged in a 50-ml conical centrifuge tube at 1,500 rpm for 10 min at 4°C, and the pellet was resuspended in RPMI 1640 medium supplemented with 10% heat-inactivated fetal calf serum, 15 mM HEPES buffer, and 2 mM l-glutamine. Macrophages from mice lungs were excised aseptically and digested for 1 h with collagenase containing RPMI 1640 medium. The digested lung tissues were gently mashed, and homogenates were prepared. RBCs in the tissue homogenates were lysed by RBC lysing buffer. Cells were washed with excess of serum-free RPMI medium. Primary blood mononuclear cells were isolated from citrated blood of healthy volunteers, following informed consent by the ficoll-based separation method. Monocytes were allowed to adhere to tissue culture wells (24-well plates) for 1 h prior to removal of other cells by washing with serum-free RPMI. Isolated monocytes were cultured for 7–8 days in a macrophage-SFM medium (Invitrogen Corp., Paisley, UK) at 37°C in a 5% CO_2_ incubator. The CD11b+ Gr-1 (−) macrophages from lung and peritoneal lavage from mice and PBMC from healthy donors were purified with CD11b+ Miltenyi micro beads, and the cells were FACS sorted for CD11b+ and Gr-1-negative population. Cells were counted and checked for viability by the trypan blue dye exclusion method. Cells were cultured at 2.5 × 10^4^ cells per well in a final volume of 200 µl in flat-bottom 96-well plate and incubated 0/N for adhesion at 37°C in a humidified environment at 5% CO_2_. Non-adherent cells were removed by washing with serum-free RPMI 1640 medium. Cells were stimulated with either LPS (1 µg/ml), IFNγ (50 ng/ml), or both (LPS + IFNγ) as needed.

### *Chlamydia* *pneumoniae* *Stock*

*Chlamydia pneumoniae* strain VR1310 was propagated in HEp-2 cells. These cells were infected with *C. pneumoniae* and lysed by mechanical disruption with a rubber policeman 72-h post infection. Bacteria were subsequently harvested by centrifugation at 500 × *g* at 4°C for 10 min. The pellet was ruptured using glass beads, and the lysates were centrifuged as before. The supernatants were removed and centrifuged at 45,000 × *g* for 45 min at 4°C in a SS34 rotor (Sorvall Instruments) to pellet Chlamydia. The bacteria were resuspended in sucrose phosphate glutamate buffer and stored at −80°C.

### Animal Experiments

WT C57BL/6, XIAP (B6.129X1-Birc4 tm1 Thsn/Hsd), and cIAP-1 knockout mice were used in this study. These mice were infected with *C. pneumoniae* serovar TW1310 by a non-invasive aerosol method as described earlier (10, 11). The mice were kept in S-2 animal house facility for a period of 20 days. The mice were sacrificed by cervical dislocation and whole lung tissue was harvested and analyzed for various M1 and M2 effector proteins by Western analysis. All animal experiments were performed as per the guidelines laid down and approved by Institutional Animal ethical committee approval (UH/IAEC/HP/2014-I/21).

### Microbial Infection

*Chlamydia pneumoniae* was propagated and cultivated in Hep2 as described. Mouse macrophages and epithelial cells were infected with *C. pneumoniae* at MOI-1 in RPMI medium containing 5% FCS. Cells were centrifuged at 700 × *g* for 1 h at RT for infection. After centrifugation, the cells were incubated for 1 h in a CO_2_ incubator at 37°C. After 1 h incubation period, the medium was replaced with RPMI containing 5% FCS, 1% Gentamicin, and 1 µg/µl of cycloheximide. The cultures were incubated further for different time intervals at 37°C in a CO_2_ incubator at 95% humidity. Glycerol stock of *Ms* was revived in Middle-brook 7H9 medium supplemented with 10% FBS and 0.2% glycerol and incubated at 37°C. *Escherichia coli* (*Ec*) was cultured in Luria broth at 37°C while *Leishmania donovani* (*Ld*) was cultured in RPMI medium containing 10% FBS and incubated at 25°C. All the cultures were periodically passaged. RAW macrophages were seeded in 24-well tissue culture plates (0.2 × 10^6^/well) and incubated overnight in a CO_2_ incubator at 37°C. Next day morning macrophages were infected with log phage cultures of *Ms* and *Ec* at MOI-10 and *Ld* at MOI-5. Before infection, bacterial clumps in *Ms* culture were removed by passing culture through 25G needle 15–20 times. After an hour of infection extracellular bacteria were removed by washing thrice with PBS, and then respective treatments were given with IFNγ (50 ng/ml), birinapant (60 µM), and combination of IFNγ and birinapant in RPMI medium (without FBS) containing Gentamicin (10 mg/ml). In the case of *Ld* infection, macrophages were infected for 6 h, and then subsequent treatments were given as described above. The cells were incubated further in a CO_2_ incubator and at different time intervals, cell culture supernatants for NO quantification and cell lysates for Western blot analysis of various proteins were collected. To demonstrate bacterial killing by macrophages, bacterial counts were performed over a time period of 48 h. For this purpose, cell lysates were serially diluted in sterile PBS and different dilutions plated over Middle-brook 7H10 agar plates for *Ms* and Luria agar plates for *Ec*. The bacterial colonies were counted after 3–5 days, and bacterial number was documented as colony forming units per milliliter.

### Macrophage Polarization Assay

RAW macrophages were cultured in complete RPMI medium at 2.5 × 10^4^ per well in 96-well plates for 24 h at 37°C. Spent medium in each well was replaced with fresh culture medium containing hypoxia inducer (CoCl2), angiogenic stimuli [vascular endothelial growth factor (VEGF)], and PancO2 tumor lysate, respectively, with and without birinapant and indicated Th1 stimuli.

### NO Production and Quantification

Nitric oxide production was quantified in macrophage culture supernatants through the standard Griess reagent method. Briefly, equal volumes of the culture supernatants and Griess reagent [1% sulfanilamide/0.1% *N*-(naphthyl) ethylenediamine dihydrochloride prepared in 5% o-phosphoric acid] were mixed and incubated for color development. Absorbance was measured at 550 nm using a Spectra max spectrometer (Molecular Devices ORT), and NO titers in samples were quantified against a NaNO_2_ standard curve generated using software provided with the Spectra max spectrometer (Molecular Devices).

### Western Blotting

CD11b+ Gr-1 (−) mouse pulmonary macrophages or lung tissues were lysed in RIPA buffer (50 mM Tris-HCl, pH 7.4, 150 mM NaCl, 2 mM EDTA, 1% Nonidet P-40, and protease inhibitor mixture) and sonicated. The lysate was centrifuged at 14,000 rpm for 20 min at 4°C to separate the particulate fraction. Protein concentration was determined by BCA kit (Peirce). 20 µg of proteins/group was separated on Nu-PAGE gradient gel and blotted on a PVDF membrane by wet electroblotting. Blots were blocked with 5% non-fat dry milk in TBS-T at pH 7.5 (20 mM Tris base, 137 mM NaCl, and 0.1% Tween 20) and then incubated over night at 4°C with primary antibodies followed by the HRP conjugated secondary antibody. For *in vitro* experiments, 1 × 10^6^ RAW macrophages from each experimental group were lysed, and proteins were prepared and analyzed for various signaling parameters by the Immunoblot method. For *ex vivo* stimulation experiments 1 × 10^6^ macrophages per well were used, while for infection experiments 1 × 10^4^ macrophages were used. Blots were developed by ECL reagent (Amersham Life Sciences), and actin was used as a loading control for normalization. The Western blots were quantified for densitometry by Image J software, and mean densitometry value of independent protein was divided with its mean densitometry value of its respective β-actin band intensity value to present the relative expression of each protein as a mean in the ratio of protein to actin.

### Immunofluorescence Staining

Cells cultured on glass cover slips were fixed with 4% PFA for 15 min and permeabilized by PBS-TritonX-100 (0.1%) for 5 min and blocked with TBS + 1% BSA + 1% sera from species of the respective secondary antibodies. After two washes with PBS, cells were incubated with primary antibodies overnight at 4°C followed by incubation of cells with respective secondary antibodies (Alexa Fluor-488 for green and -569 for red color) for 1 h. Nuclei were stained with DAPI for 5 min. Stained cells were washed, mounted on glass slides, and analyzed using a fluorescent microscope (Axivort, Carl Zeiss) under 63× magnification.

### Metabolic Activity Analysis

The MTT reduction assay is used to determine the level of metabolic activity in macrophages which relies on the reduction of MTT, a yellow water-soluble tetrazolium dye, primarily by the mitochondrial dehydrogenases, to purple colored formazan crystals. The formazan product is analyzed spectrophotometrically (550 nm) after dissolution in DMSO. Obtained OD values for control samples were normalized to 100%, and the difference in the metabolic activity of treated samples compared to control samples was analyzed.

### ELISA

Pulmonary titer of IL-10 in WT and xIAP KO mice both infected and uninfected and release of TNF in IFNγ-induced iNOS+ macrophages from both WT and xIAP KO were analyzed by sandwich ELISA according to instruction manual from manufacturer (R&D system). Similarly, soluble/shed TNFR1 (sTNFR1) in cell culture supernatants of cIAP1 shRNA transfected and vector control stable HeLa cell clones were infected with *C. pneumoniae* for one infection cycle were analyzed by ELISA according to the manufacturer’s instructions (BIOSOURCE).

### Wound Healing Assay

HeLa cells were grown in complete RPMI medium at 2.5 × 10^4^ per well in 24-well plates for 24 h at 37°C to form a cell monolayer before the wound was made by a scratch. Spent medium in each well was replaced with either fresh culture medium or conditioned medium by primary macrophages stimulated and treated with and without BP. The cells were cultured for another 24 h with and without BP, CoCl2, and VEGF, respectively. Images of the wound areas were taken by using an Inverted Fluorescence Microscope (IX73; Olympus Corporation, Japan) at 10× and compared with the images taken immediately after scratch was made. The wound areas were measured using the ImageJ software, and the percentage closure of the wound area was represented as mean ± SE from three independent experiments.

### Global Gene Expression Analysis

Both *cIap1* WT and KO macrophages were purified and stimulated with TNF for 24 h duration. mRNA was extracted from these cells, purified and then analyzed by whole mouse genome 44k microarray Kit (Agilent technology Cat No: G4122F, Chip ID no: 014868) various genes that were analyzed by Pathway Tools version 15.0, MetaCyc version 15.0. Onto-Tools^1^ Pathway Express was used to map differentially expressed genes, and Gene Spring 12.6.1 software (Agilent Technologies, Santa Clara, CA, USA) was used to prepare corresponding heat map. Pathways that were up/downregulated during TNF induction and/or CIAP-KO were identified using the previously developed method on Gene-Set enrichment analysis (16). Genessets for mouse were downloaded from Bader lab.^2^ The Gene sets were obtained by translation of human counterparts using orthologous relationship from homologene database. The Gene sets consist of pathways from PID, Reactome, Panther pathways, and gene ontology process from Mouse Genome Database. The data set was submitted to GEO website, and accession number of the same was obtained, which is mentioned later in “Results” section.

### Intracellular Signaling Arrays

PathScan Intracellular Signaling Array Kit was purchased from Cell Signaling Technology (Cell Signaling Technology, #7323). Whole cell lysates of naive and IFNγ stimulated macrophages with and without birinapant under both infectious and sterile inflammatory conditions were prepared using lysis buffer provided in the kit. 100 µl of each lysates was placed onto the membrane window of the antibody array-slide. The lysate treated slide was incubated overnight at 4°C on an orbital shaker. The slide was then washed with 100 µl 1× array wash buffer and incubated on the orbital shaker for 5 min at room temperature. This procedure was repeated three more times. 75 µl of 1× detection antibody cocktail was added to each of the 16 wells and covered with a sealing tape provided in the kit and incubated for 1 h at room temperature on an orbital shaker. Following three washes with 1× array wash buffer, the slide was incubated for 30 min with 75 µl 1× HRP-linked Streptavidin. The slide was washed and treated with LumiGlo and peroxide. The Bio-rad Gel Documentation System was used to take detailed pictures of the array. Images were analyzed by using ImageJ software, and mean densitometry values were plotted in terms of relative expression.

### Statistical Analysis

Data indicate mean ± SEM of representative experiments. Statistical significance was calculated by two-tailed unpaired *t* test for two data sets and two-way ANOVA followed by the Bonferroni test was used for multiple group comparisons (*p < 0.05; **p < 0.01; and ***p < 0.001).

## Results

### IAPs Control M1 Polarization of Macrophages during Bacterial Infection

**Figure 1:**
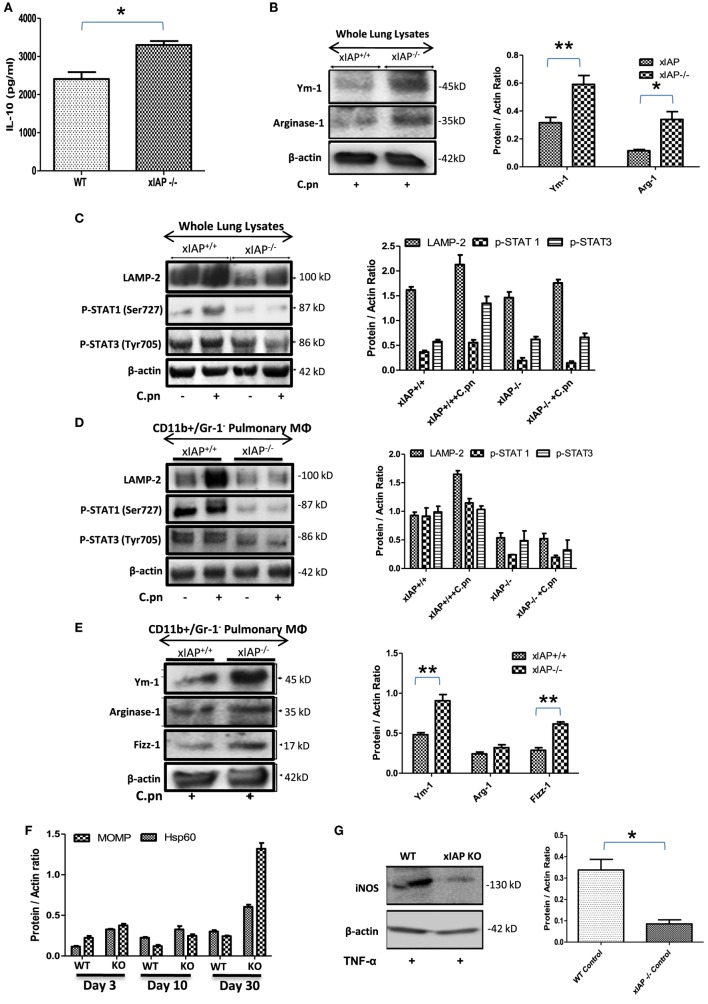
XIAP deficiency promotes M2 polarization during the course of infection. **(A)** Pulmonary titer of IL-10 in xIAP KO mice and its WT counterpart at 20th post infection day was analyzed by sandwich ELISA. **(B–E)** Whole lung tissue and CD11b+/Gr-1 (−) pulmonary macrophage cell lysates from both WT and Xiap KO mice 20 days after *Chlamydia pneumoniae* infection were analyzed by Western blotting for M1 and M2 signaling markers. **(F)** Expression of chlamydial HSP60 and major outer membrane proteins in the lung of XIAP KO-infected mice with time and **(G)** expression of iNOS protein in XIAP-deficient CD11b+/Gr-1 (−) pulmonary macrophages over WT counterpart in response to TNF stimulation were analyzed by Western blotting. 20 µg of protein per sample was analyzed by the immunoblot method as described in the “Materials and Methods” section. β-actin was used as a loading control. Representative blots from three independent mice infections with similar outcome are shown. The Western blots were quantified for densitometry by Image J software, and mean densitometry values of independent proteins were divided with its mean densitometry values of its respective β-actin band intensity value to present the relative expression of each protein as a mean in the ratio of protein to actin. Data shown here is the ±SEM from three independent experiments. Statistical analysis was conducted either by using two-tailed unpaired *t*-test and/or by using two-way ANOVA followed by Bonferroni post-test, respectively (*p < 0.05; **p < 0.01; and ***p < 0.001).

*ciap1* (11) and *Xiap* (10) KO mice failed to clear *C. pneumoniae* in spite of having high titers of IFNγ and TNFα which could be due to enhanced pulmonary titer of IL-10 in these mice (Figures 1A) over WT counterpart indicating Th2 biased immune response in XIAP-deficient mice. To further substantiate this, several M1 and M2 markers including STAT-1 and 3 (17), Ym-1, Fizz-1 and Arginase-1 (18, 19) in whole lung homogenates as well as in the purified CD11b+/Gr-1 (−) pulmonary macrophages from WT and xIAP KO mice were analyzed. Western blot analysis of infected XIAP KO lung (Figures 1B,C) and purified CD11b+/Gr-1 (−) pulmonary macrophages (Figures 1D,E) revealed downregulation of phospho-STAT-1 and upregulation of Ym-1 and Fizz-1 proteins in XIAP KO mice demonstrating M2/Th2-rich environment. Increase in the expression of chlamydial HSP60, whose expression was shown to be greatly enhanced in chlamydia infection and major outer membrane protein (an immunodominant surface antigen) in the lung of XIAP KO-infected mice was observed suggesting their possible involvement in promoting macrophage polarization (Figure 1F). Interestingly, reduced expression of phospho-STAT-1 proteins revealed IFNγ signaling defects in XIAP KO mice (17, 20). Moreover, reduced iNOS expression in XIAP-deficient CD11b+/Gr-1 (−) pulmonary macrophages over WT counterpart, in response to TNF stimulation (Figure 1G) revealing the importance of XIAPs in TNF-mediated skewing of M1 phenotype in these macrophages while explaining in part the redundancy of albeit high TNF in these mice. In similar lines, reduction in the expression of LAMP-2 proteins was evident in both infected XIAP KO lungs and in pulmonary macrophages (Figures 1C,D), and this could be due to strategic depletion of acidic lysosomes in host cells by these bacteria for adapting to hostile environment of XIAP-deficient macrophages (21).

Upregulation of cIAP proteins and reduced expression of XIAP proteins during the course of infection (11) revealed a possible compensation of IAP proteins in ciap-1 KO animals. However, XIAP proteins remained redundant in controlling cIAP-1 proteins in XIAP KO macrophages and lung. Therefore, we anticipated cIAP-1 dependent regulation of TNF response in both cIAP-1 and XIAP KO macrophages. To that purpose, global gene expression analysis was performed in purified CD11b+/Gr-1 (−) peripheral macrophages from WT and *ciap1* KO mice. In line with our previous observation, microarray data (Accession Nr: GSE106451) revealed the upregulation of genes responsible for angiogenesis and downregulation of genes responsible for immune, anti-microbial response in cIAP1 KO macrophages over WT counterpart (Figures S1A–E in Supplementary Material; Table 1A,B). Most interestingly, the *ciap1* deletion resulted in upregulation of IL-5, NOS1, VEGF, and TAK1 and PPARG genes in response to TNF, all of which are associated with M2 immune responses indicating the sensitization of IAP-deficient mice for infection and macrophages for M2 polarization.

**Table 1:** Global gene expression analysis by microarray in purified CD11b+/Gr-1 (‒) peripheral macrophages from WT and ciap1 KO mice.

(A) CIAP1-KO Vs WT (Naive)

Upregulated	Downregulated
TNFR2, INTRINSIC, NO1, VIP, 41BB, TNFR2, ACH, and NKT	PYK2, IL1R, MAL, MITOCHONDRIA, HCMV, IL17, RAS, NEUROTRANSMITTERS, ERYTH, HER2, HCMV, BARR_MAPK, FAS, SARS, and GLYCOLYSIS

Positive regulation of ER associated ubiquitin-dependent protein catabolic	Cranial nerve formation
Regulation of receptor internalization	

Peptidyl asparagine modification	Stress response to metal ion
Regulation of cholesterol esterification	Regulation of receptor internalization
Positive regulation of cholesterol esterification	Negative regulation of leukocyte adhesion to vascular endothelial cell
Protein N-linked glycosylation *via* asparagine	Regulation of membrane repolarization during action potential
Cranial nerve formation	Adenylate cyclase activating G protein-coupled receptor signaling pathway
Negative regulation of retrograde protein transport ER to cytosol	DNA replication-dependent nucleosome assembly and organization
Negative regulation of protein exit from endoplasmic reticulum	Common myeloid progenitor cell proliferation
Positive regulation of regulatory T cell differentiation	Positive regulation of transcription from RNA polymerase III promoter
Regulation of NFAT protein import into nucleus	Negative regulation of megakaryocyte differentiation
Regulation of cytokine production	Regulation of type B pancreatic cell proliferation
Regulation of cytokine biosynthetic process	
Regulation of interleukin 2 biosynthetic process	
Regulation of immature T cell proliferation in thymus	
Serine family amino acid catabolic process	

FOA2 and FOA3 transcription factor networks	FOM1 transcription factor network
Glucocorticoid receptor regulatory network	EphrinA EPHA pathway
Alpha6 beta4 integrin ligand interactions	Non-genotropic androgen signaling
	Arf6 signaling events
	GMCSF-mediated signaling events
	IL27-mediated signaling events

Heterotrimeric G protein signaling pathway	X5 hydroxytryptamine degradation
Gi alpha- and Gs alpha-mediated pathway	Adrenaline and noradrenaline biosynthesis
Heme biosynthesis	

Gluconeogenesis	Integrin cell surface interactions
Defective CSF2RB causes pulmonary surfactant metabolism dysfunction 5 (SMDP5)	
Defective CSF2RA causes SMDP4	

Glyoxylate metabolism and glycine degradation	Clearance of nuclear envelope membranes from chromatin
Trafficking of myristoylated proteins to the cilium	MAPK1 ERK2 activa
Regulation of beta cell development	Defective CSF2RB causes SMDP5
Anchoring fibril formation	Diseases associated with surfactant metabolism IRAK2-mediated activation of TAK1 complex
	Formation of ATP by chemiosmotic coupling
	Keratan sulfate biosynthesis

**(B) CIAP1-KO Vs WT (Th1 primed)**	

**Upregulated**	**Downregulated**

SODD, IL15, AMI, INTRINSIC	IL10, AKT, RANKL, Glycolysis, SARS, IL17, RAS, GCR

Regulation of protein deubiquitination	Positive regulation of ER-associated ubiquitin-dependent protein catabolic
	Regulation of receptor internalization

Actin nucleation	Regulation of Golgi inheritance
Arp2 3 complex-mediated actin nucleation	Regulation of NFAT protein import into nucleus
Chromosome movement toward spindle pole	Positive regulation of regulatory T cell differentiation
Cellular response to parathyroid hormone stimulus	Cell adhesion mediated by integrin
Meiotic cytokinesis	Cellular amino acid catabolic process
Carbon dioxide transport	Pyroptosis
Cholesterol homeostasis	Histamine metabolic process
Sterol homeostasis	Imidazole containing compound metabolic process
Stress response to metal ion	Inflammatory response
Positive regulation of deacetylase activity	Positive regulation of plasminogen activation
Positive regulation of histone deacetylase activity	DNA dealkylation
Sphingoid biosynthetic process	DNA demethylation
Cellular modified amino acid catabolic process	
Heme transport	
Hormone catabolic process	
Fructose metabolic process	

Alpha6 beta4 integrin ligand interactions	Trk receptor signaling mediated by PI3K and PLC gamma
Endogenous TLR signaling	FOA1 transcription factor network
Beta2 integrin cell surface interactions	Alpha9 beta1 integrin signaling events
Syndecan 4-mediated signaling event	Arf6 signaling events
Integrin family cell surface interactions	

Inflammation mediated by chemokine and cytokine signaling pathway	N-Acetylglucosamine metabolism
	Angiogenesis
	Vitamin D metabolism and pathway
	Gamma aminobutyric acid synthesis

TAK1 activates NFkB by phosphorylation and activation of IKKs complex	Defective CSF2RA and CSF2RB cause pulmonary surfactant metabolism
	Diseases associated with surfactant metabolism
	Classical Kir channels

Innate immune system	Defective CSF2RB causes SMDP5
Toll-like receptor 3 cascade	Diseases associated with surfactant metabolism
MyD88-independent TLR3 TLR4 cascade	Amine-derived hormones
TAK1 activates NFkB by phosphorylation and activation of IKKs complex	VLDL biosynthesis
	DEx H box helicases activate type I IFN and inflammatory cytokines production
	Metallothioneins bind metals, response to metal ions, and classical Kir channels

### IAP Deficiency Renders Infected Macrophages Refractory for Immune Stimulation

**Figure 2:**
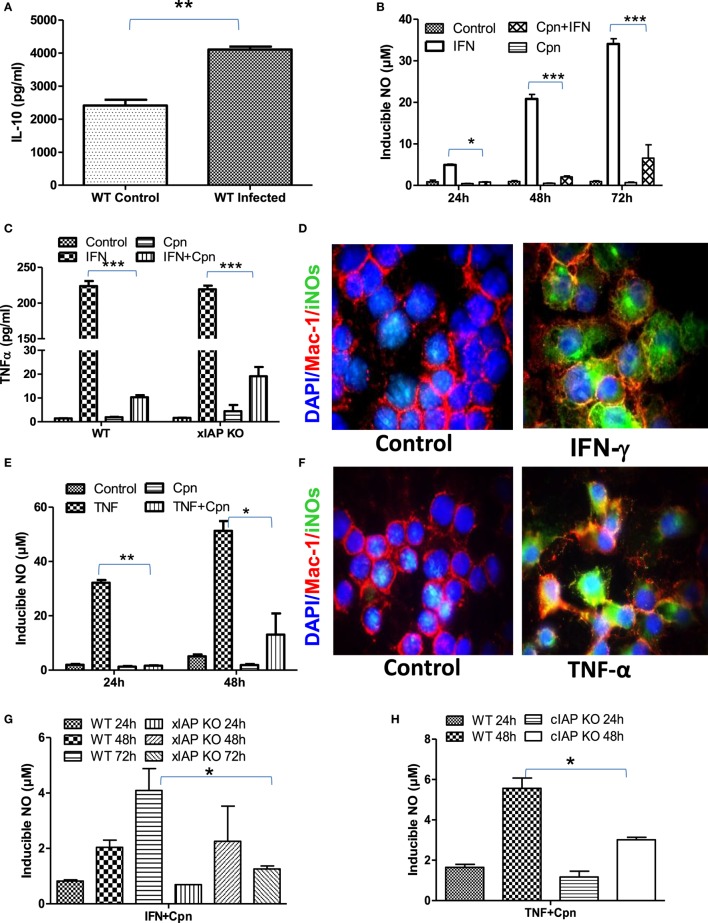
*Chlamydia pneumoniae*-infected macrophages are refractory for immune stimulation. **(A)** Pulmonary titers of IL-10 in both WT-infected and uninfected mice were analyzed by sandwich ELISA. **(B)** IFNγ induced generation of NO by WT, and cpn-infected CD11b^+^/Gr-1 (−) peritoneal macrophage was quantified from the culture supernatants by the Griess reagent method. **(C)** Release of TNF in IFNγ-induced iNOS+ macrophages was quantified from both WT and xIAP KO by ELISA and **(D)** expression of iNOS proteins in CD11b+/Gr-1 (−) peritoneal macrophages from WT mice was confirmed by the immunofluorescence method upon stimulation with IFNγ for 24 h showing M1 polarization of these macrophages against control. In similar lines, TNF induced generation of NO by both WT and cpn-infected CD11b^+^/Gr-1 (−) peritoneal macrophage was quantified from the culture supernatants by the Griess reagent method **(E)** and the expression of iNOS proteins in CD11b+/Gr-1 (−) peritoneal macrophages from WT mice was confirmed by the immunofluorescence method upon stimulation with IFNγ for 24 h **(F)**. IFNγ induced generation of NO by WT and xIAP KO **(G)** and TNF induced generation of NO by WT and cIAP KO upon infection with *C. pneumoniae* was quantified from the culture supernatants by the Griess reagent method **(H)**. Statistical analysis was conducted either by using a two-tailed unpaired *t*-test and/or by using two-way ANOVA followed by Bonferroni post-test, respectively (*p < 0.05; **p < 0.01; and ***p < 0.001).

Macrophages are double edge components of the innate immune system and promote adaptive immune response for the clearance of infection on one hand while being responsible for various infection-related idiopathic disorders mainly during bacterial persistency on the other. *Chlamydia* containing endosomes in foamy macrophages in coronary atheroma patients clearly demonstrate the dichotomy of macrophage behavior during persistent infection which was defined to be due to polarization of these regulatory macrophages toward refractory/patrolling/foamy phenotype by these bacteria for their survival (22). To address this, IL-10 titers in both *C. pneumoniae*-infected WT and XIAP−/− mice were analyzed. Increased pulmonary titer of IL-10 in infected mice (Figure 2A) clearly revealed Th2 programming of immune response by these viable bacteria. *In vitro* data with *C. pneumoniae*-infected CD11b+/Gr-1 (−) peripheral macrophages also demonstrated a significant inhibition of NO generation upon IFNγ induction (Figure 2B) and release of TNF in IFNγ-iNOS+ macrophages (Figures 2C,D) clearly demonstrating the loss of Th1 programming in these infected macrophages. IL-10 expression has also shown to inhibit IFNγ-dependent NO production by macrophages and thereby their ability to control parasitic infections (23). In similar lines, these bacteria also abolished TNF-induced generation of NO in iNOS+ macrophages (Figures 2E,F). Induction of spontaneous shedding of sTNFR1 in their culture supernatants of Chlamydia-infected epithelium (Figure S2 in Supplementary Material) demonstrated the sabotage of TNF response by these bacteria. Most interestingly, IAP-deficient macrophages remained more refractory over WT-infected macrophages indicating the dependency of macrophages on IAP proteins for immune restoration (Figures 2G,H). Chlamydia infection not only inhibited the immune mediated activation of macrophages, but also abolished sodium nitroprusside (SNP)-induced release of NO in these macrophages (Figures S3A,B in Supplementary Material) which is most likely due to chelation of Fe^+++^ to Fe++ by these bacteria which promote bacterial persistency. Thus, these data, in support of our hypothesis, provided experimental evidence that these bacteria are capable of rendering infected macrophages refractory to immune-mediated stimulation during persistent phase. C. pneumoniae infection alone or in combination with Th1 effector cytokines could not enhance Caspase-3 levels in macrophages (11), suggesting that apoptosis in these macrophages may not have accounted for M2 polarization of infected macrophages. To further elucidate the reason of this interference, we analyzed the mitochondrial activities which are known to have an impact on iNOS proteins and their active involvement in anti-bacterial defense of infected macrophages (24). Standing in line with NO and TNF data, complete or significant reduction in the metabolic activities (25) of both naïve as well as Th1-primed M1 or SNP -treated macrophages by C. pneumoniae (Figures S4A–D in Supplementary Material) was evident suggesting dysregulation of mitochondrial functions by these bacteria. This could be a potential and additional mechanism involved in skewing M1 dim/M2 polarization of macrophages due to the fact that during the course of M2 polarization, macrophages get metabolically programmed to derive their metabolic energy through oxidative phosphorylation which is known to suppress mitochondrial membrane potential (MMP) and mROS production. To clarify this, we used intra- as well as extracellular-unrelated bacteria like Mycobacterium smegmatis (Ms) and Escherichia coli (Ec) and expected their redundant impact on macrophage stimulation. Unlike Chlamydia, stimulation of Ms- and Ec-infected macrophages with IFNγ enhanced the infection induced levels of NO synergistically at both 24 h and 48 h post infection (Figures S5A,B in Supplementary Material), confirming Chlamydia-driven interference of macrophages.

### Smac Mimicry Promote Anti-Bacterial Response of Naive Macrophages

**Figure 3:**
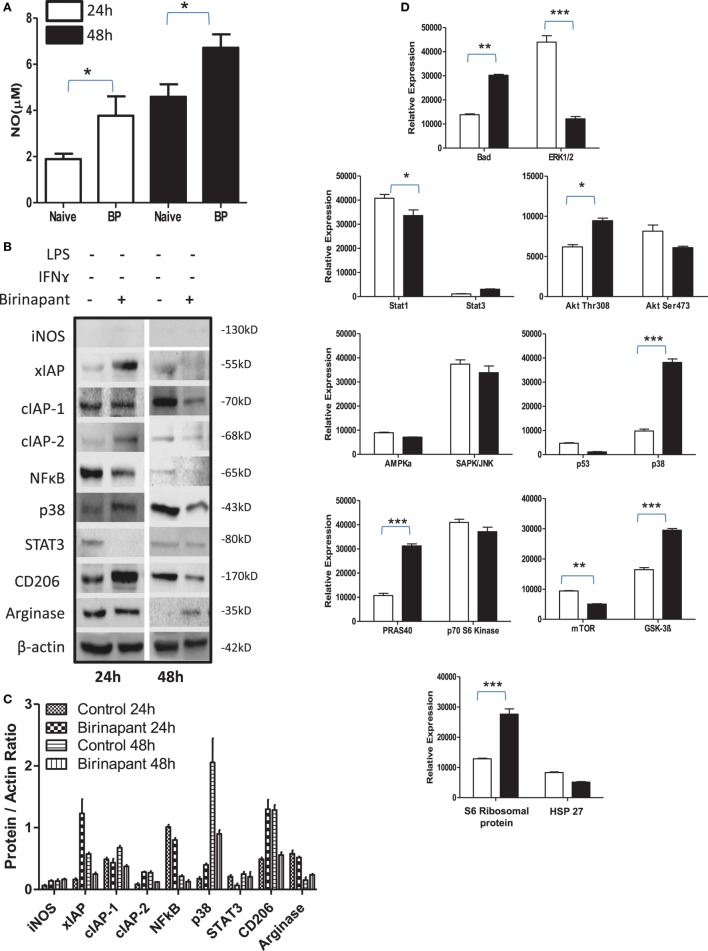
Birinapant regulates phenotypic and immune-metabolic programming in naive macrophages. RAW264.7A murine MΦ was treated with and without birinapant and cultured for indicated time points. **(A)** NO titer was quantified in the culture supernatants by the Griess reagent method. **(B)** The cultures mentioned under **(A)** were lysed and analyzed for various M1 and M2 effector proteins, inhibitors of apoptosis proteins, and signaling markers by Western blotting. **(C)** The Western blots were quantified for densitometry by Image J software, and mean densitometry values of independent proteins were divided with its mean densitometry values of its respective β-actin band intensity value to present the relative expression of each protein as a mean in the ratio of protein to actin. **(D)** To monitor the intracellular signaling, important metabolic signaling component activation was observed using PathScan Intracellular Signaling Array Kit from Cell Signaling Technology. Images were analyzed by using ImageJ software, and mean densitometry values were plotted in terms of relative expression. Statistical analysis was conducted using two-way ANOVA followed by the Bonferroni post-test (*p < 0.05; **p < 0.01; and ***p < 0.001).

During persistency, *Chlamydia*-infected cells display typical characteristics of tumor cells and relay tumorigenic signals. Therefore, we argued whether their stimulation with Smac mimetic compound: birinapant, a well-known IAP-specific inhibitor (26–28) known to boost immunity against various tumors, would revert refractory phenotype of infected macrophage. To this end, murine macrophage activation was analyzed upon treatment with birinapant. Birinapant enhanced constitutive levels of NO generation (Figure 3A) without influencing the expression of iNOS (Figures 3B,C) thus indicating its capability of skewing M1 phenotype in naïve macrophages. This outcome could be either due to enhanced expression or release of mitochondrial NO synthase along with SMAC Diablo which could have contributed for high NO titers by naïve macrophage. Birinapant treatment also downregulated AMPK/mTOR proteins and upregulated BAD/GSK-3 pathways (Figure 3D) which are important for metabolic pathways, indicating immune metabolic programming of smac flushed macrophages. On the basis of these results, we presumed whether birinapant would enhance the defense mechanisms of macrophages. To demonstrate this, macrophages were infected with *Ms* and *Ec* followed by stimulation with IFNγ in the presence or absence of birinapant. Interestingly, treatment of infected macrophages with birinapant either alone or in combination with IFNγ enhanced infection-induced NO levels and bacterial killing capacity of macrophages significantly for *Ms* and transiently for *Ec* (Figures S5A–F and S6A,B in Supplementary Material), indicating the novel anti-bacterial characteristics of birinapant in line with its anti-tumor impact. Birinapant treatment enhanced IFNγ-induced expression of iNOS synergistically in infected cells (M1 proteins) at 48 h post infection time interval (Figures S6C,D in Supplementary Material) which was accompanied with the upregulating XIAP proteins. This indicated a compensation of XIAP in birinapant-exposed macrophages and suggested probable involvement of XIAP proteins in birinapant-induced anti-bacterial response in macrophages. On this basis, we argued whether birinapant would be able to maintain M1 effector phenotype in macrophage in response to *Ld* which drives M2 polarization in macrophages. To test this, RAW macrophages were infected with *Ld* and analyzed for changes in NO titer during the course of infection as indicative of M1 phenotype. Unlike *Ms*/*Ec* infection and in line with *C. pneumoniae* infection, *Ld* infection not only reduced constitutive NO titers in these macrophages but also resisted the generation of NO upon their costimulation with Th1 cytokines (Figure S7 in Supplementary Material). Contrary to our prediction and unlike *Ms*/*Ec* infection, birinapant could neither influence *Ld*-induced NO levels over *Ld* control nor assisted IFNγ for boosting NO level over IFNγ control, indicating its redundancy for affording immunity in the macrophages infected with pathogenic microbes.

### IAP Deficiency Promotes M2 Polarization of M1 Effector Macrophages

**Figure 4:**
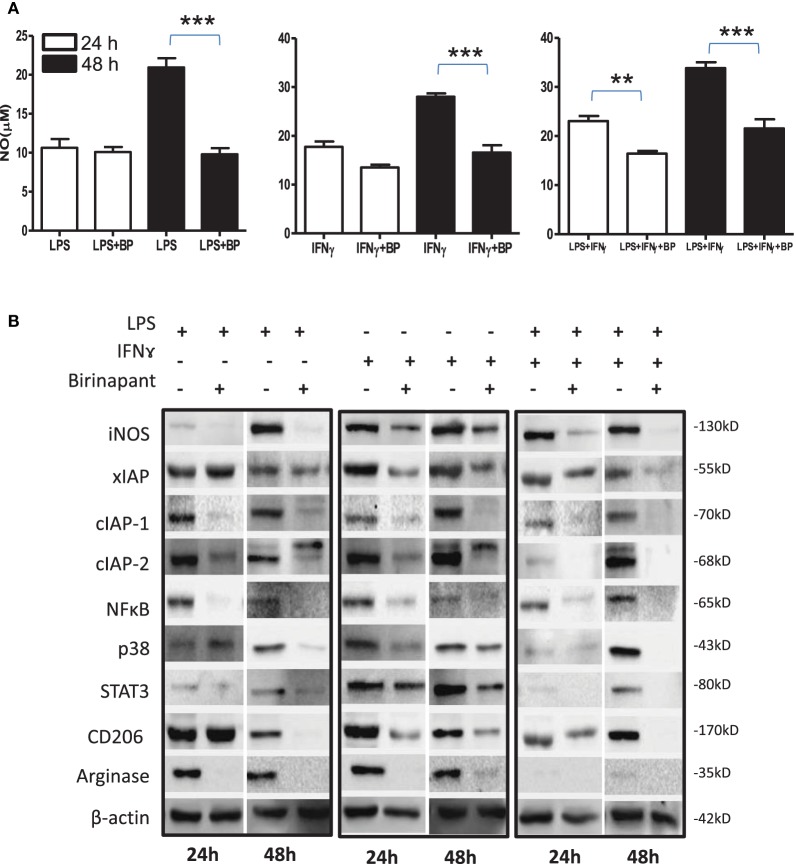

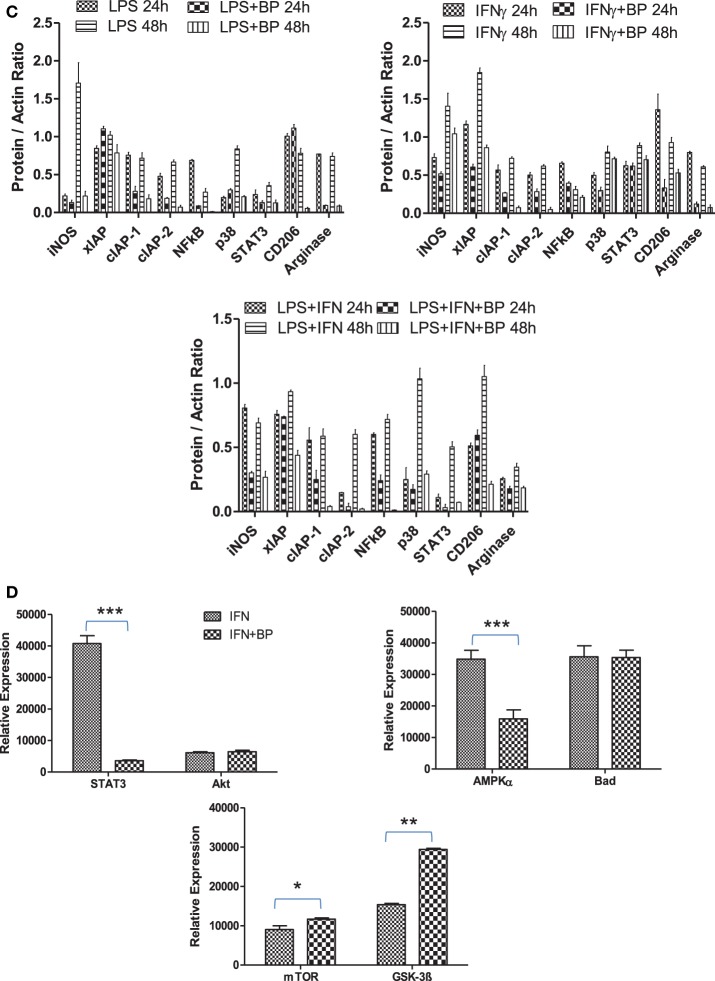
Birinapant regulate M1 programming and immune-metabolic programming in lipopolysaccharide (LPS) and/or IFNγ-skewed iNOS+ macrophages. RAW264.7A murine MΦ was stimulated with Th1 cytokines LPS or IFNγ and both LPS and IFNγ with and without birinapant and cultured for indicated time points. **(A)** NO titer was quantified in the culture supernatants by the Griess reagent method. The data are represented as mean μM of NO ± SEM, and statistical analysis was conducted using two-way ANOVA followed by the Bonferroni post-test (*p < 0.05; **p < 0.01; and ***p < 0.001). **(B)** The cultures mentioned under **(A)** were lysed and analyzed for various M1 and M2 effector proteins, inhibitors of apoptosis proteins, and signaling markers by Western blotting. **(C)** The Western blots were quantified for densitometry by Image J software, and mean densitometry values of independent proteins were divided with its mean densitometry values of its respective β-actin band intensity value to present the relative expression of each protein as a mean in the ratio of protein to actin. **(D)** To monitor the intracellular signaling, important metabolic signaling component activation was observed using PathScan Intracellular Signaling Array Kit from Cell Signaling Technology. Images were analyzed by using ImageJ software, and mean densitometry values were plotted in terms of relative expression. Statistical analysis was conducted using two-way ANOVA followed by the Bonferroni post-test (*p < 0.05; **p < 0.01; and ***p < 0.001).

**Figure 5:**
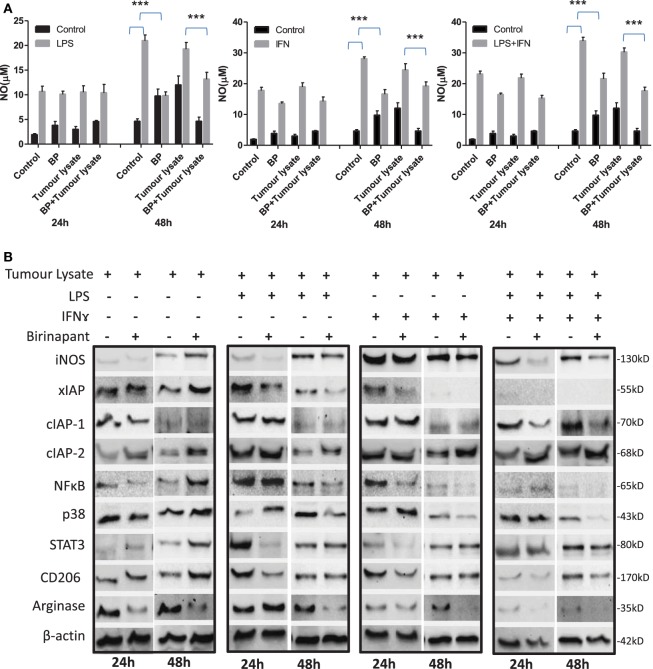

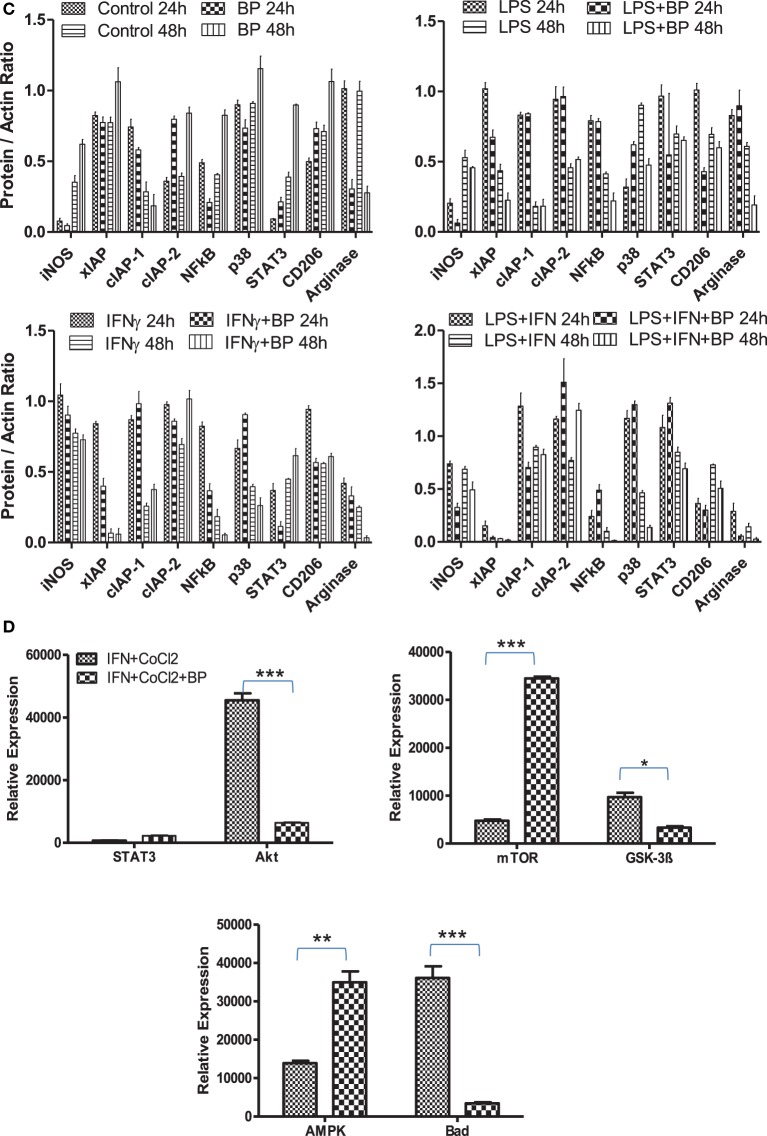
Inhibitor of apoptosis proteins (IAP) regulates tumor lysate mediated sterile inflammatory responses and immune-metabolic programming in iNOS+ macrophages. **(A)** RAW264.7A murine MΦ was stimulated with Th1 cytokines lipopolysaccharide (LPS) or IFNγ and both LPA and IFNγ with and without lysate prepared from PancO2 pancreatic tumor cell line (100 ng protein) and birinapant and cultured for indicated time points. NO titer was quantified in the culture supernatants by the Griess reagent method. Shown here is the mean μM of NO ± SEM from three independent experiments. Statistical analysis was conducted using two-way ANOVA followed by Bonferroni post-test (***p < 0.001). **(B)** The cultures mentioned under A were lysed and analyzed for various M1 and M2 effector proteins, IAPs, and signaling markers by Western blotting. **(C)** The Western blots were quantified for densitometry by Image J software, and mean densitometry values of independent proteins were divided with its mean densitometry values of its respective β-actin band intensity value to present the relative expression of each protein as a mean in the ratio of protein to actin. **(D)** To monitor the intracellular signaling, important metabolic signaling component activation was observed using PathScan Intracellular Signaling Array Kit from Cell Signaling Technology. Images were analyzed by using ImageJ software, and mean densitometry values were plotted in terms of relative expression. Statistical analysis was conducted using two-way ANOVA followed by Bonferroni post-test (*p < 0.05; **p < 0.01; and ***p < 0.001).

On the basis of anti-bacterial nature of birinapant, we argued whether birinapant would enhance M1 phenotype further in Th1-primed macrophages or not. In contrast, birinapant treatment inhibited M1 programming in LPS- and/or IFNγ-primed macrophages evidenced by significant inhibition in NO generation and reduction in the expression of key signaling, immune- and metabolism-related proteins in committed macrophages (Figures 4A–D). These results, in line with both our microarray and *in vivo* data, supported the involvement of IAPs in immune-metabolic programming of committed macrophages indicating the M2 polarization of birinapant-treated macrophages. To further confirm this observation, PancO2 tumor cell-derived lysate was used for inducing M2 or tumor-associated phenotype in M1 macrophages (29), both in the presence and absence of birinapant. Tumor lysate-pulsed M1 macrophages produced minimal NO titers analyzed from their cell culture supernatants. Interestingly, NO titers were further put down upon treating tumor lysate-pulsed M1 macrophages with BP comparatively (Figure 5A). These observations were also accompanied with expected changes in iNOS and other key M1-associated proteins like STAT-3, NF-κB, and phospho-p38MAPK and IAPs (Figures 5B–D) thus suggesting the temporal regulation of IAPs during polarization of macrophages.

During chronic inflammatory conditions, extreme hypoxia prevail manifesting in the stabilization of HIF-1 which is one of the decisive tissue specific factors that control macrophage mitochondrial functions during inflammatory. Our previous report has also identified HIF-1 stabilization during persistent infection with *Chlamydia trachomatis* persistency. Therefore, we anticipated that IAP deficiency might promote HIF-1-associated response in macrophages as well. To demonstrate this, iNOS+ macrophages were treated with CoCl_2_ (Cobalt chloride) for inducing the expression of HIF-1 (Figure S8 in Supplementary Material) in the presence and/or absence of birinapant and NO, innate immune and metabolism-related proteins were analyzed. CoCl_2_ treatment resulted in the increased production of NO and iNOS expression levels in naïve macrophages but inhibited the same in M1 macrophages (Figures S8A–E in Supplementary Material). Interestingly, birinapant treatment further reduced NO levels in CoCl_2_-treated M1 macrophages over CoCl2 control and influenced innate immune and metabolic proteins and promoted HIF-1-induced M2 polarization. To confirm the impact of IAP on angiogenesis, which is sensed by VLH and executed by VEGF, one of the HIF-1 target proteins and which gets upregulated in M2 polarized macrophages for compensating O_2_ demand; effect of birinapant on VEGF-treated macrophages was analyzed. Interestingly, VEGF stimulation has not altered constitutive NO levels significantly in comparison to control either in naïve or Th1-primed conditions. However, treatment with birinapant resulted in NO inhibition significantly while influencing signaling and metabolism-related proteins (Figures S9A–D in Supplementary Material). These cell line-based results provoked us to confirm the same in mouse primary macrophages. For that purpose, purified CD11b+/Gr-1 negative peripheral macrophages from mice were treated with either CoCl2 or VEGF and subsequently analyzed for NO titers by these macrophages in naïve or Th1-primed conditions upon treatment with/without birinapant (Figure S10 in Supplementary Material). In line with our *in vitro* data, treatment of these macrophages with VEGF/COCl_2_ alone or in combination inhibited IFNγ-induced NO titer slightly in naïve (Figure S10A in Supplementary Material) but significantly in M1-differentiated macrophages (Figure S10B in Supplementary Material). This was reduced further upon costimulation of these macrophages with birinapant, while proving the involvement of IAPs in VEGF/hypoxia-driven M1 dim polarization in mouse primary macrophages.

### Smac Mimicry Promotes Wound Healing Potential in Polarized Macrophages

**Figure 6:**
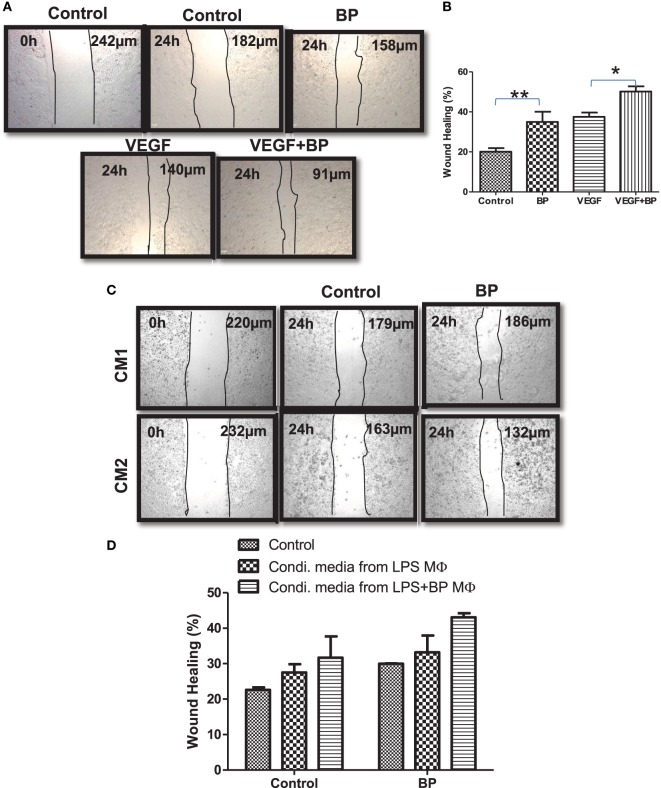
Inhibitors of apoptosis proteins regulate wound healing efficiency of primary macrophages. **(A)** HeLa cells at 2.5 × 10^4^ per well in 24-well plates cultured in complete RPMI medium for 24 h at 37°C to form a cell monolayer before the wound was made by a scratch. HeLa cells were allowed to migrate for 24 h upon treating with and without BP and vascular endothelial growth factor, respectively. Images of the wound areas were captured using an Inverted Fluorescence Microscope, at 10× and compared with images taken immediately after scratch was made. **(B)** Wound area was quantified by analyzing the images using ImageJ, and the percentage closure of the wound area was plotted as mean ± SE. **(C)** HeLa cells described in **(A)** were cultured in media conditioned by primary macrophages stimulated with lipopolysaccharide (LPS) (CM1) and/or in media conditioned by primary macrophages stimulated with LPS and treated with birinapant (CM2). Images of the wound areas were captured using an Inverted Fluorescence Microscope, at 10× and compared with images taken immediately after scratch was made. **(D)** Wound area was quantified by analyzing the images using ImageJ, and the percentage closure of the wound area was plotted as mean ± SE. Images presented are the representative of three independent experiments done in triplicates. Statistical analysis was conducted using two-way ANOVA followed by the Bonferroni post-test (*p < 0.05 and **p < 0.01).

M2 macrophages are potentially refractory and bear wound healing capacity. On the basis of M2 polarization of IAP-deficient macrophages, wound healing or angiogenic potential of IAPs in macrophages in the presence and absence of birinapant was analyzed. For this purpose, CD11b+/Gr-1 (−) peripheral macrophages from healthy donors were isolated, purified, and cultured for 4 days in the presence of G-CSF-rich medium to allow their maturation. These cells were stimulated with LPS with and without birinapant. Their conditioned media were collected 24 h post treatment and used as a source of various cytokine/growth factor secreted by these macrophages during polarization. Monolayer of HeLa cells was used for analyzing the wound healing potential of birinapant under the influence of macrophage-conditioned media. Birinapant alone or in combination with VEGF promoted wound healing in the HeLa cells providing an evidence for wound healing, angiogenic potential of Smac-mimetics (Figures 6A,B). Interestingly, the extent of wound healing of HeLa cells treated with birinapant directly or the condition media obtained from birinapant-pulsed macrophages remained comparable suggesting that IAPs not only control macrophages polarization but also angiogenic potential of the macrophages (Figures 6C,D).

### Deficiency of IAPs Promote Immune Metabolic Programming in Macrophages

**Figure 7:**
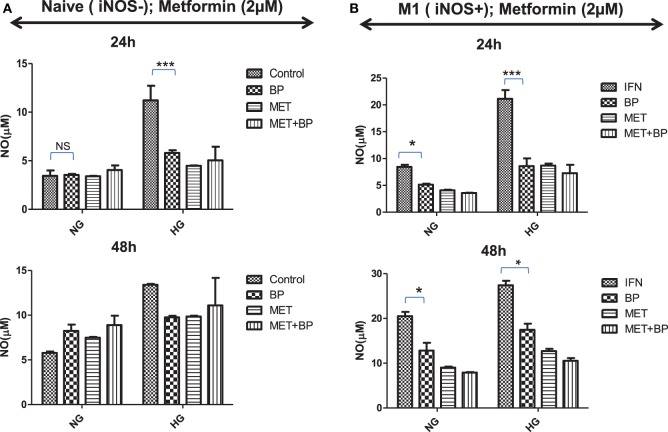
Inhibitors of apoptosis protein reprogram metabolic activity in both naive and Th1-primed macrophages during macrophage polarization. Naïve and Th1-primed RAW264.7A murine MΦ with **(A)** Naïve and **(B)** Th1 primed RAW264.7A murine MΦ (M1; iNOS+) were treated with metformin and cultured in both normal and high glucose condition in the presence and absence of birinapant and cultured for indicated time points. NO titer was quantified in the culture supernatants by the Griess reagent method. Data shown here is ±SEM from three independent experiments. Statistical analysis was conducted using two-way ANOVA followed by the Bonferroni post-test (*p < 0.05; **p < 0.01; and ***p < 0.001).

M1 or committed macrophages, during their encounter with pathogens, rely on glucose-induced glycolytic and Warburg reactions, deriving energy for performing various immune related functions. In contrast, M2-differentiated macrophages perform their function on the energy provided by oxidative phosphorylation which fuel polarized macrophages at much lower rate compared to glycolytic pathways. As it is quite evident that immune metabolism is decisive for their functional plasticity, the functional role of IAP in immune metabolic programming of macrophages was analyzed. Smac Diablo is a mitochondrial protein with active involvement of mitochondria in both immunity and metabolism, we anticipated for birinapant-mediated metabolic reprogramming of macrophages and found that birinapant downregulated AMPK/mTOR pathways while upregulating BAD/GSK-3 pathways in macrophages. On this basis, it was rationale to presume that IAPs other than controlling cell death and immunity also play a decisive role in cellular metabolism in macrophages. To confirm this hypothesis, both naïve and iNOS+ M1 macrophages were treated with different concentrations of glucose and analyzed the glucose treatment effect on NO generation as indicative of M1 polarization and metabolic activities in macrophages. Culturing naïve macrophages with high glucose over normal glucose containing medium not only enhanced NO levels but also enhanced their metabolic activities while conferred M1-like phenotype, similar to the phenotype of macrophages from diabetic patients (Figures 7A,B). Most interestingly, treatment of high glucose fed macrophages with birinapant reduced both NO levels and metabolic activities (Figure S11 in Supplementary Material) in these cells significantly indicating disruption of M1 signature by targeting IAP. On the basis of birinapant-driven downregulation of AMPK, we anticipated that birinapant may improve the action of metformin which also inhibits AMPK. To address our hypothesis, macrophages were treated with metformin alongside birinapant treatment in both low and high glucose conditions. Although the treatment of naïve macrophages with metformin did not influence the levels of NO in these macrophages when cultured in normal glucose conditions, the same inhibited NO titer significantly in the macrophages cultured in high glucose containing medium (Figure 7A). NO levels in metformin-treated group remained comparable to birinapant-treated macrophages. Interestingly, and unlike naïve macrophages, exposing iNOS+ macrophages to metformin strongly inhibited IFNγ-induced NO levels (Figure 7) which were further reduced by birinapant. Interestingly, compared with naïve macrophages, birinapant remained more effective in Th1-primed cells in breaking down the M1 phenotype. Thus, these results revealed for the first time the involvement of IAPs in immune-related metabolism.

## Discussion

Host cell apoptosis is vital for orchestrating optimum immune response against microbes and tumors. In this study, we discussed and provided a mechanism explaining how dysregulation of apoptosis can jeopardize the immune response of macrophages. Macrophages are unique and display both phenotypical and functional plasticity enabling them to perform a wide range of functions ranging from immune to physiological roles including tissue homeostasis. During pathogen encounter and other inflammatory assaults, these cells get exposed to various stresses where cells require high metabolic index to encounter the same while maintaining optimum immune response. M1 macrophages are highly metabolic in nature which utilized glucose as their main source of energy. These macrophages produce many mediators *via* glycolytic pathways and promote Warburg reactions which directly or indirectly help in controlling infection while impacting on the phenotypic plasticity of inflammatory macrophages in diseased conditions (30, 31). Here, we demonstrate that IAPs are novel proteins which can dictate both functional plasticity and metabolic activities of macrophages during both bacterial encounter and inflammation-related stress conditions. Pathogens like *Chlamydiae*, which depend upon IAPs, hijack the plastic nature and mitigate immune and metabolic activities in inflammatory macrophages for their own benefits. Under certain pathological circumstances manifested by high IFNγ, these bacteria undergo persistency. This is accompanied with catabolism of tryptophan which is decisive for the metabolic activity of macrophages, deficiency of which is known to enhance immune tolerance directly and reduced mitochondrial functions indirectly. IFNγ-mediated persistency *via* activating indoleamine 2,3-dioxygenase deplete cellular depot of tryptophan thus further contributing for the induction of RB conversion to EB, the form in which the bacteria resist various antibiotic treatments. Similarly, upregulation of MAPK14 by *Chlamydiae* infection in host cells seems to be another mechanism by which these bacteria could disrupt effecter phenotype of macrophages (32). Upregulation of MAPK14 proteins inhibit the expression of iNOS while upregulating the expression of Arginase-1 proteins (33, 34) which are associated with M2 polarization of macrophages during parasitic infection (35) and bacterial sepsis (36). Other than this, upregulation of MAPK (particularly p38MAPK) promotes the shedding of TNFR1 and it is quite likely that in the absence of TNFR1, either upregulation or shift toward TNFR2-associated pathways like PI-3K/pAKT/p38MAPK pathways could also have contributed to M2 polarization of effector macrophages. In similar lines, internalization of TNFR1 in infected macrophages could have contributed for infection mediated blocking of NO in TNF-primed macrophages. During persistency, these bacteria release type 3 secretions like HSP60 which interact with various signaling or metabolic pathways including aerobic respiration and oxidative metabolism in the host cells including macrophages (35, 37, 38). HSP60 is responsible for majority of immunopathological damage during the infection (39), and among those stabilization/upregulation of HIF-1 (32, 36) in the infected host cells is one major mechanism by which these bacteria could suppress Th1 immune response and regulate the activity of iNOS+ M1 macrophages (32). Hypoxic environment is known to jeopardize the anti-bacterial defense potential of macrophages (40) and inhibits mitochondrial respiratory chain functions and impair metabolic activities which are essential for killing of several bacterial pathogens including *C. pneumoniae* (41, 42) by professional phagocytes like macrophages. Other than this, HSP60 from *C. pneumoniae* is also responsible for foamy transformation of inflammatory macrophages (43, 44), which are having unusual pro-inflammatory signaling due to constitutive upregulation of PPAR signaling and accumulations of certain lipids like cholesterol and/or sphingolipids in macrophages (20, 45–48). Therefore, it is quite possible that accumulated HSP60 in infected XIAP KO lung could have skewed M2 polarization in XIAP KO-infected macrophages, rendering them metabolically inactive and poor in anti-bacterial defenses.

Although several cells of immune system other than macrophages like granulocytes, neutrophils and B cells constitute the innate immunity, macrophages are capable of controlling T cells immune response and thus contribute to adaptive immunity as well (49). Therefore, in this study, we mainly analyzed the impact of birinapant on macrophages particularly. However, we cannot overlook the impact of birinapant on other immune cells also in the course of reducing inflammatory reactions in animal models. From our data, it is also clear that increase in the metabolic activity of host, other than immune stimulation would certainly be paramount for the effective management of persistent infection and associated idiopathic diseases. In summary, our data demonstrated birinapant-mediated immune metabolic programming of M1 macrophages during their encounter with bacteria and/or sterile inflammatory stimulus. Thus, our study also suggests that the birinapant could be used as a drug candidate for controlling infection/inflammatory diseases which depend upon the macrophages.

## Ethics Statement

All animal experiments were performed as per the guidelines laid down by institutional Animal ethical committee and approved by Institutional Animal ethical committee approval (UH/IAEC/HP/2014-I/21).

## Author Contributions

VN, AM, LS, VBM and HJM conducted the experiments. VN, SY, RP, MBM and HJM analyzed the data. VN and SY designed the figures. RM, SK, and TR contributed to the research tool. HP conceived the idea. VN and HP wrote the paper.

## Conflict of Interest Statement

The authors declare that the research was conducted in the absence of any commercial or financial relationships that could be construed as a potential conflict of interest.

## Footnotes

^1^http://vortex.cs.wayne.edu/projects.htm.

^2^http://baderlab.org/GeneSets.

## Supplementary Material

Figure S1 **(A–E)** cIAP1 proteins regulate TNF-mediated immune response in macrophages. mRNA from cIAP-1 WT and KO macrophages stimulated with TNF for 24 h duration was analyzed by whole mouse genome 44 k microarray Kit, Agilent technology (Cat No—G4122F, Chip ID no. 014868). Pathway-Express (PE) was used to map differentially expressed genes and Gene Spring 12.6.1 software (Agilent Technologies, Santa Clara, CA, USA) was used to prepare corresponding heat map. Pathways that were up/downregulated during TNF induction and or CIAP-KO were identified using previously developed method on Gene-Set enrichment analysis. Genes Sets for mouse were downloaded from Bader lab and were obtained by translation of human counterparts using orthologous relationship from homologene database. **(A)** The representation of up- and downregulated total number of genes by cIAP-1 in macrophages in the form of Venn diagram while **(B)** are the gene sets from Reactome_3 pathways, **(C)** from BP_3.5 pathways, and **(D)** from NCL_2 pathways and gene ontology process from Mouse Genome Database (MGD).

Figure S2 *Chlamydia pneumoniae* infection induced sTNFR1 shedding in TNF-stimulated HeLa. cIap1 KO upon infection with *C. pneumoniae* and further stimulation with TNF was quantified at defined time intervals. Normal, cIAP1 shRNA transfected, and vector control stable HeLa cells clones were infected with *C. pneumoniae* for one infection cycle and soluble/shed TNFR1 were quantified in their culture supernatant by sandwich ELISA method (Bio-Sources). Data are SE from two independent ± represented as picograms per milliliter of sTNFR1 released experiments. Statistical analysis was conducted using two-way ANOVA followed by Bonferroni posttest (**p* < 0.05; ***p* < 0.01; ****p* < 0.001).

Figure S3 *Chlamydia pneumoniae* interfere with iron mediate nitric oxide generation in macrophages. CD11b^+^/Gr-1ˉ peritoneal macrophages from WT, cIAP KO mice **(A)** and xIAP KO mice **(B)** were infected with C. pneumonia and stimulated with SNP (sodium nitroprusside), which release NO (nitric oxide) in iron-dependence manner. NO titers were quantified in their culture supernatant at indicated time interval. Data represented as mean of micrometers of NO ± SE and statistical analysis was conducted using two-way ANOVA followed by Bonferroni posttest (****p* < 0.001).

Figure S4 IAP deficiency promotes *Chlamydia pneumoniae*-mediated interference of macrophage stimulation and their metabolic activity. **(A)** WT, cIAP KO, and xIAP KO macrophages were infected with *C. pneumoniae* and the fate of altered metabolic activity in these macrophages at specified time points was analyzed by MTT assay. In similar lines, *C. pneumoniae* infection mediated altercations in metabolic activity of IFNγ **(B)**, TNF **(C)**, and SNP **(D)** stimulated macrophages were also quantified. Statistical analysis was conducted using two-way ANOVA followed by Bonferroni posttest (****p* < 0.001).

Figure S5 Smac mimicry enhances *Mycobacterium smegmatis/Escherichia* coli infection-induced NO production macrophages. RAW 264.7 macrophages were infected with *M. smegmatis/E. coli* and after 1 h postinfection extracellular bacteria washed off and cells +BP.γ or BP and combination of IFNγ co-stimulated either with IFN NO production by these macrophages was quantified in cell supernatant at 24 h **(A)** and 48 h **(B)** postinfection. Bacterial counts (colony-forming unit per milliliters) were measured in macrophage cell lysates at 24 h **(C,D)** and 48 h postinfection **(E,F)**. Data are represented as mean ± SE of three independent experiments in triplicates and statistical analysis was conducted using Student’s *t*-test (**p* ≤ 0.05, ***p* ≤ 0.01, ****p* ≤ 0.001).

Figure S6 *Mycobacterium smegmatis and Escherichia coli* do not interfere with IFNγ-induced iNOS production in macrophages. RAW 264.7 macrophages were infected with *M. smegmatis and E. coli* and stimulated either with/without IFNγ and/or BP and expression of iNOS in these whole cell lysates were analyzed by Immunoblotting at 24 h **(A,B)** and 48 h **(C,D)** postinfection. Data shown are the representative of three independent repeats and the densitometry analysis of the representative blots was done using ImageJ and presented as ratio of proteins with actin.

Figure S7 *Leishmania donovani* skew M2 phenotype in macrophages and interfere with IFNγ-stimulated generation of NO in macrophages. RAW 264.7 macrophages were infected with *L. donoveni* (MOI-5) and, after 4 h, the extracellular parasites were washed off. Macrophages were stimulated with IFNγ and treated with BP while maintaining untreated cells as controls. NO titres were quantified in cell culture supernatant at 24 and 48 h postinfection. Data presented are mean of three independent experiments done in triplicates and statistical analysis was conducted using Student’s *t*-test (**p* < 0.05; ***p* < 0.01; ****p* < 0.001).

Figure S8 IAP regulates hypoxic-mediated sterile inflammatory responses in iNOS+ macrophages. RAW macrophages were cultured in presence of CoCl2 and the expression of iNOS and HIF1 proteins was analyzed by immunofluorescence *(A)* for confirming M1 polarization of these macrophages. RAW264.7A murine MΦ were stimulated with Th1 stimuli viz LPS or IFNγ and both LPA and IFNγ with and without CoCl2 and Brinapant and cultured for indicated time points. **(B)** NO titer was quantified cell culture supernatants and the data were represented as a mean micrometers of NO ± SEM. Statistical analysis was conducted using one way ANOVA followed by Bonferroni posttest (*p < 0.05; **p < 0.01; ***p < 0.001). **(C)** The cultures mentioned under **(B)** were lysed and analyzed for various M1 and M2 effector proteins, IAPs and signaling markers by western blotting. **(D)** Densitometry quantification of western blots from three independent repeats were analyzed by Image J and the data were plotted as mean of protein/actin ratio **(E)**. To monitor the intracellular signaling, important metabolic signaling component activation was observed using PathScan Intracellular Signaling Array Kit from Cell Signaling Technology. Images were analyzed by using ImageJ software and mean densitometry values were plotted in terms of relative expression. Statistical analysis was conducted using two-way ANOVA followed by Bonferroni posttest (**p* < 0.05; ***p* < 0.01; ****p* < 0.001).

Figure S9 IAP regulates angiogenic programming in iNOS+ macrophages. RAW264.7A murine MΦ were stimulated with Th1 stimuli viz LPS or IFNγ and both LPS or IFNγ with and without VEGF and Brinapant and cultured for indicated time points. **(A)** NO titer was quantified in the cell culture supernatant and the data was represented as the mean micrometers of NO ± SEM. Statistical analysis was conducted using one-way ANOVA followed by Bonferroni posttest (**p* < 0.05; ***p* < 0.01; ****p* < 0.001). **(B)** The cultures mentioned under **(A)** were lysed and analyzed for various M1 and M2 effector proteins, IAPs, and signaling markers by western blotting. **(C)** Densitometry quantification of western blots from three independent repeats were analyzed by Image J and the data were plotted as mean of protein/actin ratio. **(D)** To monitor the intracellular signaling, important metabolic signaling component activation was observed using PathScan Intracellular Signaling Array Kit from Cell Signaling Technology. Images were analyzed by using ImageJ software and mean densitometry values were plotted in terms of relative expression. Statistical analysis was conducted using two-way ANOVA followed by Bonferroni posttest (**p* < 0.05; ***p* < 0.01; ****p* < 0.001).

Figure S10 IAP control hypoxia induce polarization in Th1 committed CD11b+ mouse peripheral macrophages. CD11b+ peritoneal macrophages were purified by MACS-based method and cultured o/n and stimulated with VEGF and CoCl2 in presence of Brinapant. NO titres were quantified in both naïve **(A)** and IFNγ-stimulated **(B)** macrophages. Shown here is the mean micrometers of NO ± SEM from three independent experiments.

Figure S11 IAP proteins re-program metabolic activity in both naive and INOS+ macrophages during macrophage polarization. RAW264.7A murine MΦ either naïve **(A)** or stimulated with Th1 effector cytokine IFNγ **(B)**; were treated with metformin in both normal and high glucose condition and in presence and absence of Brinapant and cultured for indicated time points. NO titer was quantified and the data represent the mean micrometers of NO ± SEM. Statistical analysis was conducted using two-way ANOVA followed by Bonferroni posttest (**p* < 0.05; ***p* < 0.01; ****p* < 0.001).
