# Supplementary material for: Inhibitors of Apoptosis Protein Antagonists (Smac Mimetic Compounds) Control Polarization of Macrophages during Microbial Challenge and Sterile Inflammatory Responses
Source: Front Immunol. 2018 Jan 9;8:1792. doi: 10.3389/fimmu.2017.01792 (PMC5767188; doi:10.3389/fimmu.2017.01792)
Supplement: Figure S1 — (A–E) cIAP1 proteins regulate TNF-mediated immune response in macrophages. mRNA from cIAP-1 WT and KO macrophages stimulated with TNF for 24 h duration was analyzed by whole mouse genome 44 k microarray Kit, Agilent technology (Cat No—G4122F, Chip ID no. 014868). Pathway-Express (PE) was used to map differentially expressed genes and Gene Spring 12.6.1 software (Agilent Technologies, Santa Clara, CA, USA) was used to prepare corresponding heat map. Pathways that were up/downregulated during TNF induction and or CIAP-KO were identified using previously developed method on Gene-Set enrichment analysis. Genes Sets for mouse were downloaded from Bader lab and were obtained by translation of human counterparts using orthologous relationship from homologene database. (A) The representation of up- and downregulated total number of genes by cIAP-1 in macrophages in the form of Venn diagram while (B) are the gene sets from Reactome_3 pathways, (C) from BP_3.5 pathways, and (D) from NCL_2 pathways and gene ontology process from Mouse Genome Database (MGD). [file Data_Sheet_1.zip › Data.PDF]

Gene	Wild type control	Wildtype +TNF	CIAP-1KO control	CIAP-1 KO +TNF
	17801.8	17729.8	19838.66667	16220.86667
0610005C13Rik	8156	10434	14189	15258
0610007N19Rik	8462	40695	11274	6446
0610007P14Rik	3175	4329	1495	628
0610008F07Rik	10617	10179	14849	14502
0610009B14Rik	4810	39825	8885	12250
0610009B22Rik	25359.5	27096	21770.5	20409.5
0610009K14Rik	24087	25148	27258	26126
0610009L18Rik	11531	7619	10272	4493
0610009O20Rik	21378	18924.5	16702	37955
0610010F05Rik	12377.66667	11652	21670.33333	21903.33333
0610010K14Rik	11567	13493	14216	12895
0610011F06Rik	7084	14294	13702	13846
0610012E21Rik	28705	27620	32350	393
0610012G03Rik	20178.5	21696.5	19115.5	20647
0610012H03Rik	1396	12749	5812	14828
0610025J13Rik	21891	23532.5	19177	3983
0610030E20Rik	12776.33333	9741	34662.66667	32199
0610037L13Rik	9964.5	14189	10067.5	11208
0610037L18Rik	31550	33317	34836	33428
0610039H22Rik	22310.5	13974.5	15772	22019
0610039K10Rik	14986	15013	18829	12545
0610040A22Rik	3782	7583	13123	11222
0610040B10Rik	29838	26927	31383	14881.5
0610040J01Rik	13498	37910	18481	402
0610042G04Rik	40745	2624	3400	5899
0710001D07Rik	14545	18790	19159	19829
1100001G20Rik	39983	20323	32209	12131
1110001J03Rik	37490	7484	4211	2075
1110001M07Rik	41000	26146	5616	6520
1110002J07Rik	16104	37958	6919	22293
1110002L01Rik	29051	30445	32539	30985
1110003F10Rik	9414	6431	9058	16130
1110004E09Rik	26196	36286	34724	38034
1110004F10Rik	16588	16963	21896	21699
1110006G14Rik	27972.5	33470	31404.5	16645.5
1110006O24Rik	40796	37170	39425	39305
1110007C09Rik	36223	38025	41083	39070

1110008E08Rik	3951	6678	3647	10143
1110008F13Rik	31050	37780	35065	36386
1110008L16Rik	14420	16590	17540	17315
1110008P14Rik	855	3365	4930	4259
1110012L19Rik	29605	40802	35944	40641
1110013I04Rik	29023	30324	32354	30542
1110014L15Rik	2797	30566	4196	9831
1110015O18Rik	687	35219	40025	2825
1110017D15Rik	13730	19832	15979	16894
1110018F16Rik	14177	5318	12408	8660
1110018N20Rik	8823	40950	13274	9172
1110019B22Rik	15721	17126	23154	23984
1110019B24Rik	11875	2496	15438	3744
1110020A10Rik	27581	29053	30750	33067
1110020A21Rik	5714	2432	6115	48
1110020C17Rik	29984	31602	33864	32130
1110025P21	30821	33164	32251	34915
1110032A03Rik	20598.5	2169	1086	38916.5
1110032F04Rik	6854	7374	11655	12495
1110032L06Rik	25167	32186	28890	222
1110033F14Rik	26583	27994	2713	28080
1110034G24Rik	14016	15674	14864	11876
1110035E04Rik	17009	9730	21170	21788
1110035H17Rik	33160	31654	32456	13640.5
1110035M17Rik	15134	11247	12132	9094
1110037F02Rik	16459.33333	15345	13817	15013.66667
1110038B12Rik	19669	23337	22086	22245
1110038F14Rik	38520	38581	39398	40308
1110046J04Rik	15224	13716	20485.5	17627.5
1110051M20Rik	26678.25	29669.75	20925	31533.25
1110054M08Rik	18138	21214	19509	21271
1110059E24Rik	20740.5	15175	16175	18947.5
1110059G10Rik	27225.5	18242	17361	13728
1110065M07	23606	23539	21623	18647
1110065P20Rik	26862	34732	30091	31252.5
1190001L17Rik	38734	4074	4313	3016
1190001M18Rik	37336	35223	28946	28042
1190002F15Rik	35488	28006	33971	34078
1190002N15Rik	35807.33333	22428.66667	25601.66667	23483.66667

1190003J15Rik	26487.5	9353	12172	11515
1190003K10Rik	12635	17027	21923	26153
1190005I06Rik	14869	12520	16225	7574
1200007C13Rik	27205	39006	31155	6484
1200014J11Rik	15755	15697	27906.33333	28844.66667
1300002K09Rik	9266.5	22087	3267	20060
1300015D01Rik	19484.5	24410.5	25118.5	29516
1300017J02Rik	24134	6507	18981.5	19880.5
1500002C15Rik	3085	39775	3013	40298
1500002I01Rik	33098	34758	36733	34928
1500002K03Rik	36601	18	3462	9684
1500002O10Rik	24977	26129	27843	2393
1500006F24	30	19416	34651	17999
1500009C09Rik	16684	18968	24030	24474
1500009L16Rik	5167	38406	35755	7689
1500010C09Rik	38378	38007	4910	39941
1500011B03Rik	2250	2127	39557	606
1500011K16Rik	33112	887	38429	38969
1500012F01Rik	22847	26691	28809	28934
1500015L24Rik	6220	7244	12348	11151
1500015O10Rik	23215	24316	26108	25051
1500016L03Rik	12434	9981	15482	16881
1500026H17Rik	30176.66667	24411.33333	29125.66667	24597.33333
1500032F14Rik	39749	33331	40287	41083
1500035N22Rik	5129	36520	2638	12493
1500037F05Rik	2476	37929	3201	34982
1520401A03Rik	33576	39023	3484	11083
1600002D24Rik	5395	4272	14501	12117
1600002H07Rik	22315.5	24867	22546.5	22965
1600002K03Rik	22960	30492	25935	26988
1600012H06Rik	22511	24013	24669	27371
1600012P17Rik	3656	7634	14181	21550
1600014C10Rik	15862	17601.25	12881.5	13004.5
1600014C23Rik	36481	40214	1922	39210
1600015I10Rik	16116	15937	20495	20320
1600017P15Rik	23900	24994	26850	25884
1600020E01Rik	40895	36399	35281	32360
1600025M17Rik	19172	20856	34411	26907
1600027J07Rik	23762	24843	34814	2325

1600029I14Rik	29196	1487	3239	5262
1600029O15Rik	7434	10253	8804	9218
1700001C02Rik	19026.5	22255.5	35453.5	35957.5
1700001C19Rik	1616	11623	19047.5	11315.5
1700001F09Rik	5617.5	31329	27437	23638.5
1700001G17Rik	34794	28800	2774	26157
1700001J03Rik	33256	29556	248	39057
1700001J04Rik	21658.5	24337	6990	8859.5
1700001J11Rik	20135	30853	17333	30341
1700001K19Rik	21076	18717	23073	22155
1700001K23Rik	8358	9718	14571	15561
1700001L05Rik	24607	23618.5	28682	28945.5
1700001L19Rik	38629	30866	38170	40029
1700001N15Rik	33846	26376	28548	27401
1700001O22Rik	28056	29003	32692	33531.5
1700001P01Rik	11226.5	15272.5	13823	15913
1700003E16Rik	16157	14070	19200	17628
1700003F12Rik	3523	10337	6698	8493
1700003F17Rik	26317	27681	26294	25317
1700003G18Rik	24590	23081	36983	32052
1700003H04Rik	29403	33549	35284	19631.5
1700003M02Rik	27324	28683	30500	29195
1700003O08Rik	2402	2001	33169	35483
1700003O11Rik	1181	7417	20959	24832
1700006A11Rik	20656	27410	37770	35643
1700006E09Rik	25416	16488.5	19485.5	37424
1700006J14Rik	24508	20187	24991	24021
1700007B14Rik	32350	33602	35953	33982
1700007G11Rik	32820	34280	36817	9013
1700007H22Rik	35379	38243	6137	2044
1700007J24Rik	8041	18833	8123	10523
1700007K09Rik	11426	13300	21248	22860
1700007K13Rik	17609.5	16497	19792.5	18306
1700008A07Rik	18617	19340	25655	31084
1700008H02Rik	30340	32101	32908	31403
1700008O03Rik	27431	25804	37251	29355
1700008P02Rik	8022	3638	34951	5962
1700008P20Rik	25796	7593	31660	5818
1700009J07Rik	7784	25487	12959	13651

1700009N14Rik	16647	22038	33421	34454
1700010B08Rik	21169	20511	20233	18116
1700010B13Rik	13257	12265	9729	5948
1700010D01Rik	38303	1206	7468	4596
1700010H22Rik	20669	24095	33625	37030
1700010I14Rik	22507	22251	25320	24847
1700010L04Rik	32134.5	14043	14882.5	18499
1700010N08Rik	27743	9889	30185	35858
1700011A15Rik	36840	40175	3914	4358
1700011B04Rik	29869	31473	33793	32059
1700011E24Rik	7334	2558	13625	5565
1700011H14Rik	30866	905	5540	40770
1700011I03Rik	24187	25322	27392	32914
1700011J10Rik	37213	35546	34686	31534
1700011L22Rik	31559	28609	34725	33147
1700012A03Rik	34684	35150	968	45
1700012B07Rik	27505	38577	37311	37365
1700012B09Rik	7101	11689	10140.5	6103.5
1700012B15Rik	15505	12944	11290.5	12416
1700012C08Rik	28073	29377	31413	34544
1700012D14Rik	11414.5	9710.5	12104	26397
1700012L04Rik	23238.5	23842.5	27252	19105
1700012P22Rik	23213	17752.5	24608	14192
1700013D24Rik	7698	10263	6594	7619
1700013F07Rik	38540	6193	4505	4784
1700013G24Rik	39623	40038	3530	2164
1700013H16Rik	37257	26080	27939	26920
1700014B07Rik	5832	11050	15410	11893
1700014D04Rik	29218	30796	31853	40175
1700014D06Rik	26726	27160	28736	31053
1700015E13Rik	34237	32163	34482	40769
1700015P03Rik	30952	32298	33966	32285
1700016A09Rik	4729	2734	10971	10779
1700016B15Rik	7535	11590	12678	15548
1700016C15Rik	33906	30092	29561	28583
1700016D06Rik	30749	26906	28120	27105
1700016D08Rik	7696	12454	10659	14735
1700016D18Rik	20889	24007	39244	1429
1700016F12Rik	32575	34374	36681	34444

1700016G14Rik	9923	10874	23232	21814
1700016G22Rik	30756	32445	34191	32436
1700016H13Rik	18613	21740	16921	18171
1700016J18Rik	9235	23518	465	18695
1700016K19Rik	18513	18772	22174	22693
1700016M24Rik	22369	23170	24049	26531
1700017B05Rik	16266.5	12150	15999.5	14517
1700017D01Rik	33413	1079	211	4886
1700017G19Rik	26974	28064	30704	29336
1700017I07Rik	5503	26306	37641	27439
1700017N19Rik	25246	26363	28377	39739
1700018A04Rik	654	37496	7689	5792
1700018A14Rik	12017	14656	13421	20566
1700018B08Rik	39617	9953	16685	15508
1700018B24Rik	20271	19379.5	19901	24390.5
1700018C11Rik	37539	35849	40724	33087
1700018F24Rik	9976	15383	16844	17277
1700018G05Rik	18770	17062	16541	15652
1700018M17Rik	37276	36305	34225	29960
1700019A02Rik	21610	24818	680	875
1700019B03Rik	17125	18780	22971	23675
1700019D03Rik	10416	13118	19404	19368
1700019E08Rik	36332	4831	13475	14217
1700019G17Rik	13089	12502.66667	21099.66667	21868
1700019H22Rik	27493	28980	30613	34592
1700019J19Rik	24211	26025	40633	8046
1700019L03Rik	16948	19170	16405	17895.5
1700019M22Rik	27483	38258	31977	30299
1700019N19Rik	12439.5	15960	13846	16124
1700019O17Rik	14166	18196	19574	20276
1700020A23Rik	19523	15692	31458	30237
1700020D05Rik	12385	17948	17008	17727
1700020G17Rik	1599	32576	32712	39106
1700020I14Rik	19412.66667	17458.66667	19471.33333	17272.66667
1700020L24Rik	40779	3745	5425	5595
1700020N01Rik	15768	21767	22506	27581
1700020N15Rik	33247	35320	3161	2326
1700020N18Rik	13117	14169	4723	40287
1700021A07Rik	1074	11824	17506	23470

1700021F05Rik	34139	39372	37408	37658
1700021F07Rik	18266.5	24731	27628.5	37569.5
1700021J08Rik	9144	9553	11438	10209
1700021K19Rik	11770.5	30086	17793.5	31146
1700021O21Rik	1236	20703	26008.5	16210
1700021P04Rik	4820	1427	8679	8171
1700022A21Rik	6386	13584	15970	14662
1700022A22Rik	27307	41084	2232	2741
1700022E09Rik	31869	11449	35772	33864
1700022F17Rik	30686	25890	28041	35896
1700022I11Rik	14919	19450	27812	24644
1700023F06Rik	15998	15731	17395	17678
1700023L04Rik	1083	19783	7543	12168
1700024B05Rik	10533	14410	18683	15189
1700024G13Rik	21530	23235	37437	37245
1700024I08Rik	26031	27527	29058	27753
1700024J04Rik	30308	17927	3126.5	31025.5
1700024P04Rik	6960	9283	18685	17903
1700024P16Rik	30766	27977	2711	39459
1700025F22Rik	20551	28365	33042	35658
1700025F24Rik	21166	18333	20295	24120
1700025G04Rik	25514.66667	11808.66667	11171	10130.33333
1700025H01Rik	1520	2528	9821	16587
1700025K24Rik	23549	24694	26234	25202
1700025L06Rik	8601	3456	6238	6985
1700025O08Rik	22412	24572.5	24879.5	23354
1700026D08Rik	7907	16095	23926	5777
1700026D11Rik	15743	16432	15415	14155
1700026J12Rik	9293.5	26383.5	30574.5	17326.5
1700026L06Rik	29651	25544	25490	21227
1700026N04Rik	11185	9955	18146	17994
1700027A13Rik	29650	31170	32203	30770
1700027J19Rik	17434.66667	21734.66667	18663.66667	12464.66667
1700028E10Rik	23265.5	20486.5	24398.5	29826.5
1700028J19Rik	34307	27427	22936	26909
1700028K03Rik	35932.5	430	14511.5	32793.5
1700028N14Rik	4397	6445	13985	14983
1700028P14Rik	15322	10804	12032	13867
1700029E06Rik	25153	26387	28191	27042

1700029F12Rik	24626	25853	25342.33333	13977
1700029H14Rik	16404	6585	9795	36433
1700029I01Rik	11919.83333	24718	25266.33333	16784.83333
1700029I15Rik	13740	23270	14120	24908
1700029J07Rik	17825	13667	17477	10106
1700029M20Rik	23276	26269.5	21715	23169.5
1700029N11Rik	31333	7557	17118	17465
1700029P11Rik	13142	16819	16857	16611
1700030C10Rik	3047	30800	30804	29359
1700030J22Rik	12873	11629	13368	12554
1700030K09Rik	22548	19198	21776.5	21174.5
1700030L20Rik	24653	27034	28242	36717
1700030L22Rik	24734	39140	617	35646
1700030N03Rik	12289.5	29337.5	35771.5	19109.5
1700031A10Rik	2443	4424	11731	12703
1700031C06Rik	10182	20515	25946	27263
1700031F05Rik	8066	12589	15785	12181
1700031F10Rik	25555	35685	28973	27758
1700031L13Rik	29483	23103	38299	25869
1700031M16Rik	35695	6711	438	4097
1700034E13Rik	29878	35922	32868	35780
1700034G24Rik	27072	34398	30361	28928
1700034H15Rik	40107	40279	7706	4581
1700034I23Rik	26557	27891	29928	28533
1700034J04Rik	36060	20825	34540	32748
1700034J05Rik	31038	16253	16461.5	19754
1700034O15Rik	38565	40258	8397	9619
1700036A12Rik	36531	929	6588	7413
1700037C18Rik	21172	24715	7563.5	25690
1700037H04Rik	19315	21061	19918	23389
1700037N05Rik	25042	26243	28985	41047
1700038C09Rik	29264	3357	32479	30919
1700039M10Rik	37742	12815	33170	31667
1700039O17Rik	27425	28948	29838	28558
1700040A12Rik	13776	15845	18583	19446
1700040D17Rik	26773	27883	30213	35017
1700040F17Rik	38733	1608	33140	9696
1700040L02Rik	29755	35193	29607	30377
1700041B01Rik	8205	10853	39812	8038

1700041C23Rik	24086	27654	6604	1979
1700041G16Rik	21660	23022	23695	23645
1700041M19Rik	26774	28175	29902	35392
1700042D02Rik	33056	34631	39045	37581
1700042D18Rik	32882	34515	650	35756
1700042G07Rik	22003	23514	27648	27882
1700043A12Rik	4651	16788	34631	396
1700045I11Rik	15021	13276	16990	20805
1700045I19Rik	16481	15993	19411	19959
1700046C09Rik	28278	29788	31261	29909
1700047A11Rik	29048	31015	36495	29241
1700047E10Rik	31916	2241	35174	33700
1700047K16Rik	37466	31654	33012	31435
1700048B10Rik	28544	30076	32136	39890
1700048C15Rik	2081	7784	13053	6406
1700048G13Rik	34254	30697	3983	37467
1700048O20Rik	778	40967	6283	6958
1700049E15Rik	35245	41004	33467	36228
1700049E17Rik1	25442.33333	24585.33333	27561.33333	21707.33333
1700049G17Rik	23938.5	37862	22847.5	20904.5
1700049J03Rik	10761	8348	6211	13878
1700049L16Rik	33857	9349	38206	10316
1700052I22Rik	38239	35085	37816	38689
1700052K11Rik	8583	11740	11025	9956
1700054H16	8884	12480	12760	12343
1700054M17Rik	7666	7970	8071	1177
1700054O19Rik	26663	28272	28884	27862
1700055D18Rik	26987	28562	29394	28227
1700056E22Rik	28555	29330	36330	34713
1700056I18Rik	26878	28340	34374	39036
1700056N10Rik	17243	11166	14699	13968
1700057G04Rik	2829	38899	34511	33154
1700057H21Rik	24507	12182	8509	12167
1700061G19Rik	15758	10412.5	6780	6510
1700061I17Rik	32367	20208.5	17722.5	24799.5
1700063A18Rik	31222	32875	34590	32829
1700063H04Rik	278	4634	7048	8101
1700063K16Rik	27902	29270	31443	29936
1700065D16Rik	23531	15252.66667	12673	17112.33333

1700065I16Rik	22467	18990	25692	28100
1700065O20Rik	26305	27326	30506	29075
1700066C05Rik	11308	20838	9661	10245
1700066D14Rik	28041	29454	31371	29868
1700066J03Rik	5345	40474	3486	36683
1700066J24Rik	15	2656	673	2932
1700066M21Rik	7188	12548	10052	11834
1700067K01Rik	8235	10997	12575	13863
1700067P10Rik	34797	38183	37581	37405
1700069L09	24765	24365	28881	23697
1700069L16Rik	13918	15339.5	19299.5	18396
1700069P05Rik	28720	30216	31724	30270
1700072F12Rik	38284	39450	4247	1673
1700072H12Rik	3063	5000	8479	14980
1700073E17Rik	26905	28468.5	34602	34184.5
1700074P13Rik	35225	38387	6356	7185
1700080E11Rik	8092	20651.5	30107.5	28866.5
1700080F18Rik	32801	34592	36093	34044
1700080G18Rik	11179	14290	13802	13917
1700080O16Rik	28006	5358	4980	40781
1700082M22Rik	26150	27432	29207	28007
1700083H02Rik	16962	16928	24146	39895
1700084C06Rik	18469	20201	23814	23039
1700084E18Rik	2158.5	5569.5	7549.5	7019.5
1700084J12Rik	23867	35056	39840	2195
1700084K02Rik	40518	6747	36925	655
1700084M14Rik	15202	34660	32978	35133
1700084P21Rik	32174	33783	36038	34157
1700085B03Rik	23167	24251	26116	25034
1700086D15Rik	26844	28086	30841	29382
1700086L19Rik	17487	19038	20413	19697
1700086P04Rik	14145	32235	24811	28696
1700087M22Rik	1539	1544	40542	38746
1700088E04Rik	38459	40955	2499	4316
1700092C02Rik	14695	16600	28229	27015
1700092C17Rik	15308	18259	21039	22432
1700092E16Rik	19922	20153	23921	22053
1700093C20Rik	31488	24520	26197	25147
1700093J21Rik	950	3028	10748	12214

1700093K21Rik	21087	21844.5	23018	23896
1700094I16Rik	11895	11281	13371	14612
1700094J05Rik	27035	28626	33668	28125
1700095A21Rik	1446	37384	37668	40894
1700095B10Rik	24060	25206	26965	26024
1700095J03Rik	32665	34422	35994	40765
1700096K18Rik	27295	25164	24482	22231
1700097M23Rik	27695	11479	481	387
1700100I10Rik	31431	33176	35471	740
1700101C01Rik	2782	1074	10903	8456
1700101I11Rik	6364	6038	8139	9127
1700101I19Rik	24101	30426	35761	36258
1700102F20Rik	39424	37519	116	4329
1700102J08Rik	36160.5	33612.5	20380.5	26756
1700102P08Rik	5298	2474	8557	8416
1700104B16Rik	19171	24612	23045	27479
1700108F19Rik	34735	32641	30399	28983
1700108J01Rik	32250	34702	34720	32883
1700109F18Rik	34069.5	36263.5	23703.5	11432.5
1700109G14Rik	36051	26140	28403	27236
1700109H08Rik	30830	20299	23486	23019
1700110I01Rik	31565	33238	34746	39701
1700111E14Rik	30649	7232	27009	25938
1700111N16Rik	29957	31524	32996	31482
1700112E06Rik	14794.25	20918.75	22795.75	21102
1700112J16Rik	33314	37727	26504	25385
1700112M01Rik	15981	16829	21240	14486
1700112M02Rik	27774	37039	29834	35813
1700113A16Rik	21812	27266	23307	32911
1700113H08Rik	7007	10150	14199	13476
1700116B05Rik	38271	1274	8789	9869
1700120B22Rik	31270	32933	34568	32800
1700120E14Rik	20056	22704	32758	32084
1700120K04Rik	8735	13563	14006	15843
1700121C10Rik	32563	34872	715	2458
1700121I08Rik	32670	34182	37812	41154
1700121L16Rik	24772	28939	40624	258
1700122C19Rik	32631	33866	35786	33881
1700122O11Rik	38112	1972	8097	8873

1700123I01Rik	20081	30773	25856	33604
1700123K08Rik	3504	38610	4438	5187
1700123L14Rik	28495	38306	11029	40534
1700123O20Rik	14097	14367	11819	15867
1700125G22Rik	33622	40984	5065	7059
1700125H20Rik	30529	32082	38245	40941
1700128A07Rik	27615	29093	30438	29231
1700128E19Rik	21691	21180	24879	24661
1700128F08Rik	32456	28461	11287	9768
1700129C05Rik	18884.5	18602	20931.5	19155
1.7E+25	35930	1332	36282	38186
1810007D17Rik	26896	28136	30800	38071
1810008B01Rik	26438	37619	30141	38850
1810008I18Rik	23738	24902	26737	31598
1810009A15Rik	36509	40677	37973	39005
1810009J06Rik	34174	38344	35148	35329
1810009N02Rik	23172	26622	25191	29730
1810010H24Rik	19826	25465	20814	20994
1810010K12Rik	1084	28171	30680	29225
1810011H11Rik	27072.5	6673	25175	19764.5
1810011O10Rik	14903	24052.5	30301.5	19261
1810013D15Rik	21918	25006	30579	32510
1810013L24Rik	14305.25	5799	5117.75	9477.5
1810014B01Rik	28876	12219.5	28899	30184
1810018F18Rik	32701	34201	36929	34979
1810022K09Rik	38827	41077	38307	762
1810024B03Rik	4999	7199	8471	4712
1810026B05Rik	11474	11226	13388	10992
1810026J23Rik	25054	28462	25224	32121
1810027L02Rik	13439	16377	17109	18475
1810030J14Rik	33932	30465	34683	30689
1810030O07Rik	16187	13745.5	11235.5	12437
1810032O08Rik	13696	13119	10983	8164
1810034E14Rik	22218	24526	22238	21268
1810037I17Rik	13465.5	20717	16531	18151.5
1810037O21Rik	26443	27969	40306	28392
1810041L15Rik	29923	116	40744	38330
1810043G02Rik	22196	27465	15701.5	17213.5
1810043H04Rik	1548	6337	10731	10172

1810046K07Rik	25763	35608	33167	40422
1810053B23Rik	24629	25777	27874	26673
1810055G02Rik	12144	12550	11437	14700
1810058I24Rik	23177.5	16471	17887	16906
1810058N15Rik	16073	17939	20282	22894
1810059H22Rik	28392	17496	20311	25291.5
1810060K07	7818	14059	8420	12460
1810062G17Rik	39481	34154	37138	848
1810062O18Rik	13932	8714	17218	5140
1810064F22Rik	4976	3769	6225	5225
1810065E05Rik	7252	11454	20568	16736
18S ribosomal RNA	24492.5	9332.5	16197	20452.5
1LB6scFv	23791	24831	4374	1213
2010001E11Rik	27684	28870	31069	29718
2010001K21Rik	20523	17855	22308	17538
2010003D24Rik	28453	29836	32547	34876
2010003K11Rik	5352	10955	11952	18067
2010003O02Rik	10146.5	9853	8953	21694
2010004M13Rik	1239	40084	3873	37565
2010005H15Rik	34012	38554	10755	13870
2010007H06Rik	34657	29703.5	2606.5	38259
2010013B24Rik	14162	8075	8324	6051
2010016I18Rik	17081	19715	13889	17264
2010106E10Rik	33254	34964	1786	35241
2010107C10Rik	24432	25775	27731	28688
2010107E04Rik	3694	2922	4853	6257
2010107G23Rik	16920.5	17108.5	19104	24236
2010109A12Rik	40087	40179	5359	3655
2010109I03Rik	11932	17244	23322	21092
2010110G14Rik	33918	25708	27445	26479
2010110K18Rik	38389	741	8477	8991
2010111I01Rik	20266	22612.83333	21571.33333	21284.66667
2010204K13Rik	31240	37694.5	38296.5	34578
2010300C02Rik	11961.5	7621	10386	6458
2010308F09Rik	19463	17437	26405	24633
2010309G21Rik	40502	3254	20024	9662
2010315B03Rik	18909	3189	12368	16312
2010320H13Rik	9224	10210	13753	16156
2010320O07Rik	1466	30158	1663	34375

2200002D01Rik	16798	32946	25257	34481
2200002J24Rik	6765	12982	39020	21868
2210008N01Rik	6771	10942	7140	5897
2210010C04Rik	8645	14292.5	15316	15239.5
2210011C24Rik	12234	10881	16346	10056
2210011G09Rik	15226	14027	12692	11301
2210013O21Rik	3733	3816	3332	39545
2210015D19Rik	16773	23740	22034.25	24381.5
2210016F16Rik	16409	21477.5	18287.5	19917.5
2210016H18Rik	1298	25202	3292	32357
2210016L21Rik	16927	15816	12015	12899
2210018M11Rik	22475	13565.33333	16155	17026.66667
2210019G11Rik	22625	35963	2322	5031
2210019I11Rik	1918	9267	16806	16280
2210022J03Rik	34366	40730	957	30954
2210403K04Rik	17364	19149	21403	24267
2210404O09Rik	25544	31928	28744	28799
2210406O10Rik	488	33449	8950	7329
2210407C18Rik	29697	22510	39591	35229
2210408F21Rik	28123	16469	18097.66667	16454.33333
2210408I21Rik	18460.5	16144.5	23530	35070
2210408K08	4614	5135	738	5254
2210408O09Rik	19533	1399	24123	25583
2210409D07Rik	7714	11601	13822	14437
2210409E12Rik	23796	27399.66667	27935.66667	31032.66667
2210411G17Rik	26598	27745	30988	29497
2210412B16Rik	18554	18297	23374	22931
2210416O15Rik	14666	15312	25092	18961
2210418O10Rik	32972.6	28763.6	22763	25153.8
2300002M23Rik	7472	38246	34078	5472
2300009A05Rik	30961	36681	27388	30021
2300010F08Rik	28919	32208	27912	26796
2310001H17Rik	21242.33333	14671	19405.66667	13675.33333
2310001K24Rik	9181	8036.5	14576.5	13007.5
2310002F09Rik	25463	26723	28538	27363
2310002L09Rik	33813.5	35019	2689	25874.5
2310002L13Rik	34810	36179	29925	3373
2310003L06Rik	32906	35609	35899	33859
2310006M14Rik	33909	36477	27747	35708

2310007B03Rik	34479	28689	39052	1869
2310009A05Rik	17087	19224	18867	18134
2310009B15Rik	13451	21557	18386	18746
2310010J17Rik	16883	23245	24411	21861
2310011J03Rik	13720	15441	18689	20146
2310014F07Rik	37462	32728	37877	40126
2310015A10Rik	24701	32194	26924	28567
2310015B20Rik	39404	29441	1201	27168
2310016D23Rik	13400	30731	32021	30706
2310016G11Rik	36566	429	34284	32563
2310021N16Rik	36514	28009	30050	31066
2310022A10Rik	14971.5	13247.5	16512.5	14969
2310022B05Rik	26665.5	23761.5	24849	27287
2310024J23	8650	12730	10899	12675
2310026I22Rik	28281	29655	32463	30892
2310030G06Rik	18489	18171	21200.5	20498.5
2310031A07Rik	5433.5	5626	13827.5	11143.5
2310033P09Rik	20957	20350	21694	20529
2310034C09Rik	40571	40766	2970	5669
2310034G01Rik	38391	193	1514	299
2310035C23Rik	11294.5	8018	12211	12679.5
2310035P21Rik	26416	14285	20394	15410
2310036O22Rik	17759	21044	23971	24017
2310039H08Rik	19852	30142	22796	29290
2310040G07Rik	37255	1678	40365	40323
2310041K03Rik	31201	32869	1361	37139
2310042E22Rik	15395	17454	22069	16095
2310043M15Rik	37470	40663	37936	9406
2310045N14Rik	27105	28755	29037	28013
2310047K21Rik	12713	12820	9142	8388
2310047N11Rik	26115	27454	29289	27994
2310050B05Rik	24694	25913	39619	26470
2310050C09Rik	39735	1815	1696	2751
2310057J18Rik	31278	29724	39330	40925
2310057M21Rik	13709.33333	17224	24153	14114.66667
2310057N15Rik	35498	36655	992	2519
2310058D17Rik	9866	13171	15585	16140
2310058N22Rik	24725	12750	22007	16917
2310061I04Rik	17572	15516	15781	14576

2310061J03Rik	9725	11758	9118	8721
2310061N02Rik	24756	26098	27279	26185
2310067B10Rik	39995	39685	40249	37982
2310067E19Rik	22517	35342	27365	34651
2310068C19Rik	22864	16027	21089	20535
2310068J16Rik	17900	35678	18958	20313
2310069B03Rik	35087	4362	8765	12955
2310079G19Rik	12406	13498	16664	17150
2310081J21Rik	15049	20326.5	17344	16914
2410002F23Rik	11011	10723.33333	11558.33333	11686.33333
2410003L11Rik	9986	8432	6806	12594
2410004B18Rik	21984	28330	26662	29855
2410004P03Rik	28319	35836	529	627
2410006F04Rik	6952	15130	17941	19205
2410007B07Rik	33159	34853	1220	8528
2410015M20Rik	6497	7026	7895	7416
2410016O06Rik	19426.5	18669	18812.5	17043.5
2410017I17Rik	32219.66667	31987	34695.66667	31700.33333
2410018L13Rik	1768	5041	26888	40668
2410022M11Rik	26059	28591	25481	23600
2410024F20Rik	38757	29318	32691	35591
2410024N13Rik	32314	37298	1083	4308
2410039M03Rik	36569	965	29085	27909
2410089E03Rik	4222.666667	27879.33333	28073.33333	16906.33333
2410124H12Rik	18656	16506	23666	18532
2410131K14Rik	23122	27191	21521	22791
2410133F24Rik	26028	21490	10651.5	12717
2410137M14Rik	40554	39264	3076	4494
2410141K09Rik	39377	40599	2238	2640
2510002D24Rik	35565	37581	28784	27594
2510009E07Rik	7155	9127	8040	14832
2510016G02Rik	10502	12148	13007	6465
2510017J16Rik	18385	22380	16204	20195
2510022D24Rik	34677	29689	29115	24306
2510039O18Rik	19402	16738	18592	15690
2510042H12Rik	25232	26491	28329	27248
2510042O18Rik	36311	1071	618	2129
2600006K01Rik	16573	12381	17345	9554
2600014E21Rik	33200	34812	5715	2036

2610001A08Rik	34014	28221	29714	28413
2610001J05Rik	12054	14475	16019	14703
2610002J02Rik	25198	25047	28057	32088
2610002J23Rik	30800	28127	25067	27181
2610002M06Rik	24141	18492	19327.33333	32755.33333
2610005L07Rik	19891.125	24983.875	19211.875	23409.375
2610005M20Rik	17059	16354	19175	17390
2610007B07Rik	7259	10856	15408	13665
2610008E11Rik	34662	26662	30038	27172
2610015P09Rik	13536.5	13782.5	6500.5	21155
2610017I09Rik	15161	18821	22391	20804
2610020H08Rik	24226.66667	13066.33333	25181	12515.33333
2610021A01Rik	23463	29644	28303	31880
2610021J01Rik	31062	32682	34242	32544
2610027F03Rik	6862	11843	17488	20744
2610027K06Rik	29105	31033	30778	29499
2610028E06Rik	28877	589	31927	41127
2610028H24Rik	10569	2980	3688	2583
2610029I01Rik	24818.33333	12508.66667	17007	15397
2610029K11Rik	22832	17383	15573	12179
2610030P05Rik	34316	10967	16085	12646
2610034B04Rik	29940	31460	33741	32081
2610034M16Rik	18151	16074	26179	28906
2610035D17Rik	27782	24656	36309	28650
2610036A22Rik	5862.5	11297.5	7228	11282
2610042L04Rik	21197.5	22296.5	7790	10101
2610044O15Rik	22218.25	21071.75	25551	18691.25
2610100L16Rik	6467	13138	34833	39354
2610203C20Rik	17785.5	16852	20436.5	19274.5
2610203C22Rik	1623	39422	2249	39170
2610204G22Rik	22015	21843	19034	17212
2610206C17Rik	17092	17016	21144	16682
2610209C05Rik	3146	33864	33298	21358
2610300M13Rik	23035	6680	27280	38262
2610301B20Rik	23885	24075	26034	24793
2610301H18Rik	24475	25759	27023	36107
2610303G11Rik	25821	27337	28151	27008
2610305D13Rik	16055	15056	16604	18976
2610306M01Rik	32249	39996	31567	35832

2610307P16Rik	28630	33767	25923	28949
2610317O13Rik	28764	26873	34775	32992
2610318N02Rik	22100	33833	23432	27744
2610507B11Rik	16202	19607.5	21121.75	21905.5
2610509F16	786	36456	31875	35508
2610524H06Rik	9535	13909	12423	13963
2610528J11Rik	16616	19490	20006	20721
2700008E08Rik	25263	20652	25461	30086
2700029M09Rik	22176	18118.33333	11403.66667	16864.33333
2700038G22Rik	3521	35898	30124	28884
2700046A07Rik	27330.5	29113.5	35653.5	35639
2700046G09Rik	6670	15774	11765	12669
2700049A03Rik	14277.66667	14128.33333	13679.66667	3838.333333
2700054A04Rik	27223	28404	30672	29337
2700057C20Rik	32431	34100	36085	33993
2700060E02Rik	8685	9247	7823	9063
2700062C07Rik	5496	6607	4111	6172
2700068H02Rik	33873	25457	1247	37199
2700081O15Rik	6121	8698	10023	10384
2700086A05Rik	32222.5	32479	35138.5	15581
2700086H06	19516	20219	19460	21376
2700094K13Rik	15724	16767	17360	13187
2700097O09Rik	20945.33333	18398.66667	21459.33333	20321.33333
2700099C18Rik	24873	20661.5	24986.5	23513.5
2810001G20Rik	22333.66667	26231.33333	16576	13953
2810002D19Rik	17133	22308	20712	23079
2810004N23Rik	26331	31554	29342	35641
2810006K23Rik	14854	10654	9964	8317
2810007J24Rik	23489	22502	22202	22591
2810008D09Rik	25546	26047	27018	29438
2810011L19Rik	26238	11299	27458	7844.5
2810013P06Rik	27749	35975	26201	36129
2810021J22Rik	15146	15255.5	15761	17359.5
2810025M15Rik	18694	16483	24054	17834
2810032G03Rik	24955	26049	28421	27237
2810038D20Rik	27136	14924	28127	29456
2810038L03Rik	24134	25251	27171	26032
2810039B14Rik	21716.5	24359	26902.5	10060
2810040C05Rik	28116	29592	36944	37118

2810047C21Rik1	8329	9952.5	10915	8748.5
2810049E08Rik	27879	29362	38137	31869
2810055G20Rik	32092.2	32599.8	16147.4	33586.4
2810403A07Rik	14254.2	12478.8	18704.6	19036.6
2810403D21Rik	16730.5	16587.5	16225	11815
2810404F17Rik	24059	25140	27202	26058
2810405F15Rik	31879	33645	34939	33155
2810407A14Rik	1812	6646	17814	14708
2810408A11Rik	25664	10334.5	12109.5	23814
2810408B13Rik	35920.5	33777	4100	40274
2810410L24Rik	20404.33333	33527.33333	36745.33333	35949.33333
2810410P21Rik	25306	26749	27711	26574
2810416G20Rik	38645	26189	1637	24360
2810417H13Rik	31226	29335	27687	35040
2810427C15Rik	13185	7549	7919	10800
2810428I15Rik	21293	29850	24928	30015
2810428J06Rik	13463.5	28152.5	29385.5	28738.5
2810429I04Rik	25166	27645.75	25072.75	22319.25
2810442I21Rik	18046	22928	24004	25371
2810449D17Rik	9343	8081	4681	3610
2810452K05Rik	8398	2490	7672	5390
2810455B08Rik	31019.5	24225.5	28730.5	28588
2810455D13Rik	11371	8059	4478	9817
2810455O05Rik	12181	7164	10707	40684
2810459M11Rik	5969	31428	38444	9354
2810468N07Rik	24770	25226	30945	36256
2810474C18Rik	40845	8114	6704	8581
2810474O19Rik	18274.33333	12255.66667	12133.33333	20106.33333
2.81E+17	22550	26156	25621	30137
2900002J02Rik	28830	30817	30531	29195
2900005J15Rik	753	38426	6689	7138
2900006A17Rik	26790	14303	28652	27677
2900009J20Rik	37256	30482	28145	26517
2900011G08Rik	35188	38066	28187	29142
2900011O08Rik	27071	28476	29284	28033
2900016J10Rik	27901	29665	30202	38490
2900017F05Rik	29502	30798	37719	30929
2900017G11Rik	5489	31588	33102	31929
2900018K06Rik	28255	29649	31844	30332

2900022M07Rik	24611	25960	35575	26374
2900024I21Rik	23516	24571	26593	25467
2900026A02Rik	28669	35689	39551	36598
2900027M19Rik	9415	12085.5	15432	16546
2900041H08Rik	11533	14917	17917	36181
2900041M22Rik	28749	30476	30355	36022
2900042K05Rik	17598	6161	13847	11160
2900045O20Rik	31455	30897	40282	30983
2900052L18Rik	27265	26581	5744	39804
2900052N01Rik	12686.5	13570	19044	18011.5
2900055J20Rik	39904	32969	34802	32952
2900056B14Rik	39215	34135	37516	34895
2900056M20Rik	22424	12532.5	13918	12101
2900060N12Rik	33670	8498	36125	38416
2900060N18Rik	36025	40882	39139	978
2900064B16Rik	5387	4576	5788	7036
2900064F13Rik	25333	26602	28488	27552
2900064K03Rik	33166	26523	28338	27228
2900069M18Rik	12775	28487	6203	38663
2900072G19Rik	25898	27351	28863	27632
2900079G21Rik	33150.33333	34636.66667	36404.33333	27308.33333
2900080J11Rik	9120	15339	13403	14193
2900082C11Rik	38122	6592	27632	26632
2900092C05Rik	3610	30882	34434	1719
2900092D14Rik	14527.5	22122.5	23254	8799
2900093K20Rik	10148.5	28798.5	13599.5	13787.5
3000002C10Rik	33136	30201.5	28347.5	30823
3010003L21Rik	16998	14815	20147	17843
3010026O09Rik	18623	19325.33333	19728.66667	22484.33333
3010027C24Rik	38764	6824	10962	11316
3010033K07Rik	32348	34639	36555	40635
3021401L19Rik	24073	25312	29677	38085
3021401N23Rik	37009	38129	59	40852
3100002H09Rik	36834	23742	25065	23425
3100003L13Rik	40968	1883	9902	11311
3100003M19Rik	15707.5	33482	19703	19156.5
3110001I22Rik	21330	25204	20866	24390
3110002H16Rik	8165	15769	9341.5	16739.5
3110005L24Rik	37676	24730	21296	10551

3110009E18Rik	7132	10756.5	14346	11589.5
3110018I06Rik	2652	1301	5350	4453
3110021A11Rik	4384	9165	11272	14040
3110021N24Rik	9180	7531	12799	12666
3110023J12Rik	29250	30632	32295	30623
3110035C09Rik	34218	7294	26647	9394
3110035E14Rik	38262	39010	2323	41085
3110037B15Rik	19197	34891	24926	23556
3110039M20Rik	4950	8205	13510	10806
3110040N11Rik	37216	2513	606	3176
3110043A19Rik	3402	12962	12190	12840
3110043O21Rik	20485	23679.5	15380.5	24331
3110045C21Rik	28241	37643	29802	35492
3110049I03Rik	29244	31200	30960	29626
3110052M02Rik	20655.25	21233	20108.5	20934.5
3110053B16Rik	256	35816	26346	24051
3110054G05Rik	30162.5	14498.5	35517.5	34654.5
3110056K07Rik	26614	28048	33872	28183
3110056K14Rik	29165	30569	32603	31051
3110057O12Rik	18145.25	26053.5	24964.25	26704.5
3110062M04Rik	4963	6097.5	9191	9071
3110067C02Rik	25123	26439	28048	26987
3110068a07rik	29873	31385	33638	32007
3110070M22Rik	19114	23868	18976	21770
3110073H01Rik	22885	14741	20966	17119
3110076C19Rik	24400	25545	27338	26401
3110079O15Rik	34221	39940	36769	35872
3110080O07Rik	6710	30797	753	35999
3110082I17Rik	18110	18204	20267	20583
3110099E03Rik	29535	31094	32617	33316
3200001D21Rik	8943	11540	23844	24865
3222401L13Rik	36754	31324	26892	32094
3230402G14Rik	7554	1583	9385	15361
3300002A11Rik	40685	33676	6553	12189
3300002I08Rik	25773.5	21252	22370	29163
3300002P13Rik	80	2468	9934	9850
3632451O06Rik	25486.5	25186.5	7798.5	27608.5
3632454L22Rik	31692	34235	34273	32478
3830403N01Rik	32230	33783	35659	33764

3830403N18Rik	24984	29600	39732	37920
3830406C13Rik	11447	14402	9578.333333	12860
3830408C21Rik	7250	204	4849	8563
3830417A13Rik	11905	11833	20686	24429
3930402G23Rik	20561	21185	29711	31237
4122401K19Rik	24135	14417	22098	19088
4430402I18Rik	5008	7091	38086	37597
4631419I20	32317	34123	40223	34075
4631422I05Rik	32016	33157	37145	40360
4631423B10Rik	26999	19508	23308	23324
4632404H12Rik	9959	10768	26919.5	27703
4632411P08Rik	2156	34537	31827	30315
4632415L05Rik	5341	5534	35067	33197
4632419I22Rik	20765.5	34505.5	18227	35704
4632427E13Rik	14176	11183	7755	9954
4632428N05Rik	22833	16647	25916	20404
4632433K11Rik	9917	5411	8447	7476
4633401B06Rik	7477	5337	6690	6350
4731417B20Rik	2347	34379	4568	13331
4732416N19Rik	15916	19314	18286	8187.666667
4732419C18Rik	19654	21928.5	21251	16070
4732456N10Rik	7859	4509	15965	17469
4732460I02Rik	26753	9233.5	14513	11450.5
4732465E10Rik	37762	35711	38837	34509
4732465J04Rik	28310	34945	39667	39566
4732468M13Rik	25761	26989	29425	37325
4732499D12Rik	23737	24895	26373	25377
4733401D01Rik	25984	27235	29109	27932
4733401I12	22813	29822	2294	2299
4733401N17	30391	31989	33455	32118
4831440D22Rik	31624	26909	27141	27934
4833405L11Rik	13604	15050	20097	21679.5
4833408A19Rik	19911	24003	21304	25582
4833408D11Rik	13420	33802	32324.5	33188.5
4833408G04Rik	39497	40598	6289	12041
4833411C07Rik	11989	14810	15455	14490
4833413E03Rik	490	40587	36936	3986
4833414E09Rik	15143	37976	40199	1525
4833415N18Rik	4716	33888	8659	34472

4833417J20Rik	28365	15142	23257	13501
4833419E13Rik	24326	13636	2102	3377
4833420G17Rik	35144.66667	36470.66667	38098.66667	36533.33333
4833421G17Rik	7838	33769	5343	35075
4833422M21Rik	35761	890	4783	8071
4833423E24Rik	13591	12252	29563	28723
4833427G06Rik	33266	1347	31829	37641
4833428M15Rik	21197	26992	19615	21151
4833431D13Rik	25350	26654	28565	27373
4833432P19Rik	28615	18983	23282	20537
4833439L19Rik	11381	10132	9451	10046
4833444C15Rik	29690	1239	6793	13367
4833444G19Rik	35867	20341	20372	19851
4.83E+17	40123	1431	30659	38960
4921501E09Rik	35938	36606	14819	38516
4921504A21Rik	26611	37864	4258	6525
4921504E06Rik	24231	25376	33122	31353
4921506M07Rik	34820	29465	31223	26506
4921507G05Rik	30767	35825	38096	38722
4921507L20Rik	1970	33737	33563	35359
4921507P07Rik	18008	33242	16645	19356
4921508A21Rik	32986	34015	4887	37441
4921508D12Rik	26575	24794	37061	5022
4921508M14Rik	32262	33458	2088	40977
4921509C19Rik	27347	3109	37583	14942
4921509J17Rik	33545	21515	24877	26624
4921511C20Rik	35446	33780	35749	34015
4921511E18Rik	40484	33334	33687	32169
4921511H03Rik	24329.33333	15215	17442.33333	16850
4921511M17Rik	28118	591	31025	29592
4921513D11Rik	36830	31488	35352	2182
4921513I03Rik	8243.5	7516	19830.5	16947
4921515E04Rik	32878	30393	38256	30841
4921515G04Rik	31583	33221	34771	32983
4921517D21Rik	16455	20772	26791	36775
4921517D22Rik	37361	8519	4705	40131
4921522E08Rik	31889	26773	38941	27026
4921522H14Rik	24306	25595	26822	25723
4921522P10Rik	33358.5	33222.5	34196.5	20358

4921523L03Rik	33028	11521	35554	33715
4921523P09Rik	26607	28066	32850	33228
4921524J17Rik	13027.25	13526.5	15272.5	16948.5
4921524L21Rik	32729	36290	36039	38215
4921525H12	33369	35537	40406	100
4921525O09Rik	32014	3069	19888	13376
4921527H02Rik	32271	2949	35484	33641
4921528I07Rik	22211	23542	26663.5	23559
4921529L05Rik	8043	9800	15569	22004
4921530L18Rik	943	3900	3815	3059
4921530L21Rik	27771	29176	18619	666
4921531C22Rik	20816	19040	18295	15942
4921533I20Rik	10675	4765	29535	174
4921534A09Rik	27909	31335	29766	33043
4921534H16Rik	22942	25668	36449	35258
4921536K21Rik	23070	33808.33333	17337	26568.33333
4921537I17Rik	25221	16415	19406	24232
4921539E11Rik	25235	40095	30488	36610
4922502B01Rik	16981.5	39941.5	21945	2928
4922502D21Rik	31667	33328	34741	32955
4930401B11Rik	14273	18292	28086	27902
4930401C15Rik	30325	31901	33409	31959
4930402D18Rik	36009	32245	33011	6992
4930402F06Rik	35995	35029	9703	7237
4930402F11Rik	237	7020	35413	35718
4930402H24Rik	8775	17838	21208	18627
4930402I19Rik	38624	40577	38411	3906
4930402K13Rik	32585	33991	35818	33907
4930403L11Rik	37234	35160	38771	39366
4930403O15Rik	25022	26450	1219	36132
4930404H11Rik	31530	30965	39914	4702
4930404I05Rik	867	28730	37554	38382
4930404N11Rik	28820.5	18731.5	17719.5	15682
4930404O17Rik	9650	13715	15465	19869
4930405H06Rik	37204	41057	2269	41080
4930406M16Rik	29574	30778	35919	31253
4930407I10Rik	18877	22124	26097	26376
4930408K08Rik	32753	34972	39036	33896
4930408O17Rik	40959	4401	9460	9355

4930412C18Rik	29511	40376	29536	4858
4930412F09Rik	25096	26279	37990	26990
4930412F12Rik	18880	15822	15102	20065
4930412M03Rik	14277	22736	16308	22222
4930413E15Rik	4837	40	15398	20329
4930414L22Rik	28588	39591	32238	39296
4930414N06Rik	2871	37032	38664	4156
4930415C11Rik	25009	26248	28060	27045
4930415F15Rik	12740	9064	9871	4662
4930415O20Rik	38786	6766	12836	10108
4930417O22Rik	33300	35377	68	39600
4930419G24Rik	32330	22720	35021	33193
4930420G21Rik	32404	31740	33084	31992
4930422N03Rik	24072	25303	26656	25661
4930425F17Rik	13637	6308	13110	20495
4930426D05Rik	28193	35719	28709	27550
4930426L09Rik	40955	40983	882	1696
4930427A07Rik	18645	20721	25569	18908
4930428F12Rik	6530	2317	10195	12705
4930428G15Rik	32408	34420	34875	33133
4930429B21Rik	6212	18689	15364.33333	15950
4930429F11Rik	33472	28334	4395	36836
4930430A15Rik	12398	3494	12885	13900
4930430F08Rik	16103.33333	22642.66667	20102.66667	21426.66667
4930430J02Rik	27064	28831	28780	27634
4930430J20Rik	16003	18374	28943	35545
4930430M16Rik	29628	31204	32591	31327
4930430O22Rik	2841	6857	12996	12595
4930431F10Rik	35131	2085	33009	5562
4930431F12Rik	22417.5	37698.5	22765.5	16544
4930431P19Rik	24250.5	23282.5	22883	23416
4930432E11Rik	28645	27358	31291	23169
4930432F03Rik	35416	31269	32372	30945
4930432F04Rik	36136	18468	19857	16751
4930432J09Rik	3273	3695	5629	4634
4930432K21Rik	24022.5	20491.5	9535	4763.5
4930433E05Rik	25510	26782	28770	34757
4930433I11Rik	7235	14791	8356	13830
4930433N12Rik	7845	9785	15764	10821

4930434F21Rik	23473	33242	37367	35278
4930434J06Rik	29586	31029	32631	30914
4930435E12Rik	30440	32128	20457	21250.5
4930435F18Rik	34788	35934	6013	7282
4930435H24Rik	26933	28253	30456	29013
4930437M23Rik	40146	5807	1565	36702
4930438A08Rik	14035	18978	10163	17026
4930439G18Rik	26855	34867	29691	31011
4930440C22Rik	31569	33966	36253	1042
4930441O14Rik	27266.5	31503	16445	34037
4930442E04Rik	39585	6330	13814	13142
4930442H23Rik	40392	30520	38069	34547
4930442J19Rik	6104	12143	17670	13526
4930443G12Rik	37592	5031	1004	38755
4930444F02Rik	27867	40994	8579	13280
4930444G20Rik	25885	30461	33081	39410
4930444K16Rik	22981	16260	29271	21567
4930444P10Rik	25625	26740	29296	28039
4930445E18Rik	26992	28038	4889	1636
4930445N08Rik	33627	27744	12504	15459
4930447A16Rik	30358	31863	1780	40194
4930447F04Rik	29816	30573	30832	29888
4930447J18Rik	29061	182	39774	30707
4930447K03Rik	39730	38362	614	37538
4930447N08Rik	28893	30368	31734	30703
4930448D08Rik	25708	27155	28520	31031
4930448F12Rik	13893	19308	25334	31912
4930448K12Rik	24819	26209	30467	40915
4930448K20Rik	112	10149	5537	12626
4930448N21Rik	83	35634	12095	10653
4930449A18Rik	30290	32158	32755	31291
4930449C09Rik	1367	2838	3925	5267
4930449E01Rik	15735	14919	19795	20178
4930449I04Rik	39115	479	29454	28141
4930449I24Rik	29435	28817	34804	34460
4930451E10Rik	21626	30276	31571	28731
4930451G09Rik	11898	26417	18890	21514.5
4930451I11Rik	10208	14075	12644	12221
4930452A19Rik	35191	38644	5134	7548

4930452B06Rik	31703.33333	33292	19167.33333	37542
4930452L02Rik	20272	22561	29619	32514
4930452L12Rik	28328	29759	32692	31060
4930453L07Rik	29609	31662	31394	29952
4930453N24Rik	14180.5	16003.5	30977	14520.5
4930455C13Rik	6050	10634	16605	15163
4930455F16Rik	1158	30665	31160	33883
4930455J16Rik	26629	9512.5	19443.5	15853
4930456K20Rik	29967	31479	33670	32039
4930456L15Rik	12273.5	14123.5	17342	19581
4930457A20Rik	33216	34882	36617	734
4930458B22Rik	5456	6983	12680	11952
4930458D05Rik	26810	28258	32284	37445
4930461C15Rik	27796	29598	29433	28329
4930461L14Rik	25063	35297	28326	3614
4930463O16Rik	18472	18381	15728	13997
4930465A12Rik	19107	21470	21988	20222
4930465K10Rik	32762	37098	40481	38267
4930465K12Rik	27953	29514	30893	29260
4930465M20Rik	5915	40129	15189	14496
4930467D21Rik	29381	30881	32074	30567
4930469B13Rik	17625	25253	26012	27532
4930469G21Rik	27854	13013.5	27668	32954.5
4930469K13Rik	26771	28200	1862	34965
4930470F04Rik	38381	32365	1274	35201
4930470G03Rik	13993.66667	7558.666667	17710	18741.66667
4930470H14Rik	27866	29201	32223	30634
4930470P17Rik	39680	38692	40811	37676
4930471D02Rik	33332	3341	17146	13821
4930471G03Rik	25829.5	37275.5	33262.5	20448.5
4930471M09Rik	28606	22456.5	24778	29096.5
4930473A02Rik	35946	5862	2048	1998
4930473B18Rik	8595	8450	11608	16627
4930473M17Rik	27688	29117	30541	29540
4930473O22Rik	13340	11184	17441	20010
4930474A20Rik	4290	1641	30859	2966
4930474G06Rik	30003	31453	32861	31407
4930474H20Rik	23507	30878	26751	25655
4930474M22Rik	23898	25072	35897	37010

4930474N05Rik	10034	8331	11015	7795
4930477O03Rik	30368	32080	33495	34995
4930478K11Rik	40667	33304	35655	33501
4930478L05Rik	34028	13641	12938	16425
4930478P22Rik	15543	17594	17764	21740
4930479M11Rik	7184	9121	3051	8555
4930480E11Rik	32668	34760	35498	38751
4930480K23Rik	38846	32109	29216	1756
4930481A15Rik	37679	38466	26674	36728
4930481B07Rik	19049	19460	26705	27797
4930482G09Rik	31084	26986	28779	30325
4930483J18Rik	18462	19835	20673	24323
4930483K19Rik	31228	36957	8054	39430
4930485B16Rik	27490.5	28826	37366	29387.5
4930485E13Rik	13571	9668	20436	21237
4930485G23Rik	8512	5091	11719	7497
4930486L24Rik	23714.5	24907.5	16935	18046
4930488B01Rik	15441	18722	24969	27102
4930488L21Rik	20087	20667	23645	22230
4930488N15Rik	7033	4456	18157	12142
4930488N24Rik	8361	14672	3618	15234
4930500F04Rik	28820	30396	38009	1404
4930500G05Rik	33791	32384	33989	32442
4930500J02Rik	37966	34585	5590	38594
4930502C15Rik	19327	24166	32550	1325
4930502C17Rik	32987	29811	31222	30081
4930502E18Rik	19235	23898	32112	36584
4930503E14Rik	21770.5	28139.5	13666.5	26981.5
4930503E24Rik	32502	34045	37143	1087
4930503H13Rik	12560	15848	19779	19422
4930503L19Rik	22999.66667	20737	22068	21780.66667
4930504O13Rik	25134	26215	39248	34618
4930505A04Rik	15031	18355	19653	17534
4930505H01Rik	30578	32195	33998	36056
4930505N22Rik	8770	6779.5	26169.5	10756.5
4930505O20Rik	38558	36440	823	10751
4930506C21Rik	20049	20261	23438	21857
4930507A01Rik	31443	1453	34933	18272
4930507D05Rik	38450	35993	10587	9157

4930507D10Rik	102	9811	13109	8360
4930509H03Rik	38008	20154	21255	14982
4930509J09Rik	33721	32739	36201	3690
4930510E17Rik	26052	27567	28901	27701
4930511A02Rik	27732	29027	30462	37844
4930511E03Rik	32720	34325	35569	33716
4930511J24Rik	24040	25184	26910	25969
4930511M06Rik	32155.5	33719	35824.5	33920.5
4930511M11Rik	39990	5915	40719	9435
4930512B01Rik	26923.66667	34373	33769.66667	33100.33333
4930512H18Rik	32510	34278	36758	33607
4930512M02Rik	27832	29274	30694	29693
4930513N10Rik	36212	37554	33355	36401
4930513O06Rik	16088.5	16139.5	27091	24685.5
4930515G01Rik	27383	36616	25556	37006
4930515G13Rik	20522	21543	22472	23277
4930515G16Rik	7454	12342	12610	15268
4930516K23Rik	36383	34156	35623	4281
4930517O19Rik	9250	19578	16894	16014
4930518C09Rik	28868	30348	36335	30647
4930518F22Rik	14875	22116	24468	34095
4930518J20Rik	1879	36894	27700	31428
4930518J21Rik	30644	35848	2019	6776
4930519D14Rik	28038	29620	30785	29285
4930519F09Rik	9750	11924	11445	13896
4930519F16Rik	18061	27824	17237.5	19433.5
4930519G04Rik	35558.66667	25650.66667	16174.33333	16837.66667
4930519H02Rik	19692	37231.5	7114	7370.5
4930519L02Rik	1909	8663	10556	5127
4930519P11Rik	7870	10060	10159	10663
4930520K10Rik	23719	24353.5	9472.5	26706.5
4930520O04Rik	4271	27723	16141.5	20131
4930522H14Rik	17867	19453	20008	21758
4930522N08Rik	29642	29131	31720	30221
4930522O17Rik	27320	28645	31855	30239
4930523C07Rik	21315.2	25996.4	25271.2	20796.8
4930523O13Rik	26073	27599	28837	27688
4930524B15Rik	606	906	7143	5964
4930524J08Rik	13044	14232	15041	16449

4930524L23Rik	38482	40322	35626	34880
4930524N10Rik	3444	40271	1907	39766
4930524O08Rik	17087	17368.5	22478.5	38177
4930525G20Rik	34445	33141	860	34404
4930526F13Rik	32274	29360	5952	1352
4930526I15Rik	20026	21464	22063	21277
4930527F14Rik	30011	32085	31867	30415
4930527J03Rik	25896	30012	36638	36890
4930528A17Rik	25549	26834	28621	28787
4930528J11Rik	24345	25505	27357	26316
4930528J18Rik	9834	9235	16877	12980
4930529F22Rik	27110	33255	30362	35199
4930529F24Rik	23783	24965	6758	28585
4930529I22Rik	26470	34487	2069	32721
4930529K09Rik	11532	11491	18523	16029
4930532J02Rik	785	6418	9564	9617
4930532M18Rik	28345	30152	30222	28912
4930533K18Rik	23565	11557	25620.5	10974
4930533L02Rik	1168	780	368	1867
4930533N22Rik	19659	22321	22466	23035
4930533O14Rik	23729	20274.66667	23790	24856.66667
4930535C22Rik	31537	33203	34634	32862
4930535E02Rik	37691	16970	23598	41128
4930535F04Rik	13851	13692	19940	19480
4930535I16Rik	17010	16816	19757.5	18491.5
4930538E20Rik	27666	28958	30933	31552
4930538K18Rik	15429.66667	20407	22944.33333	18340.33333
4930539C22Rik	23786	24847	40710	31702
4930539E08Rik	16933	21728	25505.5	21706
4930539H15Rik	26545	39050	23008	37305
4930539J05Rik	35047.4	33793.4	20859.2	21304.2
4930539N22Rik	37038	5990	18898	8691
4930540E01Rik	9167	11150	38522	3132
4930540M05Rik	23934	24973	26914	37073
4930542C12Rik	28558	27052	28442	1008
4930542C16Rik	29710	30939	32975	31422
4930542D17Rik	3892	38474	14854	12335
4930543E12Rik	2136	5120	6953	5603
4930544G11Rik	20232	24322	16309	21387

4930544L04Rik	35240	30257	4388	4710
4930544M13Rik	1096	37463	30965	1179
4930546C10Rik	16696	27516	12648	11368
4930547C10Rik	36758	29918	6955	11123
4930548F15Rik	4157	11244	18217	15920
4930548G14Rik	29879	14099	31769.5	36352
4930548H24Rik	40852	5294	36845	38626
4930548J01Rik	20394	22987	23716	26592
4930549C01Rik	29522	4388	29295	28033
4930550C14Rik	1517	163	40877	4276
4930550G17Rik	21318	21721	21995	20096
4930550L24Rik	19557.5	23078	23226	26103.5
4930551E15Rik	24703	25826	28122	26894
4930552N02Rik	1532	40439	38994	3249
4930552P06Rik	24533	25617	27860	26684
4930553I04Rik	14224	12397	11805	13186
4930553J12Rik	14066.5	16615.5	23544	20883.5
4930553M12Rik	23487	24539	26603	25474
4930554N03Rik	27821	29172	31515	30008
4930554P06Rik	28783	38735	31585	30519
4930555F03Rik	31174.66667	33504.33333	35591.33333	24192.33333
4930555G01Rik	27858	12319	37401	26918.25
4930555K05Rik	31339	32986	34475	32703
4930555K19Rik	37128	37966	39898	39287
4930556A17Rik	28716	30702	30370	29039
4930556A20Rik	28884	26726	35730	33838
4930556H04Rik	2676	36032	5553	4202
4930556J24Rik	25149	39993	5085	1079
4930556L07Rik	4162	10047	8273	12286
4930556M19Rik	20415	21595.66667	7662.666667	20878.33333
4930557A04Rik	3833	16112	8977	13318
4930557B06Rik	9456	9461	13873	20649
4930557B21Rik	28428	30208	30403	29074
4930557K07Rik	27540	30545	30180	37072
4930558C23Rik	36625	39423	9485	4684
4930558J22Rik	41076	6084	3763	6922
4930558K02Rik	30936	33161	33757	38795
4930562A09Rik	1349	7195	578	8858
4930562C15Rik	40832	28521	28115	39759

4930562F07Rik	3120	32583	3229	41017
4930562F17Rik	30791	31972	33575	39926
4930563D23Rik	32011.5	31829	34628.5	19251.5
4930563E18Rik	27197	28819	29324	28233
4930563E22Rik	8379	9856	10782	10879
4930564B18Rik	7960.5	13471.5	12770.5	17905
4930564C03Rik	24302	1479	31887	26831
4930564D02Rik	28129	29483	32215	30665
4930564G21Rik	31046	32723	34357	32760
4930564K09Rik	29555.33333	21522.66667	25661.66667	27524.66667
4930565D16Rik	27451	28977	29893	28613
4930565N06Rik	27430	29096	37956	28094
4930566N20Rik	27788	29125	31757	30254
4930567H12Rik	32207.5	33735.5	4180	22716
4930567K12Rik	23407	24533	26223	26339
4930568D16Rik	5713	9814	11066	11562
4930568E12Rik	30241	31758	33275	32745
4930569F06Rik	9540	12308	17614	17281
4930570B17Rik	24659	39151	28592	32218
4930570G19Rik	25828	28305	29610.5	34971
4930572J10Rik	28296	29790	32454	37708
4930572P05Rik	29415	7192	2216	39324
4930573C08Rik	6428	13044	10015	9367
4930573O21Rik	32603	9211	39474	40576
4930577N17Rik	26862	31603	25144	21561
4930578C19Rik	32112	30094	10963	3843
4930578I06Rik	40036	28842	35242	39934
4930578I07Rik	3854	7713	14996	16868
4930579C12Rik	7085	17528	20076	27582
4930579D07Rik	26585	29000	28795	36245
4930579F01Rik	33341	29750	9461	30468
4930579G18Rik	34000	31197	26842	5451
4930579G24Rik	31909	35037	38148	37215
4930579H20Rik	23281	24330	12743	28336
4930579K19Rik	16805	23700	24370	27309
4930581F22Rik	31017	28195	29790	32746
4930583I09Rik	4993	1007	8163	3455
4930583K01Rik	10058	8863	22207	22490
4930584F24Rik	1778	100	35978	1737

4930588G05Rik	1641	17132	23131	23610
4930588J15Rik	32966	5648	37035	4040
4930589P08Rik	35466	38715	33476	31873
4930590J08Rik	22167	27167	24870	26922
4930591A17Rik	14503	17976	21565	23154
4930592A05Rik	32947	34976	35396	33563
4930592I03Rik	35783	29863	8155	38039
4930593A02Rik	3703	2056	5724	11153
4930594C11Rik	26841	27259	29795	28416
4930595D18Rik	11960	19264	16401	11615
4930595L18Rik	31410	27037	37079	2144
4930595M18Rik	6811	7918	27091	25937
4930596D02Rik	39338	1303	12345	13769
4930597G03Rik	34135	31026	937	31026
4930597L12Rik	7294	2810	10003	11381
4930598N05Rik	32343	39848	37504	28146
4930599N23Rik	14066	12255.5	12231.5	9592
4931400O07Rik	18352	19341	25328	26446
4931402G19Rik	29623	31314	971	30129
4931402H11Rik	33193	34729	37503	39217
4931403M11Rik	3671	11053	16722	9135
4931406B18Rik	22333.33333	23287.66667	17939.33333	29338.66667
4931406C07Rik	12689.5	8983	18100.5	10948
4931406H21Rik	18553	15410	10878	10004
4931406P16Rik	7942	40749	6507	2997
4931407E12Rik	12118	35624	4401	1716
4931408D14Rik	16842	19941	21053	20005
4931409K22Rik	39325	32560	32865	31370
4931414P19Rik	10368	9059	2580	1913
4931415C17Rik	24088	22945	24590	26271
4931417E11Rik	23773	36720	27118	1559
4931419H13Rik	2697	8928	15656	16364
4931422A03Rik	8845	6196	37495	546
4931423N10Rik	7795	1159	39585	10075
4931428F04Rik	32682.33333	23473	23873.33333	18115
4931428L18Rik	35545	20392	22572.5	21956
4931429I11Rik	26170	18856	30611.5	8978
4931429L15Rik	30297	30685	1070	1058
4931429P17Rik	23262	24312	26453	27354

4931430N09Rik	30516	32072	34034	32364
4931431C16Rik	13580	8816	6819	6092
4931431F19Rik	19384	21818	23754	26139
4931432P07Rik	28729	29894	31806	30433
4931440F15Rik	8663	7860	3524	482
4931440J10Rik	38963	1557	39974	40567
4931440L10Rik	20709	24018	24948	31331
4932407I05	14049	12735	24725	21057
4932411E22Rik	36366	2121	33215	31672
4932411K12Rik	27727	35590	30823	29507
4932411N23Rik	9039	8893	15852	14356
4932412D23Rik	32546	34331	36650	34415
4932414J04Rik	17116.5	18663.5	7508	9249.5
4932414N04Rik	29076	30570	32546	37203
4932415G12Rik	29349.33333	24487.66667	23627.66667	26452
4932415M13Rik	27514	26185	11811.5	12826.5
4932416H05Rik	7568	9456	9892	10385
4932416J16Rik	28955	30422	31817	30211
4932416K20Rik	17869	20533	21341	28246
4932418E24Rik	5977	8414	14194	16933
4932422M17Rik	1699	39202	32257	35583
4932429P19Rik	29564	33718	11618	36216
4932431P20Rik	31063.5	31049.5	17315	33838
4932433N03Rik	6466	9742	7761	7973
4932434E15Rik	35474	38325	2337	4038
4932435O22Rik	9759	17211	16914	19498
4932436B18	26206	27310	30078	28755
4932438A13Rik	22825	20468.83333	24922	23358.5
4932438H23Rik	28775	21224	29662	23288
4932439E07Rik	6787	8411	7840	9647
4932441J04Rik	24437	28185.5	32336	14080.5
4932442A14Rik	31329	971	36360	34432
4932442E05Rik	29448	31433	31369	32716
4932442G11Rik	35589	36923	33916	6708
4932442L08Rik	32747	34574	36066	35886
4932443L11Rik	36485	39414	4404	5682
4932702P03Rik	36792	409	31513	29999
4933400A11Rik	32999	34862	36358	34258
4933400C23	33990	37315	30333	40055

4933400F03Rik	30667	32305	33817	32437
4933401B06Rik	14234	16109	12717	12749
4933402D24Rik	25753	27058	28574	27704
4933402E13Rik	25078	26171	28606	27421
4933402J07Rik	25722	26883	39481	38042
4933402J10Rik	25155	35862	27625	26505
4933402N03Rik	7078	6426.5	9912.5	15711.5
4933402P03Rik	26352	34948	13714	12558
4933403J19Rik	6342	9689	38159	25280
4933403O03Rik	7603	10539	13689	13625
4933403O08Rik	32190	33929	5921	5047
4933404G15Rik	26098	27445	28961	27777
4933404O12Rik	20688.5	19934.5	21654	15654.5
4933405D12Rik	34164	9584	5514	3279
4933405L10Rik	30210	30334	39684	39234
4933405O20Rik	30527	26887	35173	3
4933406B15Rik	29118	30597	31931	30922
4933406B17Rik	18434	25203	25308	31406
4933406C10Rik	19779	11492	13433	14006
4933406F09Rik	32724	34129	35803	33906
4933406I18Rik	27329	28531	34330	29292
4933406J08Rik	26534	27612	30521	29154
4933406K04Rik	5165	33703	2892	5883
4933406M09Rik	24537	25835	27236	28380
4933406P04Rik	23288	23603	21670	21455
4933407A17Rik	32565	34434	35781	34300
4933407G14Rik	30485	32081	33622	32119
4933407I05Rik	6008	7589	16714	11576
4933407K13Rik	24692	15031	23196	18327
4933407L21Rik	27700.5	31342.5	38307	32094
4933408A14Rik	25863	27201	28804	27656
4933408J17Rik	34657	37420	31954	35161
4933408M05Rik	32520	33663	37489	35511
4933409F18Rik	5754	7189	17319	15749
4933409G03Rik	27409	28877	30199	30359
4933409K07Rik	11333.66667	5292.666667	20162.66667	8799.333333
4933411B09Rik	11620	9238	41128	3545
4933411E06Rik	33245	34846	37153	35293
4933411G06Rik	17120	18103	24611	15640

4933411K16Rik	1525	4832	7684	7381
4933411O13Rik	27214	28659	30095	28779
4933412A08Rik	32488	33926	38203	34067
4933412E24Rik	5788	161	1113	1069
4933413G19Rik	39038	1783	5286	3342
4933413I22Rik	31397	33056	34521	32734
4933413J09Rik	28975.5	32134	14540	16414
4933413L06Rik	27464	28849	30797	29310
4933414I06Rik	17821	14243	24265	22291
4933414I15Rik	13103	16258.5	14018	19712.5
4933415A04Rik	18614	21805	23217	24858
4933415F23Rik	30582.5	29238.5	28963.5	33522
4933416E14Rik	40455	33618	6374	38337
4933416I08Rik	20012	36067	28665	33386
4933416K23	7661	6703	9622	14525
4933417A18Rik	11223	14593	22860	18077
4933417G07Rik	9944	16253	12086	17559
4933421D24Rik	20496	13482	17997	20411
4933421I07Rik	3128	7852	8074	7748
4933421O10Rik	35486	7720	1833	6211
4933422H20Rik	28827	34797	28715	39480
4933423P22Rik	8145	10552	4795	7796
4933424G05Rik	24499	25590	27784	26604
4933424G06Rik	16585	36573	21998	20398
4933425B07Rik	38966	34880	36426	34299
4933425H06Rik	6423	11194	13812	16792
4933425L06Rik	23103	25939	26127	31691
4933426D04Rik	16031	18499	24824	24947
4933427C19Rik	1340	1378	40005	37739
4933427D06Rik	26449	27553	30364	29009
4933427D14Rik	22532.5	26881	25009	27125.5
4933427E11Rik	24132	25237	27213	26067
4933427E13Rik	29776	31350	32153	30795
4933427G17Rik	17534.5	15793	28678	28883
4933427G23Rik	34886	35206	30994	36192
4933427I18Rik	32199	35256	38596	6111
4933428M09Rik	1506	1969	1571	5810
4933428P19Rik	24265	25389	27295	26166
4933429H19Rik	25994	19523	12115	9516

4933430I17Rik	31902	12745	24421	1879
4933431E20Rik	40112	35983	34333	40535
4933431I19Rik	16736	23665	26351	33484
4933432G23Rik	39894	6935	30636	29373
4933432I03Rik	31825	40351	35250	7225
4933432I09Rik	9757	13512	15901	15630
4933432K03Rik	24510	25785	38498	27946
4933433C11Rik	36944	377	39925	12417
4933433G08Rik	40023	31057	30569	35794
4933434C23Rik	23029	19173	28423	29446
4933434E20Rik	23278	26779.66667	24059.66667	22022.66667
4933434M16Rik	27032	4208	31653	32803
4933434P08Rik	24287	25384	27446	26288
4933435E02Rik	14813	11514	23445	24777
4933435F18Rik	33313	33652	27206	26268
4933435N07Rik	29214	31185	30906	29543
4933436C20Rik	35591	7644	2345	6918
4933436E20Rik	31848	6869	41107	10875
4933436I01Rik	28282	29071.5	18949	29087
4933436I20Rik	32707	1032	6243	10611
4933437N03Rik	12049	22077	26932	31235
4933438A12Rik	40096	25208	2207	39761
4933438B17Rik	11545	5249	9038	3900
4933438K21Rik	31195	24665	38501	33529
4933439C10Rik	19061	17934	18191	16810
4933440M02Rik	34439.5	16758.5	2454.5	2072
4933440N22Rik	21313	20582	21762	21400.5
4.93E+23	31251	3262	6516	10755
5031410I06Rik	31073.5	3622	18590.5	21322.5
5031414D18Rik	40173	40670	51	1570
5031420N21Rik	31282	917	34197	32583
5031425E22Rik	31678	29739	12128.5	28571
5031425F14Rik	27960	18946	16882	11082
5031426D15Rik	27675	29020	31478	29975
5031434C07Rik	37566.5	19632.5	20305.5	20632.5
5031439G07Rik	14512.5	27692.5	31990.5	26223
5033406O09Rik	8073	10827	9945	14134
5033418A18Rik	2728	25524	23991	35687
5033421B08Rik	22739	15012	29152	24122

5033423K11Rik	29674	31075	32572	31040
5033428I22Rik	2758	374	2381	12446
5033430I15Rik	9518	11120	9447	11484
5133400J02Rik	2636	29066	29822	40980
5230400M03Rik	13913	12271	14565	16306
5330411O13Rik	22726	12183	26290	25214
5330416C01Rik	26578	39459	30412	29056
5330417C22Rik	12655.5	27821	35684	39757
5330417H12Rik	23842	25020	26471	25495
5330421C15Rik	24183	14713	25106	20193
5330421F21Rik	38068	32132	39971	31306
5330426L24Rik	39793	35585	31017	34908
5330426P16Rik	25168.75	16764	26613.25	19693.75
5330427O13Rik	36466	21454	26016	27284
5330430B06Rik	30723	32902	32858	31276
5330430C04Rik	30599	35939	37932	37194
5330430P22Rik	23033.5	21123.5	23712.5	5563
5330431K02Rik	8788	11124	12952	12721
5330437I02Rik	21680	18753	25558	27905
5330437M03Rik	28181	29622	32619	30935
5330438D12Rik	22419	20939	22442	22493
5330438I03Rik	24817	28177	34071	34570
5330439B14Rik	13039	12008	11339	11129
5330439M10Rik	8132	8475	13523	12863
5430401F13Rik	11590.5	18518.5	34922.5	17722
5430401H09Rik	31775	33603	35188	33675
5430402E10Rik	18270	23479	23002	24352
5430402P08Rik	33688	27848	29116	27784
5430403G16Rik	20549	14364	15323	13510
5430405H02Rik	23833	22258	21942	22190
5430410E06Rik	30073	36506	31808	34644
5430410O10	2753	896	1459	168
5430411C19Rik	14225	11005	11263	5914
5430414B12Rik	27069	33142	29653	2331
5430414B19Rik	23199	24258	26242	25149
5430416B10Rik	22818	3615	18749	10425
5430416O09Rik	19207.5	13934	17996	35164
5430417C01Rik	21160	19262	21067	19202
5430417L22Rik	15941	16653	16862	18255

5430419D17Rik	26437	27619	31104	29522
5430425J12Rik	8520	15710	39392	24798
5430427F17	22344	23812	17994	26618
5430427M07Rik	23227	24321	26120	25081
5430427O19Rik	9724	661	3560	30931
5430430B14Rik	34350	38385	34519	35615
5430434G16Rik	21174	20045	22827	24824
5430439C14Rik	960	37142	39491	29012
5430440L12Rik	2170	793	4614	158
5430440P10Rik	25248	21232	24189	20913
5530400C23Rik	19380	18049	21694	22896
5530401A14Rik	30777	18060	35489	19241.5
5530401N12Rik	8864	8635	12391	10754
5530402H23Rik	39461	18516	25056	27506
5530601H04Rik	11895	11201	10437	12751.5
5730403I07Rik	39040	36019	37114	34114
5730405A10Rik	36328	39880	38197	37220
5730407I07Rik	28864	36766	31400	29970
5730409E04Rik	21839	20539	21839	21479
5730409L17Rik	18647	22424	22654	27739
5730410E19Rik	11135	6750	12402	17016
5730416F02Rik	22905	19700	22850	23543
5730416O20Rik	36417	27405	7535	6333
5730419A17Rik	25661	27189	27905	26919
5730419F03Rik	38203	31997	31880	30441
5730433N10Rik	27558	31659	31195	29689
5730437C12Rik	30275	38652	32290	31509
5730446D14Rik	36876	27269	30911	31630
5730453C05Rik	2000	31725	34092	31722
5730455P16Rik	22151.33333	24951	20301	23467.33333
5730458M16Rik	4247	29846	39473	32318
5730460C07Rik	29228	30615	32438	37375
5730480H06Rik	14903	11159	16385	15358
5730488B01Rik	36776	28907	38010	41140
5730493B19Rik	29974	7215	33422	31808
5730507C01Rik	30996	37798	33343	31679
5730508B09Rik	22390.5	4980.5	3943.5	5402
5730510P18Rik	36091	39462	31231	37971
5730521K06Rik	11391	20571	18709	21430

5730522E02Rik	22351.5	23298	26258	25684.5
5730553K21	10144	557	9643	9471
5730559C18Rik	27224.33333	16586	15880	9716.333333
5730564C23Rik	20940	1086	6573	7896
5730575G16Rik	13199	12997	5539	8035
5730575I04Rik	29225	20271	25826	23537
5730577I03Rik	31730.5	27627.5	28239.5	30475.5
5730585A16Rik	32888	34507	4318	36345
5730596B20Rik	25305	29087	35275	36780
5830400J07Rik	8100	8020	2946	7702
5830403L16Rik	31071	33104	34510	32560
5830403M04Rik	34138.5	34560.5	16522.5	37534.5
5830405M20Rik	3378	8601	7884	12238
5830408C22Rik	18844	18224	16044	18424
5830409B07Rik	13335	17580	17314	20095
5830410O09Rik	24713	25892	27851	26685
5830411J07Rik	2878	38897	3277	3950
5830411K02Rik	24795	26160	27240	26147
5830411N06Rik	32879	34340	36035	36874
5830415B17Rik	21566	10837	10435	12152
5830415F09Rik	3830	21258	14089.5	11435.5
5830416P10Rik	15823.5	26218.5	23587	12585
5830417A05Rik	26945	28378	29684	28692
5830417I10Rik	22182	17871.66667	19507.33333	29867.33333
5830420C07Rik	19378	31301	12617	12008
5830427D03Rik	154	34863	29797	29627
5830428M24Rik	14118	5548	4539	6953
5830432E09Rik	7020	18656	14366	22543
5830432F11Rik	1077	1527	3210	9793
5830434F19Rik	8370	11416	2649	564
5830435N06Rik	9050	32879	33453	36906
5830453K13Rik	22465	32315	35714	34248
5830454E08Rik	1934	4020	5126	2823
5830458C19Rik	18849	16009	20051	21166
5830461L22Rik	31956	33224	36607	34575
5830462O15Rik	9798	6429	3833	4164
5830468F06Rik	10468	11425	16528	16148
5830468K08Rik	24286	10626	15331	15848
5830469G19Rik	3435	40407	1518	40054

5830472F04Rik	21923	18705	21085	19999
5830473C10	929	6111	10967	4162
5830474E16Rik	26209	27684	28738	27579
5830477G23Rik	22755	35200	34194	40587
5830490A04Rik	23712	24874	26653	767
5930403N24Rik	33860	31025	35743	39376
5930416I19Rik	30372	28188.5	27201	26505.5
5930422O12Rik	26162	27093	29817	1112
5930427J20Rik	25186	24596	12577	14626
5930436O19Rik	36842	30430	34271	29436
5930438M14	27552	36499	30684	29369
6030419C18Rik	38071	36228	7836	6499
6030422H21Rik	36344	41113	930	6875
6030426L16Rik	31946	33178	36513	37926
6030440G07Rik	17312	18854	25392	30821
6030442H21Rik	37260	1853	39961	37772
6030443J06Rik	17245	15804	21211	27006
6030445D17Rik	33218	32066	33410	31885
6030452D12Rik	12394.5	25606	14532	13140
6030458C11Rik	29051.25	33215.25	32677.5	31629.5
6030468B19Rik	463	5994	10790	14318
6030487A22Rik	37498	23030	35436	39694
6030498E09Rik	28222	2017	40208	7626
6230400D17Rik	29117.5	26094	25434.5	25099.5
6230414M07Rik	31268	38635	4125	5326
6230415J03Rik	29306	30849	31889	37351
6230424C14Rik	19579	1278	5910	28445
6230426I18Rik	15086	9899	10223	14125
6330403K07Rik	7189	13165	32047	9378
6330403L08Rik	16355	16810	16607	16376
6330407A03Rik	22437	10797	16843	16749
6330408A02Rik	12172	9214	11423	11784.33333
6330409D20Rik	38419	3153	12239	16945
6330411D24Rik	12339	13447	9830	17081
6330411E07Rik	14611	10579	10079	11808
6330415B21Rik	27156	9117	187	421
6330415G19Rik	28357	29763	31902	30388
6330416G13Rik	13886.33333	27860.33333	22291.33333	10851
6330436F06Rik	38222	33218	33937	38848

6330437I11Rik	36682	39826	33507	5476
6330444E15Rik	23586	24633	39211	333
6330509M05Rik	38116	35449	28600	27292
6330510M09Rik	25695	26872	5521	27296
6330526H18Rik	2948	34689	37211	35348
6330549D23Rik	29009	29011	32959	34351
6330565B04Rik	28681	30402	30605	29437
6330566A10Rik	31322.5	33039	34520.5	35426
6.33E+19	19436	21797	22418	28249
6430500C12Rik	35536	32640	39809	2944
6430514K02Rik	14584	15413	15999	18349
6430519N07Rik	35606	1575	29061	34560
6430531B16Rik	15371	17191	19755	16407
6430547I21Rik	31562	33246	35043	33336
6430548G04	2032	10414	21876	24202
6430548M08Rik	20757	23869.5	30082	23562
6430550D23Rik	28208	31457	23698	24440
6430562O15Rik	25351	22423	22748.5	23412.5
6430571L13Rik	26574.5	16017.5	16697	19081
6430573F11Rik	37288	4223.5	9331.5	10373.5
6430584L05	24878	26083	27863	26906
6430590A07Rik	3466	4652	912	473
6430590A10Rik	25701	26829	29650	35629
6430604M11Rik	26767	27895	38503	38073
6430628N08Rik	140	37600	32570	31174
6430706D22Rik	29870.33333	21840	22691.33333	18439.33333
6530402F18Rik	9873	11930	11934	12360
6530403H02Rik	15709	26085	21788	24266
6530403M18Rik	33048	34531	37237	35345
6530406A20Rik	24153	19355	25624	22462
6530409C15Rik	37404	25978	29203	40290
6530411M01Rik	11278	7950	18449	14796
6530439I21	15749	16359	35200	38315
6620401J10Rik	25435	26635	28517	27381
6720401G13Rik	11360	9018.5	9219.5	9251
6720406K03	31626	33357	34255	32623
6720422M22Rik	23010	4495	8006	1776
6720462K09Rik	7476	39306	6510	3019
6720468P15Rik	10107	18882	13297	7155

6720470G18Rik	15242	16550	13984	14332
6720473M08Rik	36501	15350	21871	23524
6720473M11Rik	360	30906	32776	519
6720474J12Rik	23641	24770	26377	25301
6720475J19Rik	1529	24156	24492	23246
6720482D04	33186	34733	37263	35385
6720482G16Rik	14113	5468	849	2385
6820402A03Rik	15554	33026	40914	33036
6820402I19Rik	14949	14437	15579	30110
6820408C15Rik	6421	5948	15236	22183
6820431F20Rik	11807	10718.5	10296.5	11543.5
7030407O06Rik	8232	3039	7270	4455
7420700N18Rik	31070	32684	33921	38457
7530428D23Rik	3230	1111	18402	16257
7630403G23Rik	35026	33639	58	34997
8030402F09Rik	38345	39387	10528	7855
8030411F24Rik	217	33898	1897	39006
8030423F21Rik	4285	1161	10032	8341
8030423J24Rik	38272	31426	31853	38688
8030431J09Rik	22197	20103	17343	21250
8030445P17Rik	31932	33166	36227	34225
8030450B20Rik	24472	25695	27306	26357
8030451A03Rik	28827.66667	31868	23990	31904
8030453O22Rik	30259	24455	10990.5	7941
8030462N17Rik	12168	17447.5	15674.5	20806.5
8030474K03Rik	901	37361	39237	3748
8030476L19Rik	27500	28983	29776	1485
8030488J09Rik	9051	10280	13114	13242
8030497O21Rik	26146	27526	28921	27728
8030498B09Rik	2641	2033	38949	6074
8030498J20Rik	12933	31363	16018	17889.5
8430406H22Rik	11065	39063	1538	4931
8430406M14Rik	1721	39437	40002	37225
8430406P12Rik	10024	5920	9872	7287
8430408C20	8213	5389	2695	2145
8430408G22Rik	13480	4103	17480	40536
8430408O14	35457	15000	26275	17429
8430416G17Rik	20097.5	23960.5	21170	21940.5
8430419K02Rik	24046	28791	14907.5	33738.5

8430425A16Rik	25192	27644	27377	26312
8430428J23Rik	6582	2252	7733	6261
8430429K09Rik	6071.5	8904.5	8589	7484
8430431K14Rik	35009	29157	29562	28441
8430432A02Rik	34377	38859	36593	31705
8430434A19Rik	36855	30991	3040	3534
8430436N08Rik	14540	19868	22830	17498
8430437B07Rik	25624	26743	29348	28082
8430437N05Rik	23714	24837	26521	25407
8430437O03Rik	11168	2579	8304	9005
9030201C23Rik	37129	34670	36713	32587
9030203C11Rik	33454	23802	30779	37178
9030405F24Rik	36737	38944	35294	3976
9030407C09Rik	32473	34107	36407	34517
9030407P20Rik	2528	5377	1500	6152
9030411M13Rik	36243	28297	309	28620
9030419F21Rik	10488	8039	2416	40759
9030425P06Rik	5462	3114	2445	5604
9030607J07Rik	10749	37396	32650	31246
9030607L20Rik	22434	19627	21885	23240
9030612E09Rik	23172	24250	26714	25094
9030617O03Rik	17351.5	14760	15264.5	13231.5
9030619P08Rik	33888	39499	30186	38242
9030622M22Rik	28158	29304	40986	36230
9030624G23Rik	31435	33160	34785	33017
9030624J02Rik	7901	17858.25	26764.5	27009
9030625N01Rik	16522.5	21705	18390	17942
9130002K18Rik	4042	30521	40805	39158
9130004C02Rik	18011	16952	16054	15969
9130008F23Rik	24423	29300	38160	27839
9130011E15Rik	25870	28904	23631	25169
9130011L11Rik	11633	11209	14002	12334
9130016M20Rik	34169	24816	26745	25750
9130019P16Rik	18991.33333	18235.66667	21282	22788.66667
9130020O15Rik	18091.66667	16851	32096.33333	21263.33333
9130023H24Rik	22797.5	21163.5	19340	21019
9130024F11Rik	18234	16944.5	27226	22352.5
9130204L05Rik	24066	25483	26852	3776
9130206I24Rik	22245	21147.5	20542	16626

9130214F15Rik	27486	29111	29587	28448
9130221H12Rik	30733	25773	29009	26064
9130221J18Rik	26293	27865	28917	29148
9130227L01Rik	30199	27597	30568	29152
9130230L23Rik	27966	21132	16667	25207
9130230N09Rik	15442	20722	16529	18501
9130401L11Rik	29676	31332	37051	1790
9130401M01Rik	13236.33333	18926.66667	14274.66667	21286.33333
9130404H23Rik	28085	37495	31693	2697
9130409I23Rik	24620	25863	27417	26277
9130414P19Rik	27592	36502	31363	29876
91300000000	14202	16426	12569	15273
9.13E+15	29151	30582	32035	30432
9230104K21Rik	36988	26202	27486	30389
9230104L09Rik	26143	27270	30735	38628
9230106D20Rik	19866	22034	26102	24319
9230109A22Rik	10535	26881	4922	37808
9230110C19Rik	39916	34946	2593	39403
9230110F11Rik	26757	31243	34036	2747
9230110F15Rik	6879	13032	19516	20725
9230110I02Rik	36127	32530	9492	9903
9230110K08Rik	13536	8567	15469	9289
9230112D13Rik	18404	16982	21604	23950
9230112E08Rik	10088	4773	8762	6653
9230112J17Rik	12445	15626	17126	18593
9230114K14Rik	24468.5	20171	23406	23354.5
9230115E21Rik	32628	1510	36399	2361
9230116L04Rik	36049	2091	697	2437
9330101J02Rik	18916	20377	32863.5	18176
9330102E08Rik	40543	3766	30191	4681
9330111N05Rik	31648.5	31566	21053.5	30541.5
9330119M13Rik	34977	17081	22535	20945
9330123L03Rik	24001	25141	26865	25910
9330132A10Rik	24139	25266	27824	26125
9330133O14Rik	10309	12225	36098	1255
9330151L19Rik	24020.5	18504	19184	18385
9330153N18Rik	13470	31809	12868	6229
9330154F10Rik	30349	32227	32640	31166
9330154J02Rik	28358	30160	30858	35083.5

9330154K18Rik	4336	9236	18864	17140
9330155M09Rik	38934	39421	27683	26484
9330158H04Rik	29745	31225	32989	35425
9330159F19Rik	31187	27730	30125	35908
9330159H11Rik	24539	25742	27452	26307
9330159M07Rik	18550.5	22251.5	29573.5	20697.5
9330159N05Rik	32215	41095	36916	11503
9330159N22Rik	25957	27324	28769	27810
9330161L09Rik	10620	13675	12147	14959
9330162012Rik	27091	34349	29761	39611
9330168M11Rik	24773.5	27274.5	25671.5	30047.5
9330168O09Rik	38552	28229	29773	28465
9330169L03Rik	22238.5	28089.5	33694.5	32952
9330174C13Rik	6233	3872	2617	5804
9330175E14Rik	3403.333333	7894	17752.66667	8425.666667
9330175M20Rik	23219	24297	26262	25444
9330178D15Rik	26185	27339	30054	28705
9330179D12Rik	29739	30972	32756	37748
9330182L06Rik	23285.8	26057	25371.2	25333.8
9330184L24Rik	32535	33982	35517	39715
9330185C12Rik	32837	27225	36706	33852
9330188P03Rik	24381	4716	36138	38852
9330199G10Rik	23852	24897	26798	35339
9430002A10Rik	3459	3697	7516	9014
9430007A20Rik	12789	36470	2285	13986
9430008C03Rik	14672	15808	15183	16840
9430012M22Rik	5046	31824	32280	38389
9430013L17Rik	17162	11348	13345	13045
9430015G10Rik	10970	2196	2033	40730
9430016H08Rik	14770	16237	13840	15411
9430018C23Rik	31147	33308	33308	31724
9430019C24Rik	5919	32133	4234	36960
9430020K01Rik	27276	24388.5	32865	33453
9430021M05Rik	27258	36702	32250	41126
9430022A07Rik	31616	9274	35501	33521
9430025N12	8549	1668	5549	200
9430027B09Rik	9244	3752	10696	9148
9430029A11Rik	24235	25381	27142	26214
9430031K09Rik	24	30880	32637	34930

9430034F23Rik	17668	11600	13573	15770
9430034N14Rik	5874	738	36008	36075
9430037G07Rik	15301	27892	22279	37154
9430038I01Rik	14674	17583	14089	13456
9430040K09Rik	10499	3847	1820	2644
9430041J12Rik	831	290	2039	1047
9430047G12Rik	37558	34810	36111	2681
9430047L24Rik	23831	24986	26494	25534
9430052A13Rik	33161	34694	37777	4335
9430062P05Rik	29965	36431	32315	30960
9430064G09Rik	3748	8907	1826	4795
9430065F17Rik	28247	29940	7194	28781
9430069I07Rik	15336	17210	19881	18646
9430070O13Rik	4608	5691	14556	881
9430076C15Rik	27991.5	14899	12799	17121.5
9430076G02Rik	35604	2450	1778	1878
9430077A04Rik	39811	34394	36209	1235
9430079G20Rik	16989	22682	23215	28446
9430081G06Rik	25303	26603	28111	38359
9430081H08Rik	36701	40684	35544	36875
9430083B18Rik	10278	4139	38797	24969
9430085L16Rik	38462	7661	32138	6151
9430087N24Rik	41011	31817	36317	31187
9430091E24Rik	31751.5	29994.5	27898.5	29967.5
9430097D07Rik	1564	36112	28669	27995
9430098F02Rik	1937	35573	36355	38998
9430099H24Rik	34763	24996	28090	35862
9430099M06Rik	24861	26044	27995	26809
9530001N24Rik	22805	19255	10165	39990
9530001P21Rik	25816	21474	24840	22766
9530002B09Rik	30764	32901	33085	31463
9530003J23Rik	7679	6008	7569	15728
9530004P13Rik	9532	12558	22667	21410
9530006C21Rik	12242	19474	18153	28190
9530009G21Rik	9839	6095	530	5164
9530010C24Rik	28812	38287	32255	38017
9530013L04Rik	28758	27824	30069	940
9530018F02Rik	13023	33375	5200	1360
9530018H14Rik	9696	12647	15894	5658

9530019H20Rik	33129	34684	37113	35247
9530020O07Rik	30351	14314	34477	38286
9530022L04Rik	29823	31487	33389	31593
9530026P05Rik	27090	28366	31650	30056
9530029O12Rik	10506	2417	7337	7075
9530039L23Rik	22952	30344	36691	39590
9530048J24Rik	6413	11222	14643	14830
9530049O05Rik	7706	27229	38970	1585
9530051E23Rik	27419	29145	29106	28073
9530051G07Rik	24924.5	26142	27959	26879
9530052C20Rik	30734	39791	3673	37195
9530053A07Rik	20913	22944	28513.5	29250.5
9530053H22	33215	34774	11380	12255
9530053J19Rik	6976	40720	3369	3787
9530056K15Rik	37133	33846	36579	27803
9530059O14Rik	5423	4101	33204	2837
9530062K07Rik	32170	33696	39876	34806
9530067L11Rik	26586	27777	30675	29239
9530068E07Rik	7837.333333	8713	3751.666667	18664
9530076L18	7833	5180	17718	23088
9530077C05Rik	34952	34814	27342	26371
9530080O11Rik	22756	18236	35442.5	19433
9530083O12Rik	17294	27350	22257	26219
9530085L02Rik	5923	9836	12512	14026
9530091C08Rik	22800.33333	13993.33333	27991.66667	27588.33333
9530097N15Rik	23526	24568	31686	25429
9630001P10Rik	8546	2716	16201	6770
9630003H22Rik	932	8370	34063	8303
9630010G10Rik	21297	16289	13253	17542
9630013D21Rik	5045	29823	13747	7635
9630013K17Rik	23485	24550	26464	28150
9630019E01Rik	15892	4464	3939	7958
9630021D06Rik	31649	10821	40238	9270
9630023C09Rik	27927	35947	37293	30003
9630025F12Rik	28488	29939	31063	34468
9630028B13Rik	30337	32168	32696	31231
9630028I04Rik	6298	33192	2142	5757
9630030I15Rik	718	5717	13715	9174
9630033C03Rik	13106	9001	7683	6939

9630039A02Rik	31729	33516	34960	33946
9630041G16Rik	36850	5820	3461	9318
9630041I06Rik	27665.5	29085	32719.5	29274
9630050P21Rik	14942	18702	19579	24577
9630055L06Rik	7979	25559	21251	36120
9830107B12Rik	14826	14860	25892	24840
9830144P21Rik	5012	142	2489	6471
9830147E19Rik	14943	18206.5	21090.5	21593.5
9830163H01Rik	36884	14657	22742	18689
9930004E17Rik	25040	26385	27727	26734
9930012K11Rik	36621.5	36883	33282	36604.5
9930014A18Rik	35745	36309	35100	33272
9930017N22Rik	18901	11765	12053	21472.5
9930021J03Rik	7648	6188.5	6608.5	27047
9930022D16Rik	17439.5	19083	21877.5	26373
9930024M15Rik	24264	12411	20585	13975
9930032E11Rik	32756	34314	35656	33785
9930038B18Rik	4116	40971	21503	22501
9930104L06Rik	39129	1006	6257	5906
9930111J21Rik2	14407	17883	17877	19134
A030003K02Rik	22456	598	29900	3693
A030003K21Rik	14861	15157	21016	21985
A030005K14Rik	5863	3411	11917	10037
A030005L19Rik	39645	131	3102	5358
A030007N12Rik	2029	40299	37084	36787
A030009H04Rik	10355	14198	15770	18754
A030010E16Rik	2323	19498	8918	10731
A030011A13Rik	2712	575	6106	6833
A030012G06Rik	29373	30892	32370	30872
A030014E15Rik	19584	20698	27055	30886
A1300008O04Rik	11138	6899	11978	13749
A130001C09Rik	33649.5	22995.5	34109	33577.5
A130004G07Rik	24924	15728	21410	26445
A130006I12Rik	7718	2847	14720	15759
A130009I22Rik	28407	32493	31433	30337
A130010J15Rik	13410.5	16427.5	16582.5	13544
A130012E19Rik	26443	27556	30664	29230
A130012F09	36436	27672	36798	29685
A130013F12Rik	18414	17900	18720	17774

A130014H13Rik	25914	27101	29551	28235
A130015J22Rik	24616	25799	27629	26456
A130019P10Rik	13756	9262	5358	5715
A130022F02Rik	29093	28179	22810	27749
A130022J21Rik	6741	41081	5684	5846
A130023I24Rik	36450	40021	1469	38802
A130038J17Rik	8289	1115	36231	8093
A130042O14Rik	35289	26084	27047	26037
A130050O07Rik	1026	5055	13781	12034
A130064L14Rik	26970	28457	40956	32635
A130072N09Rik	1437	39917	4525	39698
A130074J08Rik	330	3661	1193	7700
A130079P16Rik	29857	31936	31675	30209
A130082J08Rik	1837	34307	11020	12557
A130096G17Rik	27142	28701	30019	28526
A130099P19Rik	31824	13359	16892	14649
A1bg	37044	38701	9391	254
A1cf	36312	28727	32433	36201
A230006K03Rik	31774.66667	30466	10366	24167.33333
A230009B12Rik	38113	29518	29595	35416
A230046K03Rik	18633.66667	7431	8321.333333	16351.33333
A230050P20Rik	17402.66667	12110.66667	18674.66667	16575.33333
A230051N06Rik	24624	242	10722	7154
A230055C15	21305	20619	28205	27337
A230057D06Rik	27152.33333	28663.33333	21448.33333	19471.66667
A230059G12Rik	7350	12017	11006	11508
A230059L01Rik	36276	27696	30340	28954
A230060L02Rik	406	26247	37883	7276
A230062I15Rik	37681	16333	22799	10991
A230066D03Rik	10859	10592	40982	8016
A230070E04Rik	25960	38085	29424	39453
A230072C01Rik	8389	8299.5	14569.5	12281.5
A230072E10Rik	19364.5	22009	28694.5	28584
A230081H15Rik	37120	29934	29517	9721
A230083G16Rik	1065	40583	32102	3408
A230092J17Rik	38087	28605	29915	32456
A230101C19Rik	32555	23777	3897	32378
A230103O09Rik	28967	30361	32932	31332
A230106O10Rik	33299	33855	35665	33791

A230107N01Rik	24570	19763	22398	12861
A230107O07Rik	27351	29097	29190	28266
A2m	29182.5	30563	34490	31217.5
A330008L17Rik	27075	33400	16218	31458
A330023F24Rik	26471	24858.5	28370.5	30475.5
A330032B11Rik	420	205	2211	817
A330032P22Rik	24116	25189	27351	26194
A330033J07Rik	9576	14113	16856	18596
A330035P11Rik	29086	30561	32523	36647
A330041D05Rik	29355	926	34313	8892
A330041J22Rik	33164	35127	36176	34148
A330041K01	14305	17253	18618	20221
A330043J11Rik	14190	14427	27533	26602
A330044P14Rik	29588	30955	32306	31417
A330050B17Rik	30506	629	35291	29380
A330053N03Rik	26650.5	28899	31192.5	31906
A330070K13Rik	12335	14809	17465	15994
A330072L02Rik	26922	28145	31100	29623
A330074H02Rik	29655	31201	32143	30737
A330076H08Rik	27598.5	29040.5	30433.5	29094
A330078L11Rik	17917.5	22483.5	36672.5	20697
A330084C13Rik	6024	14201	6219	40289
A330093E20Rik	28312	29700	32937	952
A330102K18Rik	33869	25675	27788	26614
A330105O20Rik	10884	39666	1188	1517
A330106F07Rik	18990	7106	16935	16321
A330107A15Rik	26686	28359	28493	27538
A430005L14Rik	10750	14147	15113	13806
A430010J10Rik	24528	20637	23395	24450
A430023P06Rik	40074	5215	37957	10117
A430027C01Rik	3313	40160	6816	6878
A430033K04Rik	388	36043	25688	37328
A430034D21Rik	31872	32273	32623	31184
A430035B10Rik	23602	23440.33333	11401.33333	26424.33333
A430036H03Rik	25569	26679	29209	27966
A430046D13Rik	22487	22839	25107	23449
A430050A11Rik	27944	30595	30674	37189
A430057L12Rik	22626	22915	18703	25894
A430057M04Rik	39261	39467	36470	181

A430057O09	27059	28471	30163	34771
A430061O12Rik	6927	23824	31944	37212
A430062P19Rik	38226	39885	27017	26082
A430068E04Rik	13309	20413	4498	12755
A430071A18Rik	35771	39522	19324	10355
A430072C10Rik	23607	24734	40302	27548
A430081F14Rik	22540	29335	24667	20519.5
A430085C19	8139	7952	7205	3591
A430089I19Rik	29363	2770	2945	2881
A430090L17Rik	32509	32000	33955	30692.5
A430093F15Rik	35660	8738	2089	8625
A430103D13Rik	5628	34	37682	38442
A430106A03Rik	26797	28247	29527	28482
A430106F12Rik	16057	1669	9740	11400
A430107P09Rik	28922.16667	29455.83333	22093.5	21842
A430108G06Rik	14724	9164	21513	12613
A430110C17Rik	27847	29691	6774	32823
A430110M15Rik	27793	10018	21847	21251
A4gnt	10882	4244	8128	12298
A_51_P101283	28911	20808	32731	33340
A_51_P103133	15125	13596	19006	11881
A_51_P111434	35768	5445	16140	5155
A_51_P114236	37744	446	31489	1296
A_51_P114403	18786	20478	17174	19407
A_51_P114407	36984	30447	39999	33637
A_51_P115192	33822	37199	39655	917
A_51_P116157	23956	27975	28893	34409
A_51_P116226	22172	22765	26921	26398
A_51_P133148	39330	1243	5185	2007
A_51_P144143	8089	7926	10030	14634
A_51_P151675	13940	11948	6934	5841
A_51_P151888	590	5853	3438	5540
A_51_P153702	39807	1003	9806	7267
A_51_P157250	10073	9817	13872	13502
A_51_P157496	34892	25771	8820	8475
A_51_P163045	35795	32154	37697	1746
A_51_P169576	34146	38121	37718	35602
A_51_P170529	875	1251	32341	3741
A_51_P174350	23961	36149	32902	1773

A_51_P182813	10136	15992	16260	18088
A_51_P183701	3538	8027	13338	13523
A_51_P183703	34680	35769	25821	24480
A_51_P188401	28234	27972	406	38646
A_51_P191116	9953	9829	20438	20496
A_51_P191154	35637	1057	7763	27508
A_51_P191170	7971	16353	8030	16690
A_51_P191274	39418	27095	7296	551
A_51_P198596	40677	2860	11262	6396
A_51_P200564	23601	22812	22569	21764
A_51_P211758	33063	34718	36694	34888
A_51_P221938	25240	33005	29738	26231
A_51_P222946	18995	22107	25858	24136
A_51_P226791	5336	39790	6215	1277
A_51_P237890	11438	11463	16518	15071
A_51_P246305	33749	31122	27570	30913
A_51_P256798	16434	13233	16837	20731
A_51_P256818	33907	26893	25689	30817
A_51_P262374	19341	15203	15380	12223
A_51_P263137	15524	18134	23310	20126
A_51_P264139	36081	34939	81	39224
A_51_P269045	33548	38879	26793	30417
A_51_P273449	1460	38188	14295	16954
A_51_P277048	30283	36790	693	39002
A_51_P277241	38951	11298	38697	33569
A_51_P281057	30258	17069	15904	28687
A_51_P281078	28406	29893	31411	30007
A_51_P284741	2817	2259	3564	4918
A_51_P292332	15317	18121	23431	22665
A_51_P295858	21745	20298	21516	23579
A_51_P297993	18139	36009	23360	28456
A_51_P301713	8490	11375	9667	10996
A_51_P305138	4394	27208	24175	7087
A_51_P305350	23039	20360	29359	26978
A_51_P305453	3587	1460	7905	6463
A_51_P306031	23020	18605	21542	17613
A_51_P310910	23299	23284	22891	23804
A_51_P311836	18212	6901	21326	18713
A_51_P320477	21431	22423	26438	26055

A_51_P326259	24579	25884	27148	26204
A_51_P333594	40105	1709	2674	4144
A_51_P338963	1293	8496	7519	10337
A_51_P342773	21248	35785	15786	36917
A_51_P342782	22267	20355	30170	27603
A_51_P346472	13136	11346	11405	10657
A_51_P348258	9949	1998	13679	7025
A_51_P350172	16512	11911	16110	9915
A_51_P352196	7846	10417	11588	13751
A_51_P370934	19956	10146	7429	9654
A_51_P375418	37768	29351	37955	37258
A_51_P376478	37900	5146	27553	39977
A_51_P376590	2739	34477	39960	33140
A_51_P378501	34280	40009	14186	18444
A_51_P378531	4052	7752	10048	10280
A_51_P380350	28462	33525	37610	36485
A_51_P383950	3868	24855	1795	34531
A_51_P384066	6194	8126	4479	11661
A_51_P386003	12371	18288	21719	33928
A_51_P387913	36517	12680	33186	32355
A_51_P393544	11624	2735	8718	15082
A_51_P395519	6262	10433	16143	9103
A_51_P402908	3595	4864	3095	8130
A_51_P409327	30048	24426	26452	34849
A_51_P410404	29265	37029	40202	37817
A_51_P410421	10835	9430	12841	19863
A_51_P414188	3000	7633	12101	14214
A_51_P421578	10078	13399	14783	15494
A_51_P426986	27385	18942	22009	21650
A_51_P428842	2373	16865	17136	14104
A_51_P444015	40660	37165	3872	4292
A_51_P445924	9911	17767	15028	18044
A_51_P447568	13071	13486	14460	15614
A_51_P451416	22062	26485	35797	35814
A_51_P451508	15604	18985	18046	21094
A_51_P458800	13132	9572	14312	13950
A_51_P460622	16913	18276	11567	17468
A_51_P462771	33434	4957	29311	36123
A_51_P464621	20586	23222	1669	14911

A_51_P478544	12099	17693	16271	19895
A_51_P486046	37502	39773	38317	39710
A_51_P487137	31240	27028	39532	8365
A_51_P499979	4963	7497	12969	8643
A_51_P507637	25919	38295	23016	34883
A_51_P510301	25486	5076	29294	39071
A_51_P515046	6882	13839	12674	17215
A_51_P516033	10965	14050	17467	18279
A_51_P517720	194	37580	38744	38386
A_52_P1004880	30768	32837	34080	40618
A_52_P1005171	34324	1988	12734	7370
A_52_P1013232	18136	40045	7324	33051
A_52_P1013396	26754	2209	18021	4088
A_52_P1013441	36026	40700	39959	134
A_52_P1013608	13310	14811	25298	22819
A_52_P101501	31272	32995	34562	33122
A_52_P101878	31097	26391	27950	26989
A_52_P1021224	39278	150	4156	3583
A_52_P1021640	9	38044	40822	785
A_52_P1029561	23359	24437	27735	30112
A_52_P1037106	21213	17076	18736	15592
A_52_P1045181	1122	3541	4041	5053
A_52_P1053015	9810	18547	6682	17878
A_52_P1053094	18288	19990	25765	24753
A_52_P1053544	20788	17448	22361	21164
A_52_P1053662	23178	26794	26809	26460
A_52_P1061635	4648	2711	11561	16977
A_52_P1069249	14807	17774	15103	17549
A_52_P1069333	7891	14759	23142	23415
A_52_P1077067	16273	40654	23060	23845
A_52_P1077588	38014	39367	35705	39313
A_52_P1085448	2795	37133	16446	15403
A_52_P1101014	16550	16505	21250	20752
A_52_P1101021	29747	26342	14092	13325
A_52_P1101277	35886	1491	4729	5428
A_52_P1101414	3025	13560	20428	19783
A_52_P1101503	26702	922	39323	7292
A_52_P1108967	14164	15849	21977	22455
A_52_P1117630	37621	23872	25134	21568

A_52_P1119090	17190	22595	28836	28316
A_52_P1125131	10971	8260	16374	13668
A_52_P1125306	34386	31615	33121	31633
A_52_P1133381	11148	12429	15146	14313
A_52_P1141039	14828	17818	19078	18271
A_52_P1141438	5888	40244	40941	4321
A_52_P1148855	19713	38960	26973	31699
A_52_P1165294	6217	9933	13727	14925
A_52_P1165687	38823	2497	3311	4196
A_52_P1173559	12192	5799	7584	4277
A_52_P1173798	14067	16225	21401	24992
A_52_P1181407	7267	6734	12736	12262
A_52_P1189114	21585	21208	25452	22560
A_52_P1197192	16809	38399	21457	589
A_52_P1197206	11757	4961	6580	8648
A_52_P1197466	7224	36609	34030	2915
A_52_P131372	37526	3061	3492	2764
A_52_P13337	9207	13369	14935	15727
A_52_P139439	33547	40292	3302	3535
A_52_P174368	2261	5026	8756	8218
A_52_P182347	26776	28372	28912	27901
A_52_P18720	34727	31246	33261	39125
A_52_P187677	36493	33394	33526	38159
A_52_P187690	16778	22531	17438	22616
A_52_P190357	18642	21794	23733	22607
A_52_P214516	18456	19311	25781	28352
A_52_P2659	22671	19618	21193	22458
A_52_P281617	39001	38173	27140	29836
A_52_P335218	8429	4062	35945	34725
A_52_P362072	29573	38818	2832	865
A_52_P372762	25613	27238	33698	26464
A_52_P383526	3135	1521	33443	386
A_52_P40168	29635	36905	36867	34343
A_52_P404930	11601	13211	18764	19255
A_52_P404979	11845	10847	10627	7773
A_52_P407668	22134	35248	25271	31059
A_52_P410404	25915	27255	28941	27716
A_52_P437215	18091	21209	18026	17868
A_52_P442601	37379	39291	7556	5970

A_52_P453315	10673	12799	18666	17475
A_52_P453344	21054	19516	24178	19852
A_52_P469316	1941	218	4566	5596
A_52_P477359	20754	22319	37062	30941
A_52_P477386	15517	12700	15095	16417
A_52_P480044	33033	37336	36292	37099
A_52_P490470	7727	36631	11095	13320
A_52_P501586	25664	26926	28601	27468
A_52_P504217	6688	3947	11010	8836
A_52_P506920	39139	32167	8794	1478
A_52_P517668	37217	33778	40541	37392
A_52_P53626	36924	178	39270	41062
A_52_P541760	10891	12880	20355	18505
A_52_P549754	1857	38634	2074	37586
A_52_P576678	6540	12781	10972	11070
A_52_P587462	36875	2707	6643	1545
A_52_P589808	3608	9277	10423	12541
A_52_P641123	27851	26162	2104	2460
A_52_P649151	21474	20336	32117	35691
A_52_P649214	12891	15757	15948	21372
A_52_P692753	22109	20118	21071	18287
A_52_P700200	40362	39852	35079	37169
A_52_P708886	39474	40588	38093	37460
A_52_P716487	33032	39243	630	4170
A_52_P716654	24169	19239	26166	24703
A_52_P716675	18733	23805	22126	28230
A_52_P724603	37843	39313	39388	573
A_52_P724652	10386	12616	15879	15041
A_52_P724703	20813	22759	26071	24424
A_52_P724724	1878	8084	8013	9149
A_52_P724737	40682	30179	9702	4209
A_52_P732274	7120	13529	18469	24739
A_52_P732433	2563	39147	910	3061
A_52_P732508	40066	38698	38374	40197
A_52_P748402	9170	3639	4596	5386
A_52_P756169	6573	3304	29941	37087
A_52_P756294	14205	17365	22397	21700
A_52_P756747	24495	25808	27096	27548
A_52_P764117	29761	31998	32394	37545

A_52_P764315	22884	22953	24509	27780
A_52_P764477	7980	37355	2881	40387
A_52_P764584	13653	15744	15152	16252
A_52_P771912	24094	31295	27331	37097
A_52_P772584	22852	20061	21147	19562
A_52_P772671	27267	31241	32134	35165
A_52_P788434	16059	16459	24848	23402
A_52_P796659	33852	29653	2910	38509
A_52_P796719	36820	38113	7709	7129
A_52_P804224	11078	1380	3057	135
A_52_P804370	21394	21571	39601	39117
A_52_P812212	9665	12501	18819	20254
A_52_P812372	34257	29354	33992	33093
A_52_P812648	10156	6699	2768	39832
A_52_P812717	17161	22864	22187	24575
A_52_P812870	9937	10841	11814	11043
A_52_P820445	9698	5973	17801	16957
A_52_P828639	7796	37548	26041	26867
A_52_P836776	30547	15511	23833	24514
A_52_P836919	25434	29623	27477	27625
A_52_P837066	781	6122	8246	12011
A_52_P844271	26128	27886	38868	37594
A_52_P852322	15718	19785	15077	16952
A_52_P852457	2125	32229	24878	26337
A_52_P852662	4510	6861	33758	35464
A_52_P852771	17006	18349	36130	35410
A_52_P854619	35050	40535	5890	5842
A_52_P860410	3065	8322	11028	39678
A_52_P860487	5534	4135	7346	5587
A_52_P860519	15474	10256	14394	14500
A_52_P861158	24798	26161	28376	35840
A_52_P868705	36747	27590	39833	38041
A_52_P868798	37802	36267	1067	40357
A_52_P876692	25142	24132	24196	27962
A_52_P877058	28095	29343	31426	32577
A_52_P877097	5685	6490	10947	11401
A_52_P877171	16663	19072	24818	27950
A_52_P885203	36365	2294	29574	2197
A_52_P892476	5616	8556	15015	15753

A_52_P893105	2801	1746	39296	41055
A_52_P901184	2122	631	15586	12390
A_52_P917036	36063	23716	21638	23208
A_52_P924462	17357	16064	19387	22357
A_52_P925012	38033	8116	9144	14328
A_52_P925038	15991	15844	20969	26320
A_52_P932574	17475	17550	21936	20852
A_52_P932685	23926	26019	39143	38410
A_52_P932984	22095	39029	38593	3860
A_52_P940816	36290	29264	27485	35871
A_52_P941191	35778	37324	4906	2815
A_52_P941235	10671	7701	11092	13684
A_52_P949155	15223	22088	17735	15554
A_52_P957248	9620	11580	11442	10665
A_52_P96577	19754	20034	23685	22075
A_52_P980916	7035	10379	14443	14864
A_52_P980948	21027	25319	24805	24755
A_52_P981004	15338	20897	25026	24308
A_52_P981018	22622	24288	34750	32870
A_52_P981179	40407	2924	24522	33837
A_52_P981301	12933	12345	18602	17629
A_52_P988817	31963	36628	41121	40336
A_52_P988880	40063	32549	34251	32502
A_52_P989183	18505	23566	24531	30523
A_52_P989547	36862	39832	10328	8837
A_52_P996776	39236	272	409	4309
A_52_P996797	12764	9560	12432	13505
A_52_P997193	14301	3340	8020	12165
A_52_P997449	11471	5241	5707	8348
A530010F05Rik	28249	29962	30079	28936
A530013C23Rik	8543	11542	14829	13064
A530016L24Rik	3539	4348	5851	7608
A530017D24Rik	20009	16579	7897	4895
A530020G20Rik	13988.5	17661.5	21905	20667.5
A530021J07Rik	32202	34019	35178	33373
A530028O18	8689	4224	16543	9314
A530032D15Rik	12397	16384	15417	11470
A530040E14Rik	35155	1650	30623	33042
A530041M06Rik	7290	39099	5468	5122

A530053G22Rik	24120	25195	27364	26202
A530058N18Rik	15336.75	9819.5	25422	19509
A530064D06Rik	22040	36897	12319	33839
A530076I17Rik	32514	33868	37003	35043
A530079E22Rik	27863	29663	29725	28416
A530083I20Rik	24850	34965	899	7461
A530088E08Rik	14072	38103	32447	27723
A530099J19Rik	14479	13853	22578	18392
A630001G21Rik	18197	14523	20107	16962
A630005I04Rik	35206	30996	25594	27311
A630006J10Rik	22644	22221	32900	19332
A630012P03Rik	31965	33607	35373	33526
A630020A06	26837	28190	30830	39501
A630023P12Rik	31485	31772	39027.5	25956
A630026N12Rik	30774	8767	11371	11005
A630031M04Rik	38329	35865	37942	6629
A630031M23Rik	39072	20276	19553	12489
A630033H20Rik	23045	18474	23581	23812
A630038E17Rik	30971.5	18362.5	19711.5	21767
A630052C17Rik	39018	34211	39890	40264
A630057J21Rik	35884	2304	35900	3072
A630057N01Rik	25034	33626	16611	18866.5
A630072M18Rik	20825	5134.5	31923	20796.5
A630077J23Rik	12024	12784	17582	12056
A630081D01Rik	34418	21610	21078	33124
A630089N07Rik	12542	11270	12444	14372
A630091E08Rik	17340	19843	21667	23267
A630095E13Rik	38696	4240	7996	10369
A730008I21Rik	32732	34809	35131	33327
A730009E18Rik	39584	39592	3682	4513
A730011C13Rik	31554	28128	32423	34364
A730013G03Rik	31077	25692	5922	7349
A730017C20Rik	29392	28403.66667	34739.33333	34438.66667
A730017L22Rik	21092	26123	27783	971
A730018C14Rik	856	38693	7381	7732
A730020M07Rik	26996	28419	30089	35240
A730021G18Rik	33922.5	16666	20055.5	32267.5
A730027B03Rik	33073	22130	27037	34321
A730028G07Rik	34962	31275	28691	33326

A730036I17Rik	10013	1030	4955	11722
A730041O05Rik	28088	25089	27446	25046.5
A730046J19Rik	28403	29845	31562	30049
A730049H05Rik	20315	24821.5	26241.5	12628
A730054J21Rik	30225	31892	36325	2870
A730056A06Rik	38781	5903	1612	12318
A730059M13Rik	27452.5	28878	18394.5	29363.5
A730063M14Rik	35817	29616	34184	30973
A730081D07Rik	5632	9862	14428	15955
A730089K16Rik	31096	32796	37166	32693
A730090H04Rik	30330	32151	32723	31252
A730090N16Rik	6627	3431	9917	9603
A730093L10Rik	27949	36962	9116	2781
A730094K22Rik	27421	28787	31974	30334
A730094L24Rik	41003	3619	2862	6580
A830005F24Rik	22656	35152	34873	35089
A830009L08Rik	9225	30644	31591	30155
A830010M09Rik	23634	36068	26337	25370
A830010M20Rik	15978.33333	16652.33333	14324	17191.33333
A830012C17Rik	21695	23990	38716	36018
A830018L16Rik	29492.25	20648.5	19616.75	31013
A830019P07Rik	31991	33688	40814	3547
A830021K08Rik	13105	24913	10438	29766
A830021M18Rik	29681	33848	35345	37256
A830031A19Rik	37579	1164	2722	10812
A830039N20Rik	21210	19015	24327	24248
A830054O04Rik	23756	32290	26691	31037
A830054O07Rik	31792	33739	33914	529
A830060N17	24238	38205	27218	36148
A830073O21Rik	14996	17364.33333	20146	20406.33333
A830080D01Rik	14165.5	14037	14337.5	17047
A830080L01Rik	15111	3141	36575	40582
A830082K12Rik	24445	25577	27462	26310
A830091E24	10901	8018	4688	7311
A930001A20Rik	35877	36274	7012	13134
A930001C03Rik	6577	30	5244	3938
A930002H24Rik	12545	9151	13325	11601
A930002I21Rik	37857	14276	19517	19487
A930003A15Rik	23685	24747	26665	25646

A930003O13Rik	30138	31670	33003	31519
A930005C09Rik	18274	20218	35965	32174
A930005G22Rik	31266	33421	34530	32585
A930005K07Rik	23408	24453	26544	25410
A930005N03Rik	32327	34181	37925	34329
A930006I01Rik	13137	31242	31793	30390
A930006J02Rik	25902	27519	3353	28293
A930006K02Rik	13833.5	30663	13033.5	11488
A930009E08Rik	40763	5530	39453	7698
A930009L07Rik	30927	32528	34472	37413
A930010G16Rik	18851	18544	18229	21585
A930011G23Rik	27607.66667	28505.66667	20258	20282.33333
A930011O12Rik	2964	16033	50	11228
A930012O16Rik	3634	7689	7382	6648
A930016O22Rik	9066	9826	12838	9097
A930017K11Rik	6784	8781	13521	17642
A930017M01Rik	14256.5	21762	32682	22603.5
A930018M24Rik	28737.5	30322	31236	29934
A930018O16Rik	28631	30491	30235	29053
A930018P22Rik	35398	39071	3988	5299
A930019D19Rik	27046	28291	40168	32406
A930019H14Rik	33024	34958	36494	1856
A930024E05Rik	39437	39743	5722	4159
A930025A13Rik	1985	36627	3231	33854
A930025H08Rik	4817	34634	3012	36392
A930033H14Rik	19993	16682	15562	17516
A930035E12Rik	28605	29380	28474	34578
A930036M01Rik	31552	33709	33901	32326
A930037H05Rik	27834	29339	30697	29534
A930037O16Rik	29651.5	31282.5	32518	30936.5
A930038B10Rik	25843.5	26904	32317	29950
A930038G18	2853	8813	11295	11198
A930041C12Rik	34612	31795	33216	36393
A930104D05Rik	24411	25519	27657	26453
AA438147	21569	7363	16486	18657
AA545095	11450	14527	28765	36774
AA674562	11504	12797	16229	21072
AA718294	37834	23374	30114	27413
AA763164	40049	36202	38402	36425

AA792892	24434	24927	37341	32894
AA986860	14791	11133	17069	13180
Aaas	21600	2193	3022.5	23247.5
Aacs	39397	40086	31021	28778
Aadac	15254	19731	23836	22384
Aadat	12864	7541	19792	20089
Aaed1	6646	12514	7716	8630
Aagab	6610	2553	11315	6909
Aak1	17435.6	14356.4	14657.6	9129.4
Aamdc	25940.66667	26416	27130	25930.66667
Aamp	13055.5	31584.5	31728	31808.5
Aanat	34857	20254	2774	3350
Aar2	30578	28520	22486	24412.5
Aard	8967	15989	11969	20339
Aars	22482.6	20250.8	20012.6	20160.8
Aars2	36000	35012	29130	32595
Aarsd1	19038	22732	20132	22924
Aasdh	32211	26688.33333	25911	29300.33333
Aasdhppt	12928.33333	18108.66667	15506.33333	17577.66667
Aass	7834	11895	13113	11690
Aatf	21303.33333	21097	32846.33333	22633.66667
Aatk	29139	19196	25330	19076
AB010352	3528	2785	39502	639
AB041803	13259.5	25214.5	20447.5	11325.5
AB041806	1	40237	9527	8183
Abat	34540	35575	31497	30012
Abca1	7125	13816	7881	18340
Abca12	27596	29424	31771.33333	32622.66667
Abca13	32545.33333	24554.66667	19026	29874.66667
Abca14	13693	28281	14202	18125.33333
Abca15	21169.5	23521	24352	25516
Abca16	32824	34454	39201	34759
Abca17	6982	16247	6032	7254
Abca2	31758.5	27207	22857	22075.5
Abca3	20380.75	22213.75	19838	22141.5
Abca4	2149	15805	30308	28911
Abca5	13610	21812.2	17730.8	17265.6
Abca6	21065	34659.5	10844	26440
Abca7	3086.5	6066.5	3358	6351

Abca8a	19096	19383	29664	24310
Abca8b	22866.5	20746.5	32174.5	29914
Abca9	23921	15804	28198.75	15659
Abcb10	13195	29258	26852.5	27767.5
Abcb11	38874	38117	6517	5033
Abcb1a	15420	28069.5	31737.5	12473.5
Abcb1b	8391	31002.66667	31012.33333	27490
Abcb4	5276	14449	20657	15615
Abcb5	25603	26896	35668	27458
Abcb6	1644	4311	533	6053
Abcb7	17749.66667	30008	31516.66667	30963.66667
Abcb8	22773	18568.5	16186	39378
Abcb9	34153	31009	32373	28485
Abcc1	26170.33333	30673.66667	23210.66667	30833
Abcc10	39524	33750	35617	33967
Abcc12	29158	18594.5	3318	28473
Abcc2	29791	39171	31795	35280
Abcc3	36686	23448.5	27270.5	21020
ABCC3	31283	32952	34108	2655
Abcc4	10761.5	14761.5	6296	13753.5
Abcc5	16800.5	22121.25	7670.25	17666.75
Abcc6	39086	5109	33518	7992
Abcc8	9636.5	9032	22607.5	18284.5
Abcc9	30455	31408.33333	30846.66667	29467.33333
Abcd1	22632.5	21541	18256	18615.5
Abcd2	14033.5	16798	7095	29146
Abcd3	12775.25	18891.5	22999.5	14481.25
Abcd4	31981	23279	26642	26099
Abce1	20450	19946.5	33222	18971
Abcf1	9428	9631	11472	12509.5
Abcf2	21100	21528	19913.5	23056.5
Abcf3	26093.5	19401	20072	21081.5
Abcg1	20727	13778	22863	18049
Abcg2	5519.5	19143.5	18020.5	31345
Abcg3	16278	35205	5913	25857
Abcg4	32619	41117	40764	3290
Abcg5	17680	20658	33508	34606
Abcg8	23358.5	24616	24387.5	9063.5
Abhd1	19299	19310.5	19235	21440.5

Abhd10	17453.33333	16139.66667	17937.33333	16240.66667
Abhd11	12913	16298	16770	16273
Abhd12	19181.5	40140.5	20894	18990.5
Abhd13	16725.33333	14902	14753.66667	15979.33333
Abhd14a	33814	22872	27441	28584
Abhd14b	38250	24501	12942	9574
Abhd15	30931	13550	26030	32518
Abhd16a	17137	19976	13986	13575
Abhd16b	36638	37540	15218	15270
Abhd17a	35882	36039	26063	21542
Abhd17b	19057.33333	21042.66667	18113.66667	21058
Abhd17c	16219	20477	12240	19409
Abhd2	16053.33333	19474.33333	18891	24012
Abhd3	19979	23997	19673	22937
Abhd4	21925	21285	21732	23683
Abhd5	10160	12215.5	8544	10572.5
Abhd6	23136	23544	26082	34753
Abhd8	20437	7457	19873.5	24721.5
Abi1	16602.33333	17639.33333	18671	20111.33333
Abi2	13212.33333	21255	10969	22231.66667
Abi3	29668	3600	26567	3774
Abi3bp	29116.33333	33596	32237.33333	31361.33333
Abl1	21612	20753.33333	8447	5169.333333
Abl2	17058.5	22874	24058.25	22824.75
Ablim1	22653.14286	17192	20870.28571	25175.28571
Ablim2	21537.5	10901	27668.5	14375.5
Ablim3	20152.5	22157.75	21922	24146.5
Abo	19737	20738	18830	27166
Abpa	37071	38230	2680	38882
Abpb	13360.5	16875.5	16137	18861
Abpd	26051.5	29733.5	35411	21960.5
Abpe	31492	38566	10988	4756
Abpg	30006	21401	34432	32664
Abph	29516	31003	33430	31793
Abpz	31303	36668	34669	33857
Abr	38876	16094.5	36482.5	19385
Abra	26907	28678	29074	36695
Abracl	13388	10797	27760	18495.33333
Abt1	13333	17315.33333	22277.33333	22271

Abtb1	16025	13215	12306	10626
Abtb2	27644.5	21051	35806.5	22463.5
Acaa1a	23864.66667	27828.33333	17653.33333	26361
Acaa1b	19077	22236	20739	29843
Acaa2	14043	6625	8621	998
Acaca	15531.85714	25726.42857	21195.57143	28668.28571
Acacb	17627.5	18098.5	21238.5	20290.5
Acad11	23324	26979.5	22229	27788
Acad12	28603	23970.33333	23720.66667	9915.666667
Acad8	27066	21956	22141	21592
Acad9	16259	14018	10952.5	8263.5
Acadl	25443	22977	24409	25799
Acadm	7467	6494	7917	3417
Acads	9403	5590	4207	1371
Acadsb	9795	7063	6532	10250
Acadvl	6770.5	8835	28293.5	9753.5
Acan	8276	9884	12469	13080
Acap1	22272	21456	22143	27683
Acap2	14493.66667	37229.66667	21189	11661.66667
Acap3	40427	38064	37872	35852
Acat1	13049	14853	11820	10041
ACAT1	27381	29537	29393	35694
Acat2	15186	25592	19780	23726
Acat3	14574	23503	15009	19181.5
Acbd3	10278	10812	7078.333333	11819.66667
Acbd4	17991	20401	20577	20997
Acbd5	23010.5	22163.75	21746.25	22815.75
Acbd6	20520	22162	21190	22549
Acbd7	28036	20645	29604	23788
Accs	10543	5368	6206	2787
Accsl	31072	32683	37815	4356
Acd	22157	22210	25693	24923
Ace	21732.66667	25715.66667	24312.33333	24636.33333
Ace2	5201	3829	3407	6512
Ace3	29661	37704	32663	31107
Acer1	40831	11097	10591	12747
Acer2	30864.33333	17650.33333	19045	22411.66667
Acer3	24538.66667	21928.33333	24285.66667	16898.66667
Ache	13127	9002	12977	14074

Acin1	20511.75	24258.25	19847.25	27391
Ackr1	17475	23415	10795	9409.5
Ackr2	15043	16529	20785	23188
Ackr3	35324	1505	8446	15091
Ackr4	24009	27570.5	32123	32511.5
Acly	16825.5	14366	10759	10723.5
Acmsd	13831	18755	18256	21339
Acnat1	3891	512	4378	108
Acnat2	23563	24602	26720	25580
Aco1	9844	6860	6769	2485
Aco2	17426.33333	30969	19990.66667	22242.66667
Acot1	33634	13317	22109	5630
Acot10	34270	5003	38857	4898
Acot11	30919.5	26757.5	29686.5	23570.5
Acot12	31276	32380	32435	40883
Acot13	19611	22892	21514	20371
Acot2	25114	21807	21840	15139
Acot3	22779	34874.33333	22870.66667	24912.66667
Acot4	28331.5	28545	24280.5	16249.5
Acot5	40724	384	5982	6050
Acot6	8541.333333	18141	16153.33333	12242.33333
Acot7	19293	24518	17245	25738
Acot8	21999	24356	24755	30531
Acot9	9612	6017	5009.5	10905.5
Acox1	7035	6229	5260	6061.5
Acox2	11170	8068	14203	7868
Acox3	9068	4605	921	435
Acoxl	15410	16458.5	6470.5	20018.5
Acp1	22130.66667	23991.66667	34779	26032.66667
Acp2	24116.66667	19270.66667	18496.33333	27670.66667
Acp5	39082	18533	28677	14141
Acp6	22370	19017	22028	18435
Acpp	26643.66667	8152.666667	9110.333333	27854.66667
Acpt	6430	8189	9186	8884
Acr	11915	14490	15667	14349
Acrbp	18757.5	28970.5	15112.5	31248.5
Acrv1	14216	12766	19995	16522
Acsbg1	39460	35759	36100	35098
Acsbg2	1818	37075	7129	2058

Acsf2	19420	32329	22920	24504
Acsf3	15108	14692	16323	15620
Acsl1	13390.66667	23867.66667	13293.33333	28443.33333
Acsl3	7840	11361	6544	8346
Acsl4	19313.5	9792	22905	10256.5
Acsl5	286	1790	38284	39312
Acsl6	16452.25	21145	28068	26465.5
Acsm1	713	39873	30315	30489
Acsm2	26940	22787	31538.66667	26514.33333
Acsm3	20837	25021	25933	28096
Acsm4	35522	34440	6250	38906
Acsm5	35104	36485	5915	32690
Acss1	8011	6359.5	7015	10929.5
Acss2	22992.25	18865.25	25937.5	21894
Acss3	41080	2267	41032	39263
Acta1	13902	15378	13564	18977
Acta2	23181.4	15748.4	32361.8	23861.2
Actb	25413.5	16521	17253	10449
Actbl2	30385	31904	33447	31829
Actc1	12146	14620	15394	17673
Actg1	19630.5	28501.5	15108	12166.5
Actg2	22030	15219	23144	20192
Actg-ps1	5431	5189	9351	8222
Actl10	36605	3376	8771	6434
Actl6a	29308	28621	24275	23279
Actl6b	8595.5	9802.5	15150.5	16172.5
Actl7a	14040	29389	21618	36843
Actl7b	19684	23463	24704	26074
Actl9	38364	37155	1561	39798
Actn1	38814	3065	4826	6419
Actn2	1835	5385	12119	14499
Actn3	16425	12792	16923	16513
Actn4	13126	8391	13328	11355
Actr10	12734	14375	13606	14995
Actr1a	17448	14931	14924	14910
Actr1b	18769	18906.5	18656	17808
Actr2	13208	12232	13223	8508
Actr3	66	37099	3420	39954
Actr3b	10139	24160	12852	8859

Actr5	14642	26336	11929.5	30169.5
Actr6	19249	17298	17939	15998
Actr8	34079.5	39120	19827	20174
Actrt1	32201	33561	36006	34031
Actrt2	17053	18608	16743	17676
Actrt3	1893	4095	6040	5963
Acvr1	10411	6349	8667	7328
Acvr1b	24301.5	30823.5	23364	31867
Acvr1c	27731.5	30224.5	24338	23353.25
Acvr2a	31882.75	27216.75	20184.5	20231.5
Acvr2b	12020	10401	5258	5011
Acvrl1	7788	4784	38413	1700
Acy1	40320	40375	38173	3595
Acy3	6752	9879	13177	13783
Acyp1	7906	6839	12121	4833
Acyp2	7886	10173	830	40240
Ada	2987	32012	9064	38274
ADAD1	28979	30831	30967	29562
Adad2	29643.5	21353	31315	32609
Adal	10566	14400.5	28686	28092.5
Adam10	14170	15462.5	20098.5	20891
Adam11	22763	24381	29460.33333	29438.33333
Adam12	30221.5	31194	30946.75	31767.25
Adam15	22442	36841.5	15880.5	13374.5
Adam17	21194	6406.5	36390	8671
Adam18	25285.5	26823	30306.5	30575
Adam19	13723	7206.333333	15305.66667	7887.333333
Adam1a	10435	11470	7487	13582
Adam1b	26082	30627.66667	20063.66667	28434
Adam2	8136	13558	13385	16455
Adam21	33415	38827	40244	1955
Adam22	23378	24479	26256	25261
Adam23	21012.66667	23177.33333	25980.66667	26600.66667
Adam24	27791	37073	31097	1695
Adam25	20015.5	26751	21868.5	24417.5
Adam26a	31163.66667	36425	34926.33333	34571.66667
Adam28	14426	18622.5	17650.25	25819.5
Adam29	23823.5	25688.5	27919.5	27790.5
Adam3	6867	12146	13443	16184

Adam30	25176	27873	28287	29706.5
Adam32	38924	30609	447	1962
Adam33	23266	11420	16332	10313
Adam4	23939	20459	18853	24208
Adam5	9303.5	13051	17988.5	22736.5
Adam6a	3478	6118	34659	90
Adam6b	1120	3205	5281	3936
Adam7	27529	28997	36042	28734
Adam8	12177	24819	13729	1019
Adam9	14536	13425	14732	16514
Adamdec1	3002	3756	14809	12897
Adamts1	16291	15006.5	23755	25785
Adamts10	21657.33333	23336.66667	25621	17098
Adamts12	15086.5	16599.25	17654	23210.25
Adamts13	107	30423	39807	30517
Adamts14	19568	20707	22593	30961
Adamts15	38620	21055	33960	25303
Adamts16	21992	8058	14099.5	16530.5
Adamts18	7288	10389.5	14806	15037.5
Adamts19	19849.66667	29180.33333	33221.66667	30851.66667
Adamts2	18420.25	18928	22470.25	15873.75
Adamts20	21007.33333	15941.66667	14486	12559
Adamts3	35985.5	20602.5	29141.5	11516
Adamts4	10534	40546	22032	1271
Adamts5	20931	22221.5	29696.5	21892
Adamts6	19821	20866.25	20367.5	19984
Adamts7	23278	23724.5	15934	20682.5
Adamts8	13479.33333	36576.33333	25655.33333	25052.66667
Adamts9	26235	16364.66667	16719	24058.33333
Adamtsl1	22857.66667	35521.33333	19286.66667	20746.33333
Adamtsl2	10825	10540	21952	26768
Adamtsl3	15015.5	7708	9239.5	11766
Adamtsl4	2548	7712	2689	7469
Adamtsl5	14772.66667	13362.33333	11718.66667	13915.66667
Adap1	27990	28757.5	9485.5	10502
Adap2	17296	11220	15017	9328
Adar	9684	10626	8699	13258
Adarb1	22482.33333	21012.66667	25218.33333	24840.66667
Adarb2	15962	17948.66667	30877.33333	20786.33333

Adat1	17411.33333	16207.33333	8743	20420.66667
Adat2	3263	12260	10961	11937
Adck1	7146.5	5610	21227.5	19636
Adck2	2451	39424	445	38344
Adck3	14237.5	20182	16392.5	21265
Adck4	5296	3197	1527	931
Adck5	18688	18425	19020	20921
Adcy1	30237.25	30003.75	20619	27189.75
ADCY10	29442	38890	32559	39547
Adcy2	31870.5	37007.5	36304	28075
Adcy3	4977	22480	12750.5	27287.5
Adcy4	38302	36572	10860	11060
Adcy5	25412	29639	29090	27375
Adcy6	13497	12365	17940	17471
Adcy7	14412	16074	25294.66667	17452.33333
Adcy8	32712	34518	4190	2580
Adcy9	17890	11393	16655	10594
Adcyap1	34986	36701	39915	1728
Adcyap1r1	9522	11565.5	20877.5	21390.5
Add1	29473	21345.5	25187	23250.5
Add2	22502.6	22886.2	18351.2	26174.4
Add3	35639.5	25864	33190.5	26282
Adgb	9739	16572	10338	19258
Adgra1	31403	28423	29612	38307
Adgra2	38616	7010	11187	19638
Adgra3	11846	11808	12913	13807
Adgrb1	13217.5	14190	16222	16602.5
Adgrb2	18775	18155.5	28318	27250.5
Adgrb3	31347.33333	30314.33333	30667.33333	22871
Adgrd1	22460.33333	19763.33333	21117	24094.66667
Adgre1	23672	29370	21740.5	29384.5
Adgre5	5804	29369	40762	27591
Adgrf1	12164	7620	11890	8186
Adgrf2	27094	28494	30059	30416
Adgrf3	36179	34533	37287	35494
Adgrf4	29813	31316	37587	34120
Adgrf5	30972	26797	27340	29277
Adgrg1	19393	39596	1789	31093
Adgrg2	24081	25137	27564	36470

Adgrg3	22217.85714	16369.85714	18598.28571	20955.85714
Adgrg5	40226	39060	3325	3997
Adgrg6	21405	15476.5	23703.5	19203
Adgrg7	10373	3960	20018	14680
Adgrl1	25241.75	26273.75	20203	21145.5
Adgrl2	40259	25273	25833	22741
Adgrl3	25714.76923	30350.07692	29496.76923	28029.76923
Adgrl4	31853	33286	34832	33048
Adgrv1	22106.375	19994.75	25498.25	18725.875
Adh1	23265.33333	24710.66667	18408	15776.66667
Adh4	2600	2542	9836	16633
Adh5	13753	15009	11390	13702
Adh6a	29623	19778	17393	18176.5
Adh6-ps1	1356	3204	2150	8357
Adh7	36465	984	38608	4546
Adhfe1	14173	12389.5	15586.5	14579.5
Adi1	2079	39503	32675	35286
Adig	13890	13225	22906	19010
Adipoq	37392	29215	1394	8676
Adipor1	7967	4428.5	25520.5	21647.5
Adipor2	22037.33333	16849.33333	17440	15253.66667
Adk	15289.5	17731	2053	19605.5
Adm	5181	7799	12114	15083
Adm2	23739	24764	27594	25822
Adnp	23699.33333	18340	17150.33333	15238.66667
Adnp2	36556	34417	16017.5	18152
Ado	18411	18726	15223	18682
Adora1	4270.333333	5266.333333	12540.66667	10956.33333
Adora2a	26073.5	19759.5	29749	21552.5
Adora2b	29036	27447	21726	22055
Adora3	18047.66667	22429.33333	17775.66667	25706
Adpgk	40495	3203	39353	2973
Adprh	16770	18914.66667	18372	21270
Adprhl1	6441	10287	13358	13847
Adprhl2	30413	27646	22840	22690
Adprm	32910	1980	38587	41113
Adra1a	9350.5	8638	10878	11759
Adra1b	9275	8691.5	15444.5	17546
Adra1d	12064	13792	17353	17378

Adra2a	22656.66667	24623	29961.66667	29228
Adra2b	28779	30980	40712	39571
Adra2c	18524	19480	18033	20482
Adrb1	10021	23992.5	11098	22778
Adrb2	15342	8513	9397	3566
Adrb3	7100	9917	24621	22825
Adrbk1	22715.33333	22605	19662	17366.66667
Adrbk2	25395	25707.5	19746	21267
Adrm1	13935	14412	30000.5	14640
Adsl	13573	15591	12066	15472
Adss	21878.66667	19910	23958	22611.33333
Adssl1	28037	16954	26614	18620
Adtrp	21624	35566	17853	19299.5
Aebp1	920	37536	2851	1165
Aebp2	22633.5	24403	22026	24036.5
Aen	23490.66667	23968.66667	22761.33333	25923.66667
Aes	21966	22367	19766	18790
AF013969	6285.5	24167.5	4439	21470.5
AF062525	26724	22890	20563	24195
AF067063	26981.5	30814	33494	35785.5
AF085738	14197	11391	18412	18639
AF251705	25456.66667	18184.66667	30041	18754.66667
AF357471	3027	3334	3863	5111
AF366264	17555.5	36579.5	19502.5	16983
AF529169	36508	41076	32496	3832
Afap1	23362.75	23239	27195.5	28082.75
Afap1l1	5833	18922	14342	22161.5
Afap1l2	17990	11099	23460	26078
Aff1	5427.333333	29922.66667	20175.33333	18905.33333
Aff2	27378.66667	18619.33333	30734.66667	28445
Aff3	26984	29328.4	10419.6	25241.4
Aff4	28944.25	21151	22576.5	25937
Afg3l1	25112.33333	22086	19472.33333	21150.33333
Afg3l2	13264	16395.5	16136.5	20189.5
Afm	24389	30353.5	31939	18283.5
Afmid	16560	17353	15123	13905
Afp	1249	29786	7929	34986
Aftph	19033.66667	15749.66667	13949	15024.66667
Aga	30987	37641	35574	39417

Agap1	36235.5	32955.25	29676.25	31805.75
Agap2	29313	28057.5	13003	10681.5
Agap3	29985.5	29283	29430.5	27452.5
Agbl1	16991.5	16766	27330	20450.5
Agbl2	30483.5	33988.5	34681	30464.5
Agbl3	15964.5	22198.25	16612.75	22549.25
Agbl4	32419	33735	35918	33962
Agbl5	12756.5	14518	16799	16942.5
Ager	15200	13795	10916	9120
Agfg1	37658	35562.5	31837	36249
Agfg2	19664.66667	16343.66667	19207.66667	16281.33333
Aggf1	18412	19281	19575	19361
Agk	18979	14414	13511	12744
Agl	13273.5	14179	16974	18479.5
Agmat	10890	13329	16425	17644
Agmo	16798	25213.5	14469	25509.5
Ago1	30557.33333	26050.33333	23351.66667	22225
Ago2	18914.25	13372.5	27858.75	22416.75
Ago3	15843.6	19485	16335.6	12301.2
Ago4	16539.2	22180.8	11761	27669
Agpat1	22034.5	26895	22494	26638.5
Agpat2	13668	15873	13201.5	13247
Agpat3	23108.5	21171.5	23594.5	23188
Agpat4	10937.5	15303.5	11606	18496
Agpat5	23267	25381.66667	22856.66667	25709
Agpat9	13221.5	8800	19353	15206
Agps	30496.75	22043.75	24299.25	19433
Agr2	10463	7994	17758	13775
Agr3	22573.5	31317.5	28761	29568
Agrn	23888.66667	17578.66667	18026.66667	33387
Agrp	16548	20146	30866	35041
Agt	6439	6658	12718	15629
Agtpbp1	9999	16389.75	7644.75	8701
Agtr1a	25805.5	16071	35908	19831.5
Agtr1b	38242	10145	12990	10437
Agtr2	31322	32504	38080	32555
Agtrap	13473	12652.5	14521.25	12133
Agxt	21165	22837	21080	21996
Agxt2	38349	733	32060	30587

Ahctf1	23924.66667	25276.66667	21826	24976.33333
Ahcy	10222.33333	10533.33333	21939.66667	22427.33333
Ahcyl1	7392.666667	7662	16135	5295
Ahcyl2	17359.16667	25324.83333	23634.16667	23649.66667
Ahdc1	33900	34250	35726	33844
Ahi1	19054.66667	19180.33333	20221	21890
Ahnak	17880.28571	17188.28571	14284.71429	16535.28571
Ahr	21399	8246	25454	17293
Ahrr	16830	2637	20946	6443
Ahsa1	18343	17044	22422	21535
Ahsa2	14774	15356	14642	16638
Ahsg	37920	3218	39078	3451
Ahsp	36865	29294	29892	36644
AI118078	21909	25599	32382	35065
AI131855	4825	2142	3933	5389
AI181833	24860	26067	28277	36211
AI182371	21958	20450	21786	23514
AI256396	10221	3232	4756	12096
AI314180	16581	17608	13634.8	20565.6
AI314760	10563	1241	9913	10193
AI314831	28750	30184	32006	30483
AI316844	38824	32623	34270	34878
AI317395	2209	39405	32982	31452
AI326223	8259	11392	17412	18796
AI413194	24492	23136	23847	26900
AI413582	3792	6461	4024	3202
AI415730	30098	17719	12653	15502
AI427529	18932	18543	23478	23398
AI428996	31336.5	27369.5	26051.5	23105
AI464131	12584	9824	18364	15864
AI464935	18350	20449	25432	26934
AI467606	35336	23548	25463	21165
AI504432	27122.5	17364	22406.5	34259.5
AI507616	4778	37585	4123	4265
AI508575	15449	18226	16154	21451
AI509003	12382	36227	19044	17824
AI530533	12973	15553	22565	21903
AI530704	14946	16879	14262	13157
AI553296	6095	2961	9175	7801

AI586267	10181	9184	11563	10204
AI593442	30992.66667	32555.33333	34175.66667	23085
AI594692	20434	21800	21179	25283
AI595560	15793	28428	25189	235
AI597479	14406.5	12728.5	16943	19475.5
AI605151	18736	9737	17144	16700
AI607873	20675.5	14373.5	14761.5	15542.5
AI608085	20223	18147	21781	19124
AI614438	12300	4363	13975	7601
AI646519	25047	26283	29195	8599
AI661453	19517	5037	22520	1173
AI662270	18244	23778	20106	23764
AI834762	39053	31767	39439	6010
AI834886	24320	38269	13195	3803
AI835086	37325	39430	9630	11865
AI835779	17235	16601	20766	20088
AI837181	20923	19803	18521	17747
AI837370	18545	5650	17364	15874
AI838397	13706	20303	19899	20357
AI838745	39510	1022	2818	1384
AI839650	10709	14450	18564	17257
AI839651	39561	26642	7206	6672
AI839979	38298	39113	40422	38653
AI840334	4942	16196	23332	21424
AI843588	1394	40732	10944	16592
AI844789	18926	17445	23206	20662
AI846148	14168.66667	2913.333333	29174.66667	14879.66667
AI849053	30722	30056	5150	5115
AI854003.1	19565	22695	21186	23161
AI854246	31382	35157	35246	956
AI854517	28758.5	23750.5	31703.5	29586
AI854703	14477	14122	17205	19918
AI987944	3861.5	21976.5	18720.5	18433
Aicda	35315	38286	39329	481
Aida	14574.66667	15945.66667	15067.66667	17906.33333
Aif1	27426.5	24447	27870	31300
Aif1l	20801	21629	33367	37834
Aifm1	11491.5	13937.5	11462	13937
Aifm2	26207.5	8672	25346	9505

Aifm3	34051	25955	1025	4008
Aig1	22833.5	23064	23488.5	21021
Aim1	4231	38876	3153	38533
Aim1l	32861	3778	2255	13483
Aim2	25869	27371	27674	8206
Aimp1	18575.33333	26511.66667	15257.66667	19705.66667
Aimp2	11652	11241	5797	11002
Aip	9729	13009	11594	12690
Aipl1	16454.75	14029.5	19901.75	20918.5
Aire	18512	19586	22470.5	24118
Airn	14472.25	16233.25	18368.25	19110.25
AJ315977	10082	22526	32114	30800
Ajuba	21328	36777	31600	38712
AK004585	20805	7429.5	21155.5	5155
AK005018	23593	37372	12783	22152
AK005483	9943	8684	6979	7906
AK005803	3741	9009	3061	30358
AK006309	6715	5302	7142	40917
AK006312	1870	32570	34470	36591
AK006573	23668	24726	27125	37332
AK006604	33797	36852	28458	36249
AK006628	774	5048	2109	15677
AK006784	33039	34651	36755	34925
AK006831	25413	28602	37886	28894
AK007090	191	4331	13648	13279
AK007186	12203	2800	15443	16924
AK007258	8119	9278	13363	12177
AK007723	11377	19368	18814	17623
AK007819	2869	40659	36181	27947
AK008541	29078	30305	31970	30611
AK008871	16622	16012	19833	23621
AK009443	8757	4663	6133	2315
AK009963	6816	15049	10033	15431
AK011803	18198	10054	9697	2990
AK011948	8287	1776	40363	38221
AK012034	22059	24909	32266	33442
AK012612	28475	28329	22171	22983
AK012975	39305	30966	468	34235
AK012991	34876	492	37070	1190

AK013113	3259	10932	14583	19290
AK013239	8445	13719	4551	12633
AK013499	18480	16913	24442	24169
AK013505	19777	13339	15375	14246
AK013994	7943	5162	44	39884
AK014007	22481	24865	32342	36704
AK014588	23925	24663	26776	25642
AK014660	1003	2155	18445	14235
AK015074	20084.5	25470	24415	25715.5
AK015077	25780	27227	29029	27440
AK015109	7165	9179	38763	37621
AK015319	33878	28004	30780	36343
AK015367	29959	31464	2844	36846
AK015592	32183	728	36455	37259
AK015669	37535	27025	1632	35996
AK015801	33037	34806	36367	34693
AK015955	29312	30775	32520	30949
AK016058	34737	37981	2225	347
AK016162	7990	11676	14979	15894
AK016228	21510	26010	27765	25785
AK016336	24915	37839	38380	37380
AK016435	5233	32981	874	10956
AK016486	1933	37708	30701	32404
AK016503	5857	2848	12781	8944
AK016549	25365	40208	38370	1809
AK016576	32491	36990	36716	37397
AK016580	6491	9520	19374	23927
AK016596	30621	32259	33969	32205
AK016792	10786	13552	13929	15211
AK016823	30845	32481	34393	32641
AK016887	30780	831	1298	2850
AK017011	25085	26286	28312	27107
AK017085	32671	34273	35551	33704
AK017327	20400	19754	23501	9069.5
AK017409	9204	7453	17429	8767
AK017915	35731	40726	36215	40193
AK019053	11302.5	16395.5	29540	31189.5
AK019581	22617.5	20422.5	31607.5	28723.5
AK019605	23848	29613	2059	38971

AK019700	17832	29364	18392	19906
AK019816	10236	9610	14487	17062
AK019935	3722	15227	12930	20862
AK020721	27364	31128	29133	27941
AK021137.1	24877	139	6271	39713
AK021360	22317	19905	22169	20885
AK027926	26065	39614	28768	27804
AK027952	31019	28913	31701	30190
AK028065	30922	23505	22198	23170
AK028073	11667	10391	1303	38844
AK028094	28044	41045	31543	28985
AK028245	12150	8424	15317	12699
AK028328	3611	24394	38922	33787
AK028390	14430	20520	22589	25632
AK028437	36530	2890	35701	40724
AK028471	31870	22769	23952	22961
AK028473	30370	31503	41015	2513
AK028486	27370	25456	21169	25762
AK028495	31797	38982	35903	2795
AK028511	32842	34465	35585	33744
AK028520	1461	1484	4947	40494
AK028579	31110	36891	377	34184
AK028736	11709	11505	39847	29849
AK028903	6836	9825	13829	13499
AK028934	23966	29730	31275	31965
AK028935	6804	10956	15944	17266
AK028988	27060	28479	29818	28825
AK029063	1681	19903	22159	21724
AK029132	32527	33934	36934	34959
AK029197	21451	6606	787	7653
AK029209	31773	33447	35016	35208
AK029218	23213	24298	26014	34381
AK029223	649	6950	16258	11067
AK029269	27132	28923	28785	27662
AK029272	24997	26339	27695	26711
AK029296	7824	10239	9511	6198
AK029495	32462	33713	35886	33953
AK029515	35408	30042	30154	28824
AK029547	34462	19462	32062	30754

AK029558	997	14934	4987	16317
AK029592	23697	24749	26783	25671
AK029611	30412	36169	38390	32570
AK029646	30309	32647	37819	34675
AK029687	31856	33048	5759	3629
AK029739	24767	25998	277	26424
AK029834	12974	4536	38267	39082
AK029885	32498	34150	39405	34449
AK029910	26501	28236	29053	39883
AK029919	26479	7237	29853	35987
AK029975	15665	16979	20167	20211
AK029983	27178	31040	29472	30329
AK030034	37858	32534	33498	31982
AK030114	28223	38414	31604	30238
AK030150	10770	14266	25587	23350
AK030217	32944	34715	36365	34657
AK030228	29837	26863	29071	27779
AK030229	29907	34194	260	31178
AK030243	29090	30519	6134	30371
AK030263	26081	27595	28757	39632
AK030271	36024	37832	40122	3788
AK030286	25077	35446	27494	26395
AK030373	31315	33215	33699	32155
AK030462	27542	39445	33766	30405
AK030467	6316	9045	10498	13336
AK030494	4543	32454	2388	40939
AK030508	8238	40227	40964	88
AK030526	27981	29436	31050	29587
AK030544	36122	36773	35795	39956
AK030587	25910	27055	29385	28153
AK030614	32951	37278	35614	33781
AK030641	28013	29427	31243	29740
AK030693	28400	29855	31311	30240
AK031009	8808	37269	13043	28119
AK031098	24149	25393	26779	25669
AK031157	998	38186	16626	15033
AK031168	3915	35807	39647	40994
AK031173	26493	27949	29278	28285
AK031195	20177	19907	25301	28303

AK031219	33381	36361	2055	4439
AK031223	4037	36996	3906	7012
AK031258	10023	10795	17226	15474
AK031269	23444	185	26191	25181
AK031320	28248	21262	15138	21723
AK031434	35413	19208	18952	20869
AK031532	39066	30366	38631	39033
AK031552	18200	14366	39153	30613
AK031765	33202	34840	36872	35070
AK031876	10077	9361	9358	9140
AK031889	26257	16523	18765	19802
AK031920	28841	15282	25395	26824
AK032087	24726	32953	28076	26841
AK032119	8821	6595	30232	28873
AK032183	31362	33024	34684	34875
AK032241	28796	4599	32302	2378
AK032269	33064	34821	36785	37533
AK032303	25017	26247	27953	26949
AK032312	29578	34472	33364	31741
AK032313	38011	32753	38333	26825
AK032387	26524	26466	28186	30119
AK032544	7383	11882	18369	17769
AK032574	33253	38915	38234	6081
AK032608	20164	12367	24317	24565
AK032685	32977	34365	37459	36562
AK032764	13430	3542	16504	16356
AK032780	28346	30061	30436	29113
AK032795	31992	32963	34461	32527
AK032893	3742	9024	7080	6945
AK032924	5580	13653	16775	18417
AK032939	24723	26024	27389	26263
AK032954	28311	30959	30532	27685
AK032987	32672	25879	28001	26785
AK033032	37022	21412	22449	19063
AK033120	29169	31077	30658	35305
AK033125	29890	31669	32050	30650
AK033143	32840	34637	36068	34491
AK033185	35007	32999	33163	34190
AK033194	1735	30978	37069	38080

AK033221	28976	30963	6002	553
AK033242	28989	34033	33694	35868
AK033258	4747	2373	9445	7746
AK033266	1007	39204	31321	1613
AK033297	40409	8386	5219	4556
AK033311	7281	37628	8676	11563
AK033312	35708	33925	33663	32080
AK033351	31171	32792	34355	32621
AK033359	40714	39343	5077	10119
AK033367	24505	23529	31078	29475
AK033456	26108	5722	8727	5435
AK033468	8513	10399	18577	16732
AK033492	30978	32894	34392	32649
AK033508	28358	30252	29936	28708
AK033527	33207	34947	36696	34945
AK033546	25831	38057	37107	7151
AK033575	28689	30379	30745	29530
AK033595	9521	1560	5505	6630
AK033617	2375	10939	7241	7610
AK033777	30514	32579	32487	30976
AK033780	2519	32286	37972	28083
AK033817	3824	12506	12383	13942
AK033839	25730	27048	28523	27604
AK033846	28445	29829	32668	33969
AK033876	25748	14802	5298	6048
AK033886	29875	31361	32565	31131
AK033926	7488	10212	10979	9872
AK033971	24017	30443	38282	30972
AK033995	19485	2027	8836	21420
AK034021	14831	5251	8203	8418
AK034047	253	33932	7268	33341
AK034056	32018	33313	36505	34480
AK034116	24874	26000	27811	38250
AK034268	27754	29079	5933	8196
AK034273	40403	2886	13096	12210
AK034281	29779	31287	32906	31322
AK034299	31547	35830	1574	302
AK034316	10953	11095	5115	39173
AK034318	26951	27511	28301	33628

AK034319	39775	2348	4919	4123
AK034352	32667	35054	34718	2372
AK034441	32198	34677	37115	35097
AK034452	34339	35014	2803	1876
AK034470	26836	28516	28643	27668
AK034481	23981	25101	26949	25821
AK034485	26078	27311	29260	28044
AK034486	17623	18401	25747	24599
AK034548	2391	25996	25514	27460
AK034604	23317	24398	26259	25158
AK034606	36904	36802	37531	31275
AK034630	31987	33819	380	33068
AK034668	1391	1744	661	39836
AK034700	32788	19365	18098	19714
AK034716	19673	20052	25326	25614
AK034726	21370	11246	9161	13639
AK034767	29817	38792	28018	115
AK034791	31522	33880	4150	32455
AK034929	13265	9585	4014	40028
AK034950	31743	33585	35046	33622
AK034972	32666	34139	36959	35010
AK034993	31624	33363	34830	33027
AK034994	25677	27137	28198	27187
AK035023	34290	24469	26270	25286
AK035143	23561	18044	24428	26980
AK035148	24021	25153	26933	25986
AK035186	20403	9937	18687	16570
AK035254	37036	16128	19072	11776
AK035359	19190	17957	20205	19746
AK035362	25101	26340	28021	33384
AK035376	32165	35783	35110	33299
AK035382	26452	27583	41079	29071
AK035404	32489	33722	35455	33618
AK035470	7173	1025	31780	12005
AK035482	16537	16351	23370	22469
AK035497	36017	5154	40829	11840
AK035522	27442	31723	30247	28972
AK035567	28672	30045	32119	30243
AK035611	29351	30795	33034	31449

AK035617	28970	30692	37197	30035
AK035621	28839	30329	31819	30372
AK035636	28701	30607	30295	29085
AK035685	40168	6940	31360	40601
AK035694	28031	29418	36921	29402
AK035709	15662	827	5364	14900
AK035714	17584	17154	13782	19875
AK035785	32034	33705	1216	1482
AK035808	32300	34548	35830	32874
AK035829	181	5603	9893	6452
AK035842	24752	26069	27316	26359
AK035983	18729	15496	24440	22732
AK036023	31921	33606	35534	33745
AK036030	24070	25307	38039	25868
AK036031	30606	32507	33552	31865
AK036034	26908	28684	28593	27497
AK036061	30146	25214	5292	4643
AK036074	29741	14013	3786	39247
AK036096	34981	10778	7583	4804
AK036133	36788	35686	16665	19495
AK036142	27471	26050	27266	32864
AK036256	39628	34720	29735	34473
AK036268	24420	26734	33550	34724
AK036287	24301	25558	38397	25576
AK036490	10160	12901	8905	9052
AK036500	31696	33399	34805	33037
AK036506	32983	34769	36783	41097
AK036535	29572	31463	31409	30084
AK036564	29775	20281	32439	31772
AK036601	25995	27277	29030	27858
AK036620	11655	9261	7940	233
AK036634	22221	19546	17805	16951
AK036645	25732	26836	29461	28195
AK036787	19170	17889	30461	23919
AK036850	38751	27220	33984	27904
AK036853	23364	29932	32614	33145
AK036887	29069	30560	31997	30623
AK036949	22741	14394	26443	25342
AK037050	6634	9372	12317	11983

AK037052	31436	40688	29212	30510
AK037080	21238	16302	17173	20863
AK037117	31150	37435	4562	6284
AK037123	29931	31489	33823	32124
AK037125	6699	3781	36327	34714
AK037159	36549	25741	32164	29658
AK037161	22949	14012	17623	14115
AK037171	36016	24922	27642	26151
AK037186	22964	14647	18382	14563
AK037194	30561	32515	33146	31547
AK037270	25786	27109	3736	215
AK037292	14000	16837	23498	22063
AK037312	31292	33298	33574	32038
AK037317	11456	872	3912	34134
AK037327	10515	1839	4331	3953
AK037341	29534	31090	31906	1799
AK037363	817	9830	6074	7805
AK037426	24531	25607	27887	26700
AK037428	30041	27859	31866	29198
AK037439	33066	37775	1710	3540
AK037444	5924	17945	13438	16256
AK037448	32938	34369	37265	35334
AK037460	40679	23960	25196	25030
AK037474	15916	6174	6968	10709
AK037495	29122	30527	35644	31481
AK037503	33921	23037	22089	25304
AK037544	8923	7564	9163	11449
AK037570	34492	26610	28024	26997
AK037579	14311	8164	1549	6055
AK037603	37697	37448	41034	39684
AK037635	15864	15607	14119	40875
AK037636	23145	24229	26126	25048
AK037653	28470	27104	5816	4884
AK037684	27335	25688	31687	29345
AK037715	29335	30637	34407	30789
AK037756	27550	25119	26962	28565
AK037765	10310	38035	39522	39654
AK037788	4887	3077	14434	10796
AK037792	26313	28062	28156	27152

AK037804	2607	39232	37465	39664
AK037823	7758	9449	2564	1587
AK037839	2678	32624	34827	38500
AK037888	17726	20874	15163	13657
AK037912	25431	34676	28854	27593
AK037919	22923	18920	20180	21168
AK037972	12580	17575	16235	16508
AK038022	27765	32913	31592	36361
AK038032	35829	4640	39252	30967
AK038033	36778	29119	29932	41117
AK038075	17984	23471	19032	24182
AK038083	28233	29705	31348	29869
AK038096	19166	8788	17577	19264
AK038114	28606	30283	35474	34596
AK038131	27342	28653	31245	29761
AK038137	29558	31112	632	30679
AK038147	7025	468	39351	8454
AK038190	39137	30749	23148	22782
AK038193	35479	34152	1504	1072
AK038197	4094	7814	10602	11079
AK038230	5920	3806	6985	2918
AK038241	39968	23534	2553	34789
AK038246	15557	1863	7898	4305
AK038312	17735	9183	10200	17329
AK038314	25150	26368	2455	35216
AK038359	201	35664	34041	32907
AK038377	27163	28568	30204	31807
AK038388	1803	41064	32610	22095
AK038400	9083	8032	14433	12910
AK038482	26135	27888	28650	38631
AK038521	32344	33617	40426	35073
AK038550	13833	18432	32368	37427
AK038627	14292	14063	17407	15910
AK038629	6303	6249	16613	17855
AK038647	30793	3580	2673	4020
AK038668	32333	34071	35228	33389
AK038710	5188	36186	39937	40473
AK038711	32922	34707	36261	34616
AK038725	29568	31050	33018	34380

AK038867	3191	11829	3360	9200
AK038886	39649	14748	39686	38208
AK038901	38077	29147	39114	34102
AK038949	38074	25535	30132	35047
AK038954	13089	30411	1556	36118
AK039003	37810	31195	40539	31222
AK039079	32677	34907	35269	35807
AK039091	27805	29549	29316	1601
AK039103	26517	32732	33899	34735
AK039133	28156	35424	31839	702
AK039163	28381	35141	30272	34338
AK039229	7801	17361	3797	6432
AK039234	24511	25680	27531	26444
AK039250	40912	20462	38490	23341
AK039291	22594	19810	30007	18144
AK039334	29446	31410	31292	29854
AK039352	29097	30663	41031	30833
AK039362	32523	34298	36139	34393
AK039370	26923	19432	24003	20739
AK039385	22272	16583	22534	19244
AK039524	17502	22801	17195	21901
AK039527	25191	26326	28630	27404
AK039539	26012	36586	29099	27846
AK039545	29339	30788	33158	31563
AK039580	36004	40791	174	6624
AK039632	34741	33391	37131	9701
AK039641	32043	33380	36424	34401
AK039663	15143	14018	11715	14713
AK039680	30005	31755	32225	30878
AK039684	30405	31982	33505	32034
AK039697	29979	31780	32075	30744
AK039784	36352	31541	31493	24778
AK039803	37488	6579	26361	25358
AK039823	40083	40694	4526	880
AK039830	32361	38454	35869	40460
AK039831	38611	4712	10106	10958
AK039835	23248	24341	26144	25110
AK039925	29792	31759	37713	30621
AK039947	31129	31319	33721	40000

AK039989	33258	34916	37118	36434
AK040015	25324	26775	27627	26644
AK040033	33106	35185	35921	33933
AK040047	27465	25218	33712	32181
AK040144	39951	12045	8340	9321
AK040149	28309	31737	1250	35742
AK040193	26092	18246	17822	18496
AK040273	32101	33387	36091	34097
AK040293	26023	5938	13145	2687
AK040334	809	3636	6320	7156
AK040362	30070	32046	32019	30595
AK040368	21142	39521	38487	6022
AK040392	15030	13281	21740	22695
AK040417	38069	34750	37640	75
AK040434	21028	40737	17302	4506
AK040443	35308	34971	36058	33958
AK040490	20645	24912	28605	25330
AK040515	33196	34884	36747	34971
AK040557	29083	30585	33244	31516
AK040617	6332	1514	4892	8672
AK040636	28294	30080	30365	37005
AK040653	30466	31967	33558	31837
AK040669	35467	4107	8021	34907
AK040676	12421	13652	6965	11192
AK040677	11121	12311	12318	11184
AK040742	27176	28949	28767	27709
AK040747	27136	28547	39792	28733
AK040755	27922	29782	29618	28360
AK040776	615	34578	38826	36024
AK040797	24373	25458	27688	26514
AK040808	26120	27752	28232	27256
AK040811	37232	26482	9720	41107
AK040820	20924	7298	9576	6451
AK040829	26239	27386	29867	28572
AK040858	22844	6498	8731	10994
AK040918	32504	35955	36224	37679
AK040981	24953	26228	27874	26857
AK041006	25277	26361	28797	27590
AK041152	31730	33431	35089	36385

AK041215	33285	34963	36948	35143
AK041221	22240	17128	24895	23695
AK041294	30706	32296	34229	32533
AK041295	28538	30383	29863	38095
AK041319	4333	36034	38000	3106
AK041339	37056	25927	27942	26766
AK041360	2335	10041	8229	11820
AK041448	32992	34474	37274	35355
AK041455	28427	29908	31467	30012
AK041466	7170	39745	4441	40594
AK041471	38474	13457	21579	22971
AK041484	37636	39415	10569	4107
AK041510	28076	21469	21223	17292
AK041515	28629	30108	31602	30473
AK041531	38619	39210	8573	11487
AK041553	24733	25894	27882	26719
AK041569	25790	18173	9313	16835
AK041575	804	3727	31144	2991
AK041585	32557	34920	35252	33293
AK041645	2037	7528	9016	5350
AK041701	1216	36083	37290	39392
AK041705	30549	32146	33705	32153
AK041794	39218	27489	6355	27459
AK041795	11603	13717	12462	26620
AK041827	24460	25635	27409	26266
AK041855	20688	17803	33728	19974
AK041859	12069	14821	17495	16447
AK041861	39870	37719	2199	27258
AK041868	25926	27408	13366	26927
AK041974	7663	15133	16422	17163
AK041984	31791	33535	34903	33126
AK042092	9026	31390	27107	30402
AK042108	8225	29269	231	38101
AK042125	25182	26672	27610	31587
AK042162	12642	8335	6773	9092
AK042218	14701	11506	22138	17490
AK042300	25411	26682	28443	27461
AK042306	37319	30967	32076	30720
AK042349	32162	37328	35723	34023

AK042395	31737	39332	13406	16245
AK042403	33424	30875	33350	35233
AK042441	36340	10056	24549	23892
AK042451	29025	31119	31751	31893
AK042495	23283	24342	26267	25224
AK042535	33919	31511	39305	37228
AK042543	21559	28131	37301	40734
AK042550	9017	5759	5273	8744
AK042563	29364	30884	32429	36200
AK042564	30709	32389	38976	1197
AK042566	1448	12889	17662	17872
AK042572	28197	35957	33282	40420
AK042580	27919	29396	30863	29523
AK042586	26163	328	40778	36725
AK042592	33019	34786	37147	4504
AK042611	25099	26341	28070	27130
AK042631	30650	32196	33768	32110
AK042637	29236	31101	30993	29724
AK042648	5031	6570	8556	15758
AK042680	18738	18701	21928	22210
AK042687	8152	35388	30402	28969
AK042740	26505	28178	33981	27367
AK042768	39172	34768	35122	36417
AK042793	26272	28035	28163	27054
AK042797	25364	27763	28447	27399
AK042806	29540	31037	32597	31039
AK042825	25093	29673	28503	27339
AK042859	31033	32707	34353	33023
AK042886	29344	31139	31560	35472
AK042908	28034	29444	31389	29878
AK042909	39383	33881	6378	3352
AK042938	24365	25522	27307	26362
AK042944	40453	3579	31644	10853
AK042984	12537	7979	5692	11829
AK043060	31815	33021	36601	34550
AK043066	5306	24919	26753	30939
AK043105	23861	24957	26742	25793
AK043184	26594	27984	29623	28461
AK043237	24079	25116	27938	37870

AK043254	5202	10333	37055	40207
AK043256	33645	33361	2718	32997
AK043289	34813	32758	34299	32937
AK043296	28687	40866	2155	39471
AK043317	24252	25386	27277	26220
AK043364	38502	7389	27465	1565
AK043378	29156	33389	32857	895
AK043400	41082	32302	32414	26203
AK043462	33168	33382	34860	33069
AK043488	10402	10737	13314	15965
AK043520	32140	33981	35686	34060
AK043527	31174	32548	9596	1330
AK043537	24667	26158	33537	28460
AK043575	10724	13064	16853	16822
AK043581	904	32889	35191	33212
AK043627	26200	27902	27893	35030
AK043660	26612	2843	9209	28876
AK043715	25942	27657	33873	35084
AK043717	13242	13910	15822	21233
AK043736.1	27594	28826	30909	29557
AK043788	30019	31538	33174	31573
AK043797	38196	31742	41105	40326
AK043869	38708	41122	13578	13779
AK043872	22502	27874	31888	40951
AK043935	25312	26475	28690	27455
AK043936	36891	12764	9400	17365
AK044003	30095	34203	31066	38569
AK044019	36761	23750	12381	10591
AK044083	34773	32661	33071	31614
AK044089	29712	2154	31886	37684
AK044147	33955	39637	887	320
AK044165	25929	2616	1441	505
AK044196	3509	1817	7363	10236
AK044198	24089	40340	27186	26036
AK044315	26282	27407	1769	29659
AK044317	31612	26058	998	36814
AK044348	33102	37617	30943	36440
AK044363	18749	20379	24898	24535
AK044390	352	3477	12603	10478

AK044473	31669	33296	35130	35404
AK044491	27621	35273	39765	545
AK044498	1069	37142	13115	14256
AK044500	28715	30705	30361	29032
AK044509	6875	9093	16045	16113
AK044542	40140	5584	14900	21053
AK044628	33158	7872	18038	18433
AK044646	33565	31941	33325	32194
AK044650	25657	26909	29143	27495
AK044680	29861	26582	172	6376
AK044681	25472	26616	28813	27629
AK044722	5511	6528	8309	8088
AK044799	14656	6314	9808	2387
AK044873	39918	19414	18937	18224
AK044886	27907	27827	29042	27880
AK044951	3973	33062	28331	37003
AK044965	30033	31771	32258	30903
AK045016	479	9411	30330	39872
AK045039	30316	31818	34140	37132
AK045054	24602	40512	601	1667
AK045058	39106	4953	9433	37314
AK045080	36555	28392	32023	38400
AK045171	17127	16971	23155	15922
AK045243	1666	7381	37468	32261
AK045265	25924	27288	28803	27713
AK045275.1	30732	32391	33822	32550
AK045293	28668	30226	31187	29833
AK045313	32214	8204	36270	33239
AK045332	18329	10754	16792	15719
AK045381	31418	32748	19139	12967
AK045389	23636	24671	31665	6395
AK045416	35158	18380	25183	20018
AK045458	14015	13857	15364	17749
AK045463	3884	40958	35866	4302
AK045464	36637	29676	32549	35409
AK045486	7700	6187	7632	8515
AK045559	6078	35703	37399	38441
AK045569	27007	28313	30720	29263
AK045593	39117	40779	2569	38654

AK045595	34238	38096	37926	39039
AK045702	39276	31448	35372	31578
AK045766	26432	27515	30535	29146
AK045769	8506	35068	30407	41
AK045779	35200	29956	28354	27374
AK045794	23366	6743	30814	38875
AK045817	39828	20008	8382	8966
AK045826	25188	26431	28005	3087
AK045835	29172	30577	33112	31505
AK045845	13349	3776	13461	5673
AK045855	32294	34567	34635	32890
AK045858	39652	11058	1174	38732
AK045928	20757	28736	33744	30673
AK045961	15624	15802	22794	23000
AK046013	24584	26073	27537	26575
AK046042	23752	38800	37328	39517
AK046053	23818	21757	38255	34216
AK046060	25772	26958	29360	28052
AK046151	4539	33508	34084	32493
AK046168	28515	37662	37059	38602
AK046200	27363	29151	28958	27865
AK046202	26284	28055	28164	27061
AK046212	32259	327	35467	33676
AK046216	29684	36800	34142	39689
AK046241	26956	28480	29327	28114
AK046257	40378	22581	35186	33381
AK046285	30573	32181	33560	32407
AK046310	31811	33611	36471	7917
AK046342	32302	34844	39837	37197
AK046345	34524	25572	27463	26417
AK046358	40217	36603	35491	34859
AK046412	1219	30842	2258	353
AK046413	105	40379	37213	3725
AK046423	36612	33883	39464	31854
AK046453	12212	5893	39525	10432
AK046563	33326	28005	34790	36646
AK046564.1	36053	4818	14389	11348
AK046642	10231	5300	11279	8389
AK046687	11203	15215	12839	13916

AK046703	30481	11630	35406	4519
AK046735	8573	4699	2215	39755
AK046742	14767	3714	16008	31254
AK046743	25604	27158	27743	26746
AK046770	24054	25265	26735	25760
AK046810	25582	27772	2537	39016
AK046815	24090	25391	27348	26195
AK046827	29116	31291	31881	31239
AK046855	24524	33	27332	26390
AK046873	10250	13007	11812	15190
AK046926	13327	13246	20610	14355
AK046930	15374	1173	7661	1841
AK046946	31024	32646	34201	32503
AK046982	24951	26121	28181	26974
AK046987	26919	38363	28459	27416
AK047007	29283	31183	31015	29711
AK047032	28795	29272	35220	26159
AK047056	1267	6741	7055	7022
AK047065	25455	26799	28675	27418
AK047066	25761	27353	2955	37229
AK047097	32456	34396	10730	34423
AK047137	36774	37925	38526	36733
AK047151	25812	35782	37415	35474
AK047176	31725	33505	34956	33160
AK047181	32357	33993	36200	34347
AK047189	28570	30353	30626	29274
AK047195	32785	34479	35493	33686
AK047235	37471	23535	20235	15695
AK047244	28237	20099	32051	31571
AK047247	12982	29854	11523	33410
AK047305	35711	37577	18287	14558
AK047328	30677	32755	5527	31004
AK047335	30851	39584	33852	32189
AK047343	37491	26646	23860	28798
AK047382	32892	12392	40260	35436
AK047392	28981	30540	4281	32270
AK047395	28838	30239	32371	30806
AK047444	7412	34776	5442	8874
AK047450	24839	25946	30848	27179

AK047453	1777	31331	38808	2026
AK047457	32980	34376	37604	38863
AK047461	32608	2156	38765	4436
AK047488	29762	35235	31898	30594
AK047500	30713	1317	33635	34354
AK047526	23727	5315	27205	26048
AK047560	28961	30466	31846	30635
AK047573	41056	947	2308	40214
AK047642	29647	31148	32244	30793
AK047645	32113	33770	36174	37076
AK047679	30530	35462	33086	31491
AK047739	21313	18433	21377	17489
AK047753	30218	31669	3351	34722
AK047771	40953	30060	31111	40020
AK047779	32281	33531	36996	34992
AK047809	30595	40777	33629	434
AK047842	3447	40499	2548	3247
AK047907.1	18024	22182	23280	32749
AK047920	15105	16023	31377	35740
AK047934	34175	33270	36986	7767
AK047954	38884	973	9790	2274
AK047970	26713	28461	28366	27328
AK048085	37839	29395	37813	25365
AK048091	26863	28067	30192	28917
AK048095	9849	8048	9059	8600
AK048105	18477	34692	20714	22338
AK048129	7961	9684	9519	8739
AK048157	9067	10486	13001	11084
AK048206	16896	16742	24715	18052
AK048224	32658	34419	36279	34524
AK048285	30440	31979	33471	32206
AK048318	32391	33838	35796	33879
AK048351	27269	6922	1827	1187
AK048370	23258	32261	26117	25070
AK048465	28521	30455	30122	29303
AK048476	23717	24799	26681	25559
AK048479	28224	29662	31445	29935
AK048541	37891	21093	37000	342
AK048549	25778	28773	29256	34389

AK048554	28414	30196	30373	29042
AK048558	31699	27089	34838	27442
AK048563	35252	25275	26827	39171
AK048568	32189	34004	5057	34228
AK048585	29569	31082	32493	31371
AK048602	26253	26365	27588	34899
AK048620	38800	31590	26032	36726
AK048677	23623	20779	21455	23258
AK048779	21155	30266	25757	27212
AK048812	4478	6780	17642	19009
AK048842	29771	28371	28689	27536
AK048849	36735	32872	37155	35095
AK048856	33427	25156	4346	11364
AK048858	38367	35292	37753	35269
AK048863	30437	32020	34146	32429
AK048889	26652	27814	30842	29388
AK048897	7227	1667	11564	22724
AK048928	4911	8577	29331	28122
AK048939	5164	2382	27238	26306
AK049026	25770	27131	28452	27312
AK049042	33405	26168	3804	382
AK049068	13064	6282	8314	9484
AK049403	1927	36133	3150	1741
AK049441	35661	38510	28872	34980
AK049577	16125	9654	8900	15345
AK049591	32501	18630	12852	14946
AK049610	23221	24319	26113	25062
AK049654	30602	32688	32869	31294
AK049727	32965	41114	27963	26820
AK049746	5712	10775	10365	16052
AK049858	28671	30547	733	28792
AK049921	25281	24376	26639	33295
AK049947	9255	7730	9043	8536
AK049961	15691	12894	16931	15856
AK050073	33338	29455	1793	39591
AK050117	11174	7706	14636	4518
AK050119	26346	27794	29156	28140
AK050149	39862	20490	37196	3107
AK050258	5032	6939	39074	5773

AK050275	7530	6691	9000	5134
AK050325	25331	21408	26694	26976
AK050466	24595	25722	27912	26697
AK050562	26921	28211	34187	29048
AK050631	2422	5517	15929	12823
AK050745	33185	34815	37029	35195
AK050786	21206	20843	16245	21325
AK050809	30432	32171	33483	31835
AK050816	30334	9766	34220	32473
AK050863	20370	6985	10464	7093
AK050866	26529	27742	30528	29108
AK050867	7978	9376	4355	3285
AK050890	894	2778	1691	9887
AK050935	29994	31519	33104	31502
AK050941	16569	21927	22078	25862
AK050947	31172	32681	33869	32325
AK050976	26640	27798	30753	35068
AK050977	13968	17649	15064	17943
AK050994	9534	9285	18048	11846
AK051046	19942	12633	13465	14695
AK051067	6165	35726	40535	29777
AK051069	40166	31442	39680	40382
AK051074	33116	34630	37392	36462
AK051118	693	26653	28265	31033
AK051244	35819	33815	36876	449
AK051319	21635	10759	12582	13049
AK051337	30445	35406	33385	33000
AK051347	26960	27988	36141	29603
AK051356	25016	26231	27992	27051
AK051391	12494	36908	34233	3537
AK051402	28235	29758	31196	29900
AK051417	25853	27521	27821	26732
AK051484	14525	35401	39563	3418
AK051498	23292	22676	26919	33717
AK051538	3799	1614	270	39588
AK051563	27629	29285	29855	33794
AK051616	29106	30638	9666	35638
AK051631	186	32520	2876	12816
AK051712	16852	15346	7622	4640

AK051757	30986	32631	33615	4560
AK051759	22807	710	20182	17218
AK051782	40132	30331	32214	30658
AK051785	38161	1840	27830	26826
AK051825	344	38572	4328	2379
AK051847	31682	41123	38191	908
AK051888	26055	27718	28212	27069
AK051986	11397	10306	17492	17601
AK052019	24974	26238	27955	26917
AK052021	40644	17371	17149	14621
AK052044	26336	29804	28299	27169
AK052093	20308	20138	27183	26755
AK052097	27174	30729	32300	25811
AK052115	23629	11508	11972	12118
AK052163	11548	6891	9280	10544
AK052254	30144	31741	38273	31798
AK052259	36571	31156	32654	31265
AK052496	38526	18962	22871	22049
AK052554	26054	27190	29930	28596
AK052646	32874	34387	35587	33753
AK052688	18129	34200	315	41098
AK052728	16542	25449	37385	25636
AK052874	10209	38099	40203	9873
AK052881	25779	25906	27616	26670
AK052896	28023	29516	31287	35745
AK052909	493	33709	7776	35706
AK052923	34867	28210	28575	36035
AK052949	38949	36370	2056	2064
AK053006	28450	29957	31340	30234
AK053049	25860	27468	27915	26915
AK053079	32153	33576	36232	36342
AK053200	30579	32518	33238	31621
AK053244	30605	32250	33980	32220
AK053247	25371	26624	28441	27266
AK053272	9465	40864	33511	32008
AK053286	29177	30499	31913	30571
AK053290	28306	3820	16896	5168
AK053302	29117	33227	30867	29529
AK053304	30244	32007	32629	31194

AK053455	34987	31165	32054	35571
AK053467	35419	29963	33942	25393
AK053534	31742	3088	5754	8332
AK053585	25936	27582	28470	37237
AK053595	1039	32288	3946	39855
AK053597	14827	40693	9314	13224
AK053705	30841	36221	33126	34581
AK053721	11759	16355	19282	21805
AK053748	17443	13774	16402	16211
AK053770	2053	33563	9452	33434
AK053777	4545	25655	27562	26591
AK053810	32687	30532	26073	20159
AK053928	32436	33541	1184	4666
AK053931	10175	28778	37178	37947
AK053947	29509	34390	33143	30114
AK053952	24866	10454	7028	7066
AK053967	37421	35796	40468	38368
AK053990	32445	34030	35538	33677
AK054078	32224	34173	34770	33057
AK054094	26037	27398	29052	28037
AK054100	32853	34471	36778	34867
AK054126	25255	26750	27354	28796
AK054176	34738	34163	35602	33750
AK054187	18140	4143	13722	16849
AK054189	2025	1415	15903	10061
AK054208	40000	37610	32391	2420
AK054216	30616	32260	33543	7183
AK054225	13034	10288	11250	8185
AK054282	27852	29161	36303	29451
AK054316	123	391	12476	4642
AK054376	31154	40752	37064	39821
AK054384	29785	31046	32998	31471
AK054427	15499	19846	14052	23122
AK054491	20093	20253	19397	18840
AK054507	21915	22627	18885	20389
AK075627	29855	31932	31672	30210
AK075639	30363	32156	32882	31362
AK076168	26309	27863	41065	27246
AK076276	6532	7848	34024	29828

AK076366	26356	27480	33362	29088
AK076452	5471	8677	20175	9681
AK076480	37751	27478	32044	1081
AK076547	29887	31900	31758	30308
AK076552	27018	28530	37199	38238
AK076567	7887	1628	38707	1497
AK076568	13768	17558	17464	15792
AK076573	30785	32846	33433	31744
AK076582	25773	27441	28178	35531
AK076665	35163	28911	30616	32259
AK076685	33197	34909	37210	2382
AK076715	32927	34901	36667	34453
AK076723	25526	2041	29353	513
AK076741	36818	9352	30791	35325
AK076794	23616	24755	26293	25306
AK076845	30345	27933	31320	29752
AK076852	31666	34102	35022	34716
AK076876	5186	3217	1980	1521
AK076884	11763	1906	18023	18666
AK076901	23711	24751	26886	25734
AK076906	24598	25880	27272	26164
AK076918	37815	40863	29614	28265
AK076935	3243	37105	12917	7122
AK076949	9554	2433	8120	15280
AK076950	28786	32718	30507	29333
AK076966	32815	34494	35500	33697
AK076973	16276	15645	18561	12242
AK077006	22195	18629	19925	27306
AK077175	31621	33292	35707	12529
AK077207	38220	37972	27439	26454
AK077230	31686	39032	12419	209
AK077237	17663	12208	17390	19294
AK077428	18372	14668	15162	13999
AK077526	31895	33115	36258	34247
AK077687	5131	5824	5305	4811
AK077805	26980	40941	6058	8479
AK077809	27452	28936	34381	2612
AK077945	25193	26607	40942	26299
AK077974	19196	3333	15411	6846

AK078008	24989	30027	28670	35402
AK078010	24102	39317	6303	12314
AK078072	29510	31068	32499	31137
AK078141	29777	993	37343	31364
AK078177	36769	29577	13793	39509
AK078234	15812	14126	12470	15755
AK078235	39429	26072	27239	26280
AK078238	30819	32409	33918	32274
AK078248	28991	30452	31956	30493
AK078295	33044	35134	36277	34175
AK078302	5513	10564	13887	18072
AK078356	17772	11118	11487	12622
AK078400	231	37571	27063	25920
AK078423	10270	34215	37008	436
AK078447	28364	29861	31283	30078
AK078537	33884	29578	35754	38589
AK078574	28359	29847	997	29654
AK078618	33701	36951	417	40247
AK078638	32345	33853	671	12116
AK078656	25694	38555	40538	9093
AK078757	37573	26656	23602	24074
AK078885	14530	9711	11286	8548
AK079007	11792	11006	9158	7108
AK079050	13273	2540	36810	4505
AK079054	30814	33004	34468	31301
AK079064	36726	11974	8759	5173
AK079094	15026	11105	11826	13628
AK079114	25301	26039	870	5790
AK079151	36125	1454	40321	39510
AK079154	11983	9396	8630	7864
AK079158	38400	32025	32443	31038
AK079181	11543	17475	22122	21368
AK079190	26851	28273	30942	1646
AK079230	17278	8982	5909	609
AK079237	30046	32366	6939	6479
AK079279	3883	36661	10439	7290
AK079391	13689	8908	26934	29177
AK079403	7521	7275	18498	14380
AK079467	14457	12554	21096	16630

AK079482	1672	12256	4966	7648
AK079491	34726	29681	31178	29861
AK079555	29734	31034	32826	31284
AK079562	19864	9055	12028	5067
AK079617	6157	29363	18946	2970
AK079630	12420	29923	7248	39774
AK079697	6648	27869	37428	32656
AK079698	17396	19818	2101	13144
AK079714	38595	32910	32664	31189
AK079717	29199	30737	32103	30824
AK079740	5401	25165	2859	25409
AK079750	31066	32679	34033	35976
AK079758	34851	28077	33857	11057
AK079788	31683	33168	35290	39253
AK079795	910	29526	40519	2938
AK079798	2287	36648	2495	39319
AK079828	20875	14211	11697	13160
AK079840	30300	39692	3207	7084
AK079851	23519	30267	1785	25783
AK079943	39092	2325	31115	1246
AK079958	23922	25036	1217	262
AK079984	31319	32950	34428	32662
AK080082	8195	9677	17208	18066
AK080112	23418	24466	26559	25425
AK080115	25784	3125	30510	29123
AK080127	27234	28512	31267	29782
AK080168	5779	6910	14806	14648
AK080247	29416	30883	32893	31318
AK080263	34049	28694	471	7068
AK080271	24995	26300	27252	3166
AK080390	9419	7611	14438	9160
AK080398	36518	923	33157	1698
AK080404	30415	32023	33749	41044
AK080443	31769	28683	28698	28283
AK080444	5379	35026	37306	34768
AK080458	23640	35391	26893	25844
AK080474	6515	14966	15876	16099
AK080494	29056	30245	31986	30622
AK080558	18015	9299	16952	17387

AK080565	40262	4758	30525	433
AK080570	31602	33241	34910	33114
AK080577	35784	29275	29028	36561
AK080632	35302	17861	18821	17900
AK080633	28766	30646	30385	29218
AK080652	35523	31739	31521	30070
AK080653	26513	27834	29990	28602
AK080659	40026	26115	27721	26569
AK080684	29217	30667	32467	32792
AK080694	34332	942	29710	35605
AK080742	1033	24331	33836	34040
AK080816.1	28111	10011	30740	29363
AK080851	29180	30436	32018	30662
AK080931	32587	34104	36771	34810
AK080994	3772	28148	31627	29990
AK080995	6905	5218	6483	10689
AK080997	12121	8898	19235	14543
AK081043	32470	33677	7307	35333
AK081044	18571	20548	39174	39416
AK081065	27817	36299	30056	39220
AK081083	39976	23949	25386	24119
AK081101	24179	25298	27111	26182
AK081106	24074	25117	27317	26170
AK081111	27233	28952	29117	31562
AK081141	19131	21761	19738	26174
AK081163	5318	10477	22223	13419
AK081170	25656	26955	28549	27667
AK081189	32660	34064	37058	35787
AK081239	29554	31144	33524	31761
AK081257	29192	30617	33211	31579
AK081265	31380	33134	34624	33988
AK081277	27455	29150	35019	29207
AK081302	1274	1291	2166	2814
AK081391	28033	25451	27973	31075
AK081414	24277	25424	4814	1769
AK081464	24641	22570	22105	23467
AK081475	26212	36574	1231	8187
AK081614	26371	6087	25704	27642
AK081617	40823	4267	29400	539

AK081632	31780	34099	34124	32475
AK081644	32073	33208	35813	37479
AK081649	25749	27213	28263	27252
AK081660	32338	33388	35731	33820
AK081687	22749	35198	39953	2879
AK081713	18679	20156	35434	30645
AK081747	24649	26006	27141	26041
AK081791	30450	32404	32833	31297
AK081795	27708	29511	29319	28220
AK081910	27284	18112	23251	25664
AK081935	31807	33450	35140	33303
AK081945	30998	32702	34375	35916
AK081948	4787	5944	34645	39439
AK081997	33137	28693	37449	56
AK082068	35438	38811	242	38701
AK082070	8645	11185	12768	15107
AK082087	36652	34025	36416	1919
AK082143	23838	27287	33319	1372
AK082155	37565	38071	2504	30075
AK082172	33133	34888	36546	34822
AK082198	24012	25267	26553	25601
AK082248	27321	28729	40558	41119
AK082290	27601	28932	31939	36958
AK082307	32533	3144	1407	13768
AK082308	30951	2402	2339	33335
AK082314	30820	40665	9709	6530
AK082363	20728	9777	15575	14845
AK082378	27921	29249	30880	29549
AK082409	40702	1945	40655	2885
AK082412	32931	34475	35660	33825
AK082415	23932	25061	26838	25699
AK082417	29084	30472	32955	31368
AK082423	26748	28155	31738	32944
AK082430	31149	32808	34582	32809
AK082454	12148	1388	2141	39193
AK082531	20905	40514	14557	40878
AK082585	25132	26384	28154	37104
AK082620	26270	27700	37698	28405
AK082626	9208	12509	26640	25605

AK082628	28813	5469	32208	30659
AK082634	27266	28710	29860	28595
AK082644	9020	38514	1801	31864
AK082671	27209	28532	31052	31484
AK082760	27447	33798	34167	29676
AK082805	25799	27308	28109	27115
AK082830	3469	32643	259	37448
AK082951	25097	13681	13156	6760
AK082956	4516	1	3225	277
AK083076	10916	6616	25936	33052
AK083098	26792	28226	29515	28527
AK083150	29181	30708	32005	30969
AK083154	27168	34057	30110	32425
AK083162	20242	15461	18831	17658
AK083203	9274	10233	27910	26923
AK083204	25658	27013	29287	27268
AK083219	30636	32617	32702	31224
AK083252	6884	648	6139	2868
AK083277	14364	5404	11424	10311
AK083355	9094	38288	2375	41090
AK083439	17252	29726	30709	37165
AK083469	26496	27941	29496	32338
AK083492	29928	39601	34936	4246
AK083493	34214	33654	36120	35779
AK083530	31233	33403	34254	32413
AK083569	6015	6540	10566	5014
AK083607	34380	4619	36937	7464
AK083633	31678	33398	1039	32688
AK083637	12097	13974	12811	15112
AK083642	20981	25832	19058	34664
AK083648	35039	38134	6305	4754
AK083657	26893	28434	29162	29806
AK083701	2423	23409	13392	36063
AK083715	31587	37978	28113	38219
AK083738	39029	3823	1701	2027
AK083758	32215	5155	35609	33728
AK083933	21441	9590	8374	15540
AK083952	28984	30419	32049	30524
AK083958	25347	37130	37778	25509

AK083995	31827	40318	40288	3257
AK084036	27929	39651	39945	30509
AK084043	40815	9752	40850	2668
AK084089	25171	26620	27775	31661
AK084126	12080	34270	3751	6786
AK084146	20883	6025	8077	11510
AK084166	16353	7418	5488	13890
AK084172	21427	35914	2287	3724
AK084210	36153	27706	29138	28197
AK084228	35250	15918	17104	6611
AK084265	22379	20682	12158	13047
AK084281	37402	34852	34522	32762
AK084367	18069	19317	22643	22364
AK084369	29952	32031	31767	30307
AK084416	26840	28276	39443	28362
AK084422	9529	2781	108	34529
AK084511	32017	34164	34490	32788
AK084568	17529	5347	23978	27394
AK084583	32831	37389	1836	33828
AK084599	23865	25069	27082	39769
AK084624	33169	30694	27448	26474
AK084656	38902	10576	280	39128
AK084662	25475	26838	1160	36471
AK084674	26917	28490	29333	28177
AK084694	24325	25486	36868	25991
AK084807	3240	37543	4226	6877
AK084859	39159	2122	40033	41018
AK084865	30186	32152	32205	30746
AK084883	30030	31686	33139	5787
AK084963	9289	3438	5731	9207
AK084999	26168	27573	28989	27963
AK085018	26290	27428	30245	28895
AK085025	24785	25696	27930	26738
AK085058	26379	27766	29549	28223
AK085074	25889	27554	28469	28644
AK085088	27333	28784	31268	6994
AK085108	33423	38145	34395	1080
AK085146	5798	40253	33512	30429
AK085216	12452	29961	39795	1099

AK085247	5656	36753	9335	16104
AK085256	37783	32882	32466	35830
AK085261	29167	31113	30857	29531
AK085301	32235	33415	35415	38489
AK085302	33228	29488	2049	39374
AK085317	30288	32218	32432	30952
AK085341	30994	35559	36110	38931
AK085452	25310	29403	30935	1770
AK085462	39533	23412	31968	20296
AK085621	26661	35329	29178	34498
AK085631	12055	3327	4509	35677
AK085636	27745	30279	30714	29312
AK085649	25352	26575	28501	27299
AK085706	33692	13397	17032	13978
AK085715	25315	26639	28341	27232
AK085763	34908	25602	5255	4837
AK085783	2348	37881	40915	514
AK085785	12254	15353	18414	19878
AK085791	25080	26338	8943	4226
AK085795	1432	4844	17216	17151
AK085800	26014	27416	29076	31143
AK085825	16240	21252	19858	26407
AK085855	28908	30391	38431	30590
AK085862	31375	33129	34607	33255
AK085872	27446	29243	39322	27722
AK085875	7163	3857	33588	32028
AK085877	26425	27904	29035	27868
AK085879	28906	30494	38015	7607
AK085881	27030	30451	30032	28022
AK085942	3698	38089	31539	40123
AK085949	35035	39417	5167	3532
AK085953	32630	34485	36497	33
AK085960	40537	33445	39704	33562
AK085999	32598	34244	35447	33610
AK086005	40787	38430	14963	10608
AK086046	32368	33831	35492	40026
AK086048	28596	30541	30283	28991
AK086066	6825	20848	7118	16692
AK086070	23161	24246	8844	5399

AK086095	5366	32844	33053	32671
AK086110	24958	26416	27216	26226
AK086147	25460	38741	29247	37309
AK086157	25095	26194	28642	27437
AK086234	25239	26751	28107	40898
AK086238	9664	34450	10671	6355
AK086255	30979	32648	34272	32680
AK086261	24185	28380	12607	14464
AK086288	3503	4012	33035	31489
AK086336	40479	11483	16099	17455
AK086345	4555	39708	16520	10803
AK086383	24545	18963	18367	20035
AK086393	23434	24535	2939	9809
AK086399	31954	33536	1774	5988
AK086403	29370	30917	32348	30946
AK086409	30871	32458	33581	40801
AK086414	23157	24228	26230	25248
AK086422	29385	32883	29616	30017
AK086452	9803	31184	7254	374
AK086484	31997	33589	35172	33278
AK086485	28055	25731	28124	26911
AK086486	40822	4991	33142	31634
AK086521	27322	28517	30724	29405
AK086525	27065	28311	31325	29832
AK086535	28543	29929	32340	30783
AK086600	23267	24326	26311	25207
AK086608	32744	34555	37010	1408
AK086629	29652	31346	37857	30837
AK086683	4004	2549	15738	15507
AK086706	33050	34826	36410	34709
AK086739	23787	37889	1490	27523
AK086749	16765	39867	30559	29083
AK086813	34561	38575	13152	11612
AK086858	31701	34248	34219	32462
AK086860	25353	26891	27549	26423
AK086877	9339	37200	7765	5259
AK086882	29476	30979	32539	30984
AK086893	36828	39656	4864	32837
AK086916	21271	15073	15264	13493

AK086961	10145	36575	1077	670
AK086969	36428	17902	21315	16105
AK086995	14261	15716	18804	21797
AK087072	39488	39747	1604	40899
AK087084	33772	19577	21570	18037
AK087086	31514	1367	20378	14260
AK087135	22053	14069	14949	15456
AK087240	28695	2002	38145	34903
AK087286	32186	33744	35081	33266
AK087306	10695	8572	21912	16199
AK087351	40711	29108	31809	30297
AK087374	30504	31966	33556	31841
AK087404	33276	26148	27383	26261
AK087409	26334	27333	31060	29062
AK087420	38888	39548	4912	839
AK087429	17787	18266	2122	11986
AK087549	5860	30218	33702	28286
AK087578	27463	28915	30187	29262
AK087609	27547	30159	33316	29800
AK087619	24395	25677	26891	26694
AK087625	40106	3653	29876	28661
AK087639	15436	19782	15958	18568
AK087644	6128	34658	35338	33508
AK087671	2565	3337	6152	6230
AK087742	26769	28187	29374	28161
AK087779	6199	13610	17905	18799
AK087857	39484	8302	3333	12342
AK087862	38952	30920	30806	29457
AK088083	35378	33656	38176	35111
AK088106	6518	26181	36351	28412
AK088163	24588	25947	40066	25693
AK088509	17332	10563	12106	6578
AK088611	13011	41093	2156	6511
AK088623	38153	35317	40571	37711
AK088687	37149	28854	37726	41124
AK088797	20263	3439	13383	12432
AK089089	28741	30439	38149	30005
AK089147	5018	9916	14470	12775
AK089203	25742	27063	28748	31086

AK089204	23763	24800	31656	1583
AK089219	18630	18831	26044	25904
AK089240	2312	34050	39395	36015
AK089277	30473	40453	40933	39575
AK089297	7107	38683	3367	40910
AK089315	3106	3434	4641	5663
AK089361	1936	27314	3528	1612
AK089428	30016	1040	33340	31748
AK089447	11241	6534	9866	9290
AK089448	37280	31495	26926	33074
AK089456	11768	40877	10082	2477
AK089475	31277	40059	34466	34062
AK089482	20337	19684	24073	22859
AK089511	23313	24389	26991	25204
AK089527	26032	38459	34358	1151
AK089562	32164	33827	35949	34121
AK089572	32227	34829	40887	6083
AK089586	12231	5983	9323	4803
AK089588	23709	24883	34439	28093
AK089591	23642	24797	26289	25241
AK089595	3235	38523	6524	40605
AK089606	2604	39877	31663	30252
AK089607	23568	24629	26565	25529
AK089623	32926	34597	36300	4236
AK089631	36285	2083	13457	13237
AK089634	32435	76	15319	15726
AK089636	13025	12427	17539	25160
AK089643	35907	34592	6490	2312
AK089647	2543	162	17071	16549
AK089658	32106	33948	35160	33357
AK089670	31985	38920	5269	37543
AK089673	28362	29838	32761	31034
AK089699	19952	19839	20249	22023
AK089700	31750	33526	2470	2172
AK089714	8069	38586	37497	37018
AK089722	32137	33437	36062	34074
AK089734	14898	16527	36021	34394
AK089745	33304	40982	40507	10134
AK089757	39714	10814	7338	8121

AK089760	30759	32374	33956	32423
AK089774	24417	25664	40997	26751
AK089783	29208	31017	31009	29755
AK089799	29920	31675	32181	31295
AK089848	27681	29002	31970	30442
AK089854	24215	25325	27275	26143
AK089856	13303	3702	7445	4267
AK089883	27063	28343	30119	28859
AK089909	6020	6302	20351	17113
AK089910	31243	32937	34367	33071
AK089924	27014	28374	30861	36812
AK089955	5929	6333	8907	496
AK089956	13455	14215	23518	17612
AK089990	34299	21017	23846	23099
AK090094	25005	26261	27697	39687
AK090144	23433	24577	29204	25535
AK090164	3709	37998	38812	2752
AK090167	30525	32655	32504	30962
AK090237	25954	27273	29539	35441
AK090249.1	16194	11932	11246	4655
AK090297	28900	30406	31761	30640
AK090377	29854	38991	30070	28382
Ak1	5762	10324	8841	16441
AK134496.1	31396	11295	25860	7687
AK149454.1	4854	11966	16060	24761
Ak2	21167.33333	28184.66667	23045.33333	30147.66667
Ak3	13276.66667	21677.33333	31777.66667	19748.33333
Ak4	40159	19008	8912	23465
Ak5	9597.333333	17002	28460	29765.66667
Ak7	31363.66667	29064.33333	21923	22161.66667
Ak8	19009	8920	13018	3306
Akap1	13560.66667	15625.33333	12526	15128.66667
Akap10	21620.85714	26431.71429	19216.57143	22534.28571
Akap11	25850	21178.5	6615.5	3532.5
Akap12	23077	29430	29135	39696
Akap13	24390.6	18368.4	22481.8	22862.4
Akap14	31997.5	33180	16264.5	14745.5
Akap17b	18028	11865	17709	13156
Akap3	33675	14362	4764	9312

Akap4	15732	14200.5	25179	22450
Akap5	40962	1085	35458	40713
Akap6	20580	23847.66667	15677.66667	29769.66667
Akap7	26633	28079	19248	37504
Akap8	12820.5	13541	14455	14443
Akap8l	17969.5	8391.5	14302	12582
Akap9	19180.66667	19024.33333	19685.66667	17938
Akd1	11703	12770	16183	17947
Akip1	7120.5	17329	11782.5	18603.5
Akirin1	27861.5	25863	20125.5	25664
Akirin2	15986	24414	16902.5	31548.5
Akna	13466	15065	12741	17022
Akp3	34827	3244	3951	5636
Akr1a1	38642	35473	2260	1578
Akr1b10	32088	20742.66667	22829	28888.33333
Akr1b3	16302.33333	14606	19979.66667	17759
Akr1b7	9062	7920	10943	9351
Akr1b8	18358	21790	21975	29614
Akr1c12	11430	20505	15897	22918
Akr1c13	34533	2999	39893	5010
Akr1c14	25948	27284	7925	10792
Akr1c18	661	19003	19407	23045
Akr1c19	17916.5	23428.5	23936.5	29569.5
Akr1c20	20387	21472.5	28974	22007.5
Akr1c21	34245	32521	2068	739
Akr1c6	9821	13406	19135	15054
Akr1cl	23273.66667	28166	27415	27935.66667
Akr1d1	23343	24432	27952	25221
Akr1e1	34827	37730.5	32107	33946
Akr7a5	6067	5158	39462	36786
Akt1	20617	19797.5	18468	16129
Akt1s1	11468	12298	8355	7317
Akt2	24607.5	24707.5	26451.5	23664.5
Akt3	5451.333333	4902.333333	7492.666667	5380
Aktip	13521.5	19333	19418.5	20819
AL024073	29670	17175	22359	22052
AL626805	28240	32651.5	39997	15083
AL833780.6-2	2845	35834	9022	32512
Alad	15149	30987.5	26201	29188

Alas1	13461.5	10023.5	13599.5	12310
Alas2	39661	2904	2952	38843
Alb	9661	13309	13513	11953
Alcam	16751.66667	19678.66667	17207	13328.33333
Aldh16a1	14744.5	13477	11857	10066.5
Aldh18a1	10429	9962	8337	6199
Aldh1a1	3184	2042	9735	8565
Aldh1a2	3501	20	1782	463
Aldh1a3	30656.5	31893	16212	21421
Aldh1a7	30730	40641	39865	40902
Aldh1b1	20399	17700	15688	14111
Aldh1l1	2496	3824	954	39586
Aldh1l2	15637.5	15895.5	8448.5	11822.5
Aldh2	21978	12855	13204	11964
Aldh3a1	30316	31980	1065	31333
Aldh3a2	19873	18252	16738	16639
Aldh3b1	37668	37709	39364	1551
Aldh3b2	17600	17205.5	18572.5	1446.5
Aldh3b3	16428.5	35621.5	19908.5	18353.5
Aldh4a1	12218.5	11812.5	14603	13803
Aldh5a1	18430.5	14115.5	21777.5	19330
Aldh6a1	13165	12462	12892	13119
Aldh7a1	14355	16006	16506.5	17050.5
Aldh8a1	10225	10988	15191	15745
Aldh9a1	18176	22356	24911.5	20233
Aldoa	14836.5	17450	18994	24954
Aldoart1	398	17034	14682	306
Aldoart2	20334	7866.5	8940	11998.5
Aldob	23736.33333	21365.66667	22969.33333	22533.66667
Aldoc	18358.5	21854.5	10387.5	13405.5
Alg1	5946	3533	4220	4129
Alg11	32534.5	30331	24679.5	26295.5
Alg12	19555.66667	18676.66667	18065.33333	19037.33333
Alg13	20988.33333	22585.66667	24710	24343.66667
Alg14	7471.5	10530	7771	8805
Alg2	13664	14228	12519	13985
Alg3	35748	31032	24861	31382
Alg5	11336.5	14125	12483	12357.5
Alg6	16910	17901	16185	16090

Alg8	32933	29805	20557	24221
Alg9	21839.66667	14367.33333	13626	19349.33333
Alk	29430.33333	25909	23236	16089.66667
Alkbh1	37224.5	25671	27293.5	25086.5
Alkbh2	23279	535	36892	2009
Alkbh3	15963.5	15377.5	16210.5	18126.5
Alkbh4	7071	5391	2669	1472
Alkbh5	35141	33416	37606	38046
Alkbh6	17039	18255	17102	18011
Alkbh7	21261	9236.5	6697.5	8248.5
Alkbh8	28523	27204	13768.33333	16613.33333
Allc	12742	11223	10228	12965
Alms1	15699.33333	9849.333333	10494.33333	15118.33333
Alox12	11873.33333	28377.66667	33937.33333	32025
Alox12b	7692.5	28478.5	31668	32774.5
Alox12e	22714	25399	39512	40821
Alox15	22998	20710	23578	29987
Alox5	11218.33333	12282	23650.33333	23081.33333
Alox5ap	26205	25511	26699	24805
Alox8	24489	36544	37676	34250
Aloxe3	7525	31312	11956	9741
Alpi	11762	15572.5	15775.5	14499.5
Alpk1	35585	26442	31246	36217
Alpk2	2216	1609	13141	15416
Alpk3	5244	3758	2482	4427
Alpl	37487	36909	3298	4514
Alppl2	4330	8869	10039	10491
Als2	18459.5	17155.25	15514.5	14059.75
Als2cl	16665.5	20712	23331.5	23787.5
Als2cr11	32265.5	16074	33484.5	34247
Als2cr12	36936	34468	34912	33537
Alx3	9827	13427	19560	18925
Alx4	27534	14808.5	33488.5	18152.5
Alyref	2815	10400	11507	11287
Alyref2	14669	22464.5	24576	26066
Amacr	10987	10998	8647	8627
Ambn	31798	33529	35612	1292
Ambp	5527	10297	10143	17636
Ambra1	22290	21627.75	28717.25	21230.75

Amd1	22265.5	19927.5	17762.5	22392.5
Amd2	31549	22164	19489	18828
Amdhd1	37289.5	30115	20539.5	23450.5
Amdhd2	30326.5	33259	24003	26598.5
Amelx	15018	34461.5	18504.5	15704.5
Amer1	5773	1517	897	38603
Amer2	20905.5	20598.5	27371.5	26474.5
Amer3	19192	18937	24871	23397
Amfr	40149	39346	38910	36743
Amh	14880	12533	16522	16785
Amhr2	16459	12908	25674	25961
Amica1	26364	38629	31715	37639
Amigo1	18491.5	19272	22968	29252.5
Amigo2	34198	27630	30640	40325
Amigo3	2959	1655	2083	4619
Ammecr1	16774.5	27957	12511	7717
Ammecr1l	22401.5	21730.5	21286.75	19764.75
Amn	10845	14382	6198	11789
Amn1	28345	29032	30517.33333	21044.66667
Amot	24460.33333	22809.66667	27069	26286
Amotl1	9195	26080	38034.5	40580
Amotl2	4745	6064	11398	12124
Ampd1	31596	33337	5882	350
Ampd2	22710	21423	21121	23608
Ampd3	7534	24449.5	11987.5	30000.5
Amph	23711.5	18124.25	20512	26751.25
Amtn	11997	14846	19581	19351
Amy1	15111	16695	16920	18017
Amy2a5	23049	25168.66667	26024.66667	25352.33333
Amz1	20647	10521	19749	11945
Amz2	23021.5	22524.5	19904	24145
Anapc1	17219.16667	24045.5	17357	22064.66667
Anapc10	20965	21359.5	20216	21959.5
Anapc11	20482.33333	20619	21065.33333	20046.33333
Anapc13	17973	20227	19176	17579
Anapc15	14807.33333	12717.66667	13806.66667	24871.66667
Anapc16	28520.5	26160	24003	22943
Anapc2	31312	26972.33333	26656.33333	25522
Anapc4	6630	8213.5	5857	8110.5

Anapc5	29989	26082.5	24470.5	25439
Anapc7	34110	32046	31460.5	33461.5
Ang	26087	33351	37818	37088
Ang2	13588	8080	18766	13853
Ang4	5706.5	31401.5	23733.5	11983
Angel1	22518	22795	24250	24287
Angel2	13241.5	11743	9382	7583.5
Angpt1	28117.75	29735.25	25565.5	22923.25
Angpt2	35815	37694	2291	40677
Angpt4	1872.5	13429	20890.5	21390.5
Angptl1	25499.5	32691	33914	27661
Angptl2	17383.25	31241.75	16046.25	19698.25
Angptl3	19177	26006.5	20496	12511.5
Angptl4	10526	2123	9092	36750
Angptl6	15047	16463	17975.5	16130.5
Angptl7	39746	18689	37547	24085
Ank	24864.5	2704	18536	5891
Ank1	18604.2	17742.2	13774.8	20051.6
Ank2	21426	25657.57143	28299.28571	20017.14286
Ank3	18630	25899.5	23081	18015.5
Ankar	437	881	9008	4271
Ankdd1a	40929	5216	18834	23895
Ankdd1b	40593	31613	33630	31583
Ankef1	29932.5	35038	1237	20819
Ankfy1	26764	21665.5	22501.5	24332.5
Ankhd1	16721.57143	26842.42857	23948.28571	21384.14286
Ankib1	23158.5	16660.83333	20892.83333	22075.5
Ankk1	12811	2371	27716	22207
Ankle1	24178	31702	32703	33362
Ankle2	17730.5	12698	12069	13383
Ankmy1	24920	26424	29966	27206
Ankmy2	24985	25688.5	24061.5	23372
Ankra2	17345.33333	17394.33333	13747.66667	11556.33333
Ankrd1	23988	23694	29301	855
Ankrd10	4386	5289	4340	2147
Ankrd11	18994.5	25798.5	25340.5	24619.75
Ankrd12	25391.85714	24542.57143	22981.71429	24364.85714
Ankrd13a	16622	18049.5	13509.5	12538.5
Ankrd13b	896	27346	4523	35963

Ankrd13c	3837	7811	3909	4861
Ankrd13d	9304	7795	2292	4296
Ankrd16	23268.5	17736	19932	23132
Ankrd17	21025	23288.4	10700.8	10887.4
Ankrd2	24428	32236	33375	32799
Ankrd22	25231.5	33781.5	16784	35759
Ankrd23	17405	15196.5	17966.5	17730
Ankrd24	15183.66667	26926	24934.33333	19997.66667
Ankrd26	6493.2	19172.2	16203.4	19104.4
Ankrd27	16808.33333	16968.66667	13496.66667	15028.33333
Ankrd28	27038	20260	27906	23142
Ankrd29	20731	20723.5	18440	22939.5
Ankrd31	31647	33342	34757	32996
Ankrd33	4864	10143	9652	11712
Ankrd33b	25905.75	22240.25	27149.25	27624.25
Ankrd34a	13685	18872	18294	19316
Ankrd34b	29383	30851	33259	31656
Ankrd34c	28757	26898	7566	455
Ankrd35	25650	28186	36094	33834
Ankrd36	24387.5	25732.5	26746.5	26002.5
Ankrd37	40928	1649	17776	14425
Ankrd39	17281	17777	15967	16701.5
Ankrd40	25175.33333	18872.33333	18848	17856.66667
Ankrd42	20957.33333	10580.66667	23270.66667	23295
Ankrd44	16699.25	18352.5	17547.5	17364.25
Ankrd45	18746	16403	24875.5	25764
Ankrd46	29883	28664	25593	24038
Ankrd49	10668	10019	10726	11049
Ankrd50	5409	7259	11973	12125
Ankrd52	34423.5	25943	24742.5	25997
Ankrd53	1038	38785	3818	451
Ankrd54	20890	20059	18462	18474
Ankrd55	7946	8346	13617	8480
Ankrd6	8785.5	13864	17864.5	20039.5
Ankrd60	13119.5	13161.5	16285	19053.5
Ankrd61	16776	13060	8953	18080
Ankrd63	4819	10739	15279	12807
Ankrd7	28404	35097.5	22000.5	3657
Ankrd9	24743	8570	25866.5	27547

Anks1	33522	22948	25810	21501
Anks1b	24122.66667	24854.66667	26927	25676.66667
Anks3	21704.5	13358.5	15927	19628.5
Anks4b	25827	27092	29140	33387
Anks6	20297	19939	27438	25579
Ankzf1	17600	15939	14589.66667	20049.66667
Anln	20821	9631.5	18665.5	18103.5
Ano1	32379	33998	36112	33549
Ano10	25010.5	20935.5	19328.5	21259
Ano2	35429.5	19653.5	22024.5	21754
Ano3	38210	36731	40392	39389
Ano4	17169.66667	17423	30803.66667	17737.33333
Ano5	33639	4936	3412	36609
Ano6	11898.75	7958.5	21083.75	19034
Ano8	14394	14759.5	14876.5	14171
Ano9	1683	36235	38140	5174
Anp32a	11117.5	29049.5	25589.5	22924
Anp32b	20941	17433.5	28547.5	15201
Anp32e	10399.5	12268	13332.5	13384
Anpep	3561	37153	4924	274
Antxr1	20830	39829	25619	8393
Antxr2	18669	35508	22459	36104
Antxrl	21987.5	7687.5	29257.5	14293.5
Anxa1	7077	38358	9404	3687
Anxa10	4955	10562	39519	37857
Anxa11	11051.5	27166	9640	27054
Anxa13	21781	24951.5	25662.5	18838
Anxa2	22529	15758.66667	18181	16670.66667
Anxa3	21671	15207.5	33691	35002
Anxa4	27184.66667	26586.66667	25292.33333	32511.66667
Anxa5	3299	39331	3008	4568
Anxa6	16997	10487	32154.5	29070
Anxa7	13394	28811	30183	28669.5
Anxa8	31261	27866	983	38394
Anxa9	3437	92	39396	36497
Aoah	17255.5	4013	15751.5	21349
Aoc2	34352	32487	35310	37689
Aoc3	29401	3436	4960	39985
Aox1	20872	39448	24462	4507

Aox3	28783	24814	19048	34457.5
Aox4	32920	34496	35913	36504
Ap1b1	30743	21397	25937	20883
Ap1g1	29718.5	22750.25	13334	24668.5
Ap1g2	6462	5083	3928	4138
Ap1m1	3114	1736	2201	1758
Ap1m2	13119	17605	16938	17457
Ap1s1	34389	35499	40584	39909
Ap1s2	20111	18507.66667	27508.66667	13562.33333
Ap1s3	12715	12570.5	11796.5	10150.5
Ap2a1	38414	39200	35402	36267
Ap2a2	8945	2536	4612.333333	2250.333333
Ap2b1	25998.5	22450.5	21879	23794.5
Ap2m1	12085.6	17573.4	10553.6	10245.4
Ap2s1	36493	18602	38222	38802.5
Ap3b1	13608	13931.5	15380	17631
Ap3b2	27780.5	28435	21473.5	21362
Ap3d1	8630	23228.5	17773	15337
Ap3m1	25163.75	27340.5	25526	20046.75
Ap3m2	34307.5	32742	31207.5	32863
Ap3s1	19690.25	26069.5	26146.5	15786
Ap3s2	26108.5	27611.5	23639.5	26280
Ap4b1	12336	14161	13020.5	15808.5
Ap4e1	20782.66667	20596	6009	22442.33333
Ap4m1	19687	17401	15092	15794
Ap4s1	40338	1249.5	1172	37285.5
Ap5m1	19117	19716	20008.33333	19224
Ap5s1	19232	16311	13690	15362
AP5Z1	8182.666667	20131	22640	21205.33333
Apaf1	15466.66667	13937	13156.66667	16040.66667
Apba1	22243.5	17527.5	4189.5	5183
Apba2	11632	17521	17512	16128
Apba3	7989	9170	9102	9729
Apbb1	30500	20309	38448	22441
Apbb1ip	15901.5	24745.75	25375.75	27880.75
Apbb2	23167.5	20957.25	27324.5	23430
Apc	12207.2	14982	17290.8	13588
Apc2	11694	15499	16979	17448
Apcdd1	21103.33333	20305	26739.33333	26660.33333

Apcs	28391	35564	3482	3391
Apeh	20433.5	38100	36865.5	19923
Apex1	36739	1244	33002	79
Apex2	29155.25	19929.5	27898.5	35722.5
Aph1a	29149	22525.5	26257.5	21218.5
Aph1b	22415.33333	21888.66667	27801.33333	19783
Aph1c	8090	3210	2607	37161
Api5	22543	19419.5	18212.5	21499
Apip	37384	4031	2369	2462
Apitd1	26571.5	11325.5	12849.5	15867
Aplf	12211	11615	9589.5	10432.5
Apln	10447.66667	12779	13103.66667	14317
Aplnr	25954.5	6965	9511	8612.5
Aplp1	7321	11066	16075	18314
Aplp2	17958	17327.33333	18369.66667	19134.33333
Apmap	29771.5	33333.5	28281.5	36498
Apoa1	10421	7140	11389	10012
Apoa1bp	14150	15420	14613	14185
Apoa2	3639	1005	7477	40818
Apoa4	13964	8813	13161.5	16407
Apoa5	31295	32384	20642	14923
Apob	13542.6	19826.8	23510.8	15716.6
Apobec1	19914.5	8585.5	17779	10224.5
Apobec2	26159	27453	29349	31299
Apobec3	11545.33333	16752	19953	14842.66667
Apobec4	40372	39738	6906	8962
Apobr	8893	5811	12175	8161
Apoc1	33809	36451	10870	9433
Apoc2	24804	26180	32025	28299
Apoc3	29463.5	29335.5	30823	31415
Apoc4	32092	21751	864	25806
Apod	3438	3267	19841	21966
Apoe	15652	2337	19208	11181
Apof	9758	3874	33662	36571
Apoh	33027	32839	33683	40630
Apol10a	40148	4321	3416	2818
Apol10b	33238	34903	5998	8089
Apol11b	21377	22605	33474	36447
Apol6	12412	10985	16750	16958

Apol7a	22593.5	24675.5	15959	26720
Apol7c	28202.5	12514.5	29200.5	22121.5
Apol8	31686	25596	29482.5	19468
Apol9a	36073	32775	5031	11803
Apol9b	28532	4042	5912	12600
Apom	22174	25248	40275	38376
Apon	27312	30143	30406	34063
Apoo	30571	33179.5	29243.5	29596.5
Apool	12160.4	14480.4	7988.8	10391.2
Apopt1	28444	29622.5	27794.5	10640.5
App	20403.42857	18927	27840.85714	22556
Appbp2	10188.75	19361.5	18835	18387
Appl1	18628	19234	18476.5	20317.5
Appl2	28380	20884	25825	23310
Aprt	16024	29312.5	38013.5	35972
Aprt-ps	31705	33189	2099	36751
Aptx	15044	21072	17716	20750
Aqp1	3375	37457	19624	15805
Aqp11	11050	7486	11313	3324
Aqp12	2984	2459	6244	4187
Aqp2	17873	20083	25311	27257
Aqp3	34207	34210	4753	3299
Aqp4	21285.66667	19769.66667	34890	35262.66667
Aqp5	4938	23266	4415	24427
Aqp6	27410	23841	1724	40217
Aqp7	9570	11693	12878.5	16598
Aqp8	7654	11339	14276	13635
Aqp9	17662.33333	27346	20199	20467.66667
Aqr	21133	20506	17131.66667	18509.33333
Ar	38878	39145	40663	55
Araf	34612.5	34011.5	38811.5	36900
Arap1	7734	8308	8467	9906
Arap2	37137	28903	22782	26013
Arap3	26632	27560.5	7100.5	7382.5
Arc	32240	30872	690	416
Arcn1	22083	22019.57143	19249.42857	21148.85714
Areg	31409	32863	34450	32660
Arel1	36846.66667	36030	37075.33333	36274.66667
Arf1	13176	11477	11188.5	13825.5

Arf2	5554	8398	4872	8045
Arf3	10752	26773	31469.5	28681.5
Arf4	7858	6970	7021	4692
Arf5	17749	21227	15508	16829
Arf6	21021.5	23189	27452	22729.5
Arfgap1	24218.66667	16867	17486.66667	17464.66667
Arfgap2	4160	5862.5	21673	3513.5
Arfgap3	19100.33333	20516	17573.33333	20505.66667
Arfgef1	23222.33333	33312.66667	34899.66667	32304.66667
Arfgef2	6890.5	4344.5	21993	20067
Arfgef3	18731.5	9959.75	24579	25410.25
Arfip2	23160	5683.5	5278	5279
Arfrp1	12805.33333	15619	14781	16395.33333
Arg1	39729	16561	21155	1198
Arg2	37803	4730	1294	2279
Arglu1	29065.66667	26967.33333	15606.33333	18181.33333
Arhgap1	14303	11835	10325	9550
Arhgap10	20132.66667	30122.66667	27822	25449.66667
Arhgap11a	11925.5	20157.5	10943	4634.5
Arhgap12	15699.33333	7311.333333	22377.33333	9348.666667
Arhgap15	28086.66667	15258.66667	22920.66667	32563
Arhgap17	30093.5	17234	20531.5	14709.5
Arhgap18	17272	2996	15062	40516
Arhgap19	6500.333333	18547.66667	11833.33333	24870.33333
Arhgap20	21735.75	17836.5	13720	15371.5
Arhgap21	10204	11065	9910	9813
Arhgap22	1747	7044	9312	17913
Arhgap23	15801	19851	20010.5	20655.5
Arhgap24	20285	30833.8	27136.2	21347.2
Arhgap25	23091	7545	25249	14316
Arhgap26	18575.5	21057.75	22330.75	24632.25
Arhgap27	17527	17192	4590	15371
Arhgap28	5434	19507	16795	17596
Arhgap29	31976.5	29229	17012.5	20595.5
Arhgap30	8402	2502.5	6825	21814.5
Arhgap31	27714	3401.333333	14309.66667	18048.66667
Arhgap32	23952.5	26953	27243.5	13079.5
Arhgap33	3007	5443.5	8016	8116
Arhgap35	14084	12624	14079	14684

Arhgap36	25082.5	26088	31002	34492
Arhgap39	752	22610	33651	20645
Arhgap4	12389	5774	7420	711
Arhgap40	31973	33828	6513	1370
Arhgap42	21029.16667	21193	28175.5	17751.66667
Arhgap44	14985.66667	14605	15743.33333	18932
Arhgap5	26287.75	21914	24147.5	24278.25
Arhgap6	30295	21114	34895	23349
Arhgap8	7349	14074.5	12787.5	10168.5
Arhgap9	3168	37515	3653	34463
Arhgdia	17988	14761	17426	16234
Arhgdib	17497	20568	17602	20106
Arhgdig	36888.5	5881.5	22089.5	18457
Arhgef1	6488	3848	6402	1947
Arhgef10	10587.5	11843.5	17690	15251.5
Arhgef10l	22485.5	21672	24590	20651
Arhgef11	15075.33333	11945	13930	23595.33333
Arhgef12	14156.25	18993.25	20107	24351.25
Arhgef15	15908	18866.66667	17279.66667	21159.66667
Arhgef16	10734	6231	14909	11264
Arhgef17	18594	21132.5	23829.5	25695
Arhgef18	7727.5	20993.5	25906.5	21313
Arhgef19	21195	22951	26149	32176
Arhgef2	20193.5	18955.5	16877.25	16363
Arhgef25	6618	9145	12025	18938
Arhgef26	26713	22042.6	25477.4	23432.2
Arhgef28	21518.5	10732	9738.5	9981.5
Arhgef3	22477	22231	23286	24525
Arhgef37	34923	809	2335	1959
Arhgef38	12822.5	15801	32563.5	13995
Arhgef39	25660.5	18967.5	31009	24579.5
Arhgef4	40785	38075	27324	27835
Arhgef40	29035.66667	29729	26264.33333	31099.33333
Arhgef5	21718	20562	37933	34956
Arhgef6	16564.25	4984.75	8531	20351
Arhgef7	7191	7243	5577	21655
Arhgef9	21700.5	16047	22587.5	16961
Arid1a	35127	24538	29065	27527
Arid1b	25214.66667	16426	22008	15759

Arid2	32610	26989.66667	27713	30175
Arid3a	36794	28238	35483	26208
Arid3b	23959.5	19406.25	21476.5	20475.25
Arid4a	18048.33333	22042.33333	27218.66667	22182.66667
Arid4b	19154.75	9051	16606	17790.5
Arid5a	19148	20803	16072.5	20848
Arid5b	13515	13926.5	16202.5	25490.375
Arih1	17791.45455	25872.36364	21826.54545	19887.27273
Arih2	29842	22467	23876	24846
Arl1	17906.5	1594	40367.5	2167
Arl10	21224.5	18763	19352.5	17873.5
Arl11	33361	21465	24346	21909
Arl13a	17445	17341	29044	31692
Arl13b	20838	19320.33333	31380.66667	29066.33333
Arl14	34686	1110	40838	37090
Arl14ep	31168	20012	32330.66667	33396.33333
Arl15	19291.5	26723.75	26960	26255
Arl16	40460	40131	33979	38944
Arl2	23529	29827	25573	30370
Arl2bp	22521	23119.5	4467	23527.5
Arl3	30116	38483	35051	37177
Arl4a	13656.66667	15408	17823	17144.66667
Arl4c	18858.66667	28261.33333	24480.33333	20643
Arl4d	16384.5	14894	18307	19243.5
Arl5a	28067	18734	8071	18534.5
Arl5b	23433.5	14740.25	15768	6680.25
Arl5c	3343	9694.5	14460	22517.5
Arl6	1521	6852.5	7236.5	6984
Arl6ip1	9632	5335	10711	7681
Arl6ip4	31045	15087	33872	34490
Arl6ip5	20935	19660	19617	23549
Arl6ip6	11523.33333	13159.66667	12783.33333	12059.33333
Arl8a	23871	23492	25374	28051
Arl8b	24289.25	26121.75	17531.75	18941.75
Arl9	21953	23373	27876	28001.5
Armc1	28930	26303.5	26855.5	29385.5
Armc10	15594	16564	15121	16362.66667
Armc12	3161	2457	7756	6005
Armc2	8167	7340	14343	5106

Armc3	36921	21830	34416	18552
Armc4	32613	17484	20020.5	22316
Armc5	19016	16220	14660	16181
Armc6	3837	3049.5	2868.5	21149
Armc7	8474	5526	6722	7221
Armc8	23073.33333	22994.33333	9235.666667	23787.33333
Armc9	23035.2	23132.6	12409.2	16819.8
Armcx1	9633	7338	11590	7977
Armcx2	27247	24218	23955	24229
Armcx3	14115.5	12446	13231.5	13956
Armcx4	37682	13851.5	20383	13952
Armcx5	40454	38139	38733	37758
Armcx6	35543	30318	40291	36607
Armt1	14608	20262	18697	20075
Arnt	16962.6	17367.4	20615	20887
Arnt2	11409	4146	8427	12988
Arntl	20598.5	32573.5	16625.5	17562.5
Arntl2	36200.5	32239.5	34771.5	20464.5
Arpc1a	361	38999	2	39762
Arpc1b	8674	6837	9763	11010
Arpc2	25437	21958	21958	20838
Arpc3	12336	15204	36736	16757.5
Arpc4	17357.5	17581	15072	14557
Arpc5	20556	19718	21974	22349
Arpc5l	37861	40754	39069	38422
Arpin	26773	6496.5	31578	26644
Arpp19	22425.5	4375	22490	26166
Arpp21	27518	26993.83333	29936.16667	24217.16667
Arr3	29989	8978	33548	10455
Arrb1	20208.5	20645.5	19130	21106
Arrb2	36854	37521	455	40511
Arrdc1	18427	16169	14412	14039
Arrdc2	23392	20390	22546	23884
Arrdc3	3962.5	19399.5	17806	24953
Arrdc4	16034	32343	19680	37128
Arrdc5	23347	26380	30700	32271
Arsa	8705	4939	12538	8840
Arsb	10275	37746	39962	18237
Arsg	10910	3062	14128	5807

Arsi	40876	1385	7529	7592
Arsj	28803	35774	1955	30580
Arsk	37448.5	33313	33553.5	28990.5
Art1	3303	36913	6900	5247
Art2a-ps	2956	18715	11552	20978
Art2b	23141	6737	26719	25621
Art3	26367	28994	29555	28228
Art4	29725	40393	7824	37840
Art5	39864	40800	4732	2483
Artn	38579	19955	6706	24834
Arv1	31874	40196	39129	1231
Arvcf	22159.5	35805	6501	15131.5
Arx	25104	26876.5	29998	30219
Arxes1	2775	40123	34128	41138
Arxes2	32436	34775	35563	1784
Arzc	12133	15820	22514	24558
As3mt	17806.5	19684	11922.5	15199.5
Asah1	20929	16755	19088	18334
Asah2	13442.25	18309.25	19834.75	18383.5
Asap1	21686.5	15061	11870	13137
Asap2	7824.5	18678	8774	11640
Asap3	38145	3518	31770	474
Asb1	3988.5	5833	6333	7154.5
Asb10	14438	7105	2777	12147
Asb11	22959	24844	24499	32420.5
Asb12	31912	33148	36311	34287
Asb13	16226	16900	17325	15242.33333
Asb14	12554	24579	464	39245
Asb15	28710	30158.66667	34702.66667	31172.66667
Asb16	15686	16960	23560	3158
Asb17	33175	34923	37141	2340
Asb18	35215	40710	1957	953
Asb2	39507	2795	11829	16643
Asb3	28417	36506	25330	17588
Asb4	23153.5	16448.5	23339.5	11653
Asb5	21235	21579	22551	18296
Asb6	28051	26664	25380	29159
Asb7	38303.66667	25395.33333	13615.33333	15168
Asb8	14259.33333	14749.33333	14499.66667	14730.66667

Asb9	29575	26781	39498	5108
Ascc1	26507	23966	22281	20954
Ascc2	25943.5	25252	25488	33353.5
Ascc3	15435.8	12851.2	15204	17631.4
Ascl1	39224	41107	4721	4553
Ascl2	30656	34756.5	22981.5	3923
Ascl3	27081	28188	13503.5	18531
Ascl4	32484	33928	35308	33478
Asf1a	11279	14091.5	28860.5	34127
Asf1b	3840	5532	5980	2652
Asgr1	15551	18308	23504	23612
Asgr2	26326	23755	27524	26372
Ash1l	27252.66667	15534.66667	15833.66667	15368.33333
Ash2l	18045.5	20217	18370	19522.5
Asic1	35945.5	15369.5	36566	36808
Asic2	25372.5	12056	36653	34890
Asic3	9886	9945	14955	15006
Asic4	14917	4320	7909	15646
Asic5	26154	27674	29187	28810
Asl	37000	39096	38621	39706
Asna1	4607	9131	8145	11727
Asns	13125	16117	16948	17303
Asnsd1	7223	6487	5023	3460
Aspa	10060.5	24507.5	18035	12852.5
Aspdh	16293	17073	24594	24321
Aspg	8954	8617.5	14841	14022.5
Asph	21319.6	10970.8	14098.8	14495.2
Asphd1	9430	13993	24339	21695
Asphd2	11159	14530	15461	15732
Aspm	24016.25	26578.5	28393.25	30740.75
Aspn	28596	30098	36321	30014.5
Asprv1	19076	22331	21131	28515
Aspscr1	5635	8640	3745	5523
Asrgl1	4441	6981	9363	5588
Ass1	20004.5	34922	4450	20110
Aste1	25010	26235	36412	33678
Astl	32261	35552	37337	36421
Astn1	17855	17808.33333	24567.33333	21291
Astn2	17665	28763.75	29907.5	31833.5

Asun	11386	14043	11639	12868
Asxl1	26996.33333	20964.66667	23847.66667	23564.33333
Asxl2	16102.5	23216.5	21287.75	23201.5
Asxl3	15292	24466.66667	17869	21709.66667
Asz1	37278	2107	39524	1393
Atad1	15843	17293.33333	16566.33333	18268.66667
Atad2	12521.25	18968.75	24325.75	14831.75
Atad2b	16836.25	9036.25	16849	16568.5
Atad3a	10378	13132	11366	12336
Atad5	13759.5	19941.75	25009.75	16111.75
Atat1	25733.66667	23500.33333	20743.33333	24635
Atcay	17793	20819.5	18768	20448
AT_D_3	34623	38275	2071	39327
AT_D_5	33669	36362	35356	38254
AT_D_M	33957	174	31298	31391
Ate1	15588.25	16388.25	17398	18148.75
Atf1	11277.5	11606.75	17119.25	16853.75
Atf2	19511.33333	19052.33333	19308	19861.5
Atf3	35004	24265	35028	28398
Atf4	38307	36088	31007	36648
Atf5	11955	10460	11495	13124
Atf6	29038.33333	28261.66667	30133.33333	28690
Atf6b	19054	22193	21723	21941
Atf7	19190.66667	29257.66667	30404	29101.66667
Atf7ip	14621.75	19368.5	11407.25	11607
Atf7ip2	37841.5	17904	27889	29789.5
Atg10	24114.66667	26506	12295.66667	21841.66667
Atg101	40799	40653	832	3688
Atg12	12898.66667	16860.66667	18173.66667	19375.66667
Atg13	8139	24262	23523.5	9517.5
Atg14	8075.5	22080.5	15789	13582
Atg16l1	34987	34724.5	34817.5	34767.5
Atg16l2	39105	3498	269	1136
Atg2a	20021	20017.66667	18250	17936
Atg2b	22322.66667	22397	22466	24890
Atg3	13266.66667	6520.666667	9602	12370.33333
Atg4a	13569	21634	12082	19557
Atg4b	11478	12869.5	10645	13216.5
Atg4c	13712.5	19054	21848.75	21060

Atg4d	11397.33333	13027.66667	16114.33333	16082.66667
Atg5	31184	2126	30225	38395
Atg7	28313	22981.66667	24129.33333	10794
Atg9b	11255	14132	15691	18675
Athl1	1218	39531	36759	35699
Atic	19146	20862	16823	19511
Atl1	15523	13209	23984	21130.66667
Atl2	10904.33333	15893.66667	16872	18527.66667
Atl3	24839.4	24545	22387.8	24677.6
AT_L_3	34441	33184	36162	35042
AT_L_5	30386	36372	41104	30801
AT_L_M	37792	39309	37879	40318
Atm	16518.5	11983.5	11284	11138.5
Atmin	11945	30635	12552	30446
Atn1	11900	12387	14146	13546
AT_nB_3	6887	5605	12893	14831
AT_nB_5	37465	38854	40299	40276
AT_nC_3	8197	3250	884	2714
AT_nC_5	31192	29928	36205	39846
AT_nD_3	13030	13319	22644	20277
AT_nD_5	139	1968	1009	5202
AT_nX_3	39836	35870	33290	38324
AT_nX_5	35237	32468	33125	37563
Atoh1	17091	19943	19952	19007
Atoh7	21885	25709	24915	24724
Atoh8	20158	23408	24859	26888
Atox1	24035	34402	25809	36698
Atp10a	13642	12349	12631	12502
Atp10b	36103	32373	9539	10487
Atp10d	23671	14230.33333	20970.33333	14889.33333
Atp11a	11505.5	15083	14913	17741
Atp11b	8764.75	7075.75	7155.5	14494.5
Atp11c	19400	14848	21778	21179
Atp12a	16981	17817	27792	26725
Atp13a1	23930	22014	18803	18923
Atp13a2	34937	37053.5	25410	28108
Atp13a3	34963	25630	24721	29565
Atp13a4	10722	8506	18320	24841
Atp13a5	30771	31926.5	32096.5	16816.5

Atp1a1	21322.66667	20799.33333	27074.66667	28664.66667
Atp1a2	10539	9850	14937	18112
Atp1a3	7739.5	4283	23872	21375
Atp1b1	34713	38815	2380	5980
Atp1b2	15483	18365	21203	21680
Atp1b3	21413.75	22756.25	20611.75	22708.5
Atp1b4	23592	34734	26664	25698
Atp2a1	27437.5	36141	15794.5	19590.5
Atp2a2	22093.75	13776	11218.25	13308.75
Atp2a3	527	516	3099	3221
Atp2b1	10540.8	19035.4	14998	20640.6
Atp2b2	20651	21174	28101	24839
Atp2b3	36152	39759	40722	1985
Atp2c1	9947	21510.75	14241.5	14923.75
Atp2c2	37775	39992	9061	38821
AT_P_3	1054	398	2772	2304
Atp4a	20833.5	25807.5	29615.5	27721
Atp4b	8722	8974	15458	13025
AT_P_5	33928	30369	36423	31801
Atp5a1	25231.66667	21595.66667	22436	24098.66667
Atp5b	18474	17945.66667	14699	21462.33333
Atp5c1	38195	943	38989	39114
Atp5d	3575	2943	814	2275
Atp5e	35508	405	756	748
Atp5f1	29084	25216	22251.75	26377.25
Atp5g1	6704	10547.5	7794	8252.5
Atp5g2	23670.66667	24734.66667	12090.66667	25098
Atp5g3	37268.5	950	5169.5	20867
Atp5h	27942.5	32489.5	31375	31746
Atp5j	22202	26455	23366	22622
Atp5j2	14190	12972	11840	12859.5
Atp5k	38522	2444	146	40631
Atp5l	21782	33083	28472	30792
Atp5o	21516	25502	23565	24305
Atp5s	27692	29250	24053	24369
Atp5sl	8728.5	3643	15190.5	16566
Atp6ap1	14423	14988	14728	14727
Atp6ap2	13931	7544.5	8741.5	9317
Atp6v0a1	18976.66667	19905.66667	14782.33333	17411.33333

Atp6v0a2	6557.5	26004.5	23071.5	26511
Atp6v0a4	29131	16877.5	33391.5	36737
Atp6v0b	18501	23530	21520	33471
Atp6v0c	12850	9507	4073	8574
Atp6v0d1	19479	16134	13785	16436
Atp6v0d2	18340	10557	17569	11951
Atp6v0e	39955	4382	742	2618
Atp6v0e2	12961.5	11675.5	20612	27438
Atp6v1a	18599.5	11040.5	14312	13826
Atp6v1b1	6615	11430	10600	15084
Atp6v1b2	8159	3535	8737	5252
Atp6v1c1	20329	19283	22414	20062
Atp6v1c2	33408	30435	32530	30977
Atp6v1d	24736	28110	27960	35775
Atp6v1e1	11173	14824.5	10134.5	19288
Atp6v1e2	18878	21383	25323	24979
Atp6v1f	31633.5	16884	12730.5	15219
Atp6v1g1	20981.5	7765	7498.5	10437.5
Atp6v1g2	13337	13752.5	15530	15364
Atp6v1g3	29075.5	40000	35355.5	39203.5
Atp6v1h	8157.333333	7803	5095.333333	10611.66667
Atp7a	4295	38637	4615	40186
Atp7b	40077	3013	3788	3969
Atp8a1	16540	24082.4	29260	20477
Atp8a2	21957.5	28683	29520	16825.5
Atp8b1	31633	30995.33333	33780.66667	36787
Atp8b2	22198	27870.5	23493	27513
Atp8b3	10359.5	12105.5	13611.5	11127.5
Atp8b4	27650.5	14862	17083.5	11690
Atp8b5	21906.5	26313.5	31403	12934
Atp9a	3845	40003	37823	36820
Atp9b	34066.5	31064	29749	29428
Atpaf1	17975	18576.33333	17423.66667	16036
Atpaf2	11760.5	11625.5	11639	13076
Atpif1	38702	14674.5	12714	12966
AT_P_M	37785	36749	1447	634
Atr	19237.4	18281.4	21618.2	19252.4
Atraid	12247	13489	13885	12674
Atrip	17292	17618	14737	16478

Atrn	16008.4	15459.2	16443.2	24322.4
Atrnl1	20788.2	20982	22976.4	22178.2
Atrx	13309.33333	23879.66667	13713	14284.66667
AT_ssM_AB_3	20860	10209	21011	16157
AT_ssM_AB_5	3116	28197	38521	32123
AT_ssM_GD_3	39339	36759	29408	38425
AT_ssM_GD_5	17587	12559	7743	18371
AT_ssM_PC_3	21615	8910	27150	11683
AT_ssM_PC_5	13782	14964	14680	15847
AT_ssM_RR_3	1549	6792	10149	21351
AT_ssM_RR_5	3376	17800	17137	25068
AT_ssM_TR_3	25061	946	36543	37778
AT_ssM_TR_5	22199	21126	12865	5934
AT_T_3	1586	627	2013	5933
AT_T_5	2158	37055	773	3760
AT_T_M	31648	36050	36275	32489
AT_W_3	28814	34389	32281	34490
AT_W_5	36541	35632	36074	37373
AT_W_M	7040	3453	8045	6190
Atxn1	19938	17105.66667	19321.66667	18294.66667
Atxn10	24034	23138	24312	23649
Atxn1l	14420.5	12157	18631	14222
Atxn2	19451	21148.4	21188.8	23367.4
Atxn2l	17769	13755	19039	17873
Atxn3	22568.5	19823	17754	20319
Atxn7	38300	21326	19064	17354
Atxn7l1	25101.33333	23426	31503.66667	33297
Atxn7l2	607	40225	2882	1077
Atxn7l3	24327	19619	17549	14778
Atxn7l3b	17113	27634.5	27267.5	26237
AU018091	32838	1710	28071	6710
AU019823	32476	27589	19826	19490
AU020206	11719	41119	7437	39306
AU021092	6512	10151	15435	13560
AU022252	34755	34746	24429	24142
AU022840	31526	33290	34856	33402
AU022855	5391	40572	862	31766
AU023762	33353	35815	150	3896
AU040320	17616.33333	15306	17744.66667	28945.33333

AU051595	35456	40876	39065	1399
Auh	28829	14953	14604.5	16880.5
Aunip	35550	37911	2217	5154
Aup1	832	40590	36054	37774
Aurka	40581	37809	1058	39160
Aurkaip1	20769	23537	26390	25980
Aurkb	1132	31585	34205	6962
Aurkc	12212	13924	13375.5	13380
Auts2	21591.22222	18711.88889	20619.55556	25782.11111
AV039307	19160.5	37616.5	23165	22059
AV046434	3294	7204	3674	493
AV046780	32373	33976	35187	33374
AV080718	10826	17628	17200	18684
AV166465	18099	23065	19910	22696
AV240687	40312	2084	1777	10052
AV242843	39314	40516	39894	223
AV280394	33022	30673	805	14501
AV372127	15443	12928	16897	18129
Aven	5417.333333	4349	6596	7073.666667
Avil	1819	25420	2058	36923
Avl9	22712.5	20414	18692	22355
Avp	11769	13478	8118	13002
Avpi1	396	14414	6295	17524
Avpr1a	35890	32350	32793	38171
Avpr1b	17771	19750	15037	18425
Avpr2	25795	5552	27308	1299
AW112010	5400	9026	9824	8487
AW209491	13366	16534	14106	13190
AW261531	32369	34763	34877	34163
AW476689	3457	37639	8570	10190
AW495116	30260	36143	8326	41088
AW549877	10223	11174.5	15574	16059
AW551984	33565	24564	20612.5	21136.5
AW554918	18923.66667	11996.33333	15075.33333	13154.33333
AW557046	33049	36272	2173	5717
AW702099	9991	7095	10000	3397
AW986112.1	11075	7330	9937	11405
Awat2	33746	30961	36958	334
Axin1	7804	4760	3743	2459

Axin2	38233	39547	7207	11351
Axl	38130	36931	19234	20187
AY036118	10902.5	22469.5	25509.5	23127
AY074887	6523	18091	15414	21349
AY248756	17195	28525	8392	13412
AY358078	2221	30300	39146	40831
AY498738	31808	33463	36036	5616
AY761185	10934	13770	16478	17554
Aym1	7005	15046	9119	7431
Azgp1	37716	39892	2202	642
Azi2	6782.666667	11260.33333	8270	14002
Azin1	17232.66667	22257.66667	17599.66667	11672.33333
Azin2	30213	38249	33532	35582
B130011D17Rik	25418	26694	28404	27271
B130011K05Rik	15759	14655	24006	29361
B130020M22Rik	26345	37214	3828	1691
B130021B11Rik	26601	28046	29462	28256
B130024G19Rik	31756	34177	34094	32430
B130024M06Rik	28652	30370	30429	33558
B130034C11Rik	25137	26367	28415	34932
B130040O20Rik	11933	8090	10051	13127
B130052P14Rik	17185.5	30704.5	35927	22917
B130063F10Rik	1722	32033	39217	3302
B130065D12Rik	19298.5	16788.5	34844	33666.5
B230104C08Rik	23314	24368	26317	25275
B230104C14Rik	37007	32554	31657	39083
B230110C06Rik	27938	29500	28379	27505
B230114P17Rik	25280	26755	27581	26491
B230117O15Rik	26687	28082	29538	28518
B230118H07Rik	4186	7509	5348	6956
B230118I11Rik	27175	28473	30990	29510
B230205o20rik	11157	38346	3719	2105
B230206F22Rik	16717	16691	22268	20349
B230206H07Rik	33744	34660	19586	11049
B230206I08Rik	25702	27027	28603	27520
B230206L23Rik	33270	170	4702	10795
B230208B08Rik	31576	33231	35003	33237
B230208H11Rik	35145	35425	40443	34626
B230209E15Rik	31370	35886	34494	32707

B230213L16Rik	30601	32173	34419	40333
B230214O09Rik	11777	37368	3317	4975
B230217C12Rik	9729.5	27929	27668	21270.5
B230217O12Rik	32124	28402	28149	22678
B230219D22Rik	23778.5	18821	13608	30871
B230219N05Rik	12417	21664	22608	23852
B230220B15Rik	26873	28284	29849	28489
B230220N19Rik	28060	29924	29649	28415
B230303O12Rik	15004	8905	16462	14614
B230308N11Rik	35081	23853	25573	26556
B230311B06Rik	17034	19085	17517	19370
B230317F23Rik	27539	30025.5	28446.5	10655
B230319C09Rik	17646	18054	16122	20995
B230325K18Rik	554	31336	28209	478
B230337E12Rik	15684	12104	12537	13728
B230340J04Rik	2576	12948.5	3250	40934.5
B230343J05Rik	26062	27322	5446	5212
B230354K17Rik	17654	18383	21365.5	23715.5
B230359F08Rik	15520	21641	21981	26319
B230369F24Rik	19182	12224.33333	11908.66667	27150.33333
B2m	21802.66667	24758.33333	20297.33333	14913.66667
B330016D10Rik	37845	2069	39136	2984
B3galnt1	19013.5	15880	22821	15854.5
B3galnt2	11831	11837	10716	13734
B3galt1	20744.66667	23978	26027.33333	26029.33333
B3galt2	27271.66667	30387	31810	25418.66667
B3galt4	12988	13485	13348	14607
B3galt5	33001	39663	30547	29155
B3galt6	34728	39649	36348	38816
B3gat1	19557	23503	30986.5	11532.5
B3gat2	13676	15537	31175	25808
B3gat3	24481.5	19980.25	19116.75	18211
B3glct	246	26285	36781	8592
B3gnt2	13245	12961.66667	10798	12625
B3gnt3	14911	15003	22152	19286
B3gnt4	27211	34202.5	39249	37663.5
B3gnt5	28840.5	23831	28205.5	21606
B3gnt7	15354	16159	13055	16603
B3gnt8	18845	15571	11170	9078

B3gnt9	37348	38909	32660	34809
B3gntl1	12883.5	8787.5	6572.5	27422
B430007K19Rik	29354	31363	31072	29671
B430105A11Rik	26747	28075	29690	28425
B430209F14Rik	23230	24314	26158	25127
B430217B02Rik	33792	36093	91	6571
B430306N03Rik	19184	17405	16779	13614
B430316J06Rik	32148	33766	12676	33514
B430319H21Rik	19179	22421	25185	26512
B4galnt1	17224	14844	19536.25	16373.75
B4galnt2	20627	27973	27454	38871
B4galnt3	15718.5	15481.5	14428	20505.5
B4galnt4	614	8196	28701	12467
B4galt1	10190.66667	18215.33333	9901.333333	18860.33333
B4galt2	31404	27505	17666	15220.5
B4galt3	15788	12965	14859	15503
B4galt4	36412	26717	37117	28765
B4galt5	8658	5712	8189	4407
B4galt6	13314	8014	10938	9266
B4galt7	11285	16406	13119	14829
B530045E10Rik	6180	13279	15180	12851
B630005N14Rik	8420	3917	5660	2793
B630006K09Rik	23697	24777	26583	25623
B630019A10Rik	26897.5	25062.5	29024.5	22954
B630019K06Rik	18747	16497	16418	12986
B830004H01Rik	31574	30131	31550	30302
B830007D08Rik	34557	2254	33078	3841
B830012L14Rik	30735	36247	33592	32070
B830017H08Rik	4527.5	4938	9522.5	7648
B930025P03Rik	31167	35102	36255	39283
B930032E21Rik	30804	32205	33521	32011
B930041F14Rik	2345	38768	38222	33517
B930042K01Rik	4649	1546	37157	26872
B930046C15Rik	533	20433	33341	4326
B930049G02Rik	32839	34346	37353	40811
B930054O08	34931	27833	10837	38236
B930063P07Rik	28493	29712	31370	31011.5
B930068K11Rik	7057	29289	6054	6272
B930069K15Rik	11251	615	4899	6155

B930086A06Rik	27786	29074.5	35618	884.5
B930095G15Rik	25273	27545	35631	36495
B930095M22Rik	6425	40575	9773	8478
B930096F20Rik	21729	17820	20178	19136
B930096M23Rik	30893	26191	25093	29407
B9d1	16615	25698	20661	22581
B9d2	36556	6085	886	5707
Baalc	30657.66667	32365	29504.66667	37180.66667
Baat	29558	31306.5	35409	30888
Babam1	25080.5	25216	26071	26121.5
Bace1	26251	20841	20291	19427
Bace2	17383	32392	23848	4419.5
Bach1	19438	5821	40493	26567
Bach2	21779.85714	25178.28571	19998.14286	28659.28571
Bad	20587	20544	23649	22069.5
Bag1	951	2617	40963	1089
Bag2	11354	14642	16726	18455
Bag3	20000.5	27377.5	15433.5	31664.5
Bag4	23181.66667	14218.33333	20238	20373.33333
Bag5	35076	35702	39109	36430
Bag6	8604.5	27330.75	17783	27027.25
Bahcc1	31707	25290	38872	30204
Bahd1	25883	23337	24917	20775
Baiap2	8168	25123.5	9876.5	9054
Baiap2l1	25786.66667	10472.66667	24266.66667	23226.66667
Baiap2l2	10312	7604	1279	13225
Baiap3	39266	39761	873	34674
Bak1	32942.5	33569.5	36467.5	37359.5
Bambi	20254	16903	20044.5	33693.5
Banf1	4295	12019	12647	15853
Banf2	579	4150	10679	9457
Bank1	18011.33333	33697	14203	30686
Banp	15938	19938	15497	20209
Bap1	29345.5	22918	21275	21721.5
Bard1	31494	33086	34026	37022
Barhl1	34182	22146.5	20816	25244
Barhl2	18084.5	22970.5	22110	21960.5
Barx1	9228	11195	850	11299
Barx2	13014.33333	17668	26250	25466

Basp1	11841	10926	8745	8740
Batf	14810	21733	15196	19701
Batf2	1368	11445	5964	15392
Batf3	24473	17600	19603	7903
Bax	9293	15751	13122	15747
Baz1a	9760.333333	22043.33333	19932.33333	20433
Baz1b	10815.5	27253	21321.5	21052
Baz2a	12335.83333	17534.66667	15638.5	13765.66667
Baz2b	13565	10145	24168.33333	22316.33333
BB009967	16670	22042	20691	24009
BB014433	30623	32571	33315	31682
BB016812	17998	21384	28259	30076
BB031773	23442	37016	27771	29455
BB064041	38938	948	1798	38839
BB078705	33282	35437	1307	1609
BB080160	290	36775	5108	7720
BB085087	3508	38177	38791	35453
BB096820	17685	18141	22511	21102
BB111701	12946	9611	10477	7942
BB124324	35106	32408	3611	2679
BB127498	37201	39242	4775	4549
BB144693	5102	2193	1744	735
BB169534	27598	28882	30733	29428
BB182164	26546	29322	25229	25427
BB201250	14918	22896	26703	33273
BB206048	10367	11737	9465	15137
BB230127	30117	30407	27732	28063
BB251370	20199	19233	19386	21558
BB282404	9206	26835	31420	24289
BB283564	6763	1693	1487	37149
BB298822	17906	19950	18126	17287
BB311275	25242	19805	22157	21466
BB345768	35134	4999	728	1400
BB393209	37860	21986	31189	24313
BB431852	33118	35064	36239	34200
BB432378	4166	10058	1515	11326
BB462381	18798	19875	26468	25023
BB532938	25850	27015	29105	28564
BB536409	14769	14478	15846	13937

BB618745	7958	10522	7291	7188
BB674559	3031	4200	2229	3264
BB680855	3549	39002	40304	34484
BB697153	6313	2250	4850	37226
BB728650	34736	36420.5	21019.5	18477.5
BB822924	13479	8761	13724	8059
Bbc3	40355	40430	5434	2904
Bbip1	23897.5	22046.5	16390	20900
Bbox1	36987	5790	21157	22778
Bbs1	14381	8990	14328	11452
Bbs10	16422.5	12382	15933	13729.5
Bbs12	9266	357	4683	981
Bbs2	24812	18650	18073	17206
Bbs4	10877.5	9950.5	8477.5	11905
Bbs5	11412.5	16411.5	14933	16612.5
Bbs7	34450	22607.66667	34101.33333	22740.66667
Bbs9	21728.5	20040	22269.5	21788
Bbx	23017	14643	17045.33333	13733
BC002059	17608.5	11303.5	13768	14837.5
BC002118	29906	31446	32450	31055
BC002189	36343	2180	34546	33483
BC003331	9263	6068	3431	5641
BC003965	5913	11791	15280	15509
BC004004	12929.5	11481	13028	13949.5
BC004015	24947	15695	13604	11848
BC005537	18035	22052.66667	16000.66667	9503
BC005624	39295	40860	2073	579
BC005705	24218	25280	8311	12525
BC006028	35776	38282	39848	771
BC006965	23736.5	21682	24307.5	35851
BC010981	613	38156	34600	36678
BC012698	36241	32703	40799	37126
BC013561	19562	40532	20109	4398
BC016201	12947	14826	16147	11416
BC016548	887	33381	14408	8904
BC016579	30926	32588	34487	32699
BC016608	32548	17283	36221	3633
BC017158	33392	32115	25209	22638
BC017643	14030	16307	38276	17282

BC020150	34674.5	21764	36323	19750
BC021394	2217	1818	34610	2535
BC021614	14754	12581	17753	15475
BC021767	36697	8846	25058	40561
BC021785	31264	13395	14679	15740
BC021831	7121	4	20697	18543
BC021891	10289.5	11838	14506.5	15207.5
BC022654	13158	10396	10868	11165
BC022687	40634	4273	4580	4575
BC022713	34326	38676	9441	2656
BC022771	10703	1996	3995	39853
BC022960	37954	33924	4103	33379
BC023004	16528	17273	24405	20970
BC023105	33105	35039	321	7838
BC023202	13377	14167	19840	20807
BC023719	3952	38814	36105	3096
BC023829	16661	17452	18322	18262
BC024063	2480	2203	20628	18185.5
BC024137	3514	62	1301	40919
BC024139	23121	23822	39748	38621
BC025446	30396	32499	39401	40591
BC026585	34572	34624	23347	31648
BC026762	15558	25175	15195	21706
BC026852	4203	5423	3305	4074
BC027072	38755	38621	6038	6697
BC027183	1822	7239	9873	9755
BC027231	7929	7031	3115	9204
BC027278	15289	19150	20561	20319
BC028528	13248	13810	16123	12451
BC028660	29654	35907	584	4229
BC028799	19794	17876.5	11532	12027
BC028808	4977	6144	8439	9769
BC029127	26107.75	22972	20630	22941.25
BC029214	16479	17566	17897	15763
BC030307	26303	24462	20213	20785.66667
BC030336	16206	11320	8826	5172
BC030500	28659	30113	31909	30121
BC030867	5323	8200	10879	9920
BC031181	22947	22372	19985	23847

BC033599	10232	11528	16734	14143
BC034090	27216	28598	30386	28957
BC034902	25493	25920	27947	34339
BC034904	4990	12701	17493	14801
BC035954	6207	38887	1840	39204
BC037034	19410	15380	20189	19816
BC037438	38181	32984	33218	31568
BC039142	22120	23646	22751	25129
BC040756	23953	37263	5545	10102
BC043934	5200	4827	38796	36046
BC048403	10667.5	17050	14671.5	23641
BC048502	3133	3609	7170	5753
BC048507	23428.5	8580	23280.5	25705.5
BC048546	29270	30908	36203	40969
BC048562	30344	1452	9135	1363
BC048671	7810	12516	15565	17817
BC048679	11004	9459	8753	39716
BC049702	31120	32836	34267	32961
BC049715	41005	35138	37104	36949
BC049730	28467	30232	33398	29350
BC049762	24845	25158	33440	37289
BC051019	36266.5	31674.5	29371.5	36273.5
BC051076	27530	28920	30316	29041
BC051142	37658	26934	35309	33522
BC051435	11180	18721	20688	16851
BC051628	40315	4247	10753	11852
BC051665	35332	28215	2871	34678
BC052040	8393	6097	6102	3155
BC052486	913	41091	6020	11858
BC053393	40015	32799	29807	28400
BC054101	23556	26357	28987	30305
BC055004	6794	22744	40801	19424
BC055324	39555	30719	36544	29951
BC055401	34438	36139	38497	38074
BC061212	37675	12016	1165	651
BC061237	31995.5	3305	39789	16743
BC064078	30338	16287	19764	7420
BC065397	20161.5	36940.5	32790	19146.5
BC067036	13029	10656	7588	10367

BC067074	26384	28040	36807	40386
BC068281	22652	19127	16569	11407
BC071254	40892	34005	29739	27262
BC085283	1802	4823	1501	5232
BC089491	9078	13662	14290	15900
BC089597	36072	33499	40407	32232
BC089618	33930	25932	39466	36441
BC094578	34247	23595	28306	18498
BC096458	26189	27932	31117	26913
BC100451	12184	10158	15956	10210
BC107364	9968	8611	15396	20228
BC146447.1	27971	29297	31984	30479
BC148665.1	22568	23871	34370	36988
Bcam	3253	12409	5675	9221
Bcan	14262	17488	21093	20602
Bcap29	21837.5	22829.5	17987.5	19031.5
Bcap31	30097.5	29002.5	13000	13520.5
Bcar1	34875	40919	4492	9985
Bcar3	4449	26213	199	23404
Bcas1	10505	16785	17978.5	17225.5
Bcas2	24664	26574	2212.333333	27547.66667
Bcas3	27341.33333	26305	24290	27237.33333
Bcat1	15110	14168.5	12246.5	9991.5
Bcat2	14954	19827	18837	22536
Bccip	9098	13126	10886	10977
Bcdin3d	22513	21994	20507	19626
Bche	32091	34248	35685	36915.5
Bckdha	13769.5	30628	26754.5	28753.5
Bckdhb	3758	8531	14487	18930
Bckdk	19308	16670	13000	13399
Bcl10	9031.5	14401.5	11095	13645.5
Bcl11a	7776.666667	24354.33333	26676.66667	17152.33333
Bcl11b	14568	15702	23530	27688.5
Bcl2	22565.5	13443.75	30800	14014.5
Bcl2a1b	33871	40624	29283	404
Bcl2a1c	33519	4242	31487	1989
Bcl2l1	17949.33333	14198.66667	17228.66667	12592
Bcl2l10	33354	8511	2399	12062
Bcl2l11	19418	14485.5	11446	6628

Bcl2l12	6200	5328	21507	17706.5
Bcl2l13	12374	12171	9402	10824
Bcl2l14	29371.5	29908	11355.5	33515.5
Bcl2l15	9346	6420	15241	17891
Bcl2l2	18449.5	19062	19899	19314
Bcl3	27726	40750	30052	4757
Bcl6	8019	695	15011	7923
Bcl6b	12460	9387	11876	8146
Bcl7a	12801	12873	16710	18081
Bcl7b	2220	2933	789	847
Bcl7c	20529.25	22320.25	21969.5	20479.25
Bcl9	22269.66667	19319.33333	17123.33333	15237.66667
Bcl9l	6290	9911	9007	10750
Bclaf1	22019.4	28701.4	23330.6	23960.4
Bco1	30087	31649	33358	39333
Bco2	20397	20820	23607	22659
Bcor	12499	11380.33333	13786.33333	13722.66667
Bcorl1	26603.5	27401	29210	27623
Bcr	22427	13097.25	23041.25	17155.25
Bcs1l	4374	6358	1983	3030
Bdh1	17850.5	20638	21605	21870
Bdh2	12200.5	14688	20197	21706
Bdkrb1	21473	23481	30100	32679
Bdkrb2	3813	8855	11512	14274
Bdnf	15634.5	16342	18184	19288.5
Bdp1	18782	14659.5	11551	13591.5
BE368005	20143	10478	20645	19494
BE371292	32210	34008	40626	5924
BE447944	26621	27911	30151	28770
BE646931	953	1899	2329	2071
BE651380	37928	32535	36286	34471
BE686687	36839	34463	11582	37041
BE687029	4718	18184	5596	6976
BE945021	32205	31545	2462	31046
BE955693	4194	6581	15249	14887
BE956710	13419	17256	22339	22303
BE980334	16279	16648	22420	20455
BE981005	37732	30387	37269	39091
BE981145	40972	8896	3198	11451

BE987854	40018	38470	35137	1002
Bean1	32774	12671	34977.5	35053.5
Becn1	34727.33333	35631.66667	35991	32086
Bend3	4340	21991	25980.5	26593.5
Bend4	4682	39216	225	31842
Bend5	10405	12089	38914	6541
Bend6	21880.5	26993	23187.5	27695
Bend7	29503.5	31116	33477	30928.5
Best1	9508	26120.5	10369.5	24187.5
Best2	25918	28448	28171	27358
Best3	23309	1133	28190	25203
Bet1	19290	23783	19279	21182.5
Bet1l	13408.33333	13597.66667	25827	15313
Bex1	25089	20232	31546	20273
Bex2	2938	13520	11106	18566
Bex4	12426	14626	17772	18439
BF021831	35675	31220	28507	31197
BF149076	12832	14505	22066	19003
BF166728	8547	3202	13842	8176
BF167521	5850	8456	6423	4894
BF464414	11139	14621	14894	15320
BF682133	9374	9392	15769	13380
Bfar	25325.33333	21100.66667	20346	21748
Bfsp1	36524	25238	24680	16670
Bfsp2	13294.66667	15778.66667	16658.66667	18028.66667
BG067131	34072	39774	5377	4204
BG068695	6017	7694	8106	11832
BG068807	11207	9406	6363	8615
BG079886	20424	16424	15436	12157
BG080473	4352	6825	33225	213
BG294923	14864	15327	20727	24238
BG294955	5526	8944	12351	12184
BG806432	24637	29081	39414	38557
Bglap	34334	36345	2561	6935
Bglap3	40915	2082	7149	8079
Bgn	32821	33901.5	32606	35022.5
Bhlha15	5046	7874	7404.5	8683
Bhlha9	40241	39714	40343	39932
Bhlhb9	6588	1068	5920	36097

Bhlhe22	18902	17000.5	22756.5	24535.5
Bhlhe23	29897	31462	33906	33556
Bhlhe40	12950	8681	12966	10211
Bhlhe41	37577.5	5029	35225	3702
Bhmt	34220	36511	5352	38180
Bhmt2	31861	33067	1013	5112
BI100969	30808	25597	10757	13442
BI151587	28941	36181	4466	531
BI247908	10138	11019	17748	20643
BI662359	19728	15184	17383	17422
BI694651	3798	11448	6645	12036
BI697602	18400	20553	12450	27085
BI732528	24465	25800	38649	26361
Bicc1	12881.66667	10446.33333	17238.66667	20474
Bicd1	32994	30222.5	35033	34215
Bicd2	16592	13368.5	30197.5	30106
Bid	20390	24580	19363	24469
Bik	20841	19161	18360	12022
Bin1	32834.66667	34702.66667	21916	36204.33333
Bin3	23690	19215.5	19437.5	17754.5
Birc2	7375	8516	13803	14083
Birc3	12509	9838.5	10228	10693
Birc5	38244	39719	1140	1929
Birc6	22762.33333	23601	25513.33333	25859.66667
Birc7	3218	6213	15931	15956
Bivm	16748.5	21310.5	19950	37159
Bj2	41068	8279	33161	3503
Blcap	11687.33333	12947.66667	11606	18205.33333
Blk	37146	34828	40257	39720
Blm	35224	38558	37409	36878
Blmh	16670	18819	16861.5	21040.5
Blnk	2090	29166	889	35748
Bloc1s1	20210.5	27920	10924.5	29103.5
Bloc1s2	18470	22345	21172	22598
Bloc1s3	5138	9072	7298	6218
Bloc1s4	2935	4404	1756	2948
Bloc1s5	22734.5	22663	21403	23182.5
Bloc1s6	16424	17848	15506	15767
Blvra	8985	18184	9585	6219

Blvrb	4313	15911	9531	18775
Blzf1	26742	25660	23704.66667	25087.66667
BM932442	8363	4537	7253	5634
BM939341	21851	11781	12817	8846
BM939644	21138	12726	23723	24024
Bmf	10414.33333	12048	13025.33333	13457.33333
Bmi1	22118.5	22156.5	18448	20427
Bmp1	3405.5	4851.5	7793	10991.5
Bmp10	15307	14539	16266	14188
Bmp15	24030	25234	27245	25829
Bmp2	11857	10915	13654	9220
Bmp2k	22928	18802	17584.33333	15327.66667
Bmp3	11436	11431	16808	16764
Bmp4	26077	23896	32040	30798
Bmp5	27804	25056.5	28635.75	26860
Bmp6	11784.5	30068.5	19843	26848.5
Bmp7	28844	25612	13336.5	12470.5
Bmp8a	8688	4457	6460	12623
Bmp8b	28863	30562	30549	37011
Bmper	18673.5	19393.5	11799	14435
Bmpr1a	7177	2756.5	15353.5	17882.5
Bmpr1b	31355	30888	31741	30385
Bmpr2	16917	19099	22685	18333.33333
Bms1	20304	16899	16546	24249
Bmx	2065	34621	923	1564
Bmyc	31549.33333	29619.33333	20693	28843
Bnc1	4958	32426	3370	32846
Bnc2	31679.33333	11726.33333	23102.33333	17262.33333
Bnip1	12820.33333	13655	13821.33333	15762
Bnip2	28251	29569.5	27982	28809
Bnip3	25372	34858	36517	2890
Bnip3l	10998.5	30458.5	14905.5	12921
Bnipl	3721	17462	21187.5	923.5
Boc	10304	15112.66667	19822.33333	9333.333333
Bod1	36275	31455	38711	37878
Bok	8266	10126	13770	17611
Bola1	38943	3211	3114	1864
Bola2	11975	19032	17958	19426
Bola3	3096	9458	10989	10287

Boll	34313	16120	7963.5	22037
Bop1	21968	28034	23700	30205
Bora	10061	17139.33333	21221.66667	21575.66667
Borcs5	12731	9980	3839	5802
Borcs6	28388.5	26659	23402	17125
Borcs7	21016	24578.66667	18438.66667	20657.33333
Borcs8	38314	38639	38075	37292
Bpgm	20907.5	20865	20031	19434.5
Bphl	29043	27733	26208	25180
Bpi	5636	3219	34276	11395
Bpifa1	23879	35308	26843	30670
Bpifa2	2597	39957	6008	2921
Bpifa3	27831	37306	30650	29222
Bpifa5	15996	19358	24151	24522
Bpifb1	19951	23025	32406	30436
Bpifb2	18407	16229	20158	12983
Bpifb3	38101	1338	40459	5007
Bpifb4	31997	34411	35670	33548
Bpifb5	17210	17387	24256	25797
Bpifb6	6385	11820	12071	15066
Bpifb9b	29045	29355.5	32957	36341
Bpifc	23449	9893	27302	6810
Bpnt1	38517.33333	9816.666667	10643.33333	13475.66667
Bptf	15871.75	19486.25	21238	19534
BQ033444	4847	6583	14342	15096
BQ042196	21906	23173	17447	22575
BQ044049	29691	31724	32501	30092
Braf	20132.25	16976	16577.5	18456.125
Brap	6550.5	6120	4473	4103.5
Brat1	12109	10004	10178	7506
Brca1	24506	24688.5	20564	7771.5
Brca2	17071.5	14252	13097	8435
Brcc3	21736.66667	24258.33333	25311.66667	26823.33333
Brd1	21347	24036	19413.5	24346.5
Brd2	22444	21758	29708	26759
Brd3	15888	14661.33333	12392	11435.66667
Brd4	20160.5	16354.5	17435	15104.5
Brd7	14576.5	16238	17618.5	19337.5
Brd8	11322.6	13310.4	12451.6	13424.4

Brd9	13878	20420	13290	17901
Brdt	23720	17368	22103	18338
Bre	21549	22453	21229	25782
Brf1	22361.5	15774.5	11785.5	10119
Brf2	18646	14987	16636.5	19179.5
Bri3	30298.5	22702	21213.5	19445.5
Bri3bp	13671.6	18608.4	15423.6	11931.2
Bricd5	762	32220	8800	9502
Brinp1	31706.5	14905	18456.5	17688
Brinp2	16481	18864.5	20391	19394.5
Brinp3	32596	34185	36622	34699
Brip1	25497.66667	21773	25700	25372
Brix1	31403.5	38142	16284.5	20932.5
Brk1	19591	13823.5	15252.5	14589.5
Brms1	23576.5	23095.5	17970.5	20338.5
Brms1l	13925	23001	15200	19821.5
Brox	25880	20321	21817	21839
Brpf1	38422	35393	39382	39788
Brpf3	11699.5	8276	1261.5	21213
Brs3	4124	6086	29706	28350
Brsk2	33988.5	1974.5	6098.5	5067.5
Brwd1	24126	30022.14286	22596.57143	24432.14286
Brwd3	25614.5	17928	17230.5	18032.75
Bscl2	20318.5	14871.5	11686.5	12460
Bsdc1	26296	22105	23917	25447
Bsg	21380	24803	22112	24907
Bsn	15803.5	13573	15740	31429.5
Bsnd	8048	11106	17774	18175
Bspry	13784	12034	15595	14404
Bst1	38636	16735	1631	20451
Bst2	13585	40806	21216	4084
Bsx	31814	1711	2309	7950
Btaf1	14688	8071	9373	9239
Btbd1	13839.5	31427	32883	30852.5
Btbd10	26061.5	17008.25	16848.25	14603.25
Btbd11	26683.5	28150.5	29190.5	27967.5
Btbd16	28881	30508	31211	29845
Btbd17	16444	8533	8068	38859
Btbd19	6185	13479	13610	17649

Btbd2	15992	14133	14702	12241
Btbd3	19570.5	14032	14039	13080
Btbd6	37911	35746	35881	33605
Btbd7	19747	16610.75	10132.25	9235.25
Btbd9	25129.2	21290.4	11885.6	20226.6
Btc	15279.5	15709.5	33569	31750
Btd	9682	11109	5455	7719
Btf3	19270.5	23712	26718	23311.5
Btf3l4	24169	24761	21349	20116
Btg1	23107.5	19808.5	27822	2286.5
Btg2	25002	17873	19260.33333	17464.33333
Btg3	26977	39883	31256	41000
Btg4	24849	25968	28253	27027
Btk	22743	19179	27717	27558
Btla	28944	24239	26430	40340
Btn1a1	25039.5	26083	28209.5	33228
Btn2a2	25284	26535	34851	30474
Btnl1	13668.5	13945	16564	16023.5
Btnl10	17154	14357	21537	22966
Btnl7	23799	24937	26610	25498
Btnl9	6283.5	25163	25651	29077.5
Btrc	15236	13899.5	11805.5	13494
BU531427	3329	38102	2350	31445
BU534286	151	41086	5767	4972
BU554808	27614	12864	25844	11793
BU592067	25293	22939	35725	30926
BU605312	3054	32948	5290	35181
BU937187	39482	5093	4938	6822
BU961420	5609	40027	33111	40504
BU962533	20124	24119	34826	36706
Bub1	29376	25487	24526	26720
Bub1b	28738	7716	23664	1512
Bub3	13439.5	22819	13544	18892.25
Bud13	16741.5	18085.5	20127.5	18311.5
Bud31	25796	18313.66667	28676.33333	18152
Bves	24675	25648	36238	30409
BY372015	15416	17564	19036	19347
BY426437	23134	25170	34467	28553
BY557806	964	36706	8814	5743

BY713104	13748	13776	29548	28264
BY734332	28138	29579	30681	34602
BY765165	16100	23974	4483	12945
Bysl	26017.33333	17929.33333	15306.33333	20102
Bzrap1	34762	29129	2134	34785
Bzw1	19518.25	15211.25	11802.5	13560
Bzw2	13899.33333	32373.66667	24842.66667	32039
C030005H24Rik	11225	5292	17188	11619
C030005K06Rik	31709	32865	40408	34669
C030006F08Rik	36629	37793	9529	9422
C030007I01Rik	32640	34334	35403	33588
C030007I09Rik	7751	7832	9238	5649
C030011G24Rik	29760	31210	32473	31017
C030014I23Rik	6442	8933	11245	11679
C030014L02	25644	32858	29191	27917
C030014O09Rik	11218	7588	16918	12424
C030015A19Rik	9623	2663	4436	1181
C030015D19Rik	1360	33030	38063	34419
C030016D13Rik	31583	36222	1689	32283
C030017B01Rik	30682	32237	33893	32240
C030017G13Rik	26679	27885	30695	29259
C030027H14Rik	12201	23633	15718	30011
C030032O16Rik	32191	24918	32486	31028
C030034I22Rik	19977	22024	22758	22686
C030038I04Rik	39526	26495	28052	27005
C030039E19Rik	27250	28857	29417	28193
C030039L03Rik	23730	22394	19145	24527
C030040A22Rik	29000	25534	27556.5	26469
C030041B07Rik	39841	37037	3291	36771
C030046M01Rik	31975	33832	35102	33301
C030047K22Rik	31862	33510	3113	5583
C130006E23	36757	20013	22025	14844
C130021H21Rik	12736	5027	6652	6754
C130021I20Rik	29752	31297	32624	31555
C130023O10Rik	26777	28009	29937	28667
C130024J02Rik	24615	25988	27013	25936
C130026I21Rik	18599.5	4146	7264	5314
C130026L21Rik	27875.5	26391.5	27636	26942.5
C130030J05	1958	33097	38190	5885

C130030K03Rik	24345	25469	27598	26402
C130036L24Rik	2292	34315	40027	39182
C130050O18Rik	28691	31578	25811	34642
C130051F05Rik	14798	31606	16293	12138
C130051G18Rik	7274	3968	27411	26476
C130057D09Rik	23367	24419	26496	27001
C130057N11Rik	275	37440	36287	36769
C130060K24Rik	28980	30903	16012	35657.5
C130070B15Rik	15733	17678	18622	17996
C130071C03Rik	25741.66667	18487	28473	27994.33333
C130073F10Rik	33058	35030	35773	33873
C130074G19Rik	34256	32512	34136	32538
C130076G22Rik	14977	35889	2020	13724
C130078N14	3661	28788	32728	1502
C130079B09Rik	38445	30261	26069	35951
C130079G13Rik	34173	39144	11788	10850
C130081A10Rik	7154	13658	10809	16396
C130083B21Rik	8289.5	12610.5	26925	26870.5
C130083M11Rik	25890	39710	30501	29076
C130098B18Rik	5678	31547	24882	22034
C14orf39	21079.25	22458.75	25948	25942
C1d	15331	20217	13582	16350.5
C1galt1	21138	32806	30688	18320.66667
C1galt1c1	35840	36700	30358	35009
C1qa	11742	6519	21818	16020
C1qb	16134	37498	15000	34953
C1qbp	12886	13632	13528	13750
C1qc	33842	10790	28504	12291
C1ql1	1113	40817	31590	38313
C1ql2	14156	13474	22003	27885
C1ql3	29133.5	11560.5	17559.5	15795
C1qtnf1	4160	3954	10089	10772
C1qtnf2	6162	3366	34926	7440
C1qtnf3	16239.5	33578	20351	16128.5
C1qtnf4	23378.5	26476	18955.5	18728.5
C1qtnf5	27490	33868.5	37150.5	19769
C1qtnf6	35803	39960	1866	5385
C1qtnf7	29872	36673	39962	10738
C1qtnf9	34830.5	19279.5	6183	2915.5

C1ra	8978	8158	8873.5	26205
C1rl	14683	26153	16838	22959
C1s1	8259.5	18909.5	13477	27765.5
C2	20225	11670	9537.5	21245
C230004F18Rik	29904.5	17027.5	34391.5	32010.5
C230012O17Rik	31243	18845.5	33300.5	18746
C230013L11Rik	27402	8033	2393	9520
C230014O12Rik	37534	32456	33217	31651
C230029D21Rik	25865	17643.5	20838.5	18156.5
C230029F24Rik	26838	37827	2954	18
C230030N03Rik	32251	37273	35580	17
C230035I16Rik	7086	5085	7559	11602
C230047L17Rik	31289	33018	34526	33148
C230057H02Rik	31971	34455	34364	32611
C230060E24	26514	28127	29263	28563
C230066G23Rik	31545	33978	34531	32615
C230072F16Rik	25731	27810	40093	2570
C230075M21Rik	11141	773	5035	5974
C230083P08Rik	10803	5345	4542	9982
C230086J09Rik	32503	35773	36631	38523
C230088H06Rik	20471.5	22180	35967	15912.5
C230094B09Rik	24127	10386	11790	13542
C230096K16Rik	15703	13761	9406	9168
C230098O21Rik	9361	11812	23797	20964
C2cd2	21825.66667	30134	29429.66667	30234.66667
C2cd2l	13677	10914	10250	7849
C2cd3	22064	22805.33333	28234.33333	28040.66667
C2cd4a	13458	15781	19562	19967
C2cd4b	11532	23355	15205	15108
C2cd4c	16648	16685	21803	22390
C2cd5	13734	10750	14367	11462
C3	27018.66667	18639.66667	22834	21253.33333
C330006D17Rik	40418	38480	5072	5253
C330006P03Rik	38790	24696	34318	39352
C330007P06Rik	25444.66667	28709.66667	24644.66667	30542.33333
C330011M18Rik	15548	12637	19372	15420
C330013J21Rik	15931	19890	11724	6275
C330018A13Rik	31087	32367	2688	2082
C330018D20Rik	10577	13586.5	13366	14460.5

C330020G15Rik	12459	6778	9636	2489
C330024D21Rik	26695	28112.5	32990.5	34930
C330026H20Rik	36373	19090	16039	15175
C330026N13Rik	23466	24548	26423	25417
C330027C09Rik	33072	32501	11124.5	30571.5
C330046G03Rik	18982	19440	32797	31379
C3ar1	11782	24155	21636.5	26834
C430002N11Rik	40993	8939	1020	38112
C430019N01Rik	12373	31667	4248	25849
C430039J01Rik	25027	26305	28050	26993
C430049B03Rik	18243	20625	26861	24885
C4a	40851	35475	13666	13984
C4b	22651	14329	37493	24623
C4bp	37035	37445	2307.5	4711.5
C530005A16Rik	39301	685	34438	32683
C530007A02Rik	5731	141	2432	40544
C530008M17Rik	32390.8	27643.8	22465.2	21116.2
C530014P21Rik	17098	21097	27274.5	29429
C530025M09Rik	31164	32774	34389	32653
C530025M11Rik	188	29023	30614	29542
C530030A11Rik	36939	39263	2577	40475
C530030K21Rik	2711	40145	10229	28344
C530030P08Rik	2811	31514	31980	30686
C530036F05Rik	11280	13299	16240	16819
C530038A18Rik	31286	32988	34570	32738
C530042K13Rik	29934	31929	36104	30336
C530043A13Rik	40230	3101	7955	11052
C530043K16Rik	15761	9217	17062	17063
C530047H08Rik	1667	4089	9374	14619
C5ar1	23630	19820	32949	19324
C5ar2	12606	21609	11894	17198
C6	37947	39451	38511	38021
C630004L07Rik	4467	7136	11088	11178
C630043F03Rik	22560	24146	20475	21923
C630050I24Rik	11519	18696	17362	22445
C7	30424	32533	32408	30814
C730002L08Rik	28216	29736	31153	29914
C730014E05Rik	18348	32265	27751	28327
C730027H18Rik	14959	20541	16306	16755

C730034F03Rik	20984.5	26947.5	31178.5	10776.5
C730036E19Rik	23740	24894	26380	25390
C730045O03Rik	19395	14455	21209	22085
C730049O14Rik	5506	1090	11071	40067
C77080	9791	2182	10334	2740
C77370	25394	27207.33333	24516.66667	27335
C78859	27948	29546	30314	29014
C87414	24173	4992	828	19462
C87436	17906.66667	28903	28442	26625
C87499	14677	13745	27498	29571
C87977	28408	25536	9524	6027
C8a	2254	3627	4898	1426
C8b	34924	29375	32333	1911
C8g	5812	5692	7029	3552
C9	40197	29716	28995	37720
C920006O11Rik	182	1242	40783	1987
C920008G01Rik	35116	5287	9250	9439
C920008N22Rik	10459	658	38500	37162
C920009B18Rik	8086	15778	15155	19994
C920021A13	2103	11658	9520	16695
C920021L13Rik	19473.5	21535.5	23204.5	23675
C920025E04Rik	8548	13490	10434	17478
CA322787	523	3215	395	1932
CA481501	11177	8879	21063	18905
Caap1	26078	25970.66667	12837.33333	14727
Cab39	17427	26516	19521.75	27664
Cab39l	25837	25962	21010	26255.33333
Cabin1	26345	21653.33333	19168	19100
Cables1	25428	22997	29849.5	23267.5
Cables2	6648.5	11175.5	2681.5	10943.5
Cabp1	9229	15956	10326	16565
Cabp2	22629	21760	27381	26776
Cabp4	39104	26828	37728	29435
Cabp5	32902	34831	1249	34306
Cabp7	37475	7624	1741	38867
Cabs1	20299.5	14925	36835.5	6026.5
Cabyr	8909	7228	5317	11653
Cacfd1	11686	15083.33333	12561	13627.33333
Cachd1	31185.25	28511.25	27909	26701

Cacna1a	21918.5	21998	25689	23838
Cacna1b	16389	17287.5	14644.5	19397.5
Cacna1c	21627	21965.33333	10626	13689.33333
Cacna1d	20999	22732.75	18273.25	23986.5
Cacna1e	29611	30910	32737.33333	20115
Cacna1f	8218	11313	9512	11626
Cacna1g	21338.5	22700	18791.5	21670.5
Cacna1h	30665	28839.5	34644.75	36510.5
Cacna1i	13010	12938	20898	18396
Cacna1s	2443.5	6108.5	14017.5	14875.5
Cacna2d1	18225.75	19172.75	16714.5	14114.5
Cacna2d2	17892.25	21118.25	15733.5	24479.5
Cacna2d3	35234	33637	27608	26437
Cacna2d4	26404	27506	30727	29282
Cacnb1	16383	18996	16903	18895
Cacnb2	6602	5407.333333	6396.666667	7345.333333
Cacnb3	27798	36582	30600	574
Cacnb4	16448.14286	22834.28571	19719.28571	19363
Cacng1	36568	483	31434	3304
Cacng2	28108.5	21141.25	11698.75	12592
Cacng3	1117	3521	7293	5870
Cacng4	15429	17236	20288	22779
Cacng5	14566	12312	28627	22429
Cacng6	9955	11420	16624	15601
Cacng7	13050	15068	19002	18554
Cacng8	24396	24196	36628	34271
Cactin	17241.5	16166.5	19667	18340.5
Cacul1	16088.5	16409.66667	12759.83333	20113.16667
Cacybp	13959	20199	17049	21074
Cad	14233	16019	15319.75	14782.75
Cadm1	13321.66667	23901	21911.33333	15220
Cadm2	23729.2	25995.6	30032.2	27053.2
Cadm3	21983.66667	22831.33333	26524	28304.33333
Cadm4	40809	2095	8218	10359
Cadps	6883.75	19157.75	16578.75	20670.75
Cadps2	24353.4	31913.8	22263.2	15426.4
Cage1	11111	40717	31010	864
Calb1	36610	8996	1913	40140
Calb2	24863.66667	23287.33333	27169.33333	27042.66667

Calca	35065.5	20423	8823.5	4364
Calcb	1363	38494	32856	31437
Calcoco1	15270	12389	13664	11444
Calcoco2	21964.5	30025	29149.5	29751.5
Calcr	24209.5	24620.5	25842	29399.5
Calcrl	20008	23533	21242	30174
Cald1	14425	17094.66667	14702	16363.33333
Calhm2	21292	17451.5	21193	16642
Calm1	13933.83333	17343	17669.83333	19563.33333
Calm2	6857	23209	10747	3331.5
Calm4	23170.5	19967.5	24846.5	26340.5
Calm5	3545	4159	38818	26706
Calml3	25697	34503	30216	37322
Calml4	28314	21393	939	26984
Caln1	30001.5	34810.5	20293	20095.5
Calr	20998.5	18950.5	17273	20652.5
Calr3	37562	14040	7222	20866
Calr4	31343	24561	36756	32589
Calu	15049.33333	30333	19366.66667	20327
Caly	17827.5	21225.5	23848.5	26627
Camk1	25083	20183	18114.5	14429.5
Camk1d	27915	17259.33333	27147	24968.33333
Camk1g	23851	27230	29732	31503
Camk2a	33515.5	35568	21686.5	21712.5
Camk2b	15408	9363	26340	19217.5
Camk2d	23353.16667	16394.83333	17716.83333	20618
Camk2g	18017	14712	18538	14950
Camk2n1	15790	16061	14723	21312
Camk2n2	5820	4970	8931	7846
Camk4	21077.4	20839.8	25493.8	24653.4
Camkk1	2263	13297	4587	10646
Camkk2	12325	7668	11244	9847
Camkmt	24536	19863	22134.5	23443
Camkv	21538	20937	26759.5	26601.5
Caml	35048	36201	35836	38452
Camp	25218	10327	1670	6329
Camsap1	26406	23302.5	24841.5	24154.5
Camsap2	22414	19667	10821	10632
Camsap3	23397	23519	26511	28127

Camta1	20464.33333	22935.66667	26075.66667	24410.66667
Camta2	13603.5	18145	12513.5	15698.5
Cand1	5711.333333	5095	5880	18589
Cand2	18366	20458.5	17998	7197
Cant1	14145.5	15916.5	11685.5	13879.5
Canx	29333	28766	31123	33267.33333
Cap1	34740	27083	24627	24838
Cap2	36682	38069.5	20394.5	36598.5
Capg	4535	2136	7482	7180
Capn1	9117	8762	6514	5120
Capn10	10315	10879	7575	5692
Capn11	4908	800	30413	31344
Capn12	26117.5	6674.5	7692.5	10681.5
Capn15	13915	18755	10279	12847.5
Capn2	12726	27006.5	14102.5	29669.5
Capn3	13214	12028	11829.5	26562
Capn5	38687	25475	33464	23987
Capn6	11510.33333	14512	17452.66667	21385.33333
Capn7	27347.5	21984.5	20573.5	20926.5
Capn8	15020.5	18469.5	28725.5	28644.5
Capn9	40986	39732	9180	15069
Capns1	5189	5537	8098	8142
Capns2	6409	16079	33985	9080
Caprin1	16201	8017.5	8441.75	9783.25
Caprin2	16413	13588	14744	12474
Caps2	23578	32725	26417	25319
Capsl	37270	17505	24318	10229
Capza1	25896.5	6959.5	8678.5	9991.5
Capza2	32596.5	25867	21112.5	22065.5
Capza3	37076	27819	6496	39068
Capzb	33584	39723	20937	19487.5
Car1	20006	22223	24362	24742
Car10	25833	25494.66667	24172.33333	34763.33333
Car11	2176	1054	8780	7354
Car12	19550	17353.5	22832.5	9259.5
Car13	22909	40928	31474	3592
Car14	28851	21703	35490	32769
Car15	16038	7169	16129	17078
Car2	6126	10598	8748	9740

Car3	13346	17242	22084.5	20578
Car4	379	7656	388	7445
Car5a	13085	16306	16841	17074
Car5b	27985	25560	27742.5	25763
Car6	20678	24682	40847	37544
Car7	38867	1502	4049	7136
Car8	30739.5	13638.5	31390.5	32975.5
Car9	37441	34565	258	38496
Card10	23915.5	9097	26229.5	8863.5
Card11	19214	31463.5	8115.5	19881
Card14	16545	17517	21485	21510
Card6	6810	1446	2723	40058
Carf	25980.33333	21721.33333	25791	27266.33333
Carhsp1	31769	32970.5	35817.5	17594
Carkd	15813.5	14007	8076	27186.5
Carm1	39074	36142.5	28270	27993
Carnmt1	6075.5	25789.5	24813.5	26345.5
Carns1	24983	24613.5	28371.5	27058.5
Cars	13623	12809	25658	14815.66667
Cars2	12703.5	9462.5	26926	28149
Cartpt	16868.5	20812.5	25113	23708
Casc1	25071.25	22078.75	16084.5	11428
Casc3	22910	22533	25178	23827
Casc4	29793	34014.66667	24528	30164
Casc5	17110.66667	15188.66667	16918.33333	32342.33333
Casd1	15394	12284	16479.66667	13597
Cask	12267.4	16621.6	10837.4	18204.8
Caskin1	14882	37938	35805	4951
Caskin2	8257	12235	14836	18079
Casp1	15094	15896	11908	17942
Casp12	34089	38734	39227	1348
Casp14	12429	15719	19754	18821
Casp2	39715	36073	39897	35174
Casp3	16028.5	14805	14108	17199.5
Casp4	36492	10681	18226	31289
Casp6	38739	40510	1245	36020
Casp7	17118.5	24002	18270.5	24205.5
Casp8	1263	347	3064	37531
Casp8ap2	2404	2021	7130	4587

Casp9	40591	31337	39935	34835
Casq1	33696	37449	29915	28434
Casq2	37340	40060	34476	6983
Casr	9035	4313	17754	18878
Cass4	28641.5	9678	10082.5	8522
Cast	21561.5	18899.5	9807.25	9488
Casz1	28644	20758	25634	22775
Cat	7677	14855	11234	20716
Catsper1	19338.5	20190.5	17884	19102.5
Catsper2	9941	15321	13959	17938
Catsper3	37793	23283	17467	3584.5
Catsper4	1418	37289	3037	30309
Catsperb	24944	30885.33333	26767.66667	26727.66667
Catsperd	25865	27347	3650	34782
Catsperg2	18940.66667	18333.66667	19625.33333	22363
Cav1	429	21677	21651	4724
Cav2	17465	22117.5	21689	28628
Cav3	24757	26136	37943	9698
CB227806	21694	26580	36906	28509
CB525263	34196	29555	40536	29885
CB590298	28584	22118	22992	29187
Cbarp	14727	12547	14116	13572
Cbfa2t2	12594.5	9968	25615	8189
Cbfa2t3	28215.5	9503	18713	26005.5
Cbfb	25842.5	9083.5	9988.5	10099
Cbl	20620.33333	16547.33333	16234	27366
Cblb	23044	31785.5	20208	16661
Cblc	20218	20995.5	24902	22664.5
Cbll1	13867	25345.5	24374	26888.5
Cbln1	1688	4817	6198	10478
Cbln2	16616	8562.666667	24105.33333	28815.66667
Cbln3	30965	34485.5	21217.5	20457.5
Cbln4	4997	1597	9081	11283
Cbr1	29380	38178	37484	38165
Cbr2	17426	40245	19789	38347
Cbr3	2333	21594	9633	22618
Cbr4	16701	18161	16670	18001
Cbs	39576	37542	2633	933
Cbwd1	25171.5	11983	6351.5	13679.5

Cbx1	21381.5	21429.5	21041.5	19364
Cbx2	10071.5	14213.5	17997.5	17316.5
Cbx3	22485	21451.75	21389.75	18444
Cbx4	15917	12692	14453	16071
Cbx5	23193	24645.5	25595.25	16968
Cbx6	19904.33333	17849.66667	25983.66667	23180
Cbx7	18114	10277	16247	11568
Cbx8	18571	17231	19512	16783
CBX8	28094	29972	31382	31123
Cby1	34378	39325	40070	37112
Cby3	9956	10696	31198	29288
Cc2d1a	11544	8650	10920	8591
Cc2d1b	22995.5	18055.5	20254.5	17220.5
Cc2d2a	15799	18847	6957	19594
Ccar1	21832.5	18307.25	18627.75	20984.5
Ccar2	9756.5	10532	12373.5	11345
Ccbe1	31971	19015.5	14734.25	26113.25
Ccbl1	15632.5	16284.5	20079	19634
Ccbl2	3074	6440	40643	1663
Ccdc101	9137	12224	29755	9514.5
Ccdc102a	11248	8720	14542	11617
Ccdc103	7083.5	11204.5	16196.5	15496.5
Ccdc105	33007	29897	33046	34285
Ccdc106	18437	21490	24441	23091
Ccdc107	15355	20325	19107	20014
Ccdc108	22753.5	17366.5	6015	4760
Ccdc109b	16786	15912	14303	15671
Ccdc110	27820	29729	31396	1176
Ccdc112	20802.5	24030.5	23169.5	21771
Ccdc113	39101	37573	31573	38374
Ccdc114	433	40100	9383	12796
Ccdc115	32337	30316	29884	27456
Ccdc116	32830	34986	17981	19825.5
Ccdc117	15703.5	18602	15994	19586
Ccdc12	20313.5	21322	27400.5	24110.5
Ccdc120	18811	20735	21200	25265
Ccdc121	30739	32677	33811	32064
Ccdc122	19870	944	23856	1618
Ccdc124	325	3594	5721	7343

Ccdc125	37321	40048	26606	34239
Ccdc126	24186	28995.5	26100.5	29177.5
Ccdc127	16013.66667	16389.33333	16670.66667	16841.33333
Ccdc129	28623	30137	4414	30515
Ccdc13	37736	37764	7677	14835
Ccdc130	7254	6283	7034	6548
Ccdc132	20760	20980	19836	21607
Ccdc134	2247	829	6245	4385
Ccdc136	6912	1499	40705	10882
Ccdc137	29737	27232	26372	27396
Ccdc138	21435	15535.2	10589.4	19014.8
Ccdc14	26636	25728	26445	27128
Ccdc141	20510.33333	21857.33333	19606.66667	20095.33333
Ccdc142	33582.5	17798	5800	5314
Ccdc144b	26984	28401	30670	29346
Ccdc146	26436.5	27757	29468	28267.5
Ccdc148	17174.5	13570.5	14276	15145
Ccdc15	30732	26585.5	24550	21287.5
Ccdc150	27904.5	25234.5	6687	8507
Ccdc151	20509	19749.5	29401.5	31472.5
Ccdc153	16195	19124	24275	22474
Ccdc155	14327.5	23599.5	23544.5	27060
Ccdc157	7617	18346.33333	25910.33333	15244
Ccdc158	31436	33346	17846.5	33148.5
Ccdc159	13536.5	16607.5	17867.5	17671
Ccdc162	1547	281	8017	7790
Ccdc163	20548	18672	19436	20492
Ccdc166	24604	24879	23575	24456
Ccdc167	20882.33333	25482.66667	23093.33333	24149.33333
Ccdc169	4512	33172.5	15568.5	21970
Ccdc17	17784	19753	14449	16955
Ccdc171	16539	12455.33333	12043.33333	21392.33333
Ccdc172	14902.5	26880.5	31025.5	31954
Ccdc173	23352	22044.5	25566	25712.5
Ccdc174	5409.333333	30288.33333	28087.66667	27026
Ccdc175	31845	33495	35086	33245
Ccdc178	27988.5	29627.5	15942	15452.5
Ccdc18	13069.5	9336	10441	27399.5
Ccdc180	23543	23524	31702	36463

Ccdc181	25538	25343	28200	26643
Ccdc182	4641	13644	12464	11605
Ccdc183	31610	33180	39896	2985
Ccdc184	2899	2072	30344	729
Ccdc185	28385	37196	33426	445
Ccdc186	17943	14092.66667	15941	15496
Ccdc22	6696	2989	4452	2628
Ccdc23	4141	9723	10727	8100
Ccdc25	20558.5	17257.5	12758.5	16965.5
Ccdc27	39389	33478	7169	9385
Ccdc28a	13060.33333	15082.66667	12036.33333	12399.33333
Ccdc28b	13186.5	18522	17290.5	14908.5
Ccdc3	26287	27381	30851	29311
Ccdc30	17811.5	26552.5	19313.25	20489
Ccdc32	19020	17038	19429.5	16540
Ccdc33	19251	13678	19550	19878
Ccdc34	12339	30877	33611.5	32194
Ccdc36	40938	27175	41113	30865
Ccdc37	23510.5	1286	7279	10288
Ccdc38	1439	38912	35125	39301
Ccdc39	35659	33652.5	19956	17433.5
Ccdc40	26577	30516	21919	17105
Ccdc42	31099	33356	32935	845
Ccdc43	26110.5	12935.5	10976.5	15181.5
Ccdc47	17320.66667	18513	20001.33333	18606.66667
Ccdc50	26042.75	21552	22109.5	12062.75
Ccdc51	7013	8810	9407	9563
Ccdc53	21395.5	8480.5	6817.5	6435.5
Ccdc54	2783	581	12230	11039
Ccdc55	25611.8	31989.6	32974.6	33894.2
Ccdc57	17618	18938	22166	22891
Ccdc58	18244	15378.5	16267.5	34956
Ccdc59	9794.5	22339.5	24170.5	21492
Ccdc6	8453	6816.5	7899	7250
Ccdc60	30052	31666	32519	31136
Ccdc61	18718	16989	19966	20109
Ccdc62	8871	5518	16224	14722
Ccdc63	4835	8303	16693	18793
Ccdc64	24430.25	26740.5	25055.5	15464.5

Ccdc64b	33142	754	7916	15861
Ccdc65	16900	21847	23653.5	23109.5
Ccdc66	22270.33333	17874.33333	19718	16159.66667
Ccdc67	8611	4369	4001	41051
Ccdc68	8995	12448	26520	28023
Ccdc69	26221.5	24410	26760	21623.5
Ccdc70	29856	31358	33720	37672
Ccdc71	17546.5	16999.5	15670.5	14119
Ccdc71l	20025	26268	17035.5	21265.5
Ccdc73	33974	32167.33333	31106.66667	20167.33333
Ccdc74a	26964.5	24779.5	22021	25371
Ccdc77	21564	17902	16118	15225.5
Ccdc78	32745	34522	25716	20843
Ccdc79	32298	31506	31265	29884
Ccdc7a	31075.5	32967	19359	31810
Ccdc7b	32070.5	32031	20158.5	15725.5
Ccdc80	34868	20860	35254	31522
Ccdc81	24898	26335	27002	33049
Ccdc82	38558	39141	36599	37963
Ccdc83	2942.5	22943	23722.5	24248
Ccdc84	314	36415	31477	34503
Ccdc85a	20076	19261.66667	24478.33333	20949.33333
Ccdc85b	10707	12841	14903	13189
Ccdc86	23757	38097	24572	37820
Ccdc87	2521	5558	12756	7671
Ccdc88a	25687.66667	25295.5	26671.66667	22537.83333
Ccdc88b	3267	17694	1749	21673
Ccdc88c	16959.25	26404.75	23015.25	31248.75
Ccdc89	35650	37050	1989	38001
Ccdc9	40639	2441	775	163
Ccdc90b	17541	18878	17494	17037
Ccdc91	15200.33333	18222.66667	15043.66667	15770
Ccdc92	26855.5	27908.5	29113	32898
Ccdc93	16709.66667	11891.66667	16143	12912
Ccdc94	17588.33333	17421.66667	18043	17035.33333
Ccdc96	11079	24514	21310.5	21109.5
Ccdc97	33502	23182	26808	26034
Ccer1	29230	30890	31518	30134
Cchcr1	26003	27678	26238	33203

Ccin	35586	34279	31603	32314
Cck	19663.5	12272	32465.5	20293
Cckar	30620	32437	32867	31405
Cckbr	19175	20767	25235.5	26554.5
Ccl1	39131	40617	35399	2014
Ccl11	25820	1771	28559	32986
Ccl12	13958	25261	19981	27765
Ccl17	18285	16942	19491	20405
Ccl19	26909	28133.5	24946.5	27550.5
Ccl2	38288	32394	26257	31536
Ccl20	5767	9228	7993	23015
Ccl21c	4622	12897	11958	9325
Ccl22	24236	25374	30441	26172
Ccl24	10423.5	25809.5	29211.5	26164.5
Ccl25	23869	11642.33333	10010.33333	13952.66667
Ccl27a	14052	21710	18826	19340
Ccl28	22525	25146	316	1054
Ccl3	2537	4359	36048	36047
Ccl4	33233	23451	32573	22813
Ccl5	30369	22853.5	18433	21120.5
Ccl6	4681	36212	7380	37664
Ccl7	10096.5	18483.5	12413	24027
Ccl8	38135	3879	7452	6064
Ccl9	22691	1408	22168	1749
Ccm2	36949	28223	23359	22061
Ccm2l	9628	11274	13524	12134
Ccna1	14039	7393	20768	20839
Ccna2	13951	13336	14808	17556
Ccnb1	36185	25751.66667	19332.33333	24592.33333
Ccnb2	27868	23653	32596	35586
Ccnb2-ps	40943	36571	31505	24197
Ccnb3	40272	779	924	6748
Ccnc	13141	16020	12935.5	14746
Ccnd1	36603	19023	29351	19544
Ccnd2	26565	19668	15516	16205.33333
Ccnd3	18078	15072.5	19572	39491.5
Ccndbp1	5961	5818	4781	5910
Ccne1	5067.5	9036	6925	8968
Ccne2	9467	12329.5	12119.5	17643

Ccnf	7234	8824	9874	10374
Ccng1	27441.5	26370.5	23885	24467.5
Ccng2	18495	21489	20079	23230
Ccnh	17382.25	16990.25	25080.75	22781
Ccni	21612.71429	17420	16736.71429	21047.57143
Ccnj	3216	4651	1353	3212
Ccnjl	2005	12767	6168	1802
Ccnk	17077	7944.5	10868.5	12504
Ccnl1	23600.66667	21776	15131.33333	16630.33333
Ccnl2	10912	20524.25	19874.5	20195.5
Ccno	20619	30071	23780	40319
Ccnt1	12377	24012	19432	20413
Ccnt2	22910.33333	17335	19038.33333	21119
Ccny	15739	10149.33333	23337.33333	21479
Ccnyl1	14046	15106	14753.5	14227
Ccp110	11153	15622	18176	25736
CCP110	9362.5	2712	19469.5	19479.5
Ccpg1	25137.25	23576.25	14914	22247
Ccr1	9611	16352	21047	23302
Ccr10	5413	1690	12873	9480
Ccr1l1	28755	36061	37030	37917
Ccr2	283	7347	39469	8972
Ccr3	31904.5	31657.5	18142.5	19022.5
Ccr4	20350.5	19329	27093	22658
Ccr5	21056	10796	37992	21858
Ccr6	36105	1096	3522	4361
Ccr7	37593	2442	17799	24194
Ccr8	10128	10890	15836	20521
Ccr9	38678	1413	7647	9257
Ccrl2	8150.5	16978	6742	16761
Ccs	3370	3013.5	22142.5	23310.5
Ccsap	10197	28925.5	13122.5	24708
Ccser1	27895	31085.75	25769	26397
CCSER2	14248.75	23273	22949	23350.25
Cct2	19765	21622	16318	19615
Cct3	24414.5	24129.5	18000	22563
Cct4	30654.66667	22173.33333	18717.66667	21553.33333
Cct5	21597	21768	19647	21148
Cct6a	18078	21099	17277	22745

Cct6b	38110	40187	39446	1064
Cct7	14897	16217	12956	16953
Cct8	8294	10428	8527	13200
Cct8l1	40628	37753	1641	3676
Ccz1	16828	19540	15540	17170
Cd109	16615	28518.66667	12898.33333	16378.33333
Cd14	6443	20019	12052	23719
Cd151	7955	1732	8131	6079
Cd160	9547	19456	16156	17380
Cd163	34129	21409	41054	17195
Cd163l1	13310.5	24541.5	12686	10825
Cd164	32033.5	21833	21664.75	22916
Cd164l2	3552	6300	9833	9453
Cd177	17895	21078	23059	24571
Cd180	29322.5	17906.5	21120	19658
Cd19	4654	9111	10416	11737
Cd1d1	16657	690	15975	39961
Cd1d2	7500	26718	16196	27277
Cd2	37868	28193	29748	29078
Cd200	10789	12158	10708	14059
Cd200r1	38318.5	29743	36783	25111
Cd200r3	24542.5	16129.5	20559.5	7405.5
Cd207	7238	9222	14131	9889
Cd209a	34402	15766	39003	13882
Cd209b	22062.5	31355	6335.5	37752
Cd209c	40039	29977	37192	34448
Cd209d	26302	27431	5	29578
Cd209e	4791	2563	670	29891
Cd209f	22362	23522	26819	27855
Cd209g	6751	4569	8108	11688
Cd22	19914.5	17695.5	10449.5	11493.5
Cd226	25144.75	24391.75	18730.25	20371
Cd244	22281.33333	17536.66667	26691	22151
Cd247	23633.25	19544.75	19421.75	21280.5
Cd248	13097	14708.5	23740.5	26499
Cd24a	22997	18231	15131	15819
Cd27	12182	8682	11557	13732
Cd274	18974.5	20851.5	20419	22011
Cd276	3623	835	22233	28291

Cd28	23421	19916.5	29725	35610
Cd2ap	16070.5	27351	31820	24739
Cd2bp2	13227	13224	12887.5	15419.5
Cd300a	22939	29893.5	21876.5	32768
Cd300e	11455	2744	11105	37951
Cd300lb	27053	20655	24237	22451
Cd300ld	37726	36914	39376	38556
Cd300lf	12866.5	22422.5	11567	18909.5
Cd300lg	11202.5	25126	6350	23578.5
Cd300lh	9075	6264	10362	6120
Cd302	1085	23041	3143	22821
Cd320	22636.5	9292.5	23259	8196.5
Cd33	27202	32390	24158	27378
Cd34	17836	12836	15862	14928
Cd36	31969.5	17355	36804.5	27808
Cd37	5405.5	20706	20438	20983.5
Cd38	28446	31901	19071.5	34247.5
Cd3d	29432	24455	27039	2625
Cd3e	24094	29505.5	27353	27135
Cd3eap	1920	1186	39335	37718
Cd3g	20675	21532	22711	24589
Cd4	22035.5	24761.5	7913	9785
Cd40	34383	13535	40560	14320
Cd40lg	39964	1808	15270	22980
Cd44	14521	28269.33333	29491.66667	29227.33333
Cd46	24214	25435	37412	35757
Cd47	19404.14286	9714.428571	20460.14286	14562.57143
Cd48	25005	17119	18660	16109
Cd5	16394	17363	18383	17484
Cd52	35432	6159	35093	5158
Cd53	31695	31694	25272	30657
Cd55	22798.25	19344.25	14283.25	15103.75
Cd55b	30563	29939	31835.5	29215
Cd59a	18486.5	18059.5	20357.5	31258
Cd59b	20088	19774	25431.5	19376
Cd5l	20884	8504	28051	19513
Cd6	7930.5	9505.5	12250	12124.5
Cd63	15189	16402	17742	17683
Cd68	7686	2850	4954	6460

Cd69	40111	10516	37811	5049
Cd7	9593	12743	14677	12772
Cd70	5370	2807	7536	6423
Cd72	15200.5	38385.5	18982.5	36820.5
Cd74	18702	23293	15576	19993
Cd79a	28275	27443	35561	38728
Cd79b	15810	13456	13951	6097
Cd80	13110	13755.5	12009	12843
Cd81	37749	36203	36564	28839
Cd82	11683	21123	9826	19990
Cd83	13107	1725	6543	38123
Cd84	34771	27113	34893	31765
Cd86	18353.5	21357.5	19087	23832
Cd8a	17627.33333	32952.33333	12631.33333	26606.33333
Cd8b1	18390.5	20976	15714	17590
Cd9	16995.75	24590.75	18296	19487.25
Cd93	28264	14038	27079.5	16264
Cd96	29529	4477	32178	36106
Cd99	19859.5	24395	21623.5	22866
Cd99l2	15643.33333	13248.66667	15494.66667	11759
Cda	26904	24787	39077	35262
Cdadc1	7757.333333	11891.33333	5930.333333	15253.33333
Cdan1	28865	27239	27153	27864
Cdc123	25672.33333	4357.333333	14824	13811.66667
Cdc14a	23062.6	19458.4	21654.8	18712.2
Cdc14b	19978	20491	17041.5	36574
Cdc16	19011.33333	27486.66667	18423.33333	19485.66667
Cdc20	31105	26837	30961	36213
Cdc20b	36627	31283	30741	29517
Cdc23	16176.5	15655	13295	14931
Cdc25a	8889.5	25845	18644	20960
Cdc25b	13821.66667	21700.33333	12261.66667	21992.33333
Cdc25c	15443	14194	10863	12527.5
Cdc26	23016.4	16824.8	20426	16927.4
Cdc27	15190	6939	26034.33333	23342.33333
Cdc34	17980	20371	16933	18830
Cdc37	30811.5	31718.5	29211.5	11655.5
Cdc37l1	13547.25	10859	18607.25	19351.75
Cdc40	29636.33333	28215.33333	25619.66667	13375.33333

Cdc42	23291.25	23443	20650.5	25279.75
Cdc42bpa	24779.25	25919.25	28258.75	19288
Cdc42bpb	1813	785	40919	39237
Cdc42bpg	2129	10606	2709	5728
Cdc42ep1	20486.5	19084	13309.5	13395.5
Cdc42ep2	21499	29309	16640	27521
Cdc42ep3	19083.5	38066	23239.5	12675.5
Cdc42ep4	13462	10538.5	13788.5	8845
Cdc42se1	27062	6264	7068.5	25263
Cdc42se2	32014	26013	24884.5	27335
Cdc45	14633	9907	12975	11799
Cdc5l	10111	9186	9572	8531
Cdc6	37387	23186	16169	12830
Cdc7	14445	16584	12550	14902.33333
Cdc73	20900	20423	21053.66667	20947.66667
Cdca2	32906	19861.33333	30042	29406
Cdca3	15515	12094	14425.5	31285.5
Cdca4	3910	3178	1586	2023
Cdca5	23213	22995.5	20494.5	22128
Cdca7	14217	20777	10503	18447
Cdca7l	38273	27714	39160	27660
Cdca8	17088.66667	17608.33333	27912	29466.33333
Cdcp1	33930.33333	22125.33333	18539.33333	18969
Cdcp2	8343	15249	20519	20723
Cdh1	14759	1059	10791	37277
Cdh10	28174.14286	24428.85714	22790.42857	12357.42857
Cdh11	25177.5	21095.33333	25040.16667	22989.83333
Cdh12	31114	2708	4843	1527
Cdh13	16284.5	29421	19585.5	24431.5
Cdh15	14829	14971	24357	25418
Cdh16	31159	27673	39363	39543
Cdh17	30944	32635	34495	32685
Cdh18	19690	19058	25877.33333	13839
Cdh19	28154	29578.5	31722	32552
Cdh2	30707	24297.75	34949.5	36784.5
Cdh20	19186.66667	20365.33333	26369.33333	23793.33333
Cdh22	3990	12248	3997	7286
Cdh23	25045.5	22108.5	25471	6918
Cdh24	18079	20447	26893	26772

Cdh26	33435	36946	40575	39779
Cdh3	3224	10365	7192	15779
Cdh4	27983.66667	33833.66667	23930.33333	25021.66667
Cdh5	36210	19312	20128.5	10545.5
Cdh6	8973	4582	9941	36807
Cdh7	29629	29274.5	10092.5	28365.5
Cdh8	28322.66667	24607.66667	31205	31698.66667
Cdh9	39922	7830	6346	5890
Cdhr1	32935	34700	36926	1954
Cdhr2	8442	9254.5	12526	15131.5
Cdhr3	26886	34796	39554	39460
Cdhr4	16288.5	20072.5	22559.5	23298.5
Cdhr5	20419	24174.5	24833	26492.5
Cdip1	23255.75	18782.25	21003.25	19663.5
Cdipt	18141	13476	16055	17227
Cdk1	16093.5	11329	17573	17937
Cdk10	20180.5	17546.5	18155	23087.5
Cdk11b	13235	27138.5	26786.5	27115.5
Cdk12	9999.25	14488.75	17649.5	15699.75
Cdk13	16170.2	25684.2	26732.2	24822.8
Cdk14	21038.42857	24757.71429	21338.57143	22362.85714
Cdk16	23809.5	22608.5	21245	21887.5
Cdk17	19084.2	16902.8	16910.2	16906.2
Cdk18	19653	14483	22195	18514
Cdk19	11317.5	6739.5	9209.5	4509.5
Cdk2	20804	22864.5	22097	22061.5
Cdk20	7632.5	14594	24965.5	15218.5
Cdk2ap1	35644	28853	23202	22917
Cdk2ap2	775	39475	1104	37831
Cdk3-ps	31611	28781	32585	30068
Cdk4	33471	33417	29754	35899
Cdk5	8385	11018	11210	13477
Cdk5r1	5633	6240	3429	3500
Cdk5r2	22785	16017	11685	17252
Cdk5rap1	1252	4497	2560	4016
Cdk5rap2	21189.5	19746.5	10790.5	10295.5
Cdk5rap3	19411	21308.5	21755.5	22097.5
Cdk6	23622.75	23543.25	26556.25	27809.75
Cdk7	9531	12835.5	19438.5	19727

Cdk8	20190.33333	19604.33333	19562.33333	19699.66667
Cdk9	16578	14009	18410	16628
Cdkal1	15500	16455	13917	12401
Cdkl1	37400	37830	1181	38868
Cdkl2	24186	21112	23720	24079.5
Cdkl3	18325	18664.5	21948.5	24114
Cdkl4	24766	39326	28564	29413
Cdkl5	8290	37353	3121	273
Cdkn1a	19267	14157	17175	17918
Cdkn1b	10534	37618.5	15856	30694
Cdkn1c	19416	13046	19264	16740
Cdkn2a	36468	2422	30131	35548
Cdkn2aip	16686.33333	19524.33333	17886.66667	18782.66667
Cdkn2aipnl	16656	18467	19126	17762
Cdkn2b	34505	5711	39903	11768
Cdkn2c	16257.5	14075.5	18188.5	8468
Cdkn2d	16789	16537	18525	17179
Cdkn3	18032.66667	18951.33333	16631.66667	19406.66667
Cdnf	2001	1879	7609	4287
Cdo1	33006	14333	36563	14990
Cdon	1864	6235	7435	11408
Cdpf1	35027	37913	36751	35710
Cdr2	17997	22072	25195	26804
Cdr2l	1327	39698	5904	5506
Cdrt4	32637	37968	37517	4206
Cds1	26210.5	30137.5	24550	30964.5
Cds2	21803.8	21193.8	24958.4	23711.6
Cdsn	20037	22885	23962	25855
Cdt1	7975	32888	5045	35803
Cdv3	17596.75	10503	19622.75	21812.5
Cdx1	25079	26270	28330	27119
Cdx2	24182	28551	28749	35210
Cdx4	27405	28862	30266	28943
Cdyl	22248	27452.5	22971.5	28171
Cdyl2	23674	31223.5	20578.5	27431
Ce9	16683	17886.5	6251	6940
Ceacam1	10359.33333	9513.666667	7630.666667	5994.333333
Ceacam10	22466	1506	1990	13122
Ceacam11	2161	1455	463	8282

Ceacam12	24710	3667	38942	5034
Ceacam13	14179	13522	22037.5	18241.5
Ceacam14	985	8104	13549	1228
Ceacam15	32094	33366	36740	34705
Ceacam18	31079	35810	37403	6876
Ceacam19	2005	11397	21865	8570.5
Ceacam2	29429.5	12989.5	19405.5	21342
Ceacam20	33917	40099	13966	13390
Ceacam3	38710	37333	518	32191
Ceacam5	34542	2112	10917	12785
Ceacam9	7359	33199	8055	3625
Cebpa	9455.5	6684	8472.5	4535
Cebpb	9679.5	20894	15659	24199
Cebpd	30206	40655	26118	34518
Cebpg	21173.66667	17585.66667	20562.33333	19423.33333
Cebpz	22462.5	21226	18449.25	19892.75
Cebpzos	22121	26071	24547	22317
Cecr2	19618	17052.5	18443.5	18630
Cecr5	22177	26960.5	26572	10693
Cecr6	487	2831	39001	700
Cel	5932	9481	17035	17794
Cela1	17837.5	10156.5	17602.5	4124.5
Cela2a	29043	30715.5	32735	30921.5
Cela3b	37952	39557	7240	6938
Celf1	33803.66667	16456.66667	32006	31198.66667
Celf2	24548.09091	16799.27273	22493.18182	15454.09091
Celf3	18227	20699	23873	21393
Celf4	9376	3348	2391	38943
Celf5	17893.66667	8613	18247.33333	19444
Celf6	21239	13808	12482	12455
Celsr1	35956	31302	30068	36519
Celsr2	19525.66667	21274	24368.66667	24903.66667
Celsr3	14311.5	11337	12485.5	13809
Cemip	23983.75	18642	14609	14774
Cend1	38280	4654	1539	6225
Cenpa	3064	18940	49	17915
Cenpb	38202	37360	1400	41019
Cenpc1	31778	28646.5	30704.5	29822.5
Cenpe	24954.66667	19926.66667	33024.66667	20449

Cenpf	13028.5	31977.5	16490	14529
Cenph	21247	14679.5	16917	19723
Cenpi	1811	33054	37970	37061
Cenpj	3862	3860	3891	2123
Cenpk	20848.33333	18432.33333	34182.66667	32870
Cenpl	24070	7509.5	6913	7658
Cenpm	35358	959	31274	6773
Cenpn	4236	40902	1815	40564
Cenpo	20439.33333	17537.66667	10133	6402.666667
Cenpp	18083.33333	20602.66667	13758.66667	24664.33333
Cenpq	23356	23850	25820	25331
Cenpt	40836	37068	38438	34399
Cenpu	13008	8139.5	30327.5	27491.5
Cenpv	7614	10820	13752	11398
Cenpw	8226.5	17765	10822	12502.5
Cep104	18351	15212	16882	17155.75
Cep112	32687	24978	7884	22078
Cep120	27522.4	26109.6	20477.8	22758.6
Cep126	34015.5	30237	31859	30366.5
Cep128	16195.42857	13564.42857	17614.57143	18211
Cep131	10841	7032	10836	4014
Cep135	14056.5	13710	12920	33396.5
Cep152	39033	31894	32683	31293
Cep162	27810.66667	17991.66667	34126.66667	30636.33333
Cep164	12767.33333	10627.33333	13030.66667	12186.66667
Cep170	24529.16667	17010.5	16355.5	21434.33333
Cep170b	20733	23315	30117	36270
Cep19	27811	28420	29082	26969
Cep192	21896	17639.5	24921.75	28927
Cep250	18434.5	33736	35437	31049.5
Cep290	11164	5134	9319	8013
Cep295	21904	21278	20775	22168
Cep350	5050.5	3786.5	22365.5	4904.5
Cep41	16633	14334.75	14592.5	13955.25
Cep44	14638	37596.5	33506	34390
Cep55	12132.5	14320.5	15814	14574
Cep57	24253	29944	23719	31590
Cep57l1	26158.66667	30877.33333	15348.33333	26699.66667
Cep63	24139.5	25916.5	19699.5	22278.5

Cep68	35063	27617	28402	34122
Cep70	18801	13936.66667	14766	10892.33333
Cep72	22216	17088.5	19898	23639.5
Cep76	15095.5	15329	12958	17427
Cep78	12469	18327	14004	19102
Cep83	11389.33333	14010.33333	13756	15092.66667
Cep85	11286	14639	15052	17884
Cep89	11907	9337	14322	12035
Cep95	18371.5	15394	17808.75	18830.5
Cep97	8416.333333	19094.33333	10402	18391
Cept1	5491.5	6399.5	2192.5	6360.5
Cer1	31834	33691	35059	33688
Cercam	15087	15951	19884	16939
Cerk	8925.5	2648	9790.5	4104
Cers1	29024	23166	27273	27702
Cers2	17920	13257	16920	13642
Cers4	24244	11352.33333	10608	13849.66667
Cers5	26275	21850	23385	21539
Cers6	12162.5	13104.5	8400	10667.5
Ces1b	31738	28604	985	38588.5
Ces1c	16475	17407.5	20599.5	24992
Ces1d	14565	16320	21671	22350
Ces1e	37357	29925	7173	3359
Ces1f	30625	32765	32720	31155
Ces1g	35132.5	19624	22831	19929.5
Ces1h	20640	22970	25656.5	25611
Ces2a	22956	27845	35124	35796
Ces2b	26318.5	26089	11281.5	10310
Ces2c	26288.5	32300.5	32509.5	31331.5
Ces2e	16756	17202	33061	34821.5
Ces2g	9692.666667	16117	13209.33333	13869.66667
Ces3b	21031	21038	38404	37310
Ces4a	22438	28095	35109	3463
Ces5a	17853.5	3711.5	20029	5161.5
Cetn1	18802	21576	24005	24936
Cetn2	25916.5	29356	27046	26662.5
Cetn3	17080.5	19037	14925	18657.5
Cetn4	40176	29994	4880	37525
CF750939	25200	36411	17634	36167

Cfap126	35984	24219	25842	21116
Cfap20	34860	28565	25004	24940
Cfap221	3398	36845	34999	37539
Cfap36	14333	32214	15588.5	15776
Cfap43	19760	16456.33333	20834.66667	18150
Cfap44	2244	2692	12244	11259
Cfap45	29014	33081	36891	37999
Cfap52	40374	2699	36097	6232
Cfap53	40960	2482	4557	4793
Cfap57	30234.5	26638	33375	17744.5
Cfap58	39874.5	33921	20923	23010.5
Cfap61	29140	30426.2	21422.2	23455.2
Cfap69	12462	14906	28618	31910
Cfap70	26871.5	26760.5	24594	24771
Cfap74	14169	17831	37497	21327.5
Cfap97	2304	2454	2715	2650
Cfb	39560	4087	871	4245
Cfc1	14071	17510	21372	22517
Cfd	3046	26389	4563	12219
Cfdp1	4204	9547	11277	13908
Cfh	19077	15572.8	23408.8	15201
Cfhr1	25875	26985	29533	28270
Cfhr2	27146.5	22657.5	28431.5	23429.5
Cfi	5218	1775	11249	10390
Cfl1	32471	31516	33394.5	32958
Cfl2	40001	35729	36489	38335
Cflar	20431	16158.42857	18663.28571	20604.28571
Cfp	20941	11662	20238	13637
Cftr	15820.5	18435.5	19635	19003.5
Cga	20811	22191	24850	30197
Cgb	8128	8003	13165	12688
Cggbp1	10621	8527	6188	10059
Cgn	5230.5	16492.5	9093	4949.5
Cgnl1	18943.16667	24831.83333	20628.83333	26620.16667
Cgref1	17812	6234	30529	15829
Cgrrf1	9193	12609	8003	10260
Ch25h	10341	35837	16879	9424
Chac1	15211	18880	17103	18988
Chac2	34920	1948	38279	1281

Chad	9805	12371	19141	18115
Chadl	17571.33333	17323.33333	22323.66667	20445.66667
Chaf1a	33564	22215	24309	20696
Chaf1b	15530	19980	18633	21590
Champ1	21762	20851	23689	22786
Chat	37292.5	35477.5	23710	36492
Chchd1	14566.5	7545.5	8132	8572
Chchd10	34894	1123	26748	39876
Chchd2	29082	34290.5	31970	34317.5
Chchd3	23547.25	12572	11516.75	11426.75
Chchd4	20611	26448	21363	26726
Chchd5	14451	18084	16889	16452
Chchd6	24056	1633	36853	1899
Chchd7	28413	32265.5	33451	34205.5
Chd1	17051	24523.25	14585.5	16754.25
Chd1l	12838	11102	11699	10372
Chd2	14570.33333	24107	27594.33333	22203.66667
Chd3	14779	26987.83333	19656	20989
Chd4	25753.75	30425	26981.5	23301.25
Chd5	17279.33333	17348.33333	33566.33333	10976
Chd6	16732.83333	11112.83333	12323.16667	11042.33333
Chd7	23852.5	21290.75	17361.5	18928.75
Chd8	26477.5	16678.5	21785	23091
Chd9	19299.83333	28244.16667	28990.83333	26234.16667
Chdh	32568.66667	31128	34091.33333	31860.33333
Chek1	16235.5	11563.5	14716.5	16684
Chek2	18870	15582	17745.5	12476.5
Cherp	37542	35133	37250	35169
Chfr	22001	19728.66667	17192.66667	17956.66667
Chga	23416	24774	741	1217
Chgb	26422.5	34122.5	34193.5	32898.5
Chia1	26404.5	31452	37675	25333
Chic1	4195.5	20428.5	23889.5	24488
Chic2	27883	30784.5	27093	31653
Chid1	25502	21973	19068.66667	23198.66667
Chil1	6909	10946	13891	15374
Chil3	27641	22895	24867	22685
Chil4	33635	25084	25247	26748
Chil5	25231	29950	2170	4700

Chil6	16520	19728	23195	23957
Chit1	9794	12198	13381	20966
Chka	20414.5	24361.75	21734.75	22602.75
Chkb	26670.5	19105	20755	18741.5
Chl1	27825.5	29114.5	31474.5	20395
Chm	11280.66667	10730.66667	26520.33333	14436.33333
Chml	19257	36439.5	32710	38334.5
Chmp1a	8833	3290	4289	4973
Chmp2a	5531	8914	11322	12260
Chmp2b	17987	15913	14959	16265
Chmp3	10713.5	13612.5	11871.5	13883.5
Chmp4b	30795.5	30617	34804.5	36337.5
Chmp4c	35063.66667	37116.33333	3152.333333	2862
Chmp5	23819.33333	27646.33333	16382.66667	18404.33333
Chmp6	4520	5075	40861	3457
Chmp7	5063	5582	11059	11241
Chn1	23344	19907	13515	21999.66667
Chn2	20412.66667	26039.33333	24731.33333	17506.33333
Chodl	26819	30900	35862	39121.5
Chordc1	17283	22122.5	19883.5	23288
Chp1	20564	19481.66667	21724.66667	22049.33333
Chp2	21622	36754	12275	38613
Chpf	13917	22266	21456	25952
Chpf2	39272.5	6771	17961	5552.5
Chpt1	16217	19474.33333	10084	15421.33333
Chrac1	28721.33333	34426.33333	29971.66667	33027.66667
Chrd	15192	17430	21008	21516
Chrdl1	14661.33333	21090.33333	20803.33333	21749.33333
Chrdl2	34795	28657	29633	30614
Chrm1	12943.5	31737.5	34344.5	34848.5
Chrm2	24121	2899	29627	25766
Chrm3	22029	26216	25638.5	26974
Chrm4	20437.5	21219.5	7522	10100.5
Chrm5	12043	14565	19946	17560
Chrna1	19250.5	9999.5	35777.5	20700.5
Chrna10	28390	33601	829	2060
Chrna2	35429	3983	37619	7262
Chrna3	28746.5	30425.5	21147.5	29368
Chrna4	21130	22736	26298.5	24669

Chrna5	5613	38576	7300	4027
Chrna6	24936	26386	40784	2338
Chrna7	23581	25109.33333	20207.33333	20163.66667
Chrna9	17981	18194	20026	15646
Chrnb1	10698	10075	12749	14411
Chrnb2	29397	12782	23040	22751
Chrnb3	20220	24151.5	25605.5	26816
Chrnb4	38833	2596	29388	40119
Chrnd	20535	12816	18783	22342
Chrne	11266	9790.5	13562.5	13889
Chrng	11241.5	20723.5	21305	16899
Chst1	38693	10338	824	10704
Chst10	36074.5	36234	19382	17531.5
Chst11	24802.33333	24635.66667	15629.33333	15495.33333
Chst12	15071	16138.5	12747	15198.5
Chst13	29408	30898	33881	37677
Chst14	21902	18003	18715	13685
Chst15	10742	18165	12737	18699
Chst2	19054	35962	24478	19449
Chst3	26112	22042.5	19413	30565
Chst4	4633	5620	14200	14469
Chst5	40531	9539	21065	20447
Chst7	40856	37973	241	34414
CHST7	6924	1681	6499	12608
Chst8	32298.5	33924.5	21172	21046
Chst9	33226	34950	36857	36761
Chsy1	15893	15949	15716	16848
Chsy3	22916.66667	28370	26603.66667	20832.33333
Chtf18	16310	9515	13850	8152
Chtf8	30200	25561	26966	26036
Chtop	22291.5	23005	22306	22599.5
Chuk	22691.5	20248	17690	20710.5
Churc1	5756	7842	8709	6405
Ciao1	26638	27434	22291	27591.5
Ciapin1	25683	28074	27921.5	31239.5
Cib1	8815	13077.5	11323.5	12756
Cib2	3028	5072	36010	35915
Cib3	33145	35099	35962	40041
Cib4	18547	20101.5	25228.5	23637

Cic	29061.33333	24748	27697	24132
Cidea	3343	7323	9343	2302
Cideb	6682	4951	19865	17274
Cidec	38265	39744	4575	2046
Ciita	12368	10463	2268	4687
Cilp	35515	36174	30313	36558
Cilp2	2286	5082	12023	10097
Cinp	15946.25	24074.5	29065.75	31682
Cipc	25318.33333	15709	20248.33333	18114
Cir1	26672	29583	30914.5	10760.5
Cirbp	5067	671	10115	4492
Cirh1a	10197	14395.33333	7484.333333	11300
Cisd1	3966	10053	1428	5956
Cisd2	10793	22136	18400	22121
Cisd3	17653	23195	19727	22185
Cish	36887	7637	26829	3872
Cit	21525	17278.33333	16500.66667	14923.33333
Cited1	10214	12351	12162	15240
Cited2	28316.5	27996.5	31906.5	23210.25
Cited4	13842	13043.5	13958	12647.5
Ciz1	8896.5	28456.5	11402.5	11583
Ckap2	22036.33333	17738.66667	26476	25589.66667
Ckap2l	19363	17713	17650	22846
Ckap4	16077	11880	10175	5347
Ckap5	14674.25	6617.25	17382.25	15940.25
Ckb	15641	10783	14312	14488
Cklf	13564.5	13026	11921.5	10620.5
Ckm	20665	19510	24261	22238
Ckmt1	34674	36751	39787	3204
Ckmt2	24046	25174	26937	35975
Cks1b	13456.5	13622.5	16012.5	15942
Cks2	12110	11837	21530.5	15191.5
Clasp1	9616.666667	10084.5	13383.66667	19170.16667
Clasp2	24481	19731.33333	26262.66667	22823.66667
Clasrp	4502	1302	6962	6294
Clca1	8383.5	23504.5	24544	27387.5
Clca2	21610.5	18625	29758	27315
Clca4a	13381.33333	16455	14149	18938.33333
Clcc1	11941	10633	10662	11340

Clcf1	35239	39193	41045	900
Clcn1	4918	24446.5	9689.5	4431
Clcn2	34162	30282	35431.5	37037.5
Clcn3	24351	30216.25	31242	22521.5
Clcn4	7345	4618	3785	39734
Clcn5	7540	3160	23440.5	25058.5
Clcn6	24094.5	25558	22315	24548
Clcn7	13701	19018	8922	16195
Clcnka	32472	31328	1993	1199
Clcnkb	2585	2124	10275	9461
Cldn1	35832	33397	34712.5	32792.5
Cldn10	21609	20164	20379	19675
Cldn11	12003	14542	16131	13088
Cldn12	15238	11453	8948	10868
Cldn13	12623	14989	23601	21598
Cldn14	21658	23925	26340	26521
Cldn15	19358	34068	19786	25256
Cldn16	33340	38747	30452	30109
Cldn17	16507	17184	28786	25885
Cldn18	39875	35221	36102	8730
Cldn19	26843.5	29658	16843	17088.5
Cldn2	37098	4934	27113	9929
Cldn22	8913	13699	24892	27046
Cldn23	22079	36026	14314	24927
Cldn3	33261	33550	40277	41074
Cldn34b2	30091	20954	22964	22818
Cldn34b3	12656	17793	17698	17242
Cldn34c1	21291	26920	28909	23631
Cldn34c4	5206	7757	6076	3045
Cldn34d	4746	7442	12039	14711
Cldn4	33359	849	38709	59
Cldn5	27026	24850	31673	34255
Cldn6	22965	25185	33108	33392
Cldn7	40983	1603	7001	4462
Cldn8	27704	29402	3952	5128
Cldn9	5837	10235	35681	3859
Cldnd1	26336	9285.5	6802	9654.5
Cldnd2	3327	2479	7947	5949
Clec11a	25529	32304	19951	24372

Clec12a	37745	35747	29666	13078
Clec12b	33793	24846	33392	37902
Clec14a	27281	24647	27555	24573.5
Clec16a	24137.5	21736	23026	22419
Clec18a	18821	25332	23770	33415
Clec1a	2225	16118	31082	35124
Clec1b	16823	17182	20867	20876
Clec2d	34446	25528	5923	4225
Clec2e	13838	28234	34785.5	17953
Clec2g	8629	4190.666667	16801.33333	15381
Clec2h	36276	33161.5	26800	23874.5
Clec2i	4585	37987	38013	34799
Clec2l	34464	37483	40230	38072
Clec3a	31207	2116	12417	10550
Clec3b	38553	1476	7093	6339
Clec4a1	27441	23321	22289	22849
Clec4a2	23244	22995.33333	23577	22335.66667
Clec4a3	682	33646	38153	27269
Clec4b1	39919	34589	39361	25794
Clec4d	35978	9102	40894	12249
Clec4e	35551	16485	3446	18305
Clec4f	1309	5100	31262	37264
Clec4g	37422.33333	37977.33333	5333.666667	14836.33333
Clec4n	10258	12783	19260	23792
Clec5a	18880	10200.66667	12730	23405.66667
Clec7a	12749	6873	13593	13248
Clec9a	23069	30304	20119	36071
Clgn	21892	23211	27250	22792
Clhc1	29186	39563	27419	29079
Clic1	6166	8970	11426	10262
Clic3	10974	12309	16641	16291
Clic4	18519.66667	19235.66667	19718.66667	22460.66667
Clic5	13713.5	25934.5	26035	34478
Clic6	18777.5	18401.5	26640.5	24319.5
Clint1	22097	20583.5	19979.5	21231.5
Clip1	20135.66667	26239	30230.33333	26687.66667
Clip2	34407	29567.5	30442	29092
Clip3	20434	21167.66667	24507.33333	25955.33333
Clip4	18364	18960.5	12646	11057.5

Clk1	21621	37772.5	20263	18801
Clk2	6245	7024	8567	5593
Clk3	18118.5	16120	17972.5	19657
Clk4	4071.75	5084	4766	4984.5
Clmn	39455	18221.5	4921.5	1039
Clmp	14623.5	15847.5	14255.5	17743.5
Cln3	35762.5	27714.5	24617.5	24723
Cln5	13435	18377	13112	20156
Cln6	12455	8595	5787	39983
Cln8	22610.5	17301.5	19568.25	18502.25
Clnk	28427.5	11372.5	31065.5	12222.5
Clns1a	35142	38661	40198	408
Clock	17827.75	21376.25	19534	23694.25
Clp1	9341	9615	5934	8033
Clpb	23727.33333	23043	12742	21374
Clpp	35435	41068	40142	650
Clps	28949	29245	24253	24445
Clptm1	9406	8547	5834	5938
Clptm1l	12653	10971	8831	9588
Clpx	22564	17939.33333	16866.33333	18083.33333
Clrn1	27983	12357	18294.5	15862.5
Clrn3	3719	5874	39373	6145
Clspn	37031	32672	37406	32506
Clstn1	23956	34072.66667	17649.33333	15094.66667
Clstn2	28750	18141.4	20937.6	26068.8
Clstn3	22217	22545	28160	26671
Clta	5318.5	20684	6549	6168
Cltb	7506	9768	9574	9807
Cltc	30987	31721.75	32887.75	35397.5
Clu	3995	39112	19577	17961
Cluap1	21420	20292.5	18835	18115.5
Cluh	6318	1522	7201	3340
Clvs1	36078	25666.33333	25374.66667	22976
Clvs2	29033.66667	28175	20935	21060.66667
Clybl	26879	26607	20012	25036
Cma1	26788	7221.5	21819	17932
Cmah	26072.66667	27374.66667	25069.33333	5807.333333
Cmas	34422	24544	22568	24166
Cmbl	28194	24381	22351	16459

Cmc1	31045	18998	29794.5	34764
CMC2	19534	21807	21532.5	12824
Cmc4	4864.5	7927	10528.5	8098
Cmip	22385.5	18501	16298.5	12295.5
Cmklr1	19588	22173	24968	24331
Cml1	13732.5	15475	22541.5	4808.5
Cml2	21958.5	21998.5	12919.5	10450.5
Cml3	20745	22913	22057	26640
Cml5	34500	31776	31437	29866
Cmpk1	2979	2469	9017	6052
Cmpk2	14513	9389	24749	11023.5
Cmss1	19420	11055.75	21675.25	18788.75
Cmtm1	13901	16575	21716	19774
Cmtm2a	7573	7639	6452	4734
Cmtm2b	1415	7756	14969	16082
Cmtm3	12716	6582	15244	10964
Cmtm4	26908	31048	31349	30825.66667
Cmtm5	10097	21338	22316	23217
Cmtm6	11049	6200	10689	5233
Cmtm7	16354	15456.5	14662	16248.5
Cmtm8	22899	37836	36479	40963
Cmtr1	35521	37500	33919	36137
Cmtr2	17458	17088	17785.5	18086.5
Cmya5	21324.33333	32869	23382	12057
Cnbd2	10122.5	26380.5	27757.5	24613
Cnbp	20807	24102	24418	26272
Cndp1	12750	12002	13643	19617
Cndp2	16089	15756	12643	14702
Cnep1r1	30329	30037.5	27298	24656
Cnfn	15723	15979	19060	18633
Cnga1	15145	12069	20200	21435
Cnga2	15803	18353	21764	21781
Cnga3	36537	9751	15282	6438
Cngb1	221	32737	6995	31859
Cngb3	28534	27526	34475	29672
Cnih1	17220	13928	14658	13426
Cnih2	12819	13298	15681	14732
Cnih3	11723	5569	34512	4670
Cnih4	18776.5	24778	18562	20722

Cnksr2	24232	25315	27791	35222
Cnksr3	11316.33333	4127	11566.33333	6324.333333
Cnn1	20857	23008	22635	24961
Cnn2	1687	32202	39113	24475
Cnn3	38318	5551	38045	4817
Cnnm1	25042	28761	30953	35254.5
Cnnm2	15469	15016	17521	14304.5
Cnnm3	27308.33333	21138	20955	16276.33333
Cnnm4	15297	10674	9490.5	13111
Cnot1	25474.33333	24484.66667	21551	24398.66667
Cnot10	23690.33333	22550.66667	19036.33333	18634
Cnot11	4732.5	15456.5	2469.5	20481.5
Cnot2	14543.5	23311.25	23944.25	23917.75
Cnot3	14942.33333	9657.333333	22022.66667	20273.66667
Cnot4	17053.88889	19295.11111	19283.77778	25126.22222
Cnot6	28269	21576.33333	22019	21594.66667
Cnot6l	25219.25	19347.25	22687	19177
Cnot7	17851	24708.5	23872	29419.5
Cnot8	11956.5	14454.5	13618	15267
Cnp	15623	9426	10339	6299
Cnppd1	26275.5	18526	23487.5	20644.5
Cnpy1	31533	33683.5	1635	20221
Cnpy2	12140	15146	14510	15205
Cnpy3	4550	4525	6484	3502
Cnpy4	17663.5	18135	18105	15786.5
Cnr1	18937	22369	21364	21885
Cnr2	20408	24269.5	12864.5	11310.5
Cnrip1	16799.5	18457.25	20914.75	2948.5
Cnst	16952	37003	40430	20013
Cntd1	31328	33693	33931	32182
Cntf	26792.5	23265	24696	24668.5
Cntfr	10246	10160	15048	14403
Cntln	30783	21781.33333	23067	25412.66667
Cntn1	27307.5	18460.75	14227.25	16587.75
Cntn2	31264.33333	24614	25503.66667	25461.66667
Cntn3	17617.5	17616	23814	18479.5
Cntn4	34999.25	25957.25	26824.25	16934.5
Cntn5	24647.33333	23740.66667	23768	22948
Cntn6	32629	39613	35432.5	2153.5

Cntnap1	11421	26346	26977.33333	29727.33333
Cntnap2	19465	22297.66667	13461.66667	13519.66667
Cntnap3	14469	10858	21739	14319
Cntnap4	26307.66667	26699	29272	28213
Cntnap5b	35760	6284	4901	6857
Cntrl	11142	6770	26673	5058.5
Cntrob	20672	25123	20909	21669
Coa3	18992	21094	19938	20131
Coa4	35814	33872	31957	34005
COA5	21666	23456.66667	35478.66667	22387.66667
Coa6	16346.5	20155.5	17397.5	17573.5
Coa7	6562	8815.666667	19534.33333	20715
Coasy	24815	25638	23113	24684
Cobl	39414	13721	6468	15321
Cobll1	23579.33333	29102.33333	7939	33016
Coch	26826.66667	21647	30245.33333	29428
Cog1	33662	30588	35142	33809
Cog2	4591.5	4015.5	20392	21253
Cog3	31957	29519.5	31985	31650
Cog4	15001.25	23766.75	23871.25	9932
Cog5	1575	38754	29080	28659
Cog6	21870	21302	20512	21637
Cog7	31744	25512	23663	23590
Cog8	9202	9770	6808	6828
Coil	8812	10744	12690	13196
Col10a1	26991	28165	30380	29084
Col11a1	30129.5	30639.25	31656.25	31351.25
Col11a2	16957.5	15020	26522.5	22871
Col12a1	33352	14506.5	21845	23460
Col13a1	22114.66667	20610.33333	28304.33333	17212
Col14a1	15126.5	16996.5	18740	18432.5
Col15a1	9398.5	34958	26554	26216.5
Col16a1	36085.5	13934	28726	9467
Col17a1	32791	37961	24981.5	21335.5
Col18a1	21044	27711.5	29655	17689.5
Col19a1	22372.75	29205.75	28665.5	32351.5
Col1a1	10564	22648.66667	30824.33333	13624.66667
Col1a2	19977.5	18195.5	23602	25595
Col20a1	4453	2342	6826	4564

Col22a1	23432.33333	14922	16650.66667	16193
Col23a1	12829	13614	24198.2	16759.8
Col24a1	10554.5	3060	15740.5	11985
Col25a1	29752.4	24556	27437.6	20791.6
Col26a1	33315.5	34264	36111.5	18518.5
Col27a1	14092.33333	19530.33333	11893	31779
Col28a1	26366	41047	475	39513
Col2a1	9176	12526.66667	16210.33333	19217.33333
Col3a1	25076.66667	21497.66667	26898.33333	29929.33333
Col4a1	29504.33333	19442.66667	20184.66667	25279
Col4a2	17846.5	29786	26487.5	34795.5
Col4a3	29566.33333	27909	21958.66667	22227.66667
Col4a3bp	20560.33333	27274.66667	20873.16667	18713.33333
Col4a4	35084.5	17297	38734.5	37302
Col4a5	6823	4163	16713	18880
Col4a6	22918	24343	30528.33333	31911
Col5a1	13564.5	18407	16321.5	21980
Col5a2	35654	30202	628	7576
Col5a3	17185	23718	13940.5	12196.5
Col6a1	39044	32088	784	39
Col6a2	15785	14244	24077	25314.5
Col6a3	16535.66667	14224.66667	12952.66667	18268.33333
Col6a4	34526	36567	40587	39578
Col6a6	32881	36481	15932	13701
Col7a1	6395	3950	9617	11911
Col8a1	2759	23859.5	21248	23533
Col8a2	192	38144	34889	36968
Col9a1	15702	17966	22368	15552
Col9a2	19415	18909	21775	19187
Col9a3	18924	19524	21755	29462
Colec10	24879	37233	3256	40680
Colec11	33741	36685	38524	33891
Colec12	20253.33333	28755.66667	18212.66667	13295
Colgalt1	7191	10752	5927	10474
Colgalt2	6730	22610.5	30681	13856
Colq	4579	2835	13200	10728
Commd1	34702.33333	14693	22716	26628.66667
Commd10	33713	36807	28258	31560
Commd2	20112	20824.5	13831.5	16883

Commd3	2173	6365	5122	5653
Commd4	36007	30684	29460	27724
Commd5	26730	30755	27035	28857
Commd6	22206.5	30840	31036.5	34412
Commd7	25080.5	26187	19327	21277.5
Commd8	29227	28679.5	24671.5	24652.5
Commd9	11630	12936	11851	13640
Comp	25103	12374	32287	22580
Comt	12504	509	6599	37013
Comtd1	20071	24607	21088	25948
Copa	11677	10398	9500.5	12310
Copb1	4342.5	24098.5	20967	23758
Copb2	12835.5	8698	14599.5	13849
Cope	7501	9018	12268	14305
Copg1	8723	9346.666667	6796.666667	7869.666667
Copg2	22308.5	20728.5	18258.75	10367.25
Copg2as2	26307	30304	21403.66667	35921
Coprs	11965.5	16516.5	16366	16726.5
Cops2	33929.5	15841	20125	14452
Cops3	20142	20204.5	19765	22369
Cops4	4824	6878	2958	4821
Cops5	11855	16818	15035	18290
Cops6	12758	16981	15014	15621
Cops7a	12854	12682.5	9854.5	10418.5
Cops7b	19402	22520.5	22113	22128
Cops8	31219	24990	24733	30107
Copz1	14850.5	9986	9941	9582.5
Copz2	30676.5	29926	25275.5	24448
Coq10a	40178	21496	18855	7312
Coq10b	16093	19081	17245.66667	22524.66667
Coq2	37752	35636	34148	31876
Coq3	12544	14439	15403	19430
Coq4	11159.5	12992	11343.5	11669.5
Coq5	12485.66667	9670.666667	8083.666667	8446.333333
Coq6	33847	38502	39192	85
Coq7	17201.5	26511	15757.5	24824.5
Coq9	11539	11186	9145	8887
Corin	40465	31253	7680	3488
Coro1a	19994.5	9538	18011	28266.5

Coro1b	2894	4997	901	5615
Coro1c	5029	39999	3851	40542
Coro2a	20518.66667	15536.33333	18213.66667	25127
Coro2b	25209	23506.5	25365.5	22692.5
Coro6	31933	6569	36004	39638
Coro7	25687.5	23173.5	25824	4350.5
Cort	11233	11443	17038	18154
Cotl1	9192	34963	11070.5	21210.5
Cox10	17742.5	18610	35127.5	22537.5
Cox11	18458	21913	19912	21075
Cox14	16258	18481	18512	16713
Cox15	4522	4352	5186	6140
Cox16	26311	17927.33333	27918	27326.66667
Cox17	17109	22753	19626	22823
Cox18	9755.5	13326.5	8423	9476
Cox19	21773.5	27593	23101	24873.5
Cox20	21572	22607	22689	20040
Cox4i1	1555	3993	6278	7067
Cox4i2	20162	24220	20179	24935
Cox5a	31831	7191.5	22881	7044.5
Cox5b	27454	31347	27979	31439
Cox6a1	9188	13670	15922	17394
Cox6a2	34428	6048	38495	7163
Cox6b1	11507	14517	3993	6377
Cox6b2	15148	17680	20072	20207
Cox6c	19034	23388	21792	21736
Cox7a1	35665	36417	2513	36375
Cox7a2	9727	13138	5802.5	10116
Cox7a2l	11249	11251	6885	7037
Cox7b	5477	13963	11333	13651
Cox7b2	30657	32272	35488	40672
Cox7c	4709	12721	11748	13590
Cox8a	37908	481	2116	4262
Cox8b	8998	38578	9659	6394
Cox8c	33788	25669	1928	34897
Cp	2009	12824	3971	15558
Cpa1	33983	37459	982	1290
Cpa2	21787	33119	25137	32953
Cpa3	19907	23557	11151	18109

Cpa4	14047	16163	24643	28898
Cpa5	27081	28528	29921	28655
Cpa6	30903.5	31961	33081	33572.5
Cpb1	34478	34393	40845	39952
Cpb2	19304	15393	30116.66667	20141.66667
Cpd	9588	15731.33333	23480.33333	26223
Cpe	27314.5	22379	29628	29117
Cpeb1	31453	18513	23487	23069
Cpeb2	24017.66667	12845	19900.66667	27312.33333
Cpeb3	15658	19508	17570.5	22262.5
Cpeb4	27031.5	19836.5	22311.75	23342.5
Cped1	22346.2	28570.4	24544.4	17489.8
Cphx	34511	31872	4904	35093
Cplx1	7177	6729	20652	23816
Cplx2	23913.25	13885.5	22891.5	18541.25
Cplx3	10712	14607.5	18354.5	19357
Cplx4	26814	27402	4668	6891
Cpm	15619.66667	18353.66667	29377.33333	29916.33333
Cpn1	24930.5	28640	17303.5	17676.5
Cpn2	825	32145	11647	2047
Cpne1	14770.5	13470	14380.5	13659.5
Cpne2	20826	196	26481	4956
Cpne3	27932.66667	21786.66667	19020	15067.33333
Cpne4	17100	6437.666667	27939.66667	15339
Cpne5	19143	28244.66667	26122.66667	36398
Cpne6	17868	23905	38520	1239
Cpne7	17270	23679	32254	36585
Cpne8	11560.5	10741.5	23706	22141.5
Cpne9	20566	19138	23227	24611
Cpox	5567	5891	4923	4868
Cpped1	16250	17172	14178	16536
Cpq	22534	21787	25015	24932
Cps1	26716	31354	30028	33771
Cpsf1	20994	21749	19441	19171
Cpsf2	15951	17366.66667	13476	27323.66667
Cpsf3	21056.66667	32821.33333	29248.33333	32203.66667
Cpsf3l	15268	20162	18647	22260
Cpsf4	13077.66667	14210.66667	17245.33333	17467
Cpsf4l	11081	30803.5	14981	16290

Cpsf6	20025.25	18710.5	17593.25	10435.5
Cpsf7	34991	24822	29705	28582
Cpt1a	39217.5	28800.5	40043.5	28825.5
Cpt1b	14367	15181	18199.5	20223.5
Cpt1c	5812.5	3827	22740	7083
Cpt2	34360	24760	25545	24415
Cptp	36070	36141	30270	32528
Cpvl	25596	27199	2410	39802
Cpxm1	2657	36817	40973	237
Cpxm2	13521.33333	12544.66667	14778.66667	7061
Cpz	29863	29725.5	30683.5	31047.5
Cr1l	20291.66667	11979.33333	10440.33333	11757
Cr2	18968	37380.5	38030.5	17319
Crabp1	10538	17080	18575	13964
Crabp2	32016	33825	35683	34573
Cracr2b	34651	22924	31700	20675
Cradd	10177	11447	8182	4845
Cramp1l	13989.66667	11005.66667	13953.33333	13701.33333
Crat	3014	35010	28084	22708
Crb1	20622.25	20979.25	32664.75	23599
Crb2	31021	20173	29682	26089
Crb3	32522	33699	39773	34504
Crbn	36790.33333	22989.33333	30663.33333	21435
Crcp	21326	28092	28728	34276
Crct1	7608	10456	13118	17283
Creb1	18256	19509.33333	18348.66667	18285.66667
Creb3	2388	3078	4507	3177
Creb3l1	382	41120	39768	54
Creb3l2	11850	30462.5	16211.5	14689.5
Creb3l3	15085	5448	14191	22266
Creb3l4	19287	19360	19782	19828
Creb5	13852	20919.66667	23370.66667	10710.33333
Crebbp	17807	11726.5	17715	15223.75
Crebl2	20596	19734.5	33279	29979.5
Crebrf	12808	8353	27427.5	26742.5
Crebzf	17107	18703	17309	15743
Creg1	17321.66667	9134.666667	5952.333333	10693.33333
Creg2	28074	31852.5	23032.5	25834.5
Creld1	17429	19814	13965	17632

Creld2	12463	16846	14459	18953
Crem	23975.5	23167.66667	23458.16667	21421.16667
Crhbp	17442	9828	14688	18918
Crhr1	19898.5	24299	7494.5	8520
Crhr2	2955	4168	5408	6317
Crim1	21276	28314	19809.5	28787
Crip1	35081	23852	37272.5	26034.5
Crip2	4054	1833	4522	7490
Crip3	20870	22067	24786	29976
Cript	16867	21269	19652	23200.5
Crisp1	28002	29355	31679	30163
Crisp2	31747	28830	29999	29006
Crisp3	28595	28569	16086	12530
Crisp4	11014	3432	13359	11284
Crispld1	4211	11529	16307	21367
Crispld2	26263	35811	28815	35272
Crk	29798.25	27679.5	20078.5	29675.75
Crkl	14202.33333	38339.66667	12132	14465.66667
Crlf1	15522	11028	15395.5	14151.5
Crlf2	33908	27054	31643	34177
Crlf3	26426	10011.5	7211.5	10584
Crls1	6674.5	8410	26582	27016
Crmp1	39501	1339	7124	15555
Crnkl1	18960	21713	20057	23822
Crocc	38333	32966	36693	30757
Crocc2	26693	27719	30589	29204
Crot	18943	17913	17649	17224
Crp	11067	16612	25306	24945
Crtac1	17564	22664.5	25737.5	26795
Crtam	14313	14006	19147	22737
Crtap	11149	5889	8417	4310
Crtc1	15965	14985	25054	24004
Crtc2	23308	20277	23038	22513
Crtc3	27445	23553	26261	26155
Crx	574	660	28927	27650
Crxos1	15629	10495	19193	15962
Cry1	24753	23838.5	21988.5	29387
Cry2	16241	13472	16949	18333
Cryaa	14081.5	14164	20641	25478.5

Cryab	24101	22738	27121	33716
Cryba1	17172	10817	14697	9562
Cryba2	136	4145	2030	1200
Cryba4	13639	12391	9072	9077
Crybb1	21515.5	17369.5	6686.5	22115
Crybb2	18406	20279	23031	25053
Crybb3	35529	36658	4468	2942
Crybg3	33691	23813	31183	24804
Cryga	39420	2130	4412	4151
Crygc	20425.5	19916.5	28050.5	27468
Crygd	7957	8849	14444	14699
Cryge	16729	20692.5	34052.5	762.5
Crygf	5234	7904	10721	10013
Crygn	11178	13979	11008	18421
Crygs	28746	33306	38025	35849
Cryl1	11399	5982	11845.5	8115
Crym	28539	35723	36363	37650
Cryz	10838	11988	6759	6015
Cryzl1	13997.5	15671.5	19992	14877.5
Cs	12206	12536	32927.5	16037
Csad	12243.5	20586	19741.5	5473
Csdc2	39618	4003	41060	3395
Csde1	21524.5	21462.75	17516	18615.75
Cse1l	26090	28426.5	24523	28502
Csf1	27254	40736	35098	6850
Csf1r	17951.33333	22120.66667	27752.66667	25509.66667
Csf2	38852	1987	38099	2514
Csf2ra	38971	37004	9	805
Csf2rb	23600	7021	19681.5	23962
Csf2rb2	33615	26208	38193	36612
Csf3	35920	9418	8073	14457
Csf3r	29011	21538	19334	14924
Csgalnact1	29127	27998	30919	35390
Csgalnact2	34538	26761	37907	30036
Csk	14538	9248.5	10678.5	5831
Csl	3139	5187	5588	11374
Csmd1	23879.16667	32548	20983.16667	20935.33333
Csmd2	4208	31965	8514	5159
Csmd3	28595	34612.75	22816.5	21966.75

Csn1s1	18847.5	11691.5	11713.5	13592.5
Csn1s2a	28871	30764	30781	29391
Csn1s2b	31446	31780	32953.5	28344.5
Csn2	26888	28332	30184	37918
Csn3	22034	21323	24374	22752
Csnk1a1	21133	16054.6	15365.6	14511.2
Csnk1d	18641.25	15174.5	15718.5	15503.25
Csnk1e	28365.5	23693	7643.5	4915
Csnk1g1	17002	14610.33333	11780.66667	15059.33333
Csnk1g2	36287	30740.5	28978	25668.5
Csnk1g3	28063.66667	17754.66667	26619	29135.33333
Csnk2a1	17687.66667	18775.33333	17667.33333	21581.66667
Csnk2a2	26146.33333	22055.33333	24162.33333	23652
Csnk2b	17838	16709	14763	15534
Csnka2ip	40940	4698	36148	2174
Cspg4	10255.33333	14132.33333	22571.66667	6989.666667
Cspg5	10857.5	9274	11361	28484.5
Cspp1	15616	11270	19384.8	19699.4
Csprs	16480	23547	21705	23958
Csrnp1	16587	16704	19921.66667	20875
Csrnp2	25266.5	27121	22938.5	24446
Csrnp3	28573.75	21346.25	16261	18844.25
Csrp1	30775	28960	21332	20575
Csrp2	20963	12530.5	23462	11935.5
Csrp2bp	38388.5	36458	37414.5	34834.5
Csrp3	36598	30672	369	6486
Cst10	10494	14683	19937	23313
Cst11	13320	14334	28993	29776
Cst12	28102	24684	31717	33233
Cst13	28625	31644	1508	37564
Cst3	2262	39296	7328	2271
Cst6	32607	19227	17023.5	15985
Cst7	30365.5	25219.5	29775.5	21219
Cst8	23873	29601	26811	33404
Cst9	8268	11128	17368	12692
Csta1	312	36059	34017	349
Cstad	17883	14342	18773	7918
Cstb	25086	10311.5	26544	14543.5
Cstf1	27154.5	27364	27072.5	28122

Cstf2	22877.66667	19874.66667	20063	21354
Cstf2t	39610	38111	38955	37295
Cstf3	20047.66667	28894.66667	26177.33333	28142.33333
Cstl1	3232	34988	4421	1895
Ctag2	32296	33407	36732	4840
Ctage5	12907.5	9070	25195	21401.5
Ctbp1	11492	8757	7781	5718
Ctbp2	9438	27637	9144	28751.5
Ctbs	13181.5	16711	18555.5	19816
Ctc1	7236.5	21601	8269	11041.5
Ctcf	21350	20122	22182	22809
Ctdnep1	4188	5052	5039	4369
Ctdp1	19131	20194	19534.5	18455
Ctdsp1	17177	16420.5	11473	13367
Ctdsp2	25689	22243	24108.25	23043.5
Ctdspl	35976	29554	29673	28914
Ctdspl2	22392.66667	24472	18402.83333	17502.33333
Ctf1	22145	27070	23339	23623
Ctf2	36970	36697	114	1273
Ctgf	5050	11943	17187	30697
Cth	39457	7671	11998	18048
Cthrc1	28654.5	15197.5	36447.5	5619
CTIF	29498	26578	28017	26879
Ctla2a	28572	37271	11400	13593
Ctla2b	20002	25055	2938	5928
Ctla4	1420	31738	33250	3576
Ctnna1	28218.5	19918	23145	22008.5
Ctnna2	19431.25	19700.5	21723.25	22042.75
Ctnna3	31949.66667	21686	33652	33526.33333
Ctnnal1	25486	20562.25	9665.25	17922.25
Ctnnb1	19063	19206.66667	16838.33333	21596
Ctnnbip1	15272	10569	12288	8133
Ctnnbl1	23499.5	22371.5	21512	20560.5
Ctnnd1	16784.33333	18308	21482	20873.66667
Ctnnd2	26281.5	24376.5	26468.75	26700.75
Ctns	1906	40166	40452	37364
Ctps	6235	12455	10928	16823
Ctps2	12831	12219	9879	10932
Ctr9	11502	10865	15711	14825

Ctrb1	20787	24388	24344	28541
Ctrc	12154	16159	20358	22643
Ctrl	7741	10629	12404	14538
Cts3	27659	29057	30237	33707
Cts6	3524	8410	3819	2785
Cts7	25209	24585	35167	25781
Cts8	26883	28592	28783	27615
Ctsa	9572.333333	17750	8922	8036
Ctsb	12299	19388.5	10484	21552.5
Ctsc	3721	19367.5	18106	17777.5
Ctsd	14857	21870.5	11502	27707.5
Ctse	7123	4966	7193	5353
Ctsf	24986	20488	27022	24772
Ctsg	15852	15503	18457	16259
Ctsh	5324	39095	40237	30669
Ctsj	1624	33962	32045	30553
Ctsk	19288	23840	26007	37632
Ctsl	11351	3353	16352	6622
Ctsll3	33934	2599	32289	2191
Ctsm	8313	14082	16489	18654
Ctso	4322	646	2825	40960
Ctsq	26480	27889	29432	28372
Ctsr	18808	18865	25222	25599
Ctss	12179	7127	14861	12579
Ctsw	8655	9049	17470	19546
Ctsz	8484.5	11725.5	8876.5	15263.5
Cttn	31400.5	15784.5	19924.5	24404.5
Cttnbp2	30346.5	31434	25132.5	29307.5
Cttnbp2nl	19368.33333	28129	18816.33333	15334
Ctu1	40267	2275	1305	602
Ctu2	10084	10911.5	8270	25859.5
Ctxn1	23166	21737	27911	27477
Cubn	24152.5	25553.5	25389.5	21821.5
Cuedc1	16940.33333	17875.33333	21276	20774.66667
Cuedc2	20075.5	21148.5	21385.5	22490
Cul1	22137.33333	21393.66667	19496.66667	20745.66667
Cul2	8420.5	8981.5	28175	11768
Cul3	20198.33333	30430.33333	31711.33333	18324
Cul4a	34592	34576.33333	31989.66667	19568.66667

Cul4b	22500	19784	23384	21123
Cul5	20747.5	18055.75	15878	14925.5
Cul7	7681.666667	16991.66667	19182	19089
Cul9	22258	23760	22290	24835
Cuta	38571	39069	37866	37882
Cutc	27958.5	10207.5	9961.5	29388.5
Cux1	23655.6	16370.6	20699	18319.6
Cux2	26419.5	27698.5	29481	28296.5
Cuzd1	23527	34990	26855	27294
Cwc15	24923.66667	27805.33333	28166.33333	29193.66667
CWC15	4068	6067	3562	5427
Cwc22	14153	12093	12659	11215
Cwc25	26251	8955	8115.5	7628
Cwc27	13186	8476.5	9110	12749
Cwf19l1	24722.66667	36386.33333	33638	35752.33333
Cwf19l2	22086.25	15015.25	9611.25	9462
Cwh43	2843	6313	8298	8888
Cx3cl1	24012.5	7688.5	28610	23628.5
Cx3cr1	16550	27065.5	21715	25036.5
Cxadr	32744	19159.4	29338.4	27473.6
Cxcl1	39977	19719	17784	35520
Cxcl10	28086	22911	34252	20670
Cxcl11	32890	34431	35588	33755
Cxcl12	27584.5	17279	6433.5	13217
Cxcl13	15846	7976	21233	7928
Cxcl14	1544	34704	6214	1850
Cxcl15	30799.5	31868.5	16260.5	17841
Cxcl16	25275	6717.5	13344.5	11347.5
Cxcl17	37876	37763	13450	8380
Cxcl2	39130	20470	2980	24853
Cxcl3	40705	35065	38388	13503
Cxcl5	38316	39279	11504	10321
Cxcl9	8037	1930	20270	34797
Cxcr1	2154	12124	2164	12877
Cxcr2	18890	30502	6497.5	9145
Cxcr3	23111	2866	13488	3529
Cxcr4	16939	14406	16203.5	13677.5
Cxcr5	7169	13079	16532	20465
Cxcr6	23720	5867	4135	15644

Cxx1b	40867	40859	39275	36318
Cxx1c	638	1503	1525	259
Cxxc1	14921	14673	13495	13820
Cxxc4	32704.5	28915	17341	27499
Cxxc5	17484.5	14122	19355	18124
Cyb561	14907	3569	10373	3141
Cyb561a3	20343	22203	18663	20976
Cyb561d1	12100	11121	12818	10927
Cyb561d2	20774	22149	19013	18315
Cyb5a	21554	30876	22881	36246
Cyb5b	11367	25183	35122.66667	39070
Cyb5d1	11087	8005	11959	5760
Cyb5r1	9697	27094.5	7020.5	23695.5
Cyb5r2	14495	9804	20517	19743
Cyb5r3	26072.5	8070.5	25990	26061.5
Cyb5r4	8244.666667	9713.666667	21454.33333	20042.66667
Cyb5rl	37834	37039	36930	38175
Cyba	40261	1215	29064	39101
Cybb	7578	28950	8951.5	29515
Cybrd1	37086	36369.5	18381	19122.5
Cyc1	9135	6923	4122	8684
Cycs	22519.33333	30042.66667	21399	27359.66667
Cyct	5130	6640	12536	12261
Cyfip1	17080.5	15591	19356	20356.5
Cyfip2	24427.25	18182.75	20441	16036.5
Cygb	2243	5102	2257	3485
Cyhr1	27243.75	27472.5	24737	27061.25
Cylc1	5421	40412	25505	23068
Cylc2	28663	25013	27223	26062
Cyld	6556	5927.666667	16399.66667	4388.333333
Cym	7423	5624	15043	11218
Cyp11a1	22012.5	20876	19233	7865
Cyp11b2	31423	29110	35520	39583
Cyp17a1	30222	27018	34263	31385
Cyp19a1	15107.5	18963.5	19124	18954.5
Cyp1a1	29946	32369	35711	29580
Cyp1a2	22334	21181.5	30713.5	28539.5
Cyp1b1	26125	14638.5	11778.5	23287
Cyp20a1	9001	13093	10858	11261

Cyp21a1	3404	7944	10472	9334
Cyp24a1	3707	11942	14789	12316
Cyp26a1	23412	18006	23205	22719
Cyp26b1	19078	18752	36655	32628
Cyp27a1	9079	38822	1896	26757
Cyp27b1	38194	21799.5	21465	21856
Cyp2a12	31601	35683	34780	33448
Cyp2a4	16205	24268	24079.5	7958
Cyp2a5	17924.5	34750.5	1652.5	4634.5
Cyp2ab1	2824	24861	6321	32333
Cyp2b10	25440.5	29028	32002.5	31236.5
Cyp2b13	29935.5	30599.5	23276.5	19160.5
Cyp2b19	23094.5	8892	17397	15561
Cyp2b28-ps	17076	19661	22521	23027
Cyp2b9	25830	8988	8467	9367
Cyp2c29	17745	18411	23389	24464
Cyp2c37	21489	22523.5	9649	10317
Cyp2c38	30971.5	32358	34185.5	18266
Cyp2c39	6636	3832	6854	39401
Cyp2c40	20665	9689	21967.5	22132.5
Cyp2c44	33388	2473	9213	11809
Cyp2c54	1761	25510	27871	26693
Cyp2c55	14033	12839	27904	24594
Cyp2c65	29470	30233	35463	34820
Cyp2c66	20473	21835	36961	37898
Cyp2c70	18949	19491	19536	21304
Cyp2d10	36999	19080.5	2649.5	21455
Cyp2d12	14706	14926	29505	36863
Cyp2d13	10417	2931	39454	2750
Cyp2d22	21854.33333	26995.33333	25566	22582
Cyp2d26	4985	6512	10713	10237
Cyp2d34	34067	34130	33780	32065
Cyp2d9	9858.5	9890	18444.5	17486
Cyp2e1	2879	1197	845	35846
Cyp2f2	2075	41074	34924	36870
Cyp2g1	30979	36669.5	18915.5	34247.5
Cyp2j11	20419.5	22506	26108.5	28992
Cyp2j12	5687	10897	11929	13253
Cyp2j13	26094	25995	20980.5	21398

Cyp2j5	16299	15867.5	17090.5	34064.5
Cyp2j6	33603.33333	18141.66667	33340.33333	12040
Cyp2j9	40316	22332	39313	23635
Cyp2r1	15380	19338	14176	13291
Cyp2s1	22718	19006	25652.5	25872.5
Cyp2u1	36067	3147	40604	5845
Cyp2w1	4420	7358	8529	10164
Cyp39a1	29643.5	16918	19019	19255.5
Cyp3a11	10419	15738	18752	16275
Cyp3a13	710	6503	228	39285
Cyp3a16	15903	14777	20684	19463
Cyp3a25	31908	32193.5	15412	15666.5
Cyp3a41a	13481	15164	18028	18263
Cyp3a44	30929.5	32545	36540.5	19580.5
Cyp46a1	16846	20869	21143	24089
Cyp4a10	13509.75	14618.25	14272	21860
Cyp4a12b	17700.5	20846.5	22290	23241
Cyp4a14	3016	6319	6735	8125
Cyp4a29	33382	7446	35995	3920
Cyp4b1	24556	25734	27505	26551
Cyp4f13	19149	18577	18091	19276
Cyp4f14	40211	31613	1299	34938
Cyp4f15	36403	40839	36328	1595
Cyp4f16	13717.5	13087.5	12697	13585
Cyp4f18	5805	22213	35208	16640
Cyp4f39	16332	14910	15463	12923
Cyp4f41-ps	13864	6536	7212	12054
Cyp4v3	5098	5079	39365	2037
Cyp4x1	7538	9488	13005	12957
Cyp51	17039.2	17986.6	20613	12448.2
Cyp7a1	23764	28022.5	15245.5	16698.5
Cyp7b1	20849	37326	30739	7152
Cyp8b1	30352	31921	33439	31991
Cypt1	29496	37061	33106	36203
Cypt12	38091	1254	34716	37787
Cypt15	2101	2313	27233	26152
Cypt2	29071	33634	5604	35072
Cypt3	36644	36822	3021	2670
Cypt4	1659	7220	7813	8950

Cyr61	4392	9174	10010	20003
Cys1	31135	33374	33677	31970
Cysltr1	32956.5	31772	24942	25987
Cysltr2	6974	2307	9701	4578
Cysrt1	18380.5	20965	24444.5	23348
Cystm1	1386	4040	10666	12515
Cyth1	30055	28045.5	12118.5	28261
Cyth2	15271.5	14772.5	15263.5	15117.5
Cyth3	17731.5	16383.5	14367.5	13382.5
Cyth4	16173.25	16116.75	9116.75	31895.5
Cytip	15317.33333	12224	16366.33333	11926.66667
Cytl1	1336	1440	19442	23435
Cyyr1	34407	38947	33322	31681
CZ594907	40417	7184	4733	6168
D00570	35645.5	17192	16938.5	16043.5
D030004A10Rik	29717	31308	32107	30738
D030005H02Rik	717	7972	17587	13286
D030020J04Rik	23771	24916	26514	25550
D030025E07Rik	25072	26381	28321	34506
D030028A08Rik	40812	39770	38359	35197
D030028M11Rik	29360	31039	40934	37251
D030029J20Rik	35189	24161	34394	25624
D030034A15Rik	23361	24451	26272	25177
D030035A18Rik	25560	8488	15638	12641
D030036P13Rik	26285	26017	28269	27094
D030044L04Rik	32144	33436	37068	39565
D030051J21Rik	38849	36829	5282	6785
D030056L22Rik	17795	20167	21308	21428
D030059C06Rik	28658.5	33410.5	32841	30590
D030063E12	35051	27365	32052	29808
D030068K23Rik	19968.5	37125	20665.5	24734
D030074K08Rik	29958.5	28847.5	30206	28977.5
D10Ertd755e	29145	29112	30646	29249
D10Jhu81e	19461.5	20658.5	16239.5	35751
D10Wsu102e	35176	27106	24304	27811
D11Wsu47e	12981	14643	12045	14186
D130004A15Rik	30651	32283	33785	32434
D130009B15Rik	29999	31579	33867	32172
D130009I18Rik	12033	21597.66667	13088.33333	18080

D130012P04Rik	33140	39277	36941	4331
D130017N08Rik	8789	12190	15744	11435
D130020G16Rik	6478.5	6634	25641.5	27625.5
D130020L05Rik	16552.33333	23792.33333	17846.33333	22051.33333
D130039L10Rik	25874	27535	32796	40882
D130040H23Rik	19280	38381	18358	35408
D130043K22Rik	35651	18813	20960	4818.5
D130051D11Rik	12959	5945	16187	16956
D130052B06Rik	1715	5978	17910	5334
D130058E03	36281	8390	16982	5665
D130060J02Rik	4613	3547	21092	16804
D130060J10Rik	26954	33211	30490	29183
D130061D10Rik	5369	7598	9067	8608
D130062J10Rik	9332	40285	40788	10302
D130067P18Rik	29781	31779	31607	30187
D130076A03Rik	14359	6731	16087	11342
D130079A08Rik	32192.5	25115.5	24290	33845
D130084N16Rik	3507	32854	36280	36532
D130095D21Rik	34277	28509	34129	39829
D14Mgi8	597	7883	16535	19634
D15Ertd621e	12711	12755.33333	10418.66667	12754.66667
D16Ertd472e	17476.75	18007.75	24407.25	19519.5
D16Ertd519e	30715	32347	34299	33742
D17H6S53E	35845	36428	34848	34156
D17H6S56E-5	16230	19672.5	13457.5	32862.5
D17Wsu92e	18955.5	19650.5	16381.5	22997.5
D18Ertd232e	24599	1991	34390	698
D1Ertd622e	31590	26347	23935	24817
D1Pas1	33277	38663	35558.5	35134
D230002A01Rik	20774.66667	19949	36883	34077.33333
D230014I24Rik	37480	39077	39240	35512
D230015J17Rik	31323.5	25611.5	29728	32559.5
D230019N24Rik	26316	28076	27861	271
D230021J17Rik	38944	11318	6397	9998
D230025D16Rik	4765	680	33734	38991
D230034L24Rik	27318	28783	30061	29415
D230035N22Rik	22693	15959	16989	15787
D230038C21	2486	35088	2279	38666
D230040A04Rik	18320	20384	20815	21078.5

D230040N21Rik	26953	2099	31140	38527
D230044P21Rik	24565.5	23463.5	29798.5	26607.5
D2hgdh	4761.5	19495	36306	14881
D2Wsu81e	39099	989.5	37190.5	19117
D330005C11Rik	39547	7205	29913	40163
D330013E07Rik	7460	6806	12048	13060
D330022K07Rik	26686	27972.5	29455.5	29806
D330023I04Rik	31958	33229	36997	4877
D330040H18Rik	29864	13372	18138	11295
D330041B21Rik	20908	20158	33708	28862
D330041H03Rik	27272	29008	30188	36492
D330045A20Rik	10796	23866	15694	27599
D330050I16Rik	40060	39356	38486	39953
D3Ertd254e	10523	8297	8925	9641
D3Ertd751e	20575.66667	29591.33333	33667.66667	29743.33333
D430006K04	16847	19051	10864	16834
D430019H16Rik	18425	20386	31498	26810
D430020J02Rik	22013	12202	23520	21232
D430022A14Rik	17363	29941	36646	27113
D430030G11Rik	10038	11112	17202	21006
D430032J08Rik	9072	5229	2810	33845
D430036J16Rik	8418	17331	2888	3442
D430041D05Rik	34834	28475	10490	31596
D430042O09Rik	31236.5	25930.5	27790	25065.5
D430047D06Rik	36012	350	30789	29180
D45207	29299	38338	37501	4402
D4Ertd179e	30020	23317	23871	23734
D4Ertd681e	38168	30327	7494	3297
D530014G21Rik	18144	24493	22248	21938
D530015H24Rik	28117	29954	29718	28538
D530037P16Rik	27034	28315	30973	29498
D530049N12Rik	280	5796	20196	19380
D5Ertd577e	7082	14035	23228	23875
D5Ertd579e	18470.8	14415	10151.2	9165.8
D5Ertd798e	40373	37524	29023	27727
D630003M21Rik	16829	19288	21881	27434
D630004D15Rik	25325	26857	27862	35533
D630013G24Rik	29652	31176.5	33306.5	31639.5
D630014O11Rik	26921.5	4323.5	31016	15505.5

D630023F18Rik	6107	2249	4397	6249
D630028G08Rik	537	3570	14137	12151
D630029K05Rik	30266	29909	1308	7597
D630032N06Rik	36983	3228	1338	2925
D630033O11Rik	5702	1802	34517	26
D630039A03Rik	39057	40656	9786	10021
D630045J12Rik	36492	24797.5	34125	28452
D6Ertd160e	23639	24782	26339	25276
D6Mit97	34058	307	38728	40245
D6Wsu163e	27525.5	27625	30625	26281
D730001G18Rik	15979	23311	23550	24678
D730039F16Rik	29245	29776	39176	34111
D730045B01Rik	32215.5	34130	35355	34955
D730047E02Rik	39141	29296	40818	36583
D730048I06Rik	29284	30896	36469	30495
D730050B12Rik	5311	4876	5873	6856
D7Ertd413e	22498	8431	3447	6277
D7Ertd443e	34942.66667	26801.33333	22437.33333	13699.33333
D7Ertd715e	5787	9420	14259	12768
D830012I24Rik	707	23733	26558	23130
D830013E24Rik	30526	32069	33669	31934
D830013H23Rik	29824	31476	2293	40210
D830014E11Rik	14090	16853	18922	23191
D830016O14Rik	39138	34525	34114	32486
D830024N08Rik	18523	18894	19866	22115
D830026I12Rik	26948.5	28335.5	29829	29952.5
D830029L11	14247.5	16754.5	17496.5	18139.5
D830030K20Rik	37187	39330	33176	39831
D830031N03Rik	40247	25506	34160	29043
D830039M14Rik	19414	22643	25962	23780
D830044D21Rik	14347.5	18294.5	31433.5	32575
D830046C22Rik	5419	5056	31229	29743
D8Ertd563e	34242	30704	30098	28883
D8Ertd738e	11660	9712	12787	13790
D8Ertd82e	24918.66667	22814	27169	20690.33333
D930015E06Rik	27023.33333	18873	19095	23206.33333
D930016D06Rik	12168	7163.5	9225	10713.5
D930017F01	23567	34204	26848	25753
D930017J03Rik	19251	4538	7621	5915

D930020B18Rik	14724.5	34551.5	2846.5	22259.5
D930021H04Rik	21294	14404	17762	19636
D930021N14	10439	21058	11586	13869
D930030D11Rik	17794	21874	22409	28030
D930030K17Rik	28177	29425	31364	30006
D930030O05Rik	30160	32073	32236	30804
D930031A20Rik	40643	1986	9748	9356
D930033H10Rik	40133.5	18283.5	15307	21261.5
D930043N17Rik	30669	23985	17020	20806
D930043O14Rik	31879	13716	27226	26159.5
D930044I17Rik	13291.5	33911.5	20089	15842.5
D930046H04Rik	30034.5	23288	28165	27123
D930048N14Rik	39337	35453	22710	24962
D930049A15Rik	34395	38779	4315	5452
Daam1	21189	24721.75	23478.75	28108.25
Daam2	19275.66667	21312	27093.66667	26462.33333
Dab1	22545	19847.6	23741	21373.2
Dab2	24971.33333	29906.66667	27718.66667	22307.33333
Dab2ip	13650	15247	20967	22552
Dach1	38445	27092	19704	34379.5
Dach2	20194.25	21126	23227.5	26065.75
Dact1	8076.5	23658	16558.5	20770.5
Dact2	20040.5	11238	23295.5	15507.5
Dad1	5629	4277	132	1280
Dag1	14399	20098.5	22340	12173.5
Daglb	13062.5	18975.5	28096	16346.5
Dalrd3	18941.5	15179	12131.5	11613
Dand5	40708	1785	2572	1862
Dao	8730	10694	17521	21554
Dap	3844.5	4108	22533.5	4219
Dap3	19345.5	18725.5	16317.5	18575
Dapk1	22577.33333	15855.33333	9769	20961.66667
Dapk2	36611	40798	40636	27065
Dapk3	35475	40491	30278	38408
Dapl1	39742	17547	8705	4690
Dapp1	13847	15193.66667	16565.33333	14631
DarkCorner	36408	38297	38609	37238
Dars	21707.5	25976.25	25989	27111.25
Dars2	39228	37614	34414	34850

Daw1	27087.5	26714.5	19336.5	15145.5
Daxx	6339	7977	9338	13130
Dazap1	29525	27272	26794	32160
Dazap2	3880	40493	3834	2131
Dazl	20893.5	22049.5	23971.5	27476
Dbf4	25817	26304	28469	24442
Dbh	38266	39040	1876	5281
Dbi	26289	24845	26104	23144
Dbil5	7895	14370	13938	11394
Dbn1	13532	16167	21236	30074
Dbndd1	23232	32207	39887	947
Dbndd2	9897.666667	32675.33333	34349	32033.33333
Dbnl	23321.33333	22957	13083.33333	27235
Dbp	7765	31805	8335	34172
Dbpht1	30645	35480	36669	32787
Dbpht2	11384	15052	35155	23904
Dbr1	40424	3085	5224	6365
Dbt	22553	21173	22050	23355
Dbx1	28476	227	34591	31188
Dbx2	34233	20390	34119.5	34258.5
Dcaf10	16747	12979.25	20705	19083.25
Dcaf11	11240	9627	8316	9251
Dcaf12	14174	27755.33333	25456.33333	29460.66667
Dcaf12l1	28481	26550	6910	2706
Dcaf12l2	17574	19558.5	19751.5	21262.5
Dcaf13	15414	19175	17684	24006
Dcaf15	5694	3798	5837	2812
Dcaf17	15718.5	15212	14598.5	14564.5
Dcaf4	30167	26564	24280	23887
Dcaf5	31231	30188.5	31525.5	31441
Dcaf6	14275.5	17084.5	21478	21656.5
Dcaf7	16644	34137.5	32736	33925.5
Dcaf8	22608.25	22593.5	22498.5	22061
Dcakd	30611.5	27553.5	11230.5	28592.5
Dcbld1	20433.5	26151	31034.5	31686.5
Dcbld2	15887	32378	19779	16577.5
Dcc	25545.4	23786.6	28595.4	24259.6
Dcdc2a	14885	18967.66667	11888	22181.66667
Dcdc2c	37367	30799	1707	37037

Dcdc5	24456	29134	528	38259
Dchs1	10494.5	6725.5	27825.5	23252.5
Dchs2	34499	8041	32444	32253
Dck	26848.33333	10126	24846.33333	16087.33333
Dclk1	20920.6	18796.8	21416.8	14926.6
Dclk2	13312	14380.5	15290	14121
Dclk3	3555	8986	11218	9401
Dclre1a	38184.5	31521	29790.5	27905
Dclre1b	18003	17915	16410	17584
Dclre1c	20385	24065.25	22474	26315.75
Dcn	15396.5	19870	21198	21613.5
DCP_1_0	3633	39282	4154	3473
DCP_1_1	40098	34520	310	40658
DCP_1_11	548	39629	35249	35861
DCP_1_2	33710	24012	33452	33947
DCP_1_4	19127	16058	20395	20596
DCP_1_7	4618	1741	5591	4990
Dcp1a	20176	18474	20914	20453
Dcp1b	15482.5	9506	27741	29620
Dcp2	18978	23983	18941.5	24356
DCP_20_0	34272	21802	30755	28277
DCP_20_1	30966	20244	26990	25420
DCP_20_3	20492	14418	20511	19320
DCP_20_5	4438	39105	5702	3798
DCP_20_7	39867	35168	974	41029
DCP_20_9	10798	15354	16494	16114
DCP_22_0	17106	17013	20815	24562
DCP_22_2	8868	8473	13401	16970
DCP_22_4	39238	38475	3217	6635
DCP_22_6	15208	19106	20831	24938
DCP_22_7	35236	35902	1290	6716
DCP_22_9	15951	15661	25169	26676
Dcpp1	32911	24553.75	29960.75	17800.5
Dcps	21798	25097	24820	23421
Dcst1	5909.666667	7924.666667	16812.33333	17809.33333
Dcst2	13006	11586	22700	26385
Dcstamp	7495	15740	2333	8492
Dct	33755.5	38958.5	17603.5	36346.5
Dctd	27270	30747	20049	23908.5

Dctn1	38205	34615	34752	26131
Dctn2	4538	3859	3589	3527
Dctn3	19868	24078	25039	26545
Dctn4	22338	22091	20561.33333	21727.33333
Dctn5	17693	18437.33333	20065.33333	20180.33333
Dctn6	8647	7517	8959	8406
Dctpp1	812	1037	408	37278
Dcun1d1	18542	18724.66667	18416.66667	18227
Dcun1d2	22077	19032	16995.66667	17706
Dcun1d3	15871.66667	18776	17214	22496.33333
Dcun1d4	18188.5	17464.5	12686.5	17252
Dcun1d5	30676.33333	24468.33333	21106	23536
Dcx	35362.66667	34266.33333	22883	34761.66667
Dcxr	14507.5	15597.5	16190.5	11187
Dda1	25073.33333	25598	30441.33333	37022
Ddah1	15727	24717.5	11268.5	13254.5
Ddah2	40300	1435	9494	6745
Ddb1	21524	16932	18393	21606
Ddb2	28694	20768	24349	23041
Ddc	10558	13828.5	19218.5	18576
Ddhd1	17529.5	18360.25	9685.5	17447
Ddhd2	29292	22069.66667	21035.33333	21236.33333
Ddi1	27722	29188	31137	37224
Ddi2	18932.5	7874	14537	11014.5
Ddias	27189	21203	21032	20098
Ddit3	22791	23637	25912	28501
Ddit4	27516	37733	39215	6032
Ddit4l	14441.33333	12631	22603	20155.33333
Ddn	17058	18891	23485	22603
Ddo	22461	24573.66667	23290.66667	22542.66667
Ddost	9684	8180	5365	5497
Ddr1	5715	11995	15662	23028
Ddr2	20775.5	19826.5	26279	11304
Ddrgk1	20031	17486	15223.66667	16920
Ddt	39195	4175	4375	3531
Ddx1	27940	15022.66667	26489.33333	11688.66667
Ddx10	6832.5	9025	5525	7776
Ddx11	9673	5464	40723	8226
Ddx17	13926.66667	22040	22249.66667	22300.66667

Ddx18	16838	17397	14985	15613
Ddx19a	13515.5	13953	10233.5	11552.5
Ddx19b	14746.5	16220	17252.5	17645.5
Ddx20	23977	21349	21606	21579
Ddx21	24209.66667	11854	21911.33333	8351.666667
Ddx23	6022	12548.5	24407.5	21684
Ddx24	20960.66667	20120.33333	17693.66667	7612.333333
Ddx25	29507	37619.5	20667.5	15363.5
Ddx26b	17041.25	25087.5	23764	25354.5
Ddx27	34380	36457	21769	21818.5
Ddx28	14528	12895	9846	12779
Ddx31	18725	15938	17688	16271
Ddx39	3720	5809	1405	6808
Ddx39b	9461	6879.5	8795	10455
Ddx3x	19366.33333	28601.33333	27920.66667	27059.33333
Ddx3y	14686	8797	10217	8336
Ddx4	20045	18206	19777	22534
Ddx41	8545	7444	6879	6424
Ddx42	16888.5	31015	24550	23684
Ddx46	17600.66667	17239	13104.66667	24015.33333
Ddx47	20701	17654	14346	15857
Ddx49	10615	11031	5678	7501
Ddx5	19010	13512.5	19252.5	17661.5
Ddx50	15799	12622	13395	12979.5
Ddx51	22852.5	22655	23663.5	22656.5
Ddx52	12289	12028	7926	10440
Ddx54	11626	8702	11377	10820
Ddx55	38690	37322	35425	36243
Ddx56	28136.5	28207	9543.5	9077
Ddx58	21486.25	22372	15704.25	20123
Ddx59	10095.5	10537	10895.5	11743
Ddx6	11678.5	16307.5	17420	17253
Ddx60	5363	7970.5	36042	8470
Deaf1	35268.66667	21650	20898.33333	30029
Deb1	9197	10995.5	12416	12457
Decr1	8428	7085.5	22425	17730.5
Decr2	4546	4922	4767	3129
Dedd	8760	11532	12168	12170
Dedd2	24149.25	23801.5	13302	13345.75

Def6	24515.66667	13345.33333	14344	10947.66667
Def8	23858.5	8265	20158.5	24807
Defa1	30873	32491	34067	32328
Defa12	26246	27662	29041	28098
Defa20	40808	2372	6210	6061
Defa21	27964	29278	32016	37654
Defa22	35307	2972	3096	28116
Defa23	26004	36906	29101	27842
Defa3	23828.5	7182	8741	9146
Defa4	13906	22593	20198	22253
Defa-rs1	16203	17962.5	27890	25554
Defa-rs10	33181	34446	806	1596
Defa-rs12	26350	27682	30157	241
Defa-rs2	29651	31021	33378	15577
Defa-rs7	26876	26375	34133	30855
Defb1	18566	37926	32882	36978
Defb10	6375	8956	15675	10549
Defb11	36631	181	9843	9303
Defb12	32612	34875	36005	33875
Defb13	13343	18180	18170	16919
Defb14	1207	28584	38178	37884
Defb15	25332.5	26713	28450	27058.5
Defb19	30758.5	33030.5	34879	39468.5
Defb2	7515	7919	4867	10235
Defb20	24832	26029	27818	26758
Defb22	20874	23629	27325	34595
Defb29	3119	7035	16090	19941
Defb3	20328	22144	24671	25250
Defb34	15879	16126	20812	19782
Defb35	9896	12443	15550	23295
Defb36	34676	26688	38226	38885
Defb37	15296	19319	9992	6522
Defb38	13702	20369	20049	24716
Defb39	19797	17582	23626	23390
Defb40	35431	36608	31490	3311
Defb41	29421	37488	31058	29703
Defb42	24760	25917	28003	26787
Defb5	29836	31273	32666	31209
Defb50	29601	36040	29898	30306

Defb6	29590	1941	744	2117
Defb7	25304	26548	28347	27184
Defb8	28621	30046	1291	3833
Defb9	37986	35506	36121	26835
Degs1	30806	21459	22162	22452
Degs2	1484	10123	12528	4935
Dek	21052	21641.5	36409	38038
Dennd1a	23826.5	15426.5	22247	17223
Dennd1b	12109.2	28401.4	29431.8	32383.2
Dennd1c	14622	12857	13493	12448
Dennd2a	2111	39300	4025	40418
Dennd2c	23330	15151.5	9130	4877
Dennd2d	12670	20900.5	30300.5	15188.5
Dennd3	7494.5	27796	27131	20948.5
Dennd4a	23846.4	22727	20927.2	19718.4
Dennd4b	20583.5	10696.5	17772.5	28120.5
Dennd4c	14046.2	12469.2	12725.4	24890.8
Dennd5a	29181	28496.5	26788.5	26444
Dennd5b	28274	23780	24278	31202.5
Dennd6a	19715.5	36088	37658.5	36495
Dennd6b	14970	13856	15041	13567.5
Denr	32115	17760	14475	15553
Depdc1a	37446	2264	9668	7467
Depdc1b	35268	24148	26723	31274
Depdc5	21375	19731.33333	18688.66667	17480.33333
Depdc7	20386	20509	20127	19521
Deptor	10238.33333	3256.333333	18058.66667	17766.66667
Dera	27416	31431	27895	33226
Derl1	17945.5	19607.25	20085.5	19894
Derl2	13320.5	18862.5	14537	19353.5
Derl3	31698	34003	39082	38948
Des	20096	26520	23933	35698
DESI1	39109.5	36699	30982.5	30192
Desi2	24097	23217.33333	23298.66667	22781.33333
Det1	17188	19305	19074	18520
Dexi	31551	27784	25790	24833
Dffa	19590	31762	29760.66667	28064
Dffb	5177	422	45	1494
Dfna5	18981.8	18243.6	19970.6	19963.8

Dgat1	8340.5	6574.5	3315	5905.5
Dgat2	15577.66667	21200.66667	17933.33333	22967.66667
Dgat2l6	26422	27871	30137	37400
DGCR14	23102.5	19184	20601	21351
Dgcr2	18864.33333	29161	27340.33333	26750.33333
Dgcr6	33154	36245	30540	34497
Dgcr8	13810	31421.75	23670.25	32613.75
Dgka	11916	9139	11192	6765
Dgkb	11653.16667	25598.66667	26131.66667	28923.66667
Dgkd	23801.5	12131.5	19468	12679.5
Dgke	17977	24759.5	17423.5	19317.5
Dgkg	28025.5	11968.5	2494	4347
Dgkh	24572	16445.25	22085	18611.75
Dgki	23055.75	23391.75	25097.75	24393
Dgkk	2160	37080	33678	4639
Dgkq	23810	17304.5	17634	18114.5
Dgkz	31462	27161	37312	30671
Dguok	22405	37508	24791.5	21490.5
Dhcr24	24910.5	23583	26819	27171
Dhcr7	19858	18887.5	29506	25816
Dhdds	10750.5	14588	14573.5	16784.5
Dhdh	29029	30485	32582.5	31195.5
Dhfr	24472.66667	29701.66667	17816.66667	29787.66667
Dhh	40467	36568	5547	1090
Dhodh	6276	8108	6329	9125
Dhps	37741	1730	40262	1999
Dhrs1	16183	10183	11686	10974
Dhrs11	15615	17668	15305	19160
Dhrs13	15603	20536	17533	19423
Dhrs2	39845	34073	34498	8623
Dhrs3	28836.5	28671.5	10981	10548.5
Dhrs4	6967	5227	5047	4341
Dhrs7	24970	7888.5	25791.5	24105
Dhrs7b	17026	19448	17025	18223
Dhrs7c	25554.5	8821	13773.5	12515
Dhrs9	6945	20446.5	24358.5	20777.5
Dhrsx	22635.5	23662	22440.5	22427
Dhtkd1	7525	8305.5	5346.5	24428.5
Dhx15	3739	5722	6619	8685.5

Dhx16	29459	24683.5	28744.5	27508
DHX16	21786	28269	27569	33474
Dhx29	25840.5	26272.5	16925.5	18188
Dhx30	11506	6571	10728	9672
Dhx32	28367	24175	26983	24454
Dhx33	24532.66667	24690.33333	22894	25696.33333
Dhx34	27948.66667	19795.66667	27643.33333	20461.33333
Dhx35	12639	9792	10784	9122
Dhx36	13202.33333	24232.66667	10097	9423
Dhx37	18180	17644	17116	17112
Dhx38	28696	28177	20145	21228.5
Dhx40	12082.5	9894	11893	11944
Dhx57	19933.5	18476.5	17380.5	17423.5
Dhx58	16951.5	23823	21204	32419
Dhx8	21868	22917.5	24096.5	23314
Dhx9	21217.5	12627.5	11269.5	10797.5
Diablo	16041.5	12289	9890.5	12914
Diap1	12978.5	10828	14247.5	13426.5
Diap2	9967	22114	16841	16188.8
Diap3	19360.66667	18067.66667	30755	30739
Dicer1	32149	25495.5	19782	17602
Dido1	14138.33333	24972	27522.33333	27153.83333
Diexf	33822	12326	31131	32200
Dimt1	6444	10193.5	26993	11121
Dio1	39094	38255	2670	40063
Dio2	8053	7718	12494	15607
Dio3	29	3504	1391	1556
Dio3os	23706	26882	24906	25965
Dip2a	8946.5	8998	8622	8943
Dip2b	24159.2	16636.2	18408	15427.6
Dip2c	302	39529	4580.5	20097.5
Diras1	36176	1264	41119	2591
Diras2	27555	38427	6927	7976
Dirc2	24041.66667	24993.66667	23259.33333	24131.66667
DIS3	14596.5	14210	11720.5	13829
Dis3l	12781	12888.5	11764	9641.5
Dis3l2	22375.33333	16848.33333	24206	27199.33333
Disc1	25798.66667	23482	25143.66667	12497.66667
Disp1	16979.5	13339.5	13419.5	33811.5

Disp2	12247.5	11295.5	19119.5	18160
Dixdc1	19433	23096	23866	20563.5
Dkc1	9905	11301.5	5185.5	10214
Dkk1	13864.5	23566	6784.5	11752.5
Dkk2	1090	29739	3258	39997
Dkk3	18886	12024	16883	13860
Dkk4	25471	26745	6945	1874
Dkkl1	5127	7425	9350	9353
Dlat	20542	21574	16862	19452
Dlc1	24307.2	25171.6	22950.8	28869.6
Dld	15712	6951.333333	25349.66667	3381
Dlec1	35876	24317	4759	2156
Dleu2	16550	29647.66667	14405	22189
Dleu7	23793	24944	26503	26227
Dlg1	26775.83333	24531	28617.16667	25817.5
Dlg2	27965.875	17891.375	21036.375	24177.625
Dlg4	21260	5296.5	4997	5236
Dlg5	22961.5	22164	10792.5	11392
Dlgap1	25929.8	27090.2	22444	22190.8
Dlgap2	21147.25	16202.75	18766	20860.75
Dlgap3	21542	24613	956	772
Dlgap4	3503.666667	20555.66667	32436	16235.66667
Dlgap5	9233.5	8936.5	12600	15341.5
Dlk1	15085	12266	12807.5	16098.5
Dlk2	2438	3096	8186	6888
Dll1	19832	20436	25818	26588
Dll3	15233	21370	23690	24111
Dll4	18628	18806	24435	23602
Dlst	13287	11373.66667	12330.33333	14342
Dlx1	11051	7433	13517	1327
Dlx2	4650	34009	35286	33468
Dlx3	32710	34832	1947	36281
Dlx4	25710	27008	28790	27732
Dlx5	4401	12259	12195	13006
Dlx6	30099	37544	4405	1274
Dlx6as1	25807	25567	28607	23367.5
Dlx6as2	39415	38966	3842	41120
Dmap1	28304.5	27338.5	25003.5	27496
Dmbt1	15511	19134	19807	19348

Dmbx1	19991	22431	7682.5	8539
Dmc1	28431.5	29804.5	31665	15065
Dmd	27165.90909	27603	25939.27273	27511
DMD	27667	28934	30860	29524
Dmgdh	13410	16783	23185	23764
Dmkn	24806.5	23440	25857	5369
Dmp1	15015	8776	24653	25929
Dmpk	10277.33333	7155	6704	29355.33333
Dmrt1	32141	28455	29859	28855
Dmrt2	12726	5695	1086	40598
Dmrt3	22136	28873	26630	29119
Dmrta1	25533	26822	28365	27438
Dmrta2	36150	27939	35333	28640
Dmrtb1	20438	21312.5	21424	19908.5
Dmrtc1a	29677	5631	9761	11093
Dmrtc1b	14915	11572	516	13624
Dmrtc1c1	8032	15642	14243	20704
Dmrtc2	16492	15067	14729	21487
Dmtf1	23980	4751	5825	7123.5
Dmtn	14912	16045.66667	12448.33333	8343.333333
Dmwd	10211	11390	15866	15559
Dmxl1	25757.66667	20784	22156.83333	17726.5
Dmxl2	20667.75	19752.5	23437.25	25460.75
Dna2	17871.33333	11453.33333	10157.33333	21738.66667
Dnaaf1	22755.5	22702	20383.5	2120.5
Dnaaf2	19617	20968	21878.5	21810
Dnaaf5	12094.5	11777	13386.5	13487
Dnah1	27393	18114	27186	27608.25
Dnah10	1589	880	10633	13107
Dnah11	8485	5060	16574	12635
Dnah17	9292	10287	17816	17716
Dnah2	18702.8	18117	21329.6	20792.8
Dnah5	9811	26700.66667	16388	17094.66667
DNAH6	16694.5	20143.5	35101	29796.5
Dnah7a	15921	15905	24570	32375
Dnah7b	31917	34372	34793	38726
Dnah8	22034.33333	10042.33333	12555.66667	17398.33333
DNAH9	31144.6	26014.2	26713.6	22976.4
Dnahc12	19052.33333	26836.33333	24152.33333	16473.33333

Dnahc14	5457.5	20222	21633.5	20726.5
Dnaic1	14121	15053	17987	18242
Dnaic2	6878	9014	9567	6185
Dnaja1	22919	29861.33333	25577	29980.33333
Dnaja2	34592	38318	40052	4006
Dnaja3	17814	16637.5	14667.5	17192.5
Dnaja4	4701	6995	25738.5	14438.5
Dnajb1	13940	14898.5	13037.5	14961
Dnajb11	13415	16154	15345	17194
Dnajb12	19121	18275.5	17493.5	22889
Dnajb13	16678	21660.5	20822	16261.5
Dnajb14	14201	10029	11534	6576
Dnajb2	13189	14819	13758	15430
Dnajb3	4677	5117	19305	15657
Dnajb4	18813.25	18137	21260.5	21885.75
Dnajb5	12542.66667	18795.66667	15473.66667	18324.33333
Dnajb6	28538	27967.66667	29110.66667	23404.33333
Dnajb7	32560	33794	39981	6640
Dnajb8	13196	240	25324	834
Dnajb9	23523	31049.66667	25720.33333	26801.66667
Dnajc1	31549.4	28539.8	32123.4	30013.4
Dnajc10	23026.75	20786.75	22981.75	23391.75
Dnajc11	22850.5	23194.5	2259	2345
Dnajc12	24351	24685	25217	21543
Dnajc13	23309	21369.66667	23816.66667	24538.33333
Dnajc14	11726.5	10921	30132.5	30766
Dnajc15	17377	24097	20177	24165
Dnajc16	9675	7279	9517.25	7001.75
Dnajc17	29525	11949.5	14369	15854
Dnajc18	14562.5	20231	18760.5	20286
Dnajc19	24487.66667	19097	19813	14811
Dnajc2	11601	13590	7292	10646.5
Dnajc21	8880	14570	12037	16294
Dnajc22	3822	13117	21511	20644
Dnajc24	8295	15253	14954	12829
Dnajc25	4901	4186	38447	38428
Dnajc27	23861.33333	24635	23101	8422
Dnajc28	28631.66667	30549.66667	24916.66667	25743.33333
Dnajc3	24429.5	24403.5	20589.75	22752.75

Dnajc30	38169	38738	39173	38861
Dnajc4	23824	23819	24876	23234
Dnajc5	21687.33333	17598.66667	19633.66667	20357.33333
Dnajc5b	23209.5	7594	18848	17739
Dnajc5g	24781.5	28037.5	12896.5	31784
Dnajc6	37401	26224	8532	5765
Dnajc7	39696	40548	2679	663
Dnajc8	10626	11498.5	12805	14557.5
Dnajc9	35696.5	27880	33820.5	24690
Dnal1	28983	28489	26367.5	35734
Dnal4	26690	21925	23254	19867
Dnali1	14066	20769	17746	19366
Dnase1	8582	12811	18269	14635
Dnase1l1	28579	20339	28340	24566
Dnase1l2	10207	11750	17316	16865
Dnase1l3	5552	10978	14175	20258
Dnase2a	27228.33333	18559.66667	21439.66667	18193.33333
Dnase2b	36963	3992	3515	7587
Dnd1	7357	6457	6628	6244
Dner	29477	11591.5	14993.5	13353.5
Dnhd1	27052.5	16127.25	21819.25	24246.25
Dnlz	18080	20320	20860	20321.5
Dnm1	11237	10387	19657	20915
Dnm1l	14031.75	12123.5	13367.5	5732
Dnm2	22563	19168.33333	17351.33333	16795
Dnm3	31128.25	30087.75	33093	32630.25
Dnm3os	28586.75	23579.25	25641	23725
Dnmbp	7324	19661.66667	9234.333333	19950
Dnmt1	22454	20437	23858	20762
Dnmt3a	16766.33333	7576	14477.66667	7038.666667
Dnmt3b	6279	36394	2741	40170
Dnmt3l	14123	1100	22424	3694
Dnpep	24301	23993.5	20873	22874
Dnph1	15791	20223	21515	23022
Dntt	20585.66667	22453.33333	26301.66667	24509.33333
Dnttip1	657	40337	2265	2192
Dnttip2	6979	7569	8815	8408
Doc2a	7361	10091	11012	12294
Doc2b	40395	37748	37240	27516

Doc2g	28085	27404.5	24421.5	29217.5
Dock1	19392.2	19132	19792.4	19606.4
Dock10	35413.66667	31928.66667	33172.66667	24938.66667
Dock11	26448	19731.5	23743	21651.25
Dock2	24433.5	15820	25432.5	12859
Dock3	30951.5	31952	19345.5	1920.5
Dock4	22930.11111	23550.77778	23357	23748.55556
Dock5	10155	21406	15416.66667	17951.66667
Dock6	16136.4	14930.4	16974.8	16070.2
Dock7	18388.75	27295.25	26369	20835.5
Dock8	17068	21100.28571	16487	23466.57143
Dock9	20021.33333	21214.66667	27926.66667	26107
Dohh	11063	12829	12695	13162
Dok1	20281	36331	17464.5	34012.5
Dok2	23114	14275	24958	14439
Dok3	10995	9965	8882	7931
Dok4	16317	14914	18758	15304
Dok5	27816	29173	32120	39803
Dok7	36627.5	24626.5	4748	13361.5
Dolk	23909	25603	24942	27073
Dolpp1	32763	23320	25434	21052
Donson	15050.5	17492.5	10677.5	16659
Dopey1	19729.2	26948.4	13710.8	19879.6
Dopey2	18037	12764	16654.5	11773.5
Dot1l	22918.33333	14063.33333	18188	15297.66667
Dpagt1	4442	1893	1098	40553
Dpcd	23211	27381.5	22286	25209
Dpep1	39374	38491	562	40873
Dpep2	22844.5	5062	23653.5	8660.5
Dpep3	2187	8093	4629	10271
Dpf1	22477	25378	25161	26606
Dpf2	28698	25202	26057	27187
Dpf3	24013	32917.5	3616.5	9795.5
Dph1	12117.5	13094.5	7703.5	11708.5
Dph2	16263	17819	17995	19832
Dph3	31436.33333	19489	18772.66667	19225
Dph5	22799	27955	25141	27812
Dph6	23954.66667	28744.66667	25571.66667	29865.66667
Dph7	8264	10668	9481	7916

Dpm2	37415	32386	35177	34730
Dpm3	22913	28244	19753	17397
Dpp10	22487	23628.5	21849.66667	21021.66667
Dpp4	22231	20031.66667	15475.33333	35832.66667
Dpp6	18618.66667	23011	22647.33333	22143.66667
Dpp7	18533	26669.5	21825.5	28677.5
Dpp8	16530.75	13988	13698.25	12553.25
Dpp9	2517	1648	41002	39809
Dppa1	32084	33682	38353	37121
Dppa2	14345	15929	16864	18057
Dppa3	38336	20842	3022	20979
Dppa4	23836	25028	26276	31495
Dppa5a	6701.5	12315	15083.5	15083.5
Dpt	25049	291	26774	25831
Dpy19l1	22922.66667	24204.33333	18378.66667	20397.33333
Dpy19l2	31689	33318	34988	33179
Dpy19l3	26475	2296	16200	24519
Dpy30	10387.5	14720.5	10805.5	29261.5
Dpyd	22804	24173	25563	27286
Dpys	21389.5	7700.5	19494.5	9196
Dpysl2	25508	26210.5	24339	21613.5
Dpysl3	7615	4317	9608	9188
Dpysl4	28784	30176	31661	31913
Dpysl5	28971.5	34312.5	36121	21803.5
Dqx1	21224	18088	25557	19287
Dr1	30357	31067	28917.5	30684
Dram1	33913	40887	24285	38309
Dram2	5207.666667	19824	16224.66667	17027.66667
Drap1	34596	35868	36572	33097
Draxin	19327.66667	22193.33333	13866.33333	26444.33333
Drc1	21227	26806	149	40800
Drc7	18044	7231	27492.5	10723.5
Drd1	37039	8631	35422	311
Drd2	31692	33446.5	35359	34492.5
Drd3	3558	5133	8962	8002
Drd4	21833.5	21741.5	8820.5	9539
Drd5	18533.5	20402	19399.5	18294
Drg1	30508.5	33466	29783	34297
Drg2	2932	3617	39754	1389

Drosha	28023.33333	23859.66667	21363.66667	23458
Drp2	8080.5	24793	28712	19107.5
Drr1	18354	29374	22154	21982
Dsc1	28394	30251	36022	41133
Dsc2	23425.66667	26543	20221	27854.66667
Dsc3	28934	30601	33228	29701
Dscam	18550	33081.66667	15287.33333	21148
Dscaml1	12827.6	15479.6	17026.4	16821
Dscc1	26036	25399.5	7707.5	24259.5
Dscr3	15117	12884	13319	12786
Dse	17271	15600	16186	12561
Dsel	23904.33333	16783.66667	28691	30926
Dsg1a	27719	29140	30867	29384
Dsg1b	14961	18346.66667	13130	22431.66667
Dsg1c	716	38637	581	10031
Dsg2	28580.2	29667	34711.8	23525.8
Dsg3	8978	27339	18920.5	18252
Dsg4	1890	8624	19040	17065
Dsn1	9842	18548	10306	17725
Dsp	25250	29231.25	28091.25	31452
Dspp	29876	31376	33495	31878
Dst	16672.85714	20105	18537.28571	23908.28571
Dstn	18721.5	16988.5	9108.5	9342
Dstyk	10639.6	9685.8	27079	19894
Dtd2	32619.66667	29098.66667	32395.66667	28364.33333
Dtl	26248.33333	26804.66667	28752	27549
Dtna	27616.83333	28939.16667	30184	31105.16667
Dtnb	19377.66667	10998	33917.33333	13219.33333
Dtnbp1	23869	21077.5	24443.75	24584.75
Dtwd1	939	5804	3097	6297
Dtwd2	21977.33333	22604.66667	12023	24003.66667
Dtx1	8670	12521	2816	4063
Dtx2	19381	23357	17320	22646
Dtx3	18625	20480.66667	18381	19057.33333
Dtx4	15408	28166	16462.66667	17757.66667
Dtymk	14802.5	20370.5	18512	19035.5
Duox1	39289	40394	3685	3622
Duox2	3511	29584	40026	9493
Duoxa1	38879.5	37551.5	22781	5290.5

Duoxa2	21035	25121	31341	33655
Dupd1	9625	19675	15951	19059
Dus1l	36295.33333	23416	21140.66667	23213
Dus2	13674	16234	16040	18900
Dus3l	35738	31282	31514	29747
Dus4l	17539	19057	17967	20282
Dusp1	16402	19403	24687	27448
Dusp10	18526	8529	23027.5	21506
Dusp11	15596.75	16949.25	14520.25	16661.5
Dusp12	16488	33244	24454.33333	12647
Dusp13	18883	20775	17879	23458
Dusp14	16317.5	19665.5	28169	8801.5
Dusp15	26536.66667	26064.33333	21634.33333	22082.66667
Dusp16	27471	29710	29295	21439.66667
Dusp18	18819.66667	7090.333333	27472.33333	16845
Dusp19	22203.5	25799.5	28681	23051
Dusp2	33595	21518	24430	19460
Dusp21	28096.5	30199.5	30833.5	29546
Dusp22	4900.5	5585	6620	3489
Dusp23	18256	20654	20145	21403
Dusp26	39060	1584	28924	40699
Dusp27	19208.5	22499.5	18838	7567.5
Dusp28	8341	21015	25381	15662.66667
Dusp3	12976	2354	3401	39344
Dusp4	13248	29416	22756.5	21287
Dusp6	20587	22926	22542	23309
Dusp7	39947	19893	10594	2280
Dusp8	13194	15466.5	16843	20817
Dusp9	23759	20909.5	25884.5	22841.5
Dut	17636.5	21063	21089	18295.5
Duxbl	25935	27296	28739	27864
Dvl1	16212.5	19872	18042.5	19564.5
Dvl2	1370	37807	38979	32251
Dvl3	11175	12386	14782	14077
DXBay18	9542	7646	5624	7759
Dxo	7770	8968	5780	5863
Dydc1	40791	28107	31071	29605
Dydc2	25253	26498	28143	27147
Dym	21106.5	16450.5	21669	16912

Dync1h1	10980.66667	9487	6650.333333	8967.666667
Dync1i1	16444	24159	32528.5	3244
Dync1i2	31254	29848	30230.5	31267.5
Dync1li1	19424	19828.5	18070	20303
Dync1li2	25589	28228	29013.66667	18549
Dync2h1	20832.66667	15954.88889	22159.11111	28943.77778
Dync2li1	17275	23984	25748	22577
Dynll1	15869.5	16416.5	33065	13475.5
Dynll2	27198.33333	24325.33333	32743	28187.33333
Dynlrb1	7076	12725	10982	10810
Dynlrb2	17070.5	4853	5365.5	6727
Dynlt1b	17363.33333	12538	15889.33333	11447
Dynlt3	13178.5	18188.5	15429.5	15794.5
Dyrk1a	18875	18987.33333	23393.33333	21515
Dyrk1b	17219.66667	20508.66667	19900.33333	19140.33333
Dyrk3	3732	5253	7392	8055
Dyrk4	3359	4925	28394	3494
Dysf	24646	24022	23815	27291
Dyx1c1	39619	37545	40642	39827
Dzank1	32346.33333	14599.66667	32669.66667	32015.33333
Dzip1	23003.33333	20386.66667	34451.66667	25125
Dzip1l	29631.33333	17514.66667	21920.33333	22154.33333
Dzip3	17101.66667	16014	13813.33333	12450
E030002G19Rik	8410	9600	13738	18803
E030002O03Rik	27939	29751	1107	28112
E030003E18Rik	27378	29687	4456	7436
E030003N13Rik	26219	27501	29658	28301
E030004H24Rik	40147	28697	31253	36539
E030011O05Rik	31220.5	30625	16455	20799.5
E030026E10Rik	521	25	2108	7522
E030030F06Rik	3591	37005	32986	31499
E030030I06Rik	19709	19250	24481	23120.5
E030042N06Rik	34464	17140	18803.5	18529
E030046B03Rik	3588	35743	33686	34665
E130006D01Rik	4436	7172	16074	13468
E130006N16Rik	32586	34530	31555	28639
E130008O17Rik	1448.5	28422.5	31547.5	31251
E130009g09rik	5226	30475	31823	30300
E130012A19Rik	2902	5810	4788	8212

E130101M22	24857	25958	28196	27011
E130102H24Rik	19874	17554	22753	21879
E130104P22Rik	32963	4235	36561	4949
E130106K03Rik	22900.5	8997.5	11715.5	10735
E130108L08Rik	28654	30057	32043	30178
E130112N10Rik	17342	14514	19749.5	18841.5
E130114P18Rik	33631.5	20183	24091	28448
E130118H10Rik	22216	15473	24297	20427
E130119H09Rik	6778.5	10356	16684	15463.5
E130120C16Rik	23481	35224	26545	25408
E130201H02Rik	5939	9913	9068	7930
E130202H07Rik	1736	13436	32172	2852
E130304I02Rik	28027.5	27741	12168.5	11847.5
E130307A14Rik	7142	25328	24712	30607
E130308A19Rik	28486.5	24545.5	21078.5	20114.5
E130309D02Rik	22551	25458.5	3918	5443.5
E130311K13Rik	5393	7804	6591	4422
E130314M14Rik	12124	11727	11700	12378
E230001N04Rik	27567	27857	36024	40904
E230008J23Rik	26959	22330	26992	25912
E230008O15Rik	9855	2879	10305	9893
E230012J19Rik	40429	7862	12510	11434
E230015B07Rik	26089.5	15324.5	30411.5	15289
E230016K23Rik	23897	24490	26634.5	22492.5
E230016M11Rik	8779	5199.5	13088.5	12852.5
E230024E03Rik	15934	14090	15522	14107
E230025N22Rik	4109	8901	12877	14160
E230029C05Rik	35139.5	25668	34477.5	26772
E2f1	8757	11223.5	27632.5	9169.5
E2f2	38674	27558	21832	12127
E2f3	19788.5	20919	22110.5	22471.5
E2f4	3204	3839	40526	1740
E2f5	22966.66667	24883.33333	19986.33333	23037.33333
E2f6	21754.5	24915.5	19573	22945.5
E2f7	29391.33333	27871	18279.66667	19464
E2f8	12367	18540	6380	14366
E330009E22Rik	9504	18863	25488.5	9425.5
E330009J07Rik	21148.5	21853	20510	21929
E330013P04Rik	27591	32717.5	17296.5	16781

E330013P08Rik	27631	17004	25991	21824
E330017A01Rik	1901	4938	14875	15549
E330018D03Rik	33636	19576	18243	17217
E330018M18Rik	29744	31705	31580	30194
E330019L11Rik	39704	937	34671	1632
E330021A06Rik	22583	15792	10564	18729.5
E330021D16Rik	23028	23745	34702	34555
E330024J20Rik	39504	31537	38401	32097
E330032C10Rik	23688	40497	27084	26063
E330034G19Rik	16876	17243	15413	21599.5
E330037G11Rik	15570	12495	12278	16226
E330037M01Rik	31316.5	15657	30892.5	39210.5
E430013K19Rik	33136	10482	33324	31678
E430014L09Rik	3689	18243.5	12229	19969.5
E430018J23Rik	17616.5	19034.5	18959	19313.5
E430021H15Rik	27524	28940	30310	38548
E430022E14Rik	7018	3538	8176	6632
E430024P14Rik	19131.5	13400	19309.5	15661.5
E430025E21Rik	1248.5	20834	18762.5	21416
E4f1	19522	17372	20447	18577
Eaf1	22189.5	19872.5	18815.5	24320.5
Eaf2	20890.5	28177.5	25333	27800
Eapp	38577	3946	4425	2560
Ear1	26943	13366	24326	39028
Ear10	33608	11620	18789	28679
Ear14	30457	32049	34154	32446
Ear2	36369	24708	35215	25293
Ear6	27537.5	17222.5	12369	21430.5
Ears2	29452.5	31588	30446.5	13141.5
Ebag9	19150	25385	24521.5	23591
Ebf1	25008.83333	25670.66667	30675.33333	28795.66667
Ebf2	21730	26906.5	33759.5	33123.5
Ebf3	23326	11534	21110.5	22433
Ebf4	21708.66667	25779.66667	28087	30226.66667
Ebi3	40169	39094	26870	32480
Ebna1bp2	8679.5	10271.5	8370.5	9734.5
Ebp	20677	18791	20193	18269
Ebpl	8779	11859	9573	9064
Ecd	28916.5	27749.5	24441.5	25393.5

Ece1	7994	20566.5	11760	28731
Ece2	23852.66667	13096	27051	29113.66667
Ecel1	14444	17876	18675	19317
Ech1	37765	33806	97	36374
Echdc1	1171	7774	6567	7893
Echdc2	39956	623	718	6815
Echdc3	14768	6261	13272	1465
Echs1	14691	15014	13015	13356
Eci1	25385	37752	26700	34522
Eci2	8301	13550	6254	9680
Eci3	4818	11451	3361	8724
Ecm1	33137.5	28609.5	19067.5	18347.5
Ecm2	33811	32448	33765	32801
Ecscr	21446	37951	35936	8787
Ecsit	14233	14409	13315.5	13579
Ect2	13580	13112	17606.5	16581
Eda	31101	20688	22688.33333	22990.33333
Eda2r	31618.33333	23178	36613.66667	23172.66667
Edar	8675	9364	22779	20123
Edaradd	17563.33333	12629.66667	9459.333333	19077
Edc3	35929	33965.5	12968	30131.5
Edc4	39577	34693	31916	34690
Eddm3b	31606	33383	34975	33489
Edem1	7284.333333	17822.66667	19124.66667	4664
Edem2	18794	17176.5	17629	13397.5
Edem3	1694	39793	1390	40983
Edf1	21148	22927	25088	34059
Edil3	27661.14286	30778.42857	23810	26034.71429
Edn1	36922	37215	30827	29624
Edn2	5822	3433	5734	8384
Edn3	21065	24960	9965	27719.5
Ednra	24709.4	22536	30814	25834.2
Ednrb	35296.5	13917.5	12935	19066
Edrf1	14039	12706	12574.5	15727
Eea1	18522.16667	23892.66667	19187	24322.83333
Eed	19067	25222	24463	26603
Eef1a1	19130.5	15686.5	4728	6106
Eef1a2	5192	32474	40630	37923
Eef1b2	34603	28482	30803	28665

Eef1d	21763.66667	18583	24269.33333	24598
Eef1e1	29146	40278	36979	39625
Eef1g	19101.5	21280	18862.5	22145
Eef2	19310.83333	16749.5	12504.66667	12574
Eef2k	18963.33333	24111	30426.33333	15408.33333
Eef2kmt	798	3586	40669	1060
Eefsec	36771	39741	37037	40712
Eepd1	22795.33333	31132.33333	19276	20616
Efcab1	13920.75	16432.75	26850	17031.75
Efcab10	27854	29292	30767	29725
Efcab11	22497	20361.5	23159.5	18552
Efcab12	14551	4051	5391	7621
Efcab14	19805	27538	14554.8	18295
Efcab2	13977.5	18081	16516.5	18451
Efcab3	23629	19474	23175.5	20391
Efcab5	17322	22747	26872	27824
Efcab6	32117.5	35164	15874.5	15767.5
Efcab7	3876	37965	13244	16177
Efcab8	35553	447	9933	8517
Efcab9	4008	6469	38858	4994
Efcc1	25048	23917	36180	34948
Efemp1	15509	17101	9354	7661
Efemp2	14529	13488	15748	16498
Efhb	4850	27743	7850	40808
Efhc1	37253	28406	38077	37180
Efhc2	22555.66667	23661.33333	27554.66667	26181.33333
Efhd1	27277	30885	30224.5	34741
Efhd2	37689	29060	35139	29825
Efna1	16259	18155	24333	20778
Efna2	28834	7207	33484	1430
Efna3	3439	33169	36730	34695
Efna4	26742	31379	1106	3362
Efna5	21418	23138	9414.5	8796
Efnb1	33898	30133	37839	37339
Efnb2	19668	19405	25733.5	8699.5
Efnb3	14108	18718	27483	29236
Efr3a	19856.75	21419.25	17920.5	19564.375
Efr3b	26047	5520	22745	5148
Efs	19119.5	18355.5	4946.5	23345.5

Eftud1	28880	32224.5	31521.5	30590
Eftud2	26582	22340	20286	19676
EG193330	30392	609	37952	1364
EG195281	1477	35001	37389	21825
EG383189	16285	14262	18533	18281
EG433614	721	5471	41112	281
EG436227	12856	18069	20212	22418
EG547260	17287	11994	15871	14347
Egf	37377	37048	39726	37750
Egfem1	26693	27180.25	35887	24199.25
Egfl6	30132	31761	32584	31190
Egfl7	30884	37327	2550	10228
Egfl8	38100	39440	5610	4058
Egflam	18268	5393	10735	9761
Egfr	22299.42857	19515.14286	16184.85714	18427.85714
Egln1	14766.5	13621	13948	14259
Egln2	29349	23693	22537	22341
Egln3	19070	19633	25774	30488
Egr1	586	37649	35553	310
Egr2	23475	19315	26587	29301
Egr3	2781.5	37875.5	16606.5	6635.5
Egr4	32570	36684	935	38582
Ehbp1	21239.5	18047.25	19967	18883.25
Ehbp1l1	16117	11383	16640	13782
Ehd1	988	28843	3921	27843
Ehd2	21795.66667	5993	7631	9624
Ehd3	22080	21551	3119	40824
Ehd4	31188	14319	28214	17645
Ehf	19016.66667	18393.33333	23760.66667	10683.66667
Ehhadh	17391	17790	11842	12163
Ehmt1	22813	25851	25276.5	24328.5
Ehmt2	18289	16975	20822	20782
Ei24	27436	24364	28125	26669
Eid1	10598	5407	12774	697
Eid2	19905	23210	26194	25780
Eid3	40104	7386	37846	15413
Eif1	23600.5	15244.5	20764.5	17432
Eif1a	12832.5	18353.5	14805	16181
Eif1ad	9747.5	14034	14279	16706

Eif1ax	6525.5	10676	7359	9602
Eif1b	7828	7551	8210	4731
Eif2a	12498	27097.5	28901	29897.5
Eif2ak1	20447.75	20542.25	18289.5	20394.75
Eif2ak2	39180	4190	896	8723
Eif2ak3	20242.5	14070	15610	14182
Eif2ak4	9872	11437	11095	10745.33333
Eif2b1	28822	29864	22373	23109
Eif2b2	9116	7567	10388	12563
Eif2b3	9752	6397	15693	16223
Eif2b4	28797	24460	23013.5	28069.5
Eif2b5	19435	21074	19934	21217
Eif2d	14819.25	17424.5	11802.25	14943.25
Eif2s1	10245	15121	14666.66667	16336
Eif2s2	18192.66667	17891	16143.33333	18631.33333
Eif2s3x	23443.25	26942.25	25070.25	26484.75
Eif2s3y	28890	31224	21957	23125
Eif3a	9747.333333	8318	22523	13573.66667
Eif3b	23494.5	22813	21633.5	22876
Eif3c	23404.75	22360	17889	17033
Eif3d	14972	15345	16674	17950
Eif3e	9092.5	30430.5	25876	27526.5
Eif3f	15943	7038	4984	4675
Eif3g	33323	35558	38044	37740
Eif3h	35274	25132	25288	26866
Eif3i	28000	40079	2317	4829
Eif3j1	34258.66667	21752	19555.66667	20522
Eif3k	36642	31478	32311	32712
Eif3l	34778	35629	35516	35827
Eif3m	20635	21058.5	20929	21196
Eif4a1	17079.66667	15926	22119.66667	20898
Eif4a2	15214.5	15498	16765	16513.5
Eif4a3	35575	32772	34561	37053
Eif4b	30952.25	32248.5	30728	31018.75
Eif4e	26669.25	17243.75	14234	20392
Eif4e2	31846.5	22692	15189.75	18854.25
Eif4e3	8916	9949.5	24158	22367
Eif4ebp1	5894.5	13075.5	11956	13699
Eif4ebp2	27045.5	18040.5	16000	10866.5

Eif4ebp3	21246.5	15642	17880	16956
Eif4enif1	17927.8	14159.4	22567.2	22715.2
Eif4g1	4646	3055.5	20851	20705
Eif4g2	21144.75	11132.5	8801.5	12738.5
Eif4g3	5227	2210	6138	22395.5
Eif4h	16786.5	15480.5	14141	17146
Eif5	19938.66667	27202.33333	37736.66667	36070
Eif5a	28370	26510	20405.5	21665
Eif5a2	26261.5	25520	27118	27585.5
Eif5b	21785.8	18826.6	21649	20079.4
Eif6	11291	22418.33333	15713	27942.66667
Elac1	34865	33322	33386	36329
Elac2	19244.5	18279.5	38231	38464.5
Elavl1	9758.666667	11755	13010.66667	14077.66667
Elavl2	24170	11913.66667	27116.66667	15462.33333
Elavl3	16382	35293.5	38215.5	34982.5
Elavl4	4180.5	22034.5	26249	25932
Elf1	18268	23949	15876.66667	25592.66667
Elf2	31227	23119	19563	13340
Elf3	23766	24930	26799	34487
Elf4	18236.25	14588.75	15933	17054.25
Elf5	40704	6784	40065	12837
Elfn1	21929	22082	25817	32447
Elfn2	31010	35376	1340	2218
Elk1	25991	25358	27656	26448
Elk3	26478.5	11920.5	22849	11514.5
Elk4	16276.75	10616	18969.25	13582
Ell	16977.5	30202.5	10469	28703
Ell2	9307.333333	27694.66667	10550	18793
Ell3	29796	39215	31553	30195
Elmo1	23978.42857	18162.42857	18173.28571	15635
Elmo2	24169	35877	18552	30138
Elmo3	7863	11827	7936	13623
Elmod1	28874	13910.5	33549	17110
Elmod2	13372	11292	10078	9259
Elmod3	40354	37640	37657	35942
Elmsan1	26727	21593	15525	13206
Eln	17046	18640	21320.5	20620.5
Elof1	16334	18123	16964	16558

Elovl1	24583	19602	17328	19860
Elovl2	5928	11939	6445	21342
Elovl3	6208	1236	11485	10153
Elovl4	37862	29877	31929	37828
Elovl5	18714.5	14254	17725	12609
Elovl6	24071	23437	19439	16866
Elovl7	20832.5	31117	34080	30448
Elp2	12446	11076	6415	7417
Elp3	8450	7747	4609	6014
Elp4	23220.66667	27364.33333	19664.5	19916.16667
Elp5	33455	38727	34594	37985
Elp6	12075.5	12491	13222	13237
Emb	26125.66667	23218.33333	10042.66667	19815
Emc1	12765	12380.33333	14079.33333	13366.66667
Emc10	3100	40432	39964	40329
Emc2	23937	25716	23760	25714
Emc3	31817.5	32698	32302	32423.5
Emc4	13407	17706	18204	21038
Emc6	37240	1019	38003	39406
Emc7	26346.5	23390.5	29146	29800.5
Emc8	21222	22498	21274	21118
Emc9	38337	38290	40948	37527
Emcn	35041	37027	36699	3105
Emd	39596	37599	87	38105
Eme1	23728	23625	23313	24875
Emg1	36505	1400	3885	2347
Emid1	40824	7102	7968	13754
Emilin1	41061	3540	5354	265
Emilin2	16096	28487	32221	22724.5
Emilin3	24539	25121.5	31696.5	30474.5
Eml1	20012.5	21363	22287	22808
Eml2	1765	517	1093	157
Eml3	36696	34489	38146	37623
Eml4	11429	20098.66667	34027.33333	20491
Eml5	25609.6	23134.8	23511.4	25651.4
Eml6	24846	17668	16911.66667	19893.66667
Emp1	16405	39677	17915	3287
Emp2	18242	14825	16490	19066
Emp3	34037	39935	155	1665

Emr4	3337	18270	40410	8269
Emx1	12067	14749	10481	13232
Emx2	22151.5	32645.5	29467.5	16087
En1	28257	31619	34132	35049
En2	3712	8051	8177	11763
Enah	23155.83333	24591.83333	27835.66667	31090.83333
Enam	24746	26045	27427	26295
Enc1	11673	12243	18411	10327
Endod1	38597	15623	29774	10909
Endog	1062	7310	2118	4430
Endou	4291	29332	3508	30701
Endov	22728.25	21957.5	22303.25	22737.75
Eng	5673	6076	7110	4019
Engase	1086.5	26337	38200.5	20611
Enho	35303	34644	1854	40916
Enkd1	10706.5	10179.5	29880.5	10460
Enkur	36446	35764	33738	32001
Eno1b	19249	12230	5826.5	13403
Eno2	14575	12414	15360	14091.5
Eno3	8149	14680	15359	18009
Eno4	20327	19851	28794	27933
Enoph1	19245	22643.5	18510.5	22765.5
Enox1	40801	40832	18401	15682
Enox2	24638.5	24431	28008	24894
Enpep	29884	33730.5	19397.5	15710.5
Enpp1	20552.5	23783	34504	17168
Enpp2	7683.5	27543	23007.5	12934.5
Enpp3	24504	25616.33333	15369.33333	18366
Enpp4	10979	24181	28034	39329.5
Enpp5	16390	22364	22512	26171
Enpp6	13471.5	31369.5	29333	33686
Enpp7	7792	4683	12785	16373
Ensa	32056	20902	23277	24046.33333
ENSMUSG00000069586	15896	14916	12218	14221
ENSMUST00000014223	21995	24820	29687	32507
ENSMUST00000016770	24862	30136	27875	28974
ENSMUST00000020357	23490	24640	26217	25161
ENSMUST00000021175	3829	5247	10935	8498
ENSMUST00000021240	10889	14315	12255	13949

ENSMUST00000021489	21896	38148	28481	38581
ENSMUST00000021743	8228	14305	12944	11359
ENSMUST00000023249	35430	36147	4047	32853
ENSMUST00000024003	11222	14717	17169	18181
ENSMUST00000024718	5179	8719	13183	20188
ENSMUST00000024744	33722	36344	95	753
ENSMUST00000024982	5639	4945	12814	12799
ENSMUST00000025191	15427	17001	22054	23076
ENSMUST00000026683	24821	35404	37830	37726
ENSMUST00000030569	40277	38436	37858	1599
ENSMUST00000034640	37855	38506	2721	352
ENSMUST00000036036	19462	21079	25489	24783
ENSMUST00000037357	34219	36106	37016	35263
ENSMUST00000038620	38706	34978	32722	1388
ENSMUST00000038873	8938	4703	10043	10066
ENSMUST00000039549	6158	33341	4446	641
ENSMUST00000039920	36756	2808	2050	5393
ENSMUST00000040006	35712	41054	37296	1780
ENSMUST00000040643	39572	28733	36977	24900
ENSMUST00000040998	384	5657	8019	15093
ENSMUST00000042867	38591	37349	1934	3630
ENSMUST00000043170	19150	21536	24633	24110
ENSMUST00000043333	16815	16984	19403	16012
ENSMUST00000043542	8458	9202	12456	12374
ENSMUST00000044915	25118	30900	33587	34631
ENSMUST00000044989	24554	25912	26946	25943
ENSMUST00000046911	14751	24274	27941	34345
ENSMUST00000047386	37730	30943	2024	1754
ENSMUST00000047643	36858	38839	37656	39686
ENSMUST00000047876	8741	18501	19306	10174
ENSMUST00000048800	37154	34445	27075	34679
ENSMUST00000048958	12191	14042	19567	17974
ENSMUST00000049869	12880	15290	17687	17777
ENSMUST00000050430	19455	22756	19008	20302
ENSMUST00000050537	11854	12275	10931	14208
ENSMUST00000050712	15752	17756	12044	14034
ENSMUST00000052243	21574	25198	25235	21642
ENSMUST00000052719	33368	15814	41108	12
ENSMUST00000052880	31237	34642	40649	3276

ENSMUST00000053291	36174	1495	7074	6162
ENSMUST00000053608	11418	8919	6269	7496
ENSMUST00000054243	21982	18868	22135	23224
ENSMUST00000054478	5557	10878	10130	10596
ENSMUST00000054828	22715	28630	29235	31826
ENSMUST00000055154	14702	11510	3459	14176
ENSMUST00000056681	1948	4901	5901	6107
ENSMUST00000056717	12089	9992	5958	12141
ENSMUST00000058512	25724	25792	27522	26813
ENSMUST00000059921	14406	16800	15324	16972
ENSMUST00000061184	30503	32071	1873	32026
ENSMUST00000061548	35286	23924	23457	25640
ENSMUST00000062016	18051	22280	16356	21965
ENSMUST00000062198	15058	17456	27007	26699
ENSMUST00000062754	39632	21253.5	17463.5	25083
ENSMUST00000063087	25044	39638	22094	37432
ENSMUST00000063943	39881	13850	5229	11041
ENSMUST00000064589	12827	16190	16716	18591
ENSMUST00000064708	29449	30913	8864	4600
ENSMUST00000065449	16739	12340	14884	10484
ENSMUST00000065540	39634	38777	7051	4091
ENSMUST00000067723	9670	19802	15776	11545
ENSMUST00000070540	12435	11127	20678	20261
ENSMUST00000071461	12965	10718	20450	22422
ENSMUST00000071587	13886	18395	19648	20564
ENSMUST00000071702	19521	26289	23720	29158
ENSMUST00000071734	4171	11836	27501	1122
ENSMUST00000071757	31518	26309	423	36331
ENSMUST00000071777	36724	38251	3134	2313
ENSMUST00000072006	11584	15193	16437	15602
ENSMUST00000072166	20901	26018	25479	23032
ENSMUST00000072181	12657	17752	15311	14562
ENSMUST00000072368	12966	15337	20376	15722
ENSMUST00000072786	18832	17804	27467	31918
ENSMUST00000072864	11688	12875	15300	14787
ENSMUST00000072871	27728	35366	304	35904
ENSMUST00000073231	7826	11797	15161	4933
ENSMUST00000073319	7327	9527	13106	14376
ENSMUST00000073476	33711	35520	40800	38450

ENSMUST00000073513	675	37450	25904	118
ENSMUST00000073543	2558	3531	8775	7385
ENSMUST00000074060	39128	10512	4538	13133
ENSMUST00000074244	35925	8352	13835	7897
ENSMUST00000074313	8879	15500	19763	22689
ENSMUST00000074715	6792	8038	13033	11987
ENSMUST00000074809	34934	34263	24856	32682
ENSMUST00000075701	41025	10254	17210	8785
ENSMUST00000075966	27112	28559	30409	36355
ENSMUST00000075990	16689	17630	22309	21992
ENSMUST00000076215	33448	26138	29483	31738
ENSMUST00000076359	10779	16393	19757	20359
ENSMUST00000076447	14112	13895.5	13016.5	15514.5
ENSMUST00000076520	22163	25278	32873	35187
ENSMUST00000077110	22967	22922	25990	28892
ENSMUST00000077152	5743	10758	12164	14516
ENSMUST00000077170	28192	27925	1757	40949
ENSMUST00000077238	40568	1237	5652	3439
ENSMUST00000077326	11243	16909	25019	22768
ENSMUST00000077721	28084	24094	22107	24466
ENSMUST00000078007	37771	18601	14371	7398
ENSMUST00000078284	2544	40329	8289	10008
ENSMUST00000078295	2258	6914	9798	9008
ENSMUST00000078613	11663	9748	7228	12112
ENSMUST00000078883	38904	35967	33327	39133
ENSMUST00000079044	10300	18628	14401	14694
ENSMUST00000079520	21066	22841	13170	10146
ENSMUST00000079834	35312	23900	39383	40132
ENSMUST00000080182	5766	6529	12727	10257
ENSMUST00000080591	3367	38520	39912	394
ENSMUST00000080662	36913	35991	25097	35071
ENSMUST00000080763	17758	19838	11382	17957
ENSMUST00000080922	7099	5976	1814	7516
ENSMUST00000081530	12117	22160	27503	28497
ENSMUST00000081800	20902	21268	23188	22281
ENSMUST00000081873	5110	12383	15755	13401
ENSMUST00000082107	15930	20495	23263	22674
ENSMUST00000082346	36232	33174	142	613
ENSMUST00000084077	5795	8119	2061	12610

ENSMUST00000084458	31934	34804	39009	38996
ENSMUST00000084861	6259	16051	15636	16612
ENSMUST00000085075	33646	39444	1807	3454
ENSMUST00000085482	475	31459	1943	39988
ENSMUST00000085597	23483	24639	22679	21135
ENSMUST00000085613	5478	40217	39219	34902
ENSMUST00000085829	8828	11007	11414	11177
ENSMUST00000085831	38961	1761	39465	3830
ENSMUST00000086086	33099	35070	35870	33952
ENSMUST00000086288	35951	6321	28290	29742
ENSMUST00000086680	25375	21462	22479	22587
ENSMUST00000087819	36766	28586	2205	351
ENSMUST00000088501	5479	6855	11055	9183
ENSMUST00000088681	12568	15074	14595	13607
ENSMUST00000088886	13398	19407	14478	18794
ENSMUST00000088909	34885	39267	40236	1446
ENSMUST00000089099	5982	8433	14618	15011
ENSMUST00000089219	11883	18207	18556	17909
ENSMUST00000089431	32754	37401	7772	667
ENSMUST00000089472	35149	38000	37323	32057
ENSMUST00000089791	37594	7034	5366	36242
ENSMUST00000090542	18856	22821	20903	20715
ENSMUST00000091052	39387	1661	6194	2753
ENSMUST00000091141	34870	34192	35066	38814
ENSMUST00000091462	7528	9329	7084	9611
ENSMUST00000091555	21829	22836	23475	28180
ENSMUST00000091620	21299	23051	21454	21333
ENSMUST00000092045	35157	7037	4516	11036
ENSMUST00000092120	25829	27096	28671	29924
ENSMUST00000092184	20775	26159	25027	32888
ENSMUST00000092503	36424	34358	24213	39367
ENSMUST00000092871	17027	19168	18240	19178
ENSMUST00000093543	6551	8235	6602	4412
Enthd1	35939	39058	9657	7378
Enthd2	3368	39214	36797	34761
Entpd1	25377	23246.5	18257.5	22977
Entpd2	22578	37856	25372	3224
Entpd3	29088	37149	2220	948
Entpd4	26994	27888.5	21440	24442.5

Entpd5	34446	15320.5	36705.5	35711.5
Entpd6	29458.66667	28612	24513.66667	23083.33333
Entpd7	23264	19105	18020.5	20530
ENTPD7	16428	14491	21852	23730
Entpd8	15975	10382	29511	25240
Eny2	19277	18385	13860.5	14154
Eogt	22898	23955	20945	24217
Eomes	33641	31475	8929	30666
Ep300	15736.25	16744	20243.5	18701.75
Ep400	11175.5	17461.25	27960.5	18038.25
Epas1	27031.5	16264.5	9199.5	17550
Epb41	21039.4	20639	18643	19947.2
Epb41l1	21546.66667	26501.66667	20559	27125.33333
Epb41l2	16634	19687.83333	18011.33333	21437.16667
Epb41l3	28707	32961.5	26682	33674
Epb41l4a	23773.33333	23962	21257.33333	19472
Epb41l4b	30776	32732.5	35941	33576
Epb41l5	10502.5	15045.5	17634.5	17842.5
Epb42	32577.5	32934	16177.5	16709.5
Epc1	21444.16667	25800.83333	24585.5	25823.33333
Epc2	16835	15758	17718.5	17103
Epcam	897	6233	3622	1233
Epdr1	31947.66667	21947.66667	22336.33333	15128.33333
Epg5	9498	7186	8265	8060
Epgn	31793	25304	34507	37096
Epha1	22534	22630	35996	27714
Epha10	38331	31911	2612	32339
Epha2	35960	35194	131	3308
Epha3	30174	30328.6	34426.8	27522.6
Epha4	21666	27996	25165	13742
Epha5	34941.75	27728.75	17731.25	11503
Epha6	20038.66667	30760.33333	21648.66667	26787.33333
Epha7	19623	27122	26115	33777.5
Epha8	12172	17952	23441	33672
Ephb1	7284	644	4275	12354
Ephb2	23338	16174.5	34483.5	13913
Ephb3	36880	40102	6787	7818
Ephb4	19385.5	16933.5	37886	22529
Ephb6	1887	35175	602	36900

Ephx1	16728.5	20744.5	16800.5	23648.5
Ephx2	14641	21969	20900	29617
Ephx3	26770	23429	2528	39254
Ephx4	30173	31556	1287	31145
Epm2a	6360	8699	13417	14571
Epm2aip1	21556.5	40635.5	39802	37735
Epn1	33396	27702.5	12629.5	31719
Epn2	18304.5	19101.5	21039.5	21186.5
Epn3	8706	11165	14281	14696
Epo	15161.5	1159	8095.5	6691
Epor	5650	5439	12970	9456
Eppin	20723	23327	36885	36419
Eppk1	27349	36258	37675	983
Eprs	20042.5	21478.5	27369	31206.75
Eps15	20757.33333	16936	21519	21757.66667
Eps15l1	19896.5	17481	18138.5	17921
Eps8	16891	12623	19191	16196.5
Eps8l1	4092	39225	39471	35692
Eps8l2	19916	16945	21232	19672
Eps8l3	17820	20816	31952	36048
Epsti1	27440.66667	23574	31927.66667	28171.33333
Ept1	18157.5	16945	15682.75	17527
Epx	4332	9301	4738	11376
Epyc	34829	36162	19964	18586
Eqtn	33988	4075	5728	9838
Eral1	26893.5	4468.5	4620.5	21631.5
Erap1	26234.5	30349.5	20890	27521
Eras	34319	10900	3403	6927
Erbb2	23785	23811.5	11147	10318.5
Erbb2ip	29970	25638.4	21770.4	28779.6
Erbb3	15081	15969.4	21085.2	17101.4
Erbb4	22850.1	26557.1	27338.1	23127.7
Erc1	25000.375	21599.5	33762.125	32802.375
Erc2	20437.16667	23985.5	19693.16667	22833.16667
Ercc1	19205	19953	19895	20238
Ercc2	879	37792	35584	35890
Ercc3	25758	23171	23018	22906
Ercc4	25762	16234.5	12867.5	14723.5
Ercc5	14551.5	14233	10848	13237

Ercc6	13789.5	20317	9791.5	9191.5
Ercc6l	19743	23090.25	18945.75	34616
Ercc6l2	29081.2	25875	21304.4	20198.2
Ercc8	10099.66667	10640	21852	9646.666667
Erdr1	17163.5	14618.5	17536	18639
Ereg	27934	27582.5	40106.5	35915.5
Erf	40369	35136	3185	39782
Erg	25078.5	26431	17661	21049.5
Ergic1	26201.66667	25230	27569.33333	26188.33333
Ergic2	23726	25515.25	22234.25	24613
Ergic3	5717	8037	6630	8499
Erh	18704	25289	29156.5	28690.5
Eri1	30392	35121	31002.66667	34950.66667
Eri2	17684.66667	21868.33333	18820	21970.33333
Eri3	29327.5	29352.5	12086	14967.5
Erich2	27688.66667	29046	32346.66667	31962.66667
Erich3	21721.5	23697.5	29417	32153
Erich5	10926	11513	18962	18012
Erich6	38820	1936	370	3158
Erlec1	2354	37995	39117	39293
Erlin1	38134	37734	37938	36797
Erlin2	33881	19985	33749	34697
Ermap	4799	35521	10367	14385
Ermard	17111	22113.16667	19438.5	12381
Ermn	25470	26861	38466	26957
Ermp1	16926.25	23308.25	24110	22356.75
Ern1	16848	14735	17334	18041
Ern2	20982	24056	33197	34710
Ero1l	21755.5	25918	20320	28112.5
Ero1lb	10437.75	26848	10837.5	29127
Erp27	13002.5	32741	36033	33062.5
Erp29	8189	3124	7448	1479
Erp44	11430.25	13165.75	15525.75	17494
Errfi1	6057	4799	6999	5118
Erv3	13325	12051	15402	17155
Esam	25686	35294	29018	2648
Esco1	16285	12730.33333	9299	10439.66667
Esco2	10529	16496	39966	27643
Esd	27566.5	12806.25	15024	15855.5

Esf1	27374	26441.66667	23565.33333	24417.66667
Esm1	32859	34300	36131	37905
Espl1	8595.5	28496	25822	12721.5
Espn	24036.5	14032.5	25406.5	18157
Espnl	32995	34864	36447	34307
Esr1	11310.5	11462	5161.5	9557
Esr2	28933	31179	38653	36791
Esrp1	30445	31146.5	31887.5	18651.5
Esrp2	8105	25445.5	12949.5	9248.5
Esrra	35958.5	28645	28040.5	30835.5
Esrrb	27117	29195	33095.5	19445
Esrrg	24406.5	28271.5	26632	24684
Esx1	27183	28427	31469	29965
Esyt1	19201	14251	17637	11202
Esyt2	26650.66667	13233.66667	15823	17844.66667
Esyt3	15275.5	17958	22423.5	2969
Etaa1	22625	20472	34786.5	20052
Etd	21948	18762	21360	24597
Etf1	33883.5	25582.5	22393.5	26658
Etfa	2415	3755	1671	1074
Etfb	16788	16644	20140	17360
Etfdh	28394.75	29657.75	22775.5	24277.75
ETG02_36680	35128	36287	35789	40148
ETG04_27747	34547	35174	513	36467
ETG05_36762	2687	2336	6253	6870
ETG05_66023	20182	20754	24119	24629
ETG07_105829	29814	32099	32222	36190
ETG08_142674	30028	30259	32674	33320
ETG09_205211	28070	30149	35359	30434
ETG09_35454	28945	34735	37356	39923
ETG09_48764	35856	32892	35486	33865
ETG10_13482	29028	32003	38046	34241
ETG10_195139	922	40872	5953	8015
ETG10_234183	145	2559	40852	6000
ETG10_236652	31116	32525	36023	34640
Ethe1	21436.5	20437.5	17754.5	15111
Etl4	23290.71429	18168.85714	24629	23988.28571
Etnk1	24211	19568.66667	14199	17292
Etnk2	21651	29010	24500	35061

Etnppl	21496	21766	26104.5	28170
Etohd2	26345	24772	26564	9880
Etohi1	9962	8755	232	5372
Etos1	30081	31974	32012	34187
Ets1	23112.75	30288.75	23356	16803.25
Ets2	10669	21448	9785	22378
Etv1	29854.5	16646.5	21481	17409
Etv2	41073	2486	6231	35932
Etv3	24081.5	6785.5	6316.5	6880.5
Etv4	33656	35431	7027	1752
Etv5	21183	32378	2672	37830
Etv6	27024	28067	28358	32153
Eva1a	16631	19104	37624	35502
Eva1b	5364	17316	12070	19816
Eva1c	17569.66667	20185.66667	21192.66667	26032
Evc	8719	8513.333333	7992.666667	12980.66667
Evc2	30351	31308	15474	17970.5
Evi2a	19125	7888	15976	6971
Evi2b	9725	20035	16317.33333	14154.33333
Evi5	21436.33333	16138	18632	20976.66667
Evi5l	15007.5	22757.5	28717.25	28572.25
Evl	33481	18216	25184	21262
Evpl	12325.5	14607	16532	15979
Evx1	4431	11301	12681	17804
Evx2	19583	9536	11953	17184
Ewsr1	6071.5	24960	9205.5	9798.5
Exd1	34667	25338	29542	24015
Exd2	17325.5	15332.5	31323.5	25428
Exo1	7024	2152	6045	1732
Exo5	31816	36468	35624	33173
Exoc1	16151.66667	33714.66667	22299.66667	13966.66667
Exoc2	18027.5	9521.25	18710	11399
Exoc3	15787.33333	13694.66667	24293.33333	12938
Exoc3l	377	6186	18851	8256
Exoc3l2	20608	39005	21720	15773
Exoc3l4	23933	3532	39635	6747
Exoc4	28332.33333	21285.66667	23741.77778	22106.55556
Exoc5	27859	27947.33333	16414.33333	17341.66667
Exoc6	19124.33333	20154.33333	18900	18583

Exoc6b	10809.66667	9273.666667	22799	11363.66667
Exoc7	5902	4757	6352	5266
Exoc8	33837	29155	27704	26503
Exog	13939	8201	8213	6029
Exosc1	20722.2	24351.8	23433.6	24915
Exosc10	11317	12793	12732	13786
Exosc2	18648	20017	19366	20794
Exosc3	22982	26951.5	25033.5	25283.5
Exosc4	13903.5	15997	15009.5	16927
Exosc5	2394.5	3061	3192.5	5643.5
Exosc6	22452.5	1184	40892.5	25503.5
Exosc7	20850.5	23724	21590	21297
Exosc8	36096	19197.5	39521	40081
Exosc9	24024.5	21023	20935	25764
Exph5	21915.5	23868	25566	24402
Expi	31794.5	27022	38127	34764.5
Ext1	7449.75	22197.25	16209.5	18721.75
Ext2	40223	36444	39283	35635
Extl1	25256	24424	30434.5	34538.5
Extl2	9030	7707	8776	8237
Extl3	32550	29850.5	30226.5	31173.5
Eya1	22505	21581.5	20004	19931.5
Eya2	5036	1258	5385	6762
Eya3	10985.75	17881.75	10807.75	9985.5
Eya4	28113	19079.66667	18855.66667	18831.33333
Ezh1	37389	26592	34103	31703
Ezh2	29949	31618.5	29883.5	35676
Ezr	16361	24703	18943.5	28512.5
F10	26158	978	27135	7594
F11	30387	31344	30665	27869
F11r	23085	22586	24014	32210
F12	18319	5985	24080	23317
F13a1	10812	34572	6187	22935
F13b	40648	36835	2097	3404
F2	28891	3198	32859	34384
F2r	26779	17262	28011	38007
F2rl1	28020	29413	31424	29911
F2rl2	33681	35192	4917	1546
F2rl3	10035	16174	14895	16517

F3	36791	35965.5	19506.5	19523.5
F5	11374	23939	10242	28718
F630003A18Rik	23410	24471	26836	33629
F630111L10Rik	27862	28890.75	29789.25	29984.75
F7	24255	22707	23618	24882
F730002C09Rik	2663	23623	38283	28488
F730016J06Rik	27991	29240	31143	29797
F730043M19Rik	40236	17722	2392	20443
F730046H10Rik	10692	12251	14994	17125
F8	19464.5	33100.5	22880	29289
F830002E08Rik	357	36818	35198	3390
F830002L21Rik	24249	25513	26775	27617
F830004M19Rik	5730.5	3627	33726.5	4602
F830005D05Rik	25921	27467	32942	6057
F830005K03Rik	31281	32991	34627	33101
F830010H11Rik	5734	38456	3182	9553
F830014O18Rik	38038	33678	41021	39974
F830045P16Rik	18493	23424	24313	31105
F8a	2419	2934	2533	216
F9	15836	13041.5	32072.5	16634
Fa2h	36300	36219	3385	38454
Faah	13407	20595	16110.5	14364.5
Faap100	12673.5	26261	28248	22692
Faap24	16996	17424	12972	15524
Fabp1	5088	19137	10453	10822
Fabp12	26753.5	28897.5	34154.5	33774.5
Fabp2	19876.5	23020	24742.5	25017
Fabp3	23346	21290	24957	21878
Fabp4	12551.66667	10317.66667	16581.66667	8867.666667
Fabp5	18382	18387	17417	14984
Fabp6	32371	3873	4206	14963
Fabp7	32798	21225	4707	38940
Fabp9	21135	20149.5	27216.5	7277.5
Fadd	19360.5	19434.5	17824	18342.5
Fads1	21298	21705	18552	18229
Fads2	16503	36001	20746	33751
Fads3	23923.5	30093	28249.5	14179.5
Fads6	36587	35621.5	22803.5	3453
Faf1	14616	18798	17743.5	18506.5

Faf2	28375.33333	9111.666667	21969	6872.333333
Fah	12842	13903	9927	11230
Fahd1	40408	7651	5304	8834
Fahd2a	10132	15823	11880.5	13054
Faim	40489	3339	40310	1412
Faim2	12882.66667	15690	26284.66667	18924.33333
Fam101a	29707	30964	32907	31350
Fam101b	35209.5	24478.5	22110	19160
Fam102a	7299	7473	2839	448
Fam102b	23825.5	6889.5	11947.5	20134.5
Fam103a1	7285.666667	8546	9203.666667	19994.66667
Fam104a	11652	13918	12220.5	15038.5
Fam105a	19404	16522.5	15553	12223.5
Fam107a	560	6026	10013	4042
Fam107b	10246.33333	12207	11154.66667	25449
Fam109a	11373	12108	14658	15335.5
Fam109b	13094	16053	15285	16359
Fam110a	26028	18714	19130	24627
Fam110b	22871.33333	30591.33333	21659.66667	30977.66667
Fam110c	21160.5	24965.5	21847.5	15220
Fam111a	27661	19452	25909	23254
Fam114a1	11904	18765	13574	17758
Fam114a2	12834.5	31603.5	13019.5	32488.5
Fam117a	5078.5	974	24969.5	17118.5
Fam117b	23977.75	20051.75	25618.75	21583.5
Fam118a	33959	37243	35179	38066
Fam118b	29004	27273.5	28546	29361.5
Fam120a	16520.25	21513	22815	20895.5
Fam120b	17646.25	11773	15801.25	14399.5
FAM120C	26011	25958.75	29544	22228.5
Fam122a	2638	1444	39214	39741
Fam122b	26488.5	21634.5	25359	24269
Fam122c	6008.5	12304	23733	6596
Fam124b	39312	40973	7366	7263
Fam126a	22392.33333	21899	26154	27199
Fam126b	25609.5	24814.25	24572	23471
Fam129a	37927	24995	30045	20134
Fam129b	21018	13135	21782	15122
Fam131a	36729	40868	36735	38578

Fam131b	3906	8224	7389	8368
Fam131c	2857	35760	778	8779
Fam132a	22702	16582	18486	11889
Fam132b	1752	12184	437	32977
Fam133b	19661	31421	30144.5	28193
Fam134a	39268	38674	39362	36998
Fam134b	23359.5	20284	17325.5	17719.5
Fam134c	20359.5	19364	18767	21234
Fam135a	12017.5	15560.25	12392	16230.5
Fam135b	11995	10094	22314	21209.5
Fam136a	10846	18198	13833	19140
Fam13a	12789.66667	18737.33333	18145.66667	21287.66667
Fam13b	10961.66667	18528	18728	30819
Fam13c	25284.33333	27135.66667	24134	24654
Fam149a	34509	29496	32770	28762
Fam149b	32839.6	27033.4	32097.2	32118.4
Fam150a	26995	34226	3428	38819
Fam151a	6717	4323	15040	15479
Fam151b	39810	2071	40394	37721
Fam159b	32700	34969	35890	34100
Fam160a1	33896	30502	5822	30295
Fam160a2	29433.5	21458.5	25052	22151.5
Fam160b1	16271.75	11002.25	18880.25	16604.25
Fam160b2	18441.5	17658	24971	20100.5
Fam161a	29131.5	16520.5	19528.5	16278
Fam161b	17740.5	16652.5	16850.5	20456
Fam162a	37586	16923	33288.5	34025.5
Fam162b	28153	29593	31821	37095
Fam163a	4040	16547	4991	20924
Fam163b	14183	15617	8999	13972
Fam166a	24793	25231	496	25888
Fam166b	32617	37588	6183	7245
Fam167a	30890	36398	36422	35466
Fam167b	33182	7181	1812	13220
Fam168a	18490	26158.33333	22457.33333	30830.33333
Fam168b	15592	6103	12594	7107
Fam169a	34969.33333	36945.66667	37288.66667	24038
Fam169b	24371	25681	27493	4158
Fam170a	34819	10815	9502	11827

Fam170b	17742	19324	25070	13011
Fam171a1	32244.5	25970	20393	31564
Fam171a2	10431	12812	16061	17044
Fam171b	17796	20490	21629	24398
Fam172a	16017	24637.16667	24616	19788.33333
Fam173a	17595	18598	17248	17441
Fam173b	16089.5	13260.5	17010.5	15925.5
Fam174a	19392	14358	10989.5	12391.5
Fam175a	20432.33333	27597.66667	21018.33333	22963.66667
Fam175b	16506	13960	11357	13512.5
Fam177a	19421	20196	19559.33333	19892.66667
Fam178a	13188	9220	10895.25	9001.5
Fam178b	21798.33333	26061	30968.33333	31334
Fam179a	10637	11291	19021	18402
Fam179b	11457.5	26238.5	23525.5	22764.75
Fam180a	36208	26697	37133	39375
Fam181b	13512	7803	24508	19085
Fam183b	13562	14526	18418	12470
Fam184a	16581	31995.5	34511.5	33896.5
Fam184b	22131.33333	11526.33333	28275.66667	13637.33333
Fam185a	24291.66667	24586	22661	22467.33333
Fam186a	4815	1889	18776	15393
Fam187a	14383	17391	15420	16151
Fam187b	29480	4579	21607	10867
Fam188a	24555.66667	25500.5	21298.16667	21664.66667
Fam188b	13955	14493	10136	13745
Fam188b2	37672	36225	31285	29790
Fam189a1	9421	8428	9897	8257
Fam189a2	7563	32041	39692	39260
Fam189b	26068.5	22879.5	24660.5	22418
Fam192a	28303.66667	28300.33333	23813.66667	28842.33333
Fam193a	20312.66667	29379.33333	27155.66667	24151.66667
Fam193b	18546	15604	18557	19071
Fam195a	19041	21626	20690	19931
Fam195b	38826	36063	32278	34298
Fam198a	18356	19153	20806	17505
Fam198b	6884	26005.5	24822	16569.5
Fam199x	20062	16600.5	18357	16720
Fam19a1	21240.66667	33748.33333	21994	23740.66667

Fam19a2	19657.33333	34453.33333	23812.66667	23739.66667
Fam19a3	24920	27504.5	34753.5	40015
Fam19a4	24540	25806	27267	26318
Fam19a5	82	35117	37609	11738
Fam204a	8320	20072	19307	18057.5
Fam205c	7746	16481	32317	9699
Fam206a	36252.5	22737.5	15839.5	20893.5
Fam207a	15326.66667	28536	25601	28627.66667
Fam208a	2940	27275.8	25354.6	25816
Fam208b	14188.33333	13306.66667	26396	17132.33333
Fam209	40522	38400	41056	39365
Fam20a	32999.5	21968.5	35932	28369.5
Fam20b	21040.75	18069	20186.75	22002.5
Fam20c	29643	28207	18996.5	22760
Fam21	29886	27184	27334	26209
Fam210a	20604.5	31711	28470.5	13997
Fam210b	24779.5	24370.5	21523.5	21124.5
Fam212a	20897	39593	34275	38691
Fam212b	26798	16373.5	18191.5	10323
Fam213a	14289	12660	15923	7822
Fam213b	9711	14383	12918	14808
Fam214a	7308.5	15585.5	19936.5	13996.5
Fam214b	36241.5	37488	32911.5	32977
Fam216a	6350	9077	7111	9187
Fam216b	29929	37611	7903	33211
Fam217a	1366	6735	12762	12423
Fam217b	876	259	36463	40143
Fam219a	968	40065	39979	39865
Fam219b	23125	17522.5	16228	13495
Fam220a	6877.5	8893	8227.5	7812
Fam221a	37534	18695.5	31174	38220.5
Fam221b	37359	33985	35226	37899
Fam222a	38829	6422	23306	40694
Fam222b	19987.66667	25627	2944	16250.66667
Fam227a	5786.333333	7808.333333	20974.66667	6290
Fam227b	1436	22886	9647	7210
Fam228a	2761	35829	39151	3316
Fam228b	21602	22951.5	29470.5	33263
Fam229a	17515	35428	38293	33063

Fam229b	33760	22796	26522	20058
Fam234a	23030	28250	23150	34691
Fam234b	19355.5	19593.75	18960.25	9012.25
Fam24a	3688	6513	7800	10694
Fam25c	14814	9013	6994	6189
Fam26d	27884	31831	1121	29161
Fam26e	32632	40168	35848	37583
Fam26f	28937	11456	19499	39959
Fam32a	22956.5	6425	26035.5	24970
Fam35a	15273.33333	23626.33333	17988.66667	19393.33333
Fam3a	14343	12732	9960	11869
FAM3B	35982	1304	245	4873
Fam3c	24474	7754	25694	24741.5
Fam43a	9048	14149	16048	15971
Fam45a	16360	19428	16984	20036
Fam46a	1289	36745	41049	35172
Fam46b	22892	25814	27404	33096
Fam46c	19328	27670	17029	30164
Fam46d	26258	27570	29268	28057
Fam47e	33311	38611	4098	10631
Fam49a	10718	10120	5874	2649
Fam49b	20446.66667	17540.5	13067	10118.66667
Fam50a	5840	7630	13961	16610
Fam50b	6	36098	4694	36871
Fam53a	29410.5	29359.5	26904.5	24556
Fam53b	24959	22540.5	16714	17129.5
Fam53c	2202	40835	40897	1186
Fam57a	14115	18518	19465	20568
Fam57b	6456	9367	11792	12526
Fam58b	36460	38164	38004	33351
Fam60a	33798.5	19683	17964.5	19307
Fam63a	21392.2	16687.2	20248	16958.4
Fam63b	1378	38732	32267	25916
Fam64a	3138	41001	7044	5074
Fam65a	27495	21898	25756	24668
Fam65b	19574	19607	20006	20111.5
Fam65c	4989.5	11867.5	22046	11359.5
Fam69a	9625.666667	19027	19160	16594.33333
Fam69b	10025	2670	15678	16845

Fam69c	29278	30623	32389	30690
Fam71b	23460	24536	38024	25217
Fam71d	14993	14892	16681	22111
Fam71e1	6059	9510	11743	12190
Fam71e2	29170	30603	1175	30096
Fam71f1	29248	4998	8191	6184
Fam72a	17922	20936	20784	23490
Fam73a	23993.66667	33438.66667	16269.33333	28996.66667
Fam73b	11789.5	11770	9166	11407.5
Fam76a	21750	20308	18747	17687.5
Fam76b	22626.5	19237	21269	20468.5
Fam78a	14747.5	14815.5	11667.5	9493.5
Fam78b	27448	13145	1524	35869.5
Fam81a	17337	18043	25943.5	22200.5
Fam81b	26695	28243	29426	28144
Fam83a	18764	19581	25212	24478
Fam83b	26853.5	28174	34484	29492.5
Fam83c	38825	33315	39171	34048
Fam83d	37628	2611	4241	1842
Fam83e	10114	13602	11891	15365
Fam83f	12502	7142	13981	9797
Fam83g	27264	35671	27539	37222
Fam83h	16346	22879	20981	29801
Fam84a	28509	31671.5	37393	30054.5
Fam84b	17826	12492	16996	11419
Fam89a	7416	9487	11150	11431
Fam89b	40358	40657	37106	36693
Fam8a1	16352	16553	21336	21734
Fam92a	20571.75	20111.25	27299	26380.25
Fam96a	18449	19190	18882	18486
Fam96b	11053	12831	10634	13035
Fam98a	22539	23351	23095	24128
Fam98b	26322.66667	28043.66667	24078.33333	25519.33333
Fam98c	23692	20410	24746	22322
Fan1	28336	20724	25325	26022
Fanca	29095.5	27758.5	12579.5	8236
Fancb	10458	10408	10469	9285
Fancc	22699	23597	22149	23795
Fancd2	7159	15595.33333	7397	8937.666667

Fancd2os	28052	29590	607	39219
Fance	14670	5969	13162	2589
Fancg	17293	18038	19261.66667	17381.33333
Fanci	19455.5	35803	19754	19669.5
Fancl	22529	24336.5	20222	21231
Fancm	14695.33333	10801	24042.66667	24181.66667
Fank1	24527	27648	31547	29779
Fap	16592.5	17162.5	19828	20022
Far1	20529.83333	18702.33333	22924.5	21495.5
Far2	11716.66667	19344.66667	19409.33333	13005.33333
Farp1	16191	15594.66667	23988	14976.33333
Farp2	5231	7624	7781	6734.5
Fars2	8516	17023.5	25914	24058
Farsa	14191.75	19208.5	16730.75	18858.25
Farsb	8846	12211	9530	13407
Fas	9527	10287.66667	13393.33333	7094
Fasl	6369.5	7263	14256	12446
Fasn	6945.5	12307	3262.5	4404.5
Fastk	9143	11701	8436	10308
Fastkd1	19296.66667	20073.66667	20173.33333	13278
Fastkd2	16814.5	16009.5	15506	18114
Fastkd3	33734.5	36466.5	27505.5	35706.5
Fastkd5	32853	32216	26481	31516.5
Fat1	18514.33333	20940.66667	11248	13574.33333
Fat2	15680	16437	19741	21975
Fat3	16407.44444	24468.55556	22826.55556	20479.66667
Fat4	29399	10305	14128	9283.333333
Fate1	20395	27391	40770	40992
Fau	13594	9720	17484	14168
Faxc	25610.66667	28910.66667	18821	23035.66667
Fbf1	22869	25943	25875	32340
Fbl	22284	21991	24416	26265
Fblim1	29335	27041	9906	7354.5
Fbll1	38219	3791	40789	1419
Fbln1	31464	33337	19044	19259.5
Fbln2	21078	19647	28317	35944
Fbln5	22986.33333	27406	25398	30904.33333
Fbln7	6795	11160	9562	12084
Fbn1	20090.33333	22107	24128.66667	16707.66667

Fbn2	14122	15939	19879	19741.5
Fbp1	18473	18557	24092	28888
Fbp2	1342	38791	7707	1831
Fbrsl1	16476	13333	12557	10149
Fbxl12	36190	25534	25576	24680
Fbxl13	524	5323	7852	17202
Fbxl14	18138.5	21119.5	20997	17262
Fbxl15	11034	13022	12916	12193
Fbxl16	31202	36279	2915	1359
Fbxl17	17508.14286	18679.28571	19435.57143	18790.28571
Fbxl18	8220	4926.5	21649	24206
Fbxl19	24171.5	24695.5	26696.5	7206
Fbxl2	21318.5	19652.5	17903.5	17099
Fbxl20	23282.5	20669.25	18508	18298
Fbxl21	15838	23390.33333	27300.66667	23769.66667
Fbxl22	18708	17792.5	23397.5	21659
Fbxl3	5276.5	9371	8534	12334
Fbxl4	4858	4117	3525	6528
Fbxl5	13905.75	15607.5	18777	19839
Fbxl6	7735	6732	2570	1991
Fbxl7	23319.5	11903.75	22171.5	22724.5
Fbxl8	3266	471	526	39838
Fbxo10	21837.5	18275	2177.5	38224
Fbxo11	15075.75	11604.75	14419	16143
Fbxo15	27893	29371	32337	32813
Fbxo16	26665.5	27711.5	30537	16273.5
Fbxo17	10400	9756	17060	17346
Fbxo18	29743	25791	28280	28172
Fbxo2	25395	25729.33333	27878.33333	29799.66667
FBXO2	17612	18532	14988	18599.5
Fbxo21	27735	21484.5	25203.25	20398.75
Fbxo22	15743.5	19240.5	15216.5	18678
Fbxo24	19553	23892	25105	30338
Fbxo25	33089.5	25345.5	23306.5	26219
Fbxo27	20948	23445	32383	33207
Fbxo28	10234	19762	7508.666667	17940
Fbxo3	18882.66667	17908.33333	20120	19163
Fbxo30	20232.66667	24902	20223	25522
Fbxo31	26013.5	26477	21583.5	24549

Fbxo32	40954	32059	6077	37776
Fbxo33	8462	7940	6236.666667	8868.666667
Fbxo34	15334	15469	18422	16135
Fbxo36	25550	19741	23371	20251
Fbxo38	39573	39655	1092	2083
Fbxo39	32284	21076	17254	24215
Fbxo4	31614.5	27386	32371.5	31270.5
Fbxo40	10751	23816.5	28460.5	30123
Fbxo41	21762	25752.5	33513	32886
Fbxo42	12582	13523.5	10491.5	12861.5
Fbxo43	27412	28770.5	30479	29027
Fbxo44	22146.5	27012	20859	29549
Fbxo45	27378.5	27387	27919.5	32176
Fbxo46	3663.5	3022.5	6909.5	3995.5
Fbxo48	28099	29564	31328	35062
Fbxo5	26298	21988	24523	22939
Fbxo6	16939	20297.5	20707	23645
Fbxo7	27518	26248	28058	16187
Fbxo8	18542.5	14356	17904	16538.5
Fbxo9	22054	22680	20393	23339
Fbxw11	23673.5	18352	18241	20398.5
Fbxw13	7030	36209	425	40466
Fbxw14	13579	22706.33333	18600.33333	21651.66667
Fbxw15	23914	30614	1185	39104
Fbxw16	24873	25964	28348	27145
Fbxw17	36852.5	38074	36583	18526.5
Fbxw18	35980	3701	29659	37303
Fbxw19	20917	25353	35029	30722
Fbxw2	17870.66667	20458.66667	16109.33333	17787.33333
Fbxw21	23417	25673	13279.5	14242
Fbxw22	9277	17153	20860	23269
Fbxw24	31923	34148	39266	39391
Fbxw26	32245	36438	4178	4096
Fbxw27	17818	17769	18676	18550
Fbxw4	20602	30821	32311.5	12729.5
Fbxw5	10686	8417	358	465
Fbxw7	11783	26363	26878.5	13734
Fbxw8	23985.5	24475	24938.5	25068
Fbxw9	22689	22502.5	3648	3066.5

Fcamr	1447	15608	7187	15013
Fcer1a	7343	10555	14080	15129
Fcer1g	15975.5	15870.5	13685	20161.5
Fcer2a	8935	10384	6685	7042
Fcf1	34331	39594	32575	37800
Fcgbp	30351.5	29806.5	33427	32271
Fcgr1	19578	28851	19183.5	22669
Fcgr2b	21464.33333	23828.33333	22541.66667	25264.33333
Fcgr3	29257.5	21827.5	26642	23780
Fcgr4	9712	31042	5458	24144
Fcgrt	20893	9271	22260	13545
Fcho1	27285	24174	16907.5	15826.5
Fcho2	13410	35299.33333	21904.33333	21242.66667
Fchsd1	8099	8040	13307	13086
Fchsd2	21651.33333	21956.33333	37659	23972
Fcmr	38648	38425	30731	36577
Fcna	10476	8360	14971	11748
Fcnb	10658	15554	34121	26393
Fcrl1	15911.5	30844	5613.5	22563.5
Fcrl5	11392.5	28715.5	29793	34021
Fcrla	22008	14335	18928	14358
Fcrls	14912.5	22716	17193.5	3363
Fdft1	29660	6590.5	31607.5	30690
Fdps	23328.5	28819.5	22907.5	26535.5
Fdx1	15495	21635.66667	17496.66667	18034.66667
Fdxacb1	2364	6653	1346	3823
Fdxr	38066	37126	36053	33961
Fech	17558	19004	17308	18782.5
Fem1a	11045	11012.5	10550.5	10509.5
Fem1b	10504.5	7002.5	11066.5	12299.5
Fem1c	14827.25	15169.75	17159.5	23076
Fen1	18064	17880	17888	15309
Fer	29803.5	29667.75	30248.5	34148.25
Fer1l4	20915	25081	33096	37384
Ferd3l	21172	22262	7626	10688
Fermt1	18255	16153.5	33755.5	34387.5
Fermt2	5165.5	6931	10735.5	16390
Fermt3	5015	499	39346	38451
Fes	11412.5	6584	7173.5	24587.5

Fetub	21753	21681	25047	25132
Fev	16347	18756	21561	23460
Fez1	38654	40257	6177	5361
Fez2	1949	40310	3823	2110
Fezf1	31482	32928	34520	32722
Fezf2	25734	27212	28150	27158
Ffar1	34714	26103	33561	33078
Ffar2	19161	18437	12630	16762
Ffar4	8198	6309	37605	32330
Fga	38253	2676	36592	6260
Fgb	19139	21944	27030	24904
Fgd1	13961	13442	21906	20515
Fgd2	23000	7572	14388	33248
Fgd3	20643	13639	12012	10501
Fgd4	11964.2	28078.2	23082.8	21069.6
Fgd5	31575	33213	35232	36652
Fgd6	33480	23361	36452	27961
Fgf1	22754	21671	32351.66667	32825.66667
Fgf10	33963	40769	40928	37382
Fgf11	17940	16051.5	24668.5	24011.5
Fgf12	12480	25644.33333	16007.33333	15283.33333
Fgf13	25759.75	18319	15136.75	15193.5
Fgf14	17538.5	19483	23664.75	15567
Fgf15	21668	13267	23803	17392
Fgf16	32937	35063	35238	37078
Fgf17	5981	38878	14534	9091
Fgf18	8367	9598	11768	11422
Fgf2	13341.5	14198.5	20940	17667
Fgf20	35209	1838	35912	6441
Fgf21	33735.5	21585.5	6213	20635
Fgf22	10420.5	10415.5	18249	10966.5
Fgf23	20089.5	20562.5	24859.5	24712.5
Fgf3	3560	32797	36662	1160
Fgf5	27651	29049	33609	29651
Fgf6	18676.5	2068.5	22524.5	10634
Fgf7	1175	640	5068	5984
Fgf8	4398.5	3478.5	9792	10545
Fgf9	40059	28797	7061	36209
Fgfbp1	16917	19898	25770	23447

Fgfbp3	20801	7441.5	7577	6452
Fgfr1	21242.75	14905	25604	21518.5
Fgfr1op	4488.5	1722.5	37453	1692.5
Fgfr1op2	9465.5	10437	29369.5	30196
Fgfr2	16388	29997.66667	32903	8688.333333
Fgfr3	24877.33333	24349.66667	25200.66667	13726.66667
Fgfr4	13732.33333	15263.66667	7647.666667	7002.333333
Fgfrl1	15095	16651	17865	19625
Fgg	5947	7964	9468	11997
Fggy	16042.5	15369.5	12259.5	13933.5
Fgl1	18163	7138	25022.5	24203.5
Fgl2	9341	3879	4617	31632
Fgr	18746	36018	16215	23968
Fh1	28553	25953	23997	25496
Fhad1	15926.33333	19564.66667	18385.33333	24804.33333
Fhdc1	4134	21777	9097	31851
Fhit	1907	40343	6361	3200
Fhl1	34104	35130	32807	34603
Fhl2	21192.66667	21913	24732	26818
Fhl3	22621.5	25369	12889.5	15563
Fhl4	36232	32052.5	37723	37522
Fhl5	17319.5	21584.5	8082	9358
Fhod1	10215	8619	5448	8330
Fhod3	20745.66667	20794.33333	22634	23882.33333
Fibcd1	1930	2893	6739	3618
Fibin	5000	7561	11416	10412
Fibp	21809.5	18295.5	18084	20672.5
Ficd	19841	23091	21507	21016
Fig4	20488	18764	19542	16575
Figf	13660	13166	20437	34976
Figla	25884	27321	28992	27672
Fign	25906.66667	28407	23252.33333	24772.33333
Fignl1	17825.33333	20267.33333	18907	18147.33333
Filip1	8840	17570	20888.5	29612.5
Filip1l	4803	32285	7352	33899
Fip1l1	15654	17790.33333	20651.33333	21470.33333
Fis1	22222	24670	23053	26629
Fitm1	40294	39385	6248	6428
Fitm2	24037	12142	23627.5	13685.5

Fiz1	6567	5576	7236	3815
Fjx1	29803	39153	1720	9109
Fkbp10	12208	10913	17630	23867
Fkbp11	20286	19665.5	15057.5	13819
Fkbp14	19311.5	16943.5	26835.5	7345.5
Fkbp15	29964.83333	22170.33333	22208.5	24102.16667
Fkbp1a	33088	35492.5	33003.5	34240.5
Fkbp1b	23196.5	20189.5	19841	36102
Fkbp2	16084.5	20587.5	21055.5	22741.5
Fkbp3	38812.5	21469.5	17657	18966.5
Fkbp4	15522	20452.5	13703.5	15705.5
Fkbp5	28415.66667	19786.33333	23216.66667	12153
Fkbp6	17692.5	19470	10083.5	9477
Fkbp7	20085	16166	21794	16538
Fkbp8	12987	12883	10766	11523
Fkbp9	12565	17206	16195	19647
Fkbpl	19352	21524	21624	23116
Fkrp	23173	23367	20953	19006
Fktn	11281.33333	23476	22055.33333	26037.33333
Flad1	25926.5	25984.5	26367.5	27209
Flcn	11398	7036	9185	4041
Flg	35024	20886.5	15639.5	29730
Flg2	4326	6395	5091	5537
Fli1	35230.66667	26115.33333	31601	25237.33333
Flii	12766	9496	12526	9919
Flna	23207.25	21243	21099.25	20169.75
Flnb	8747.2	10892.4	20675.8	12444
Flnc	14141.5	22778.5	4932.75	5647.5
Flot1	34427	37952	30566	35136
Flot2	20462	19564	18433	18663
Flrt1	9584	10984	40975	4759
Flrt2	16103.5	9161	13922	12428.5
Flrt3	5779.5	31625.5	8459	23081.5
Flt1	15462	26582	16079.4	24302.6
Flt3	24801	32429	1427	2809
Flt3l	14213	11466	15223.5	8756
Flt4	34234	32842	8418	5796
Flywch1	2877.5	2887	14396	35616.5
Flywch2	15479	22403	21161	23096

Fmn1	13200.2	5797.6	12555.4	16533.2
Fmn2	34415.5	34162	1616	22047
Fmnl1	1987	20822.5	19541	36730.5
Fmnl2	22951.66667	17656.33333	25748.33333	13320.33333
Fmnl3	4501	36915	4463	755
Fmo1	30198	15403	21557	21695
Fmo2	32058.66667	22245.66667	25273.66667	18286.66667
Fmo3	18087	20647	27823	28774
Fmo4	14003	23538	25399	29291
Fmo5	22196	12040.66667	17839.33333	8048.333333
Fmo6	25721	28115	27545	1436
Fmo9	31014	26689	28497	31825
Fmod	40656	1812	7668	8280
Fmr1	31653.5	25755.5	5631.5	3483.5
Fmr1nb	19598	19921	22592	23373
Fn1	11806.5	27778.25	22722.25	32309.5
Fn3k	25084.5	24863	19613.5	11145
Fn3krp	15502	33205.5	35748.5	17903.5
Fnbp1	18708	13494	19018.66667	11044.66667
Fnbp1l	29228.75	27094.5	22951.5	23019
Fnbp4	31260	27703	35206	33033
Fndc1	12617.25	13953.5	16641.25	18198
Fndc3a	16340.33333	15410	17989	10671.66667
Fndc3b	27187.5	23268	26262.25	26623.25
Fndc3c1	25936.5	13912	20505.5	16162
Fndc4	25525.33333	16483.66667	12462	7177.333333
Fndc5	17942	16862	20745	23218.5
Fndc7	5935	11585	7518	13285
Fndc8	11529	15588	24017	21216
Fndc9	16621	15584	21345	21259
Fnip1	10926.25	26700.25	8021	17714
Fnip2	22127	14438	23391	16134
Fnta	8894	11705	9589	11534
Fntb	19600.5	20182	20552.5	21202
Focad	25526	24461.5	20130	22906
Folh1	31153	32763	34343	32614
Folr1	13967.5	15064.5	20433.5	19249.5
Folr2	30943	29958	2921	33719
Fopnl	26851	28257	30476.5	21078

Fos	1375	26596	5213	40098
Fosb	13090	17686	19427	20584
Fosl1	37268	39051	4467	3810
Fosl2	25633	27755.5	27757.5	12297.5
Fosr	32856	36664	5732	9215
Foxa1	1225	34075	4064	9209
Foxa2	549	40600	4238	3248
Foxa3	761	12542	8291	13747
Foxb1	25997	3132	5762	4752
Foxb2	17032	11583	13026	12202
Foxc1	5510	2013	844	7604
Foxc2	9585	7470	18805	19220
Foxd1	26340.66667	5666	37992.33333	16693.33333
Foxd2	26386	24634	37404	39598
Foxd3	17500	19710	24763	19239
Foxd4	27057	32425	29964	28521
Foxe1	3429	39076	39500	36418
Foxe3	18563	20067	21989	22876
Foxf1	21040	25619	37394	35102
Foxf2	4750	9942	5123	11100
Foxg1	19052	25251.5	23356.5	23242.5
Foxh1	22135	21491	22217	20926
Foxi1	966	3772	33616	1033
Foxi2	38268	7543	6634	13266
Foxj1	6326	35781	9281	41089
Foxj2	18399	18295	23806	23493
Foxj3	26583.66667	32658	36125.33333	25488.33333
Foxk1	23652.66667	24829.66667	25785.66667	13269.66667
Foxk2	25178.75	26283.5	21239	22772.5
Foxl1	8373	6100	17969	18053
Foxl2	34318	37381	1056	40208
Foxm1	11481	10464	13332	12078
Foxn1	8233	11249	16344	16968
Foxn2	12121	12287.66667	13970.33333	16607.66667
Foxn3	17613.66667	21282	30855.66667	23346.66667
Foxn4	1682	8292	38147	5438
Foxo1	9784.5	6421.5	5092	2859
Foxo3	16050.5	25087.5	23906	21917
Foxo4	26040	25350	709	2072

Foxo6	33221	16906	23697	19221
Foxp1	16039	20742.16667	18543.33333	20301
Foxp2	17340	19807.5	21291.33333	13384.16667
Foxp3	39987	9161	15735	14638
Foxp4	24522	20832.5	2786.5	17327.5
Foxq1	18410	17810	19424	20717
Foxred1	28089.5	28080.5	22528.5	27902.5
Foxred2	19501.5	10559	12112.5	8768
Foxs1	30580	37408	39701	38576
Fpgs	17395	18183	19498	19493
Fpgt	32670.66667	29743	23531.66667	35382.66667
Fpr1	19978	28789	26575	30874
Fpr2	17043.5	17146.5	22377	18521
Fpr-rs3	17054	22323	34122	38912
Fpr-rs4	25883	27360	28475	27445
Fpr-rs6	31893	38931	34872	35935
Fpr-rs7	27242	28655	30091	29023
Fra10ac1	35318	38869	40258	39681
Fras1	7316	8419	13941	10241
Frat1	18531.33333	18285	19314.66667	15210.33333
Frat2	24581	24881	26375	23055
Frem1	32737	34109	2100	35158
Frem2	33421.5	33149	18811.5	24876.5
Frg1	37090	2074	466	699
Frk	19306.5	15352.5	22078.75	8911
Frmd3	40696	6812	17142	15439
Frmd4a	15437.75	18075	19895.5	12232.25
Frmd4b	19224.75	12784.75	19404.5	20940
Frmd5	16032.42857	18910.85714	15797.57143	20913.28571
Frmd6	16516.66667	15243.33333	17658	20205.66667
Frmd7	4846	31430	35877	37779
Frmd8	21462.5	13600.5	15958.5	12837
Frmpd1	9329	32052	10407	8030
Frmpd3	24550	25858	27062	26112
Frrs1	38327	24092	31304	26535
Frrs1l	15468.5	18613.5	21355.5	9339
Frs2	14852.33333	12228	14557.33333	14099.33333
Frs3	25737	33325	36306	36758
Fry	15474.8	24482	22908	33276.4

Fryl	25070.75	24812.25	28428	28808.75
Frzb	23957	39249	3404	14061
Fscn1	36496	1780	3675	9142
Fscn2	36561	3559	882	8043
Fscn3	18391	24068	19922	21276
Fsd1	7276	15237	14629	15771
Fsd1l	24804.5	22189	26572	25546.5
Fsd2	31256	32947	34662	32895
Fshb	5578	4468	13686	14941
Fshr	232	1275	40491	38190
Fsip1	18611.5	14748	20123.5	30820.5
Fsip2	30065.5	26912	28914.5	27628
Fst	4152	3622	30096	5303
Fstl1	6093	10000	27713	37613
Fstl3	13197	14026	18140	18724
Fstl4	3937	8940	11225	15777
Fstl5	34071.75	34106.5	21161	17598.5
Ftcd	20793	20804	33770	31146
Fth1	10379	13968	13910	20532
Fthl17a	38507	2771	2589	4796
Fthl17e	8574	3268	39040	38963
Ftl1	19908	19645	23089	21662
Ftl2	17924	18423	21105	20849
Ftmt	23490	24403.5	28789	26463.5
Fto	20256.66667	17696.33333	28092	28416.66667
Ftsj1	4597.5	15758.5	20143	21332
Ftsj2	18268	23146	21398	21292
Ftsj3	14382	15160	12180	13520
Fubp1	16677.5	20857	22486	29008
Fubp3	19044	19357	20110	19345
Fuca1	17280.5	10295.5	16093	9827.5
Fuca2	5155.5	6448	2698	5225
Fuk	12592	10625	10182	9298
Fundc1	29272	29169	25822	23515
Fundc2	17145	16532	13222	14299
Fuom	13058	21434	16660	21549
Furin	11643.5	7061.5	5987.5	6576.5
Fus	20775	17597	22116.25	21435.25
Fut1	6151	8257	15971	13809

Fut10	9757	7081	13666	11440
Fut11	12814	11409	10866	8550
Fut2	19598	19680.5	20954	24676
Fut4	25164	22090	18634	18763
Fut4-ps1	21475.5	24999.5	11703.5	11559.5
Fut7	14506	1985	14804	39165
Fut8	5106.5	21734	20629	20398
Fut9	30094.42857	30806.57143	34260.28571	32000.42857
Fuz	30085.5	30631	12240.5	13725
Fv1	38414.5	903.5	3743	4131
Fv4	34566	40178	1601	1477
Fxn	14336	15976	17076	15510
Fxr1	15634.5	20674.75	23798.5	22123.75
Fxr2	2842	4891	701	40328
Fxyd1	21694.5	22752	9935.5	6992.5
Fxyd2	16186	37938	17938	39550
Fxyd3	6341	5782	39823	39695
Fxyd4	36919	1547	7190	7701
Fxyd5	20355	16876	19292	14491
Fxyd6	5279.5	4888.5	11576	12130.5
Fxyd7	6237	21	6039	11170
Fyb	19492	18282.5	20168	17978.5
Fyco1	19259.5	21633.16667	15528.5	22114.33333
Fyn	17492.5	11876.5	22817.5	19719
Fyttd1	23274.5	29893.25	21645.25	13561.75
Fzd1	17845	20787	15045	17797
Fzd10	26071.5	22581	26769	24679
Fzd2	26627.5	25420.5	29261.5	17819
Fzd3	33951.33333	33417.33333	10660.66667	22149
Fzd4	20650	15019	22988	19453
Fzd5	14235.5	38904.5	18992	16312.5
Fzd6	23376	26205	32697	36635
Fzd7	25728.5	13217.5	20980.5	13986.5
Fzd8	29532.5	29898	33188	31242.5
Fzd9	11714	12941	13196	16980
Fzr1	16436	20723	18360	21791.5
G0s2	21582	25007	36514	32068
G2e3	20442.5	21525.25	13621.5	17871.25
G3bp1	19137.66667	21244.66667	17198	20202.66667

G3bp2	14036	34153.25	24492.5	24397
G630030J09Rik	28196	29842	30954	37466
G6pc	6543	9034	11712	11076
G6pc2	33071	37784	39997	8814
G6pc3	35253	31071	25792	26230
G6pd2	21285	19267	11538	17094
G6pdx	20486	18886	18224	20815
Gaa	5062	4517	3047	3411
Gab1	29882	24748	29150	25092
Gab2	16430.5	31782	34578.5	29698.5
Gab3	37690	23046	29485	19773
Gabarap	18319.5	14000	10999	13236.5
Gabarapl1	19503	15679.5	18035.5	20663
Gabarapl2	12857.33333	11258.66667	25650.33333	25894
Gabbr1	28849.25	22209	4762	13826.25
Gabbr2	25291.66667	16894	21930.66667	31099
Gabpa	5014	6367.666667	5618.333333	6260.333333
Gabpb1	16618.5	23909.5	22117.25	24444.25
Gabpb2	18928.33333	28425.33333	29992.33333	17904.33333
Gabra1	36435.33333	25762.33333	31774.33333	21970
Gabra2	19697	20892.25	23150	24755.25
Gabra3	32276.33333	29173.33333	31781.33333	32225
Gabra4	37757	40248	7628	8504
Gabra5	25003	24916	28746	13093.5
Gabra6	7106	5182	1816	27007
Gabrb1	27751	29100	32000.5	30091
Gabrb2	32115.25	16777	24397.75	19162.25
Gabrb3	32472	36744.75	26290.5	34286.75
Gabrd	8948	7957	15214	10297
Gabre	19972	25707	31011	28985
Gabrg1	31374.5	30999.5	33003	32216
Gabrg2	23269	19679.8	25154.6	23602.6
Gabrg3	28869.5	33128.5	35169	20294
Gabrp	14755	18238	36782	33892
Gabrq	31326	38191	34744	7771
Gabrr1	16557	18916	23390	23126
Gabrr2	14512	19378	18620	21820
Gad1	18841	21747.66667	32611	34132
Gad2	29988	33199.5	18518	1326

Gadd45a	3962	33438	4608	28184
Gadd45b	13237.5	10259.5	15637.5	14815
Gadd45g	34264	16976	25455	17527
Gadd45gip1	38507	3918	2536	4578
Gadl1	29619	31189	32024	30652
Gak	2679	38997	2605	40256
Gal	13924	18613	19809	23189
Gal3st1	9907	3185	13299	14393
Gal3st2	23503	37532	9375	4319
Gal3st4	30850	25372	27300	26235
Galc	21816.66667	11556	13506.66667	22782.33333
Gale	22688	24328	23799	22102
Galk1	26677	23339	25819	24590
Galk2	39923.5	17546.5	34366	36273
Galm	9503.5	27025	26315.5	28576
Galns	39217	24185	34172	25156
Galnt1	13636	12106	11791	12130
Galnt10	16814.66667	14283	17808	12780.66667
Galnt11	15672.5	12545	16559.5	13090.5
Galnt12	38076	30631	40201	34427
Galnt13	30292.6	26503.8	24220	22691.4
Galnt14	25267.5	11617	9612.5	14753
Galnt15	35269	14951	124	21859
Galnt16	13239	13625	24397	23351
Galnt18	5699.5	26381.5	3353.5	9521.5
Galnt2	11165.5	8570.5	26678	8048
Galnt3	21454.33333	18201.33333	20168.33333	23505.33333
Galnt4	4873	22286.5	8822.5	7018
Galnt5	19517	24296.5	22390.5	21736
Galnt6	13663.5	11454.5	13459	14350.5
Galnt7	3078	20007	23866.33333	22637.66667
Galnt9	14905	16324	13092	17631
Galntl5	37612	32482	33955	3935
Galntl6	21932	26853.66667	29184.66667	27732
Galp	21407	24103	25478	34056
Galr1	21535	10493	29524.5	28454
Galr2	37300	31127	1040	40814
Galt	2028	2216	39610	37568
Gamt	13376	10584	10241	6440

Gan	1800	35556	39939	32529
Ganab	19728	19245.5	38429	37894
Ganc	15451	20605	18145	15433
Gap43	34122	656	36656	34116
Gapdhs	13952.5	15714	21651.5	20993.5
Gapt	8020	26322	36082	19278
Gapvd1	21252.66667	13743	14483	26342
Gar1	10557	15116	14305	13931
GAREM	31446	29779.5	19773	18486
Garnl3	18111.25	19683.25	24344.5	25277
Gars	11779	8851.5	24992.5	10453
Gart	10798.33333	13715.66667	7163.333333	11873.66667
Gas1	22620	35625	3352	9818
Gas2	13211	23971	11417	19781
Gas2l1	34059.66667	21556	8094.333333	18578.66667
Gas2l3	26584.5	22614	25552.5	24204.5
Gas5	4760.5	3546.5	19161	19432
Gas6	15281	40849	11813	40559
Gas7	25206.5	17658.5	17998	12485
Gas8	7022	7627	10852	10936
Gast	12883	14472	18704	18206
Gata1	21547	22123	27547	28846
Gata2	22360	19674	22992	27685.5
Gata3	24652.5	18533.5	21468	21900.5
Gata4	17920	19287.5	22027	22644.5
Gata5	14082	14948	15916	13579
Gata6	17442	23721	9503	17646
Gatad1	23648	25181.33333	24296	21928
Gatad2a	16673.5	18231	18277.5	18051
Gatad2b	10353.5	3985	22226	20768.5
Gatb	12186	10952	10349	10808
Gatc	25423.33333	25311.33333	25759	24943.66667
Gatm	26173	7777	19492	2539
Gatsl2	28225.5	23478	25856.5	19518
Gatsl3	31335	27464	23179	25752
Gba	640	37844	38339	916
Gba2	14895	11866	12357	9437
Gbas	20825	22064	27471	25987
Gbe1	17710	26317.5	24338.5	12766

Gbf1	22802	17105	16371.33333	16982
Gbgt1	34997	17157	4558	18125
Gbp2	25362	21300	24710	21778
Gbp2b	3078	16511	10842	28231
Gbp4	14170	1580	15611	4550
GBP4	31609	19455	36549	24982
Gbp5	16969	6783	19936	1221
Gbp6	24943	22974	36250	28381
Gbp7	25627	966	20589	38182
Gbp8	17798.5	8207	22888.5	11463
Gbp9	22165	13409	23827.5	16606
Gbx1	6377	6549	6456	8122
Gbx2	21383.5	36229.5	19232	25326
Gc	17744	23288	25978	27217
Gca	18513.5	10231.5	17936	27222
Gcap8	31785	33568	41057	33002
Gcat	18493	19307	21735.5	21709
Gcc1	21275.5	23772	24078.5	20050
Gcc2	9994.6	18038	17433.6	19339
Gcdh	19066	17816	18407	17352
Gcfc2	25698	25652.33333	15452.66667	24114.33333
Gcg	22542	24709	9940.5	8710
Gcgr	26678	29130	36428	39044
Gch1	29080	10730	19797.5	14925.5
Gchfr	19965	24692	29164	24694
Gck	6272	8640.333333	22511.66667	12775
Gckr	36196	29519	2969	40485
Gclc	29144	29171	20980	26158
Gclm	12033	33773	10419	36513
Gcm1	35853	35909	38767	3999
Gcm2	26192	35662	36578	36315
Gcn1l1	7595.6	13087.8	18601.2	9454.4
Gcnt1	35565.25	24113	29516.25	20702.75
Gcnt2	19801	17531.66667	19996.33333	18883.66667
Gcnt3	27987	29626	30066	28885
Gcsam	21185	23369	36940	36086
Gcsh	822	7869	40317	37058
Gda	16095.5	28385	16970	27668
Gdap1	27938.5	29380.5	30743	32079

Gdap10	35169	40878	31244	2683
Gdap1l1	9275	10133	12546	14003
Gdap2	39322	40120	33778	36537
Gdap5	18939	19962	16406	25197
Gde1	4712	14291	8852	20528
Gdf10	26850	28020	30265	28976
Gdf11	8727	16034	12176	20099
Gdf15	8471	6811	7780	4847
Gdf2	38705	39845	33961	40361
Gdf3	28122	22873	39598	38100
Gdf5	3003	3155	6609	7737
Gdf6	23438	24512	26472	26148
Gdf7	22060	28360	33277	24560
Gdf9	20335	18477	20075	18693
Gdi1	9988	10174	7953	10973
Gdi2	26715.75	26700	27845	29981
Gdnf	15168	20714	21681	26193
Gdpd1	18580	5013.5	12000.5	18557
Gdpd2	38120	3258	3209	12740
Gdpd3	6659	4604	5824	3144
Gdpd4	31572	28639	30545	36642
Gdpd5	16058	13665	19686	13104
Gdpgp1	29777	27878	26236	21397.5
GE_BrightCorner	7766	6663	6010	7749
Gem	33536	24824	7125	34912
Gemin4	30572	30894	25924	26516
Gemin5	9211	8384	9003	8138
Gemin6	21951	28864	22653	23802
Gemin7	31899	31544	31107	30010
Gemin8	18346.33333	16511	16480.66667	16458.66667
Gen1	16174.25	24503	27606.5	22694
Get4	22091	19607	20747	21195.5
Gfap	24417.5	8875.5	26307.5	25495.5
Gfer	9566	13773	12315	13361
Gfi1	25940	1911	40015	28155
Gfi1b	33715	6331	28358	7842
Gfm1	20622.5	23921.5	22550	24185.5
Gfm2	11789.5	12613	17054	21196.5
Gfod1	8606.5	20637	14086	907

Gfod2	8274	10255	11052	12667
Gfpt1	19627.75	14025.5	13699.5	21244
Gfpt2	3265	6777	11368	12708
Gfra1	22629.5	7438	26320	10616
Gfra2	39019	39961	2194	39111
Gfra3	12018	14577	16782	15889
Gfra4	26289	17584.33333	19006	10943.33333
Gga1	11193	12744	15051	13583
Gga2	29162.5	36348	30191.5	20983.5
Gga3	28491.5	21419.5	24515.5	22660
Ggact	22075.5	21045	23219.5	14676
Ggct	14009	20009	18156	20427.5
Ggcx	14562	12778	11298	12243
Ggh	6739	16715	9942.5	12919
Ggn	4048	4237	16059	15735
Ggnbp1	22997.5	3109.5	3663	1700
Ggnbp2	35822	31665	31407	35090
Ggps1	16787.66667	20306.66667	15908	19602
Ggt1	4657	2149	38351	9323
Ggt5	27917.5	17710	24890	38189
Ggt6	19904	20945	24484	25967
Ggt7	28781	12996.5	21176.5	26204
Ggta1	38082	2138	29002	1032
Gh	21663	23434	9303	11781
Ghdc	22383.5	4791	20096.5	23422.5
Ghitm	18197.66667	8887.333333	17261.66667	9241.333333
Ghr	30389.66667	15803.83333	16997.66667	22251.83333
Ghrh	24577.5	25166	10849.5	9943
Ghrhr	2282	3186	9051	8546
Ghrl	9195	27289	11228	12378.5
Ghsr	33566.5	36033.5	19383	19167
Gid4	12454.5	16470.5	16670	16762.5
Gid8	13003	11768	10706	11930
Gif	31645	33386	39116	33358
Gigyf1	23239.66667	12976.33333	15399.66667	12411
Gigyf2	27076.16667	22910.16667	22285.5	21941.33333
Gimap1	28986	35968	2592	3076
Gimap3	28600.5	16654.5	28704.5	27746
Gimap4	1840	4733	4274	1984

Gimap5	5986	26132.5	13389	14652.5
Gimap6	39431	3057	1108	10404
Gimap7	32802	35001	21823	38968.5
Gimap9	30382	6214	3227	13927
Gin1	38137	22209.5	39376.5	19105
Ginm1	9768.666667	14098.33333	19542.66667	16750.66667
Gins1	28665.5	33121	14394	13876
Gins2	1303	4671	6414	2394
Gins3	22619	24554	23816	25403
Gins4	27051	32136	28083	28768
Gip	25449	20527	33214	23921
Gipc1	25274	23875	23938	23255
Gipc2	21836	22738	24998	22301
Gipc3	19183	14360	21347	15421
Gipr	27769.33333	23373.66667	29049.33333	34841.66667
Git1	3281	3143	1613	3393
Git2	20551.5	15968.75	14783.5	11207.75
Gja1	10645.75	16708.25	15550.75	19536
Gja10	32562	35370	40802	35377
Gja3	25984.33333	24448.33333	14533	5608
Gja4	5719	9182	13029	14809
Gja5	32495.25	22448.5	18464	16768.5
Gja6	25418	26658	28832	29945.5
Gja8	23391	24373	26733	38417
Gjb1	17553	17835	26676	26364
Gjb2	26033	10038	14288.5	20246.5
Gjb3	11965	15507	9615	16100
Gjb4	15909	16357	20176	21337
Gjb5	20726	17921	17091	34743
Gjb6	32609	35098.5	19971.5	18198
Gjc1	14812.66667	20942.66667	24475	17758.66667
Gjc2	24192	28649	36907	39446
Gjd2	31764	36573	35984	5740
Gjd4	3738	36051	12142	18150
Gje1	29108.5	31785	32672	31949
Gk	29368.2	22201.4	24787.6	22407
Gk2	39511	3889	4517	7338
Gk5	30432.5	26483.25	18506.25	26445.25
Gkap1	11337.5	17270.5	19707	23333

Gkn1	34843	30044	35150	37850
Gkn2	14628	20774	18169	23426
Gkn3	27775	24860	34567	32060
Gla	11744.5	12071.5	11060	11961.5
Glb1	10673	19917.33333	11001	20505.66667
Glb1l	19054.5	25120	32802	16945
Glb1l2	21741	22472	27976	35112
Glb1l3	26697	28300	28840	27828
Glcci1	25677.16667	29207	32236.66667	28279.66667
Glce	24924.33333	15940	10834	13149.66667
Gldc	36370	7230	35001	16394
Gldn	30597	32161	34134	32459
Gle1	30309.5	29489	27377	27270
Glg1	16421	11021.5	14347	8395.5
Gli1	20235	30835	24731	26540
Gli2	21857	30180.5	25910.5	25446
Gli3	24964.75	29992.75	29215	29067.5
Glipr1	16538	26176	16162	32836
Glipr1l1	29499	31052	33538	31836
Glipr1l2	25913	27396	28765	27598
Glipr2	34966	1873	23730	27605
Glis1	5605	1943	6595	13304
Glis2	22914.5	29112.5	28372.5	12168
Glis3	27424.33333	19868.66667	25095.66667	24394
Glmn	37946	3378	39645	38891
Glmp	19543.5	19455.5	19585	20529
Glo1	13625	14094	14083.5	14907
Glod4	91	40595	37520	37615
Glod5	12930	11772	20026	21619
Glp1r	24922	22020	26800	33102
Glp2r	17944.66667	19711.33333	20805.33333	18179.33333
Glra1	6684	8668	7314	4409
Glra2	2007	5409	2379	8243
Glra3	25075.5	27976	27640.5	33538
Glra4	34435	37162	27010	25887
Glrb	32641	34973	35289	33343
Glrp1	25559	39970	39158	5492
Glrx	25311	25046	20406	20795
Glrx2	36640.5	18714	20426.5	19164.5

Glrx3	12413.5	12618.5	9254.5	11350.5
Glrx5	7485	7346	6742	8078
Gls	18135.33333	16790.83333	11849.83333	18256.5
Gls2	3026	8689	5671	6200
Glt1d1	18848	19247	21823	22003
Glt28d2	23624.66667	24094.66667	10336.33333	19747.33333
Glt6d1	33336	27436	30345	28989
Glt8d1	40331	36767	38067	33654
Glt8d2	27304	28395	39017	20376.5
Gltp	10904	5619	4091	40004
Gltpd2	6536	5788	8608	13333
Gltscr1l	18384.5	16876	17525.5	15433
Gltscr2	8447	6042	21991.5	22265
Glud1	28685	20191	27051	23184
Glul	21491.66667	32419.33333	34152	25481.33333
Glyat	21294	19244	27776.5	25503
Glycam1	24194	25270	27385	26232
Glyctk	5022	6022	2742	3235.5
Glyr1	25105.66667	17547.66667	6246	19682.66667
Gm10033	7340	9036	12437	12425
Gm10044	1871	34421	40351	31466
Gm10048	29513	31080	31930	30549
Gm10055	5359	5420	7858	2038
Gm10083	29063	30240	37193	36096
Gm10101	33763	35853	36687	35769
Gm10220	5568	13318	4339	33917
Gm10319	32751	37231	1573	8
Gm10336	32436.5	26240.5	28787	27652.5
Gm10413	21620	17838	19816.4	19788.4
Gm1045	29460	35006	33457	36377
Gm10681	16397	19883	21173	21361
Gm10937	29845	36118	31945	31171
Gm11236	3144	17573	5426	4704
Gm11346	31449	2592	3261	32916
Gm11427	37444	39797	38452	39903
Gm11487	9871	15939	23235	18308
Gm11507	27654	28888	5686	3326
Gm11545	29912	36936	28244	37200
Gm11568	40332	3892	35459	37520

Gm11961	32253	32447	421	38311
Gm12185	28476.5	21833	25308	24156
Gm12504	37618	4834	5987	5826
Gm12541	38871	39973	4137	6493
Gm12569	36390	36820	3276	2146
Gm1257	7935	6738	13101	15738
Gm12580	32829	31976	32422	30995
Gm12680	24430	37040	14659	15782
Gm12725	5828	10112	8598	12640
Gm12799	33394	32050	23714	26291
Gm128	3055	10702	8974	14745
Gm13051	28927	35375	24257	32811
Gm13103	22669	29870	36182	38356
Gm13136	6533	39117	36661	35465
Gm13141	23280	8011	40625	35434
Gm13152	32163.5	16872.5	32630	17144
Gm13154	39686	41034	30040	37767
Gm1323	6697	7818	40084	313
Gm13235	34574	38468	24286	30734
Gm13490	27220	28511	31564	35746
Gm136	11072	15917	18308	18851
Gm13600	22754	25703	35368	35613
Gm13624	33342	36770	37528	36586
Gm13762	2926	11000	22049	11339
Gm13769	4130	9126	8389	7105
Gm13777	1157	6216	15542	12357
Gm14047	30255	31842	33307	31952
Gm14322	7575	9251	3957	9282
Gm14333	15579	20811	22972	20886
Gm14418	3043	13877	6104	13621
Gm14461	6837	36727	7049	39709
Gm14644	9756	11450.5	7742	10441
Gm14920	11905	11619	15431	12859
Gm15204	13829	6003	3219	40161
Gm15380	11197	10960	9039	7477
Gm1549	25930	27616	28629	39154
Gm15800	14565.66667	13788	10705.66667	22932
Gm16223	85	29563	36193	35822
Gm16294	26060	36466	38192	27993

Gm1631	26383	38675	1784	40047
Gm16475	30754	25949	28535	35217
Gm16516	4131	3681	4689	5519
Gm16523	40461	37647	3075	5144
Gm166	19714	16726	21374	16979
Gm16932	33744	33941	36392	34474
Gm17177	23695	32633	1243	36783
Gm17751	26220	36480	31837	28341
Gm1930	8759	14476	12723	12901
Gm1965	10778	10040	18509	19969
Gm1966	11403	5880	9101	10180
Gm20555	26145	28682	30051	28730
Gm20558	18989	18531	25426	24581
Gm20559	38166	5816	37732	6725
Gm20716	28385.5	12842.5	12751.5	13341
Gm2115	32528	34293	35343	33528
Gm21967	33053	23580	2699	39767
Gm22	38644	2790	7826	10420
Gm2447	33190	1712	7603	3289
Gm252	33321	32434	3817	32759
Gm270	2506	5458	9327	10464
Gm271	26508	24324	2660	35752
Gm2762	13597	15260	21175	19379
Gm2a	21804.5	40067.5	25890.5	21151.5
Gm3120	23615	18913.5	21111.5	20115.5
Gm3336	13764	16940	18315	18327.5
Gm336	36106	22261	11324	14754
Gm355	30741	32898	4188	40044
Gm362	37837	31481	1693	1188
Gm363	3330	12568	19351	20719
Gm364	32045	33686	36439	1207
Gm37240	22708.5	4041	19996	4580
Gm378	29980	31638	33783	32137
Gm42742	16619	15772	17088	15618
Gm454	26768	28037	29971	30637
Gm4736	20098	20956	34782	30717
Gm4745	39212	1018	30657	33210
Gm4755	29313	31311	31024	29633
Gm4758	10925	27168	29655	34086

Gm4759	27582	26429	29559	385
Gm4763	33551	33689	98	12978
Gm4767	13061	12664	13208	14400
Gm4776	36981	8412	16073	21353
Gm4779	26759	31791	25373	25007
Gm4782	3701	6537	40210	2518
Gm4787	9746	7288	14494	12712
Gm4788	36578	34527	40094	8795
Gm4792	28892	31142	30644	29464
Gm4793	16980	21421	26236	30095
Gm4794	24347	25484	27104	528
Gm4814	26822	28043	30097	28832
Gm4815	24233	1269	32094	2933
Gm4820	6269	9570	12298	13781
Gm4821	8075	11519	15937	14517
Gm4827	11109	10992	24684	26017
Gm4831	6785	5602	14848	3740
Gm4861	29392	31232	31431	30002
Gm4862	20844	18243	25234	22521
Gm4870	5089	10459	8048	10954
Gm4876	24454	25583	27609	26430
Gm4884	26441	27807	30551	886
Gm4890	29889.5	36620.5	34996.5	34491
Gm4894	37368	3997	3922	13240
Gm4899	19446	23215	19438	23744
Gm4916	21670.5	25537.5	6770	13302.5
Gm4922	40897	3081	2163	1276
Gm4942	25405	26695	28634	27462
Gm4944	22253	23078	19097	22796
Gm4955	22098	25904	31652.5	14467
Gm496	30642	32241	34384	41020
Gm4968	4490	3298	8080	6448
Gm498	36816	35885	11502	7775
Gm4980	14172	14462	18607	16618
Gm4983	9827.5	8641.5	9488.5	6698
Gm4989	8772	10850	12089	14231
Gm4994	38485	7742	26913	28914
Gm4995	5747	12489	12355	12135
Gm500	8004	158	548	5206

Gm501	27033	28301	36273	32432
Gm5039	33674	4016	39709	1241
Gm5065	3304	4399	10773	6158
Gm5069	24987.33333	22394	19626	27461.66667
Gm5073	13417	35120	8192	35452
Gm5077	40829	5730	9046	7299
Gm5082	9477	5953	40521	5985
Gm5084	7403	14292	18994	13474
Gm5085	18460	20631	18200	38494
Gm5086	38926	14794	34210	4219
Gm5087	30665.5	37460	19191.5	20805.5
Gm5089	31186	32561	34075	32418
Gm5094	29507	31289	40013	29947
Gm5101	32858	34288	35746	33876
Gm5105	28643	33034	31799	31102
Gm5111	32725	34513	36529	35797
Gm5114	24894	26100	27867	26925
Gm5115	17454	15093	9721	5244
Gm5124	14418	15318	16906	18819
Gm5127	38417	6072	14462	12612
Gm5128	32237	34038	35180	33375
Gm5132	14195	11748	20599	25523
Gm5134	7904	37867	396	7023
Gm5138	32989	24812	23388	30287
Gm5142	34990	30645	4931	28700
Gm5148	29098	30388	32384	30596
Gm5150	25222	20899	22669	20481
Gm5154	5739	13377	10446	9780
Gm5168	28447	35893	37761	40393
Gm5169	15626.5	27616	20484	19469
Gm5222	26599	28052	29384	28300
Gm5226	8151	6032	14020	11828
Gm527	3736	7319	6228	8717
Gm5292	21457	21540	22826	22477
Gm5296	30399	18966	26264	23396
Gm5331	10347	8670	7957	4934
Gm5334	32392	38174	2244	2556
Gm534	24437	25509	27778	26589
Gm5356	22203	29521	24518	35281

Gm5373	26193	30862	34639	36103
Gm5376	34620	35613	24929	29338
Gm5396	15772	16944	17576	15862
Gm5420	28494	39617	36805	30123
Gm5431	9817	5131	4662	6287
Gm5433	40333	4243	4675	7645
Gm5444	32009	33294	36216	34230
Gm5447	32741	34443	35438	33644
Gm5478	34897	40570	6233	8262
Gm5483	27557	28928	30372	29094
Gm5513	25007	29853	34552	35356
Gm5547	6436	11934	7252	9124
Gm5550	21867	18870	22563	22120
Gm5591	24782	25883	28134	26943
Gm5594	25892	23668	38819	37084
Gm5595	28100.33333	31953.33333	28036.33333	32167.33333
Gm5622	30216	38341	1145	318
Gm5624	13390	4004.5	33943	19784
Gm5662	16833.5	20613	23929.5	27020.5
Gm5665	27183	26793	11536	27678
Gm568	16270	9381	19051	9265
Gm5682	15324	15293	12552	12464
Gm5699	20387	37413	39440	41081
Gm5753	9134	15214	10810	16718
Gm5766	1713	7758	2913	777
Gm5778	4221	5670	8225	10273
Gm5800	25563	36379	29079	27815
Gm5828	17423	16661	14869	17772
Gm5865	15476	18253	16286	18055
Gm591	26141	27671	29800	1611
Gm5910	1851	584	40559	928
Gm5914	28277	31944	34005	32785
Gm5919	31897	33740	2007	9
Gm5950	34649	33381.5	22533.5	36334
Gm5962	39328	37104	5978	2216
Gm597	39579	38723	4458	39711
Gm5978	4962	7120	9297	7972
Gm6003	7962	13072	14543	16726
Gm6020	7422	10451	6528	12504

Gm608	21978	20169.5	19865.5	20005.5
Gm609	32440	33721	37174	35177
Gm621	37195	26551	2459	35755
Gm6213	27317	29061	29578	38111
Gm6260	29620	31255	40603	30302
Gm6377	35969	20857	32613	24351
Gm6484	22954	25678	24099	24115
Gm6498	31301	28822	26133	36864
Gm6583	7684	12527	17652	11277
Gm6592	11901	31283	39415	32190
Gm6639	25334	28027	28434	27289
Gm671	29129	30619	33273	31569
Gm6710	21691.33333	25471.33333	17785.66667	25291.66667
Gm6787	31334	38526	6004	5316
Gm6792	2945	8839	6136	17809
Gm6793	11551	10580	9809	11307
Gm684	33889	35271	4812	1886
Gm6994	6030	3098	17292	38329
Gm7056	34697	8735	1273	9897
Gm7086	11851	18606	22123	23657
Gm7247	23193	24284	26125	25040
Gm7265	2237	27125	9384	38249
Gm732	28262	30199.5	33816	30905
Gm7461	31413	32300	1385	2405
Gm8108	10505	24002	16506	19325
Gm821	32076	33950	35299	33904
Gm839	14498	20835	18787	19070
Gm8995	13513	13211	14052	24472
Gm9	38603	41056	4927	4052
Gm9387	9112	11971	15063	13694
Gm94	15874	38615	29050	38478
Gm97	30454	10964	11288	6117
Gm9725	15431	31752	25802.5	23400
Gm9758	30155	20421.5	18826.5	4873
Gm9796	3386	38176	22	39168
Gm9801	9049	2846	13144	13324
Gm9805	26573	28351	31109	27375
Gm9871	10678	12760	12069	441
Gm9890	29000	30378	32724	31167

Gm9927	27656	29019	31319	29823
Gm993	22057	27134	29508	34326
Gm9946	28131	29505	31932	30438
Gm9975	38382	34666	28589	28418
Gmcl1	19223	21873	22272	28104
Gmcl1l	24761	25906	30121	26946
Gmds	26002	24396	21122	21995
Gmeb1	15551.5	13200	13686	13002
Gmeb2	29234	23706.5	24224.5	23112
Gmfb	4026	27558	5735	9253
Gmfg	16947	16410	5707.5	14770
Gmip	15641	11163	12997	6627
Gml	34273	125	31641	112
Gml2	22777	23242.5	13803	29318
Gmnn	7328	8242	12167	5109
Gmppa	19489	17872	17255	18949
Gmppb	16227	19630	18377	21768
Gmpr	4304	1319	247	34348
Gmpr2	17952	14740	13579	16965
Gmps	15855.66667	18284.66667	15033.33333	16915.33333
Gna11	19175.5	38319	20864	19470.5
Gna12	13063	6229	8423	1469
Gna13	22509.33333	21048.33333	19773.66667	23727
Gna14	26670	30077	38344	39046
Gna15	36455	37621	37712	37483
Gnai1	24788.5	23964.5	16357.75	24734.75
Gnai2	29694.5	26734.5	22520.5	17338.5
Gnai3	18030	16202	19399	19849
Gnal	20958	28008.6	16630.4	23231.4
Gnao1	13116.66667	21916.66667	6765.333333	25221.33333
Gnaq	15045.75	9470.75	11312.5	10280.25
Gnas	14003.11111	16013.22222	12329.77778	16455.77778
Gnat1	22584.5	24157.5	24810.5	28625
Gnat2	19100	20037	28999	28520
Gnaz	13962.5	9538	7056.5	9417
Gnb1	19552	21491.33333	26149.33333	26262.33333
Gnb1l	14883	15773	14663	13918
Gnb2	22046	20523	20467	18040
Gnb2l1	10964.33333	11811.33333	12964.33333	13842.66667

Gnb3	4519	40243	12410	9366
Gnb4	17135	20440	11592.33333	11179.33333
Gnb5	28393	35981	34599	34328
Gne	13551.33333	10104.66667	9763.333333	20195
Gng10	5253	6835	7085	3511
Gng11	37228	36283	1069	34858
Gng12	3182.5	6300.5	19778	2555
Gng13	36538	27383	5700	15245
Gng2	27998	25003.5	29815.5	20835
Gng3	12534	15808	31895	17241
Gng4	5466	6126	3576	22608
Gng5	7730.333333	9297	9179	9542.666667
Gng7	14778.5	14518.5	14510.5	18195
Gng8	12126	23878	18194	27254
Gngt1	25076	26464	27630	3597
Gngt2	18709	2963	23252	2734
Gnl1	18292	18308.5	18767.5	16649.5
Gnl2	21426	23593	24562	27876
Gnl3	16061	18735	18439	21266
Gnl3l	5805.5	8730.5	7480	9351
Gnmt	10939	13030	17598	17964
Gnpat	40586	38317	37998	37356
Gnpda1	4147	37602	32332	27038
Gnpda2	3767	4229	783	40998
Gnpnat1	19525.5	16180.5	18749	19951.5
Gnptab	20407.5	21258.25	19602	22249.5
Gnptg	39054	27576.5	32324	28456
Gnrh1	40472	39689	605	2760
Gnrhr	39282	39580	7227	7146
Gns	29106	23946.66667	20944	23152.33333
Golga1	21155.66667	17005.66667	30875	26932.33333
Golga2	13467	11458	16731	16049
Golga3	15403.25	13049.25	16313	13978.5
Golga4	20653.66667	19338.66667	21413	18173
Golga5	32971.33333	24030	21858.66667	24817.33333
Golga7	39120	39466	40340	39440
Golga7b	18872	21581	24772	22347
Golgb1	16364.71429	17842.14286	21838.28571	18666.85714
Golim4	11023.75	17212.25	15530.75	18944.5

Golm1	24741	22418	22833	20720
Golph3	20284.33333	10542	23406.33333	12249.33333
Golph3l	27381.75	30241.75	30191	31087.25
Golt1a	4668	4982.5	7632.5	7437
Golt1b	18768	25675.5	21724	22770
Gopc	14233	21218.4	22712.4	25265.8
Gorab	21120.5	21228.5	29574	26409
Gorasp1	10358	6177	4034	4694
Gorasp2	13339.33333	14195	26149.33333	16089.33333
Gosr1	30350	14712	13826.5	16059.5
Gosr2	28503	17319	13711	18610.33333
Got1	2495	1753	2781	40880
Got1l1	1526	8860	11101	3564
Got2	4245	39884	4769	2597
Gp1ba	36596	27049	29918	37170
Gp1bb	26107	23764	25186	24818
Gp2	6457	39916	37602	30009
Gp5	14023	13112	17842	20149
Gp6	9232	21897	13846	23837
Gp9	36802	31810	10172	11906
Gpa33	22517.5	21210.5	9642.5	7420.5
Gpaa1	38119	37598	25388	27816
Gpalpp1	20668	23721	22291	25865
Gpam	23154	22012	20934	23448.5
Gpank1	29686	30145	31519	35553
Gpat2	33694	29738	20923	24134
Gpat4	5612.333333	6667.333333	5525.333333	8992
Gpatch1	24449.5	24931	22006	22409
Gpatch11	17115	16693.5	28040.25	18704.5
Gpatch2	11951.33333	25187.33333	12140	13174
Gpatch2l	21573.5	14540.5	14400.5	16484
Gpatch3	19268	15691	19465.5	20297
Gpatch4	22653	25055.5	26149.5	25485.5
Gpatch8	21112.25	22078.5	19142	15153.5
Gpbar1	15273	12417	22945	24617
Gpbp1	18983	20232.5	20336.5	20876
Gpbp1l1	19796.5	20782	21949	19630.5
Gpc1	8372	17922	8136	22179
Gpc2	12245	14466	18310	21623

Gpc3	28126	29766	30374	29138
Gpc4	20375	17616	25163	8908
Gpc5	27993.66667	29376	32322.33333	34687.66667
Gpc6	22145.75	23658	24483.5	16215
Gpcpd1	19505.5	13575.5	19036	13953.75
Gpd1	23506	17091.5	26578.5	19773.5
Gpd1l	15961.5	18376.5	17208	15568
Gpd2	8010.5	20463.5	10392.5	22895.5
Gper1	7072	10622	15204	15669
Gpha2	33387	32665	26033	29331
Gphb5	30839	32438	34050	32380
Gphn	18295.5	19004	18372.75	18608
GPHN	11186	11493	7109	40465
Gpi1	4181	25154	7220.5	9503.5
Gpihbp1	19360	22355	26335	28624
Gpkow	22519.5	19552	20979	19198
Gpld1	35835	5245	38163	7175
Gpm6a	21491	26616.5	30217	29042.5
Gpm6b	30864	30538.5	17415.25	20862.5
Gpn1	20670	21860	20971	21044
Gpn2	959	5116	249	3403
Gpn3	37463	967	39831	40491
Gpnmb	21915	12650.5	18433.5	16609.5
Gpr1	23798	27495	29914	33403
Gpr101	34893	27203	41092	285
Gpr107	23765	23762.5	19346.25	23419
Gpr108	33747	31908	34341	38983
Gpr112	26457	27687	31217	29576
Gpr119	4402	7100	7276	7320
Gpr12	10721.5	26093	25873	25388.5
Gpr132	5777	30782	36218	24146
Gpr135	34424	30335	36883	37287
Gpr137	31693.5	14995	27038	30851.5
Gpr137b	36950	30771	38052	36764
Gpr137c	22739	20837	11003.5	9082.5
Gpr139	27954	8407	10856	11481
Gpr141	9053	24552	2331	20625
Gpr142	16469	16411	17105	19447
Gpr143	17889	19297	34882	33987

Gpr146	37589	27359	31730	22967
Gpr149	22544.66667	22913.66667	33588	21165.33333
Gpr15	2255	39559	32041	40373
Gpr150	9537.5	28243.5	17423	14604
Gpr151	27623	34811	7325	36548
Gpr152	24590.5	25900	11026	10573.5
Gpr153	19227	20071	23811	24747
Gpr155	16232.25	21136.25	25492	21799.75
Gpr156	29458.5	13131.5	21961	21723.5
Gpr157	4965	358	2494	39257
Gpr158	32541.33333	21438.66667	25160.66667	13619.66667
Gpr160	15383	5669	11924	2081
Gpr161	16040	17503	21640	21867
Gpr162	17978	38451	17267	24996
Gpr165	1010	10014	20780	19777
Gpr17	11135	17055	20284.5	20971
Gpr171	9424	8055	9685	11030
Gpr173	20105.25	15138.5	18895.25	25913
Gpr174	16899.5	20986.5	25054	30335.5
Gpr176	17878	23656	13296	22083
Gpr179	11124	17316.5	9881.5	8880
Gpr18	7909	15104	6330	15896
Gpr180	13549	15042	9561	8235
Gpr182	23358	17623	17721.5	17748
Gpr183	36812	19585	34866	17854
Gpr19	3088	414	5761	1018
Gpr20	6161	39162	1382	14174
Gpr21	12519	16857	19294	17983
Gpr22	25514.33333	33002	32260.66667	34345
Gpr26	21064	30006	27459	34209
Gpr27	14229	15838	23171	27376
Gpr3	27437	28918	30218	29019
Gpr31b	15057	22045	24670	26005
Gpr33	9405	11667	18363	20292
Gpr34	27029	27864	2352	30130
Gpr35	12474.5	22835.5	11359	21499
Gpr37	10303.66667	13670.66667	16670	17333.33333
Gpr37l1	3718.5	5570	9824	9120.5
Gpr39	14122	15515	21452	21917.5

Gpr4	17813.5	21059	26497	33662
Gpr45	8230	5373	14159	10093
Gpr50	13620	13763	17460	16984
Gpr55	9656	15368	24230	23025
Gpr6	2846	5143	8024	7853
Gpr61	26459	27497	30518	29110
Gpr62	11616	11814.5	10383.5	30544
Gpr63	35033	3309	2853	7914
Gpr65	26832	20894	22806	17362
Gpr68	30535	12818	27131	11371
Gpr75	38587	515	2760	2847
Gpr82	13712.5	20734	22468.5	21171.5
Gpr83	32518	15789.5	32495.5	15993.5
Gpr84	37081	6708	25653	38514
Gpr85	2690	9444	40225	7031
Gpr87	7773	6749	13182	6641
Gpr88	38052	41109	39255	32777
Gpr89	15786	16146	11650	15329
Gprasp1	15999	35642.25	18578.75	18029
Gprasp2	2989	5366	11108	9068
Gprc5a	39275.5	1882.5	3579.5	3564.5
Gprc5b	13008	972	18120	9661
Gprc5c	17278	9656	18574.5	10673
Gprc5d	9806	11650	14407	15450
Gprc6a	37637	39316	1737	4644
Gprin1	16325	22114	36482	36178
Gprin3	28808.5	33153	25427	23186
Gps1	10938	9363	8219	8914
Gps2	14991	12570	14233.5	13088
Gpsm1	13141	12643	13999	14022
Gpsm2	14706.5	17769.5	13460	16334
Gpsm3	3178	36814	736	35736
Gpt	8223	8330	8664	6065
Gpt2	3399	5882	7024	5646
Gpx1	5372.5	7377	11382.5	9076
Gpx2	8661	5169	8828	7891
Gpx3	5459	20733	23755	8113
Gpx4	32550	38582	32507	38665
Gpx5	4656	9379	13563	13472

Gpx6	1808	13368	34844	40928
Gpx7	18001	20419	24467	24886
Gpx8	4356	7779	9862	13764
Gramd1a	18267	18341	17596	19240
Gramd1b	20118.6	21863.2	20760.6	25913.8
Gramd1c	21295	13241	26247	18030
Gramd3	23239.66667	11996	13902.33333	12776
Gramd4	25908	11844	18542.5	9456.5
Grap	37873	37514	950	40652
Grap2	27029.75	22976	13095.75	20635
Grasp	9917	15156	13002	16114.5
Grb10	11547	28184.66667	21310.33333	28145.66667
Grb14	29483.5	30751	33236	31465.5
Grb2	27076.5	18770.5	24006.5	18355
Grb7	21071	19550	41072	33949
Grcc10	36037	38522	35107	32723
Greb1	31525	33219	34660	34405
Greb1l	10766	11525	19623.5	19770
Grem1	32816	3548	5647	9177
Grem2	13758	20747	20819	25817
Grhl1	8594	34542	19634.5	19314.5
Grhl2	23977.33333	30046	11603.66667	7359.666667
Grhpr	14535	16833	18459	23330
Gria1	26916	33348	29918	30460
Gria2	31462.4	27204.6	26857.8	26863.2
Gria3	32590.33333	10261.33333	20778	14591.66667
Gria4	22368	27328	28076.5	24842.875
Grid1	12529	6785	9867	10568
Grid2	34450	23254.28571	32287.42857	21950
Grid2ip	7217	13164	18925	12950
Grifin	1102	2654	35922	38972
Grik1	31244.5	24177.5	32066.25	18412.25
Grik2	20403.75	31508.25	17972.75	32276.25
Grik3	29422	31008	31749	14566.5
Grik4	26068	9885	14893	109
Grik5	36912	29409	461	37911
Grin1	22906	22824	24900	24866
Grin2a	27554	29024	30279	29321
Grin2b	15726.33333	21820	15052.66667	14524.66667

Grin2c	4274	3842	8719	9836
Grin2d	15753	8825	9127	13871
Grin3a	13493	15002	21560	25479
Grin3b	15167	17715	15426	23655
Grina	7415	4869	37831	3353
Grip1	21128.6	28837.4	22018	26476
Grip2	1409	1382	7255	4605
Gripap1	24852.25	32230.75	23849.5	30970.25
Grk1	32040	33967	39015	39110
Grk4	19044.5	16623.5	16153	25006
Grk5	17857	12658.66667	20330.33333	16633
Grk6	24995.33333	24426	25303.33333	23635.33333
Grm1	18597.5	15142.5	19084	18636.5
Grm2	11752.66667	13840.66667	28670	17252.66667
Grm3	26510	35952.5	15926.5	32621
Grm4	29321	28213	33909	30603
Grm5	15831.6	18209.4	21488.4	23500
Grm6	37328	37887	40179	39059
Grm7	25466.2	20932.8	27321.8	18603.6
Grm8	26383.5	27842	21198	17865
Grn	7610	28531.5	32390	29803.5
Grp	26131	27708	28570	29738
Grpel1	21425	11431	12077.66667	15314.66667
Grpel2	20473	22736	20489	24485
Grpr	26339	2302	30033	3172
Grrp1	2551	4408	3867	6250
Grsf1	34984	29026	29590	28391
Grtp1	37842	36580	35667	24285
Grwd1	6729.5	6736	7763.5	8495.5
Gsap	15854.33333	16436	29997	16994
Gsc	31511	33198	34304	251
Gsc2	5291	10083	13970	15031
Gsdma	16662.5	18540	22984.5	20969
Gsdma2	14675	16557	22681	22262
Gsdmc	4851	6439	12229	12546
Gsdmc2	40252	35374	6440	590
Gsdmc3	14204	16937	22478	22874
Gsdmcl1	26486	28691	30411	31572
Gsdmcl-ps	30586	32039	32132	30682

Gsdmd	38670	562	28494	37671
Gse1	4798.5	2446.5	1516	4060
Gsg1	8850.5	11836	15606	15086.5
Gsg1l	8951	4662	11373	14121
Gsg2	27750	20713	21538	20936
Gsk3b	19582	19509.25	19275.25	18891.75
Gskip	16045	19585.33333	14149.33333	13119.33333
Gsn	37960	23998	36866	23706
Gspt1	27917	26630.66667	33411.33333	28358.66667
Gspt2	21499.5	20126.5	20829.5	22113.5
Gsr	23781	6294	19138.66667	17705.33333
Gss	16207	34544	13491	38116
Gsta1	14881	5860	32534	20284
Gsta2	29896	24454	12053	6248
Gsta3	9503	6020	21022	12435.5
Gsta4	9369	14768	13518	20063
Gstcd	24135.66667	20248	27762.33333	23052.33333
Gstk1	14936	13760	17415	13458
Gstm1	10553	8752.5	8070	11851
Gstm2	32811	12652	26226	12015
Gstm3	2393	40146	192	5836
Gstm4	39528	40930	25687	32698
Gstm5	35140	7163	37391	39778
Gstm6	36832	568	40863	2017
Gstm7	23551	26059	27359.5	11302
Gsto1	18624	21565	18744	20582
Gsto2	30358	30616	12801.5	33516.5
Gstp1	24291	31321	21786.5	32151.5
Gstt1	33648	37939	5529	7143
Gstt2	1351	301	1797	38212
Gstt3	6723	35691	4119	32345
Gstt4	27469	32515	32817	15241
Gstz1	10576	13350	11330	11356
Gsx1	18453	19099	23236	23665
Gsx2	2385	5285	18686	17812
Gtdc1	18730	26978	16030.5	11059.25
Gtf2a1	7006.666667	5838.666667	5818.333333	7163
Gtf2a1l	28433	38772	35062	38480
Gtf2a2	13683	15159.33333	15541	16470.66667

Gtf2b	17992.5	11677	11374.5	13178.5
Gtf2e1	22246	25161.5	26137.5	26781
Gtf2e2	4117	9443	6319	10400
Gtf2f1	19004	19203	21460	23658
Gtf2f2	16611	20016	15025	17720.5
Gtf2h1	26019	25114	24466	24430
Gtf2h2	11961	14694.5	14921.5	16414.5
Gtf2h3	13007	16345	12882	14851
Gtf2h4	19428	19415	18641	18432
Gtf2h5	18250.5	19834.5	17104	34384
Gtf2i	15950.8	20890.2	22826.6	22170
Gtf2ird1	25021.5	27310.25	24749	25034.75
Gtf2ird2	22217.33333	18751.66667	14764.33333	10945.66667
Gtf3a	32192	29303	27489	26793
Gtf3c1	28680	23571	31865	28364
Gtf3c2	15727.5	11935.5	10321	10069.5
Gtf3c3	2814	5360	963	1914
Gtf3c4	16997.5	15931	17375	16197.5
Gtf3c5	21525	22479	21114	25014
Gtf3c6	17133	19548.5	18300.5	18486.5
Gtpbp1	19925	20399	19235.5	19602.5
Gtpbp10	8822.75	11710.75	19528.75	12092.5
Gtpbp2	14879	18302	17507.5	19260
Gtpbp3	13901	17925.33333	13606.66667	14198.66667
Gtpbp4	24402	20106	16625.5	19461.5
Gtpbp6	6047	7787	5637	9191
Gtpbp8	28112.66667	23926	25957	16576
Gt(ROSA)26Sor	22711	25713	21189	23916
Gtse1	38453	29420	37431	34204
Gtsf1	29739.5	17488.5	16430	23931.5
Gtsf1l	24197	35442	30572	36082
Guca1a	13087	17188	10986	11420
Guca1b	10319	12297	12386	11896
Guca2a	1833	5145	12341	12148
Guca2b	23910	25843	25091	23222
Gucd1	24681.5	17612	17027	21208.25
Gucy1a2	32925	34264	4445	8311
Gucy1a3	17948.75	25394.25	32151.5	23503
Gucy1b2	970	7857	10315	11686

Gucy1b3	18697.33333	18642	33969.33333	35435.33333
Gucy2c	3057	5849	9875	10039
Gucy2e	19855	17442	6644	20986
Gucy2f	39764	1293	39521	39599
Guf1	26549.66667	22683.66667	22164.66667	23043.33333
Guk1	24172	33065	36490	35790
Gulo	20251.5	20108.5	23417	2449
Gulp1	16539.5	17290	19644	18570.75
Gusb	36618	33271.5	38630.5	34805
Gvin1	15076.25	18485.25	23043.5	24987.5
Gxylt1	16826	13380	16488	18586
Gxylt2	32010.5	34915	18272.5	18816
Gyg	1854	29608	40960	26086
Gykl1	30243	38808	37919	3889
Gyltl1b	18908.5	17501	23024	23458.5
Gypa	32590	33643	36205.5	35414.5
Gypc	20170	17389	18549	14899
Gys1	16046	19374	20308	25565
Gys2	23994	25182	26746	25637
Gzf1	16726	19380	15866.5	19129
Gzma	28906	30332	31989	30276
Gzmb	31855	30384	32714	31159
Gzmc	8249	1851	16017	7707
Gzmd	15209.5	18192	20319.5	22743
Gzme	23843	34785	6372	9412
Gzmf	30478	32476	32812	31271
Gzmg	24394	25679	38324	25722
Gzmk	11591	11577	34095	27986
Gzmm	7202	11028	9706	12007
Gzmn	25132	26378	28045	27093
H13	10466.66667	16563	20598.66667	17989.66667
H19	37357.5	20964.5	22513	23294
H1f0	33486	34413.66667	3189	26533
H1fnt	20877	17953	21162	20729
H1foo	3441	8803	4462	11571
H1fx	12723.5	25658.5	23526.5	28909.5
H28	21845	24839.5	16911	16725
H2-Aa	19297.5	35581.5	18868	34364.5
H2-Ab1	20390.5	20581.75	21957.25	14456.5

H2afb1	645	991	3493	2710
H2afj	21538.5	21209.5	24078	24000
H2afv	10196	10313	13867	7974
H2afx	23533	28407	20529	25113
H2afy	13913	13334	25904.66667	13434.66667
H2afy2	21692.5	18481.5	21201	19962
H2afy3	36890	26400	32303	24620
H2afz	31219	28452	30898.5	28441.5
H2bfm	34839	27143	30297	27078
H2-Bl	31258	12573.66667	34946	20094.66667
H2-D1	12426.5	18527.5	18037.5	22867.5
H2-D4	40689	2610	38198	3683
H2-DMa	12589	12066	10056	8998
H2-DMb1	4674	9589	1458	4237
H2-DMb2	16629	20283.5	24071	27701.5
H2-Ea-ps	32991	31091	32988	31469
H2-Eb1	30002	2065	23824	38365
H2-Eb2	32010	35588.5	18528	18712
H2-K1	16329.25	12065	19210.25	17982.5
H2-Ke6	19770	20744	16480	15943
H2-M1	29646	6236	3473	13929
H2-M10.1	29863	31436	37873	31334
H2-M10.2	38325	3893	405	5672
H2-M10.3	29484	31116	32938	4904
H2-M10.4	31026	28673	29652	38158
H2-M10.5	2996	1579	11253	17648
H2-M10.6	25649	27148	30678	26942
H2-M11	30376	4481	27647	1167
H2-M2	21189	1959	24053	6147
H2-M3	37223	24794.5	21591	26548
H2-M9	33790	32656	38759	9703
H2-Oa	18274	25260	16825	20574.5
H2-Ob	24353.5	16368	28392.5	30019.5
H2-Q1	35937.66667	12446.66667	36841.66667	13711.33333
H2-Q10	25558.6	17082	19640.4	13090.8
H2-Q2	23972.5	11295	26842.5	15872.5
H2-Q5	7777	12122	7817	14421
H2-Q7	6002	9071	6940	12053
H2-Q8	10039	13556	23450	21381

H2-T10	18036	23556	18222	25367
H2-T22	16875.5	25393.5	13790.5	25007
H2-T23	2677	5272	5212	10794
H2-T24	15065	22187.5	12275.5	20711.5
H2-T3	36791	35124	3415	10370
H3116H03	35256	33105	31124	36339
H3118C03	7940	39766	39626	34286
H3f3a	34512.66667	26799.66667	26349.33333	2901
H3f3b	8685.666667	33646	14136.66667	12590.33333
H60a	14885	11326	14908	21027
H60c	37714	36152	27376	33848
H6pd	31075	31445	29051	31759
Haao	37403	39166	10244	9719
Habp2	29802	31330	32704	31609
Habp4	12543.5	12409	11188.5	28162.5
Hacd1	21033.5	21494.5	22398.5	20015.5
Hacd2	13511	15509.5	13759.5	12790.5
Hacd3	19405	17666	16875	14510
Hacd4	13256.5	3108	3797	19954
Hace1	26405.66667	14275.66667	16754.66667	25068.33333
Hacl1	31607	20027	34738	36649
Hadh	26979	22842	17938	14451.5
Hadha	11277.33333	18008.33333	24862.66667	23147.66667
Hadhb	9455	37375	6128	38724
Hagh	19390	23987	22355	23387
Haghl	35756	37206	30788	32924
Hal	32479	21687	21809	16392
Hamp	33034	6948	4418	1868
Hamp2	39169	4686	3388	3382
Hand1	30470	22470	34670	10343
Hand2	6495	6567	7876	11141.5
Hao1	10602	11979	19246	17976
Hao2	36447	32341	37806	38297
Hap1	35758	36172	33222	35310
Hapln1	11716	15106.33333	12991.33333	12687.33333
Hapln2	28	4397	28735	39514
Hapln3	21287	23986	30647	33437
Hapln4	17000	18300	21298	23690
Harbi1	18521.5	19707	21775	23716.5

Hars	12881.5	34564.5	33742.5	34592.5
Hars2	8583	8655.5	7369	27145.5
Has1	35691.5	32580	19349.5	22339
Has2	10635	16741.5	19879	21452.5
Has3	27073	36030	7097	4115
Hat1	23969	27258.5	24911.5	25378
Haus1	28305	32951	32086	25155
Haus2	38493	3800	38806	39998
Haus3	24666	17981	21947	18865
Haus4	20658	16592	18713	13434
Haus5	22639	15486.5	18269.5	13042.5
Haus6	16126.5	14815	32149	29529.5
Haus7	8334	9070	6562	7393
Haus8	6032	4297	8482	8656
Havcr1	18230	21942	25063.5	2615
Havcr2	23397	8452.5	15271	17493.5
Hax1	8250.333333	10217.33333	12060.66667	13928
Hba-a1	14737	15737	24385.5	24336.5
Hba-x	14661	17134	7498	13891
Hbb-bh1	18167.5	17828.5	20897	21559
Hbb-bt	14556.25	9614.25	14004.25	15140.5
Hbb-y	1905	9474	10028	10723
Hbegf	38657	35607	32528	39610
Hbp1	4316.5	6132.5	3784	7332
Hbq1a	40433	36065	4653	3410
Hbq1b	35808	2140	5711	3893
Hbs1l	8533.75	9527.5	8872	10585
Hc	29060	34493	40212	37119
Hcar1	28506	30432	30837	37248
Hcar2	38443	14235	31879	15687
Hccs	21251.5	23130.5	6848.5	13026
Hcfc1	17718.33333	11693.66667	11332.33333	5294.333333
Hcfc1r1	37630	29768	31406	29905
Hcfc2	12384	21109.5	21575	20537
Hck	11056	22267	10284	18236
Hcls1	21810	31826	25402	41030
Hcn1	25236.4	25896.8	27236.4	20151.4
Hcn2	37322	38271	3267	3671
Hcn3	39835.5	22438	1291.5	21041

Hcn4	6974	11002.5	16155	18304.5
Hcrt	14275	18603	19924	21686
Hcrtr1	1850	39639	4279	716
Hcrtr2	18944.66667	25455.66667	25382.66667	19712.66667
Hcst	28244	27816	34925	26515
Hdac1	21632	25220	24551	25059
Hdac10	27630.5	21413	21814	18888
Hdac11	15078	23385	9143	9488
Hdac2	13114.5	33047	30615.5	20970
Hdac3	16441	17421	16163	15441
Hdac4	38372	36830	36368	34003
Hdac5	40496	36589	39843	35116
Hdac6	414	37980	38739	36011
Hdac7	14659	16097.5	13764	13709.5
Hdac8	12462.75	16007.5	16297.75	18680.5
Hdac9	33646.25	26024	23734.25	21525.5
Hdc	23143	157	22199	6054
Hddc2	20682	26821	21310	21661
Hddc3	21519	20447	21149	22471
Hdgf	23088	27265.5	19843	27254
Hdgfl1	34357	28604	2746	40736
Hdgfrp2	29787	27040	31138	32854
Hdgfrp3	22261.66667	12583.33333	24615	24069.33333
Hdhd1a	35729	2806	31523	4538
Hdhd2	24901	26696	22564	22523
Hdhd3	12812	16724	16701	18274
Hdlbp	27676	23844.66667	21635.66667	6746.666667
HDX	26365	27920	28796	27627
Heatr1	15512.33333	11160.66667	11625	12621
Heatr3	22789	33281	24303	30491
Heatr5a	23602.85714	14801.85714	23893.42857	22486.85714
Heatr5b	20646.5	14840	24336.5	32773.25
Heatr6	14754	12877	27186	22261
Heatr9	28526	23858	36793	31233
Hebp1	8870	19009	12919	14371
Hebp2	12870	13936	9277	6609
Heca	11845.5	8214.5	10535.5	10214.5
Hectd1	29118.2	19214.6	19653	18209.8
Hectd2	13355	23839.33333	12737.66667	12724.33333

Hectd3	31385	29797	29048.5	26994
Hecw1	26120.6	27132.8	28735.2	33180.4
Hecw2	18516.5	35687.5	7333.5	3379
Heg1	13251.75	18195.5	23374.5	16508.25
Helb	13476.66667	5043.666667	7222	15122.33333
Hells	3956	7650	5578	8373
Helq	19876.33333	13748.66667	26785.33333	25815.66667
Helt	36927	34575	6249	5732
Helz	10000.66667	16548.33333	6764.666667	28748.66667
Helz2	25989	10587.5	27154.5	13456
Hemgn	25576.33333	26768.66667	25921	34644.33333
Hemk1	17257	20318	21362	26257
Henmt1	29117.5	26769.5	30433	28915
Hepacam	19175	19548.5	7391.5	7371.5
Hepacam2	35216	37914	4647	1717
Heph	15921.5	22965	19257	28338.5
Herc1	21482.66667	27354	17700.33333	22047.5
Herc2	8362	7129	5956	4186
Herc3	9852.5	10877	12599	13151
Herc4	19102.25	16000.25	10519	17047.5
Herc6	25630.33333	20184.66667	28407	17114.33333
Herpud1	27885	36031	21668	27568
Herpud2	19019	14200.5	16596.5	14038
Hes1	9265	11305	13774	18321
Hes2	14490.5	17168.5	18929	19821
Hes3	12571	16145	22051	23077
Hes5	16032	21254	21064	20871
Hes6	23330	24696.5	23848	23977
Hes7	14939	14843	19457	14152
Hesx1	36903	32675	33705	32135
Hexa	6366	41075	4659	40617
Hexb	30324	20748	28648	25639
Hexdc	19716	24244	17703	22487
Hexim1	20080.5	24006.5	20670.5	24670.5
Hexim2	17343.5	24289.5	20145.5	25441.5
Hey1	3745	34021	1935	25049
Hey2	27439.5	32894.5	35499	29512.5
Heyl	21984.5	38168	3383.5	4755.5
Hfe	21396	10998	15240	22745.5

Hfe2	27998	29348	40985	443
Hfm1	20832	24472	40806	39754
Hgd	37374	37716	40068	38924
Hgf	18252.8	17830.8	18274.4	24237.4
Hgfac	36606	37171	3275	2336
Hgh1	34881	35230	28826	33279
Hgs	15956	15494	11769	12589
Hgsnat	15047	14209	21408	22449
Hhat	33736	33288	12384.5	12691
Hhatl	5859	7165	12073	11908
Hhex	31543	24624	29142.5	18306.5
Hhip	29682.5	31220.5	33018	31372
Hhipl1	17986	18632	24217	22526
Hhipl2	21676	22571	27402	33416
Hiat1	26395.5	27206	26568.5	27769
Hiatl1	37454	40981	38687	39723
Hibadh	21284.5	14826.5	26631.5	21105
Hibch	19913	19446	23297	18846
Hic1	25866	36196	622	6352
Hic2	32990.5	26562.5	29662.5	25469
Hid1	7653	1238	31818	7289
Hif1a	19720	36781	21328	38010
Hif1an	3856	4715.333333	3788.666667	17227
Hif3a	15742	19019.33333	18959	18897.33333
Higd1a	21753	9494	22449	28753.5
Higd1b	40341	33911	11393	10310
Higd1c	28582	30122	32922	31182
Higd2a	40877	36837	33803	32858
Hilpda	22215.5	18228.5	8757	5806
Hils1	35854.5	21250	22229.5	18289
Hinfp	32978.5	36513	33719	38070.5
Hint1	4863	9229	10475	11059
Hint2	34785	34377	40156	38759
Hint3	29009.5	34911	34045	33483.5
Hip1	13188.5	16181.75	18778.25	11670.75
Hip1r	40090	305	28537	40667
Hipk1	12954	19640	10518.75	13541.75
Hipk2	20685	13498.5	17889.25	16065.25
Hipk3	20364.66667	15222.66667	15133.66667	15352

Hipk4	2044	3853	7558	14945
Hira	18308.25	22692	17955.5	22759.75
Hirip3	11018	12733	18019	17902
Hist1h1a	20422	26608	34159	35741
Hist1h1b	22389	18532	15880	16346
Hist1h1c	13623	29999	13486	37966
Hist1h1d	23316	30849.66667	22420.66667	22030.33333
Hist1h1e	27794	15559	17899	17182
Hist1h1t	8166	9097	13355	4266
Hist1h2aa	20887	20865	24374	23688
Hist1h2ab	23550	22402	29489	27041
Hist1h2ac	34522	31563	32374	30360
Hist1h2ae	30207	28746	33020	31270
Hist1h2af	10695.5	11518	15386.5	12970.5
Hist1h2ai	23535	29452	27083	27028
Hist1h2ak	32583	36738	36169	30546
Hist1h2ao	990	39233	2659	40606
Hist1h2ba	18123	15258	24094	13804
Hist1h2bc	28415.5	23442	26666.5	25129
Hist1h2be	29313.25	27558.75	22534.5	29894.25
Hist1h2bk	26791.5	21224	10099.5	16720
Hist1h2bm	39644	32598	2403	26819
Hist1h2bp	25296	23413	37294	33285
Hist1h3a	38625	29439	39461	1336
Hist1h3d	25499	22700	39150	36611
Hist1h4a	2074	40404	3822	2148
Hist1h4d	19203.5	12939.5	14646.5	11861.5
Hist1h4f	18528	13782	16573	15587
Hist1h4i	35330	34801	692	35651
Hist2h2aa1	15529	16034.5	17772.5	14252.5
Hist2h2aa2	22802	21343.5	22478	23760
Hist2h2ac	21262	21134	25193	24262.5
Hist2h2bb	6228	14805	16657	11711
Hist2h2be	19638	11793	17963	12499
Hist2h3c1	31022	25747	40477	37560
Hist2h4	4568	39855	2218	23
Hist3h2a	26291.66667	23901.33333	24259.33333	24977.33333
Hist3h2ba	15547	8922	23936	13000
Hist3h2bb-ps	31836	30092.5	19428.5	12792

Hist4h4	22060	21282	25319	11031.5
Hivep1	30065	35359	26758	31694
Hivep2	20294.5	20118.5	22729	22475
Hivep3	10876	9206.2	19101.8	11484.2
Hjurp	4421.5	4704	11182.5	10431
Hk1	17632.16667	17497.83333	19475.83333	20228
Hk2	6186	7966	5723	9528
Hk3	29700	20301	21286	19172
Hkdc1	39456	6047	19398	18680
Hlcs	24384	18793	27920	22351
Hlf	11685	12258	18027	19098
Hltf	16094.66667	16298.66667	15155.33333	14936.33333
Hlx	24925	23236	18778	17674
Hmbox1	18549.33333	13734.66667	23381.66667	22925.83333
Hmbs	40449	2683	39978	357
Hmces	16791.5	15630	14947.5	16481
Hmcn1	25699	26827	9986.5	12179
Hmg20a	7740	25648	4943.666667	29180.66667
Hmg20b	38228	25272	24660	17199
Hmga1	7896	6794	11663	14187
Hmga2	22379	26104	26923.33333	25743
Hmga2-ps1	1002	29478	3146	30061
Hmgb1	25442	21261.4	20918.8	18731
Hmgb1-rs17	7897	10708	10372	9030
Hmgb2	18524	29265.5	27346.5	29647
Hmgb3	39049	2932	2886	6753
Hmgb4	2299	928	36086	34455
Hmgcl	21388	13913	20386	16458
Hmgcll1	17418	8614	12625	19036
Hmgcr	18050	20245.4	14767.6	17624.4
Hmgcs1	23088.66667	11643	21338.33333	20539
Hmgcs2	21175.5	21765.5	24886	11248.5
Hmgn1	30003	25114.5	24690	24326
Hmgn2	21735	35676	25727	36815
Hmgn3	10255	21068.5	7436	17387.5
Hmgn5	6137.5	848	1573.5	33778.5
Hmgxb3	14443	13208	13474	12560
Hmgxb4	10586.66667	10572.33333	11485.33333	11359.66667
Hmha1	8323	16427.5	26590	16824.5

Hmmr	2545	39110	9711	6896
Hmox1	22811	24529	27999	34947
Hmox2	20063	20555	23650	21999
Hmx1	39823	823	6639	6681
Hmx2	4749.5	5229.5	15280	11546.5
Hmx3	36393	36565	38211	36160
Hn1	4395	40922	4029	40687
Hn1l	33204	4101	30229.5	2670
Hnf1a	4814	9433	10918	11472
Hnf1b	10164	10959	10570.5	28984
Hnf4a	11338.33333	17324	16578.33333	16220
Hnf4g	13901.5	15840.5	19194.5	18725.5
Hnmt	20031	12617	13117	3806
Hnrnpa0	16606	6954	39328	31985
Hnrnpa1	17246.14286	11297.57143	10719.28571	15140
Hnrnpa2b1	6564.5	3837.5	7691	7854.5
Hnrnpa3	24894	21525	14365.5	24179
Hnrnpab	12182.25	11781	11435.5	12979
Hnrnpc	36798.5	19939	2718.5	4297.5
Hnrnpd	20718.5	22712	22107.5	26010
Hnrnpdl	12804.5	13406.5	14651.25	15494.25
Hnrnpf	33355.5	33212	33536.5	35797.5
Hnrnph1	4264	5047	5959	8690
Hnrnph2	11974	11931	14113	14128
Hnrnph3	21552.33333	10409.66667	19669	12799
Hnrnpk	9281.333333	11009.66667	12754.66667	12021.33333
Hnrnpl	32500	28071	29763	32078
Hnrnpll	20374	27253.25	28959.75	26620.75
Hnrnpm	17483	15567	19268	18109.5
Hnrnpr	34498.5	30187	9049	27989.5
Hnrnpu	11291.66667	20504.66667	14081	13482.66667
Hnrnpul1	19932.5	19237.75	10194.5	9737.5
Hnrnpul2	4120	32819	12740	10845
Hoga1	40313	38524	4191	2989
Homer1	24390.5	19508.5	23112.66667	20608.16667
Homer2	24719.66667	16017.66667	34287.66667	11318.33333
Homer3	33836	37138	35265	36956
Homez	3869	21762	20313	19655.5
Hook1	32198.25	23102.25	29048.75	23310.75

Hook2	21322	22454	22783	23785
Hook3	16691.75	15495.5	15750.25	15455.25
Hopx	23119	21321	25219	17477
Hormad1	16615	29787.5	29653	16087.5
Hormad2	25777	27206	29091	34042
Hoxa1	6446	207	20918	16689
Hoxa10	32233	34641	36019	38916
Hoxa11	29911	31408	5181	31344
Hoxa13	7135.5	21223.5	20786	23166
Hoxa2	38430	38301	32139	6868
Hoxa3	23404.5	22666	28446	10404.5
Hoxa4	27075.5	28941.5	30028	28535
Hoxa5	35541	26451	3453	2249
Hoxa6	36371	38849	5971	6391
Hoxa7	20635	22675	25926	29680
Hoxa9	40209	35839	3849	6321
Hoxb1	34420	37251	38880	37995
Hoxb13	38167	25340	6343	2205
Hoxb2	28430.5	26837	27420.5	31044.5
Hoxb3	36037.5	36575.5	38288.5	20086
Hoxb4	16993	11119	4863	795
Hoxb5	12600	2179	17933	12958
Hoxb6	32131.5	37201.5	624	20803
Hoxb7	14009	17466	20604	20943
Hoxb8	26365	29987.5	15500.5	15039.5
Hoxb9	17044	28199.5	26542	27741.5
Hoxc10	15032	19302	24998	7020
Hoxc12	12834	34754	4089	37687
Hoxc13	31615	37615	10262	6347
Hoxc4	25634	30372	36146	37861
Hoxc5	38557	37955	34891	37080
Hoxc6	37356	39603	40103	40976
Hoxc8	31655	33307	34765	32976
Hoxc9	36023	1001	10921	16490
Hoxd1	31693	34093	2926	32791
Hoxd10	11372	13748	15429	14495
Hoxd11	24033	25085	27606	32046
Hoxd12	15349	17308	24601	30452
Hoxd13	13263.5	34775	36178	36711

Hoxd3	22168	21950	25721.5	25084.5
Hoxd4	32616	26059	1939	3973
Hoxd8	29759	32811	32878	31330
Hoxd9	23936.5	23122	26074.5	27834.5
Hp	19142	29477	12201	16170
Hp1bp3	18336.66667	10834.66667	31800.33333	32458.33333
Hpca	27572.5	13116	16012	11402.5
Hpcal1	15572	9220	7409.5	2092
Hpcal4	33755	36433	39368	37463
Hpd	4635	40520	7702	5364
Hpdl	3859	10990	4699	16780
Hpgd	28550	15345.75	26083	26213.75
Hpgds	35653	11653	19702	32
Hpn	30774	20950	13485.5	20615
Hprt	22521	28386	24542	24477
Hps1	21034.5	19899.5	36962.5	35175.5
Hps3	28633.5	20169	19119.5	22298
Hps4	11744	8752	9516	9027
Hps5	3104	1333	2347	40657
Hps6	24778	23741	24111	24416
Hpse	34276	25308	22663	21314
Hpse2	25698.5	21298	32289.5	32837
Hpvc-ps	7258	10138	13743	13369
Hpx	2983	4625	11941	11590
Hr	13589	8231	10390	12584
Hras	27662	30987	23383	25231
Hrasls	32154.66667	31932.66667	37765.66667	33485.66667
Hrasls5	18084	23989	20632.5	21876
Hrc	39687	34265	8901	18753
Hrct1	11318	18947	8405	4637
Hrg	10186	14301	16278	15979
Hrh1	22472	22247	26031	23221
Hrh2	25298	37700	25874	26408
Hrh3	18183	20750	20724	20888
Hrh4	39543	30506	38963	39673
Hrk	37203	26179	36453	2256
Hrnr	31845	30057	18226.5	32638
Hrsp12	6218	10342	1369	2621
Hs1bp3	16218.66667	17338	14379	15598

Hs2st1	9635	12863.5	10582.5	14399.5
Hs3st1	28230	25546	27461	26375
Hs3st2	29482.5	35373	19992.5	32984
Hs3st3a1	6356	38865	9419	40219
Hs3st3b1	4007	34193	8525	39933
Hs3st5	24478	33891.66667	32793.33333	32569
Hs6st1	7754	37817	6060	450
Hs6st2	33276.33333	27751.66667	25704.66667	19372.66667
Hs6st3	23403.5	24121.5	25989.5	27000.5
Hsbp1	7158.666667	8167.333333	8431.666667	8768
Hsbp1l1	23352	38467	26668	8902
Hscb	30664	27305	26756	23981
Hsd11b1	38063	7859	5315	6932
Hsd11b2	2449	1697	3807	6285
Hsd17b1	15153	14485	14573	12383
Hsd17b10	4595	9147	10004	10847
Hsd17b11	26514.66667	13359	26676.66667	16067.66667
Hsd17b12	22051.66667	34241.33333	29097.33333	31533.66667
Hsd17b13	12260	19913.5	19940	34509.5
Hsd17b14	12085	17342	8217	20180
Hsd17b2	11132	16860	18941	8083
Hsd17b3	18042	22673	22245	20280
Hsd17b4	16368	13601	16614	15102
Hsd17b6	21928	25772	9843	9575.5
Hsd17b7	21985.5	19201.5	30253.5	28522.5
Hsd3b1	12041	19042	18510	17118
Hsd3b2	10388	25210.33333	19968.66667	18379
Hsd3b3	32879.5	33167.5	32897.5	14986
Hsd3b5	36227	37805	40733	38862
Hsd3b6	38583	1630	27159	1573
Hsd3b7	6511	8304	10930	14135
Hsdl1	18315	17562	13676	15287
Hsdl2	11808.5	12309.5	39293	38696.5
Hsf1	24562.33333	23641.33333	23880.33333	23483.66667
Hsf2	27916.33333	23281	23228	17984.66667
Hsf2bp	28921	30409	40602	40409
Hsf3	30220	31838	33598	31827
Hsf4	17528	21891	18447	21040
Hsfy2	33306	28717	31526	30027

Hsh2d	7338	9694	11938	3253
Hsp90aa1	18345.66667	19660.33333	23979	24676
Hsp90ab1	21641.5	24661	21563.5	4744
Hsp90b1	37803.5	38159	17077.5	16971.5
Hspa12a	31606.75	30120	30668.5	32245
Hspa12b	22425	22157	23044	24033
Hspa13	15961.75	22829	17036	18021.5
Hspa14	1665	37608	39739	39623
Hspa1a	15610.5	19284.25	17272.5	26243.5
Hspa1l	6320	8309	13663	12994
Hspa2	28774	20269.5	27239	23411
Hspa4	27185.5	28791	27277.5	19640.25
Hspa4l	20410	12607.25	21667.25	12972.75
Hspa5	38574	366	38449	3356
Hspa8	27368	18411	22620	26698
Hspa9	26911.5	28124.5	26323.5	9825
Hspb1	8840	14611	18051.5	20857.5
Hspb11	22414.5	25982.5	25693.5	25297.5
Hspb2	20316	23120	25486	8363.5
Hspb3	39208	37904	30669	3987
Hspb6	25251	24676	21490	22875
Hspb7	16727	34372.5	33023.5	31522
Hspb8	22873	31685	26274	35255
Hspb9	16495	19142	24190	25691
Hspbap1	14781	20465	17504	20953
Hspbp1	11428	11270.5	28606	28748.5
Hspd1	26433.5	28764.5	25208	31182
Hspe1	2991.5	8810.5	6684.5	7776.5
Hspg2	18067	20912	24619	38804
Hsph1	38379	80	649	3280
Htatip2	3997.5	11061	11246	17558.5
Htatsf1	20898	19309	19426	16782
Htr1a	27705	29167	30476	29445
Htr1b	35322	30642	9673	40968
Htr1d	10771	13375	18089	19068
Htr1f	14788	9537	17816	12932
Htr2a	15560	17613	23738	24702
Htr2b	22936	23563	20215.5	2885.5
Htr2c	29856.66667	36776.66667	35861.33333	22153.66667

Htr3a	18586	16619	24399	25617
Htr3b	8157	23592	33463	31986
Htr4	21858.33333	22295.66667	13783	23848
Htr5a	15933	16140	22146	20606
Htr5b	25396	1962	28369	31696
Htr6	31614	23957	31068	27939
Htr7	2832	33973	3757	4673
Htra1	11619	21071	10555	18671
Htra2	20315	18563	19917	19680
Htra3	21558.66667	23451.66667	27158.66667	28111.66667
Htra4	24833	15167	24582	20130
Htt	27807	20817	22236	20253.5
Hunk	34529	38398	953	19115
Hus1	22525.66667	18681.66667	27721.33333	28552.66667
Hus1b	12005	3666	5623	8865
Huwe1	22480.85714	21407.57143	25962	20890
Hvcn1	19291	37202	19120	39537
Hyal1	14451.5	2137	20466.5	15141.5
Hyal2	11270	9440	10681	7686
Hyal3	30564.5	10854	12126.5	11414
Hyal4	4868	10591	16997	17669
Hyal5	27343	4427	31719	30126
Hyal6	33612	24515	27433	25482
Hydin	38261	965	4330.5	4254.5
Hyi	3205	2456	5234	3826
Hykk	28312	30943	32377.5	32096
Hyls1	17896	21477	22677	26190
Hyou1	29669.5	34419.5	28290.5	32421.5
Hypk	25613	9673.5	10235	10487
Hypm	3089	4727	6068	37652
I1C0022H11Rik	11169	10113	8063	8274
Iah1	23937.5	28042	22959	27977
Iapp	5251	7697	14224	13586
Iars	32285.66667	32179.33333	29323.33333	30866.66667
Iars2	32742.5	12888.5	29885.5	13494
Iba57	16400.66667	15812	16320	27483.33333
Ibsp	23643	29153	26435	37635
Ibtk	9042.5	10236.5	6259	9815.5
Ica1	10960.33333	11182.33333	25322	27466.33333

Ica1l	31029	14376	8342	8988.5
Icam1	31530.5	27940	35042.5	26246.5
Icam2	21659	20183	29631	27472
Icam4	38015	23814	37354	25911
Icam5	9635	12276	17751	16193
Ice1	18008	15523	15485	16197
Ice2	22142	28230.5	17277.5	18968
Ick	15531	16448.33333	17198.66667	18097.66667
Icmt	12769	10876	9444	9316
Icos	36673	20859	1508	20467
Icosl	23260	27524	4224.5	22000
Ict1	1810	5339	2222	3680
Id1	34327	272	20209.5	6077
Id2	10116	19130.5	9434	19441
Id3	21117	16907	22967	20315
Id4	30507	31494.5	20729.5	26095
Ide	18670	32793.5	29170.5	31376.5
Idh1	34372	22615	26755	21284
Idh2	19260	17970	13147	15338
Idh3a	29342	38632	28429	32596
Idh3b	2695	6148	4599	6961
Idh3g	14735	12210	12369	11741
Idi1	21068.5	3897.5	15519	37483
Idi2	21379	27532	35582	35826
Idnk	22857	31169	26023	31968
Ido1	39193	5638	5688	13245
Ido2	27195	37341	35601	21801
Ids	11133.33333	10371.66667	22747.66667	19743
Idua	8986	6128	1683	3926
Ier2	10153.5	7100	22007.5	23133
Ier3	2623	20047	8133	22216
Ier3ip1	20492	22061	21333.5	19446
Ier5	25265	19946	27796	24299
Ier5l	11999	36786	8989	12727
Ifaprc2	27762	29268	32291	30018
Iffo1	14973.5	6231.5	5210	19459.5
Iffo2	15602	12562	8632	6309
Ifi202b	27224	36269	38897	850
Ifi203	19257.5	11148	11591.5	14395

Ifi204	18450	7216.333333	21209	11454.66667
Ifi205	16205	19422.33333	23881.66667	25115
Ifi27	26717	15199.6	18610.8	17970.2
Ifi27l2b	10850	18392	16795	13260
Ifi30	20142	21309	16590	24507
Ifi35	13761	25194	17282	28968
Ifi44	16956	8985.5	19269	6638
Ifi47	9177	26170	10470	27571
Ifih1	12039	15818.5	8871.5	18222
Ifit1	6956	4976	16473	15023
Ifit1bl2	22037.5	29539	25703.5	34844
Ifit2	22502.5	17412.5	27877.5	21798
Ifit3	2534	17434.5	17271.5	7567.5
Ifitm1	23970	22143	29746	27504
Ifitm10	22843.5	23356.5	28262.5	26400.5
Ifitm2	15470	14359	16662	12514
Ifitm3	24669	19775	24727	22520
Ifitm5	36015	35213	40157	39751
Ifitm6	9086	21461	8251	18544
Ifitm7	18662	15920	21903	18328
Ifna1	26885	31304	31529	29561
Ifna11	25673	26777	29401	28134
Ifna12	3099	3391	5477	7229
Ifna13	12784	15464	19323	20749
Ifna2	4060	39434	17560	17603
Ifna4	36454	4816	14508	12047
Ifna6	29899	31658	2773	2078
Ifna9	10972	15223	16483	17371
Ifnab	12341	15997.5	18447	18024
Ifnar1	567	5242	1062	3301
Ifnar2	24107	27413	26455.5	10492
Ifnb1	8374	14400	3623	40767
Ifne	30074	31581	33394	31775
Ifng	18761	36056	34634.5	39599.5
Ifngr1	36470	24413	31942	24005
Ifngr2	18428.5	18611.5	13534.5	18019.5
Ifnk	10304	5205	13107	6214
Ifnl3	28607	38327	39168	37056
Ifnlr1	15589	26650	21631	27542

Ifnz	11944	8666	16527	14253.5
Ifrd1	6717	16344.5	12072.5	23737.5
Ifrd2	12250.5	14803	10078.5	10596.5
Ift122	17955	15489	21680	17263
Ift140	16976	18805.5	26525.5	24021.5
Ift172	29435.75	28024.75	27473	24630.25
Ift20	38180.5	39425	30854.5	32990
Ift22	9887	11433	9106	12757
Ift27	37871	33759	31316	31165
Ift43	11588	20278	20818	23978
Ift46	34266.5	33822.5	34546	29704
Ift52	29795	31763	24476	28708
Ift57	18007.25	24282	17446	33838.25
Ift74	1214	38764	31664	23971
Ift80	17500.66667	26961.33333	19481.33333	35061
Ift81	22331.66667	13956.33333	16043.66667	15774.33333
Ift88	26497	30000	25994	27612
Igbp1	30437	26863.5	28184.5	25797
Igbp1b	31426	35763	39854	31645
Igdcc3	26540	28731	33756.75	33580.75
Igdcc4	5439	39582	11881	14855
Igf1	18345.33333	24550.66667	25267.66667	26057.66667
Igf1r	25343.2	16570.8	24070.6	21323.4
Igf2	8928	22275	25246	29723
Igf2bp1	17222	11875	12980.66667	24549.66667
Igf2bp2	16514	13243	24533	24657
Igf2bp3	23125.5	21049.75	19836.75	21055
Igf2r	20144	19154	22352	19501
Igfals	16849	18313	30335	24375
Igfbp1	28317	37066	39514	39554
Igfbp2	23547	22368	25363	27762
Igfbp3	18782.66667	18160.66667	22485	21970.33333
Igfbp4	22165	31611.5	31648.5	26830
Igfbp5	27437	30054.5	38525	19014.5
Igfbp6	25468	22119	37261	31372
Igfbp7	3160	20195	12372	28064
Igfbpl1	16934	20239.5	18675	16426
Igfl3	10662	12937	17863	20253
Igflr1	11945	5674	8480	8334

Igfn1	30597	35286	34862	5981
Igh	13246.25	23937.5	16937.5	6791.5
Ighe	39273	31768	33999	32272
Ighg	21647.5	28112.5	25848.5	16322
Ighg1	14127.33333	18719.66667	25700.33333	17118.66667
Ighg2a	28958	19033.14286	22578	24811.85714
Ighg3	19321.5	16795.5	15716.5	10181.25
Ighm	18376	24005.66667	24268	25866.66667
IghmAC38.205.12	15949	21487	36119	8711
Ighmbp2	29553	31995	33006	36380
Ighv1-42	27358.5	14935.5	30681	11329.5
Ighv1-53	28124	28815	34808	27454
Ighv1-84	29403	30947	32265	31213
ighv3s2_m12435_ig_heavy_variable_3s2_32	22289	21263	30103	26772
Igh-VJ558	18382.64706	21038.23529	19669.29412	21181.58824
Igh-VS107	39443	787	15337	7478
Igk-V1	5677.5	10878	6228	12744.5
Igkv10-95	1980	40456	1444	3519
Igkv1-117	16827	18554	20562	22553
Igkv1-132	17329.5	20113.5	24672.5	7664.5
Igkv1-135	15307.5	30449.5	34408.5	33193
Igkv12-89	8279	18367	19205	21533
Igkv12-98	23868	25056	26517	25439
Igkv13-85	34783	36843	26985	35330
Igkv14-100	40686	38695	36123	26979
Igkv14-111	18610.5	21670.5	26030.5	27920.5
Igkv17-127	26613	28014	29386	28409
Igkv1-99	29201	30721	32098	32288
Igkv19-93	2958	12087	16454	16004
Igkv2-112	29008	30448	40239	35387
Igkv2-116	11622	17766	20274	22540
Igk-V28	14999	18130	17525	14853
Igk-V34	8758	14170	13120	17232
Igkv4-50	12942	27016	15959	15790
Igkv4-53	24929	35216	40775	171
Igkv4-55	32973	38259	23091	29593
Igkv4-57	21502	26463	33750	25430
Igkv4-57-1	4632	23460	15286	14266
Igkv4-58	42	3238	9293	9633

Igkv4-60	14793	34904	20216	22388
Igkv4-63	37990	36595	1147	9841
Igkv4-70	9296	12813	11838	7716
Igkv4-72	8107	15202	16231	17816
Igkv4-74	32127	2315	40820	1819
Igkv4-86	19733	20632	25796	31072
Igkv5-37	14290	18712	30977	25485
Igkv5-43	4664.5	14746.5	20914	20320.5
Igkv5-48	27965	29353	31416	32082
Igkv6-14	24219	25346	27572	33643
Igkv6-32	7208.5	12316.5	18274.5	8966.5
Igkv7-33	24179	30793.66667	16175	3943
Igkv9-120	20542.66667	23872	32854	30712
Igll1	23554	26614	36571	38559
Iglon5	8338	10092	16600	15325
Iglv1	16040	17993.625	21252.75	17676.625
Igsf1	29652.5	22124.5	25473.75	26326
Igsf10	33854	37474	37327.5	37303
Igsf11	24192	4625	21289	19673
Igsf21	32597	24004	29873	30365
Igsf23	38035	22142.5	21295.5	25658.5
Igsf3	27397.5	38753	37309.5	13820
Igsf5	27937.5	33609	23197.5	18157.5
Igsf6	21719	37010	22877	37847
Igsf8	6114	9647	6143	7880
Igsf9	35693	20283	20434.5	21931
Igsf9b	14315	16116	10409	4592
Igtp	28291	37471	27548	39857
Ihh	16735.5	20529.5	17046.5	22679
Iigp1	33125	23921	4601	35393
Ik	24057	24545	25487	26633
Ikbip	21283	4198.5	24160.5	21342.5
Ikbkap	17578	12800	12135	12881
Ikbkb	29328	26847	27319	25877
Ikbke	21924	28957	17595	24386
Ikbkg	21868.4	17110.6	20834.8	23901.4
Ikzf1	21168.66667	25962.66667	19681	32410.66667
Ikzf2	28219.83333	23235.83333	23538.66667	26560.83333
Ikzf3	25537.5	19201.75	22477.5	21519.5

Ikzf4	19318.66667	23246.33333	13776.33333	14838
Ikzf5	18108.33333	14316.66667	16652.33333	14256
Il10	27439	27002	36530	32988
Il10ra	454	35258	33445	26890
Il10rb	28398.5	17404	27579.5	23591
Il11	10440	13603	18144	18696
Il11ra1	16248.5	12520.5	14200.5	21293.5
Il12a	14559	25060	16194	37786
Il12b	40678	13193	8505	17156
Il12rb1	37544	12958	28970	11380
Il12rb2	32047	26457	29752	28625
Il13	35991	34782	39317	40030
Il13ra1	3697	12923	5452.5	12999
Il13ra2	31520	32917	341	4609
Il15	26635.5	25905.5	26517	25439
Il15ra	18140	25311	17688	25364.5
Il16	32642.5	14828.5	22549.5	8020
Il17a	30874	33409	34350	32558
Il17b	7619	5479	11501	11081
Il17c	8147	7419	10956	11533
Il17d	7601	11045	13900	14582
Il17f	29362	31014	40479	40961
Il17ra	6407.5	7565	21309	4399
Il17rb	6901.5	4839.5	13729.5	11336.5
Il17rc	24004	27748.33333	28466	19010
Il17rd	25583.75	28634.5	10921.5	23461.25
Il17re	27179	26206	33374.5	29111.5
Il18	9444	10561	10259	3497
Il18bp	36585	9264	37496.5	10236.5
Il18r1	4892	36271	32564	28791
Il18rap	17849.5	14127	18022	16271
Il19	4319	4780	30902	6542
Il1a	5966	24105.33333	7901.333333	27352.33333
Il1b	32247	2053	18165	40929
Il1f10	10237	7327	16850	7455
Il1f5	21282	23314	24424	25449
Il1f6	14445	1588	39846	9990
Il1f8	5931	14055	11553	14136
Il1f9	10400	12873	11607.5	24692

Il1r1	20798.5	23536	25377.5	27688.5
Il1r2	37659.5	36313.5	17713	8505.5
Il1rap	22248.2	21054	14331.6	18619.8
Il1rapl1	20390	16441.5	20323.5	15506
Il1rapl2	20359	18905	20564	24318.5
Il1rl1	6006.333333	18867.66667	12248.33333	8536.333333
Il1rl2	23498.5	6533.5	20684.5	5537.5
Il1rn	12004.5	24090	24626.5	14174
Il2	28417	29826	19071	37610.5
Il20	25102	26449	27793	26794
Il20ra	32080	36698	3756	4479
Il20rb	28700.5	17566.5	22683	15186.5
Il21	31993	33222	36899	34847
Il21r	27839	36005	27607	36667
Il22	29272.5	25601.5	17717	28333
Il22ra1	1826	2174	9320	6777
Il22ra2	30429.5	32034	33512.5	32155.5
Il23a	31180	36538	34402	32658
Il23r	30347	11349	8001	16523
Il24	18100	38436	5211	19820
Il25	8859	14306	15184	14192
Il27	38489	578	3163	4663
Il27ra	35397	5844	29123	38510
Il2ra	28108.5	28054.5	29342.25	30961.75
Il2rb	20997	28061	37276	38807
Il2rg	19126	253	17394	38901
Il3	37413	6239	38007	37609
Il31	29425	31004	31848	30486
Il31ra	3257	7837	8822	7808
Il33	27107	40976	35094	8520
Il34	31135.5	30609.5	14219.5	33587
Il3ra	33439	23567	21728	21002
Il4	3923	7648	9518	9269
Il4i1	6495	16607	6769	14652
Il4ra	27818	28523	22851	30183
Il5	5896	2451	31447	29950
Il5ra	40961	4839	33150	31626
Il6	33100	38749	2248	7960
Il6ra	19586	29221	29302.5	23223.5

Il6st	39952	20704	25908	20043
Il7	27842.66667	31250.66667	19241.33333	18413.66667
Il7r	24172.33333	18682.66667	24470	23051.33333
Il9	28791	30272	31678	30624
Il9r	12932	15213	20068	18068
Ildr1	30965.5	32828.5	33339	17932
Ildr2	8587	17559.5	26731	27082
Ilf2	3084	6041	4584	6240
Ilf3	26467	28242.5	28205.25	28354.75
Ilk	4390	39206	37739	34996
Ilkap	1692	7492	9254	10617
ilmn_199374	23473	10102	23563	13580.5
ilmn_200966	40357	1868	2545	5284
ilmn_224753	419	28990	32091	30528
ilmn_237015	39580	32213	38912	1180
ilmn_245270	12949	12948	19481	17653
ilmn_248993	37431	29415	35748	28513
ilmn_252400	17421.5	14311	18727.5	13877
Ilvbl	24987	21730	19329	20556
Immp1l	25031	24539.4	17054.4	15866.2
Immp2l	24694.5	10643.5	26360	12700
Immt	18852	19692	14882.5	18273.5
Imp3	40523	540	38846	40255
Imp4	20755	21700	20602	21794
Impa1	18618	22626.5	18667	23634.5
Impa2	12723	10615	5895	3548
Impact	8637	4748	10109	3307
Impad1	16569.5	18556	15183	16306
Impdh1	19169	17402	24264	23081
Impdh2	24892	24439	20949.5	22706.5
Impg1	20264.5	18108	26623.5	6028.5
Impg2	19764	21519.75	26277	24870
Ina	30430.5	16399	17892.5	17398.5
Inadl	12717.33333	28779.66667	24742.33333	35993.33333
Inca1	23177	24403	31333	38517
Incenp	5346	39899	5316	3641
Inf2	33475	35301	33293	38719
Ing1	16507.5	15645.5	16472	14837
Ing2	17507.66667	19173	21806.33333	21344.66667

Ing3	30370	36179.5	30727.5	30382.5
Ing4	27964	19330.66667	22355.33333	17049.33333
Ing5	10985.66667	14829.66667	13053	15872.33333
Inha	30965	12738	30509	31527
Inhba	19657	15921	39651	26044
Inhbb	26185	28259.5	12748	20162.5
Inhbc	11984.5	10853.5	21727.5	20178
Inhbe	36510	2010	298	3333
Inip	24857.5	29692.5	20979.5	24369
Inmt	40179	6674	7237	11877
Ino80	14856.33333	24387	18873	16775
Ino80b	14748	14900	14445	12513
Ino80c	10631.5	15797	16204.5	18860.5
Ino80d	296	911	33860	37869
Ino80e	23185	22294	21919	21243
Inpp1	32119	19286	30287.33333	22355
Inpp4a	12294	9390	12794	11202
Inpp4b	32295.33333	31357.33333	34886.33333	31211.66667
Inpp5a	9457	6466	5293	5176
Inpp5b	22366.5	22757	22093	21850
Inpp5d	3551	36257	39236	35456
Inpp5e	12975.5	11741	29759	27238.5
Inpp5f	12361.5	27858	14535.5	11706
Inpp5j	34779	23870	24717	22843
Inpp5k	22169	19858.5	18970	19317
Inppl1	881	28712	3226	35175
Ins1	15470	13187	10134	13737
Ins2	17619	26309.5	22563.5	22267.5
Insc	14180	19966	5000	23929.5
Insig1	25427.5	14490	23163	26944
Insig2	17316	16791	17756.33333	17293.66667
Insl3	34577	30715	28451	24892
Insl5	22473	24136	26043	26480
Insl6	31593	1931	31136	35516
Insm1	37198	40284	26899	25776
Insm2	22717	21176	32810	33041
Insr	37139	30008	12827.5	29863.5
Insrr	38664	187	8234	8622
Ints1	14107	11027	11738	7015

Ints10	18493.5	18413.5	18878.5	18307
Ints12	26698	32631.33333	28677.66667	19811.66667
Ints2	16001.33333	14830.66667	14064.33333	24577.66667
Ints3	6684.5	24116.5	25030	24930
Ints4	14827.5	40486	36323.5	19705.5
Ints5	9091	9673	8345	6325
Ints6	12866.33333	10581.66667	12681.33333	15809
Ints7	15094.75	12075.75	15353.5	13703.25
Ints8	15412.33333	14005.33333	13643.66667	12171
Ints9	25751	20556	23216	22506
Intu	18718.33333	19120	22200.66667	23812
Invs	29542.66667	14101.66667	15647.66667	17372
Ip6k1	37465	16231.25	19406.75	17651.75
Ip6k2	15700.5	18126	17893	23656.5
Ip6k3	20075	6967	23615	40936
Ipcef1	40003	18140	13897	1747
Ipmk	15754.25	17827.75	14333.5	16067.75
Ipo11	28040.6	21536.4	26388.4	21485
Ipo13	26794	29973	22437	34112
Ipo4	28546	10444.5	11186	11879
Ipo5	27802.33333	27819.33333	19471.66667	28690
Ipo7	19612.75	23234	14073.25	17617.75
Ipo8	26501.33333	22871	20851	20297.33333
Ipo9	19946	22365	19638	22707
Ipp	22025.33333	18911.66667	15958.33333	16332
Ippk	25236.66667	24458.66667	23103.33333	28751.33333
Ipw	24051	28943	27367	29024
Iqca	36107	33595	35019	33224.5
Iqcb1	20341	21365	19062	21195
Iqcc	13048.5	15574	15084.5	35865
Iqcd	11396.5	13317.5	18175	16949
Iqce	22240.33333	19104.33333	20749	21071.66667
Iqcf1	5592.5	6864.5	13199.5	17192
Iqcf3	729	36827	4897	11045
Iqcf4	10105	14994	27497	22468
Iqcf5	11838	7293	16485	12215
Iqcg	33554	36393	35018	37998
Iqch	19382.5	17545.5	23923	21633
Iqcj	32104	33714	35864	34008

Iqck	13614	11569	18956	15767
Iqgap1	16396	7438.5	9993.5	25752
Iqgap2	27480.5	12094.5	21794.5	5805.5
Iqgap3	22183	16590	20220	35202
Iqsec1	29276.5	20312	22000	20806
Iqsec3	31804	34309	34898	32933
Iqub	9707	7247	17586	18090
Irak1	16814.33333	26121.33333	23463	23094
Irak1bp1	29666	32956	22539	22300
Irak2	39191	1082	33	40051
Irak3	20905.66667	19186.33333	14652	16161.33333
Irak4	13986.5	14912	16536	13547
Ireb2	22958	20797	16974	21133.5
Irf1	6509	19465.5	25945.5	18562.5
Irf2	8227	7867	14081	9530
Irf2bp1	3652	3975	3832	40449
Irf2bp2	2653	5156	8005	6135
Irf2bpl	22368	15235	17000	13252
Irf3	15131.5	9990.5	9418.5	6222
Irf4	24823	18486	31051	29352
Irf5	13365	3710	10144	4572
Irf6	28915	32114	10531.5	13454.5
Irf7	38037	9065	39749	16645
Irf8	21935	15718	19717.5	16118
Irf9	18828.5	26425.5	21947.5	31826
Irg1	10284.5	36860.5	15716	38436.5
Irgc1	12086	8106	20880	17982
Irgm1	29051.33333	14692	28797.33333	14191
Irgm2	19052	21720	18309	26765
Irgq	3710.5	20650	15867	18199.5
Irs1	28023.33333	28676	31953.33333	33979
Irs3	37364	9943	7665	4344
Irs4	21885.5	18898	22459.5	21922.5
Irx1	15485	17951	6037.5	19283.5
Irx2	18646.33333	11618	26198.33333	16224.66667
Irx3	26144	10610	29852	28462
Irx4	11536	17479	14656	19570
Irx5	30921	32610	39851	32926
Irx6	40177	3463	7046	6447

Isca1	23931	21289	20365.33333	21497.66667
Isca2	925	5179	3513	2952
Iscu	20835	28361	23941	26298
Isg15	2192	27761	11264	37410
Isg20	11342	22423.5	17035.5	26766
Isg20l2	17526.5	18821	19841.5	18710.5
Isl1	9555	5544	12751	13421
Isl2	26405	27524	33412	29533
Islr	24337.5	22023.5	32588.5	36064
Islr2	25588	26864	28566	28712
Ism1	12637	21859.5	1897	8279
Isoc1	26873	32893	19720.5	26722.5
Isoc2b	13059	9923	12439	9935
Ispd	25404.33333	25324.66667	21527.33333	16435.33333
Ist1	37337	38631	39562	37815
Isx	27889	29225	31747	30248
Isy1	24628.5	26333	28549.5	28496
Isyna1	35697	36926	23564	26043
Itch	23549.5	17268	17460.25	18485.75
Itfg1	21233.75	26193.5	26264.25	27332.75
Itfg2	26869	33848	28883.5	30638
Itga1	13809.66667	20902.66667	18527.66667	14754.33333
Itga10	11963	1199	13310	11134
Itga11	18471	21555	33595	34311
Itga2	30309	31409.5	16400.5	13810
Itga2b	15792	16401	6877	13864
Itga3	15031.33333	18931.33333	22763.66667	10833.33333
Itga4	16506.625	20670	21601.625	17975.25
Itga5	38392	31	40328	2234
Itga6	11827	15359	19436.33333	22586.66667
Itga7	20404.5	4086.5	4243.5	17973.5
Itga8	16672.66667	15922	18521.33333	18180.33333
Itga9	20480.66667	30611.33333	24027	34383.33333
Itgae	8820	20472	13364.5	7365.5
Itgal	5481	17442	39858	7553
Itgam	35374.5	25032	30753	24117
Itgav	22086.5	23096.83333	25184.66667	23140.66667
Itgax	34921.5	31804	24338	28693.5
Itgb1	10444.66667	10268.66667	26122	15269.33333

Itgb1bp1	28901	17744	11705.5	14984
Itgb1bp2	16610	19624	15372	22827
Itgb2	4509	1166	1759	4036
Itgb2l	12535	16235	21307	22235
Itgb3	467	2750	3898	4456
Itgb3bp	22561.33333	28079	22038	14814.33333
Itgb4	15158	16963.33333	17342	15201
Itgb5	38533	17936	30309	15414
Itgb6	26725.5	24981.75	28717.25	23265
Itgb7	10839	5459	16643	12519
Itgb8	22069.4	23128.2	22388.2	13193.8
Itgbl1	38026	31903	6327	36344
Itih1	36197	38672	39678	38294
Itih2	35855	37236	209	40841
Itih3	25151	23299	32839	32582
Itih4	5522	8284	15767	16450
Itih5	19206	20474.5	22714.5	10304.5
Itih5l-ps	39343	38906	13531	14985
Itk	27727	27952	29099.33333	27565
Itln1	28421	31196	7446	899
Itm2a	14625	18830	27237	997
Itm2b	1074.5	27149	1109	32259
Itm2c	4492	5264	6145	4450
Itpa	18630.33333	19845.66667	31269	35973
Itpk1	6399	6258	8437	4415
Itpka	7622	9852	8523	11219
Itpkb	25834	27274	28694	27543
Itpkc	11271.5	12634	20664.5	19147.5
Itpr1	17531.66667	12248	14456.66667	18759
Itpr2	24573.77778	20975.77778	17840	20640.66667
Itpr3	13622	7794	18630	14593
Itpripl1	19747	16190	22317.5	16712
Itpripl2	30436	15878	13879	12447
Itsn1	19320.8	17734	21730.4	23831.8
Itsn2	18838.83333	19905.33333	19379.5	21463.33333
Ivd	22309	19356.5	38783.5	18350
Ivl	40826	41040	3324	1616
Ivns1abp	16782.66667	12623	16787.66667	26710.66667
Iws1	14446.5	16072	13746	14437.5

Iyd	31982	34234	34317	34872
Izumo1	28478	30234	30265	29147
Izumo1r	24247	23093	29298	28295
Izumo2	6223	8237	8065	8677
Izumo3	32850	40554	7095	329
Izumo4	12954	12249	15455.5	13736.5
Jade1	3328	13195	8125	16568
Jade2	28857	17022.66667	22153.66667	14904.33333
Jade3	17117.66667	19674.66667	18934	23726.33333
Jag1	24277.5	13424	11876.5	21803.5
Jag2	26553	27664	30357	29018
Jagn1	37472.5	30735	26946.5	28567.5
Jak1	29053	26706	8692.5	9322.5
Jak2	29161	13950	29547	15507
Jak3	15224.5	15730.5	14645.5	12595
Jakmip1	34902.25	31386.5	27922.25	31574.75
Jakmip2	26104.83333	23698.83333	21229.83333	25558.33333
Jakmip3	30780.5	30211.5	18500.5	20610.5
Jam2	11515.5	17544	14978	21903
Jam3	28008	26893.5	29009	34159.5
Jarid2	22128	23565	23430.66667	27694
Jazf1	23671	18519.5	31473	27759.5
Jchain	23243	26878.5	19353.5	11286.5
Jdp2	10273	17829	15027	16212
Jkamp	9853.5	8778.5	7666	5786.5
Jmjd1c	21145	23290	9906	12973.4
Jmjd4	41038	37931	34198	37496
Jmjd6	25681.66667	23877	17561.33333	21813
Jmjd8	31209	26411	24426	23437
Jmy	19386.5	29942	12632.5	30558.5
Josd1	20082	20600	18809	20580
Josd2	17946	20170	23783	24163
Jph1	32831	24755	1649	20679
Jph2	26964	28231	14353	16444.5
Jph3	35971	41082	2511	3559
Jph4	4222	7934	15420	16283.5
Jrk	6999.5	8322	30509.5	11910
Jrkl	19394	24382.5	21390	21731.5
Jsrp1	25703	26492	30012	31833

Jtb	8789	11148.5	10668.5	9223
Jun	4830	34232	7816	39195
Junb	20393	24830.5	19276.5	24933
Jund	34024	35705	39341	40109
Jup	13282	9628	16463	6608
Kalrn	22407.8	33346.6	27760.6	28139.8
Kank1	26296.33333	26054.66667	29681.66667	34834.66667
Kank2	19181	23506	16606.5	18241
Kank3	18548	14186	16206	11736
Kank4	32536.5	30566.5	32632.5	31057.5
Kansl1	22069	22050.4	15766.6	30484.2
Kansl1l	23873.75	21423.75	23684.375	22281.125
Kansl2	35788	37717	33636	38817
Kansl3	28064.5	21059.5	21704	22445
Kap	2536	3777	40977	6916
Kars	36572	37307	34506	37627
Kat2a	32764	32274	37289	29610
Kat2b	16366.66667	16745	14752.66667	15593
Kat5	23156	26186	29831	33407
Kat6a	15992	26105	24992	19691.5
Kat6b	21266.66667	27038.66667	29148.66667	29515
Kat7	17063	11714.5	25944	12893.5
Kat8	38152	40564	2072	1026
Katna1	25696	23669	22991	24407
Katnal1	10955	10870	10553	11589
Katnal2	9938.5	4243	13778.5	7609.5
Katnb1	22300	21746	20757	20691.5
Katnbl1	18456	22790.66667	22040	25091
Kazald1	17413	14144	19244	15959
Kazn	22407.33333	19147.33333	32303.33333	25309.66667
Kbtbd11	7047	17060.33333	13998	26933.66667
Kbtbd12	30650.5	13769	28175	28140.5
Kbtbd13	32409	36364	29428	35374
Kbtbd2	12536.66667	14381	14886	18064.66667
Kbtbd3	21439	25656.5	19480.5	19105.5
Kbtbd4	8501	4731	3702	5072
Kbtbd7	19165	16669	16429	18308.5
Kbtbd8	23596	19760	19905	16715
Kcmf1	22487.33333	22592.66667	20009.66667	21857

Kcna1	35030	36127	34007	37731
Kcna2	23808	19679	25488	25542
Kcna3	27268	24998	678	37686
Kcna4	41026	3423	1365	5409
Kcna5	29523	31028	32808	40348
Kcna6	23861	26847.5	29885	28137.5
Kcna7	21696	35635	22982	24707
Kcnab1	19553.25	8161	21526.5	22656
Kcnab2	22876	21333	23149	21163
Kcnab3	29512	31422	27516	34483
Kcnb1	15650.5	15672.5	19341	18737
Kcnb2	18670.5	32993	34216.5	34288
Kcnc1	25410.33333	25777	27497.33333	23976.33333
Kcnc2	23722.25	21648.5	24665	24009.25
Kcnc3	26658	29046	18658	17830
Kcnc4	8219	14395	13374	16162
Kcnd1	26820	32420	38204	38815
Kcnd2	22900.75	28236.5	21783	30251.25
Kcnd3	15352	18728	19390	19478
Kcne1	24426	25454.5	25569	26213
Kcne1l	30660	32536	33862	32100
Kcne2	36974	8209	6861	4086
Kcne3	11774	25973.5	4075	12430
Kcne4	32062	25974	27846	31737
Kcnf1	30248	38630.5	39275.5	2401
Kcng1	8502	11929	14612	16351
Kcng2	39358	14355	32987	12327
Kcng3	14655	22809.5	21482	24028
Kcng4	13300	15946	12791	18436
Kcnh1	34349	37008	34704	17398.5
Kcnh2	2748	22620	20676	25962
Kcnh3	15278.5	15896.5	16623	17667
Kcnh4	40607	5301	9677	10471
Kcnh5	10776	17420	4983	12737
Kcnh6	7610	14359	12292	16908
Kcnh7	25658.66667	31934.5	33874	22887.5
Kcnh8	27767	33410	30615	29317
Kcnip1	39667	688	18395	14527
Kcnip2	1488	2613	30966	2442

Kcnip3	16244	20801.5	20885.5	7592.5
Kcnip4	34602	20450.66667	16298	16568.66667
Kcnj1	6707	12015	23694	31659
Kcnj10	22015.5	18656.5	22649.5	22231.5
Kcnj11	12483	13604	5826	11527
Kcnj12	35932	36945	31640	812
Kcnj14	36995	7958	40344	3853
Kcnj15	26784.33333	21823.33333	24668.66667	5465
Kcnj16	25758	22785.66667	27419.33333	29813.33333
Kcnj2	20105	19422	21483	20906
Kcnj3	26913.66667	3542.666667	17237.66667	6772.666667
Kcnj4	30694.5	33070	19930.5	18610
Kcnj5	13874	10632	6551	11677
Kcnj6	16676.5	16464	19954.5	18425
Kcnj8	16840.5	16208	28536	23971
Kcnj9	14171	11484	15477	13517.5
Kcnk1	23557	24616	26406	34726
Kcnk10	16176	27136	7921	25335.5
Kcnk12	17728	21436	30105	32017
Kcnk13	32880	38234	39185	38714
Kcnk16	39570	28772	30813	33330
Kcnk2	29185	32724	29043	27974
Kcnk3	40664.5	2327.5	4289	5025
Kcnk4	23742	29007	38787	38257
Kcnk5	20651	19948.5	18344.5	24549
Kcnk6	3663	35288	651	34140
Kcnk7	36196.5	20188	21555	16798
Kcnk9	13684	10010	12422	15074
Kcnma1	20397.5	22587.5	28857.5	34392.5
Kcnmb1	31217.5	15383	33663.5	28463.5
Kcnmb2	10143	14178	23611	19847
Kcnmb4	32537	33870	37105	35128
Kcnn1	1804	40259	40117	37608
Kcnn2	18081.33333	19978.66667	21472.66667	25252.66667
Kcnn3	9485	7060	19226	16653
Kcnn4	31325	23852	21230	21940
Kcnq1	17731	28718.6	29911.2	26677.8
Kcnq1ot1	37094.5	29497.5	26454.5	28689.5
Kcnq2	35121	27178	37532	37067

Kcnq3	29132	36348	8401	4238
Kcnq4	13408	8876	9034	10610
Kcnq5	24178	24400.5	30331	30924.5
Kcns1	5813	6437	8193	6873
Kcns2	26612	23269	31921.5	26198
Kcns3	26576	28016	29341	28308
Kcnt1	21289.66667	24815.33333	16504	17103
Kcnt2	25179	31099	28956	29117
Kcnu1	21579	23444	37024	37547
Kcnv1	27637	28899	30779	29290
Kcnv2	29348	30863	31812	36420
Kcp	36307	40704	1158	2092
Kctd1	18792.5	26762	19759.5	27790
Kctd10	13145	12263	8855	8640
Kctd11	23746	24988	26480	27116
Kctd12	12548	17870.5	27878	14839
Kctd12b	25478.33333	9904	17709.66667	4326
Kctd13	7043	6472	4907	3514
Kctd14	24808	26033	28015	31529
Kctd15	4527	4462	9426	11920
Kctd16	12995	15843.5	24627.5	20682
Kctd17	10387	12752	9337	12367
Kctd18	29831	26852.5	28455	27812.5
Kctd19	14029	15174	24941	28652
Kctd2	26215	20295	21907	18748
Kctd20	14940.25	14510.75	18391	18706.75
Kctd3	11998.5	16956.5	10382.5	13150.5
Kctd4	24490.8	23295	27926.8	11738
Kctd5	26575	28362.5	27491.5	28066.5
Kctd6	6674	5489	4830	5530
Kctd7	22414.5	10233	23950.5	9729
Kctd8	31327.5	30677.5	29138.5	27913
Kctd9	7951	9666	7174	11506
Kdelc1	12521.5	14284	12293	13072.5
Kdelc2	35412.5	35392.5	27992.5	26453.5
Kdelr1	16341	10240	6911	5201
Kdelr2	24711	28144.5	10437.5	12502.5
Kdelr3	39901	6963	8733	14788
Kdf1	13849	3808	5466	5380

Kdm1a	38051	36011	37731	38709
Kdm1b	11250	11265	9599	13396
Kdm2a	11782.25	20527.25	22284.5	22562.75
Kdm2b	12189.33333	20619.33333	20066	12609
Kdm3a	13433.33333	21388.33333	24326.33333	25951
Kdm3b	40606	2396	4239	4879
Kdm4a	27909.5	29298	29369.5	29746.5
Kdm4b	24517	24804	24124	23502
Kdm4c	15946	9353	12227.66667	22869
Kdm4d	38857	4918	14076	10721
Kdm5a	10022.5	32558.75	20825.5	17283
Kdm5b	23668.25	15369.25	13394	5809
Kdm5c	13160	14154	17091.5	17834.5
Kdm5d	37177	35549	33230	29859
Kdm6a	15233	21248.33333	20554.33333	20478.66667
Kdm6b	1317	30681	38655	31122
Kdm7a	14574.33333	18029.66667	13626.33333	21027.33333
Kdm8	7956	6994	3005	4101
Kdr	33036	26153	27344.5	22729.5
Kdsr	16108.33333	14448	13266	14813
Keap1	6554	5107	2289	854
Keg1	20944	19950.5	28564	21643
Kel	36355	35709	5008	8267
Kera	34887	27990	31349	29805
Khdc1a	20874	21522	22707.5	25166
Khdc1b	19538	19797	26770	27914
Khdc3	30037	33498	37435	38695
Khdrbs1	13198.75	13005	14731	13479.75
Khdrbs2	30551.5	26888.5	24809	24547.25
Khdrbs3	4242	11132.5	11398.5	11469.5
Khk	25071.5	19449.5	21678.5	13889.5
Khnyn	34791.66667	22805	22866.33333	23490.66667
Khsrp	31754	22506	24412.5	21904
Kidins220	7324	19869	9836	5109.5
Kif11	25897.33333	26377.66667	17637.33333	16963.66667
Kif12	24680	12376	19220	23929.5
Kif13a	14750	22193	19818.25	16753.5
Kif13b	7799	24597.66667	6519.666667	2219.333333
Kif14	1922	37692	36577	4053

Kif15	26777.75	25951.5	28270.5	27338.5
Kif16b	14549.75	20418.75	21137.5	21105.5
Kif17	6356	14409.33333	29506.66667	30787.33333
Kif18a	29146.5	23643	28800	27469.5
Kif18b	2822	1716	10258	15937
Kif1a	26820.5	20977	21470.5	20728
Kif1b	28940	21531.8	24013.2	23078.2
Kif1bp	24994.33333	24877.33333	25517	25002
Kif1c	23882.5	23715.75	21228.75	22176.25
Kif20a	26271	28550.5	31018	33112.5
Kif20b	26629	24821	24754.33333	14113
Kif21a	31569.66667	27854.66667	14624	6010.666667
Kif21b	16705.66667	28857.66667	19835.66667	31873.66667
Kif22	6613	6280	9328	8127
Kif23	14823.66667	32637	14819	12188.66667
Kif24	21909	24109.5	34922	33034
Kif26a	16686	23717	25943	28556.5
Kif26b	9442	7831	11014.5	9298.5
Kif27	12591.5	39718.5	19617	38583.5
Kif2a	15289.57143	15356.57143	16981.42857	15954.71429
Kif2b	25133	26262	28407	27249
Kif2c	11998	14584	13317	16405
Kif3a	14808.5	28518	17830.5	13139
Kif3b	2255	2253.5	2578.5	2846
Kif3c	31875	15318.66667	18561.33333	20101
Kif4	26651.66667	26518.33333	27892.33333	13559.33333
Kif5a	27740.5	22394.5	29907.5	21010
Kif5b	17868	14846.5	12116.5	11287.5
Kif5c	24862	34008.66667	36864	22431
Kif6	29509	17542.5	21996.5	18176.5
Kif7	30816.5	25322.5	32031.5	32389
Kif9	2462	35602	37836	32780
Kifap3	18812	15353	15848.75	24305
Kifc1	20344	32484.5	33556.5	14479.5
Kifc2	14843	11103	16130	14424
Kifc3	6453.5	21463	10150	21576.5
Kifc5c-ps	23384	22072	25915.5	27978
Kin	15274	17577.5	20097	21150.5
Kir3dl1	40765	2543	8151	7588

Kir3dl2	39393	39292	33529	31868
Kirrel	24471.66667	23401.66667	17448	20449
Kirrel2	13361.5	11194.5	25794.5	25465
Kirrel3	18459	14497.66667	18283.66667	25234
Kiss1r	6331	2060	1283	8741
Kit	25209.5	21588.5	24501	27447.5
Kitl	26201.5	12312.5	20253.5	14346
Kiz	16620	17511	15973	15092
Kl	17222	16854	9482	10439.5
Klb	18187	18007.5	20308	23117.5
Klc2	9497	29211.5	30088.5	30575.5
Klc3	611	10268	40748	3895
Klc4	8095	37	1847	38690
Klf1	13708	13109	19091	18167
Klf10	25496	22486	37849	34027
KLF11	21882.5	18151	6138.5	24692.5
Klf12	20169.4	22144	17089.8	15569.2
Klf13	30177	26579	31036	28058
Klf14	14370	19759	16272	22882
Klf15	26988	22029	28780	25499
Klf16	2608	2386	2184	212
Klf17	15033	17079	19166	19014
Klf2	17388	10954	20204	18485.5
Klf3	6687.5	3068	20104.5	15202.5
Klf4	21134	19539	21318	19269
Klf5	15734.5	20341.5	23752.5	21479.5
Klf6	18432	14269	13953	10848
Klf7	19471.5	18558	22037.5	19312
Klf8	30950.5	25450.5	12555	9099.5
Klf9	12206	9916.5	17219	15949.5
Klhdc1	12129	11254	9844	9313
Klhdc10	18848.75	13431	23905.75	23606
Klhdc2	20127	21527.5	21934	24030
Klhdc3	1877	40276	3092	1598
Klhdc4	34437	27118	24380	24400
Klhdc7a	26395	8728.5	27719	11008
Klhdc7b	37584	724	3954	5953
Klhdc8a	35181.5	37045.5	37686.5	38958
Klhdc8b	36972	40650	34301	39321

Klhdc9	27967	32488	26782	30781
Klhl1	39030	3380	3393	4779
Klhl10	10564	4547	16663	10717
Klhl11	37917	40341	40460	4420
Klhl12	6770	9744	9159.5	12068
Klhl13	16115.5	16714.75	23873	13882.25
Klhl14	31633	31444	961	365
Klhl15	27121.25	16983.75	29436.75	27099.25
Klhl17	27899	22579	23233	22265
Klhl18	19763.5	18644	2883.5	21500.5
Klhl2	4459	7406	5703	8072
Klhl20	18440	11908.66667	14380.66667	25634.33333
Klhl21	8486	11475	5619	11211
Klhl22	1221	1934	38924	283
Klhl23	26076.33333	13815	17567	16458.33333
Klhl24	15411.33333	12247.33333	22130	23544.66667
Klhl25	24847.5	24198	21547.5	25301
Klhl26	36943	37497	39062	37692
Klhl28	27203.5	26127	9855.5	10350
Klhl29	22243	17604.5	26092	23854.5
Klhl3	10170	25729	5583	35726
Klhl30	20504	22092	27187	26317
Klhl31	20462	24538	25751	26494.5
Klhl32	9352	10848	18420	17259
Klhl35	14450	12425	15955	12421
Klhl36	10762	15647	12570	15636
Klhl38	10064	15706	14420	13057
Klhl4	28206	29582	31106	29769
Klhl40	1704	15941	8732	8518
Klhl41	36803	35049	35598	34868
Klhl42	15499.66667	12271.66667	13448	15107.66667
Klhl5	20282.5	23612	17452.5	23445
Klhl6	6192.5	3928	3088.5	19851.5
Klhl7	24097.33333	23397	19762.66667	21709.33333
Klhl8	17880	22428	15013	19886.5
Klhl9	18456	33542.33333	18482.33333	32621.33333
Klk1	10626	9745.5	15637	15258
Klk10	37665	505	1949	2565
Klk11	2812	1538	4555	1575

Klk13	38438	27825	30588	29157
Klk14	19345	24511	27890	29399
Klk15	900	2564	9199	7192
Klk1b1	26737	28152	29661	28356
Klk1b11	26063	8505	11146	27430
Klk1b16	3379	12303	1817	36606
Klk1b21	26804	29947	9372	12808
Klk1b24	7784	11210	16732	18742
Klk1b26	17681.5	32028	28367.5	27110
Klk1b27	1381	5401	5568	7060
Klk1b3	17228	16565	21697	19785
Klk1b4	6620	2463	494	38832
Klk1b5	37450	38910	140	1724
Klk1b7-ps	39127	40448	34557	28043
Klk1b8	14475	13706	17090	16005
Klk1b9	7588	8520	13589	12694
Klk4	14403	16953	20260	20940
KLK4	17399	19269	21389	15714
Klk5	37150	31217	30976	33502
Klk6	33844	36167	14027	12871
Klk7	12044.66667	12146.66667	17506.33333	17104
Klk8	36259	36285	11372	10519
Klk9	32263	35022	30656	38292
Klkb1	10944	14142	19694	22899
Klra1	3578	28601	3665	39444
Klra10	22893	17947	19187	11528
Klra12	27208	9546	30996	29657
Klra13-ps	39154	40645	8519	8414
Klra15	2383	6720	5034	5142
Klra16	25378.5	26933.5	28737	28954
Klra17	9298	30008	31533	30019
Klra18	40073	34753	8888	1854
Klra19	26355	27370	30447	29028
Klra2	31883.5	39553.5	39737	20124.5
Klra20	23897	24993	26416	1029
Klra21	5	29207	30271	17700
Klra23	31124	32009	36714	28252
Klra3	19247.66667	21731.33333	24010.66667	24234.66667
Klra5	15230.5	15668.5	24045.5	26432

Klra6	13391	17480	23046	21779
Klra7	30071.5	14409	30383	34068
Klra8	22706	16759	15395	3109
Klra9	33539	20901	20280	8099
Klrb1	27699	4191	1636	7141
Klrb1a	1328	10378	32554	37438
Klrb1b	25358	26615	28393	27789
Klrb1c	30447.5	36803.5	36269.5	34770.5
Klrb1f	28702	29241.33333	20097	29492
Klrc1	27071.5	28782.5	32921	15644
Klrc2	25761.25	27039.25	29533	19213.25
Klrd1	2272	26902	37655	4948
Klre1	19195	21053.5	21250	21647
Klrg1	2685	13577	14415	8655
Klrg2	40135	3243	7064	6667
Klri2	35899.5	32480	33445.5	32409.5
Klrk1	27340	30340.5	36995.5	33895.5
Kmo	33219	35410	13442	12520
Kmt2a	21739	20055	23774	21744
Kmt2b	15849	15468	14897.33333	14172.66667
Kmt2c	24907.2	15772.2	18510.6	17044
Kmt2d	29137	29467	28651	23965
Kmt2e	22886.25	23506.75	19341.75	18753
Kndc1	12472	23795.25	25534.5	17819
Kng1	31925.5	33825	34788	32999.5
Kng2	1753	34227	34465	28994
Knop1	11268.33333	19503.66667	11347.66667	7388.333333
Knstrn	33992	29826	28208	30032
Kntc1	11047	3120	4189	20061
Kpna1	19246	19766	17601	19801
Kpna2	29548.5	11288.5	10831	12915.5
Kpna3	11881	17495.66667	22989.33333	31655.33333
Kpna4	13819	24803	23884.25	28668.25
Kpna6	20959.5	19443.5	21071	23655
Kpnb1	15044	14177.5	16873	16544
Kprp	39196	38571	40858	36926
Kptn	11422.5	12070.5	10018	9234
Kras	13428.8	9777.4	18055.4	8899.6
Krba1	26146.5	20594.5	13067.5	23629

Krcc1	7040.5	9328	28616	8827.5
Kremen1	7197	11315.5	9190	11238.5
Kremen2	1377	4892	11510	11027
Kri1	17840	18221	19309	20858
Krit1	27407	21135.75	24666.75	25082.25
Krr1	31731	14930	21342.5	15752.5
Krt1	7568.5	10664	11786	11540
Krt10	29589	9151.5	8922.5	26523.5
Krt12	25699.5	26956.5	29908.5	34169
Krt13	30423	15086	19528.5	13402.5
Krt14	18377	21247	23169	24125
Krt15	23150	22220	30938	24567
Krt16	6761	11365	16026	15612
Krt17	18481	19092	23082	23388
Krt18	4878	40062	7592	677
Krt19	32626	14217	36860	22117
Krt2	13049	13050	13887	15056
Krt20	38598	32287	32845	32754
Krt222	36822	39976	38261	39087
Krt23	4146	3634	5369	2363
Krt25	40987	10328	36551	36989
Krt26	23431	36323	26078	38465
Krt27	10727	7749	6693	10561
Krt28	4913	39711	11201	7409
Krt31	23789	36208	2263	96
Krt32	13675	18575	20841	21176
Krt33a	38907	35974	31421	34016
KRT33A	38281	24681.5	11381	10231
Krt34	40260	36501	5346	1205
Krt35	15907	17265	33882	32807
Krt36	11094	15273.5	16534.5	20577.5
Krt4	10984	11912	6407	9560
Krt42	26076	29247	37533	28357
Krt5	9432	13351	12016	13469
Krt6a	8204	6244	8871	10526
Krt6b	38447	2405	1138	2916
Krt7	11054.5	26235.5	12320	11919.5
Krt71	17692	17201	21207	22016
Krt75	7458	12541	13877	15327

Krt76	3371	14403	4101	11156
Krt78	30031	34638	33045	31514
Krt79	9473	9079	1154	10667
Krt8	18038	15969.33333	19591	17435.66667
Krt80	13214	6800	14014	11806
Krt82	40062	38850	33131	3205
Krt83	6403	2443	10370	10864
Krt84	12256	16792	16902	19363
Krt85	14078	16578	21796	20102
Krt86	32219	34669.5	6134.5	7048.5
Krt9	40069	212	796	2161
Krtap12-1	23190	24257	1438	2450
Krtap13	16525	16868	25960	28858
Krtap13-1	27918	35045	1049	39951
Krtap14	27109	34081	39189	22013
Krtap1-5	680	4132	7934	9997
Krtap16-1	2077	4996	4134	9458
Krtap16-10b	13316	8385	23043	18717
Krtap16-2	19173.5	3256.5	4853.5	1413.5
Krtap16-3	16087.5	17358.5	17243.5	18892
Krtap16-4	9295	10264	10550	18452
Krtap16-5	38451	39253	2930	33035
Krtap16-7	11606	20075	22220	20820
Krtap16-8	11242	9175	18788	19354
Krtap17-1	32422	40344	5218	17367
Krtap20-2	32160	33807	35959	34119
Krtap22-2	33572	29105	32813	40192
Krtap2-4	25653	26971	4486	5674
Krtap24-1	27158	28228	35993	36650
Krtap26-1	16613	18585	26512	28540
Krtap28-13	38229	31527	33621	34246
Krtap3-1	26430.33333	27863	17659.66667	8178.666667
Krtap31-1	17448	20978.5	25942	25899
Krtap3-2	24975	26329	27195	40952
Krtap3-3	25989	27540	28311	27327
Krtap4-13	33687	35758	34228	37714
Krtap4-16	22634	18969	27185	31215
Krtap4-2	15935	16611	24844	23215
Krtap4-6	7812	9256	18732	16487

Krtap4-7	20785	22773	25016	24516
Krtap5-1	14288	16227	18714	18924
Krtap5-2	14690	17322	17907	22070
Krtap5-3	6246	10398	15442	16743
Krtap5-4	19571	22095	23413	23574
Krtap6-1	18156.75	12627.25	14998.5	18165.5
Krtap6-2	620	29671	27931	26729
Krtap6-3	19064	24308	22855	23094
Krtap7-1	22488	25043	39220	40835
Krtap8-1	10797	17578	32837	18572.5
Krtap8-2	31047	32720	34551	32765
Krtap9-1	27085	30086	31840	34990
Krtap9-3	39840	31531	1318	31247
Krtcap2	41029	853	39639	39135
Krtcap3	22707.5	19548	18825	19216.5
Krtdap	2911	21192.5	4586.5	9400
Ksr1	26456	25948	33994	33073
Kti12	1532.5	18606.5	37383	17188
Ktn1	21313	21129	8938.333333	12958
Kxd1	39673	39135	36467	36952
Ky	21682	35404.5	14949	19205
Kynu	37178.5	17767	31372	17381.5
L1cam	22660	23676	28167	29733
L1Md-Tf30	16109	35264.5	20268	18463.5
L2hgdh	12518	8707	12308	7944
L33255	26636	24343	25772	21412
L3hypdh	2497	40960	2075	34407
L3mbtl1	6296	4594	52	13140
L3mbtl2	24827.66667	23924.33333	19796.33333	20323
L3mbtl3	19570	26251.75	25830.25	18005
L3mbtl4	28464.5	24173	31970.5	13937
l7Rn6	17450	22580	24663	26135
Lacc1	35257	2031	23987	33444
Lace1	20847	19497	19313	21191
Lactb	11328	14967	4698	10193
Lactb2	21682.5	25498	23872	24785
Lad1	10870	9158	11450	7963
Lag3	38178	30965	32592	34502
Lage3	14703	16328	13224	12114

Lair1	5547.666667	4123.333333	23494	33877.66667
Lalba	1707	28225	21419	21144
Lama1	34759	22526	36713.5	20880
Lama2	16483	16995.75	19933.5	17217.25
Lama3	12826	9725.5	20145.5	19133
Lama4	12841.5	9102.75	14510.5	22479.5
Lama5	12885	6001	16445	14771
Lamb1	11200	7194	19101	27222
Lamb2	25465	22397	32240	30264
Lamb3	122	35767	38547	38032
Lamc1	22388	22022	26777	31054
Lamc2	12643	18288.5	34078.5	33581
Lamc3	28611.33333	22876	17578.66667	17049.66667
Lamp1	10338	2439	10718	6402
Lamp2	19186.66667	12600.33333	14958.66667	25116.66667
Lamp3	28746.25	32104.5	19188.5	25790.75
Lamp5	40184	33511	37643	12128
Lamtor1	32269.5	27419	29232	25052
Lamtor2	18686	22810	25911	25998
Lamtor3	37605	35257	34179	35567
Lamtor4	18519	20498	20811	20840
Lamtor5	8769	14007	11682	12806
Lancl1	24487	26528.5	28763.5	29411.5
Lancl2	19027	25604	23372.5	27558
Lancl3	4906	38490	1493	40766
Lao1	39679	3692	10508	15370
Lap3	24340	29123.66667	26482.66667	27521
Laptm4a	23611.5	20679.5	25205.5	21726
Laptm4b	21707.5	5344.5	6913	7518.5
Laptm5	24639	7872.5	9741.5	38302.5
Large	14702.4	20103	10847.2	14286.6
Larp1	23628.2	26362.2	21796	24243.4
Larp1b	11135	37579.33333	13255	10568.66667
Larp4	15031.75	15555.25	12330.25	12808.5
Larp4b	32310	23903.5	22057.5	23119
Larp6	30571	35943	38208	38973
Larp7	18963.5	17768	19235.5	15684.5
Lars	16591.25	22903	14056.25	13354.75
Lars2	17239	36938	2422	9751

Las1l	28022.5	27530	22193.5	21965
Lasp1	14695	11986.8	14339	10584
Lat	9934	17237	11815	18703
Lat2	16154.5	12586.5	18681.5	17679.5
Lats1	17773	16051	13154.5	17288.5
Lats2	13141.5	13437.25	14707.25	14273
Lax1	38200	32269	34678	39003
Layn	20067	40594	25017	4864
Lbh	1509	37249	1879	35709
Lbp	16017	7841	16207	14866
Lbr	15497	12525	12098	9297
Lbx1	17975	19318	17021	19581
Lbx2	21705	23941	31112	28138
Lca5	15138	19778.33333	17289.33333	19186.33333
Lca5l	33758	4523	38482	9839
Lcat	3492	9492	2398	11870
Lce1a1	24634	26978	24168	27234
Lce1a2	34831	39498	37206	10424
Lce1b	22686	25090.5	30300	30373
Lce1c	19929	20185	30924	36438
Lce1d	36817	4268	16252	20009
Lce1e	9335	10686	21859	23973
Lce1f	174	1908	24270	23938
Lce1g	20291.5	25041.5	23792.5	25717
Lce1h	27814	29165	30597	36552
Lce1i	6202	8137	14745	13164
Lce1j	4895.5	7673.5	6520	7191.5
Lce1l	15633	17629	23533	24077
Lce1m	625	74	3775	4996
Lce3c	14759.5	19624	28933	29239.5
Lce3f	19618	18471	21766	23132
Lck	35823	8591	37544	6538
Lclat1	12354.5	4941	3719	2847.5
Lcmt1	19292	27184	27172	24076.66667
Lcmt2	19590	18455	16012	17403
Lcn10	2773	39070	12854	11657
Lcn11	22055	25478	143	38321
Lcn12	18283	18322	25567	28897
Lcn2	11364	13726	19872	30625

Lcn3	34664	26594	1999	11837
Lcn4	4201	6545	13943	14915
Lcn5	7527	11659	12090	15282
Lcn6	39155	39528	2534	1607
Lcn8	3035	33818	36095	39908
Lcn9	34735	17093.5	18242	20775.5
Lcor	15719.66667	23755.66667	21679	25263.33333
Lcorl	23178.8	24165.4	26112.6	25919.6
Lcp1	18876	15988.5	20227	17158.5
Lcp2	22280	29968	17657	27351
Lct	7341	2279	9308	7374
Lctl	25393	26497	28912	27697
Ldah	36349	32931	31018	28635
Ldb1	15776.5	15592.5	14511.5	10869
Ldb2	5757	14944	10619	16217
Ldb3	6951.666667	18317	13441	21105.33333
Ldha	35451.66667	34272	26034.33333	39053.33333
Ldhal6b	41020	5998	6593	7554
Ldhb	3747	36563	212	31906
Ldhc	37615	26116	34991	27250
Ldhd	25197	37220.33333	13793	30064
Ldlr	29840	36410.5	31571.5	34866.5
Ldlrad1	38324	1181	34457	32646
Ldlrad3	24290.5	22324.5	8158	5885
Ldlrad4	16025.5	16102	15210.75	13173.25
Ldlrap1	1230	31822	1052	35902
Ldoc1	11216	13545	16646	12477
Ldoc1l	35602	37455	25618	28419
Leap2	36651	38410	34525	32720
Lect1	30307	31784	9194	31065
Lect2	22558	30537	40006	2228
Lef1	21924.6	25192.2	25832.6	21239.2
Lefty1	14837	24086	20348	24548
Lefty2	22563	29780	39261	40102
Lelp1	6859	5437	26419	26321
Lemd1	10162	9325	23387	23323
Lemd2	16121	15880	19210	22653
Lemd3	25990.6	25546.6	25072.2	25715.4
Lenep	22565	23115	24383	24329

Leng1	10894.5	14502.5	12706.5	16002.5
Leng8	11953.5	7967.5	11122	13737
Leng9	11710	12444	14778.5	16242.5
Leo1	7420	6348	5845	6651
Lep	648	6077	2280	3305
Lepr	33490.66667	33514.66667	35889.66667	33693.33333
Leprot	16093	13274	14450	11468
Leprotl1	25092.33333	22167	23012	23128.33333
Letm1	1891	37685	8361	3816
Letm2	27782.5	21284	20788	16684.5
Letmd1	1974	35274	37124	31016
Lexm	25130	34118	38112	38437
Lfng	25413.5	11962.5	17478.5	21484.5
Lgals1	7060	2176	10113	4322
Lgals12	27180	28846	29210	28001
Lgals2	34600	35496	38757	35648
Lgals3	7172	39942	10951	10707
Lgals3bp	21391	1683	21497	3434
Lgals4	12062.5	24604	10153	24989
Lgals6	16941	10753	17538	14078
Lgals7	1128	1322	11118	14606
Lgals8	18187	18460.5	17323	23144
Lgals9	39345	3025	24836	40352
Lgalsl	11571	10734	22416	20388
Lgi1	26813.2	22844.8	32548.8	34558.2
Lgi2	17457.66667	29603.66667	25171.33333	30861
Lgi3	37148	37606	3761	4753
Lgi4	4371	23229.5	5180.5	23037
Lgmn	9954	36623	10645	37495
Lgr4	20423.75	26908.75	31002.75	18830.25
Lgr5	27156	27294.5	28243	32519.5
Lgr6	10173	28599	12593.5	17748
Lgsn	8628	8744	19375	15882
Lhb	30256	29309	1240	39973
Lhcgr	27449	28815	33976.5	36239
Lhfp	12949	17488.5	16521.5	16572
Lhfpl1	33940	26640	35566	27750
Lhfpl2	6776	2842	2757	4780
Lhfpl3	28572.5	31684	31176.5	17933

Lhfpl4	12123.5	13097	15172	15636.5
Lhfpl5	36110	35251	14	486
Lhpp	23405	24700	21434	22715
Lhx1	16495.5	19187	27091.5	20867.5
Lhx2	1904	7110	22646	18434
Lhx3	38671	37561	1746	39585
Lhx4	5022	8353	8788	9723
Lhx5	27294	36347	30499	36588
Lhx6	29551.5	13255.5	13840.5	17797.5
Lhx8	27237.5	15675	8108.5	21046.5
Lhx9	26534.33333	17007.66667	18954	21795.66667
Lias	23601	32092.5	27853.5	33575
Lif	16594	18131	20327	20773
Lifr	20339.2	19182.8	25149.8	18602.4
Lig1	26102	20086	24040	20640
Lig3	14167.5	15412.5	11521	14994.5
Lig4	39759	21606.5	20098	23786
Lilra5	20570	11060	23145	16660
Lilra6	10494	9997.5	11918	14571
Lilrb3	16538	17471.66667	16738.33333	19330.33333
Lilrb4a	18743	20852	18456	24036
Lim2	15912	16388	10501	20143
Lima1	26710	17205.66667	23715.66667	16808.66667
Limch1	19939.33333	19199.33333	19660.33333	20856.33333
Limd1	18114	15673.5	13117.5	20210
Limd2	19029.5	29184.5	22409	22580.5
Lime1	16642	15663	18638	18039
Limk1	34804	31198	34085	36382
Limk2	27125.66667	25117	28687.66667	30733.66667
Lims1	28917.66667	28330.66667	25521	15813.33333
Lims2	9550	8007	16928	15806
Lin28a	33503	26343	28432	27414
Lin28b	25329.5	18335.25	16161.5	27455.25
Lin37	7312	8559	7729	8355
Lin52	29213	32512	30924.5	31691.5
Lin54	7795	2907	19448	6739.5
Lin7a	29082.6	17962.2	24577.8	20075
Lin7b	29622	26175	25881	27800
Lin7c	34069	11585.33333	10808	11981

Lin9	32243.66667	20066.66667	18014.66667	19911.66667
Lingo1	7409	9671	15089	16719
Lingo2	3037	5877	4530	6312
Lingo3	19074	19103	38049	36058
Lingo4	4899	10473	11353	11824
Lins1	15029	39054	35787.5	39193.5
Lipa	32456	17863	22763.5	19904.5
Lipc	4796	35649	2188	9660
Lipe	13025.33333	13014	14883.33333	15456.66667
Lipf	4848	8295	10174	9613
Lipg	11346	8605	9979	14379
Liph	15555.5	22659	36524.5	21534
Lipi	26361	27806	29161	28174
Lipk	29454	31024	33503	31769
Lipm	29809	402	29175	1088
Lipn	10651	11190	11242	4792
Lipo1	34949	39382	30388	34070
Lipt1	17998.5	20502.5	23442.5	22522
Lipt2	11445	10368	7705	5320
Litaf	15279	9804.5	9616.5	9089
Lix1	21865.33333	34110.66667	27267.33333	26615.33333
Lix1l	21987	28947	26632	32808
Lkaaear1	22756	25488	37463	39372
Llgl1	29088	14705	29208.5	12051.5
Llgl2	14783	14795	16181	19461
Llph	6167	11061	10841	11248
Lman1	8070.666667	19379.33333	16055.33333	20803.33333
Lman1l	22360	19988	30694.5	31187.5
Lman2	11738	7829	2318	335
Lman2l	11859	8497	8869	6906
Lmbr1	28014.8	27491.6	27795.4	28639.2
Lmbr1l	9380	8253	10261	10057
Lmbrd1	24375	14678	22519.33333	16469.33333
Lmbrd2	16120	13720	13271	13221
Lmcd1	36100	40531	27986	36304
Lmf1	26763	24761	21698	22175
Lmf2	4612	432	731	39276
Lmln	4218	22299	21269	20100
Lmna	20036	24428.33333	22460	16570.66667

Lmnb1	30568	24348	27744	23879
Lmnb2	11537.5	10476	9588	7479
Lmntd1	6750	35500	3036	6165
Lmntd2	3976	1916	7960	7789
Lmo1	19442	17231	9592	10299
Lmo2	35809	31404	1473	26069
Lmo3	5965.5	6756	9644.5	9438.5
Lmo4	8880	27976.66667	20682	25390.33333
Lmo7	20946.4	16350.4	19202.2	21106.8
Lmod1	20152	22416	28850	31809
Lmod2	31757	33608	37067	35974
Lmod3	12284	12961	14530	13717
Lmtk2	19135	13177.5	20111	22579
Lmx1a	33085	35179	35983	33968
Lmx1b	25970	21019	36488	36861
Lnp	18497	25568	20849.33333	24621.66667
Lnpep	18100.75	16444.5	17589	8376.75
Lnx1	20447	20375.5	23593.25	24285.5
Lnx2	10453.33333	20748.33333	12238.66667	21746
LOC100043315	4069	7069	9129	7538
LOC100044115	20245	19530	25261	25892
LOC100044660	32130	38031	35470	6231
LOC100047284	29198	30670	32219	30686
LOC100047363	7034	10169	9048	9771
LOC193676	22099	28325	39547	2185
LOC194586	14931	12755	6915	11541
LOC194612	1049	10968	13137	6853
LOC195290	3431	6195	21	39249
LOC195806	18062	21051	21261	24431
LOC209550	37469	3972	11627	21194
LOC210619	5784	5777	11716	11885
LOC211331	11043	12933	17797	14646
LOC211430	28862	30766	36704	29655
LOC212970	16413.5	17434.5	22674.5	22418
LOC213481	32495	34172	37722	38788
LOC215208	28646	30269	1803	30363
LOC217397	22377	22918	25660	32591
LOC218695	16995	20049	22439	23791
LOC226871	15096	16518	20487	19450

LOC227109	10202	11893	11713	13106
LOC227114	23401	38018	28285	33031
LOC227485	30483	32500	32803	31261
LOC228893	21721	22492	25367	24954
LOC229005	13224	11522	10820	8271
LOC232875	23919.66667	20862.33333	9603.333333	9388
LOC233005	33717	38182	31468	32923
LOC234097	32059	33340	36686	34658
LOC234413	5877	40533	38895	36904
LOC234907	22294	5653	25226	25379
LOC235580	9700	12179	15427	15948
LOC235927	13921	21744	19962	21296
LOC236118	32644	34168	35702	37146
LOC236260	12280	13532	10857	12664
LOC236262	39388	1973	5235	10159
LOC236622	33430	41096	38184	39213
LOC236749	13979.5	23899	9546	16941.5
LOC236875	24410	25521	32197	35778
LOC237749	27603	29437	36329	37733
LOC238427	27485	28966	30024	28753
LOC238428	14891	15351.5	8247	27848
LOC238440	2988	5256	32708	28721
LOC238447	18742.33333	15675.33333	20437	21670.33333
LOC238568	40310	31846	9364	6060
LOC239017	34784	40042	37977	40171
LOC239191	1604	37077	39286	4885
LOC239564	34436	28463	24065	32918
LOC240327	26203	27741	28569	27443
LOC240498	35316	32407	27232	31957
LOC240750	8263	14428	15761	16550
LOC241074	38897	32962	9376	13466
LOC241215	20915.5	21281	21218.5	26068.5
LOC241944	26154.5	26024	17183	20855.5
LOC242025	37360	4583	4159	2498
LOC242205	491	5314	2125	4095
LOC243823	15358	17605	16615	14672
LOC243881	7437	9672	16772	18054
LOC244512	5988	2500	1481	5046
LOC245305	22719	22815.66667	32564.33333	26779.33333

LOC245376	23986	25098	26895	25956
LOC269472	2746	3922	7562	8207
LOC277923	18520	19364	27726	26501
LOC278085	8878	5647	7030	1907
LOC280640	34832	34724	6523	4807
LOC319225	3721	1204	39509	37307
LOC327957	4310	15771	38548	11071
LOC330097	39549	27609	37481	35821
LOC330294	1234	3299	1031	6709
LOC330599	34840	4907	1837	844
LOC331528	26338	39497	28147	27157
LOC333201	15858	16256	23319	23754
LOC380735	11229	8685	10788	15883
LOC380800	28683	2782	32642	31083
LOC380823	29533	31053	37923	31202
LOC380824	21086.5	13654.5	28400	26016
LOC380843	35229	37591	33363	2564
LOC380889	21348	18153	26382	27122
LOC381219	16931	9294	14672	9928
LOC381279	23024.5	27380.5	28676	7388.5
LOC381422	38886	37516	38276	1258
LOC381483	35412	24891	31224	40556
LOC381508	3897	28019.5	17654.5	26565
LOC381727	5744	31526	39654	8044
LOC381739	16453	11788	30234	3839
LOC381764	32363	34711	23590	32305
LOC381765	17522	18958	24322	24130
LOC381783	17089.33333	5116.666667	19416.33333	6813.333333
LOC381871	8072	15651	13075	14734
LOC381912	37127	6360	38570	7573
LOC382019	4807	39432	26906	27890
LOC382133	21511	33641	35946	37059
LOC382202	26254	32175	3855	800
LOC382218	19000	20329	22941	21776
LOC382225	29596	31249	33472	31687
LOC382421	20217.66667	21181.66667	20583.66667	21240
LOC382629	27712	28089	29503	28517
LOC382692	35569	23647	35390	37024
LOC382695	37161.5	22718	20634.5	20643

LOC382762	37043	36131	429	38592
LOC382810	2640	39048	6154	10985
LOC383462	38799	27309	10998	11542
LOC383745	22440	25445	25036	22982
LOC383940	27845.5	25979.5	4428.5	4085.5
LOC384017	9861	9963	18548	18215
LOC384546	28722	17838	36388	31675
LOC384556	40668	3281	5841	4106
LOC384814	23528	7877	27568	32897
LOC384953	20511	22249	24534	22232
LOC385092	21527	30509	16963	23976
LOC385110	39146	816	38640	3098
LOC385154	22705	22475	24106	21439
LOC385319	7761	6597	8988	10281
LOC385328	22674	26107	34695	31264
LOC385414	18026	19605	22993	30710
LOC385494	19110	20881	23761	21926
LOC385747	34341	39042	7980	9534
LOC386058	24613	25794	27707	26568
LOC386478	4943	8770	11077	11663
LOC386484	12273	13648	12260	11383
LOC386509	16808	9528	17475	12400
LOC386520	38925	1102	19507	6112
LOC432442	10460	9567	11258	7747
LOC432451	35647	32760	1055	6127
LOC432492	5312	33728	11780	8639
LOC432500	24039	25181	27011	27916
LOC432536	4057	12471	11024	13906
LOC432545	10232	16093	10516	18056.5
LOC432547	16789	18120.5	22923.5	21723.5
LOC432561	20984	21196	21692	17959
LOC432594	9045	9582	3801	6105
LOC432600	12524	16663	20461.5	20331
LOC432607	19404	26499	35295	38275
LOC432625	14027	11256	19806	11239
LOC432703	5519	19613	20236	22040
LOC432751	11903	23446	13837	15578
LOC432758	40920	34777	36461	40842
LOC432777	25654.5	26177.5	20355.5	20838

LOC432823	18830	19831	14389.2	17564.4
LOC432855	25622	32726	31601	35322
LOC432906	31386	26941	36289	32835
LOC432938	21174.66667	24739	25630.66667	27969
LOC432939	27238	33051.5	32096	31530.5
LOC432942	30423	32263	32974	31427
LOC432958	5782	4955	16927	14783
LOC432965	11766	12619	19508	19960
LOC432995	25389	34599	40051	40669
LOC433001	30239	31765	33256	31677
LOC433014	40264	7372	9762	10745
LOC433045	26965	26013	37095	39402
LOC433114	5502	3765	1446	3407
LOC433156	9909	36425	5312	38535
LOC433175	28759	30241	31777	30296
LOC433197	7779	8568	21802	16258
LOC433198	25971	40751	7347	7710
LOC433237	258	5110	9035	5279
LOC433269	13078	17019	17083	19420
LOC433331	36956	35954	39963	34802
LOC433347	1562	2557	32939	31459
LOC433377	27545	39224	657	5637
LOC433412	23259	27629	34290	34718
LOC433427	35811	28307	672	36763
LOC433455	510	32942	945	35546
LOC433461	25598	39355	27578	35936
LOC433494	5864	14847	22085	29275
LOC433501	10008	9484	15026	7341
LOC433503	28105	29372	32000	41093
LOC433514	31199	32812	34003	39548
LOC433539	7512	6568	8899	8537
LOC433577	32006	33643	1760	14100
LOC433583	3032	28373	16052	35645
LOC433605	28917	30330	32259	30698
LOC433631	5543	10903	5059	2919
LOC433641	795	40674	4695	5440
LOC433658	25373	26634	28337	27402
LOC433662	32019	33708	35207	34870
LOC433718	37381	37874	37590	37207

LOC433744	39090	1060	8313	6191
LOC433746	12642	14328	16197.66667	4419.666667
LOC433757	15093	8902	11488	15262
LOC433762	37865	25627	585	34917
LOC433767	7214	7647	4296	35991
LOC433810	21979	25525	24762	21877
LOC433813	9966.5	14198.5	6943	11061.5
LOC433859	29849	14814	14710	9527
LOC433944	2650	26925	28950	27783
LOC434010	165	29206	508	41121
LOC434018	24682	25805	28119	26892
LOC434030	11013	30188	4708	4117
LOC434033	39005	30495	38445	6490
LOC434044	29582	31145	21395	657
LOC434061	32697	34350	35477	33652
LOC434064	22534	25631	27988	28596
LOC434070	34468	39033	32789	38436
LOC434119	22665	16674	20773	19655
LOC434147	4064	7401	16340	15266
LOC434213	18383.5	24393.5	22376.5	19814.5
LOC434248	25028	3349	22116	36275
LOC434264	40933	11900	11540	14458
LOC434267	34253	32824	35202	852
LOC434314	9096	5171	29668	24022
LOC434326	5237	21293	7115	20746
LOC434341	6730	17541	9547	15987
LOC434369	30538	36782	31225	28500
LOC434375	37492	2571	3555	5769
LOC434417	24527	25710	39759	28493
LOC434448	14516.66667	13491	13912	26883.33333
LOC434451	22043	5542	26854	23385
LOC434460	5009	14790	13719	11931
LOC434481	36588	22278	18708	16206
LOC434495	38856	40450	39449	10
LOC434496	34434	40108	26363	34659
LOC434512	1968	8537	29803	38670
LOC434528	24917	7819	28004	1910
LOC434536	2401	8832	37234	710
LOC434586	3935	14652	12830	14261

LOC434589	9797	8052	3602	40284
LOC434597	39722	7073	9014	9361
LOC434617	20385	6558	24678	34801
LOC434629	36702	3237	37179	3461
LOC434630	11608	8166	6586	6330
LOC434637	4159	4558	39668	1852
LOC434850	13261	12165	19142	19838
LOC435171	29188	30610	32430	30860
LOC435263	32461	36260	36301	3152
LOC435333	23673.5	21053.5	28986.5	14298.5
LOC435336	37969	494	29793	32863
LOC435366	29090.5	20953	26470.5	26457.5
LOC435456	35192	39938	2444	40629
LOC435574	26744	27894	37033	36161
LOC435591	28132	38938	11159	14867
LOC435648	39523	473	36741	1764
LOC435654	26450	27680	4784	388
LOC435726	13947	22136	22270	26212
LOC435808	23983	36983	28297	40013
LOC435864	27165	1905	30593	29270
LOC435917	10101	8308	6221	7822.5
LOC436015	3005	953	1156	1037
LOC436041	14075	8088	20505	17846
LOC436089	24888	26089	30662	32798
LOC436109	22376	32089	30895	35793
LOC436124	10905	18160	15116	10527
LOC436141	39615	38853	29545	35422
LOC436153	13271	12829	13127	14406
LOC436172	38207	39798	33913	34080
LOC436480	17499	15739	19808	18834
LOC544689	5973	8912	2602	1423
LOC544702	14500	12512	13205	6867
LOC544703	22817	23661	26355	26181
LOC544720	23478	28740	31040	36092
LOC544722	36036	33571	5799	7618
LOC544725	23175	32441	27560	31099
LOC544728	9484	38469	30822	21105
LOC544730	16421	23926	28350	31646
LOC544747	29424	27444	22543	28090

LOC544758	29804	26122	16191	19799
LOC544771	2381	4801	38459	2128
LOC544820	28772	25589	27117	4938
LOC544824	5584	7924	8272	5950
LOC544888	8319	15627	15671	13214
LOC544899	7322	12681	14429	15316
LOC544905	40884	2583	3592	2631
LOC544908	25953	27325	32328	27706
LOC544909	29972	38719	30927	38464
LOC544935	1849	26666	18083	15215
LOC544936	22679	31443	29702	24955
LOC544945	32093	5419	33468	28529
LOC544958	7693	10720	5067	587
LOC544963	9064	15938.66667	5207	19721.66667
LOC544971	6302	3107	35092	26941
LOC544994	32984	4334	37111	4066
LOC545029	16811	8993	14082	7271
LOC545048	31577	32906	346	39897
LOC545085	7090	8294	8130	10418
LOC545092	1889	3402	8148	6296
LOC545100	31031	39011	37476	34609
LOC545103	34768	40418	28704	38683
LOC545136	2496.5	26704.5	21010.5	8290
LOC545182	29207	30712	32179	30692
LOC545217	21956	19271	17744	19021
LOC545224	18591	18760	26883	28463
LOC545244	8441	3410	2404	142
LOC545261	28815	30347	35454	40089
LOC545323	21712	23755.66667	24389.33333	24705.33333
LOC545325	16581	10735	13849	6789
LOC545328	34079	38984	31135	36154
LOC545329	21646	16434	20427	15284
LOC545338	11644	6511	14523	15422
LOC545368	30401	27579	6256	38667
LOC545380	13832	14624	12925	14921
LOC545391	5144	37086	7412	1342
LOC545419	4827	39052	40988	39613
LOC545442	38836	14141	8639	18008
LOC545447	4428	8476	12271	10513

LOC545466	26593	28150	29389	28101
LOC545471	38043	38125	37838	39045
LOC545490	3117	1426	36850	40166
LOC545498	13652	27399	11410	10870
LOC545502	26399	27455	30548	29140
LOC545534	28733	30222	33024	31321
LOC545537	19297	15531	9532	2674
LOC545540	4018	757	12636	9102
LOC545542	15802	27958	17482	17240
LOC545616	3319	14060	35213	1038
LOC545619	3426	5522	8720	6831
LOC545629	6229	6409	13312	14036
LOC545633	35704	39890	38901	669
LOC545641	27968	29443	30922	29572
LOC545665	32698	40787	35607	37050
LOC545706	39143	101	411	2258
LOC545707	15970	16327	15231	18117
LOC545728	26093	29208	28886	27935
LOC545732	20196	22080.5	30419.5	25258.5
LOC545748	25504	26753	1712	4376
LOC545753	8212	15021	9985	13108
LOC545768	37395	39652	29572	28406
LOC545785	28412	37473	30382	29258
LOC545787	11171	13221	12675	13355
LOC545805	24388	28856	37985	37191
LOC545812	35017.5	18254	34216	17206
LOC545837	20739	29244	27636	27331
LOC545842	39426	39523	29782	26367
LOC545843	12810	18285	18079	20574
LOC545854	30473.625	21304	25964.875	19842.75
LOC545856	28283	22484	25517	24435
LOC545910	29159	30652	34	37589
LOC545915	28151	29635	30870	37657
LOC545925	5545	7995	40886	36670
LOC545933	37699	39600	66	1428
LOC545936	37057	30121	38	30948
LOC545940	9410	14858	16476	16828
LOC545957	13277	15672	17747	15903
LOC545963	577	27009	29886	9863

LOC545964	31947	33621	35568	33757
LOC545968	16313	19636	23454	22510
LOC545986	30477	33297	40926	40665
LOC546001	4772	1983	180	38378
LOC546002	4488	39393	335	2209
LOC546017	29854	31029.5	18305	19624
LOC546020	11825.5	15096.5	17847.5	19182.5
LOC546023	23241	19429	6972	5741
LOC546026	7748	9021	6078	8309
LOC546029	21885	19933	11668	17108
LOC546052	12131	11164	30359	28935
LOC546073	8629	14960	7662	14573
LOC546078	39364	27951	21440	20894
LOC546097	31925	29658	879	721
LOC546132	21147	19511	21185	23057
LOC546136	25832	27170	28707	27621
LOC546143	23579	29587	25356	26801
LOC546149	31786	37085	36873	1839
LOC546171	11274	14578	17505	16961
LOC546201	23004	18860	14654	13065
LOC546223	38543	12539	28019	7296
LOC546224	27099	739	30956	40493
LOC546229	20869	19405	17975	19479
LOC546232	36031	29428	38567	34294
LOC546233	26228	37927	37549	38896
LOC546235	10732	6956	6208	5500
LOC546236	39641	30049	31891	30375
LOC546237	6783	7166	6324	8794
LOC546252	39695	33474	2326	31622
LOC546257	32405	37094	26529	31997
LOC546266	18286	19591	19619	19129
LOC546296	34246	37871	504	39646
LOC546341	6049	2503	1477	37333
LOC546353	25781	30395	27782	29040
LOC546415	39340	38198	40386	5642
LOC546479	34847	6624	36420	9978
LOC546502	24312	23815	32819	30326
LOC546506	39354	1536	40370	584
LOC546508	24250.5	25079.5	8998.5	8506.5

LOC546674	7364	13091	6299	8017
LOC546692	38373	39952	1402	1347
LOC546762	10567	11044	14257.5	13566
LOC546796	26738	27910	36127	39225
LOC547005	3554	8696	5157.5	20580.5
LOC547021	9486	4919	1366	11719
LOC547122	14056	13038	9423	11473
LOC547167	9910	1809	1043	35357
LOC547242	16580	6643	12384	6483
LOC547246	20476	12035	21424	17064
LOC547308	8622.5	13414	19149.5	18267
LOC547320	17169	14250	13655	15070
LOC547327	34085	1308	6176	2127
LOC547372	12978	17940	9342	20706
LOC547403	30122	24303	25410	27241
LOC547422	27482	28700	39727	38223
LOC552877	27449	28612	30969	29608
LOC552903	38252	21774	35514	39336
LOC552906	37358	21430	24724	31116
LOC552912	19752	15909	4857	10575
LOC553090	37547	22968	27129	31380
LOC553138	1021	23549	22155	8156
LOC554327	33776	4447	32995	31267
LOC574530	8817	38783	4986	14106
LOC619994	39944	14961	9540	6161
LOC620306	27740	27031	36194	28314
LOC620382	19401	17465	18338	12285
LOC620538	4541	4584	9325	6412
LOC621184	3812	32057	32990	36237
LOC621699	15593	19857	22716	21842
LOC622116	1619	33319	39431	32126
LOC622129	6086	14140	8920	10905
LOC622175	39893	30762	31919	26554
LOC623046	32332	34108	35231	33405
LOC623346	32382	34255	35614	34167
LOC624219	733	2639	7083	12000
LOC624549	27435.5	28818	30940.5	14184
LOC625068	29243	30765	1359	30314
LOC625334	17189	22546	23417	23800

LOC625603	5065	6999	10302	5920
LOC626082	29577	31232	33107	34708
LOC626410	29444	30995	31950	30532
LOC627777	30259	31764	33739	32091
LOC627905	21662	26373.66667	19708.33333	24505.33333
LOC628062	23980	25173	26727	25616
LOC628798	23887	2426	26588	25481
LOC628856	21079	22544	22574	32751
LOC628926	26631	25594	28097	40745
LOC629091	15639	10611	26212	21375
LOC629147	29274	30784	33377	31688
LOC629206	4494	13949	19600	20908
LOC629441	35684	28288	29476	30322
LOC629826	24569	2524	4074	38345
LOC632687	24676	20358	32140	26750
LOC638575	6164	9581	14664	15058
LOC640610	30725	31977	975	34145
LOC640627	768	3805	6655	6601
LOC664628	5889	24720	28502	21385
LOC665037	27934	29251	32007	30487
LOC665043	8627	14703	11669	13046
LOC665123	32407	21520	20663	21626
LOC665189	6336	39536	26597	33061
LOC665374	40499	28731	31208	29713
LOC665384	27613	29103	9243	9482
LOC665393	14630.5	16715	29770	29530
LOC665460	32789	34249	35766	33880
LOC665732	13438	6890	8484	7763
LOC666013	13482.5	16697.5	18594	21117.5
LOC666589	24573	25709	27825	26626
LOC666625	27890	29735	29480	28340
LOC666874	4021	4804	34641	4689
LOC667086	19952	12058	29779	20148.66667
LOC667433	39736	11908	2140	39793
LOC667547	24843	24808	36391	37014
LOC667772	27299	28685	30526	29066
LOC667931	27624	29099	37217	1410
LOC668012	30742	32374	38576	32763
LOC668138	14830	16671	13467	11333

LOC668175	30194	29530	22895	24682
LOC668212	23437	6920	15961	4771
LOC668237	10492	5698	5906	7357
LOC668334	36405	31913	33318	32196
LOC668421	8123	7536	6937	6197
LOC668423	29126.5	33903.5	31710.5	32016.5
LOC668517	10159	25070	13919	15594
LOC668549	13616	20940	17229	18993
LOC668701	19592	26217	14061	13567
LOC668833	10295.5	7282.5	23922.5	20763.5
LOC671976	32553	36301	2447	4987
LOC673315	37476	22621	36211	2019
LOC674618	24048	900	40403	36533
LOC675894	9507	16149	14482	13410
Lonp1	891	39507	39972	644
Lonp2	13187.5	12492.5	29806.5	12224
Lonrf1	40768	33804	40240	40792
Lonrf2	4658	5248	5690	14846
Lonrf3	35938.5	28661	30641	28151
Lor	34980	37177	6200	5222
Lox	25473	23079	34283	31619
Loxhd1	15784	14902.5	20000.5	16957.5
Loxl1	26419	22667	35224	1584
Loxl2	17053.66667	19936	4012	10489.33333
Loxl3	1486	23306	38050	18512
Loxl4	33955.5	20280.5	19525	24988.5
Lpar1	32026.5	3807	13491	9736
Lpar2	36836	28255	3396	39077
Lpar3	5980	8877	9695	8937
Lpar4	23841	27399.5	26715.5	25691
Lpar5	12521	14519	17147	17159
Lpar6	15754	25251.5	11260.5	18040.5
Lpcat1	10836	5270	1733	38231
Lpcat2	17258	12754	15749	5228
Lpcat2b	19854	19555	38921	36055
Lpcat3	25320	25778	21084	21593
Lpgat1	11881.66667	29710	27874.33333	25079
Lpin1	19741.75	10693	15754.25	7544
Lpin2	16326	24841.6	19581.6	22374.4

Lpin3	2941	2940	8591	6506
Lpl	5878.5	36526.5	8244	23530
Lpo	8709	13894	17664	18060
Lpp	17617.42857	15588.71429	20169.28571	22678.14286
Lppr1	25700	27039	29111.33333	27693.33333
Lppr2	13357	18125	19203	23928
Lppr3	39025	24620	27926	18758
Lppr4	30991.5	21801	36962.5	3274.5
Lppr5	21796	22183.5	26404	28241.5
Lpxn	15196	21803	14649	23853
Lrat	30517	32496.5	33793.5	32995
Lrba	21930.85714	19570.14286	21699.28571	20522.85714
Lrch1	20106	21135.25	22036	25618.75
Lrch2	27660	29159.66667	30859.33333	30133.66667
Lrch3	23750.66667	26980.33333	15649.33333	25862.33333
Lrch4	19290.5	16881.5	18577.5	16121
Lrfn1	20247	30801	20489	18599
Lrfn2	31759	32939	37255	3516
Lrfn3	21825	19673	38116	30282
Lrfn4	22583	22832	32847	33462
Lrfn5	27917.33333	27201	22085.66667	20446
Lrg1	16628	17757	1416	39783
Lrguk	18413	12220	18974	19151
Lrif1	39088.5	34118.5	31893	35613
Lrig1	35052	23605	30	1534
Lrig2	18625	17092	19356.33333	8211
Lrig3	13760	12159	16706	16358
Lrit1	4845	6286	11048	11263
Lrit2	3903	2333	2624	31027
Lrmp	2713	32355	515	22109
Lrp1	21413.5	8560	18333	13021.5
Lrp10	34023	28647	26105	25305
Lrp11	79	10884	40518	10132
Lrp12	29723	23461	31227	24978
Lrp1b	25904	36937	20657	23579
Lrp2	25355.4	17559.4	19044	19202
Lrp2bp	31237.5	32878	34455.5	35948
Lrp3	9669	14268	17678	21224
Lrp4	34217	24305.5	27792	26602

Lrp5	26077	22015.5	28172.5	21897.5
Lrp6	18455	12452	14312	10497
Lrp8	24215.5	13819	25926	31065.5
Lrpap1	11759.5	15257.5	10951.5	16801
Lrpprc	26294.66667	27272	22774.33333	25149.33333
Lrr1	20401	19772	29713	23432
Lrrc1	19607.66667	18143.66667	16249	17824
Lrrc10	15787	14637	23554	24199
Lrrc14	14490.5	13539	10591	9010.5
Lrrc15	29788	35660.5	17026.5	17928.5
Lrrc16a	21762	24637.66667	25667.66667	28449.33333
Lrrc16b	40670	37655	1596	1484
Lrrc17	19239	18095	14430	10088
Lrrc18	23251.66667	21364.66667	27154	24453.66667
Lrrc19	35718	32461	6260	378
Lrrc2	6187	30774	8936	34983
Lrrc20	37513	32001	27772	26103
Lrrc23	9294	13349	15689	18852
Lrrc24	3718.666667	16414.33333	14711.33333	5427.333333
Lrrc25	1390	36104	37674	26842
Lrrc26	28444	26879	27453	33130
Lrrc27	39734	29064	35209	23466
Lrrc28	18211	16840.75	16137	18788.75
Lrrc29	17721.66667	12756.33333	24952.33333	11064.66667
Lrrc3	17576.5	19968.5	27949.5	31823.5
Lrrc31	31336	41055	26314	25312
Lrrc34	16709	17218	29510	39870
Lrrc36	7402	39578	913	4924
Lrrc37a	25454	26741	28444	27305
Lrrc38	16955	17493	19853	20073
Lrrc39	23886.2	19327.6	14739	6137.2
Lrrc3b	1872	38797	6333	5650
Lrrc4	19655	16787	19660	18884
Lrrc40	31128.66667	19159	30534	33854.66667
Lrrc41	7967	10830	14021	13679
Lrrc42	24542	7019.5	25576.5	6951.5
Lrrc43	37160	34467	5127	3845
Lrrc45	22638	19874	21568	19619
Lrrc46	19755	39206.5	5067	5021.5

Lrrc47	12677	11336	11630	10470
Lrrc48	32846	34698	36632	34437
Lrrc49	27549	21894	22981	19587
Lrrc4b	39310	39034	40046	34585
Lrrc4c	30384.5	17612	33156.5	36534.5
Lrrc51	35358	36099.5	40292.5	40784.5
Lrrc52	31021.5	32821	20669	23951.5
Lrrc55	29324	2626	38894	15731
Lrrc56	1821	32045	39895	34164
Lrrc57	22946.5	22725.5	19897	19893.5
Lrrc58	11925	11959.66667	8028.333333	12345.66667
Lrrc59	639	4366	2435	7722
Lrrc6	7690	15452	11270	19525
Lrrc61	9195	9646.5	11419.5	28265
Lrrc63	24387	40320	31255	36060
Lrrc66	10931	11038	19432	19190
Lrrc69	6081	6913	16492	11645
Lrrc7	16762.66667	22292	26623.33333	21955.33333
Lrrc71	15840	19026.5	19931.5	16552
Lrrc72	37737	16719	36968	9921
Lrrc73	9837	13866	17262	15783
Lrrc74b	17952.5	19994	23557	22475.5
Lrrc75a	18089	6721	18526	3223
Lrrc75b	34582	16431	29905	7265
Lrrc8a	16513	13615	17777	17755
Lrrc8b	24785.5	3693.5	31025.5	36629.5
Lrrc8c	33914	2702	29657	380
Lrrc8d	594	2231	2185	4325
Lrrc8e	18854	10334	19782.66667	8288
Lrrc9	7245	10295	18918	11428
Lrrcc1	11050.33333	22158.66667	20916.66667	20926.66667
Lrrd1	39863	26561	31023	33201
Lrrfip1	20313.66667	27912	15583.33333	23719.66667
Lrrfip2	12800.5	16139	14276.5	18352.5
Lrriq1	29854	32733.5	36051.5	31934
Lrriq3	18196	15058	24625	20427.5
Lrriq4	14243	14289	24116	27314
Lrrk1	20366.33333	15013.33333	12773	14184
Lrrk2	20533	25681.5	20902	24298.5

Lrrn1	29248.5	31847	36863	33331
Lrrn2	27940	25617	19247	16571
Lrrn3	35863.5	17361.5	20106.5	22550.5
Lrrn4	26834	14216	17270	12858
Lrrn4cl	34397	33940	273	38965
Lrrtm1	10354	13899	15861	11795
Lrrtm2	32895.5	32750.5	33148	31641
Lrrtm3	35007.5	33462.5	35664.5	18926.5
Lrrtm4	27051.5	28418	29904	17447
Lrsam1	33482	31512	26045	24807
Lrtm1	17564	21000.5	20424.5	3378.5
Lrtm2	39857	2226	7094	7008
Lrwd1	6184	5688	3859	5477
Lsamp	18236.75	30900.75	20696.75	15165.25
Lsg1	6275	4614	2226	3298
Lsm1	35674	1313	3811	1738
Lsm10	11297	12753	14860	11204
Lsm11	32554	37892	37744	38133
Lsm12	16580	15948	12772	12502
Lsm14a	21121	20981	23076	22091
Lsm14b	5018.5	17273	2215.5	4920.5
Lsm2	17306.5	19324	13823	14573.5
Lsm3	29157.5	35006	37740	19746.5
Lsm4	15522	18633	19315	20316
Lsm5	10281	12809	12363	11237
Lsm6	18983.2	16137.6	16188.4	14607.6
Lsm7	9023.5	10838.5	9979.5	10987.5
Lsm8	31168	40910	33903	33810
Lsp1	40256	14616	36298	9381
Lsr	27412	16924	29868	18502
Lss	5751	8800	12267.66667	15794
Lst1	9985.5	24373	14175.5	18022
Lta	5809	11752	4968	14101
Lta4h	34368	30992	24629	24327
Ltb	9714	13031	4218	6586
Ltb4r1	10277	3199	7264	4499
Ltb4r2	36298	34074	22726	23876
Ltbp1	9134	12210.33333	16444.66667	21788
Ltbp2	27882.5	35472	15352.5	2580

Ltbp3	23724.66667	20600.66667	21357.66667	32362
Ltbp4	37863	2186	3637	4567
Ltbr	6653	4711	4715	3954
Ltc4s	23088	3018	17331	1755
Ltf	14803.5	4410.5	5206	33947
Ltk	10991	10439	17064	22711
Ltn1	10257	7686.333333	6898	8034
Ltv1	14507	19268	15021	18192
Luc7l	21243.5	20462	15759	17791
Luc7l2	14471.5	15660.83333	15830.5	17700
Luc7l3	18672.5	18880	18811	18493.5
Lum	16447	16859	30826	28722
Lurap1	21497	340	34754	2857
Lurap1l	5338.5	27136	12359.5	30000
Luzp1	39602	35425	33791	32343
Luzp2	28905	34195	34001	21786
Lvrn	35610	32789	34366	32979
Lxn	25197	24976.5	21594.5	21426
Ly6a	3753	38438	9569	35058
Ly6c1	16045.5	14695.5	17129	22254.5
Ly6d	18211	25252	22652	24757
Ly6e	17658	25500	4141	20103
Ly6f	26036	21359	31820	19793
Ly6g5b	8793	9659	13006	11747
Ly6g5c	17580	20583	24149	24943
Ly6g6c	5470	5566	9232	10396
Ly6g6d	15726.66667	31113.33333	17580.33333	17740.33333
Ly6g6e	9806	7321	25259	16053
Ly6g6f	21692	4309	26927	28060
Ly6h	27789.5	30027	36807	35615.5
Ly6i	104	14207	5247	15347
Ly6k	26406	34997	57	41032
Ly75	21834	7547	22244	7005
Ly86	26786	11847	19848	2016
Ly9	7947	13914	8703	16716
Ly96	39699	39278	40819	37921
Lyar	16678	20859	21973	22920
Lyg1	31776	637	40136	4641
Lyl1	26543	16184	23545	13785

Lyn	14794.5	12283.5	10238	27574
Lynx1	14077	19723	21350	23322
Lypd1	10566	13131	22005	23241
Lypd2	14613	16741	19004	20533
Lypd3	40084	2185	4786	3043
Lypd4	10235	15265	25165	34479
Lypd5	24045	26232	28289	32101
Lypd6	22634	22376.5	21857	24904.5
Lypd6b	35530	25415	2726	37800
Lypd8	22939	16526.5	21177.5	22755.5
Lypla1	8600	15663	10423.66667	11024.66667
Lypla2	21944	19904	22029	21008
Lyplal1	36720	35139	4971	2052
Lyrm1	34595	727	37727	37
Lyrm2	7809	6106	1003	761
Lyrm4	15430	23706	25254	24683
Lyrm5	39688	4338	3228	4910
Lyrm7	23762.33333	25513.33333	14332	27735
Lyrm9	9346	10509	13520.5	10612.5
Lysmd1	18728	24455.66667	22831	25229.33333
Lysmd2	11931	11044	18695	20959
Lysmd3	23213	12796.5	18655.5	14133
Lysmd4	22192	28123.66667	28778	30021.66667
Lyst	7549	22300	7902.5	22568
Lyve1	11359	30193	27677	32939
Lyz1	38464	173	2334	3108
Lyz2	13578	14521	12193	12504.5
Lyzl1	33839	40843	35428	36913
Lyzl4	20812	19870	21534	22833
Lyzl6	21053.5	7736	20364	19430.5
Lzic	13672	14697	13095	14766
Lztfl1	18625.75	23205.75	20214.25	15131.75
Lztr1	19035.5	12385.5	12768.5	29763
Lzts1	17365	14024	13787	11289
Lzts2	21080	30127	22649	30117
Lzts3	21664	24260	30379	31339
M17518	11973	14327	16761	16003
M1AP	27149	25382	27993	23786
M29242	29830	31893	31646	30172

M29244	21004	24679	23369	24049
M54924	3165	3553	4048	11782
M6pr	20933.66667	29467.66667	24406	33373
Maats1	19575.33333	33309.66667	25757.33333	25934.33333
Mab21l1	37190	23752	20376.5	19381.5
Mab21l2	8411	15886	14291	17528
Mab21l3	33153.5	27452.5	17192	16490
Macc1	23214	23773	27050	39449
Macf1	12163	23628	26229	26971
Macrod1	20980.5	16263	18403	13079.5
Macrod2	14251.33333	25948.33333	17041	18053
Mad1l1	3391	2907	4752	4388
Mad2l1	33095	18234	25890.5	30970.5
Mad2l1bp	2115	1670	40751	566
Mad2l2	32108	33661	29998	30950
Madcam1	37585	4489	8454	10485
Madd	22376.33333	10812	16777	8816.666667
Maea	17068	13745.5	11956	32264
Mael	19586	21106	22989	21463
Maf	27250	7592.666667	17362.66667	5662.333333
Maf1	11958	11385	15203	10901
Mafa	2367	40576	3448	8829
Mafb	3909	23696	3913	23480
Maff	7712	27856	15055	33223
Mafg	23028.5	27907.5	15520	26939
Mafk	8424	8362	10502	10240
Mag	20048	27183.5	19217	26427.5
Magea1	30056	31594	33826	32158
Magea3	37138	26281	38789	29613
Magea4	35875	24641	26881	6238
Magea5	6798	9101	11369	9912
Magea6	909	2832	8466	10154
Magea7-ps	25448	30205	34287	35789
Magea8	3710	32651	39007	32541
Magea9-ps	3105	6004	7350	9753
Mageb1	35936	35365.5	23546	23336.5
Mageb10-ps	35836	33464	29593	35672
Mageb16	16657.5	23150.5	23514.5	22415.5
Mageb18	29921	31775	31914	30583

Mageb5	40117	538	6075	3459
Maged1	27434	23090	27672	26380
Maged2	10244	12825	8811	9532
Magee1	9009	17845	7725	13880
Magee2	31290.5	33151	21321	19107
Magef1	34614	14047	2515	6385
Mageh1	34590	26540	4680	1209
Magel2	4834	7398	11211	11655
Magi1	15849.5	17294.5	20011.75	17843.5
Magi2	26921.33333	29531	25676.33333	25800.33333
Magi3	21518.5	25996	28478.25	31732.75
Magix	27064	28388.5	31364.5	35277.5
Magoh	39726	1008	38737	39315
Magohb	69	7033	39985	4661
Magt1	21864	23394	24510.5	23872.5
Mak	6168	4112	19589	20716
Mak16	12155.5	17532	16872.5	20920
Mal	26280.5	29899.5	15417	29986
Mal2	25707	27352	27706	26654
Malat1	9861.25	27219.25	25083.5	19610.75
Mall	9596	7157	12018	18691
Malsu1	4271.333333	5649.333333	17099.33333	6317.666667
Malt1	13375	10191.5	28920.5	13411
Mamdc2	18691	32800	16118	31862
Mamdc4	35640	34078	38772	33012
Maml1	4280	38092	40627	34936
Maml2	31001	21632	27326	17844
Maml3	23411.5	20033	23307.5	21554
Mamld1	22288	15379	18368.5	15321.5
Mamstr	35968	15648.5	36397	22760.5
Man1a	23315.5	21747.25	24346.75	21606.75
Man1a2	4794.25	11387.25	16992.5	18257.5
Man1b1	26389	21189	20252	20369
Man1c1	11534	40920	4626	27034
Man2a1	27811.5	20537.25	20457.25	17761
Man2a2	11604.5	14791.5	23088.5	21975.25
Man2b1	17477	19478	28821.66667	22031
Man2b2	23130	21723	25959	23918
Man2c1	12548	12709.5	12082.5	12121

Manba	17831	29102	30149	26579.66667
Manbal	36948	884	40167	2045
Manea	32271	27962.33333	26539.33333	29048.66667
Manf	2533	10885	13918	17023
Mansc1	8704	8315	8908	6963
Mansc4	35374	31940	39536	39626
Maoa	28664	38658	3293	11034
Maob	40693	30982	4603	33580
Map10	15217.5	15406	15871	15412
Map1a	6091.5	19289.5	26831.75	17487.25
Map1b	21369.33333	24392	24396.33333	4084.666667
Map1lc3a	6283	5408	6754	6606
Map1lc3b	16930.5	17258.5	17493	18510
Map1s	35247	23968	27670	23123
Map2	24589.75	24318.25	36190.25	18850.5
Map2k1	40989	1011	2162	4586
Map2k2	4483	2997	194	40907
Map2k3	16592	14824	13930	12439
Map2k4	25161	25556.5	23195	6883
Map2k5	24648	19640.5	14700	18611.5
Map2k6	22171.33333	30394	20882.66667	31553.66667
Map2k7	18793	16100.5	16175	24587
Map3k1	8362	38446	4748	37266
Map3k10	22667.5	20253	17329	18001
Map3k11	14567	10896	11773	9349
Map3k12	24599	16728.33333	24805.33333	22260.33333
Map3k13	19243.33333	20682.33333	25714	24636
Map3k14	6970	11033	4944	10615
Map3k15	18041	16370	17082	14294
Map3k19	27353	35089	30618	39038
Map3k2	29543	27262	17848.75	15669.5
Map3k3	17040	13750	11383.5	8961.5
Map3k4	27319.4	27766.2	28644.4	31075.6
Map3k5	15880	15108	14507	13732
Map3k6	36077.5	35740.5	39193	19887.5
Map3k7	30121	37344	27168	35350
Map3k7cl	33807	39072	1384	37780
Map3k8	5372	40935	39741	32917
Map3k9	16845.66667	13851.66667	17323.66667	19053.66667

Map4	14713.8	25836.4	13054.2	21644
Map4k1	4232	30207	37576	21384
Map4k2	23661.5	25106	24875.5	25826.5
Map4k3	22258.2	20606.8	20289.2	21747.4
Map4k4	23983.25	23023.5	21483.5	16704.5
Map4k5	15081.75	22497.5	21570	19910.5
Map6	36717.33333	3672.333333	20343.66667	15912.66667
Map6d1	28597	30314	34734	2039
Map7	27750.4	22069.2	22667	28039.8
Map7d1	18965	15551	20096	17433
Map7d2	25715	23795	28929.5	29250
Map9	24910.5	26345	24218	24837
Mapk1	25593	21915.66667	20738.33333	21391
Mapk10	17170.25	27528.25	32783	34296.5
Mapk11	8135	17885	12451	20032
Mapk12	31566.5	15086.5	1612.5	2837.5
Mapk13	38756	36022	10075	5793
Mapk14	10036	12650	8279	10741
Mapk15	21642.5	4755	6817	6657.5
Mapk1ip1	7780	2375	474	1832
Mapk1ip1l	19560.14286	19145.28571	22054.14286	20221.42857
Mapk3	12991.5	9778	11313.5	9691
Mapk4	24379	25701	26853	25770
Mapk6	17222.33333	14566.66667	13843.66667	14004.66667
Mapk7	8509.5	5465.5	6968	24915
Mapk8	28470.2	27859.2	29317	28201.2
Mapk8ip1	21310.5	29313	31302.5	38742
Mapk8ip2	19845	22322	30894	36502
Mapk8ip3	5754.5	4878	7771.5	7965.5
Mapk9	12297.33333	14400	11501.66667	14838.66667
Mapkap1	14923.66667	14463.33333	11846.66667	14147.66667
Mapkapk2	11710.5	21771.5	5620.5	15315
Mapkapk3	39761	36607	21725	22682
Mapkapk5	22220	22829	22810	22009.5
Mapkbp1	10764	9549	13380	16130.5
Mapre1	8574.666667	18297.33333	18304.33333	18580.33333
Mapre2	29936.33333	25914.66667	17424.66667	13372.33333
Mapre3	17389	15977	14753	11905
Mapt	9948	10661	26460	17878

Marc1	17263	15372	14325	7124
Marc2	14358.75	12735	19434	10270.75
March1	24370.2	29476.6	28602.8	23990.6
March10	19835	20832	24745	24150
March11	33856	40992	36671	34442
March2	16986.66667	20651.33333	10188.66667	11517.33333
March3	21037.25	17550	15872.75	18819.25
March4	24520.5	29322	30319	30267.5
March5	19064	13837	33096.5	32969
March6	16337	19590.33333	11474	20517.66667
March7	20239.66667	14693.33333	14503.66667	15434.66667
March8	4411	40323	3553	38711
March9	15948	17159	21950	21344
Marcks	18349	11026	20443	14717
Marcksl1	38183	34492.5	36759	23539.5
Marco	14615	2212	30174	7668
Marf1	22867.75	14456.75	13278.5	14365.25
Mark1	33732.5	18517	37710	35545
Mark2	17088	16814	13237	12028
Mark3	16677.75	23390.25	20846	21414.5
Mark4	11921.5	11146	31434	30557
Mars	14448.5	17567.5	14030.5	16794.5
Mars2	10091	11460	10992	12230
Marveld1	9182	7273	3483	3705
Marveld2	24057	15060.5	15608.5	30891.5
Marveld3	19413.5	4703	24141	8637
Mas1	24878	26437.5	25539	25494.5
Masp1	17991.25	17184.25	15721.75	24056.25
Masp2	26340.33333	25633.66667	19159	14752
Mast1	38895	21762	35197	23496
Mast2	30689.5	31970.5	33035.5	13912.5
Mast3	38385	25339	30148	23630
Mast4	22315.88889	21401.11111	21956.55556	23547.77778
Mastl	28795.4	26616.2	32784.6	26482.6
Mat1a	9381	4681	12067	12475
Mat2a	20397	17052.5	20381.5	17894
Mat2b	14599.33333	9399	9666	21581.33333
Matk	21268.5	25784	34180	26887
Matn1	2146	39312	36225	36352

Matn2	555	38232	1682	2142
Matn3	16190	10786	21922	20023
Matn4	11365	14344	20439	20278
Matr3	23997.25	26879.5	26673.5	26561.75
Mau2	24731	10183.33333	9786.333333	10224.66667
Mavs	20738	20668	17151	18557
Max	12262	7898	8402	6568
Maz	12213.66667	25257.66667	13911	25989.66667
Mb	15403	12892	18419	15305
Mb21d1	21697	2308	23244.5	9081
Mb21d2	17931.5	17358.5	6642.5	28178
Mbd1	24976	22958	25720.33333	25256.66667
Mbd2	22515	20874	24950	20833.5
Mbd3	28007	26461	22488	23948
Mbd3l1	12173	15498	28595	26076
Mbd3l2	26358	27799	29307	28091
Mbd4	18362	14030	18460.5	12786.5
Mbd5	20254.5	18953.5	18844.5	13951
Mbd6	24907	20453.5	25241	22376.5
Mbip	8640	9888	9982	7962
Mbl1	1246	5406	40720	1647
Mbl2	38983	2237	8266	7859
Mblac1	7976	7893	5394	4461
Mblac2	21724.5	18723	11293	10465
Mbnl1	27998.25	21890.25	12319.25	16969.75
Mbnl2	14456.33333	20846.33333	21092.66667	22791.66667
Mbnl3	20356.66667	22863.66667	33028.33333	20212.66667
Mboat1	20951	25283	19980	21152
Mboat2	23141.5	25966.5	26968	16502
Mbp	17125.33333	14231.66667	13157	24909
Mbtd1	11462	15439.25	16480	16504.25
Mbtps1	3755.666667	4694.666667	5091.333333	4391
Mbtps2	6988.333333	28605	17856.33333	16283.33333
Mc1r	9349	9904	16424	14581
Mc2r	20190	22814	23712	23908
Mc3r	26548.5	26595.5	10369.5	11155
Mc4r	9527	13590	16790	16515
Mc5r	7729	7512	33584	29784
Mcam	23899	7868.5	9791.5	7832

Mcat	17209	18293	16066	16608
Mcc	25928	15844.5	30877.5	28574.5
Mccc1	18003	20100	34722	17387.5
Mccc2	22652.5	26269	21422.5	21672
Mcee	9524	13273	8851	11500
Mcemp1	16464.5	9286	19484.5	7137
Mcf2	24840	25924	28075	36567
Mcf2l	27172	29252.33333	25649	24070.66667
Mcfd2	17600.5	17484	16836.5	18141
Mchr1	12363	9144	9464	14605
Mcl1	28404.5	23517.5	31234.5	32608.5
Mcm10	20218	21273	22655	24803
Mcm2	38729	29878	32652	25098
Mcm3	15728	13750.5	14988.5	11570
Mcm3ap	22432	21810	22879	22125
Mcm4	34109	30713.5	34716	32400
Mcm5	4508	667	41059	39658
Mcm6	3932	39705	6526	1505
Mcm7	14037	12479	13229	11927
Mcm8	17103	15165.5	17101.5	16442.5
Mcm9	22586	26157	23653	25690
Mcmbp	22642.5	19229.5	20468.75	19607.5
Mcmdc2	26035	27162	29753	28438
Mcoln1	12140	9961.5	10344.5	9543.5
Mcoln2	31832	15137	27355	17438
Mcoln3	20834	22033.5	8671	13055
Mcph1	24717	21925	21947	20687.5
Mcpt1	22296	22657	30047	24929
Mcpt2	25832	26577	10369	8790
Mcpt4	21221.5	22409	31527	29923.5
Mcpt8	40281	37280	39829	5487
Mcpt9	29595	31163	35782	31260
Mcrs1	12983	12000.5	8746	8710
Mctp1	24665	16953.5	24593	14435
Mctp2	2726	2944	31905	33151
Mcts1	9074	9868	6651	8500
Mcts2	5601	13838	18654	22769
Mcu	28858.33333	31456.33333	27518	29559
Mcur1	7372.5	11299	25531.5	10391.5

Mdfi	4566	5296.5	26246	6967.5
Mdfic	13455.5	22831.5	30432.5	21928.5
Mdga1	36584	32916	33378	32880
Mdga2	25766.83333	31242.16667	23088	20371.66667
Mdh1	8763.333333	20935	7609	20253.66667
Mdh1b	16246.5	18374.5	14992	22249
Mdh2	39662	6654	262	7627
Mdk	22013	23245.5	5551	7168
Mdm1	32020	11704	15470.5	14681
Mdm2	15590.5	21447	20706	26264
Mdm4	13829	12464.6	13619	16943
Mdn1	25830.5	23253.75	20315.25	22872.5
Mdp1	14283	10422	9055.5	8125.5
Me1	40167.5	17916	28617.5	12893.5
Me2	12302.5	13151.5	10076.5	10436.5
Me3	11669	24217.8	19679.4	27192.6
Mea1	25858.5	30105.5	30904.5	29572
Meaf6	19465.66667	17729.33333	17746	20238.66667
Mecom	29035.5	21142.25	27738.25	18627
Mecp2	13152	14317	9486	14613
Mecr	31398	33276	27990	35891
Med1	12073.33333	11446.66667	10652.66667	8770.333333
Med10	16403	18873	18472	19234
Med11	27932	39130	38065	39900
Med12	16343	13324	16195	32718
Med12l	30060.5	17734	19271	32666
Med13	14632.83333	16809.5	18853.83333	23177.5
Med13l	23527.33333	23425.66667	17305.33333	19227
Med14	30857.5	30054.75	27658.75	28394.5
Med15	22091	15846	16477	15233
Med16	17787.5	14080	13622	12016
Med17	23940	25047.5	10025	10108.5
Med18	20174.5	28398	26625	26383
Med19	12287	14946	15523.5	16296
Med20	13214.66667	12621.66667	12760	13515.66667
Med21	39801	794	39811	2267
Med22	13006	12541.5	12462	12461
Med23	20877.66667	17406.33333	18193.33333	16039.33333
Med24	11130	8954	9948	7688

Med25	18184.33333	28343	14872.66667	13867
Med26	14515	12498	14422	14913
Med27	29775	32672	31748	14041.5
Med28	15539	16239.5	12470.5	12964.5
Med29	19050	21885	23477	25554
Med30	29389	36062	28814	28267
Med31	2993	5508	7479	8211
Med4	27108	23890	27422	28014
Med6	1425	2151	271	1550
Med7	21127	23803	24902	25122
Med8	26044	34952	32227	38141
Med9	21269	23175	22509	24563
Medag	30334	18680.5	18878.5	3514
Mef2a	10754.6	29209.6	26127.6	21368
Mef2b	19328	22292.5	7766.5	8239.5
Mef2c	22452.5	27315	34745	23142
Mef2d	16405.66667	13186.33333	18084.66667	15159.66667
Mefv	14122	659	17663	1772
Meg3	13897.2	18530.8	20782	22384.6
MEG8	29554.25	30946	32959.75	31304.5
Megf10	23306	24379	26889	39144
Megf11	16452.5	18899.5	6928	19179
Megf6	21373.5	32137.5	14566.5	14448
Megf8	17533	18175	19604	20891
Megf9	20029.5	29033	28960.5	24475
Mei1	38163	40209	7105	3217
Mei4	33541.33333	32006.66667	34611.66667	35034
Meig1	13991	21642	13675	21663
Meikin	4724	40261	5750	5139
Meiob	11925	13461	16513	16247
Meis1	8666.666667	18416	21564	30721.66667
Meis2	14509.66667	18897.33333	18406.66667	22336.66667
Meis3	11320	6931	10527	6286
Mela	23609.5	19419	26813	8325.5
Melk	16019.5	18297	22576	21966.5
Memo1	23121.5	17523	17382.16667	13555.83333
Men1	37066	38462	36135	33058
Meox1	25211.5	8216	9462	14455
Meox2	28813	32512.5	1664.5	34738

Mep1a	15235	18338	20955	21033
Mep1b	16622.5	31083.5	36062.5	37058.5
Mepce	15089.5	15937.5	16603.5	15654.5
Mepe	2224	2344	10035	8761
Mertk	33972	18404.5	27331.5	18773
Mesdc1	19848	14445.5	18347.5	13598.5
Mesdc2	16421.33333	15208.33333	16213	13916
Mesp1	21085.5	6896	9734.5	12249.5
Mesp2	5090	2828	9580	9121
Mest	26398	27823	29324	30344
Met	17866	19710	20386.5	29092.5
Metap1	24912.5	31002.5	22658.5	11760.5
Metap1d	24263	12922.25	28448.25	15819.5
Metap2	20177.66667	15643.66667	16626.16667	17372.66667
Metrn	24261.5	25326.5	19513.5	20436.5
Metrnl	25459	31984	21278.5	29976.5
Mettl1	3620	9410	7263	9244
Mettl10	13205	13273	14833.5	16470
METTL13	24419	20769	22135	22694.5
Mettl14	23787.66667	21626	23678.66667	22276
Mettl15	18854	24974	19346	21028
Mettl16	10224	9356	24665.5	22750
Mettl17	35955	30742	28032	32342
Mettl18	18922	16239	16413	14746
Mettl2	38885	40301	40058	207
Mettl20	9874.333333	16335	19443	18301.33333
Mettl21a	17121	15306	14842	14474
Mettl21c	6239	32556	3039	40174
Mettl22	37120	22443.5	38362.5	39015
Mettl23	23541	22212	19890	20730
Mettl24	39324	36839	5194	6180
Mettl25	371	2755	39263	40160
Mettl3	17532	18817	15238	17927
Mettl4	12686.5	22066	22645	19578.5
Mettl5	9511	15418.5	11819	14440
Mettl6	9738.5	12770	10578	13224
Mettl7a1	15964	11509.33333	14393.33333	16043
Mettl7a2	29964	21269	34152	25047
Mettl7b	30487	27934	37667	814

Mettl8	8822	5081	6048	3319
Mettl9	16248	16153.5	12459.5	13257.5
Mex3a	5140	4250	3987	20468
Mex3b	10382	5220	11615.66667	8012.333333
Mex3c	14736.33333	19103	21262	24022.66667
Mex3d	12281	18409	14839	15171
Mfap1a	33445	35135	39014	35998
Mfap2	8280.666667	11479.33333	11169.66667	11495.66667
Mfap3	20286	13551	9662	8886
Mfap3l	12924.75	12978	13325.5	19973
Mfap4	13974	16461	23338	22536
Mfap5	3012	40098	16146	18838
Mff	10149	14915.33333	6156	16024.66667
Mfge8	20379	10245	19170	14970
Mfhas1	8715	40748	7354	989
Mfi2	22602	26394	36443	39773
Mfn1	22121.66667	22171.66667	22129	24628.33333
Mfn2	20848	18549	24496	23016
Mfng	1507	2476	37916	40118
Mfsd1	9351.5	27165.5	27650.5	28210.5
Mfsd10	28940	23761	26206	24509
Mfsd11	10911	21784	25801	18689.5
Mfsd2a	13626	18559	19304	27807
Mfsd2b	15868	13893	40214	8288
Mfsd3	11860	10157	10264	8866
Mfsd4	21790.33333	28382.33333	26219.33333	29690
Mfsd5	9685	6682	5737	3712
Mfsd6	17165.5	23011	17046.5	24480.5
Mfsd6l	1000	4706	3170	4105
Mfsd7a	21789	7586	16355	5878
Mfsd7b	19507	15991.5	18071.5	19225.5
Mfsd7c	14282	12712	14219	11604
Mfsd8	20675	16711	15834.33333	18324.33333
Mfsd9	12552.66667	13452.33333	15623.66667	15872
mg684	30592	35788	1569	2894
Mga	12574.4	16633.6	16543.4	25125.4
Mgam	25546.5	23455.5	20820	24862.5
Mgarp	36268	3652	13950	25029
Mgat1	12183	10039.5	9483.5	27990

Mgat2	26562	27722	26786	24741
Mgat3	36113	27926.5	6824.5	22338
Mgat4a	27161.5	29200.5	12469	34479
Mgat4b	32123	26765.5	28489	28656.5
Mgat4c	30073	29358.66667	30989.66667	31207.33333
Mgat4d	26625	36971	40086	1449
Mgat5	19241	8211	15712.33333	7301.666667
Mgat5b	18114.5	20321	21883.5	21718
MGC7817	15152	18651	19497	20038
Mgea5	5432	4987	14716.5	8605.75
MGI:1930074	29157	30639	32148	30646
MGI:1931461	30232	41043	102	3883
MGI:2150387	26500	27541	32648	29080
Mgl2	37634	18241	24085	13267
Mgll	14100.25	17663.25	17661.25	20464.5
Mgme1	6111	7510	6860	6331
Mgmt	7617.333333	4012.666667	7012.333333	19801
Mgp	3202	19900	11681	2549
Mgrn1	33279	23594	29648	24902
Mgst1	26960.5	29832.5	31802	31210
Mgst2	35974	1841	21790	28775
Mgst3	8208.666667	11070.33333	9260.666667	18484.66667
Mia	25283.33333	23588.33333	25424	24396.33333
Mia2	26538	37081	29775	28503
Mia3	15096	22617.25	19941.5	18433.25
Mib1	14547	23461.75	21258	21171.5
Mib2	12425	10095	8750	5374
Mical1	21963	13543	23072	17000
Mical2	22236.5	15772.5	20332	18572.5
Mical3	28590.5	8543.25	17250	16319
Micalcl	7896.5	23341.5	25993	28988
Micall1	31180	27281.66667	13941	29995.33333
Micall2	39022	7377	1331	10873
Micu1	4396	13779.25	20917.5	14226.25
Micu2	22088	21997.5	22520	21288
Micu3	14459.66667	26563.66667	26581.66667	23528.33333
Mid1	4315	19280.66667	16732.33333	8948
Mid1ip1	16420	12394	15651	13550
Mid2	20711.66667	34609.33333	16418	22699

Midn	21575	20970	28355	27476
Mief1	20003.5	22333	24627.5	25893.5
Mief2	13796	17022	8078	14468
Mien1	15531	19834	21301	21956
Mier1	22068.42857	17993.42857	19521.85714	13936.28571
Mier2	14708	14616	13007.5	12266.5
Mier3	22333	25824	23984	25735
Mif	8271	11332	6938	16166
Mif4gd	6359	5854	7607	5606
Miip	27891	27832	25549	27570
Mill1	27374	38218	32746	31183
Mill2	23040	26618	32388	33737
Mina	33132	37074	31933	36483
Mink1	16090.5	16620	15604.5	17645.5
Minos1	8175	8869.5	22505	25481.5
Minpp1	23303.5	9887.5	26079.5	9253
Mios	14392.5	10930	9907	11021
Miox	22508	30744	39408	40324
Mip	3045	5842	11872	12760
Mipep	21900	25864	21398	22709
Mipol1	17266.6	23750.8	16371.4	22234.4
Mirg	27833	29083	31062	29726
Mis12	1023	4415	2005	3526
Mis18a	20343.5	38968	38098	28485
Mis18bp1	12961	7104.5	12344	25976
Misp	10085	16380	17301	12209
Mitd1	37872.33333	14201.33333	39138.33333	15850.66667
Mitf	26300	14720.25	15410.75	17231.75
Mixl1	7268	11359	12551	9765
Mki67	23962	21006.5	16861	15211
Mkks	11223	15076	10456	15676.5
Mkl1	11743.5	8132	10675.5	7296.5
Mkl2	23276	24431.33333	33333	35287
Mkln1	18938.6	23072.4	17759.2	21132.8
Mknk1	23650	28393	16068	22845.66667
Mknk2	35597	19790	33729	20272
Mkrn1	13241	13688.5	12901	11316
Mkrn2	11022.5	14251	9965.5	13301
Mkrn2os	5497	8950	8105	10770

Mkrn3	40865	14054	1581	5146
Mks1	11387	12191	10539	15141
Mkx	26866	31106	35703	38475
Mlana	6243	1920	6224	6521
Mlc1	23888	27157	26445	25404
Mlec	39062.5	23345	20067.5	23488.5
Mlf1	35384	39526	16198	19147
Mlf2	37284	38074	37710	35993
Mlh1	14296	13934	12030	10339
Mlh3	9779.5	22587.5	19403.5	19352
Mlip	30935	30262.5	23591.5	33468.5
Mlkl	12235	19648	14762	23798
Mllt1	15718.66667	6422.333333	4413	6130.666667
Mllt10	15671	16737	16722.5	14426
Mllt11	13862	15960	12370	17372
Mllt3	28479.33333	24846.16667	19566.33333	29926.5
Mllt4	26677	24483.75	22332.75	22302.375
Mllt6	13315.66667	18627.66667	17643.66667	21980.66667
Mlph	17705	9050	22296	13511
Mlst8	21344	19667	20173	19300
Mlx	27727.5	27085.5	24170.5	30677.5
Mlxip	27977	24862	31788	27578
Mlxipl	10360.5	14095.5	10181.5	7981.5
Mlycd	35205	30807	29047	26019
Mm.331779	14704	15246	8955	12810
Mm.60620	32288	33942	35376	33479
Mmaa	10551.5	12332.5	15107	17337.5
Mmab	30751	19354.5	31454	32559.5
Mmachc	34493	29056	29865	24200
Mmadhc	31178	38343	33987	38777
Mmd	15772.66667	15119	38065.66667	16698
Mmd2	34743	39671	38592	39297
Mme	19714	21483	24730.5	27142.5
Mmel1	38217	40425	583	2651
Mmgt1	23916.5	21166	20445	20933.5
Mmgt2	12952	9503	39652	33673
Mmp10	39721	33640	37454	38099
Mmp11	16634	21092	24682	25052
Mmp12	30201	20270	31354	35430

Mmp13	11008	39626	40591	21974
Mmp14	4821.5	30139	22028.5	25459
Mmp15	32020	33844	35619	33971
Mmp16	31916.75	31274.25	24773	28845.5
Mmp17	12698.5	13050.5	21729.5	16826.5
Mmp19	3305	35674	39472	32970
Mmp1a	11738	14919.5	18251	20645.5
Mmp2	11009	6188	9339	40866
Mmp20	27301	35735	29596	28333
Mmp21	34696	37065	2878	4085
Mmp23	33709	36035	36897	40231
Mmp24	23038	24244	24333	23978.5
Mmp25	32244	37740	39822	3686
Mmp28	20052	18255.5	1976	20083
Mmp3	18231	35479	2414	926
Mmp7	33938	38226	188	4747
Mmp8	17583	10524	23586	18376
Mmp9	20035.5	19771.5	21621	23826.5
Mmrn1	15900	30208.5	32590.5	16064
Mmrn2	33817	38429	5120	6308
Mms19	23482.6	20796.8	15680	17334
Mms22l	9856.333333	10361.33333	7739.666667	8054
Mnat1	24148.16667	17756.33333	21512.16667	23979.66667
Mnd1	19335	18323	19102	20361
Mns1	12248	13285	8339	9515
Mnt	14626	8252	12696	9605
Mnx1	10914	8792	15606	15619
Moap1	17638	19007	18015.33333	18371
Mob1a	4713.5	4448	22873.5	23349.5
Mob1b	7162	18987.66667	6642.666667	9747
Mob2	31520	22422	23455	24081.5
Mob3a	9933.666667	6545	9663.333333	20872.33333
Mob3b	15160.66667	22593.33333	18592.33333	27325.66667
Mob3c	36911	23766	27848	25079
Mob4	23100.5	4432.5	23480.5	21616.5
Mobp	25569.66667	28794.66667	19163.66667	18737
Mocos	16545	16021	12699.5	14705
Mocs1	27710.66667	23480	30639.33333	13192.66667
Mocs2	8635	12337.5	11040.5	13798

Mocs3	18023	20910	18997	19742
Mod2	24844	20801	21628	19337
Mog	30062	32758.66667	30888.33333	19046
Mogat1	25052	35708.33333	14752	14893.66667
Mogat2	2371	3007	361	10807
Mogs	15471.5	15925	13250.5	14389
Mok	15433.66667	13020	15837.33333	17285.66667
Mon1a	21139	19958	21070	20505
Mon1b	33150	18161	29520.5	38973.5
Mon2	23316.5	17629	18440	16755.5
Morc1	31824.5	27364.5	31047.5	14651
Morc2a	12552.5	13110.5	16088	16238
Morc2b	40359	1465	6209	4378
Morc3	29065.5	28812	24754	27931
Morc4	19352.33333	19705.33333	11458	12396.66667
Morf4l1	20850.16667	22028.5	29515.16667	24228.66667
Morf4l2	24777	25352	25887	26020
Morn1	26985	25691	25995	25136.5
Morn2	13640	16739	16635	18325
Morn3	25188.5	28134	13360.5	34733
Morn4	32726.5	28903	30766.5	25952
Morn5	1222	525	3603	39707
Mos	1743	28496	6869	39817
Mospd1	22571.5	23115.5	25783	25779
Mospd2	19860.5	20847.5	19421.5	19967
Mospd3	19854.5	18054	15791	13425.5
Mospd4	2624	5912	9398	7965
Mov10	6266	6206	6823	5608
Mov10l1	36084.5	37134.5	33663	33797.5
Moxd1	20922	22989	31621	34223
Moxd2	9973	12804	17240	16873
Mpc1	20433	25329.5	20127.5	23791.5
Mpc2	9642	21489	6547	23443.5
Mpdu1	5926	41085	3577	1762
Mpdz	21814	16535.5	21375	24390.5
Mpeg1	26080.5	22132.5	21366.5	23893
Mpg	10365	10880	9246	7642
Mphosph10	23414.33333	17018	23914	29887.33333
Mphosph6	26147	32567	32582	28844

Mphosph8	8333	10507	8678	10938
Mphosph9	8962.333333	20007.66667	22129	21523.66667
Mpi	40189	876	39289	239
Mpl	23830	31010	26138	28512
Mplkip	22830	22069.75	21778.75	20791
Mpnd	15070	15292	15100	17208
Mpo	25958.5	5988	11776.5	11845
Mpp1	17232.5	25830	35375.5	30474.5
Mpp2	17024	20194	20596	21552
Mpp3	29459	9986	28743	11056.5
Mpp4	23760	24844	27038	36032
Mpp5	18503	32810.5	35015.5	35715
Mpp6	10052	22327.5	7729.5	23191
Mpp7	29255	30480	22136.5	21788.25
Mppe1	36309	25360	30635.5	23575.5
Mpped1	1535	2825	9467	8807
Mpped2	2711	25980	19721	23276
Mprip	24044	23687.5	21437.5	15347
Mpst	13915	12356	6829	7443
Mpv17	35530	25422	24573	23899
Mpv17l	10497	8633	5733	4654
Mpv17l2	14326	18005	13665	16220
Mpz	36969	38896	1420	2738
Mpzl1	3073	27550	3927	34282
Mpzl2	15656.5	18222	19675	19726.5
Mpzl3	18464	21155	24110	23431
Mr1	18432	40018	30995.5	32322
Mrap	18340.5	14089.5	17309.5	13469.5
Mras	11636.5	17083.75	13960	25521.75
Mrc1	27683	19686	24016	18967
Mrc2	25431.66667	24070	16632.33333	29073
Mre11a	8905.5	8496	26380.5	8769.5
Mreg	1737	16332	4690	13386
Mrfap1	25126	23901.5	7197	3220
Mrgbp	2561	6645	7163	14017
Mrgpra1	28565	30051	4298	12064
Mrgpra2b	1191	17208	413	10368
Mrgpra3	31560	34469	38508	38045
Mrgpra4	40477	4508	3215	39062

Mrgprb1	31697	34079	34014	32366
Mrgprb2	16209	19961	18741	19204
Mrgprb3	22470	29860	36709	39851
Mrgprb5	5491	8882	23965	32351
Mrgpre	3900	38242	5431	39589
Mrgprf	27221	23979	29376	35924
Mrgprg	4621	8943	9443	11902
Mrgprh	3022	8057	16065	17532
Mri1	15901	20908	14809	12218.66667
Mrm1	20272.5	17695	16012.5	14489
Mro	35278	35193	18155	19246.5
Mroh1	19615	17669.33333	16457.66667	30285.33333
Mroh2b	28517.33333	25750	30639	24983.33333
Mroh7	38330	40879	33153	4622
Mroh8	32668.5	15690	21737.5	21094.5
Mroh9	27746	29538	29380	34586
Mrpl1	20914	12520.25	20224	22062.25
Mrpl10	22125	20962	22031	21968
Mrpl11	10565	13227	10271	9623
Mrpl12	6458	9276	7808	8577
Mrpl13	41043	4180	890	3877
Mrpl14	15838	21966	18503	20502
Mrpl15	11509.33333	16275.33333	18122.66667	17727.66667
Mrpl16	28418	36289	25695	28735
Mrpl17	28523	36115	37244	35836
Mrpl18	31362.66667	10982.66667	36316.66667	24824.33333
Mrpl19	12647	11862	10741	11836
Mrpl2	15106	20030	18731	19810
Mrpl20	15354	22113	20587	21186
Mrpl21	10661	13696	6648	9726
Mrpl22	13953	18061	18105	18629
Mrpl23	14463	19650	17628	18517
Mrpl24	38094	40838	171	1062
Mrpl27	22492	19070.5	12945	12902
Mrpl28	21191	27025.5	25860	27523
Mrpl3	17429.2	10181.8	16141.4	17526
Mrpl30	27573	10442.5	26371	23269
Mrpl32	11073	15483	12213	14027
Mrpl33	27003.5	15404.5	24286	23196

Mrpl34	33410	37034	31391	32099
Mrpl35	18077	22590	18969	23459
Mrpl36	3477	4179	40970	38772
Mrpl37	10074	12950	2271	4812
Mrpl38	13114.66667	20812	16255.33333	21577.66667
Mrpl39	25945	33949	23616	30458
Mrpl4	18333	17300	15154	14739
Mrpl40	29465.66667	20284.33333	32037.66667	33627
Mrpl41	8774.5	8362.5	24498.5	7107.5
Mrpl42	27467.5	11355	31211.5	27070
Mrpl43	8886.5	12642.5	12699	13822
Mrpl44	17280.66667	16680	16311	18148.33333
Mrpl45	32847	19435.5	28328	19822.5
Mrpl46	14348	17155	11434	13812
Mrpl47	21723	25569.33333	26095	28639.66667
Mrpl48	26473	31788	28079	35391
Mrpl49	13541.66667	13915.66667	13280.33333	13956
Mrpl50	29000.5	30060	28349	30848
Mrpl51	11167	17196	16179	18469
Mrpl52	18476.33333	24681.33333	20457.66667	22693
Mrpl53	4014	9028	5947	7492
Mrpl54	39287	5485	645	3864
Mrpl55	39341	3302	39418	41012
Mrpl57	13720	18319	20271	19859.5
Mrpl9	7450	9254	6657	8568
Mrps10	37069	2827	40427	3421
Mrps11	6542	8546	6702	8369
Mrps12	776	737	53	38936
Mrps14	26114	24551	18006	19389
Mrps15	9879	6176	5312.5	4230
Mrps16	12441	18935	16491	14814
Mrps17	27303	8197	25899.5	28078
Mrps18a	36434	39787	38695	1678
Mrps18b	25638.33333	25091	25276.33333	30456
Mrps18c	34827.5	1936	39120.5	36304.5
Mrps2	19070.33333	21975.66667	22071.66667	22868.66667
Mrps21	17493	21191	15699	14428
Mrps22	16393	19221	15629	18024
Mrps23	21012.5	22506	22335	23331.5

Mrps24	33277	38562	38959	36817
Mrps25	12078	7680.5	6695	2700.5
Mrps26	32573	33321.5	13228	33195.5
Mrps27	34765	35986	29938	33475
Mrps28	37406	6127	39785	1031
Mrps30	13127.5	13166.5	12592.5	14728
Mrps31	28516	35932	35519	36411
Mrps33	26750.66667	19050.33333	16270.33333	15494
Mrps34	6544	10525	11186	12237
Mrps35	2300	4170	40611	2983
Mrps36	30682	35678	27236	26928.5
Mrps5	17418	16019.25	16644.5	17297
Mrps6	18095	28666.66667	28319	30348
Mrps7	21122.5	27723.5	30784	32453.5
Mrps9	22150	22892	22121	23891.5
Mrrf	21500.5	22503	21442.5	22903.5
Mrs2	33239	37529	26654	35897
Mrto4	32104.5	21015.5	13566	16943
Mrvi1	20508.5	22323.5	23302	28798
Ms4a1	21704	28067	21472	19639.5
Ms4a10	16019	18016	21337	21887
Ms4a12	1320	6377	9255	10346
Ms4a13	23624	24704	26549	26025
Ms4a14	32713	20858	23640	28130
Ms4a2	13926	17106	29088	30660
Ms4a3	33517	38104	4464	10428
Ms4a4b	19713.42857	24317	23471.28571	25590.85714
Ms4a4c	5872	22396	7340.5	24070.5
Ms4a4d	26039.5	24464.5	27858	27079.5
Ms4a5	24967	27577	35549	36207
Ms4a6b	22575	36109	17668	39226
Ms4a6c	25917	2854	23178	38764
Ms4a6d	5894.5	15532	24862	17150.5
Ms4a7	13287.5	22125	16200	21485
Ms4a8a	31774	30612	39	809
Msantd1	9264	5332	9463	7667
Msantd2	16671.33333	27530	30966.66667	29533
Msantd3	17312	13566	23313	16419.66667
Msantd4	39521	663	40609	1455

Msc	31017.5	32614	34134	32426.5
Msgn1	36080	38962	3494	1760
Msh2	21259	16501.66667	17837.33333	14950.33333
Msh3	22143.75	25074.25	18725	15106.5
Msh4	30698	32262	5595	5689
Msh5	25294	28883	31309.66667	32291
Msh6	17664	17954	17473	18750
Msi1	6444	7142	10343	11195.5
Msi2	16739.16667	10723.66667	17935.33333	20283.5
Msl1	25169.75	24073.75	25261.5	23687
Msl3	17283.5	20680	20954.5	22337
Msl3l2	40880	38989	40937	37140
Msln	401	35669	6426	824
Mslnl	39363	32209	38215	37773
Msmb	7175	2979	13093	12756
Msmo1	21303	17522.5	13160.5	12310
Msn	17602.33333	23460.33333	18920.33333	26864.33333
Msr1	27148.66667	10922.33333	6460	14317.66667
Msra	35624	25080	34572	24917
Msrb1	30661	31471	30090	32268
Msrb2	16544	13798	14351	8405
Msrb3	27384.33333	19355.66667	10824	15356.66667
Mss51	9401	4121	8510	10813
Mst1	36380	40506	1546	470
Mst1r	9106	1314	11822	6898
Mstn	25888	38795	36114	35527
Msto1	28551.33333	14846.66667	15166.66667	25401.66667
Msx1	25685	28597	31922	31487
Msx2	31390	35365	39796	39342
Msx3	30904	16494	17378.5	17740
Mszf78	30999	36871	39944	34257
Mszf81	16067	16676	14506	16219
Mt1	13315	29761	10416	28444
Mt2	5484	37840	8445	897
Mt3	631	3043	2761	4247
Mt4	5900	22487.5	19940	23367.5
Mta1	16029	13413	13839	12661
Mta2	21758	23703.5	4621.5	39988
Mta3	31843.66667	30828	19026.66667	26682

Mtag2	7704	6772	18351	18410
Mtap	2034	2009	37317	39109
Mtap7d3	24629.5	31888.5	20266.5	26791
mt-Atp6	15626.5	23958	24462.5	6455.5
Mtbp	19675	22228.66667	23071.66667	13083
Mtch1	21368	19313.5	17798	17625
Mtch2	34784	14861	28293.33333	19988.66667
Mtcl1	35039.66667	25069	14368	14849.66667
mt-Co2	8080	33395	2865	2061
mt-Cytb	31320	13352	15219	15906
Mtdh	20008	21648.75	14201.25	11515
MTERF1	29139	20196.5	36250	36748
Mterf1a	26781	27964	203	38014
Mterf2	36736	8063	5875	10618
Mterf3	19874	23586	11551.5	28518.5
Mterf4	15223	15835.5	15841.5	15843.5
Mtf1	18539	19617.66667	22142.33333	22164.66667
Mtf2	19538.25	17716.5	16243.75	17242.75
Mtfmt	5425	2388	37627	37348
Mtfp1	9276	9872	8543	7968
Mtfr1	12244	10619.5	8943.5	28255.5
Mtfr1l	29062	28590	22698.5	26969
Mtfr2	7934	18667	10898	21550
Mtg1	33728	32572	24988	29035
Mtg2	9418.5	25594	23571	4617.5
Mthfd1	10015	9828.5	23447	24811
Mthfd1l	11194	22954.5	14573.5	16079.5
Mthfd2	13338	17777	9583	15346
Mthfd2l	4522	11782	22376	4979.5
Mthfr	20824.83333	27584	17636.16667	22141.5
Mthfs	26426.25	24504.25	20777.75	13822.75
Mthfsd	455	40316	1950	3658
Mtif2	8468.666667	25381	16651	16642.66667
Mtif3	6535	9368	9949	9868
Mtl5	18555.33333	13165	16560.66667	14520.33333
Mtm1	12673.5	18151.5	11387.75	15859.5
Mtmr1	17010.66667	16081	14563.33333	15173
Mtmr10	22882.33333	16606	31073	31246
Mtmr11	36825	22066	37714	19486

Mtmr12	18996.5	19573.5	15070.5	18829.5
Mtmr14	32550.5	2909.5	32311	20602
Mtmr2	35564	41037	38120	37205
Mtmr3	10653.5	27282.5	9796.5	27184
Mtmr4	29107.5	27529.5	25408.5	26968.5
Mtmr6	14034	14731	16150	14138
Mtmr7	30118.5	28826	22997.5	30373
Mtmr9	11798	13950.5	11160.5	11172.5
Mtnr1a	28644.5	30198.5	37999.5	31814.5
Mtnr1b	10832	12790	11672	12914
Mto1	21531	20626	27028	32906
Mtor	16353	20021.5	15508	17539.5
Mtpap	19637.33333	15466.66667	16554.33333	14775.66667
Mtpn	19514.25	24749.25	21369.75	26862.75
Mtr	22094.5	13930.5	15106	31472.5
Mtrf1	5640	9512	10452	10843
Mtrf1l	2974	3746	2186	3435
Mtrr	15997	30065.33333	16422.33333	19945.66667
Mtss1	27723.66667	16296	27059.33333	20559.66667
Mttp	1627	38590	8779	280
Mturn	18385.33333	19263.66667	14038	26486
Mtus1	32622.75	30350	21214.75	15222.25
Mtus2	23452.33333	24472.66667	18530.66667	23293.33333
Mtx1	24173.5	23202.5	24444	23268
Mtx2	16179.5	16591.25	20080.25	23810.75
Mtx3	32406.5	28912.5	29321	28925
Muc1	29647.5	32436	4379.5	13747.5
Muc13	30464	35663	34086	32336
Muc15	35738.5	31612.5	5143.5	6734
Muc16	9905	11500.5	14621	18181.5
Muc2	14333	18314.5	25265	27443
Muc20	29962	30328	39592	37459
Muc3	24721.5	17255.5	14192	29014
Muc4	16752.33333	19236.33333	18042	20049
Muc5b	2274	4315	8539	8624
Muc6	9536	15395	17831	18527
Mucl1	10447	12295	18051	18130
Mug1	13129	18379	22621	24823
Mug2	22151.5	26327.5	30320.5	30060.5

Mul1	7778	9188	10095	11631
Mum1	20998	24213	20454.5	21896.5
Mum1l1	2915	38373	936	8154
Mup1	31355.33333	33044.33333	31646.66667	26563
Mup2	15198	16990	20488	19903
Mup3	18897	20112	25356.5	24264
Mup5	40345	11436	13419	12592
Murc	34310	56	6268	20937
Mus81	21063	39893	37600	36425.5
Musk	7238.666667	28713.66667	20252.66667	24271
Mustn1	10389	11276	14331	8714
Mut	28320.66667	27090.33333	26441.66667	25795
Mutyh	13128.33333	11651.66667	15094.66667	9458.333333
Mvb12a	5208	11304	7186	8933
Mvb12b	24421	19587	23772	18922
Mvd	36178	6577	30327	4727
Mvk	3464	9799.5	23287	2139.5
Mvp	10866	16708	8089	12728
Mx1	15717	13886	18314.5	18160
Mx2	10508	26474	17559	40568
Mxd1	8392	10079	10799.5	11430
Mxd3	15548.5	13259	19041.5	24585
Mxd4	28062.33333	19442.33333	22505.66667	28717
Mxi1	23916.5	21285	28064.5	28153.5
Mxra7	23051	21303.5	24637.5	24023.5
Mxra8	25181	24239	29673	29853
Myadm	18725	20150.66667	20146.66667	23301.66667
Myadml2	21133.5	36313.5	40864.5	34084
Myb	12822.75	16952.5	18529	27945.25
Mybbp1a	7910	8336	8862	9324
Mybl1	28813	14820.5	28404.5	26699
Mybl2	8244	12228	11999	13184
Mybpc1	2577	980	360	7947
Mybpc2	35320	39229	39907	39434
Mybpc3	24626	27395	28037	35882
Mybph	9302.5	11956	11405.5	9411
Mybphl	10110	11999	15665	11593
Myc	9073.666667	18144.66667	15378	10649
Mycbp	11504.33333	17150	18302	21082.33333

Mycbp2	22455.875	21485.75	22369.875	23372.375
Mycbpap	19707	17077	24384	26873
Mycl	22315	23644	35337	34265
Mycn	19147	38897	19866	20076.5
Mycs	21232	20814	29601	38515
Myct1	36409	37891	1309	3677
Myd88	40318	2787	34843	40551
Mydgf	17230	23719	24314	29103
Myef2	2588	6650.5	5757.5	10238
Myeov2	15343	21360	21095	21953
Myf5	27448	28967	36824	33458
Myf6	22352	22015	38777	40306
Myg1	9754	10366	29639	9731
Myh1	30535.25	31973.5	15411	23785.25
Myh10	14519.33333	12175	20138	15304.33333
Myh11	23518	26531	32860	34845
Myh13	40776	40300	12117	8094
Myh14	26414	11755.5	12889	9466
Myh2	24188.5	25787	12055.75	20566.75
Myh3	10616	14662	19713.5	18634.5
Myh4	20290.5	26566	22790	22658
Myh6	27214	14404.66667	16321	5066
Myh7	22611.5	6129	8505	7165
Myh8	13443	24324	14411.75	20635
Myh9	32302.66667	29038.33333	31141.66667	19804.33333
Myl1	30069.5	31400	34101.5	33258.5
Myl10	21034	26652	24576	34863
Myl12a	31717	31709	34493	33030
Myl12b	7105	27852.5	8591.5	8411.5
Myl2	2874	12939	38216	1144
Myl3	3701.5	5023	27835	9130
Myl4	10878.5	10550.5	16299	13734.5
Myl6	15222.5	10717.5	17271.5	16323.5
Myl6b	30571.5	32344.5	28666.5	15921
Myl7	4904	15078	9193	18218
Myl9	3017	6694	8171	12228
Mylip	25681.5	31371.5	10888.5	13603
Mylk	22033	19267	24010.33333	23544.66667
Mylk2	4265	7504	9680	10546

Mylk3	30788	30518	32462	30897
Mylpf	4728	13528	8056	12686
Mynn	11350.5	11416.25	9506.75	11526.75
Myo10	23979.5	18957.5	22395.5	33357.5
Myo15	4039	4981	2681	29149
Myo15b	20574.5	17423.5	10333	29210.5
Myo16	31113	29755.33333	22788.66667	21152.33333
Myo18a	21165.5	16100.5	18977	11223.5
Myo18b	18641	13380.5	24204	13047.5
Myo19	12795	15168	11521	15746
Myo1a	35668	21505	3762	23633.5
Myo1b	17989.8	19182.6	22379.4	25494.8
Myo1c	37208	25344	34712	26499
Myo1d	30292.75	25345.5	32512.5	19462.25
Myo1e	24666.33333	19271.33333	32765.66667	17224.66667
Myo1f	36827	22754	27260	22986
Myo1g	26216	23795	26482	34071
Myo1h	12078	7343	18603	21150
Myo3a	26683.5	27986.5	30624	29192.5
Myo3b	24177	4623.5	9261.5	7118
Myo5a	24293.66667	14590.66667	21060	20368
Myo5b	24261	21874.75	17611	24886.75
Myo5c	22427.5	3717	10710.5	11736
Myo6	25393.66667	28165.66667	29262	17854.33333
Myo7a	16899	11245	11087	6721
Myo7b	14952	16117	19047	18660.5
Myo9a	18465.28571	22037.42857	21813.71429	15435.85714
Myo9b	22784.5	12379.5	14220	15255.5
Myoc	25744	31565	1606	558
Myocd	28433.75	26970.5	29564	29265.25
Myod1	9434	13812	13375	14977
Myof	30135.66667	20927.33333	22220.33333	34829.33333
Myog	1574	3183	8429	8302
Myom1	20308	29303	34932.33333	7739.666667
Myom2	24092.5	26340.5	10954	28920
Myom3	7418	13080	17922	19448
Myot	32620	34836	34964	33172
Myoz1	13561.5	11699.5	14341.5	9651
Myoz2	15933.5	19427.5	5975.5	23458

Myoz3	16233	19692	28249	35312
Mypn	10945.5	25290.5	18996	35104.5
MYPOP	2763.5	20613	14694	10661
Myrf	27258	5956.666667	14469.33333	12859
Myrip	18309	18421	20711.5	18924.5
Mysm1	17102	31125.5	27706	30965
Myt1	32817	35799	36623	36186
Myt1l	28486.5	29597.33333	27255.83333	33870
Myzap	31365	33078	34298	1891
Mzb1	2499	5655	9707	9941
Mzf1	2962	38379	243	35707
Mzt1	21800.66667	23962.33333	22242.66667	22635
Mzt2	15490	17202	19649	17991
N4bp1	15333.66667	20847.66667	4948.666667	10220.33333
N4bp2	19868.4	31962.6	27495.8	29806.6
N4bp2l1	15828	10530	13713	6929
N4bp2l2	21552.75	18351	15504.25	7397.5
N4bp3	1227	40505	35646	38174
N6amt1	31387	30977	25541	23779
N6amt2	32384	2109	38114	38154
Naa10	37759	39318	38360	35977
Naa11	37259	37739	29513	34668
Naa15	19518.66667	18571.66667	10731	12880
Naa16	9799.5	8777	7564.5	9253.5
Naa20	27379.5	22979.5	21593	22263.5
Naa25	21801.75	16711	24431.25	18148.25
Naa30	21965	20720.33333	23265.33333	25552.33333
Naa35	35045.5	31315	19861.75	22955.75
Naa38	13691	17973	16803	15571
Naa40	3766	5231	3084	1048
Naa50	24539	21357.25	17146.25	25893.5
Naa60	16150.5	16050.5	15062	14093
Naaa	4694	3371	35315	32058
Naalad2	23800	24964	296	25145
Naaladl2	25451	26735	28455	27502
Nab1	21398.5	25964.5	17298.5	23580
Nab2	4821	3861	4480	40810
Nabp1	17867.33333	11784.66667	15187.66667	12707.66667
Nabp2	7329	6851	4856	5126

Naca	19306	21378	22626	24090
Nacad	22300.5	18241.5	8878.5	4492.5
Nacc1	18480.33333	8838.666667	15063	9637.333333
Nacc2	18295.5	17847	18196.5	16697
Nadk	12685	15304	11255	15511
Nadk2	25116.5	25284.5	24807	27002
Nadsyn1	13276	13214.5	14858	15616.5
Nae1	36382	39801	32216	37977
Naf1	17985	13581	16116	17278
Naga	27306	21504	22620	21243
Nagk	2952	1091	7459	3891
Naglu	20750	18985	18546	17021.5
Nagpa	31354	30020	26682	30102
Nags	11999.5	14986.5	15496.5	23006
Naif1	10568	11276	3984	10179
Naip1	22872	24877	36598	38461
Naip2	40774	4835	35663	5529
Naip3	2485	40328	6930	9333
Naip5	20358	11290	20349	14011
Naip6	13799.5	8450.5	11047.5	9574.5
Nalcn	30485	24437	27714.75	32664.5
Nampt	23281	14832.33333	21289	14288
Nanog	27521	1756	39451	37023
Nanos1	12249.5	10643.5	23809	21052.5
Nanos2	21619	31423	34056	35556
Nanos3	24025	22669	25992	25054
Nanp	7564	12176	10560	11520
Nans	21404	22662.5	23743.5	26558
NAP000001-074	1820	3577	5739	4103
NAP000003-006	15190	11352	7430	17493
NAP000004-017	16825	19426	18559	17273
NAP000009-005	7992	12418	16394	15108
NAP000023-001	10885	15745	10403	11016
NAP000123-003	14678	13775	13259	16063
NAP000727-001	23916	36932	16834	28245
NAP000866-002	39537	36785	6699	17517
NAP001160-001	2121	10866	35485	32085
NAP001627-002	35897	35340	37302	34350
NAP004513-003	39346	2957	38177	39240

NAP005800-001	15225	17169	14163	17583
NAP005803-002	24909	23263	28685	24585
NAP007796-001	34640	25419	8586	40144
NAP008784-001	33460	29127	34977	31506
NAP010554-001	35831	36016	37613	37579
NAP011615-001	21494	22799	25883	26273
NAP012229-001	22075	17369	21612	17033
NAP012952-001	29366	30766	32834	37115
NAP013093-001	22171	21275	26960	22282
NAP013333-001	32217	6773	36503	8949
NAP013659-001	29378	30860	37099	30834
NAP014233-001	34030	35554	40590	40744
NAP014312-001	23033	3526	28996	3794
NAP014889-001	22348	29091	39556	38604
NAP016308-001	18899	22225	23249	32783
NAP017066-001	1440	4903	18118	20549
NAP017634-001	7489	4358	5905	4508
NAP017949-001	35940	31115	34100	26267
NAP017963-001	22050	9819	34516	17135
NAP018073-001	6371	6230	10530	9332
NAP018089-001	7132	5138	5064	11296
NAP018250-001	1014	3845	7283	5116
NAP018615-001	31425	37672	37671	35316
NAP018688-001	5024	10005	12493	11148
NAP019213-001	35851	24497	8866	13234
NAP019282-001	22277	32319	23778	35290
NAP019608-001	17645	21266	18013	18182
NAP019640-001	3843	8616	6308	9247
NAP019878-001	29147	27123	29379	34973
NAP019902-001	27480	30761	27903	33908
NAP020073-001	9438	14829	18186	24215
NAP020108-001	8824	14208	17427	18640
NAP020114-001	16755	17837	38040	28287
NAP020115-001	39133	4266	35116	37070
NAP020402-001	32176	33680	36665	4018
NAP020421-001	36109	35069	26725	36040
NAP020528-001	41015	10855	8791	15939
NAP020708-001	15023	16630	18815	19658
NAP020770-001	3464	4128	29213	36166

NAP020861-001	13215	14371	20159	18124
NAP021083-001	12748	40721	28610	23201
NAP021334-001	4376	3877	3681	2153
NAP021796-001	11789	635	10214	7892
NAP021919-001	31959	33671	35077	33235
NAP022465-001	9270	33156	33988	1931
NAP022641-001	28082	23713	37325	36735
NAP022882-001	40616	7005	29957	28574
NAP022935-001	10284	35487	19476	17027
NAP023000-001	22751	23242	20277	23951
NAP023193-001	36647	37945	33884	1908
NAP023694-001	39739	5224	7901	8166
NAP023895-001	15610	15832	14832	16701
NAP024949-001	34106	2006	386	5525
NAP025209-001	30673	30099	34588	31150
NAP025218-001	8384	20640	37315	40562
NAP025379-001	17751	14661	16905	11447
NAP025478-001	19569	22450	26641	29248
NAP025806-1	6808	13220	10213	12090
NAP025822-1	14859	22357	19268	20541
NAP025883-1	31974	36836	3488	4822
NAP025894-1	38592	3330	29512	28196
NAP025923-1	756	692	40140	4028
NAP025951-1	18108	18645	16428	18712
NAP026092-1	27676	4745	8099	12077
NAP026179-1	19787	29194	24315	35251
NAP026221-1	32857	34620	37752	34707
NAP026388-1	18653	20932	20975	20757
NAP026408-1	15019	17217	21010	19489
NAP026409-1	17397	19807	19897	19949
NAP026424-1	13076	11886	9096	9485
NAP026504-1	37048	40668	574	39531
NAP026595-1	35297	30085	4321	5869
NAP026710-1	132	4535	1439	3100
NAP026734-1	33546	29536	38002	30029
NAP026836-1	20756	32358	33004	32786
NAP026879-1	23602	24689	26508	26379
NAP026914-1	2520	4487	10695	10463
NAP026933-1	30335	37454	33891	2220

NAP026936-1	35400	34824	28480	26760
NAP026976-1	7924	13057	17007	14833
NAP027004-1	30418	2435	34069	32394
NAP027049-1	10043	11200	13395	12931
NAP027055-1	34521	20663	6306	11701
NAP027099-1	22076	24769	26083	28297
NAP027294-1	17611	17984	26789	29091
NAP027343-1	14474	12584	23168	20401
NAP027372-1	4944	3550	40729	38917
NAP027384-1	22275	22699	37994	36500
NAP027398-1	17392	19680	15065	18320
NAP027406-1	34102	27297	29170	28018
NAP027428-1	22140	21517	18992	21846
NAP027435-1	24487	25553	30816	39504
NAP027441-1	33864	39252	29313	37839
NAP027509-1	28651	30183	34120	30563
NAP027536-1	1493	3560	6797	7561
NAP027564-1	21486	2951	2759	19539
NAP027583-1	22542	19568	23363	21124
NAP027600-1	26868	5991	482	12977
NAP027677-1	16064	15741	21312	20181
NAP027719-1	29685	31247	32156	30762
NAP027724-1	9463	10994	7907	5219
NAP027859-1	35679	35876	24564	37952
NAP027904-1	1485	31256	17851	5441
NAP027922-1	11436	27690	26941	31092
NAP027924-1	6432	11434	7915	14151
NAP027948-1	11154	13407	26038	25196
NAP027952-1	3408	3263	40099	4263
NAP027971-1	14353	17905	23605	20944
NAP027986-1	18029	20215	22282	20605
NAP028025-1	12514	17523	20452	19955
NAP028040-1	24290	25357	27456	6390
NAP028105-1	36745	39982	6204	6382
NAP028203-1	4733	6361	6116	7211
NAP028212-1	3113	6387	1562	4598
NAP028279-1	19815	20416	23820	23312
NAP028292-1	13164	14057	11153	12634
NAP028315-1	34047	39928	6488	4891

NAP028427-1	13603	15493	13081	19212
NAP028432-1	27680	36482	4504	29712
NAP028505-1	6574	10227	9811	12058
NAP028542-1	29474	23901	18353	25387
NAP028546-1	35574	34162	8064	16747
NAP028604-1	9961	10761	16095	17071
NAP028620-1	7822	40743	4160	35776
NAP028664-1	17937	17602	28858	23075
NAP028715-1	40336	35704	22055	23799
NAP028748-1	39318	27683	39325	4400
NAP028749-1	10777	12213	9474	11739
NAP028752-1	31507	32911	34740	32889
NAP028768-1	36784	38337	300	39733
NAP028770-1	15481	12762	21285	19579
NAP028784-1	13233	18405	22925	18070
NAP028816-1	8521	8988	7478	6334
NAP028864-1	6221	9643	6846	4606
NAP028872-1	8062	15088	10863	15332
NAP028877-1	5235	40939	32338	6241
NAP029013-1	1655	8564	8861	8042
NAP029125-1	7483	10650	8611	10595
NAP029143-1	16119	16542	23913	27912
NAP029158-1	20996	22521	23886	24279
NAP029197-1	34862	28748	1388	39849
NAP029234-1	8959	8285	18326	15075
NAP029260-1	20839	21246	17486	27244
NAP029281-1	33067	6621	9473	12846
NAP029297-1	18428	20346	19817	21618
NAP029336-1	1746	825	4482	1706
NAP029405-1	17594	20149	28221	24118
NAP029477-1	3625	5023	7279	4825
NAP029480-1	21125	22536	17297	17162
NAP029491-1	1076	37721	3166	932
NAP029500-1	27678	1643	32323	2608
NAP029503-1	24016	25078	31769	30641
NAP029508-1	6568	32337	6156	3273
NAP029525-1	7180	15060	6991	16330
NAP029533-1	3877	6318	10821	10263
NAP029544-1	29473	31043	5248	40213

NAP029656-1	6065	1376	38484	40612
NAP029716-1	39465	39906	5802	5058
NAP029730-1	3819	7602	6464	9159
NAP029816-1	15221	22108	24052	20867
NAP029844-1	1527	2407	6246	5089
NAP029862-1	3360	4949	40133	37940
NAP029864-1	15588	19937	21954	20510
NAP029896-1	29709	36177	38677	36538
NAP030020-1	29468	35103	38469	36133
NAP030023-1	26500	28577	29405	36423
NAP030047-1	17872	21471	21924	20846
NAP030082-1	1524	7341	9216	8543
NAP030161-1	35887	41019	38769	603
NAP030172-1	17085	21237	22631	25055
NAP030176-1	14549	18897	20549	18131
NAP030232-1	13572	14210	25042	36448
NAP030342-1	31517	2062	362	1448
NAP030387-1	32624	28323	37733	35423
NAP030391-1	21775	26418	30329	35838
NAP030420-1	31422	38613	35096	38505
NAP030421-1	7578	1553	8850	5301
NAP030437-1	13026	19000	26187	27156
NAP030482-1	3499	5461	4455	3777
NAP030542-1	24439	8079	19606	9917
NAP030565-1	13124	40545	7351	18043
NAP030643-1	36029	40831	28542	29099
NAP030672-1	3920	39502	5557	35369
NAP030690-1	24727	38015	947	7432
NAP030755-1	17216	19091	31612	33570
NAP030789-1	28200	36724	37805	3372
NAP030834-1	17733	12838	15488	12954
NAP030842-1	240	3739	11496	12282
NAP030857-1	24333	36070	34369	297
NAP030859-1	22010	21769	26590	27721
NAP030873-1	5002	37301	39695	199
NAP030936-1	38048	37110	32800	31343
NAP030950-1	40082	4741	10211	7074
NAP031201-1	33146	25660	20930	18686
NAP031415-1	39917	6554	7349	4841

NAP031434-1	4852	5644	13408	10566
NAP031516-1	2320	16	39194	38161
NAP031654-1	7855	10164	6438	7861
NAP031678-1	12585	11283	12906	11427
NAP031946-1	11623	10937	7062	9037
NAP032508-1	22524	22496	22314	20960
NAP032554-1	15135.5	15597	12944.5	18723
NAP032626-1	29664	23686	24659	26039
NAP032661-1	9025	7410	5116	4391
NAP032794-1	20079	16686	21684	21202
NAP032883-1	24576	18797	18904	12782
NAP032887-1	28030	22374	20084	22656
NAP032923-1	6155	1831	1082	40840
NAP032972-1	15920	10215	15864	17866
NAP033010-1	36915	39985	3321	1014
NAP033154-1	9003	5897	9115	6163
NAP033351-1	40311	7299	4472	4294
NAP033385-1	27994	493	291	8056
NAP033617-1	1724	2885	40289	1792
NAP033850-1	11982	7945	5107	5311
NAP033903-1	5533	866	36788	36126
NAP034410-1	720	5269	39359	5666
NAP034422-1	30235	18892	16460	15993
NAP034439-1	1497	30147	35929	36784
NAP034797-1	53	6181	4808	9772
NAP034855-1	11952	10902	11947	16615
NAP035212-1	4240	37845	32105	25966
NAP035413-1	20208	15197	18430	17251
NAP035567-1	7243	19304	10768	21308
NAP035656-1	485	7249	3394	7672
NAP036015-1	12486	15428	17773	18848
NAP036221-1	10626	15654	14457	19175
NAP036354-1	33011	23204	22825	22444
NAP036887-1	30356	28102	39011	39426
NAP037326-1	22617	20522	23349	20196
NAP037342-1	6690	1299	4713	2415
NAP037408-1	35666	39388	6473	7207
NAP037463-1	6147	9602	7727	9108
NAP037484-1	7609	9316	14286	15339

NAP037603-1	9720	40788	40362	38586
NAP037823-1	6546	7573	8011	11665
NAP037914-1	35858	3583	28741	38703
NAP037944-1	17722	18487	23767	21869
NAP038019-1	6005	4638	2948	826
NAP038043-1	18371	17403	15215	23963
NAP038076-1	14439	5126	5603	3636
NAP038286-1	15776	14187	16814	14082
NAP038289-1	6232	12722	6631	10910
NAP038884-1	17351	21738	23584	29494
NAP039117-1	2907	3148	5199	3199
NAP039199-1	13903	18263	17375	18477
NAP039233-1	36725	34723	39256	35919
NAP039495-1	12427	16139	21290	22883
NAP039513-1	16973	14737	10479	12867
NAP039752-1	4947	7413	7483	9446
NAP039783-1	4299	7737	4622	4835
NAP039970-1	32851	28007	34178	31411
NAP040064-1	22700	21082	21617	22353
NAP040863-1	16145	19730	19611	19373
NAP040989-1	23502	20589	20983	20239
NAP040991-1	12414	9642	13550	13532
NAP041392-1	32107	27053	28934	27821
NAP041425-1	39285	4068	13915	16930
NAP041487-1	27611	29128	24528	30485
NAP041549-1	31235	32857	2370	36765
NAP042151-1	23413	30163	26308	28319
NAP042167-1	19561	21779	24800	26313
NAP042178-1	39150	27451	25537	26815
NAP042311-1	15977	14459	14601	13641
NAP042348-1	36933	19642	21701	18710
NAP042466-1	1809	1437	8763	13879
NAP042509-1	15073	13534	16740	18726
NAP042543-1	35344	38158	30574	34715
NAP042606-1	39562	38442	6196	6028
NAP042629-1	14413	10685	20832	23135
NAP042681-1	37774	33776	33802	32264
NAP042690-1	40402	40648	1232	2735
NAP042698-1	5248	3398	6487	7900

NAP042792-1	37639	775	4039	5045
NAP042820-1	8928	10100	14903	15008
NAP042931-1	22495	23082	23638	20766
NAP042996-1	2716	40350	3626	36674
NAP043014-1	15675	10810	18356	19330
NAP043130-1	18978	21399	25585	29129
NAP043218-1	13253	33358	29722	22892
NAP043219-1	13537	29055	30450	35092
NAP043344-1	12807	14709	17498	16798
NAP043423-1	7113	7686	12803	14811
NAP043441-1	18676	15352	22842	22872
NAP043477-1	21919	11815	13987	20007
NAP043508-1	7768	11077	19724	15337
NAP043517-1	18224	16076	14465	15723
NAP043554-1	29571	30524	33206	31478
NAP043583-1	6437	1952	20367	19688
NAP043587-1	15253	20238	20654	19718
NAP043627-1	33693	34540	30223	32151
NAP043727-1	15971	18351	18593	13822
NAP043768-1	254	3919	6750	6901
NAP043799-1	40419	7057	6917	8027
NAP043897-1	1518	3546	5558	8205
NAP043943-1	41028	21480	16739	10947
NAP044074-1	12926	15343	17604	16651
NAP044101-1	16330	22327	24089	27190
NAP044218-1	2994	3662	9886	10375
NAP044279-1	11657	4808	11984	16409
NAP044280-1	28211	26225	29463	32067
NAP044298-1	28778	33979	37950	38082
NAP044345-1	13809	17259	23651	25251
NAP044381-1	15361	18239	23597	22576
NAP044463-1	24366	24603	32870	30906
NAP044524-1	19351	14832	6795	9536
NAP044551-1	27496	35787	1022	1888
NAP044566-1	36692	38521	40615	40753
NAP044586-1	8532	12385	19319	17808
NAP044702-1	28581	30124	32193	39466
NAP044968-1	10813	15125	18221	19292
NAP045049-1	38948	36410	37906	35365

NAP045112-1	12835	15273	17830	18877
NAP045151-1	26291	40940	37741	1453
NAP045186-1	15945	19087	37268	28820
NAP045236-1	13496	15149	14581	16035
NAP045382-1	16283	21659	18936	16349
NAP045491-1	33068	38689	7554	1260
NAP045499-1	35120	31962	36613	36062
NAP045501-1	15533	17738	22978	23944
NAP045521-1	31485	22391	23681	24219
NAP045566-1	35190	37921	30457	38698
NAP045622-1	5642	6575	8156	7850
NAP045818-1	30910	21836	19781	20811
NAP045839-1	24455	32660	27685	27010
NAP045842-1	27976	39602	9669	6503
NAP045874-1	40144	31535	31915	1044
NAP045909-1	39206	38175	1703	1105
NAP045951-1	2336	35532	31471	26343
NAP045957-1	6261	3072	10575	12087
NAP046039-1	7395	10234	11687	11798
NAP046044-1	32308	29965	31099	39442
NAP046089-1	9606	5310	19979	9283
NAP046120-1	9316	2840	41103	39742
NAP046134-1	38769	1632	37467	38239
NAP046242-1	15331	36136	21086	31979
NAP046244-1	10527	18043	13370	15766
NAP046356-1	36997	17567	39102	19336
NAP046391-1	329	4656	9189	8160
NAP046511-1	18753	20056	38816	40129
NAP047128-1	652	1266	40855	1633
NAP047273-1	3653	34018	40055	40209
NAP047296-1	39399	35578	38419	33059
NAP047434-1	16121	17464	21323	20078
NAP047941-1	3771	3099	8642	4660
NAP047952-1	24158	25279	27180	26047
NAP048022-1	21813	20476	27820	34717
NAP048126-1	39574	26800	4655	8859
NAP048446-1	11908	11444	20374	20000
NAP048481-1	9965	14158	17015	17097
NAP048581-1	37282	674	12807	17093

NAP048817-1	22431	21821	22715	24505
NAP048860-1	21077	23268	26300	26713
NAP049207-1	3150	4603	12311	13589
NAP049475-1	6171	10088	19520	23158
NAP049499-1	12875	10217	4240	13854
NAP049509-1	18793	38609	12116	12838
NAP049622-1	22104	20040	9490	8742
NAP049674-1	35977	38552	6161	5829
NAP049682-1	38036	9866	24705	22921
NAP049688-1	39222	4327	5839	1804
NAP049726-1	27225	22112	28192	35839
NAP049780-1	2446	3726	12752	11112
NAP049824-1	1794	4564	7017	5502
NAP049879-1	10096	3649	4879	2963
NAP049888-1	4883	11789	9959	10163
NAP049942-1	5938	4695	6090	5333
NAP049990-1	15670	10836	22493	18230
NAP050007-1	38730	26095	40523	7764
NAP050063-1	25252	27627	28375	27834
NAP050368-1	6950	6222	16578	16988
NAP050610-1	11439	11901	14386	14390
NAP050738-1	12376	1498	15422	20946
NAP050776-1	13946	16824	16133	17325
NAP050809-1	15360	40897	34237	9737
NAP050849-1	14982	18282	23348	27023
NAP050980-1	28965	29092	30722	16657
NAP051222-1	28950	30286	31721	30383
NAP051367-1	20867	19184	23267	23748
NAP051522-1	29713	32306	38585	33977
NAP051625-1	10174	5384	13151	9911
NAP051706-1	18236	11472	15271	18788
NAP051762-1	39423	34716	2468	1566
NAP051793-1	10042	3988	13627	11149
NAP051804-1	7715	4355	8263	3959
NAP051844-1	1200	7278	6881	9238
NAP051845-1	32675	33487	1694	1237
NAP051850-1	19749	26056	37754	38415
NAP051871-1	730	2952	6489	7895
NAP051902-1	33412	29315	3890	39347

NAP051913-1	24586	26975	27655	30785
NAP052093-1	14007	14509	15221	14220
NAP052136-1	17403	21492	22450	23926
NAP052152-1	21971	21495	26988	31363
NAP052186-1	33485	33690	40534	1814
NAP052218-1	34758	39573	5492	6182
NAP052224-1	4995	6892	10386	10397
NAP052236-1	35195	40380	6799	5786
NAP052266-1	10834	8886	12645	10095
NAP052311-1	35710	39545	5888	33204
NAP052469-1	14026	6317	14498	8696
NAP052591-1	31194	28425	28668	28046
NAP052656-1	25090	13475	21933	23797
NAP052665-1	20241	15323	21191	17818
NAP052669-1	14862	17846	22610	24857
NAP052715-1	26963	11764	17673	12834
NAP052718-1	21482	21315	29584	28247
NAP052751-1	5398	39625	16627	14226
NAP052987-1	33617	40385	28808	31586
NAP053018-1	31830	31699	40951	12420
NAP053020-1	4688	4142	4804	6562
NAP053062-1	30677	32228	33952	34144
NAP053134-1	558	26456	28374	27261
NAP053162-1	33127	34833	36815	34993
NAP053163-1	15636	9407	11787	9522
NAP053178-1	11837	15286	15296	15369
NAP053239-1	35271	39163	27774	33905
NAP053249-1	36684	1846	6332	3517
NAP053264-1	4065	39403	36843	4914
NAP053302-1	22110	23453	24888	30246
NAP053422-1	6740	11277	8023	15195
NAP053480-1	15230	17965	21502	22605
NAP053505-1	18727	21022	15283	15382
NAP053619-1	23253	24352	26541	35244
NAP053648-1	23574	15295	24883	23576
NAP053675-1	29865	24772	27046	25934
NAP053724-1	40285	32134	38130	35619
NAP053891-1	24983	24871	35721	34840
NAP053909-1	5540	13554	2799	3320

NAP054178-1	25955	27097	2340	40589
NAP054201-1	21047	19135	15276	14367
NAP054202-1	35854	14876	9382	36325
NAP054284-1	39936	1894	1617	6103
NAP054413-1	20287	19238	21598	20039
NAP054504-1	20640	22737	26241	27352
NAP054534-1	18974	20564	21659	22161
NAP054603-1	18409	17395	23757	24340
NAP054660-1	11363	16028	11420	11989
NAP054669-1	40737	32979	8028	3873
NAP054776-1	39300	11644	6712	10582
NAP054962-1	5224	13676	18159	14564
NAP055006-1	17463	17468	26284	22637
NAP055071-1	16720	19549	20761	21331
NAP055118-1	16286	18809	20788	20965
NAP055203-1	24662	23565	36663	35489
NAP055464-1	24497	24443	36063	32648
NAP055542-1	19833	18389	19601	23237
NAP055718-1	10075	11134	11276	12918
NAP055769-1	27650	29089	30442	29500
NAP055877-1	14777	13747	23904	16901
NAP055900-1	16470	17056	15709	14184
NAP055908-1	38478	6961	11478	5209
NAP055926-1	13582	14591	17608	17614
NAP056033-1	7000	2384	7258	10675
NAP056297-1	37941	777	10960	8601
NAP056404-1	4422	10568	13436	20242
NAP056427-1	5034	9133	9629	12492
NAP056443-1	41090	33256	33229	36930
NAP056541-1	16450	19593	19189	22382
NAP056550-1	39043	10673	11114	11186
NAP056725-1	8292	7456	18572	16697
NAP056806-1	26799	22163	19907	20081
NAP056816-1	24044	25191	26951	25827
NAP056910-1	7215	8881	3739	11963
NAP057003-1	25453	22883	27256	20801
NAP057010-1	5646	13239	9865	17548
NAP057017-1	39226	5208	5191	37481
NAP057018-1	26428	30676	30475	33152

NAP057019-1	18568	24853	21213	18530
NAP057020-1	6394	12239	1986	10734
NAP057024-1	24422	35238	27006	34571
NAP057026-1	30727	37990	33199	33733
NAP057029-1	16864	12162	13220	13766
NAP057030-1	18853	26820	20027	22415
NAP057035-1	4212	7695	8004	13010
NAP057037-1	39971	32781	3800	1462
NAP057043-1	34863	35801	3463	1591
NAP057047-1	16056	18214	16443	14961
NAP057049-1	20804	22690	22693	27748
NAP057053-1	1161	12112	39318	39142
NAP057058-1	5450	11718	16442	16738
NAP057060-1	13318	11574	17108	16913
NAP057065-1	5066	9452	22407	19601
NAP057072-1	569	6589	15160	12079
NAP057073-1	40139	2395	14564	13032
NAP057074-1	15024	6934	18631	23181
NAP057081-1	21449	16699	25130	26154
NAP057083-1	10422	12787	14811	14435
NAP057091-1	12477	12879	17895	17410
NAP057100-1	30763	32418	38180	5345
NAP057102-1	13976	20475	22651	29371
NAP057112-1	21367	23213	31863	28816
NAP057114-1	9767	12926	13148	11700
NAP057118-1	25100	35427	37201	37250
NAP057119-1	1631	668	7334	5750
NAP057120-1	34798	2493	4835	10086
NAP057123-1	16257	20783	26599	27853
NAP057129-1	21551	20469	23781	21297
NAP057132-1	26008	38162	39951	6113
NAP057140-1	15022	20161	19528	25968
NAP057141-1	2127	8481	23765	38102
NAP057143-1	24796	27980	24853	29736
NAP057146-1	24529	30210	27060	38920
NAP057148-1	35540	40220	120	9477
NAP057152-1	18438	21583	23147	20044
NAP057154-1	31952	33079	5818	19477
NAP057157-1	31632	37923	36233	2307

NAP057162-1	32218	34089	35393	34013
NAP057171-1	12278	7439	11940	10171
NAP057176-1	29154	30548	33352	40203
NAP057184-1	21801	25629	24550	24576
NAP057190-1	21254	21987	33908	35510
NAP057192-1	6508	8795	8387	9600
NAP057196-1	1305	5255	6629	5585
NAP057198-1	24739	29094	39683	672
NAP057219-1	8700	14856	16915	18655
NAP057224-1	24046	38858	11069	14820
NAP057235-1	6712	11725	15810	13976
NAP057251-1	2837	3003	7846	7246
NAP057252-1	39220	40855	1254	39351
NAP057268-1	33575	363	40200	40588
NAP057281-1	14544	21048	26866	26996
NAP057282-1	3326	1442	12296	10393
NAP057291-1	18970	22940	21329	22498
NAP057296-1	8918	15530	13440	17934
NAP057310-1	30956	29789	38932	38440
NAP057940-1	39499	40523	39482	36596
NAP057956-1	12651	18568	22579	24228
NAP057972-1	23676	26604	14599	23830
NAP058165-1	10000	12556	9769	14012
NAP058227-1	7	34784	5925	8647
NAP058243-1	21003	20905	20877	20219
NAP058254-1	13543	12094	11094	13297
NAP058261-1	11850	6776	9455	7772
NAP058332-1	3323	675	7801	7946
NAP058351-1	11224	9622	18297	13391
NAP058429-1	36973	36553	22218	25707
NAP058430-1	38700	40024	38465	1333
NAP058442-1	18192	15351	15519	15377
NAP058447-1	4153	16301	33876	2922
NAP058478-1	38704	1925	4033	40935
NAP058594-1	21267	23664	26524	30118
NAP058704-1	2252	681	13143	18919
NAP058766-1	34181	30325	25428	30062
NAP058846-1	23028	19210.5	17492.5	17512.5
NAP058876-1	2120	11869	16023	12574

NAP058998-1	13990	19165	11035	17896
NAP059089-1	5570	5633	18983	14954
NAP059333-1	8605	13561	13609	15499
NAP059350-1	39111	37232	31239	37480
NAP059351-1	6274	8565	13551	14548
NAP059380-1	7602	40944	2621	4155
NAP059398-1	15376	24795	26193	34143
NAP059464-1	27159	33980	30986	34324
NAP059572-1	38872	3068	5159	2217
NAP059823-1	37743	4211	1772	15269
NAP059882-1	589	25262	26618	30400
NAP059937-1	37604	37042	37666	37907
NAP060054-1	20451	20512	26070	24634
NAP060374-1	1389	34506	7650	2454
NAP060483-1	7335	8641	7580	5778
NAP060490-1	2515	6138	4739	6410
NAP060775-1	33222	32314	32184	30693
NAP060969-1	39016	29755	31151	29167
NAP061005-1	17846	22730	32226	34590
NAP061133-1	34346	32768	32820	31942
NAP061138-1	12145	10665	7432	12848
NAP061234-1	37825	25585	20293	35275
NAP061240-1	11878	17874	18264	18465
NAP061269-1	29085	30542	32061	30572
NAP061378-1	1172	2793	5600	4614
NAP061407-1	33523	30651	23514	28865
NAP061412-1	30811	27188	38363	35870
NAP061425-1	15183	17052	21886	23565
NAP061435-1	21790	26788	26209	35659
NAP061444-1	18318	20221	16298	17178
NAP061485-1	1966	9201	40334	97
NAP061489-1	1271	5454	7022	6988
NAP061491-1	36184	2070	38663	9625
NAP061492-1	30320	24452	34314	36486
NAP061502-1	12671	16042	18040	22217
NAP061524-1	23921	25019	26956	25826
NAP061630-1	20712	29198	28879	34620
NAP061671-1	14586	11063	8070	14710
NAP061785-1	3823	38142	8972	5745

NAP061794-1	3651	7314	8577	7776
NAP061805-1	20130	21009	22212	16061
NAP061826-1	25882	24528	38961	35758
NAP061847-1	16843	20377	27315	28536
NAP061882-1	8811	14650	18842	15143
NAP061954-1	40136	3193	2016	4177
NAP061993-1	26671	39329	29308	28324
NAP062061-1	33036	35100	39024	39015
NAP062064-1	19591	20440	19453	21142
NAP062223-1	5010	5501	5668	5060
NAP062434-1	33029	35819	38658	32265
NAP062461-1	16515	19610	23891	24863
NAP062563-1	17686	18546	23660	23661
NAP062641-1	24467	25582	27686	26502
NAP062699-1	5937	21355	16707	20781
NAP062835-1	33625	37330	39747	35029
NAP062857-1	37557	1438	2283	2431
NAP062889-1	17979	22238	23336	22648
NAP063000-1	5515	38664	7525	3012
NAP063087-1	2481	6462	37464	4595
NAP063128-1	37073	8096	4350	3379
NAP063206-1	35069	32736	2790	3569
NAP063468-1	30831	31668	40689	38229
NAP063588-1	18835	21808	34537	29717
NAP063697-1	6455	1362	12714	5731
NAP063869-1	7397	10844	27284	1226
NAP063937-1	36931	6274	9714	2882
NAP063950-1	7303	37557	11486	28649
NAP064207-1	21811	21429	24433	24159
NAP064259-1	18331	13343	19413	17014
NAP064811-1	21784	28053	25920	28904
NAP064848-1	24609	24841	32329	34262
NAP064912-1	18351	19188	25447	29425
NAP064987-1	2939	38559	36586	34371
NAP065005-1	25041	26514	27314	26239
NAP065087-1	29805	31378	32748	31683
NAP065110-1	7105	9353	13340	12773
NAP065150-1	14520	16914	20237	19602
NAP065157-1	12934	16011	20593	18596

NAP065219-1	32039	37102	37423	6812
NAP065259-1	37996	7754	6435	13591
NAP065288-1	34846	32326	10465	13920
NAP065340-1	32730	35800	2547	14467
NAP065722-1	9082	4345	13763	12224
NAP065826-1	12324	14632	19061	20281
NAP066348-1	2990	9438	40175	13149
NAP066564-1	8806	6727	23230	25039
NAP066644-1	30679	32322	34291	37571
NAP066647-1	30585	27760	40261	39820
NAP066934-1	39023	314	39743	902
NAP067020-1	3604	7335	38502	13800
NAP067058-1	9386	13001	14669	14204
NAP067064-1	18822	24034	26089	31398
NAP067089-1	18968	24635	25094	24536
NAP067097-1	624	5374	5249	5184
NAP067254-1	19788	19486	26153	24883
NAP067262-1	23831	24868	27565	36920
NAP067426-1	38309	3086	13556	41045
NAP067549-1	25337	32696	34097	38994
NAP067784-1	19218	22739	26974	26092
NAP067942-1	38511	39750	31230	40405
NAP068336-1	32836	5327	39570	7415
NAP068347-1	3287	39861	2014	40720
NAP068654-1	7884	9516	10321	9584
NAP068884-1	17060	19390	20534	19736
NAP068899-1	39433	38489	8941	3136
NAP068969-1	23305	25767	24544	24580
NAP069078-1	3579	8181	19708	9789
NAP069146-1	12004	16632	16152	15873
NAP069148-1	9181	11621	23979	20542
NAP069308-1	14546	20327	17843	19679
NAP069339-1	19385	22622	28529	35142
NAP069690-1	10351	13607	14068	21256
NAP069724-1	6770	32599	32729	1514
NAP070009-1	38398	1707	3306	3010
NAP070124-1	14643	17178	9279	18235
NAP070138-1	18007	23002	23354	23371
NAP070416-1	35487	38922	32420	36710

NAP070455-1	4408	556	139	37412
NAP070462-1	32178	35753	35862	39409
NAP070564-1	23751	20265	37177	35419
NAP070789-1	5984	12348	11601	11761
NAP070792-1	21042	36216	36742	36468
NAP070799-1	19859	21523	22599	31941
NAP070880-1	23713	24262	23852	24898
NAP070910-1	17509	21112	21860	22621
NAP070939-1	12491	13580	21452	21024
NAP070973-1	35601	38328	2488	575
NAP070985-1	7731	3783	39616	33795
NAP071007-1	5268	8376	9955	12629
NAP071025-1	12621	13063	17541	17425
NAP071032-1	39480	30025	10252	11521
NAP071037-1	2632	3955	5190	485
NAP071046-1	17023	19306	20988	25006
NAP071060-1	30321	39728	3208	5199
NAP071064-1	23682	31730	38523	37349
NAP071090-1	12141	18960	14158	18992
NAP071111-1	17175	17898	21234	19561
NAP071160-1	27417	28726	31869	30286
NAP071189-1	20459	26316	26058	23133
NAP071275-1	36250	36744	38882	37576
NAP071276-1	3660	194	4422	37209
NAP071310-1	38933	755	10500	10417
NAP071318-1	18762	22770	30931	32915
NAP071342-1	22097	24811	28911	23793
NAP071351-1	37021	39569	5324	5738
NAP071393-1	23393	24480	37560	25775
NAP071455-1	14550	17578	16817	20600
NAP071456-1	19282	21611	24128	24293
NAP071486-1	1846	38598	31190	29566
NAP071540-1	32831	29543	40423	40281
NAP071547-1	27739	3059	30940	36327
NAP071662-1	12846	20197	27231	29097
NAP071826-1	23339	22074	23659	24412
NAP072161-1	26789	35869	40039	40389
NAP072213-1	19182	22557	22451	19924
NAP092468-001	21773	21420	24713	25291

NAP092588-001	19593	20063	17943	24235
NAP092627-001	37578	36135	37400	39470
NAP093832-001	17328	23531	27566	25087
NAP093999-001	26451	26962	37687	35198
NAP094000-001	13948	16718	20879	22464
NAP094436-001	25668	27867	28541	27678
NAP094515-001	38335	6153	3377	8390
NAP095110-001	24291	39697	37631	31532
NAP095228-001	40654	40905	2351	395
NAP095248-001	34168	21154	16591	14812
NAP095777-001	11155	14079	11796	19661
NAP096050-001	8945	1156	3500	2362
NAP096295-001	34706	36484	38262	40246
NAP096478-001	3307	39207	2181	37595
NAP096498-001	17782	23243	22265	24010
NAP096720-001	5781	11426	4619	6148
NAP097318-001	21505	22179	21765	21993
NAP098046-001	16790	37513	27496	33070
NAP098303-001	24822	26167	30936	472
NAP099299-001	39650	1113	30120	40872
NAP099360-001	16903	20565	28080	30891
NAP099398-001	11718	15836	13868	17236
NAP099819-001	5488	1726	37247	36569
NAP100068-001	13912	20753	19616	16946
NAP100137-001	35161	38370	7896	10872
NAP100574-001	10906	15025	14552	19167
NAP100709-001	20581	33404	24223	31995
NAP100785-001	23992	25224	39653	25285
NAP100987-001	26283	9204	7071	34694
NAP101150-1	38792	6893	12334	9670
NAP101299-1	18160	19737	19559	23760
NAP101637-1	36844	35698	30900	33359
NAP101638-1	8000	11435	11632	11670
NAP101659-1	22640	38663	38244	2789
NAP101960-1	41083	3965	32571	40538
NAP101973-1	14722	15391	17710	18881
NAP101986-1	18999	19738	22966	34788
NAP101988-1	4680	10683	13926	12898
NAP102091-1	13774	13806	18836	19283

NAP102121-1	13114	14249	17720	17939
NAP102249-1	29359	30837	33023	37570
NAP102257-1	1315	784	40529	2923
NAP102337-1	8307	5905	7646	3020
NAP102363-1	33573	5559	9124	6577
NAP102386-1	8678	22404	7087	8339
NAP102414-1	30933	34500	31118	38656
NAP102507-1	27717	29305	30113	28835
NAP102548-1	19806	17844	16926	17919
NAP102554-1	16540	22294	22230	23321
NAP102571-1	9183	11888	18781	18813
NAP102580-1	23845	25003	26528	25555
NAP102621-1	19032	26469	22082	21283
NAP102624-1	24543	3046	27877	37402
NAP102637-1	40121	10193	3759	1248
NAP102645-1	3195	4251	37396	39822
NAP102738-1	8504	7897	12819	11725
NAP102790-1	17880	22891	18406	22993
NAP102794-1	16835	39461	27173	31412
NAP102831-1	5316	12330	4512	2392
NAP102845-1	37180	28266	38939	31714
NAP102962-1	18152	23472	27555	24934
NAP102981-1	17100	21866	22266	23922
NAP103015-1	32266	34046	35882	34186
NAP103072-1	16625	13855	15179	9770
NAP103151-1	11373	10383	7384	11903
NAP103317-1	17173	28969	30766	34739
NAP103337-1	25073	20204	20004	16949
NAP103349-1	36333	9734	38021	17250
NAP103523-1	29267	20181	25435	19904
NAP103569-1	34066	26708	24499	25267
NAP103730-1	36749	8747	6589	40472
NAP103757-1	10739	9319	11378	12364
NAP103812-1	19116	13294	14410	10749
NAP103878-1	40659	12166	36156	3065
NAP104001-1	8743	6297	40766	419
NAP104272-1	15908	13403	11597	12438
NAP104290-1	13601	13013	12473	10660
NAP105656-1	15628	11171	7660	1466

NAP105665-1	40138	31412	41062	38637
NAP105744-1	9121	13176	18005	18201
NAP105815-1	38212	574	907	843
NAP106314-1	32770	34913	35154	33328
NAP106399-1	6575	10154	14801	11792
NAP106668-1	6817	9636	7659	7983
NAP106692-1	29489	28353	30485	29709
NAP106729-1	9813	11312	15192	15869
NAP106831-1	18981	20172	21481	23902
NAP106877-1	37115	5429	7016	11412
NAP106897-1	17934	16475	17159	20524
NAP106900-1	5118	7948	9244	8989
NAP106922-1	40517	2974	4728	5702
NAP107172-1	11643	14273	16082	18031
NAP107236-1	17464	16605	20616	31889
NAP107273-1	14371	39049	22144	24259
NAP107286-1	31438	32934	6725	28744
NAP107478-1	34432	4649	36559	38957
NAP107554-1	20969	23582	23977	25324
NAP108117-1	1844	4550	7540	5458
NAP108141-1	31651	34052	40768	38599
NAP108144-1	38007	37128	37304	39162
NAP108202-1	8332	5934	31804	38884
NAP108265-1	23027	27207	30092	33631
NAP108333-1	33679	25797	26172	23933
NAP108362-1	25617	28367	28859	36957
NAP108366-1	6747	33384	40870	38658
NAP108369-1	23903	26473	26448	25422
NAP108447-1	27531	28708	29470	28108
NAP108451-1	20667	11952	12535	17659
NAP108455-1	22191	26487	24373	23885
NAP108462-1	15751	14136	15649	15426
NAP108473-1	34016	37751	32294	40547
NAP108475-1	1313	2966	38224	3585
NAP108488-1	13296	10616	16768	12934
NAP108500-1	18291	13065	9166	11707
NAP108515-1	33234	16923	1075	20508
NAP108516-1	1685	40504	6973	5713
NAP108522-1	35786	35389	37553	39081

NAP108526-1	15126	22016	17704	23085
NAP108561-1	35265	32266	1262	1078
NAP108602-1	15540	20474	28440	31524
NAP108607-1	7659	12704	23381	21201
NAP108672-1	40295	4295	16642	14750
NAP108679-1	34659	1561	4140	8680
NAP108687-1	7103	9441	16325	16213
NAP108708-1	19601	20387	24933	24413
NAP108723-1	12733	16813	19265	19684
NAP108760-1	38570	40560	4058	4866
NAP108772-1	24939	31735	28384	27180
NAP111117-1	39934	3506	287	754
NAP111137-1	13732	15097	22562	23818
NAP111197-1	31867	324	10335	9788
NAP111374-1	29452	36412	25985	554
NAP111629-1	3865	40307	8895	6549
NAP112201-1	11259	12874	15277	12911
NAP112302-1	39298	3888	2529	7836
NAP112348-1	39767	40087	758	1056
NAP112575-1	13465	15658	17867	17374
NAP112590-1	22725	17606	13249	23264
NAP112605-1	24266	25416	27585	35285
NAP112607-1	3430	8555	6932	39408
NAP112641-1	30419	35512	41042	38211
NAP112671-1	37629	36922	31037	35505
NAP112741-1	4059	5273	8602	7302
NAP112858-1	23583	24183	24597	25612
NAP112946-1	13068	13565	15567	14211
NAP113002-1	12505	15063	13041	17697
NAP113018-1	10996	11122	18202	14388
NAP113144-1	37601	13252	39885	40789
NAP113158-1	27734	5480	5793	8509
NAP113200-1	40913	1856	13895	14197
NAP113215-1	39808	3679	880	3525
NAP113249-1	28712	7279	30609	29266
NAP113251-1	38581	14046	13949	17057
NAP113288-1	13428	16226	11637	13374
NAP113293-1	19549	22764	15259	19800
NAP113348-1	35263	38185	1723	3513

NAP113376-1	14333	12534	18721	23368
NAP113434-1	33895	36665	39331	36974
NAP113636-1	31384	23131	25843	24116
NAP113722-1	3993	2506	11014	7564
NAP113797-1	29797	36064	33792	2054
NAP113912-1	5230	20959	15137	15506
NAP114245-1	777	4978	8347	6164
NAP114258-1	21591	14172	11256	15145
NAP114294-1	40905	5516	4687	8792
NAP114506-1	8593	27245	5450	5306
NAP114798-1	2283	8078	7226	11250
NAP114957-1	6358	11446	9644	15449
NAP114978-1	29402	21673	18332	29351
NAP115012-1	37474	24500	24852	21196
NAP115231-1	15556	14270	18325	17833
NAP115237-1	36251	28100	31516	21902
NAP115274-1	7728	40642	134	34751
NAP115295-1	24451	38573	34795	35970
NAP115391-1	23884	26233	33007	37794
NAP115396-1	33077	35024	36535	317
NAP115402-1	216	8356	15471	18778
NAP115505-1	23231	24773	34535	34109
NAP120816-001	20652	38862	40698	40645
NAP121072-001	3751	6813	9131	8451
NAP121155-001	7083	14705	13173	17596
NAP121186-002	11863	17021	13626	14270
NAP121332-001	27744	38833	5635	2788
NAP121362-001	18921	19819	14492	12233
NAP121938-001	10544	8501	11505	10266
NAP122751-1	6940	10868	12605	14642
NAP122790-1	2573	2728	6542	6295
NAP122799-1	33046	28912	23835	30728
NAP122825-1	16471	15054	11988	15319
NAP122837-1	20342	21144	24956	22972
NAP122843-1	12090	14161	25096	27766
NAP122875-1	1798	31047	7863	914
NAP122876-1	37185	949	40416	1091
NAP122883-1	33770	2813	41071	23383
NAP122889-1	37252	29594	33274	5864

NAP122972-1	17581.5	19191.5	6305	6464
NAP123016-1	31783	22582	38263	32885
NAP123065-1	274	821	5242	5510
NAP123103-1	14996	17121	23299	22684
NAP123107-1	28229	40698	1426	324
NAP123115-1	32919	21946	15329	20028
NAP123123-1	37396	29307	36491	40802
NAP123135-1	22774	20809	22804	22139
NAP123168-1	21104	21152	24204	19945
NAP123201-1	11978	4126	5417	6677
NAP123204-1	10	3103	12072	11537
NAP123205-1	20865	21363	24982	25348
NAP123216-1	4573	13736	14123	17019
NAP123224-1	36787	32035	20675	31144
NAP123291-1	9833	11853	19977	21355
NAP123300-1	3270	3303	3177	4758
NAP123409-1	22830	21460	23244	20909
NAP123415-1	33604	24852	25877	23741
NAP123465-1	8961	37159	8845	10293
NAP123484-1	39323	1662	2566	2658
NAP123493-1	32478	34243	35317	33500
NAP123498-1	22990	22008	3645	28738
NAP123517-1	38542	487	30583	30397
NAP123520-1	5405	9966	8783	10472
NAP123523-1	34393	36964	1419	2211
NAP123524-1	34206	40635	674	1321
NAP123578-1	5125	9662	10366	15655
NAP123597-1	25841	27127	28710	27554
NAP123601-1	7569	10361	10049	12605
NAP123620-1	26869	21869	22921	21583
NAP123621-1	39463	5096	39761	1189
NAP123674-1	22274	25064	27203	30030
NAP123695-1	15510	18539	22269	24663
NAP123700-1	8437	12707	15664	23393
NAP123705-1	39553	36524	1846	2399
NAP123712-1	36679	40360	5771	6675
NAP123766-1	19660	21647	21026	23233
NAP123823-1	10975	13942	22744	17587
NAP123836-1	12135	6059	7005	4146

NAP123837-1	33109	30990	27261	26116
NAP123840-1	6986	2547	1264	3650
NAP123843-1	5684	9597	12704	16832
NAP123875-1	21258	23799	39842	1100
NAP123906-1	14717	9173	14196	9087
NAP123917-1	1492	40492	1392	2066
NAP123919-1	13337	21597	33175	20379
NAP123939-1	21114	16465	9789	7595
NAP123950-1	29350	25157	25502	24994
NAP123993-1	37281	316	3072	2503
NAP124210-1	25944	27285	31302	31176
Nap1l1	10875	9538	9434	11331
Nap1l2	16690	15375	12467	10711
Nap1l3	33540.5	34735.5	37242	34633.5
Nap1l4	19040.33333	18168	16561.33333	18600
Nap1l5	24776	25837	26828	36094
Napa	14814	11924	9285.5	7588
Napb	10455	7832	7595.5	9731
Napepld	4671.5	6414.5	6075.5	11348
Napg	20222	24585	23010	31961
Naprt	3434	4524	6870	6315
Napsa	7127	66	39258	28704
Narf	18083.66667	28376	20778.33333	34445.66667
Narfl	39188	36292	35770	35394
Nars	35555	30690	25808	28926
Nars2	19449.66667	18914.33333	18194.33333	21427
Nasp	35497	20997	19319.5	20478
Nat1	32004	34529	34460	32659
Nat10	30638	23839	19635	22533
Nat14	3496	3935	6592	8709
Nat2	7959	7886	7302	5511
Nat3	29212	30247	31671	30378
Nat6	7778.5	25250	9405.5	27225.5
Nat8	13486	15860	17497	18537
Nat8l	40368	17277	29670	14068
Nat9	12704	17429	19794	20637
NATD1	1339	29373	39058	28879
Nav1	23549.33333	10188	18609.33333	12598
Nav2	14485.75	18016.25	14264	27772.5

Nav3	23684.375	19722.625	22207.125	19017.875
Nbas	15902	36249	35095.5	34056.5
Nbea	21704.33333	8717.666667	19879	15636.66667
Nbeal1	16806	6724	6583	9046
Nbeal2	25980.5	19794	19913.5	16861.5
Nbl1	24115	24967	33017	34924
Nbn	30951.66667	33952.66667	30944.66667	30980.66667
Nbr1	9553.333333	8676.333333	10920.33333	9117.666667
Ncald	27797.4	25259	28966	29774.8
Ncam1	15575.11111	17583.11111	22698.33333	23550.77778
Ncam2	32120.33333	32427.66667	22484	24419
Ncan	10554	14575	6828	7361
Ncapd2	9977.666667	7714.666667	11048.33333	8157.666667
Ncapd3	29734.5	27825	28497	26409
Ncapg	22851.5	21923	22766	23060
Ncapg2	10725	3106	6888	1620
Ncaph	2294	35482	37593	33009
Ncaph2	30150	29215.66667	24052.66667	26678.66667
Ncbp1	15574.5	18804	16023	18597.5
Ncbp2	15258.5	18648.5	13612.5	19602.5
Ncdn	37280	36786	37754	37064
Nceh1	33218	9783	21385.5	10673.5
Ncf1	22367	24090	17176.5	20838
Ncf2	9552	9892	8916	13703
Ncf4	25811	28556	25303	35449
Nck1	19894.33333	14145.33333	15529	11774
Nck2	14540.5	17296.75	14468	13367.5
Nckap1	27559	23420	27359.66667	8362.666667
Nckap1l	12520	13680.5	21049.5	14655.5
Nckap5	28241	30674.66667	24116.33333	34852
Nckap5l	21702.5	24704	20665.5	16502.5
Nckipsd	4518	40770	38471	27897
Ncl	20042.75	19275	17814	18647.75
Ncln	9955	9037.5	4218	4148
Ncmap	19275	16663	18370	17324
Ncoa1	36798	24105	30267	24349
Ncoa2	16227.25	12053	13315.25	12927.25
Ncoa3	15805	24031.33333	13080.66667	23205.66667
Ncoa4	23408.75	26412.75	18153	25163.75

Ncoa5	8335.5	3457.5	24223	25468.5
Ncoa6	13997	7970.5	7653.5	5302.5
Ncoa7	35603	33795.66667	20285	7664
Ncor1	10158.125	19777.625	19965.25	21722.375
Ncor2	21723.5	14496.5	18604	13009.5
Ncr1	1607	33727	35775	775
Ncrna00085	10866	21578.5	32415.5	23400
Ncrna00086	17361	20931	22695	23408
Ncs1	16483.33333	17909.33333	20487	21923.33333
Ncstn	15147	14879	13710	16233
Ndc1	37341	30114	26796	28181
Ndc80	34243.5	27992	30266	27401
Nde1	17004	17168	14914	16672
Ndel1	13064.5	11282	8466	11586.5
Ndfip1	23376	22292.5	23722.5	22353.75
Ndfip2	21757.33333	18504	19791.66667	18843.33333
Ndn	6371	8581.5	19032.5	20914
Ndnf	29555	29036	25124.5	15502
Ndnl2	17329.5	21867	25639	25328
Ndor1	20100	28871.66667	28416.33333	29194.66667
Ndp	18088	27222	25002	26648
Ndrg1	32526	17279	29501	19978
Ndrg2	27474	19120	28310	30139
Ndrg3	24597.5	19694.75	20438.5	18946
Ndrg4	19957.5	15267	28863.5	18557.5
Ndst1	19827.5	14296.75	16200.25	12304.25
Ndst2	26602	19379	23940	19025
Ndst3	20345	20255	20099	19992
Ndst4	21748	22763	955	39368
Ndufa1	24481	28887.5	26312	26102.5
Ndufa10	33657	32996	29579	36955
Ndufa11	1613.5	10180	20698.5	3099.5
Ndufa12	18471.33333	8992.333333	20475	19425
Ndufa13	695	8021	8517	7184
Ndufa2	14161	19321	20550	21088
Ndufa3	37555.5	2928	18591	21719
Ndufa4	13508	19831	18041	18445
Ndufa5	14391	18811	16659	18142
Ndufa6	3632	10013	9831	8325

Ndufa7	12957	18345.5	20227	19162.5
Ndufa8	8422	11866	12385	12980
Ndufa9	11853	15182	17037	18673
Ndufab1	28979.25	15862.75	25553.25	16498.75
Ndufaf1	13178	18233	14750	16331
Ndufaf2	22794	35901	27718	31473
Ndufaf3	10305	21368	18178	21895
Ndufaf4	15386.33333	15992.66667	11464.33333	12557.66667
Ndufaf5	25616	35856	24391	30124
Ndufaf6	426	5071	264	3593
Ndufaf7	27695	31102	26218.5	24605.5
Ndufb10	27943	27062	29778	27149
Ndufb11	28962	33587	34211	30907
Ndufb2	22667	20699.5	20143	18241
Ndufb3	6514	11626	12314	12416
Ndufb4	15197	18589	14548	15791
Ndufb5	18952	22654	18810	19233
Ndufb6	12134	19777	19879	21473
Ndufb7	23816	31749	31763	33815
Ndufb8	25891.5	30405	28568.5	28537.5
Ndufb9	24119	35845	33323	34361
Ndufc1	20213.66667	8391.666667	16477.33333	20599
Ndufc2	19434	26930	22734	24094
Ndufs1	20180	20479	20841.5	20336.5
Ndufs2	34385	35648	28104	35417
Ndufs3	6728	10916	10118	10030
Ndufs4	20684.66667	26255.33333	24443.66667	24073.33333
Ndufs5	34837	359	40193	2592
Ndufs6	11051	15644.66667	10105.66667	12231.33333
Ndufs7	10528	10487	10266	11770
Ndufs8	22773	30989	27133	24906
Ndufv1	8837	8941	7570	9171
Ndufv2	37112	3271	1639	611
Ndufv3	26286	27031.66667	27140.66667	25140
Neat1	38630	24040	39161	40728
Neb	22367.28571	27196.71429	26412.71429	18612.14286
Nebl	25747	35274	19376.5	30292
Necab1	34083	36116	8927	1000
Necab2	13221	14782	19308	18990

Necab3	28068	31065	38648	1397
Necap1	21303	20344	19915	19817
Necap2	27446	27862	23710	27388.5
Nedd1	20275.66667	22602.33333	20526	21427.33333
Nedd4	17960	8187	21397	17340.5
Nedd4l	26748.5	18004	20987	23422
Nedd8	21578	27813	25680	29332
Nedd9	21956.5	12254	17495	30343.5
Nefh	37358	18478	3290.5	5745.5
Nefl	31417	1967	5102	23619
Nefm	32272	33655	34538.5	14826
Negr1	26447.16667	31277.33333	18972.16667	30401.66667
Neil1	8636	10436.5	8493	8306.5
Neil2	38996	22543	23070	20587
Neil3	15407.33333	15040.66667	18730	10850
Nek1	17657.14286	15535.85714	17927.57143	17736.57143
Nek11	9024	4035	12548	8635
Nek2	26337	21452	20575	26587
Nek3	1982	38482	39030	33480
Nek4	1508	38851	39994	37580
Nek5	3316	5658	13214	961
Nek6	18446.66667	24405.33333	20526.33333	27799.33333
Nek7	20885.66667	13407.33333	14481	14648
Nek8	13412.5	7420	7867.5	25013.5
Nek9	7263	2918	7234	2641
Nelfa	16481.66667	14093	14096.33333	26778.33333
Nelfb	16561	13271	15555	14368
Nelfcd	9436	4044	9551	5592
Nelfe	37901	1467	40989	40272
Nell1	27891.5	27071.75	33328.75	28072
Nell2	27605	29183.66667	19070.66667	23723.66667
Nemf	17430.33333	11758.66667	31654	30567
Nenf	10516	18422	20555	20382
Neo1	15607.75	24416.75	13801.25	25305
Nepn	12853	18085	18487	17327
Nes	9745	36621	16512	2776
Net1	30780	31168.5	26781	28061.5
Neto1	23095.33333	25257.66667	27135	26713
Neto2	27010.5	27768	29754	26486

Neu1	11314.5	14360	11469.5	15112
Neu2	37555	33506	1324	39584
Neu3	30197	25171	29345	26924
Neu4	13608	15919	20092	16853
Neurl1a	14091.5	14030	32820	17527
Neurl1b	30298	25740	22362	22639
Neurl2	36331	25878	34247	23832
Neurl3	16898	32291	26728.5	26152.5
Neurl4	38231	34034	38817	35668
Neurod1	28587	30406	30543	29205
Neurod2	12827.5	29640	22058	35311
Neurod4	27792	29003	31150	29795
Neurod6	37719	36276	36591	2179
Neurog1	7494	10415	14834	16350
Neurog2	38426	1472	10155	9683
Neurog3	20624	22976	35972	37629
Nexn	16554	17594.5	23123.5	25264
Nf1	23221.83333	17575.83333	13743.83333	20443.33333
Nf2	25459	24334	26533.5	4819.5
Nfam1	32306	21600.66667	28279.66667	31671
Nfasc	9646.666667	14657.66667	16984	14675.33333
Nfat5	22968.66667	19553.5	18376.5	20871.16667
Nfatc1	24796	13627	14485.5	11665.5
Nfatc2	21291.5	14613.5	18844.25	23692.25
Nfatc2ip	18201	17988	23051	21838
Nfatc3	17850	25834	21247.66667	34810
Nfatc4	14693	15679.5	14508.5	16533.5
Nfe2	35214	31398	21754	15068
Nfe2l1	9259	12574	11303	16495
Nfe2l2	30041.5	22434.75	25533.75	22126.75
Nfe2l3	27524	24947.5	11903	25149
Nfia	21042.75	28420.75	13141	16258.75
Nfib	26403.5	27570.66667	19433	25289.5
Nfic	28539	18571.75	31065.75	23871.25
Nfil3	11296.5	14119.5	8517	16081.5
Nfix	20772	14652	16472	8397.5
Nfkb1	13321.75	12612.75	18454	14626
Nfkb2	14148	36960	14939	31508
Nfkbia	1859	24961	38886	21397

Nfkbib	28204	288	27085	40073
Nfkbid	38749	28865	32461	29504
Nfkbie	12361	40356	16151	1358
Nfkbil1	10773	9380	9317	8987
Nfkbiz	30537	7210.5	21879.5	4372.5
Nfrkb	16806.66667	12079.33333	22787.66667	23569
Nfs1	24791	28928.5	24737.5	24064.5
Nfu1	26823.5	27719.5	27717	29264
Nfx1	12592.8	13208.4	11619.6	13551.8
Nfxl1	8869.666667	11004.66667	9596.666667	10295.66667
Nfya	30666	22699.5	21941	23397
Nfyb	29109	27043.5	27342	27540.5
Nfyc	4258	7070	2974	3996
Ngb	40127	40475	4117	3600
Ngdn	13852	16861	19257	18372
Ngef	37905	38916	4343	4380
Ngf	8863	39561	2127	10598
Ngfr	10780	13786.5	16891.5	17889.5
Ngfrap1	37497	37626	40197	35484
Ngly1	15301	19605.75	16701.5	18254.75
Ngp	29358	29852	26322	26254
Ngrn	27597	9680.5	8473.5	10982
Nhej1	24448	26348.5	25495.5	26180
Nhlh1	36814	37052	39262	30859
Nhlh2	24759	25844	28626	2040
Nhlrc1	36102	38409	34001	34736
Nhlrc2	22126	21020.75	29339.25	19294.5
Nhlrc3	39190	18927	39589.5	21648.5
Nhp2	40004	7968	6746	11047
Nhp2l1	28828	32330	25772.5	29290
Nhs	29701	31179	19841	31262
Nhsl1	22266.25	20636.25	26763.5	24850.75
Nhsl2	8169	39276	39292	39502
Nicn1	4066	2938	40791	40620
Nid1	38343	38143	4593	12704
Nid2	14571	39939	5406	4208
Nif3l1	35616	21836.5	36008	20817
Nifk	40645	4788	2044	4342
Nim1k	21381.25	10392.75	22416.75	17220.75

Nin	10194.33333	19383.66667	7684.666667	7262
Ninj1	22185	20148	19431	21234
Ninj2	3484	9596	9315	11304
Ninl	12348	18673	22216	28278
Nip7	34345.5	11571	9489.5	11107
Nipa1	16568.66667	16326	16050	12532
Nipa2	13246.5	22433.25	24185	22755.25
Nipal1	23152	23205	1845	40912
Nipal2	31004	28208.33333	35727.33333	31142.33333
Nipal3	35568.5	32444.5	34205	29997
Nipal4	31548.33333	15169	25067.66667	26951
Nipbl	19418.33333	25347.83333	25017	23216.83333
Nipsnap1	20171	21424	22461	21947
Nipsnap3a	16944	18240.33333	24317.66667	20827.66667
Nisch	19992.75	12843.75	14775.75	14214
Nit1	16518	16805.5	14792	16041
Nit2	13022	15444	12112	13688
Nkain1	10985.5	25219.5	30578.5	24837.5
Nkain2	25041.25	30332	27820.25	30101.5
Nkain3	23166.4	18837.2	24501.4	18933.8
Nkain4	20725.5	17186.5	7304.5	24409.5
Nkap	37186.5	37488	37243	37976
Nkapl	37524	38314	39772	38615
Nkd1	8530	28222.5	5873.5	9418
Nkd2	27221	13753	27823.5	30956.5
Nkg7	32832.5	35007.5	37945.5	20152
Nkiras1	16117.5	24405	2767.5	11147
Nkiras2	20748.5	17109.5	20548.5	16408.5
Nkpd1	14042	14032	15553	17989
Nkrf	6966.5	25333.5	21758	21383
Nktr	12373	10333.66667	22012.66667	25605
Nkx1-1	1472	651	7120	4452
Nkx1-2	18837	22188.66667	27114	22244
Nkx2-1	37643	36994	35871	32416
Nkx2-2	29455	40416	5654	7330
Nkx2-3	33649	33368	3575	1688
Nkx2-4	22131.5	25116	25084.5	24754
Nkx2-5	37738	40591	4010	2342
Nkx2-6	17583.5	20396.5	20143	23583.5

Nkx2-9	27723	26494	28110	27177
Nkx3-1	33041	38722	36889	35014
Nkx3-2	29515	34977.5	18663	18144
Nkx6-1	7551	9476	13683	15458
Nkx6-2	37548	38310	37267	4274
Nkx6-3	19290	22986	21470	24555
Nle1	41035	39772	39755	38103
Nlgn1	26919.44444	28250	24677	25416.11111
Nlgn2	13062	16734	18601	20453
Nlgn3	8681	21030	15863	21811
Nlk	13578.33333	9585.666667	20941.33333	19716
Nln	17118.5	17188.5	15267.5	16071.5
Nlrc3	16116.5	29663	31979	30686
Nlrc4	32786	465	23279	37554
Nlrp10	15560.5	22623	19377	25373
Nlrp12	30960	39174	33391	31715
Nlrp14	28338	39353	38143	36925
Nlrp1a	27305	25993	27654	28875
Nlrp2	16478	21415	21439	22153
Nlrp3	10099	13674	13586	14669
Nlrp4a	18517	34410	16036.5	19048.5
Nlrp4b	31117	32485.5	17802	30539.5
Nlrp4c	14710	22514	19965	25742
Nlrp4f	4978	5556	6217	13574
Nlrp5	2705	35905	2587	2593
Nlrp6	22025.5	4338.5	11908.5	12475
Nlrp9a	4916	39643	1094	5063
Nlrp9b	7645	10442	5392	40958
Nlrp9c	18651.5	39330.5	26392	7413.5
Nlrx1	18594.33333	16468	24992.33333	25866.33333
NM_009309.2	10969	13254	19663	22106
NM_015770.3	16618	17717	24493	24800
Nmb	17290	18047	18020	17370
Nmbr	23315	35025	35854	40570
Nmd3	3879	7925	6108	9330
Nme1	38206	39984	37172	34786
Nme2	38340	1101	402	2042
Nme3	20956	18953	21547	16787
Nme4	18555	14709.5	20029.5	13810.5

Nme5	7724.5	21531	3147.5	8042.5
Nme6	33456	8737	40286	7086
Nme7	8677.5	8173.5	6670.5	6178
Nme8	3756	5963	14467	4002
Nmi	32374	1981	33833	40060
Nmnat1	21985.5	5925	25167.5	6747
Nmnat2	27472	29048.5	31151	28769.5
Nmnat3	16410	15201	11674	11352
Nmral1	7722	10203	4806	3456
Nmrk1	62	7765	7225	9040
Nmrk2	20347	20810	22323	25395
Nmt1	21560	21709	20122.5	22309.5
Nmt2	20229.5	22192	39861.5	23959
Nmu	28577	3691	32360	30790
Nmur1	39098	40892	3309	6642
Nmur2	25172	26401	28136	27196
Nnat	3023	3406	2729	5369
Nnmt	15311	13523	21948	20732
Nnt	30729	29210.5	14430.25	15759.75
Noa1	5760	10106	4737	3213
Nob1	6060	11593	7731	11442
Nobox	3576	33258	2749	37185
Noc2l	12985.5	11827	13610.5	12186.5
Noc3l	19604	27862	24364	29683
Noc4l	6068	3699	3457	6823
Noct	1884	27534	37937	32349
Nod1	33486	25588	28009	25336
Nod2	21481	39830	22400	38320
Nodal	6702	9653	14525	13859
Nog	10857	8692	38673	36174
Nol10	38384	931	29323	36785
Nol11	21209	21170	18293	21560
Nol12	5463	7827	10859	10831
Nol3	22112.5	17291.5	9309.5	26578.5
Nol4	20193.25	21736.75	24976.25	26173.25
Nol4l	13760	38235.33333	13429	24334
Nol6	13400.5	13715	12907.5	12773
Nol7	11733	21349	20120.5	22877.5
Nol8	10123	7134	17263	19394.5

Nol9	19730.33333	30484.33333	14517	28754
Nolc1	15476.5	15826.5	13307	15612
Nom1	14359	15517.5	13597	15408
Nomo1	5447	7662	7215	10537
Nono	22040.5	17117	18934	17480.5
Nop10	40181	38643	29509	35340
Nop14	27071.5	24345	20975	21678
Nop16	18035.5	22472	20205	24824.5
Nop2	39407	36919	35222	36939
Nop56	23929	24302	25612	7553.5
Nop58	17410	21633	19488	20555
Nop9	16850	17971	16177	16960
Nos1	13352.5	15212	22893.5	24095
Nos1ap	8714	8636	11271	11085
Nos2	5869	7789	9054	14591
Nos3	35707	9314	38774	5186
Nosip	19193.5	2340.5	20186	40445.5
Nostrin	6454	10204	4885	6219
Notch1	30877	21476	19574.5	20645.5
Notch2	22918.33333	18382.66667	22759.66667	22563.33333
Notch3	7835	10918	21539	19468
Notch4	6757	40539	4267	35228
Notum	7583	2465.5	15872.5	11477.5
Nov	37933	30030	30637	39221
Nova1	30669.66667	20008	23388	20715.33333
Nox1	31162	34375	29147	27953
Nox3	32285	33624	36235	37241
Nox4	26819.5	14550.5	21801	38273
Noxa1	18175	20422	24163	21563
Noxo1	18684	16488	17927	17679
Noxred1	26857	22659	32954	28975
NP_001019883.1	30156	11473	40461	10175
NP060941	10198	10808	17722	13710
NP063118	21036	18021	18350	18857
NP063356	25985	30701	40326	39768
NP063435	36294	29916	28581	27369
NP063612	250	38746	3101	39086
NP064182	22589	37604	38913	39771
NP064425	16020	19282	25274	25643

NP064466	18335	16386	23226	21017
NP185245	8033	8859	13380	10408
NP377814	16944	11350	24255	20458
NP430906	1612	40959	31682	29997
NP431009	6596	1009	14693	14434
NP431110	22311	27882	38543	39683
NP431243	39096	39795	4224	4093
NP431391	9894	18482	17862	19064
NP431392	29811	31683	32950	6116
NP431517	17269	17667	29201	32642
NP431626	26920	28608	29443	37133
NP431630	4598	19495	13820	15844
NP437410	12676	6632	6646	4239
NP515327	4969	33862	820	605
Npas1	316	18668	40790	6467
Npas2	29827	23274	30666	24937
Npas3	16232.5	10103	24328.75	20521
Npas4	38594	14426	23840	11001
Npat	37315	14671	22971.5	20964
Npb	19735	16763	23326	22504
Npbwr1	37170	870	9489	10636
Npc1	16302	33619.5	35769.5	33536.5
Npc1l1	2414	1902	18064	13902
Npc2	595	39378	1555	4416
Npdc1	36416	36042	4916	5572
Npepl1	35005	31008	25550	22343
Npepps	37840	32204.5	22743.5	25126.5
Npff	39267	40327	3129	3042
Npffr1	2673	39196	13503	13327
Npffr2	21531	4708.5	28501.5	10020.5
Nphp1	12221	14619	13030	14414
Nphp3	17250	27191.5	20600.75	16452
Nphp4	38863	36510	4959	366
Nphs1	17798	14323	16218	11130
Nphs2	27330	28597	31606	30088
Npl	34835	1992	30307	39150
Nploc4	28514	31676	26328	30957
Npm2	5541	5230	10487	10569
Npm3	16688	19334	19823	21958

Npn2	30308	26408	29365	27202
Npnt	22547	24875.33333	13060	21077
Nppa	14278.5	16521	20085.5	23200.5
Nppb	12643.5	14453	18012	16786
Nppc	21393	23836	27228	27673
Npr1	39490	38047	9717	4004
Npr2	25505.33333	27801.66667	16738.66667	28639
Npr3	23449.66667	21933.33333	25346.66667	11135.66667
Nprl2	6621	3749	2448	4823
Nprl3	22265.5	22060	21680	22880
Npsr1	17482	22332.66667	23934.33333	17210
Nptn	20438.25	18332.5	23543.75	19899.25
Nptx1	33866	31943	34038	32299
Nptx2	1596	39182	6538	33588
Nptxr	7830.5	10707	11097	10057
Npvf	33877	32922	418	7261
Npy	34209	19481	31133	22324
Npy1r	31212	36555	35811	36681
Npy2r	29336	26212	33157.5	27421.5
Npy4r	11313	10314	14679	14171
Npy5r	28876	30813	33031	31269
Npy6r	31459	33231	34617	41110
Nqo1	30567	22377	39239	32726
Nqo2	21558	17155.5	19669.5	14906.5
Nr0b1	30632	32253	33923	32167
Nr0b2	2936	2022	36431	4381
Nr1d1	20521	16725.33333	24236	20451.66667
Nr1d2	12675	5753	11456	9827
Nr1h2	31478	31508	26199	26334
Nr1h3	39024	38408	38034	35048
Nr1h4	21242	22849	28168	30454
Nr1h5	30131	5577	19557	40130
Nr1i2	12163	10807	9517	14460
Nr1i3	15616	13818	12220	14392
Nr2c1	23152	20480.66667	20992.33333	19778
Nr2c2	24417	14785.33333	15921.33333	16078.66667
Nr2c2ap	17775	20403	22100	22379
Nr2e1	28472	29801	36634	31453
Nr2e3	31986	33635	38729	33412

Nr2f1	27396	28722	31907	30303
Nr2f2	23137.5	32010.25	22196.5	21069.75
Nr2f6	28159	24065	26327	24609
Nr3c1	25236.66667	28487.66667	21818.66667	23376
Nr3c2	26899.5	20425.5	22487.75	30374.75
Nr4a1	33953	26795	31483	32415
Nr4a2	5038	40088	38561	26862
Nr4a3	28747	32853	33137	778
Nr5a1	22146.5	3221	12533.5	9775
Nr5a2	20320	38280	29946	26539
Nr6a1	10630.5	23950.5	8633.5	12818.5
Nradd	26609	35182	788	1028
Nrap	1259	4069	5592	8908
Nrarp	28431	29816	32282	30736
Nras	19969.5	18122	20759	20906.5
Nrbf2	16003.66667	16558.66667	16657	16438
Nrbp1	26761.5	24495.5	22857	24255
Nrbp2	27097.5	25333.5	11941.5	33191.5
Nrcam	22619.75	15017	17347.75	19967.5
Nrd1	22224	21638	20050	21774
Nrde2	40949	39463	36707	34576
Nrep	31040.33333	32658	33465.33333	23400.66667
Nrf1	27543.5	25916.5	22167	21341
Nrg1	14747	18756.16667	24480.5	17220.33333
Nrg2	13157	10471	3259	8204
Nrg3	28327.33333	29848.33333	32290	31482.33333
Nrg4	11434	12014	14847	13755
Nrgn	12246	6289	21309	20743
NRGN	27790	29267	34916	39355
Nrip1	18079.66667	15263.33333	15495	15498.33333
Nrip2	12105	11374	16160	13841
Nrip3	4329	5503	11733	10256
Nrk	18636.5	17574.5	21806.5	18597
Nrl	38346	4740	11129	10756
Nrm	27393	21895.5	26030	20153
Nrn1	30760	5752	5379	16826
Nrn1l	16756	16214	14784	12759
Nrp1	19326.75	22015	26697.75	17113.75
Nrp2	22470.5	30340.5	23890.5	34086.5

Nrros	3486	37725	1465	38781
Nrsn1	18061	19222	24650	24442
Nrtn	37459	32578	36264	36368
Nrxn1	23683.44444	24429.77778	21029.33333	25924.44444
Nrxn2	23865	24811	11604.5	7191
Nrxn3	28082.44444	26366.55556	27112.66667	22464.66667
Nsa2	25093.33333	22942.66667	24983.33333	22816.33333
Nsd1	19158.66667	8303.666667	9358.666667	22184.66667
Nsdhl	4042	7692.333333	24534	28904.66667
Nsf	7177.5	6371	38212.5	22603.5
Nsfl1c	8747	12930	13718	16796
Nsg1	27437	28101	31198.5	14289
Nsg2	30772	32809	33875	3487
Nsl1	17577	16620.5	19296.5	15116
Nsmaf	21420	22452.5	20628	20607.5
Nsmce1	29153	38875	39299	41161
Nsmce2	22746.66667	19888	19407.33333	20129
Nsmce4a	1450	1602	1252	39176
Nsmf	19813.33333	19619.66667	20437	19420.66667
Nsun2	15836	17320	19373	20541
Nsun3	37322	37787.5	2143.5	19874
Nsun4	30453.5	24918.5	23948	25155
Nsun5	5313	7957	7803.5	8562
Nsun6	12131.16667	15266	14903.83333	22077.16667
Nsun7	36734	37651	2705	6557
Nt5c	22263.5	25523	18430.5	21470.5
Nt5c1b	13930	13660	14597	16507.5
Nt5c2	15807.33333	13134.66667	14105	14348
Nt5c3	19833.5	27012.5	18085.5	28663.5
Nt5c3b	1523	2714	3874	4589
Nt5dc1	22236	25975	24265	27723.5
Nt5dc2	36576	29895	34022	28592
Nt5dc3	15390	21332.33333	27854.33333	22017.66667
Nt5e	26340.5	20532.5	30180.5	27924.5
Nt5m	13334	13443	11739	11185
Ntan1	40216	1779	38838	40583
Ntf3	29322	891	30321	38459
Ntf5	9948	3607	14037	15452
Nthl1	16462	23649	22208	24508

Ntm	29815.33333	24319.66667	22137	22283.5
Ntmt1	17919	20695	20509.5	22007
Ntn1	11828	25249.5	26275.5	13469
Ntn4	19425.5	19071	23058	26307.5
Ntn5	18871	21651	31165	29488
Ntng1	35236.75	12683.25	24481.5	24237.25
Ntng2	20320.5	20698	22006.5	10260.5
Ntpcr	28696.5	33638	27220.5	28369
Ntrk1	35519	37757	5770	2828
Ntrk2	14785.75	17820	12577	29906.25
Ntrk3	27486.66667	29211	26108.66667	17229.66667
Nts	18422	19693	20317	18350
Ntsr1	38712	40803	3355	1140
Ntsr2	20681	1389	11446.5	14255
Nuak1	16888	20168	20902.33333	20283.33333
Nuak2	15406	15799	19636	17898
Nub1	21994.66667	24176	21784	25686.66667
Nubp1	8514.5	11931.5	6730	9550.5
Nubp2	24084	27780	26561	31936
Nubpl	15812.66667	18017	18903.66667	18852.33333
Nucb1	19675.5	17552.5	17310.5	19059
Nucb2	33444	37255	34664	37316
Nucks1	15067.25	25239	18993	17605
Nudc	22304	21103	25862	27663
Nudcd1	14618.5	26559.5	16294	22841.5
Nudcd2	7356	12919	7730	8953
Nudcd3	26815.66667	23668.66667	26869.66667	24380
Nudt1	637	4533	5177	4467
Nudt11	30145.66667	23320.33333	25091.66667	25398
Nudt12	12225.5	19377.5	20243	19453
Nudt13	13336	20732	18639	18055
Nudt14	27133	901	24833	39145
Nudt15	3911	1592	34892	36016
Nudt16	22310	20816.33333	24000.66667	21461.33333
Nudt16l1	7791	23999.5	19015.5	18975
Nudt17	18281	20294	19215	20692
Nudt18	24327	27053	9550	11064.5
Nudt19	40722	1698	38338	35485
Nudt2	6638	14173	12242	13353

Nudt21	12273	13493.5	12900	12341
Nudt22	40622	40194	35257	36231
Nudt3	22839	22912	20893	20033
Nudt4	20996.33333	19588.33333	21901	21877
Nudt5	16204	29848.5	22533.5	29136
Nudt6	17177	15688.75	13622.5	21627.5
Nudt7	25805	26221.5	23532	24616.5
Nudt8	33628	2181	37983	2239
Nudt9	21900	21550	24044	25360
Nuf2	24630.5	24506.5	27483.5	24421.5
Nufip1	9721	10947	15260.66667	14652.66667
Nufip2	9660	7367.666667	9470.666667	9155.666667
Numa1	17906	9244	22253	10607.33333
Numb	17370.66667	19748.33333	20249.66667	7726.333333
Numbl	20078	18994	20337	23280
Nup107	20414	21919	20762	20671
Nup133	33438	29767	31331	29536
Nup153	10082.25	17129.25	7604.25	10413.75
Nup155	5297.5	10422	8124.5	14510
Nup160	19538.33333	19756.33333	14663	18132
Nup188	12721	12989.33333	15030.66667	16215
Nup205	21580.66667	18195.66667	15493	18598.66667
Nup210	17586	12120	15599	11531
Nup210l	33824	37117	11497	15816
Nup214	27061.5	27473.5	26288	28313.5
Nup35	28363	27933.5	28138	27163.5
Nup37	7287	13090	9749	9767
Nup43	17705.5	23166	19766	23680
Nup50	17650	11770	14796.33333	12641.66667
Nup54	22616.33333	25522	22560.66667	24957.66667
Nup85	38802	31292	38060	33699
Nup88	10546.5	13783.5	13029	15275.5
Nup93	21989	19995	21256	19921
Nup98	14052.6	26620.8	25611	27483
Nupl1	36570.66667	19594	32144.66667	19245.66667
Nupl2	14086.5	15517.5	16169.5	17079.5
Nupr1	21943	6760	27967	1674
Nupr1l	12979.5	13295	34154.5	27931
Nus1	5692	12395	12264	14069

Nusap1	33936.5	20516	20138.5	7173
Nutf2	11928	10875	8931	8061
Nutm1	33925	37317	31787	36121
Nvl	12895.5	10384.5	20395.5	22331
Nwd1	37056	23351	6763.5	5080
Nwd2	14154	28317	29640.33333	19878.33333
Nxf1	30639	27241.5	22052	25097.5
Nxf2	26990	4239	31395	4182
Nxf3	27963	29814	7509	1555
Nxf7	24606	25847	27474	26504
Nxn	29027.5	10700	29510.5	30856
Nxnl1	25663	1094	37833	4579
Nxnl2	17549	19856	27334	14195
Nxpe2	38596	37356	38597	33779
Nxpe3	31230	14643	35158	30959
Nxpe4	16925	10529	19437.5	16272.5
Nxph1	21998.25	33530.75	33569.5	28476
Nxph2	30018	36622	32967	35274
Nxph3	19102	17825	22194	20198
Nxph4	4364	8836	7828	10532
Nxt1	16133	23287.5	21257.5	24654.5
Nxt2	2900	5960	1958	3255
Nyap1	15682	16631	27070	21638
Nyap2	7453	13271	26823	29499
Nyx	27084.5	30545	32470.5	12147.5
Oaf	19953	28132	18207	26488
Oard1	19102.5	17853	21730	15843.5
Oas1a	34718	1537	35060	2010
Oas1b	30801	32417	33724	1316
Oas1c	1658	1355	4063	2768
Oas1d	8749	14658	11718	13780
Oas1e	1282	1103	6875	7937
Oas1f	22236	38279	28487	2695
Oas1g	1414	40163	4347	7382
Oas1h	21488	24076.5	23171	21220.5
Oas2	21811	15709	27199.5	18978
Oas3	18448	26871.5	22191	11977.5
Oasl1	10613	19503	14261	23801
Oasl2	19140	39870	23569	2982

Oat	2390	3179	4635	3162
Oaz1	36500.5	19810	19979	20295
Oaz2	29363.75	17667.75	28277	18641.25
Oaz3	14842	20345	26353.5	26441.5
Obfc1	24597	6745	23970	26622.5
Obox2	26891	28341	29726	28486
Obox3	35070	31984	35571	29491
Obox5	33111	35077	35955	33991
Obox6	37779	40463	29909	4685
Obp1a	38191	24566	14240	4441
Obp2a	18550	18046	34798	33015
Obscn	18511.5	34349.5	19987	21110
Obsl1	14478.33333	15031.66667	17996	21596
Oc90	5048	8358	11535	10123
Oca2	36130	3458	24855	10735
Ocel1	18309	17815	19750	18605
Ociad1	35859.5	37969.5	38051	39578
Ociad2	40510	40525	13407	3047
Ocln	33957.5	34900	12780.5	10804.5
Ocm	1261	37334	33190	38926
Ocrl	8058	23000.25	13643.5	22751.75
Ocstamp	8891	27555	21699	22031.5
Odam	31498	33235	34667	33346
Odc1	18201.33333	14433	20122.66667	19747
Odf1	21957.5	18272	24779.5	27915.5
Odf2	11961.75	10191.5	12428.75	11979.75
Odf2l	17076	18458	19081	20103.5
Odf3	20706	22762	24823	26303
Odf3b	127	1957	2956	2799
Odf3l1	4617	13348	24024	23651
Odf4	9182	10765	18331	19528
Ofcc1	12012	14830	17163	20939
Ofd1	16957	18495	16851	17789
Ogdh	15911.5	20475	13841	18938
Ogfod1	14147	13246	14884	13761.5
Ogfod2	8151.5	8141.5	7297	8212.5
Ogfod3	20546	32734	19748	27227
Ogfr	21771	25869	26450	30357
Ogfrl1	27085	26204	27376	24576

Ogg1	28212.5	30705.5	31807.5	30009.5
Ogn	40598	33574	26613	37710
Ogt	18593.33333	14324.66667	14700.66667	15696.33333
Oip5	1078	2632	40657	40508
Oit1	25035	29001	28411	36328
Oit3	12129	17478	12735.5	20428
Ola1	39743	1371	40958	38508
Olah	34405	27278	2667	28891
Olfm1	40141	27740	21758	23546
Olfm2	6971	27053	14659	11624.5
Olfm3	20062	20584	6468.5	1030.5
Olfm4	12513	12971	36274.5	20420.5
Olfml1	28252	26579	30898	39107
Olfml2a	22079	30053.75	31131.5	30501.25
Olfml2b	22586.5	19156.5	29977	28075
Olfml3	11215	14613	15737	15435
Olfr1	23453	24020.75	27094	26268.25
Olfr1006	9694	7533	9500	11773
Olfr1008	25395	26677	29184	38268
Olfr1009	5855	15734	12866	10678
Olfr101	23573	24632	26600	25544
Olfr1010	30165	34613	37364	39987
Olfr1012	26544	27919	29762	28367
Olfr1014	38395	6725	11096	16397
Olfr1015	29985	37996	32470	8028
Olfr1016	39527	40000	8391	18872
Olfr1018	39642	2321	3356	6187
Olfr1019	15373	18059	39605	39056
Olfr102	18599	21755	26011	27990
Olfr1020	29504	36418	8431	2782
Olfr1022	1903	3646	8655	7579
Olfr1023	31613	34028	621	32652
Olfr1026	4237	6972	10135	9644
Olfr103	3155	8168	8373	9163
Olfr1030	16866	19401	20118	20698
Olfr1032	29330	34397	36904	36872
Olfr1036	24336	9948.5	14486.5	15023
Olfr1038-ps	29194	4377	34029	783
Olfr1039	1092	31618	31609	31049

Olfr1043	8719	10412	14668	15827
Olfr1044	4971	16365	23094	23062
Olfr1045	28953	2509	30383	29203
Olfr1046	38912	37896	37615	25982
Olfr1047	31999	38817	9513	12697
Olfr1048	4339	6742	14911	15059
Olfr1049	40547	8439	16153	7756
Olfr104-ps	38011	35701	302	26168
Olfr1052	23056	31121	27390	39747
Olfr1054	807	2590	550	489
Olfr1055	27141	35498	34819	36479
Olfr1056	5655	8174	8939	9760
Olfr1057	30905	32591	34435	32636
Olfr1061	29778	39043	36840	33701
Olfr1065	7808	11288	7927	11691
Olfr1066	21957	29101	34646	33109
Olfr107	17607	20374	26873	27272
Olfr1076	25759	30446	28252	27252
Olfr1079	25760	27422	27746	26689
Olfr108	24630	30165	32243	32702
Olfr1080	25515	25012	27235	26073
Olfr1086	13283	17553	19047	15244
Olfr1087	14636	14645	18304	25478
Olfr109	23426	24499	26460	25345
Olfr1090	21128	24710	32904	38175
Olfr1093	336	3928	757	41099
Olfr1094	27013	28220	30319	29029
Olfr1095	36511	38206	39624	33656
Olfr1097	14426	9171	23405	20823
Olfr1099	22329	23950	94	292
Olfr110	39083	3084	38646	3598
Olfr1100	24863	26200	27410	26283
Olfr1101	36120	30602	2152	39925
Olfr1102	28324	30023	30207	29069
Olfr1104	35276	5363	6647	14533
Olfr1106	6079	13711	17848	15307
Olfr1107	12920	14970	15647	17430
Olfr1109	15650	21728	27101	25201
Olfr1110	29495	35881	31386	30045

Olfr1111	17904	17839	19538	23672
Olfr1112	3207	5304	9675	11525
Olfr1115	34185	38124	93	1982
Olfr1118	14147	18890	24138	23275
Olfr112	23930	29980.5	20560	9542.5
Olfr1120	25723	27289	27850	26861
Olfr1121	34121	35525	38642	38214
Olfr1122	13655	15109	19331	19095
Olfr1123	35325	38873	8173	9886
Olfr1124	26641	27968	30746	28601
Olfr1126	12456	13851	18589	18082
Olfr1128	31395	36282	34597	32839
Olfr113	21277	26092	25678	27322
Olfr1130	18439	24028	25718	37065
Olfr1131	19081	22485	24812	26843
Olfr1132	33061	35169	36236	37147
Olfr1133	27937	29412	30760	29713
Olfr1134	11530	14982	17705	18416
Olfr1135	27215	28921	29151	27954
Olfr1136	34793	27185	28189	27036
Olfr1138	26548	34788	33680	2484
Olfr114	30278	37940	6874	8720
Olfr1140	15774	21250	29910	27949
Olfr1141	29948	30918	28649	27589
Olfr1143	14314	17124	16919	16735
Olfr1145	15765	34849	33870	1382
Olfr1148	35465	40089	7533	5785
Olfr1151	37660	36404	9408	7523
Olfr1153	20243	35961	23030	31329
Olfr1154	18583	21039	29144	27873
Olfr1155	27507	40900	2557	510
Olfr1156	23891	29885	33965	35475
Olfr1157	20405	14986	5598	4545
Olfr1158	31054	32674	34215	32511
Olfr1160	30916	30706	32247	30711
Olfr1161	14140	16768	19249	20308
Olfr1163	39452	39184	8469	6047
Olfr1164	32825	37686	30257	29328
Olfr1165-ps	15997	22444	25371	31148

Olfr1166	3764	10948	7547	11464
Olfr1167	9773	15945	21595	21453
Olfr1168	31936	34330	37570	33470
Olfr1170	29266	36293	5052	2469
Olfr1175-ps	19263	18660	28695	30768
Olfr1176	4702	12445	12009	18189
Olfr1178	37238	6712	14440	12770
Olfr1179	24597	26553	7582	10414
Olfr1180	20246	22150	26989	33276
Olfr1181	14442	15972	19416	20573
Olfr1182	7200	12226	15325	19520
Olfr1183	8226	13881	15058	16847
Olfr1184	28119	38192	5402	5236
Olfr1188	18173	12794	34176	36263
Olfr1189	21309	19205	20440	22968
Olfr1193	32968	38882	36158	38890
Olfr1195	20713	18820	27294	29901
Olfr1196	7392	14004	14447	14639
Olfr1198	31599	39474	38162	423
Olfr1199	2772	9915	35691	3388
Olfr12	5576	8268	10536	10785
Olfr120	13575	15843	21731	21576
Olfr1202	23168	24233	37492	2638
Olfr1204	25819	27248	2501	27219
Olfr1205	20562	22701	36908	24815
Olfr1206	16440.33333	18818	24235	24270
Olfr1208	20987	16645	18540	17479
Olfr1209	1427	2874	8798	12831
Olfr1211	1031	6459	6292	6900
Olfr1212	32848	38354	18470	17708
Olfr1214	25583	26725	28980	27774
Olfr1215	9631	16057	18573	19358
Olfr1216	4475	39122	7769	6920
Olfr1217	13488	12270	17193	20827
Olfr1218	8924	14834	13935	15831
Olfr1219	21175	30752	39307	39112
Olfr122	3198	10373	17810	15322
Olfr1220	33096	35082	36498	34346
Olfr1221	4254	9940	15820	15253

Olfr1222	34916	36986	3478	2213
Olfr1223	24246	23825	36178	40353
Olfr1225	17982	23496	21553	31098
Olfr1226	16724	20720	25568	27020
Olfr1228	32469	34327	38177	3555
Olfr123	2278	1665	10652	6644
Olfr1230	30751	25428	1102	39960
Olfr1231	16138	17754	22953	21134
Olfr1232	40541	7659	8360	7195
Olfr1233	25856	27171	28686	27809
Olfr1234	26978	41069	175	35027
Olfr1238	32388	34072	3695	10669
Olfr1239	30901	38870	92	40664
Olfr124	9735	9518	11976	13270
Olfr1240	36869	1523	2477	4210
Olfr1241	30816	32983	33105	31480
Olfr1242	37622	38728	31381	39957
Olfr1243	40508	7376	12006	9843
Olfr1245	28354	32699	39413	407
Olfr1246	18706	18378	22486	19877
Olfr1247	25363	26552	28725	27491
Olfr1248	1240	7764	7676	6058
Olfr125	21369	23588	38506	32433
Olfr1250	31008	32327	33948	32307
Olfr1254	26018	27147	29878	28544
Olfr1255	6310	6836	11260	11175
Olfr1256	19154	20612	28932	27923
Olfr1258	30145	37088	4865	4794
Olfr126	20350	21376	29730	37422
Olfr1260	36345	2102	8568	10413
Olfr1261	27686	29450	29567	28306
Olfr1262	32083	28756	29232	33914
Olfr1263	39080	585	5231	5513
Olfr1264	29581	31055	33330	31717
Olfr1269	1325	3359	13014	12856
Olfr127	14361	16967	13519	15645
Olfr1270	28802	32563	3948	4349
Olfr1271	10804	11169	10997	10307
Olfr1272	12614	13937	20143	22209

Olfr1276	40054	1694	8194	8248
Olfr1277	32841	24606	38286	33696
Olfr1278	25036	26290	28521	36182
Olfr1279	40487	33191	33289	32090
Olfr128	34374	25498	33403	24974
Olfr1280	7733	12042	13028	14795
Olfr1281	3597	400	37158	431
Olfr1282	38095	653	6521	5995
Olfr1284	7452	11437	12212	11864
Olfr1286	37061	28256	31417	35156
Olfr1288	39851	12649	9033	17539
Olfr1289	13638	16193	10253	12359
Olfr129	27800	31949	39494	1815
Olfr1290	18238	25610	25560	28032
Olfr1294	33225	4037	31242	30269
Olfr1295	11368	19083	19669	22748
Olfr1297	30270	31890	33795	38281
Olfr1298	6631	15841	20823	21228
Olfr1299	14588	16599	843	9396
Olfr13	32241	2762	11607	11703
Olfr130	28535	31104	40271	512
Olfr1301	25528	26981	27965	26970
Olfr1302	4554	9809	6727	9992
Olfr1303	40601	2105	8878	8952
Olfr1305	6531	9881	12702	13953
Olfr1306	39290	8226	6678	13952
Olfr1307	11410.5	16372	22583	23532.5
Olfr1308	32928	36633	36101	34057
Olfr1309	22900	23023	35007	35644
Olfr131	23459	35882	28460	29273
Olfr1310	25012	26263	28069	27031
Olfr1311	31779	33413	35618	37992
Olfr1312	9874	18564	14005	20873
Olfr1313	35049	35429	11193	16875
Olfr1314	9195	19077	31126	29744
Olfr1316	4413	3448	9977	6746
Olfr1317	40754	3383	6275	6890
Olfr1321	14048	13092	36515	29910
Olfr1323	36590	20771	14607	11091

Olfr1324	30094	31684	33124	31755
Olfr1325	37799	4814	15013	21280
Olfr1328	35012	977	5418	6217
Olfr1330	27810	29393	30843	32919
Olfr1333	9490	7525	16331	11566
Olfr1336	18888	19100	37768	7854
Olfr1337	37727	407	39094	1278
Olfr1338	30025	2131	12273	9112
Olfr1339	15746	18744	19590	21247
Olfr134	34304	4952.5	16986.5	19099.5
Olfr1340	32705	34188	38071	4818
Olfr1341	5833	6421	17436	18866
Olfr1342	7365	10243	15480	15851
Olfr1344	20480	24782	22800	24383
Olfr1346	25344	27768	26350	28925
Olfr1347	25356	36640	28972	36316
Olfr1348	35810	39386	660	1539
Olfr1349	40635	1626	292	951
Olfr135	25726	34403	27014	35260
Olfr1350	22736	22938	25240	24309
Olfr1351	29398	26897	28453	31227
Olfr1352	30402	791	11484	13940
Olfr1353	32525	33909	37578	5423
Olfr1355	19100	23286	28346	27689.5
Olfr1356	18525	20757	23433	24296
Olfr1358	23208	32831	38468	39157
Olfr1359	22138	27981	27114	27501
Olfr136	33862	36496	128	1744
Olfr1360	19340	24677	26090	26912
Olfr1361	38420	3840	13667	10890
Olfr1362	21498	21658	26726	28124
Olfr1364	6857	10989	15423	16940
Olfr1366	4937	4959	14425	13338
Olfr1367	36097	35734	41001	7970
Olfr1368	27438	32513	33106	33945
Olfr137	24935.5	29061	17629.5	36956.5
Olfr1370	26086	27301	30617	25
Olfr1371	29114	13374	17731.5	17482.5
Olfr1372-ps1	15727	19161.5	24344.5	20722.5

Olfr1377	15098	10590	24143	21952
Olfr1378	9214	18320	11782	16462
Olfr138	26274	27651	29104	27930
Olfr1381	2270	2977	10522	9538
Olfr1383	34627	3595	859	6984
Olfr1384	18435	23294	24410	22323
Olfr1386	9508	5959	10980	8039
Olfr1387	39181	39554	5884	5946
Olfr1388	28963	29323	36437	36708
Olfr1389	31631	33069	39712	32841
Olfr139	36279	39972	2748	4048
Olfr1391	5791	10125	19551	18322
Olfr1393	18724.5	19280.5	7326.5	25808.5
Olfr1394	6993	10809	12984	13051
Olfr1395	13700	15166	19115	19648
Olfr1396	29847	7939	3329	8397
Olfr140	27984	35724	34583	5420
Olfr1402	8030	11086	14662	14382
Olfr1404	35952	39752	39753	40364
Olfr1406	9032	11494	12324	12085
Olfr1408	13447	21319	25891	27729
Olfr1410	8774	15181	18195	17897
Olfr1411	12741	11615	22717	23158
Olfr1412	8623	10631	9791	16864
Olfr1413	29162	32014	38278	1413
Olfr1414	15305	19677	22980	23632
Olfr1416	25594	27187	27968	31637
Olfr1417	15310	17810.5	25471	24881
Olfr1419	17136	19690	21626	22867
Olfr142	29735	31307	32715	31388
Olfr1420	27238	32356	37749	40452
Olfr1423	24221	25331	3452	537
Olfr1424	6998	13426	27733	24420
Olfr1426	25837	27164	36643	34795
Olfr1427	30175	36970	4470	6804
Olfr1428	12863	18556	24387	23140
Olfr143	13002	17190	18710	14280
Olfr1431	15954	20231	36816	35209
Olfr1434	23677	24698	26447	40016

Olfr1436	33625.5	34235.5	3510.5	17155
Olfr1440	31421	33120	35319	38485
Olfr1441	12404	15198	21602	22076
Olfr1442	30795	14002.5	21519.5	21246.5
Olfr1443	16884	18562.5	22770.5	21843
Olfr1444	40550	36391	3124	39149
Olfr1445	8495	13157	19683	21596
Olfr1446	8191	19872	12921	15977
Olfr1447	16007	12904	15287	19627
Olfr1448	18756	22180	21639	17048
Olfr1449	25771	26878	29171	38888
Olfr1450	26402	34392	34783	36052
Olfr1451	22759	24809	29222	24719
Olfr1453	30613	38653	41007	305
Olfr1454	34955	5387	38127	4293
Olfr1457	29033	30530	32792	1311
Olfr1459	10571	14108	16622	16590
Olfr146	4107	349	9056	8556
Olfr1462	2881	36349	4545	34628
Olfr1463	6257	5780	40879	6707
Olfr1466	5087	9399	12157	13111
Olfr1467	36597	3316	18164	15691
Olfr1469	20259	18863	20597	22251
Olfr1471	17239	17555.5	25033.5	26519.5
Olfr1472	11890.5	12099.5	14600.5	17130.5
Olfr1477	38363	5057	14580	14597
Olfr148	3528	3421.5	11387	13705
Olfr1484	30867	40155	33342	36889
Olfr1487	29608	29158	5556	2700
Olfr1489	31653	33332	35173	33435
Olfr149	11716	16224	16149	16370
Olfr1491	1751	6007	8334	8298
Olfr1494	37943	4512	6450	1655
Olfr1495	13630	15696	21800	20986
Olfr1496	36354	4554	13281	15729
Olfr1497	21574.5	19076	28300.5	9606
Olfr1499	11108	37484	12981	22978
Olfr15	37814	5414	3335	5925
Olfr1500	21774	26805	35292	37086

Olfr1501	39930	2005	5753	6166
Olfr1502	30565	32113	37853	38674
Olfr1504	22406	19935	25121	24260
Olfr1507	5223	6932	9648	9110
Olfr1508	21390	22080	25785	33959
Olfr1509	13450	11834	17252	15109
Olfr1510	39563	37564	10945	5784
Olfr1511	26715	34656	987	39099
Olfr1512	22130	34145	36188	37296
Olfr152	12968	9970	11297	7047
Olfr153	22320	23551	25794	25440
Olfr1532-ps1	40521	30946	1638	39986
Olfr1537-ps1	37346	40825	4832	3115
Olfr154	17501.5	17456.5	19393.5	23263
Olfr155	30560	37176	33620	32250
Olfr156	31665	27520	30495	29111
Olfr157	28268	10	556	2878
Olfr159	30365	28333	37161	39412
Olfr16	27201	28368	30702	29370
Olfr160	20259.5	22255	26936.5	25234
Olfr161	11602	15811	16067	16387
Olfr164	1650	8942	8093	12246
Olfr165	2171	7081	20882	20179
Olfr166	13485.5	15830	20224	20878
Olfr167	21259	24731	28827	31365
Olfr17	33584	35566	40998	40678
Olfr170	18910	19209	22179	23440
Olfr171	37416	40842	9156	40694
Olfr172	4316	2736	10235	14991
Olfr173	40524	29306	30862	29234
Olfr175-ps1	11724.5	16306	20026.5	19482
Olfr176	29404	30877	32661	33485
Olfr177	25013	25379	35927	38409
Olfr178	29947	32019	31760	30301
Olfr18	27054	28184	30651	29307
Olfr180	12610	13218	17421	20013
Olfr181	21307	24444	34628	36876
Olfr183	22455	22302	28844	26664
Olfr186	5514	7751	12566	15913

Olfr187	39048	3404	2302	5667
Olfr19	7171	12718	14517	18904
Olfr190	3553	7759	11239	12822
Olfr193	29406	31027	33138	37158
Olfr195	21122	22735	32293	33116
Olfr196	1188	11825	11054	14511
Olfr197	18631	20180	20952	19707
Olfr2	21434	19620	26673	23148
Olfr20	25055	26141	32440	35984
Olfr201	24614	25902	28061	26285
Olfr202	30857	6538	33856	4615
Olfr203	39888	1194	1169	1120
Olfr206	17232	21612	25575	27832
Olfr208	25776	26586	39993	34094
Olfr211	24705	25907	33431	36839
Olfr212	38578	2568	40318	3604
Olfr214	33942	40026	3257	4635
Olfr215	28745	37227	31959	1567
Olfr218	35545	68	6381	3462
Olfr221	39898	6856	3780	6509
Olfr223	6686	10933	10074	11610
Olfr225	36158	34736	40216	1507
Olfr228	11093	17987	16128	19122
Olfr229	11882	13768	20217	20990
Olfr22-ps1	37883	37056	40485	39894
Olfr23	28185	4910	31900	1972
Olfr231	14446	14816	20104	18135
Olfr235	35453	836	30463	121
Olfr237-ps1	15613	21426	815	40489
Olfr239	40859	41021	7297	6157
Olfr24	17993	18427	22658	22130
Olfr242	502	33544	30934	9805
Olfr247	3179	7468	8610	9773
Olfr248	10855	19727	19892	21478
Olfr25	39031	1196	2312	4480
Olfr257	13181	13620	18920	21860
Olfr259	32685	36182	507	3842
Olfr26	38637	2460	2412	3206
Olfr262	4150	7927	3249	13672

Olfr263	18032.5	27574.5	29519	12319
Olfr266	14079	22005	20833	25997
Olfr267	23447	24513	26510	25384
Olfr27	25868	24081	36760	33748
Olfr270	8575	11511	20886	15395
Olfr272	35750	40015	816	38200
Olfr273	23344	24414	26608	33650
Olfr275	12682	13511	13347	19119
Olfr279	21785	24259	25864	6995.5
Olfr281	15408	17472	20301	17419
Olfr282	18659	22060	25519	29765
Olfr283	17530.5	23883.5	5756	8087
Olfr284	29895	33590	32875	31173
Olfr288	4896	10806	1761	8881
Olfr291	24690.5	28106.5	12795.5	12026.5
Olfr295	18700	33106	25266	28932
Olfr297	2455	7272	8059	9976
Olfr299	35680	5939	13013	12947
Olfr29-ps1	39992	6010	4216	6816
Olfr30	37027	40151	4017	6818
Olfr303	719	37703	8960	9400
Olfr304	14310	14646	22338	23722
Olfr305	32767	34063	37443	35471
Olfr307	31732	33455	34849	33079
Olfr308	27122	28821	28867	30153
Olfr309	20435	21992	24722	24859
Olfr31	25383	24106	29919	28535
Olfr313	4045	5112	11661	10673
Olfr315	21023	20561	24464	26926
Olfr318	29624	27493	39834	39058
Olfr319	9201	13999	16867	17910
Olfr32	9958	10458	16227	14857
Olfr320	17816	23290	41048	40377
Olfr323	8782	25717	7970	27329
Olfr33	30152	36338	2015	1666
Olfr330	32959	34594	36841	34964
Olfr332	9450	8960	14928	13213
Olfr338	38058	387	5147	4748
Olfr339	39985	7464	3612	9784

Olfr340	8262	15689	20659	20051
Olfr341	35068	35616	2565	1894
Olfr342	25568	29708	38022	39750
Olfr344	35757	37481	5435	4543
Olfr345	2737	5233	14965	11257
Olfr346	39672	606	4124	2426
Olfr347	39762	40622	8508	7995
Olfr348	759	36588	39682	3314
Olfr350	22427	25183	34981	40023
Olfr351	17885	21015	24038	21546
Olfr352	36253	2672	8134	9610
Olfr353	31290	27105	24277	35028
Olfr354	33667	26066	2397	39275
Olfr355	21389	23107	31317	33843
Olfr356	39950	35018	37633	6751
Olfr357	17291	20351	23961	24065
Olfr360	12762	18167	19480	23072
Olfr361	11329	13626	22856	19412
Olfr362	40964	1342	2187	438
Olfr365	11488	10921	14695	12585
Olfr366	13487	15491	15234	19169
Olfr368	10744	11890	20391	19553
Olfr370	2357	37339	10005	10071
Olfr373	28014	28218	37299	36509
Olfr374	10426	12694	17227	18075
Olfr378	30064	31798	2387	30917
Olfr38	2349	2398	6720	6545
Olfr380	30257	4110	39664	5171
Olfr381	4046	10558	18924	13177
Olfr382	5921	10536	15815	14438
Olfr384	8137	15389	19388	21452
Olfr385	36280	8899	5968	17007
Olfr389	14505	14770	37859	27521
Olfr39	29258	37045	29786	28654
Olfr390	39660	33151	39892	33866
Olfr392	26481	28070	29168	27973
Olfr393	29393	29358	31203	679
Olfr394	30154	10480	12890	16714
Olfr395	30253	37802	32815	31361

Olfr397	22761	34604	29183	33986
Olfr398	12226	16238	20624	20490
Olfr399	7019	11073	14798	15648
Olfr401	208	3787	2362	4425
Olfr402	27400	28991	29912	28614
Olfr406-ps	30849	27504	36812	32929
Olfr410	3596	2967	9394	8879
Olfr411	2039	4100	8497	9083
Olfr414	22293	23356	25945	26627
Olfr418-ps1	10451	9530	14666	13930
Olfr419	2410	5313	10181	10285
Olfr420	694	4623	3991	3780
Olfr421-ps1	35222	32565	38348	38448
Olfr424	13067	15263	19223	23279
Olfr427	26752	32070	1182	412
Olfr429	17185	15567	23240	24637
Olfr43	23736	25515	26219	25715
Olfr430	18824	30781	18873	22489
Olfr432	39978	33936	33933	32256
Olfr433	9317	26804	13768	36528
Olfr434	2064	5993	10318	11647
Olfr435	24560	38155	1570	972
Olfr437	3355	8366	13652	18395
Olfr44	28017	29156	40654	960
Olfr441	15679	16835	18965	17813
Olfr444	27534	28619	37309	38079
Olfr446	26112	24135	35637	36074
Olfr447	37886	36869	40298	40032
Olfr448	12999	10799	21857	24256
Olfr449	37940	38193	390	1935
Olfr45	29879	37697	32162	35225
Olfr450	35548	38130	5104	7835
Olfr456	27692	36488	5253	6212
Olfr457	5866	8418	13726	13678
Olfr458	14844	14328	21865	20917
Olfr459	2369	612	6883	10603
Olfr46	8203	10492	17095	10517
Olfr460	15757	19837	20287	24602
Olfr461	40298	40460	36804	2993

Olfr462	20138	19044	21580	22733
Olfr464	29390	30844	33107	31515
Olfr466	37916	4988	33098	40342
Olfr469	18666	20988	22027	23286
Olfr47	16536	13666	19595	21829
Olfr470	10848	15690	20944	21429
Olfr472	27883	39633	31781	30271
Olfr473	28608	30575	38315	1960
Olfr474	496	3053	31648	3303
Olfr476	15458	16407	21646	19387
Olfr478	37998	36799	2824	183
Olfr48	15673	16052	28030	30748
Olfr480	33214	34834	37081	35235
Olfr481	11477	13554	22674	31354
Olfr482	5428	16545	23156	28089
Olfr483	14620	18562	23944	24124
Olfr485	6390	10494	10631	12741
Olfr486	19443	24178	40947	40077
Olfr487	25679	31700	40612	38202
Olfr488	35343	3346	37792	12403
Olfr49	1009	32615	34055	8848
Olfr490	32512	38775	35280	33449
Olfr491	2630	23149	4076	9194
Olfr492	29961	32043	32446	38953
Olfr493	18260	21198.5	25187	28740.5
Olfr494	40363	5254	4541	4627
Olfr495	683	40260	10940	11976
Olfr497	37733	410	6552	5814
Olfr498	31068	32757	34398	32597
Olfr5	21600	25941	2820	3917
Olfr50	28318	39524	29827	28579
Olfr502	37848	1500	3125	2996
Olfr507	26250	29618	37841	35326
Olfr508	16101	19588	17039	18569
Olfr509	17069	12822	26399	24915
Olfr51	10665	11094	8897	8080
Olfr510	20198	23449	31102	40756
Olfr512	38097	26547	4973	3734
Olfr513	10066	12010	21660	21622

Olfr514	33535	28590	28342	34841
Olfr516	20310	22376	20266	21498
Olfr518	28874	29025	35113	34353
Olfr519	8830	6817	14496	17609
Olfr52	27970	37286	1505	726
Olfr520	9435	9241	40857	10130
Olfr521	13028	13635	19500	19228
Olfr522	33611	35658	38346	39500
Olfr523	34747	33951	39349	37859
Olfr524	10818	14595	17894	19089
Olfr53	29544	29974	40190	38060
Olfr530	3530	2811	9924	8526
Olfr531	23754	24188	32239	31124
Olfr533	34286	26802	40888	4676
Olfr535	22009	20937	23534	24615
Olfr536	27978	32993	39165	38406
Olfr539	10371	12357	23462	21110
Olfr54	6777	13568	11641	13956
Olfr541	24582	103	33187	36794
Olfr544	23755	29033	35083	36001
Olfr545	27468	28828	31008	29507
Olfr547	31778	35142	35223	33257
Olfr549	37761	37636	40252	38295
Olfr550	34184	23227	38919	34392
Olfr551	37353	1217	11554	18626
Olfr552	39205	36617	32733	35767
Olfr554	38460	4659	7963	12150
Olfr555	36469	38866	40859	1800
Olfr556	32025	33619	34983	33185
Olfr557	35273	37012	40276	39186
Olfr558	19361	25439	24910	25796
Olfr559	15685	18162	21875	22292
Olfr56	40195	40134	2687	2515
Olfr560	25923	31633	29450	33224
Olfr561	29215	30741	33345	31615
Olfr564	1543	38612	37567	40705
Olfr568	37420	8780	4803	3786
Olfr569	802	5086	9222	9853
Olfr57	7908	12753.5	21912	20015

Olfr570	22901	28750	35938	37028
Olfr571	17420	17842	20186	20553
Olfr572	38413	39219	39495	40396
Olfr574	8023	13929	12282	14241
Olfr575	4410	40776	7848	12471
Olfr576	20718	23796	24651	29093
Olfr577	15854	17050	19825	18974
Olfr578	38510	38411	4005	4345
Olfr58	31831	33628	37448	35205
Olfr583	29049	30713	35387	30142
Olfr584	31181	34542.5	3349	3875.5
Olfr585	29975	31993	31868	30455
Olfr586	15578.5	18604.5	18927	23820.5
Olfr589	29678	31263	37434	34234
Olfr59	30557	31394	34598	35415
Olfr591	13298	13337	18990	22583
Olfr593	27983	29325	31800	30284
Olfr594	33602	73	7701	11830
Olfr598	7066	10769	11640	12917
Olfr599	20873	25556	26938	31117
Olfr60	10483	16750	19288	21180
Olfr600	6864	1964	29799	28432
Olfr601	23523.5	25785	27915.5	27609
Olfr603	4557	8866	8230	8331
Olfr606	23675	33796	39141	37983
Olfr608	4630	6874	11013	11684
Olfr609	7561	9697	15601	16207
Olfr61	34251	6769	8583	23529
Olfr610	35679	20744	18093.5	1120.5
Olfr611	18116	22889	27457	31810
Olfr613	29772	31542	31892	30494
Olfr615	2809	6370	12619	13194
Olfr616	18741	20788	23908	28261
Olfr617	37485	39615	7247	4108
Olfr618	16036	19290	25156	23888
Olfr619	32273	39857	40054	447
Olfr62	6168	8355	10098	9448
Olfr620	37409	39290	4116	3211
Olfr622	7377	11329	14363	15483

Olfr623	18868	22185	25668	29174
Olfr624	19073	24780	27840	31917
Olfr628	36084	2882	31020	29668
Olfr629	512	8332	13854	12719
Olfr63	39817	1577	11472	11448
Olfr630	14926	20072	31566	27242
Olfr631	35646	24901	36448	32350
Olfr632	31043	36769	15007	14254
Olfr633	7787	10385	5862	12092
Olfr635	18544	21589	23905	25036
Olfr638	835	845	7645	2376
Olfr639	29045	30469	32212	30656
Olfr64	30406	23967	38246	35463
Olfr640	6240	11462	18793	19499
Olfr641	15222	13664	26420	34205
Olfr642	8691	12115	16525	17802
Olfr643	12666	16315	17266	19310
Olfr644	18801	23256	24170	24903
Olfr645	21736	27085	37088	37945
Olfr646	10328	7733	13218	16037
Olfr648	7159	8924	11121	10188
Olfr649	10650	13634	19735	20205
Olfr65	11552	18363	17098	17963
Olfr651	23718	29796	25952	31721
Olfr652	12984	10441	21201	22427
Olfr653	4038	5446	9264	12178
Olfr654	6505	2560	21163	19770
Olfr655	27768	24041	33682	35544
Olfr656	34302	36705	36689	38077
Olfr657	8872	9383	17381	14447
Olfr658	19678	20538	23854	24587
Olfr659	13024	11377	17176	9697
Olfr66	23870.33333	12212	13938.33333	14020.66667
Olfr661	38615	3152	40589	742
Olfr665	39868	6736	15151	16576
Olfr667	33936	30916	781	40103
Olfr668	2425	5910	13062	12707
Olfr669	19128	16873	26099	30044
Olfr67	2943	2696	7964	8633

Olfr670	23588	24681	26466	25362
Olfr672	21188	24869	21824	16898
Olfr675	23164	36928	28792	35268
Olfr676	6604	10873	18137	18487
Olfr677	29361	39696	32472	40877
Olfr678	23889	25103	26484	25428
Olfr679	34123	1615	669	3240
Olfr68	15320	21883	30677	35910
Olfr683	24514	25575	27900	26708
Olfr684	9333	14651	16175	17535
Olfr685	24209	26112	25871	25013
Olfr686	31639	38126	4500	5224
Olfr689	20500	24313	38681	38421
Olfr69	23660	39289	3814	32706
Olfr690	25659	23790	22640	25035
Olfr691	4719	7362	6264	6834
Olfr692	33529	33094	1905	2335
Olfr693	8243	11673	15114	15833
Olfr694	30399	15418	22827	22947
Olfr695	36498	36854	6803	30497
Olfr697	14968	14627	16120	15291
Olfr698	40353	4212	658	6266
Olfr70	3924	36448	3112	1718
Olfr700	22670	22549	37219	36748
Olfr701	28536.5	13986	18478	17540.5
Olfr702	28254	3863	34762	29970
Olfr703	18168	18417	16193	10578
Olfr705	14069	8543	7811	12404
Olfr706	12783	18225	20592	21302
Olfr71	5020	11337	14104	18408
Olfr710	32864	34607	36615	36914
Olfr711	20348	32653	25033	30694
Olfr713	22908	23930	31496	28742
Olfr714	26167	27300	38399	7253
Olfr715	21687	20675	33354	36250
Olfr716	21910	25765	37789	35570
Olfr720	37397	30380	27951	2955
Olfr722	10609	17857	23386	18945
Olfr724	35791	36554	36354	1086

Olfr725	32305	33602	32353	25606
Olfr726	5156	5095	12169	10811
Olfr727	14716	17691	24458	21125
Olfr729	23427	28892	32346	25823
Olfr73	33799	37490	33347	37141
Olfr730	32360	34650	40906	2107
Olfr731	32577	3513	38387	11580
Olfr732	11578	14411	22821	24211
Olfr733	26132	34290	38128	37991
Olfr734	32627	27126	8499	9012
Olfr736	13440	10727	19255	10150
Olfr738	37176	106	7134	8063
Olfr739	38589	858	9340	11746
Olfr74	9469	13656	17957	21051
Olfr740	18594	22456	25546	28545
Olfr741	3258	12054	10813	11842
Olfr744	39362	879	4674	2877
Olfr745	16676	13878	25340	30216
Olfr746	34061	40502	35751	14978
Olfr748	30507	39288	35184	461
Olfr749	22689	31877	34474	37841
Olfr76	5845	9781	13105	13176
Olfr763	18692	16950	21643	20394
Olfr765	17763	17792	24172	28788
Olfr768	7481	39789	15603	12929
Olfr769	3794	36371	30747	38326
Olfr77	13051.66667	16481	20138.66667	19969.33333
Olfr770	1203	6992	10343	9362
Olfr771	34548	28166	1509	2568
Olfr772	41078	1130	720	1284
Olfr774	78	10189	20856	22835
Olfr775	28037.5	11035	14586.5	13608
Olfr78	3377	2150	14502	7999
Olfr780	3332	9231	9904	8199
Olfr781	3387	39599	31695	40443
Olfr782	21198.66667	15003.66667	20034	25591.33333
Olfr784	32542	35802	13099	11220
Olfr786	5313	7892	11126	12833
Olfr788	19925	19380	26222	24080

Olfr790	40370	2765	996	2467
Olfr791	35953	1015	4617	2895
Olfr794	27636	29054	30323	29047
Olfr796	518	968	3083	1119
Olfr798	8439	5665	14589	16966
Olfr799	39514	2339	3421	6361
Olfr8	33025	35962	40489	38055
Olfr800	23837	24806	30175.5	9719.5
Olfr801	27380	27364	33203	33886
Olfr802	1326	30927	40992	2727
Olfr803	23302	36602	26882	1988
Olfr805	19398	20949	33797	35561
Olfr806	27753	29065	31814	30310
Olfr807	5830	8015	15119	15544
Olfr808	15397	19733	23272	29299
Olfr809	32532	40785	5044	10651
Olfr810	19621	22681	24332	27948
Olfr811	25672	26784	29411	28136
Olfr812	40041	32620	38693	30414
Olfr815	15985	18927	23563	23923
Olfr816	29784	31623	31712	30406
Olfr818	30023	32111	31861	30395
Olfr820	30824	32323	33894	32161
Olfr821	3765	12160	10966	11131
Olfr822	6312	13050	22903	19776
Olfr823	311	35067	1726	6063
Olfr825	26616	26710	37102	39735
Olfr826	37640	5615	5042	8343
Olfr827	24405	25623	27152	26026
Olfr828	4450	8031	17791	17788
Olfr829	34850	34395	39060	8400
Olfr830	6640	12772	6279	8118
Olfr832	33316	3347	802	7297
Olfr835	38197	41046	4779	4905
Olfr836	33826	5099	36122	1614
Olfr837	4022	11713	10541	10334
Olfr843	23850	24940	30466	25989
Olfr845	27981	29710.5	27997.5	31797
Olfr846	24042	25107	38577	36527

Olfr847	3070	7027	15967	17006
Olfr849	10239	14820	15523	14934
Olfr850	11469	12163	20895	21671
Olfr851	17073	19054	22701	22925
Olfr853	24226	25385	27094	26177
Olfr854	32450	22946	11992	31009
Olfr855	28413	26675	31703	31204
Olfr859	29706	33262	39921	39318
Olfr860	12582	12587.5	21130	21884.5
Olfr862	28065	32670	30156	34245
Olfr866	452	11941	14150	12452
Olfr867	6055	31834	41093	36828
Olfr868	37094	40836	1662	1158
Olfr869	26698	38628	1068	38806
Olfr870	5213	8183	10429	12069
Olfr871	2825	8127	8376	10137
Olfr874	40951	39980	4602	73
Olfr875	20524	23089	28425	33711
Olfr876	38501	39971	40796	40854
Olfr877	2547	9157	5642	10817
Olfr878	30171	32193	32088	30632
Olfr881	1786	39831	6277	3046
Olfr883	23081	24042	39118	37304
Olfr887	28253	39702	31450	29941
Olfr888	32003	17496	39326	21145
Olfr889	6003	1108	16015	8050
Olfr890	23128	31310	37310	36283
Olfr891	1265	1483	15428	10441
Olfr893	25152	26393	27944	36337
Olfr894	15627	17485	19218	18611
Olfr895	34456	24224	31810	33453
Olfr898	29219	34663	33939	1261
Olfr899	31040	37164	34363	32504
Olfr90	35246	35925	3844	3579
Olfr900	37748	33876	40564	40650
Olfr902	28968	3474	38311	38968
Olfr904	38960	14882	15869	19254
Olfr905	38740	38995	27638	26707
Olfr906	27913	27458	32910	32881

Olfr907	4358	15484	32347	34022
Olfr908	24307	30397	6671	14123
Olfr91	5774	1564	40313	975
Olfr910	15674	18502	25049	30956
Olfr911-ps1	32283	34175	35495	34089
Olfr912	10126	596	9626	13587
Olfr914	15837.5	18925.5	26277	25742
Olfr915	37561	39038	4710	39604
Olfr916	32915	34409	35673	33832
Olfr918	30541	34456	27662	29353
Olfr919	14930	12141	18220	14105
Olfr92	12370	6049	19707	21925
Olfr920	9389	9281	13108	13611
Olfr921	15356	16697	19207	18122
Olfr922	14424	16204	19195	22750
Olfr923	23899	25017	28173	36628
Olfr924	21129	24077	32234	29314
Olfr926	17192	17232	24459	23250
Olfr93	14421	13073	23218	20571
Olfr930	8082	11715	15474	15858
Olfr933	40684	3895	14019	13592
Olfr934	26372	1080	28877	34053
Olfr935	32323	34653	34653	32887
Olfr937	15939	19389	20160	17590
Olfr938	17170	18176	18934	19442
Olfr94	7205	10825	13472	16626
Olfr943	3149	3325	32625	36967
Olfr944	39703	1885	5355	6910
Olfr945	33236	35627	38171	38858
Olfr948	25419	31695	32695	38797
Olfr95	20169	19121	18740	24114
Olfr954	10032	16392	20012	14205
Olfr955	30833	30135	31059	4963
Olfr957	39914	1966	5277	3729
Olfr958	37817	10303	14958	18986
Olfr959	6213	7119	23544	24850
Olfr96	31984	33609	34955	33156
Olfr960	5187	9354	8596	13857
Olfr961	15567.5	17850	20566	20403

Olfr967	7031	5696	11220	9357
Olfr968	23733	24910	26505	28389
Olfr969	36626	8006	13492	21476
Olfr97	9233	16209	7119	13150
Olfr970	22886	24477	25201	25453
Olfr971	26332	37212	36520	39463
Olfr972	30471	32010	33530	31916
Olfr974	2595	4344	5805	5817
Olfr975	8012	10862	14630	13261
Olfr976	16549	19193	31615	28668
Olfr978	5074	7369	16016	16007
Olfr979	23093	36491	29583	34893
Olfr98	27411	34184	2917	41038
Olfr980	23832	23988	25850	30676
Olfr981	20962	18195	17247	12935
Olfr982	15944	19500	22567	18664
Olfr983	21024	23489	23875	24126
Olfr984	17560	19471	22594	31381
Olfr985	2992	333	8062	7780
Olfr986	6748	15035	14018	20658
Olfr987	8716	12382	3750	5275
Olfr99	37092	34571	34719	32972
Olfr992	18859	16449	30126	25218
Olfr993	30520	32667	32512	30966
Olfr994	32260	655	2758	11429
Olfr995	24022	25260	26604	25620
Olfr996	27957	11815.5	18145	19890.5
Olfr998	30719	32856	33418	2374
Olig1	30451	38034	6756	34174
Olig2	5586.5	7651	14981.5	3775
Olig3	27687	35041	3252	5336
Olr1	33598	8134	6117	14918
Oma1	4184	1653	39029	2236
Omd	26600	27621	30533	29139
Omg	2063	39721	3506	38487
Omp	9393	9797	9141	9011
Omt2b	31238	32909	34593	32965
Onecut1	8864.5	9675.5	32674	14785
Onecut2	17939	20006	21469	25685.75

Onecut3	29784.5	30574	13001	30185
Ooep	28990	22875	36000	35459
Oog1	2696	6442	17040	16660
Oog2	6181	8874	6385	13430
Oog3	1159	32016	32884	31404
Oog4	28320.5	26676	37883	30923
Oosp1	34504	23281	19813.5	4824.5
Opa1	17570.5	15731.5	16212	15574.5
Opa3	24445.5	27058	19499.5	22083.5
Opalin	27158.5	36964	30733	16122.5
Opcml	28604	31660	27877.33333	22252.33333
Ophn1	20429	11975.33333	20417	26868
Oplah	17086	11684	15281	12427
Opn1mw	32490	588	36629	37151
Opn1sw	26195	27723	28930	27754
Opn3	10933	9955	6402.5	7836.5
Opn4	31081	35361	1627	32892
Opn5	20352.5	5252.5	5799	6868
Oprd1	32066	33777	12777	11767
Oprk1	38423	40965	27975	5944
Oprl1	27679	25404.5	30153	28525
Oprm1	26584	27366.25	13214.75	23107.5
Optc	15538	19447	32242	36088
Optn	15075	20214	16898	21188
Orai1	7637	9631	3935	4360
Orai2	972	16383	39228	12765
Orai3	38651	35184	36406	29979
Oraov1	15692.5	18011	18596.5	20878.5
Orc1	21830	20104	27259	27795
Orc2	14978	16376	13680	13711
Orc3	18663.33333	17987.16667	11905.66667	20149.16667
Orc4	18757.25	26233.25	22556.75	26574.25
Orc5	11179.5	29807	11116	11270
Orc6	21823	25549	25645	24399
Orly	18484	23126	15142	23008
Orm1	39534	10986	21614	28612
Orm2	12257	19513	38426	314
Orm3	5670	22902	14863	40442
Ormdl1	29979.5	29959.5	29392	28964.5

Ormdl2	16302	18336	18124	19854
Ormdl3	14653	13183	12354	9732
Os9	17082.66667	25291.66667	23528	13336.66667
Osbp	4485	2662	41085	924
Osbp2	24252.5	17582	29842	28255
Osbpl10	21925.66667	9236.333333	14957.66667	13127.66667
Osbpl11	18177	7975	16312	10601
Osbpl1a	9759.333333	18318.66667	3765.666667	14536
Osbpl2	30630	26110	25671	25455
Osbpl3	3181.5	12354	19317	27583.5
Osbpl5	44	529	5876	6228
Osbpl6	26685.5	24067.5	31241.5	33699
Osbpl7	954	3836	5257	5521
Osbpl8	22509.2	16973	23409.4	18601.8
Osbpl9	20665.33333	21152.33333	22166.33333	23613.66667
Oscar	19987	20822	31309	30316
Oscp1	22114	19536	24863	18681
Oser1	36619	37810	35951	39481
Osgep	11009	13574.5	9513.5	12288.5
Osgepl1	29728	35775	25890	27837
Osgin1	15392	18964	10993.5	19061
Osgin2	7742	11702	6123	9587
Osm	11784.5	11569.5	30290	11728
Osmr	34797.5	33519	20767	7153.5
Osr1	13669	12357	18359	18646.5
Osr2	525	32060	4072	33898
Ost4	30859	34235.5	33850	34641.5
Ostc	12298.66667	15461.66667	15570	13122.33333
Ostf1	17853.5	22144.5	19086	22449
Ostm1	13832	12768.5	14891.5	18254.5
Ostn	30917	36584	33406	35490
Otc	29511.33333	32591	34863.33333	12552
Otoa	32923	32850	36462	37428
Otof	17113	13906	25800.5	25454
Otog	17349	18813	20223	19037
Otop1	38407	3484	6084	4282
Otop2	3339	37101	7175	4805
Otop3	10629	10538	12421	5135
Otor	18445	19507	25406	26902

Otos	7240	10048	10595	10818
Otp	26490	28072	29361	28049
Ott	26898	33958	28120	26985.5
Otub1	3760	23904	23634.5	5247.5
Otub2	14690.66667	5552	36778.33333	981.3333333
Otud1	15874.66667	18543.33333	27421	25480.66667
Otud3	18253	22346	23710	28522
Otud4	11453.33333	24783.33333	24293	15681.66667
Otud5	2631	2556	3436	6298
Otud6a	28043	40124	39386	3375
Otud6b	15562	19453.5	18767	20559.5
Otud7a	26455	27580	30877	29389
Otud7b	15161.8	13019.8	17188	16186.6
Otulin	31225	26574	32310	34218
Otx1	26800	22803.5	25774.5	8975.5
Otx2	32734	31190	34279	31010
Ovca2	4379	3102	424	620
Ovch2	5239	5593	6192	5147
Ovgp1	24585.5	8334	7676.5	11039
Ovol1	36489.5	4356	4953	11736.5
Ovol2	5522.5	22419.5	23574	23161
Oxa1l	10960.5	27599	24383.5	22918
Oxct1	11131.66667	13573.66667	20931.33333	12318
Oxct2a	20593	19546	22101	27129.5
Oxgr1	27280	28596	31836	30206
Oxld1	10828	14531	12316	11985
Oxnad1	17013	15271	17550	14412
Oxr1	15278	27865.66667	2378	3482.666667
Oxsm	29821.66667	29995.33333	24959	16727.66667
Oxsr1	15811.75	21874.75	19364.5	11114
Oxt	814	40747	7950	6537
Oxtr	29589	26421	13856.5	14799
P2rx1	18958	11584	24153	24540
P2rx2	15158	21787	21422	21971
P2rx3	19936.5	21301	22887.5	22234.5
P2rx4	12698	9715.5	7424	7427
P2rx5	27157	24134	25802	25096
P2rx6	10533	10547.5	13124	12465
P2rx7	41081	37427	40893	39020

P2ry1	15409	28122	8501	21563
P2ry10	29341	27036	1355	36860
P2ry12	23016	11951	13073	1903
P2ry13	4220	16400	26572	1662
P2ry14	1352	6631	32173	38512
P2ry2	17362.5	22037.5	19130	3761
P2ry4	26104	27667	29057	8806
P2ry6	14248	38508	10294	35153
P3h1	20021	26437	20041	25866
P3h2	19156	14939	25649	21119
P3h3	40161	38965	25666	25437
P3h4	39166.5	36647	1984.5	2365
P4ha1	8980	14505	14587	19745.5
P4ha2	34308.5	36227	19964	21004.5
P4ha3	26647	28375	1332	36227
P4hb	30521	24792	25473	24984
P4htm	1596	3022	6025.5	23425
Pa2g4	18715.5	16673.5	14561	17207
Pabpc1	35613.5	11425	28303	31386.5
Pabpc2	23630	15711	18347.5	17834
Pabpc4	20067.33333	24926.33333	23698.33333	28483.33333
Pabpc4l	29213.33333	33466	23340.66667	14233.33333
Pabpc5	29374	30868	32314	31162
Pabpc6	2444	8233	34218	2024
Pabpn1	13601	13633.66667	12617	12310.66667
Pacrg	38356	18525	38382	15557
Pacrgl	5834	10907	11254	10995
Pacs1	29013.5	29337	29347.5	28335
Pacs2	26143.75	17520.25	18497	17279
Pacsin1	12815.66667	11014.33333	20050.66667	15111.66667
Pacsin2	11577.66667	13272.33333	7820	10516.66667
Pacsin3	12231.5	20313.5	16428	5832.5
Padi1	17503	19053	22497	22864
Padi2	8587	35960	4345	30040
Padi3	21843	11736	24698	13792
Padi4	12834.5	16877	29878	17871.5
Padi6	20094	22265	31922	28332
Paf1	19997.5	16970	22661	20097
Pafah1b1	9253.5	23166	26148	25678.5

Pafah1b2	6458	10668.5	7965	11171.5
Pafah1b3	18814	17002	22137	21906
Pafah2	11669	12675	11463	13877
Pag1	22145.66667	31092.66667	16871.66667	22816
Pah	27759	29074	31943	30435
Paics	20089.5	3826.5	3912	4927.5
Paip1	28344	17136	16421.33333	16965
Paip2	11056.5	12478.5	12562.5	11703.5
Paip2b	14585	32595	9717.5	29870.5
Pak1	37614	30027	33335	27717
Pak1ip1	15348	13951.33333	12282.33333	15688.66667
Pak2	18239.75	20201.25	24039.75	25164.75
Pak3	7137.5	11243	14474.5	17400.5
Pak4	21025	4520.5	2931	23385.5
Pak6	16564	18087	20330	21406
Pak7	27541.25	23226.5	31728.75	26292.25
Pakap	25502	19324	34233.5	23384
Palb2	11912.5	2018	9190.5	5038.5
Pald1	15640.5	35190	18487.5	20762
Palld	15708.33333	23898.66667	14072.33333	23550.66667
Palm	18126	21101	15326	15634
Palm2	14463	13092	19269	18678.5
Palm3	12326	9125	9045	9537
Palmd	17367.66667	18103.66667	21833.33333	18829
Pam	16098	18586	21111.8	23774.4
Pam16	3975	11309	10016	9779
Pamr1	12874	10724	40171	7705
Pan2	18860.5	18125.5	19756	19439
Pan3	16642.2	25846	20171	26366.2
Pank1	23163	26370.66667	24624	26385.33333
Pank2	12848	15649	12103	14697
Pank3	40343	28088	17327	15239
Pank4	26301	23527	23451	23523
Panx1	6548.5	23507	4446.5	24229
Panx3	26569	28146	29054	27940
Paox	5737	20192.5	2956.5	33337.5
Papd4	5247	4644	3521	3183
Papd5	14265	11312.5	11640	12216
Papd7	10912	14551	10596	16318

Papl	5360	10789	17325	16368
Papln	1616	31262	32997	31303
Papola	16118.66667	5017.333333	16038	3663
Papolb	28922	30307	32880	31298
Papolg	17909.5	31865.5	15373.5	14325.5
Pappa	17171	17141.5	18129.5	20716
Pappa2	6348	6679	2735	9010
Papss1	22036	31803	24112	25795
Papss2	24750	15892.5	24757	35319
Paqr3	22429.33333	20281.33333	13024.33333	15007
Paqr4	2949	41052	2733	40791
Paqr5	39587.5	24545.5	36331.5	34399.5
Paqr6	143	5523	10563	10142
Paqr7	18934	12567	12214	6894
Paqr8	29895.5	28572	14637	12835.5
Paqr9	22494	24280	27406	24129
Pard3	16321	22579.85714	20595.85714	21672.57143
Pard3b	25862.83333	23587.5	16742	21704.66667
Pard6a	29754	26848	30142	33289
Pard6b	29929	25626	26937	29525.33333
Pard6g	22117.33333	27367.66667	29677	32655.33333
Parg	25155	24947	25929.5	25823
Park2	17518.25	9263.25	17271	21082
Park7	12821	15501	17543	17715
Parl	9171.5	25200.5	18738	24752.5
Parm1	20785.5	3551.5	7472	5326.5
Parn	22901.5	23424	21524.5	21216
Parp1	24154.5	23152	20730.5	19430.5
Parp11	4966	4615	2947	2772
Parp12	4643	8443	39628	8992
Parp14	13899	19831.66667	14384	12979
Parp16	3992	9437	7235	10952
Parp2	23006	18931	22939.33333	21446
Parp3	22304	22889.5	31320	34227
Parp4	8147	18110	19820	11920
Parp6	7667	3773	40927	990
Parp8	25495.33333	18967.33333	18086.33333	23811.33333
Parp9	22041	24760	21635	25152.5
Parpbp	13125.5	25574.5	12160.5	8270

Pars2	28859	23729	24320	24380
Parva	28956.5	9371.5	32508.5	22821
Parvb	35193	38150	37063	804
Parvg	12305	21513	11002	19476
Pask	6581	1881	5667	859
Pate2	25973	25989	11207	7507
Pate4	32757	34932	36326	34158
Patl1	12489	11845	11360	12284
Patl2	8608	9740	4459	4064
Patz1	16600.5	15859	14401.5	10602.5
Pawr	31559.5	30650.5	35484	20387.5
Pax1	29515.5	32777.5	30667	32231
Pax2	30245	24908	30289	30348
Pax3	29352.5	35412.5	22339.5	19900.5
Pax4	7605	11239	17826	8124
Pax5	25705	28802.5	29992	31705.5
Pax6	24219.66667	26222.33333	24769	26854.33333
Pax7	13577.5	13074	20091.5	21940
Pax8	1443	33749	18687	21653
Pax9	11525	12005	16495	26477
Paxbp1	16102	29725.5	34102	34119
Paxip1	21168.5	21019	23825	22605.5
Pbdc1	10264.75	20988.25	18686.25	19929
Pbk	41017	41036	1227	3977
Pbld2	15102	11238.33333	8942	20770.66667
Pbp2	8251	3486	1629	3464
Pbsn	38813	29913	40180	37719
Pbx1	20844.25	17686	23290.75	18521
Pbx2	19467	35528.5	39385	29479.5
Pbx3	6328.25	12165.5	14564.25	12272
Pbx4	13341	8146	12439.5	15509
Pbxip1	1951	8157	37988	3194
Pcbd1	536	4288	10219	11977
Pcbd2	2627	5382	6718	3126
Pcbp1	25746.5	26046.5	25641.5	26687.5
Pcbp2	21537.5	14755.5	14099	13263
Pcbp3	25840	12269.66667	24391.66667	24828.33333
Pcbp4	3241	4350	317	309
Pcca	10958	12282	7860	10450

Pccb	33417	26811	26497	25164
Pcdh1	25202	32965	38821	39025
Pcdh10	24044.5	26530	27672.5	25616
Pcdh11x	15951.5	27587.25	25528	26615
Pcdh12	22510	11295.5	21312	22119
Pcdh15	22499	24525	25213	26424.5
Pcdh17	31802.5	5513	14795.5	38747
Pcdh18	35742	29223	2869	5229
Pcdh19	19152.5	23680.5	24330	9563.5
Pcdh20	11340	13959	39594	14206
Pcdh7	16918	12066.33333	14817.66667	10222.33333
Pcdh8	32909.5	28954	21716	8363.5
Pcdh9	23359.55556	29272.22222	27874.55556	27626.44444
Pcdha4	23143.33333	24499.33333	31435.66667	31344.33333
Pcdha6	15336	14278.5	19958	20915
Pcdha9	29805.66667	17443.33333	20133	19300.66667
Pcdhb1	33087	36208	32313	30836
Pcdhb10	1541	1917	5151	9953
Pcdhb11	27167	28466	30963	29479
Pcdhb12	13790.5	30279.5	30981.5	29795.5
Pcdhb13	29333.5	31074	32943.5	21826.5
Pcdhb14	11402	13647	20410	22636
Pcdhb15	21450.5	4024	29670	4788.5
Pcdhb16	11714	29051.5	31265	16141.5
Pcdhb17	20476	28135	12394	12568.5
Pcdhb18	885	5846	13757	13962
Pcdhb19	19895	24153	23474	5163
Pcdhb2	39908	27720	39595	29439
Pcdhb20	8402	5827	10170	9113
Pcdhb21	15876	20014	21554	17402
Pcdhb22	17778	31290	16521	24335
Pcdhb3	36075	6683	15624	14255
Pcdhb4	21546	20595	23573	22558
Pcdhb5	21483	30810	26227	27905
Pcdhb6	12077	10069	10684	14232
Pcdhb7	4584	5965	8713	7769
Pcdhb8	15618	18258	22324	21922
Pcdhb9	39805	6700	5891	5710
Pcdhga1	1500	1895	40573	40390

Pcdhga12	31662	33460	34894	33532
Pcdhga4	34759.5	15580	17890	17390
Pcdhga7	20550.25	12745.75	16365	15430.75
Pcdhga9	33094	7889	16378	19607
Pcdhgb4	37674	36955	30190	2707
Pcdhgc3	23307.66667	16394.66667	29560.33333	31801.33333
Pced1a	16472.75	14681.25	13426	14061
Pced1b	34179	17225	18476	26079
Pcf11	15489.5	15495.75	15032.75	17430.5
Pcgf1	6553	9273	7885	9226
Pcgf2	14933	15864	13449	7448
Pcgf3	15940	17544	13131	18267.33333
Pcgf5	16490	22506	22952.33333	22802.66667
Pcgf6	28027	13829.5	13825.5	17561.5
Pcid2	31871	31072	28903	29296
Pcif1	23682	23544.33333	22762.66667	21678.33333
Pck1	15687	14739	24887	25539
Pck2	18195	11396	14960	7617
Pclo	29059.71429	27556.42857	24659.42857	17229.71429
Pcm1	16685.33333	28542.33333	23598.33333	23578
Pcmt1	24961.33333	9716	7913.333333	11105.66667
Pcmtd1	36863	29039.5	32049.5	29538
Pcmtd2	9018	5645	9687	7056
Pcna	38841	24423	38933	24993
Pcnp	20253.5	17315.5	8882.5	9699
Pcnt	22696	22638.5	25308.5	21569.5
Pcnx	12461.66667	13324	12307.33333	15492.66667
Pcnxl2	35442	17886.5	3810.5	22080.5
Pcnxl3	23293	20831	24113	20119
Pcnxl4	18590.8	12218.2	12997	12298
Pcolce	21476	19929	24649	31785
Pcolce2	27499	10523	40678	4773
Pcp2	25783.5	4791	9237.5	9331.5
Pcp4	24147	26000	26953	29310
Pcp4l1	36680	21251	39677	23033
Pcsk1	17761.5	30878	19654.5	22461
Pcsk1n	23232.33333	25414	28098.66667	28353
Pcsk2	20265	22469.5	6869.5	7088.5
Pcsk4	25279	22710	25632	22767

Pcsk5	29135.66667	23054.5	23106	26912.33333
Pcsk6	3468.5	5383.5	12483.5	9199.5
Pcsk7	25956.5	25089	28069	25831.5
Pcsk9	35230	37446	717	4067
Pctp	14724	13271.5	13548.5	10862
Pcx	5811	18419	10413	22722
Pcyox1	10098	7474	9256	6122
Pcyox1l	478	36687	1997	35693
Pcyt1a	26998	15041.5	17806.5	11370.5
Pcyt1b	22275.33333	18586.66667	26284.33333	15859
Pcyt2	24662.5	19422	16352.5	15766.5
Pdap1	10068	13248	16818	17247
Pdc	4323	12441	2460	3565
Pdcd1	36431	40827	38619	38954
Pdcd10	26829	9790	9367	9183.5
Pdcd11	17249.6	15807.6	17569.2	24391.2
Pdcd1lg2	21342	39840	18501	40758
Pdcd2	31911	32439	31370	34977
Pdcd2l	4063	6006	5084	3484
Pdcd4	16321	15753	16452	8538
Pdcd5	6519	11905	9196	9506
Pdcd6	22551	21788	23110	22457
Pdcd6ip	9617.25	14446.5	11496.5	10778
Pdcd7	15100	17205	16256	14173
Pdcl	12100	15435	17544	18037
Pdcl2	16328	18265	18524	18210
Pdcl3	22362.66667	17252.66667	28038.33333	17892.66667
Pddc1	34928	35360	33754	36110
Pde10a	31874	19851	14449.5	22666.5
Pde11a	24122.5	25402.75	20891	26454.25
Pde12	24335.33333	23715.33333	35926.66667	37883.33333
Pde1a	17596.75	14623.75	19064.25	18351.25
Pde1b	19622	17270	25648	26388
Pde1c	23536.42857	27024	22385.42857	22940.42857
Pde2a	7414.5	8985.5	13437.5	14256
Pde3a	27672.66667	28276.33333	31864.33333	19662
Pde3b	26231.5	7332	26571.5	28339.5
Pde4a	29249.33333	27862.66667	18218.66667	26327.33333
Pde4b	25514.25	23950.5	23530.5	27941.125

Pde4c	22117	20660	23907	23248
Pde4d	25147.42857	23007.42857	29318.57143	27959.71429
Pde4dip	9889	16786.66667	23632.66667	19408.83333
Pde5a	11930.5	18686	18980.5	6331.5
Pde6a	26210	37391	28396	27274
Pde6b	29030	27767	31541	35654
Pde6c	8932	12475	25075	23498
Pde6d	21312.5	24642	18469.5	20411.5
Pde6g	13341	16243	17017	17975
Pde6h	21125	13837	17754	14130.5
Pde7a	15996.33333	25301	25458.33333	24896.66667
Pde7b	25930	24110.66667	23745	25572.83333
Pde8a	2215	732	976	417
Pde8b	22305.5	20667	13251.5	15059.5
Pde9a	20582	18693	24107	22746
Pdgfa	16975	12422	18655.5	12959
Pdgfb	16124.5	19190	18889.5	24887
Pdgfc	24703.66667	13391.33333	26298.66667	16103.66667
Pdgfd	33159	4433.5	15604.5	29519
Pdgfra	11564	15606	14781.5	24076.5
Pdgfrb	17249	30160	21820	1957
Pdgfrl	28020	33393.5	29255	17947.5
Pdha1	20741.5	21054.5	19713.5	20631.5
Pdha2	28429	26737	30772	36805
Pdhb	3464.5	530.5	20646.5	2060
Pdhx	20440	16724	16204.33333	17649
Pdia2	6894	14742	11291	14742
Pdia3	20634	17612	17743	20167
Pdia4	19021	23793	26631	28399
Pdia5	19877	27022	22481	24121
Pdia6	22594.33333	27273.66667	21945	25626.66667
Pdik1l	27597.5	28381.5	24794.5	27772.5
Pdilt	30555	32113	33719	31980
Pdk1	38108	26966	32736	33534
Pdk2	5114	7584	10869	9327
Pdk3	23683	5560	18494	19595.5
Pdk4	37890	5805	10778	11025
Pdlim1	15796.5	12153.5	15998	18449.5
Pdlim2	10430	25010.5	22763	19465

Pdlim3	19517.5	18857	27334	30653
Pdlim4	20454	22509.5	17351	23241
Pdlim5	18257.66667	17085	17103.16667	16330.83333
Pdlim7	27459.25	20372.75	28470.25	21721.25
Pdp1	19993.5	13804.5	20907.5	13054
Pdpk1	18004.25	19295.75	17194.5	13959
Pdpn	5431.5	13953	15311.5	21363
Pdpr	14298	11735.33333	19849.33333	18124.33333
Pdrg1	5836	5482	6603	4763
Pds5a	25427.5	28529	24873	25721
Pds5b	11133.25	18648.5	31271.25	20807.25
Pdss1	28966.5	30599.5	29796	30155
Pdss2	20170	20221.4	29577.4	32455
Pdx1	20353	31098	24623	34929
Pdxdc1	4878.666667	17658.66667	6142	19039.66667
Pdxk	16820.66667	30660.66667	13541.66667	27524.66667
Pdxk-ps	19910	16187	18999	18310
Pdyn	8997	13302	13161	14583
Pdzd11	21364.33333	24101.33333	25169.33333	25825.33333
Pdzd2	21546.4	16392.8	21205.2	20872.8
Pdzd3	24049	25665	24679	35917
Pdzd4	32786	25144.33333	37862.33333	35872.33333
Pdzd7	20047	26155	29343	26779
Pdzd8	11955.5	22594.5	22719.5	20858.5
Pdzd9	33773	39565	730	10482
Pdzk1	23174	25090	28450	31856
Pdzk1ip1	36273	30743	39226	31149
Pdzrn3	26022.5	16167	23215.5	10139
Pdzrn4	23910.66667	20676.33333	22115.66667	24108.33333
Pea15a	12915.5	10139	14228	12386
Peak1	19685	15217.8	18412.6	25796.2
Pear1	18461	15157.66667	8742.666667	10011.66667
Pebp1	22190.5	25625	22030.5	26335.5
Pebp4	32773	39439.5	17854	20502
Pecam1	3125.5	7415.5	17588	14889
Pecr	13595	14131	12662	11969
Pef1	37223	310	37546	921
Peg10	29621	31109	32804	31233
Peg12	7739	9502	9974	11254

Peg3	17634.33333	24523.66667	25192.33333	26561.66667
Peli1	27571	31730.75	14076.25	30670.5
Peli2	22836.33333	15583.66667	17498.33333	15196.66667
Peli3	9501	19047	12272	19613
Pelo	37111.5	16065	32186	37127.5
Pelp1	12484.5	16974	17568.5	19889
Pemt	5693	9111.5	6554.5	7294
Penk	16187	9961	17211.5	18037.5
Peo1	18516.5	20871.5	22265.5	25628
Pepd	23651	22547	22710	8583
Pept1	29113	31235	38968	38167
Per1	10399	5884	13572	12111
Per2	11902.5	13490	17558	21569.5
Per3	40617	37900	38355	40647
Perm1	25216.5	16228.5	23205.5	17748
Perp	14298	11997	7764	6897
Pes1	31702	30773.5	12388	12397.5
Pet100	21841.5	7186	6803	5586
Pet2	38326	4360	652	11978
Pex1	18390.33333	30924	32114.66667	32384.33333
Pex10	25276	35995	23199	25596
Pex11a	23539	21878	20635	17831
Pex11b	37408	39001.5	38170	36752.5
Pex11g	23870	31139.25	24427.25	32523.5
Pex12	30574.5	34064	24515.5	29889.5
Pex13	27546.33333	22861.33333	28530	27230.33333
Pex14	17663	17930.5	17982.5	1785.5
Pex16	13286.5	25804.5	15597	23546
Pex19	12714.5	15624.5	11596	12853
Pex2	26356	12469.5	12058.5	12723
Pex26	36477	36029	36040	34933
Pex3	20743.33333	25073	22247.66667	23701.66667
Pex5	13031.5	14183	13065	13840.5
Pex6	20534	18686	19322	16551
Pex7	34133	37688	37984	35860
Pf4	16925	18779	35672	35712
Pfas	8747.5	7004.5	9866	7409
Pfdn1	9683	8622	3821	3802
Pfdn2	2428	9259	8050	6289

Pfdn4	18602.5	23401	18947.5	17811.5
Pfdn5	4612.333333	6337.666667	6737	6474
Pfdn6	35082	840	2968	4396
Pfkfb1	15823	17313	18076	19152
Pfkfb2	13502.25	23602	15580.5	23651
Pfkfb3	16260.66667	14156	5921.666667	6314
Pfkfb4	10072	8318	8890	8901
Pfkl	22448	35147	18862	25104
Pfkm	4776	2682	1699	40180
Pfkp	15396	11551.66667	14882.66667	15961.33333
Pfn1	2317	5470	6700	7457
Pfn2	24509.5	9899.5	9130.5	18871.5
Pfn3	6369	268	8398	13284
Pfn4	35105	1320	39946	3074
Pfpl	20203	27812	25814	34832
Pga5	36563	3871	4400	3753
Pgam1	2212	40398	3782	7827
Pgam2	11221	11214	4623	15696
Pgam5	7046	12648	5751	11849
Pgap1	10800.66667	23841.33333	21949.66667	20204
Pgap2	13342	20888	11818	20118
Pgap3	1297	40524	3659	2004
Pgbd1	36883	583	39302	866
Pgbd5	28203	32090	24724	24656
Pgc	37095	38266	2604	626
Pgd	26200	36021	24106.5	18198.5
Pgf	978	37552	2478	874
Pggt1b	17463.66667	19026.33333	26674	30379.33333
Pgk1	25740.5	30475	27736	21058
Pgk2	6203	2900	303	8963
Pgls	2986	6268	4428	5678
Pglyrp1	14452	6847	14208	19393
Pglyrp2	21726.5	3087	12468	6581
Pglyrp3	8097	10800	28885	39647
Pgm1	39007	2094	39100	40150
Pgm2	7231	9460	6309	10279
Pgm2l1	10397	19253.33333	22725	20031.66667
Pgm3	23295.5	24792.5	6003.5	7593
Pgm5	21441.66667	23215	32341	27205.66667

Pgp	30950.5	28949.5	21880.5	22730.5
Pgpep1	6822.5	12595.5	7023.5	10616.5
Pgpep1l	36490	29869	29717	28548
Pgr	20730.5	30646.75	26448.5	24556.5
Pgr15l	31196	32829	34585	38721
Pgrmc1	11495	9083	10194	9866
Pgrmc2	8787	9896.5	28943.5	30218.5
Pgs1	33633	26890	24414	29308
Phactr1	20117	18155	12158.25	18489.25
Phactr2	3277	39872	3691	36959
Phactr3	31640.5	12347.5	30367	28047.5
Phactr4	25984.5	26623	13048.5	11754
Phax	9200	10983	8701	9438
Phb	22472	23374	26997.2	26501
Phb2	7556	6479	6410	6567
Phc1	21344.66667	17644.66667	30494	30036
Phc2	22911.33333	19260.66667	23518.33333	25082
Phc3	19295.33333	15328.33333	16111	28521.33333
Phex	36945	32484	33205	36308
Phf1	7953	4283	5414	2610
Phf10	14330	14223	15110	13226
Phf11a	21917	39080	26053	816
Phf11d	20515	40596	24591	3139
Phf12	13937.33333	14104	27393.66667	25203.33333
Phf13	36577	33591	18088	36705.5
Phf14	18010	22782.14286	19485.57143	20316
Phf19	8483	11302	14614	15049
Phf2	1332	40295	39433	34842
Phf20	26681.25	22120.5	31757.5	31393.5
Phf20l1	25557.83333	19461	21041.16667	17625
Phf21a	18107.5	15615.5	18139.75	17727
Phf21b	39353	11658.5	4262	21636
Phf23	6506.5	4261	9251.5	6290
Phf24	15108.66667	21038	21378	24524.33333
Phf3	23923.57143	20055	21312.71429	20002.42857
Phf5a	36014	35754	31336	34874
Phf6	25481.66667	21700	26811.33333	24383.33333
Phf7	26750	21151	22819	22272
Phf8	14390.5	27942.5	13207.5	10672

Phgdh	21610	18540.66667	23320.66667	15298.33333
Phgr1	26033	11987	37639	40532
Phip	23244	21000.375	16091.875	25119.25
Phka1	20822.33333	18152.66667	19897.33333	18128.66667
Phka2	27354	19658.6	24852.2	15985.8
Phkb	37753	35071.25	25357.25	33380
Phkg1	28860	27529	6540	28785
Phkg2	13022	31442	30662	30928
Phlda1	31029	28382	19580	24410
Phlda2	25522.5	23990	34571.5	32282.5
Phlda3	27084	27490	24781	22086
Phldb1	37378	6247	5887	15616
Phldb2	30094.33333	20140.33333	34880.66667	27125.33333
Phlpp1	36077	7432	29531	4078
Phlpp2	28294	28768.5	30272.5	29783.5
Phospho1	5957	7273	6658	9870
Phospho2	32552.5	32980.5	31361.5	32873
Phox2a	2751	415	7444	7875
Phox2b	28545	31883	39884	34731
Phpt1	30284	36901	28988	30422
Phrf1	21110	19616	21899	19856
Phtf1	19364.5	18545.5	16626	18014
Phtf2	11365.5	13276	11056	15102
Phxr1	24189	25324	27097	26184
Phxr2	2314	6774	16303	15344
Phxr4	32088	28818	33339	33104
Phxr5	20714	23253	22083	21871
Phyh	39145	35670	30303	24000
Phyhd1	21414	40729	24567	648
Phyhip	16703	18463	5160	4501
Phyhipl	30320	30399	18655	34977.5
Phykpl	15837	30556	24278.5	29087.5
Pi15	28721	30227	31597	30533
Pi16	38473	1434	40543	39000
Pi4k2a	18327	12209	18755	16442
Pi4k2b	10427	19670.5	13051	21522
Pi4ka	16590.5	13764.5	15493	14018.5
Pi4kb	34025	27727	37540	33275
Pianp	19241	13705	13905	7017

Pias1	16967.5	31597	33364.5	30775
Pias2	23727.25	21114.75	21233.5	23669.25
Pias3	35981	25123.5	23401	21028.5
Pias4	36260	31717	19435	24828
Pibf1	15764.85714	15456.14286	22914.42857	22905.85714
Picalm	15711	12216.66667	17655.33333	15833
Pick1	19952.5	20067.5	9193	8937.5
Pid1	37583	2826	34345	3050
Pidd1	37528	32700.33333	15166.33333	22601.33333
Piezo1	26084	18723	32465	24730
Piezo2	23492.25	27569.25	28398.5	28792.75
Pif1	18499	16643	19167	18427
Pifo	33777	35766	28959	37659
Piga	7649.5	9291	9308.5	11006
Pigb	32257.5	30949.5	27288	31166
Pigc	37655	35672	33401	33780
Pigf	8915	11145	10057	9056
Pigg	16204.5	13435	15457.5	16737.5
Pigh	37458.5	21482.5	36282.5	36396.5
Pigk	12360	18774	10492	12887
Pigl	40447	4361	35039	37876
Pigm	29474.5	30263.5	29149.5	31024
Pign	31357.25	30770.75	18939	27734.75
Pigo	37781	36236	26470	27734
Pigp	25964	28395	30930	23228
Pigq	17201.5	16710	13151	14535
Pigr	28420	29985	1489	34386
Pigs	22131.5	13157.5	11577.5	9612
Pigt	14108	3900	16024.5	20356.5
Pigu	28522.5	31485	30231	31476
Pigv	12957	11394	12827	6860
Pigw	22138	24528.5	18517	26594.5
Pigx	19736	21544	18976	18114
Pigyl	15327.5	19766.75	17489	19642.75
Pigz	11557	20285	7510	13992
Pih1d1	23187	24735.5	24715	24166.5
Pih1d2	23453.5	7768.5	6575	7848
Pih1d3	14012	18508	21115	22219
Pih1h3b	15187	19547	20852	22910

Pik3ap1	8694.666667	20316.33333	10335.66667	8061
Pik3c2a	5465.5	24149.5	14141	17199.5
Pik3c2b	10352	18312	20448	23864
Pik3c2g	9505	17691.25	15524.75	12795
Pik3c3	26041	22005.5	25068	24088
Pik3ca	9736.5	13047	8720	13791.5
Pik3cb	12650.5	8937	23824.5	25084.5
Pik3cd	20824.5	19624	21228.5	17249
Pik3cg	4774	9554	2213	8965
Pik3ip1	21255	26246.5	20370.5	27828
Pik3r1	22912	20635.8	20641.6	19225.8
Pik3r2	34046	27244	28971	24831
Pik3r3	15448.75	15949.75	20056.25	20002.5
Pik3r4	28387	18448	14081	14213
Pik3r5	9177	12174.5	28785	8416
Pik3r6	38564	39969	33171	37372
Pikfyve	22205	17810.66667	21525	19132.66667
Pilra	14284.5	3913	10988	16582
Pilrb1	12442	2391	10176	36274
Pim1	22139.5	21039	22601	19964
Pim2	23758	15818	16026.5	13439.5
Pim3	12161	18261	10246	18587
Pin1	16989	18130.5	22357.5	23381.5
Pin4	40744	3416	354	995
Pink1	424	32034	546	34861
Pinlyp	25777	22050.5	36307	6885.5
Pinx1	17253.5	23840.5	18294.5	22448
Pip	15264	19250	21278.5	17437.5
Pip4k2a	16686.5	29334	29736.5	20536
Pip4k2b	31204	18952	30787.5	21479.5
Pip4k2c	10212.5	9183.5	11001	9139
Pip5k1a	20970	10969	30749	21525
Pip5k1b	14278.33333	11831.33333	9207.333333	12813
Pip5k1c	35844	38135	15563.5	17100.5
Pip5kl1	794	1118	5577	5079
Pipox	9081	1708	5474	11019
Pir	13713.5	23223	16727.5	24532.5
Pira1	17295	19108	15080	16533
Pira6	22519	23589	23842	32708

Pirt	30026	31507.5	36639.5	32236
Pisd	21456.5	15493.5	21152.5	21969
Pisd-ps3	22831	13895	14718.5	14521
Pithd1	27932	26628	23848.5	23357
Pitpna	11397.5	11264	11779.5	12839
Pitpnb	23948.75	22120.5	18767.75	12232
Pitpnc1	13608	22522.4	13239.2	20010
Pitpnm1	19141	14514	22147	18942
Pitpnm2	20178.25	21023.75	25838	25870.75
Pitpnm3	40947	33441	33092	31602
Pitrm1	20416	19637	18388	20423
Pitx1	10425	408	6893	10705
Pitx2	28153	29772	30688	29324.5
Pitx3	1852	4943	4110	5294
Piwil1	2718	37631	40364	2685
Piwil2	20175	32689	18991	35295
Piwil4	30634	32779	32633	31092
Pja1	23775.5	25960	26642.5	27265.5
Pja2	16492.33333	18593.66667	20514.66667	17462
Pkd1	7607	5766	6694	5249
Pkd1l1	30721	32289	33807	32147
Pkd1l2	16114	14854.5	20190.5	21420.5
Pkd1l3	38737	35239	6317	9085
Pkd2	9307.666667	17868.33333	19690	17663.66667
Pkd2l1	26226	16562	30950	3355
Pkd2l2	15654	23170.66667	8546.333333	9914.666667
Pkdcc	2362	7736	394	1357
Pkdrej	18584	16307	24485	24411
Pkhd1	30974	32860.75	24824.25	17517.75
Pkhd1l1	19133	36064	18548.5	38002
Pkia	24980.33333	23524.33333	16676.66667	26779.66667
Pkib	20281.75	22562.25	23206.25	14487.25
Pkig	18618	17091	18391	14874
Pklr	33828	36930	2236	2811
Pkm	16054	15074.5	13623.5	16600.5
Pkmyt1	2643	6886	5877	9039
Pkn1	29430.5	31428.5	27956	24310
Pkn2	17531.6	24736.8	24633	26419
Pkn3	41039	5995	37695	5937

Pknox1	5641	6973	8433	8136
Pknox2	23441.66667	28010	21158.66667	19973.66667
Pkp1	4080	6609	12534	15796
Pkp2	6819	23877	20268	643
Pkp3	20010	18303	18772	21967
Pkp4	8464.5	11005.5	22766.5	9588
Pla1a	9743	8468	28647	27945
Pla2g10	26299	30855	30886	29330
Pla2g12a	7915	16581.5	13714	15174.5
Pla2g12b	21154	23601	24438	26775
Pla2g15	11717	40544	6406	30586
Pla2g16	28242.5	15955.5	8768	12607
Pla2g1b	27277	26722	37730	36144
Pla2g2a	8831	13677	25815	23913
Pla2g2c	260	5863	4332	9991
Pla2g2d	8965	16840	716	8085
Pla2g2e	8847	10422	14474	15099
Pla2g2f	35692	38292	1360	39982
Pla2g3	23027	9757	12584.5	12211.5
Pla2g4a	20130	9266	14300.5	8085.5
Pla2g4b	24158	26935	25066.66667	29614.66667
Pla2g4c	17496	19633	23605.5	23224
Pla2g4e	23712	24831.5	26573.5	27022
Pla2g4f	24113	27961	26859	25903
Pla2g5	33393	38924	33534	901
Pla2g6	33515	26246	25663	25328
Pla2g7	8555	24142	7050	25598
Pla2r1	25201.5	12072.5	29347.5	13691.5
Plaa	17881.5	20496	17491.5	21607.5
Plac1	12073	16602	20667	20962
Plac8	21688	20134	19664	27195
Plac8l1	6655	12244	21254	21753
Plac9a	370	10605	40061	1994
Plag1	241	36055	4554	9214
Plagl1	33069.5	13633.5	10318.5	11321
Plagl2	7594	6877	3798	4628
Plat	19617	20458	24648	27098.33333
Plau	8027.5	3236	2191	3532.5
Plaur	23119	14541	19501.5	16640

Plb1	22124	30472.5	7082	16926.5
Plbd1	10760	31353	34736	14716
Plbd2	23735.5	23709.5	27534	26851
Plcb1	13310.4	30188.8	18613.2	13113.2
Plcb2	23136	7274.666667	18634.66667	15742.66667
Plcb3	33516	35999	40218	39425
Plcb4	14627.66667	20551	19815	19878
Plcd1	5085	10224	9439	13653
Plcd3	39774	1346	1978	4234
Plcd4	21183.5	16297	35695.5	35170.5
Plce1	19140.6	29700.4	24039.4	23628.4
Plcg1	23444	24477.5	28445	30121.5
Plcg2	15843	13784	15661	14677
Plch1	28483	29934	31649	32860
Plch2	1185.5	3567	9826	7126
Plcl1	20312	27827	21599.5	20652.5
Plcl2	15119.5	12671	12355.5	15497.5
Plcxd1	10490	17042	23329	23687
Plcxd2	40439	36021	39221	34663
Plcxd3	31660.66667	31274	32724.33333	31163
Plcz1	26643	28088	29053.5	31029.5
Pld1	13699.75	11597.5	19999	15599.25
Pld2	21837	22422	23106	21694
Pld3	2779	31525	33384	26865
Pld4	18491	7074	7719	34704
Pld5	30190.66667	32293.66667	32296.33333	35326.66667
Pld6	5376	7808	10240	11497
Pldi	5415	7501	12113	16661
Plec	17807	16135.4	12451.4	14015.2
Plek	8970	12648	12088.5	13954
Plek2	39892	34284	3613	39560
Plekha1	15289.66667	16051.66667	34275	16117.66667
Plekha2	15378	12234.66667	16096	15242.33333
Plekha3	14190.5	18818	16778.5	18893
Plekha4	14902.5	21723.5	18026	24203.5
Plekha5	19454.33333	20054.66667	15437.33333	17193
Plekha6	20459.66667	15764.66667	26948.33333	20638.66667
Plekha7	8276	9390.5	11310.5	10493.5
Plekha8	14301	17729.5	7087	16989

Plekhb1	7587	10176	8849	9418
Plekhb2	20696	20170	19915.66667	19511.33333
Plekhd1	24721	26132	27031	25993
Plekhf1	36141	24490	40966	40777
Plekhf2	24865	20967	22410	19890
Plekhg1	16563.33333	20837.66667	17402.33333	13588.33333
Plekhg2	10687	7480	12633	10976
Plekhg3	16688.5	28031	12584	20642
Plekhg4	39379	9993	8341	19137
Plekhg5	11123	9414	11454	8529
Plekhg6	29422	29849	37882	39169
Plekhh1	21581.33333	27517.33333	24458	32741
Plekhh2	26839	35873	27856.5	26657
Plekhh3	14487	22612	19122	24965
Plekhj1	3006	6199	3058	2480
Plekhm1	4602	36890	1081	37965
Plekhm2	29751.66667	26584.66667	26542.66667	25058
Plekhm3	16282	18852.5	14906	18815
Plekhn1	23071.5	39434.5	3016.5	4634.5
Plekho1	3542	39468	6255	2251
Plekho2	11242.5	11930	14883	13275.5
Plekhs1	16086.5	13763.5	27428.5	27729.5
Plet1	19903.5	26289	40983	7218.5
Plg	3594	6172	20231	23769.5
Plgrkt	8201	6473	6471	4298
Plin1	20666	21037	19238	19590
Plin2	9261	34820	12137	1750
Plin3	26509	22509	35466	35968
PLIN4	6292	6484	14273	13740
Plin5	16801	21826	37966	35524
Plk1	19807	16636	19876	20669
Plk2	5895	9272	11896	16341
Plk3	37760	7159	38925	9945
Plk4	17307.75	16809.75	16750.25	26552.75
Plk5	21033	19788	2928	37696
Pllp	24161.66667	23905.33333	16677	25300.66667
Pln	19591.5	17468	30204	24523
Plod1	11323	5527	5878	179
Plod2	29118	28471.5	33624	33559.5

Plod3	10074	8305	7607.5	6669
Plp1	23354.66667	19919	24364.33333	14311.66667
Plp2	9240	21330	4408.5	19643
Plrg1	19297	18755.5	17740.5	20539
Pls1	26139	30265	30639	29098
Pls3	31465	40035	33253	40321
Plscr1	20985	28675	20068.33333	24560
Plscr2	16136	19090.5	19721.5	18466
Plscr3	21989	19162	12596	31150.5
Plscr4	18113.5	21160.5	10615.5	20780
Pltp	35248	18803	25599	19282
Plvap	16021	19361	21371	21556
Plxdc1	23703	15361	19129	3270
Plxdc2	22662.66667	21009.66667	25004.66667	22313
Plxna1	27847.5	28366.5	6642.5	7965.5
Plxna2	25739	21149	21448.75	20296.25
Plxna3	15617.5	10708	13718	11868
Plxna4	26927	19456.66667	32594	25481
Plxnb1	18360	17934.5	25301	13866
Plxnb2	11015	6840	6602.5	5348
Plxnb3	12798	368	10915	37536
Plxnc1	5142.5	13051.5	25004	13627.5
Plxnd1	35130	3236	26343	37793
Pm20d1	32304	27049.5	29057	32299.5
Pmaip1	17324	22963	22937	25004
Pmch	26542	28312	28659	32657
Pmel	30100	30993	29073	32329
Pmepa1	14537	9585.5	19624	23886
Pmf1	252	940	3382	4777
Pmfbp1	16179	17222	19621	19786
Pml	20087	21660	16996	20113
Pmm1	16031	23479	23799	24909.5
Pmm2	22339	4841	23118	6113.5
Pmp2	29497	36474	31277	29996
Pmp22	31209.5	25244	34049	27123.5
Pmpca	13491.5	13292	11059.5	12365
Pmpcb	40934	2511	506	3663
Pms1	32898	33214.33333	22683	23306.66667
Pmvk	32037.5	39436	34542.5	34705.5

Pnck	8926	8066	18092	17031
Pnet-ps	19996	18459	14641	14471
Pnisr	16414.5	9812	12652.5	12106
Pnkd	9638.666667	13272.33333	13952.66667	14263
Pnkp	24471.5	21197.5	20663	20478
Pnlip	19949	28273.5	27082.5	27112
Pnliprp1	32480	39717	37967	34297
Pnliprp2	27797	36692	29571	30927
Pnma1	13442	23470	6504	10179
Pnma2	38187.5	17571.5	21686.5	10484.5
Pnma3	41087	40272	10192	2665
Pnmal1	886	507	3526	40611
Pnmal2	24047	35111	29306	31477
Pnmt	36397	38369	908	1422
Pnn	26604	26068	31387	32280
Pno1	12072	17453	14338	18034
Pnoc	11211	18919	26697.5	21888.5
Pnp	35162	6558.333333	22492.33333	6229.333333
Pnpla2	13357	7234.5	12213	7813
Pnpla3	1430	35270	11683	7327
Pnpla5	2105	2751	5000	4826
Pnpla6	19858.66667	15807	27459.33333	25549.66667
Pnpla7	6453	1900	6300	2441
Pnpla8	18637.66667	23517.33333	23440	26581.66667
Pnpo	20766	17802	16909	15039
Pnpt1	19256	19930	18792.66667	18244.33333
Pnrc1	31585	32400	23301	31627
Pnrc2	4143	477	1168	39190
Poc1a	33488.33333	21879	30204.33333	19595
Poc1b	9587	24385.5	16750	14340.5
Poc5	11267	9264	16769.5	15959
Podn	236	1752	34077	7065
Podnl1	15062	13213	31766	35893
Podxl	25117	26273.66667	18714.66667	26659.33333
Podxl2	34148.5	35637.5	36295	21254
Pof1b	25815	2246	30577	29172
Pofut1	16711.5	14209	14056	14703.5
Pofut2	16630.5	22646	16883	19444
Pogk	35729.66667	27364.66667	25297.33333	26288

Poglut1	23044.5	19610.5	18578	17875
Pogz	15492.66667	15164.33333	18160	21206.66667
Pola1	32534.5	31141	34102	28441.5
Pola2	23129.33333	26759.66667	20668.33333	29407
Polb	7277.666667	19355.33333	21823.66667	20981.66667
Pold1	14336.5	11285.5	11940	9129.5
Pold2	32524	28589	25789	27108
Pold3	20202	20844.33333	24240.66667	22201
Pold4	16961	15456	23159	19333
Poldip2	10351.5	10875.5	27457.5	29191.5
Poldip3	22908.5	20398	22098.5	23447
Pole	23222	24529.5	24884.5	22806
Pole2	25011	23576	23612	21880
Pole3	33975.5	14399	14647.5	13568
Pole4	7954.333333	14294.66667	12483	13274.33333
Polg	9754	9543	8381	9652
Polg2	35254	35950	26922	27584
Polh	26987.33333	15139.66667	14337.66667	26874.66667
Poli	40966	38254	35762	34370
Polk	12314.5	25487	9511.5	10594
Poll	24166	21613.5	24632	23721
Polm	24773	22691	25773	24041
Poln	10146	12291	20650	10431
Polq	22747.5	19528	21912	21248.5
Polr1a	24699.5	17537	18704.5	18483.5
POLR1B	31556.5	31833	32552	37043
Polr1c	6918	11628	11124	12026
Polr1d	25467.33333	26159.66667	30117.66667	30437.33333
Polr1e	22313	35465	25292	35213
Polr2a	30657.5	21956.5	25872.5	22802.5
Polr2b	31307.5	29548	27007.5	27747
Polr2c	40946	4205	5165	7634
Polr2d	32413	37967	39197	34275
Polr2e	17069.33333	20435.33333	19606.33333	20902
Polr2f	33517	34990	35262	33010
Polr2g	27388	33910	26898	28443
Polr2h	4375	12544	9756	11373
Polr2i	287	4718	1731	720
Polr2j	31781	35596	31258	28058

Polr2k	38984	40618	36989	38063
Polr2l	6677	9169	9479	9181
Polr2m	13764	13078	15247	15228
Polr3a	17241.75	25778.25	14188.5	18594
Polr3b	12294	11083.5	12746	13939
Polr3c	32090.5	31930	30550.5	30221.5
Polr3d	12906	15952	14886	16229
Polr3e	10013.5	8942	7309	6440.5
Polr3f	19630.75	22331.5	20993.5	25200.25
Polr3g	20718.5	18968	15802	14487.5
Polr3gl	36115	31088	797	29644
Polr3h	18002	19241	17708	22331
Polr3k	12938.33333	15517	25527.33333	26076.33333
Polrmt	12799	12505	11989	12843
Pom121	8166.666667	5644	16088.33333	14175.33333
Pom121l2	38179	32692	41070	37795
Pomc	11467	17105	21494	22196
Pomgnt1	13577	14194	11589	11549
Pomgnt2	36852	34160	283	37391
Pomk	9744	8369	17365	14584
Pomp	35724	38236	25077	36724
Pomt1	19315	29888	27707.5	26397
Pomt2	16425.66667	16644.33333	17529.66667	15720.33333
Pon1	39369	38322	27198	29460
Pon2	17331.25	24158	25204.5	21935.25
Pon3	19639.5	21132	35120.5	18449
Pop1	25821	15403	17857.33333	16485
Pop4	32179.5	18214.5	34319	36992.5
Pop5	11615	18790	15513	20120.5
Pop7	25739	35003	29841	34557
Popdc2	24800	23893	37899	34431
Popdc3	23290	24362	38669	38941
Por	30889	12216	24849	17984
Porcn	10702	7990	8834	6188
Postn	31748.5	24444.5	22203	38283
Pot1a	21852.5	29838	16347	15775
Pot1b	16838.66667	22672.33333	13596.33333	19780.66667
Poteg	36499	32784	37759	217
Pou1f1	5452	8544	3932	10019

Pou2af1	36779	39107	2282	1560
Pou2f1	12605.5	27429.25	13532.25	21485.75
Pou2f2	11104	8121	7635	2856
Pou2f3	16006	15205	22776	19275
Pou3f1	20721	14390	18467	13189
Pou3f2	9065	10745	39850	14430
Pou3f3	16670.5	28988	30134	12932
Pou3f4	18977.5	1768	22314	3787.5
Pou4f1	23639	23368	25529	24602
Pou4f2	19929	36768	38082	1519.5
Pou4f3	7992	27284	31367.5	32380
Pou5f1	7208	6170	23561	10597
Pou5f2	2296	38901	5141	4608
Pou6f1	13870.5	11744	18230	16606
Pou6f2	34626	40716	3255	2062
Pp2d1	12252	18317	19950	20426
Ppa1	16760	20510	19859	23999
Ppa2	16567	17863.5	13131	13553.5
Ppan	24278	23342	37101	37814
Ppap2a	21296	12810.33333	26179	13271.33333
Ppap2b	17956	20062	14108	17197
Ppap2c	17793	12788	15887	14894
Ppapdc1a	14733	16815	19784	17084
Ppapdc1b	35865	3764	30042	41157
Ppapdc2	27568.5	29008	8290	8037
Ppapdc3	20803	18296	16077	13680
Ppara	41032	4811	40781	38624
Ppard	22855	22513	36411	38160
Pparg	16803	1612	14495	39837
Ppargc1a	15920	10142	13401.5	26867
Ppargc1b	24146	19845.75	19273.75	22080
Ppat	29659	32524	32077	34168
Ppbp	5995	15924	9002	23443
Ppcdc	15385	27313	26554.5	25545
Ppcs	5406	8955	7234	8074
Ppdpf	2931	1445	7076	3470
Ppef1	18124	33943	25324	22674
Ppef2	17971	18491	22328	20435
Ppfia1	22559	19842.66667	18644.33333	21054.66667

Ppfia2	14713.5	18190	11430.5	15230.5
Ppfia3	793	3678	3368	9076
Ppfia4	8027	7521	5550	40431
Ppfibp1	18982.33333	15802.33333	15857.33333	16569
Ppfibp2	20252	11366	20664.5	16861.5
Pphln1	4066.5	5835	4406.5	5842
Ppia	14767.5	15297	5975.5	10816.5
Ppib	19950.5	22305.5	22122	23026.5
Ppic	19898	27329	28662	39308
Ppid	14317.5	20145	13809	18679
Ppie	34780	16954.5	34639	21586
Ppif	22735	23272	16981	20406
Ppifos	35629	40715	11358	1926
Ppig	35257.66667	32405.33333	30607	31961.66667
Ppih	11394	13791	12308.5	11314.5
Ppil1	19869	18917	16189	16362
Ppil2	28973.5	30161	30156	11517
Ppil3	8144	15121	10237	11246
Ppil4	10296.5	10734	12081	12443.5
Ppil6	38216	32338	33877	2298
Ppip5k1	4501.5	4205	23848	26154.5
Ppip5k2	20723.75	14749	19832	15026.25
Ppl	17978	19258	36847	2958.5
Ppm1a	18601.33333	16971.33333	18067.33333	19360
Ppm1b	13776.33333	16637.33333	13636	17269.66667
Ppm1d	24952	26541	26920.66667	26902.66667
Ppm1e	17585.66667	25373.33333	20919.33333	27634.66667
Ppm1f	29734	35205.5	31699	36805
Ppm1g	35744	35577	27764	28158
Ppm1h	30533.66667	31260.66667	27183.33333	27964.66667
Ppm1j	5037	4318	863	2537
Ppm1k	36574	27997	27249	27597.5
Ppm1l	10281.33333	22771.33333	13374.66667	23955.66667
Ppm1m	33787	28432	23367	24316
Ppm1n	37078	18610	760	8896
Ppme1	16226	14084.66667	12491.33333	14731.33333
Ppnr	8975	37434	5749	2137
Ppox	9176	7949	9656	9987
Ppp1ca	35612	27176	32106	32509

Ppp1cb	10264	13487	13390	15702
Ppp1cc	13398	11534.5	15211	12345.5
Ppp1r10	20583.33333	19184	20036.33333	19886.66667
Ppp1r11	9309	20456	21311	25505
Ppp1r12a	35369.5	30287.5	31962	33809.5
Ppp1r12b	15223	13048.33333	27260	18653
Ppp1r12c	5332.5	3540.5	7121.5	24779.5
Ppp1r13b	19241.66667	32324.66667	19925.33333	27471.66667
Ppp1r13l	17121	15773	19452.5	17962.5
Ppp1r14a	34108	31692	33767	14718
Ppp1r14b	20652.5	26086.5	27366.5	27701
Ppp1r14c	25017	24311.8	22497	23867.4
Ppp1r14d	5437	5727	14314	5077
Ppp1r15a	12783	18432.5	15322.5	19089
Ppp1r15b	28275	22706	25303	24389.5
Ppp1r16a	4738	6428	780	40738
Ppp1r16b	21024	19239	9039.666667	10382.66667
Ppp1r17	7998.5	7350.5	24327	15066
Ppp1r18	12015	4371	12283	7500
Ppp1r1a	14413	11677	18775	20618.5
Ppp1r1b	37514	36548	37978	37281
Ppp1r1c	17368	21704.5	19553.5	19410
Ppp1r2	12043.83333	11581.83333	10497.66667	11093.5
Ppp1r21	128	2132	990	39825
Ppp1r27	29271	24184	29679	30915
Ppp1r2-ps7	33013	33111	41008	5578
Ppp1r2-ps9	39637	31124	32895	31133
Ppp1r32	1153	2873	8249	5564
Ppp1r35	8337	14779	13651	13982
Ppp1r36	36274	541	4914	11023
Ppp1r37	9933	10029	11756.5	10269.5
Ppp1r3a	28991.33333	19969	24739	25275
Ppp1r3b	21702	18158	20960	18404
Ppp1r3c	27560.5	28811	31327	29713.5
Ppp1r3d	40327	40119	39199	39040
Ppp1r3e	20207.5	20915.5	25480	6167
Ppp1r3f	1553	40837	2510	40711
Ppp1r3g	23165	24248	26130	25044
Ppp1r42	34975	32436.5	17731	33147

Ppp1r7	27617.5	27505	26080.5	27829.5
Ppp1r8	13901	17546	18045.5	15950.5
Ppp1r9a	19068.83333	15091.83333	19277.33333	18382.66667
Ppp1r9b	14909	12723	15982	9248
Ppp2ca	11220	12831	12790.5	14002.5
Ppp2cb	33501	22773.5	19413	22325.5
Ppp2r1a	34460	29313	25651	30091
Ppp2r1b	16351.66667	15451	14396.33333	17927.66667
Ppp2r2a	24743	23140	21522.5	23965.5
Ppp2r2b	16374.25	28376.75	15783.25	24642
Ppp2r2c	12551	18637	19681	23890
Ppp2r2d	2588	4440	2077	2412
Ppp2r3a	11931.2	18344.8	28702.8	28429.4
Ppp2r3c	24329.5	28373	26069	28342
Ppp2r4	21819	19824	22327	17722
Ppp2r5a	20190	12830.75	20366.25	23346.25
Ppp2r5b	11196	14998	15763	20066
Ppp2r5c	17445.75	27466	15066.25	24819
Ppp2r5d	19217	20152	20130	21674
Ppp2r5e	15372.4	18412.8	11839.4	12821.4
Ppp3ca	15687	7733.333333	12783	11531.33333
Ppp3cb	19937.25	25258.25	24136	22923.75
Ppp3cc	30654	18089.25	22441.25	20363
Ppp3r1	18348.5	22198.5	27606.5	35204.5
Ppp3r2	22625.33333	24197	23566	26303.33333
Ppp4c	33863.5	18722	22673.5	20238.5
Ppp4r1	36772	29431	29881	28807
Ppp4r1l-ps	21389.2	17372.6	27067.4	20790
Ppp4r2	11638.5	13001	10462.5	10574.5
Ppp4r4	11227	8230.5	11347	13766.5
Ppp5c	19331	20414	38305.5	39151.5
Ppp6c	35308	34455.33333	31707	34165.66667
Ppp6r1	18710	14323.33333	16757.33333	29753.33333
Ppp6r2	25272.33333	25635	30784.66667	16726.66667
Ppp6r3	27446.8	16893.6	16960.2	19103.8
Pprc1	16880	15128	14109	15024
Ppt1	21575.33333	18948.66667	25241.33333	22934
Ppt2	18823	16752	14182	15560
Pptc7	2970.5	20295.5	18108.5	21098.5

Ppwd1	23387	32295	28397	26808
Ppy	1796	1221	3237	5706
Pqbp1	7791	7514	8670	7423
Pqlc1	25631.5	16867	32844.5	18768
Pqlc2	20057	16604	14967	15721
Pqlc3	6174	3963	40453	38336
Pradc1	5941	7421	6559	3507
Praf2	31199.5	39719	28881	39382.5
Pram1	16391	13070	21946	38426
Prame	7711	3770	35682	94
Pramef12	31057	32686	34293	32600
Pramef8	39493	39783	39646	39382
Pramel1	12684	12098	19831	18284
Pramel4	25283	26392	28824	27585
Pramel5	4307	38645	125	2866
Pramel6	25682	26786	29356	28107
Pramel7	31969	33243	36524	34494
Prap1	22385	30725	38987	39294
Prb1	666	31023	38701	36307
Prc1	20846	22535	28560	31296
Prcc	19645	21053	25359	23735
Prcp	25948.5	18254	19763	18173
Prdm1	705	1428	39156	4968
Prdm11	35111	35269	39612	33492
Prdm15	17638.5	18995	40369.5	20921.5
Prdm16	16630	21206	14532	18960
Prdm2	25632.5	20968.5	20941	23329.5
Prdm4	15991.5	16703	13004.5	15428
Prdm5	27856	29142	31172	1084
Prdm6	29004.5	32735	15763	21081.5
Prdm8	21085.33333	20422	8357	20811.66667
Prdm9	33106	35814	36761.5	34986
Prdx1	1228	18260	5447	26269
Prdx2	32909	36330.33333	28213.33333	33047.33333
Prdx3	32169	33309	26215	30938
Prdx4	2795.5	4865.5	9259	8487.5
Prdx5	11500.5	32116	14686	20607
Prdx6	10312.25	24587.25	7737.25	22666.75
Prdx6b	37571	24896.5	22911	29618

Prdx6-ps2	20685	4192	25028	15826
Preb	17764.5	13854	14771	15176
Prelid1	17421	17775	16723	19082
Prelid2	34937	30622	20813	24990
Prelp	17614	14684	25578	27657
Prep	20607	19575	18682	20641
Prepl	12736.66667	8883.666667	11939	10812
Prex1	24809	32628	21718	27134
Prex2	19378.5	22452	25711	29010
Prf1	1718	38008	24960	8712
Prg2	39464	27257	40371	5149
Prg3	2473	6279	1622	4512
Prg4	7317	6243	18972	23196
Prh1	16761	23253	21853	27771.5
Prickle1	37615.5	17829.5	22181.5	5344
Prickle2	16407.66667	20434.33333	18507.33333	19274
Prickle3	16102	26572	29192	25193
Prim1	2424	3329	1344	41003
Prim2	11905.5	5982	11292.5	10490
Prima1	13582	12029.5	1628.5	32821.5
Primary Sequence Name	41092	41124	41134	41164
Primpol	20928	15759	17223	14442
Prkaa1	31277.5	12416	31520	12318
Prkaa2	3038	8077	10397	15174
Prkab1	16972	17240	16469	16240
Prkab2	35451	22271	33021	28843
Prkaca	1237	39664	2247	40955
Prkacb	22136.5	14641	32326	31261
Prkag1	30901.5	25198	25151.5	28920.5
Prkag2	30093	28124	27785	24050
Prkag3	5983	8875	12352	41033
Prkar1a	20195	19964	21664	20968
Prkar1b	9753	21289.33333	24075.66667	20975.66667
Prkar2a	27841.75	26097	24786.5	23932.5
Prkar2b	32870	6642	37929	13422
Prkca	14043	13000	18716	18372.5
Prkcb	14062.5	15010	13844.5	14895.5
Prkcd	14394.5	14408	11755	14865
Prkcdbp	23185.5	20420	22390	26046.5

Prkce	26835	24331.6	20526.4	30246.2
Prkcg	40623	2913	11821	12549
Prkch	15208	6398.5	10596	6144
Prkci	13079	12090	9952	11169
Prkcq	13345	24454.33333	22142.66667	5371
Prkcsh	8382	7740	11139	10826
Prkcz	23601	24189	23393.66667	23581
Prkd1	27994	34819	21510	32322.5
Prkd2	1137	39674	3586	872
Prkd3	16258.2	9796.8	21477	18322
Prkdc	24243	21337	21559	21809
Prkg1	22629.14286	24098.14286	24062.57143	24759.85714
Prkg2	19843.5	20132.5	11706.5	7164.5
Prkra	23832.5	21784.5	23993	21371
Prkrip1	27998.5	38335	35419.5	18903
Prkrir	22863.33333	8826	20422.66667	17645
Prkx	7827	1551	4940	40861
Prl	29369	33653	38076	38854
Prl2a1	32401	8325	2288	7165
Prl2b1	17371	23120	8834	8156.5
Prl2c3	17401	25048.33333	26654	31442.33333
Prl2c5	8841.5	19424.5	23088	28712
Prl3b1	10123	14685	19947	20301
Prl3c1	27544.5	31247	16476.5	33619.5
Prl3d1	15811	17333	22755	22108
Prl3d2	35002	32450	39375	35294
Prl4a1	38806	39456	2666	862
Prl5a1	23984	25049	38079	26439
Prl6a1	38585	36086	26286	25187
Prl7a1	1392	4054	12517	9170
Prl7a2	36183	27614	29459	28151
Prl7b1	31710	33433	34840	33072
Prl7c1	29430	28481	31779	30133
Prl7d1	14977	29865	16030	17277.5
Prl8a1	22552.5	21873.5	27220.5	29897.5
Prl8a2	33518	31062	10392	1294
Prl8a6	25951	27150	29248	28027
Prl8a8	12729	15720	10969	12702
Prl8a9	24787	26111	38945	26132

Prlhr	14803	17808	24130	25120
Prlr	30769.33333	30132.33333	25987.33333	28444
Prm1	19070	22851	27948.66667	26001.33333
Prm2	36406	40820	38637	36800
Prm3	20761	20866	26249	26138
Prmt1	19454	8615	21837.5	5303.5
Prmt10	24524	25156	24744.5	28152.5
Prmt2	13048	10762	14209	14285
Prmt3	24419	17738.5	25411.75	19245.5
Prmt5	16098	14688	12875	14410
Prmt6	17726.6	24741	27251.4	27024
Prmt7	33958.5	28468	12785.5	32307
Prmt8	31252	35315	34706	32938
Prnd	34695	25367	36822	9452
Prnp	28195	21066	26052	21208
Proc	2793	4490	10648	10298
Procr	25004	13709	36057	23667
Prodh	26388.5	22889	29948	34249
Prodh2	9180	10496.5	13396	13500.5
Prok1	15242.5	18980	20567.5	20885
Prok2	9070.5	21301	10179	20641
Prokr1	31670	33768	29547	35702
Prokr2	32390	34069	35033	39845
Prol1	27106	35409	38675	2181
Prom1	38494	37747.5	32331.5	31013
Prom2	30625.5	32355	18094.5	35551
Prop1	7496	9494	14485	14182
Prorsd1	13565.66667	17181.33333	14925.33333	10892.33333
Pros1	28007	13857	25247.5	13211.5
Prosc	15056.66667	21179.66667	10786.33333	19352.33333
Proser1	23332	16198.66667	16582	19900.33333
Proser2	23340.5	38710	31891	38264.5
Proser3	37568	26139	29594	24234
Prox1	13161.33333	14856.66667	17802	15417
Prox2	10870	8763	13987.5	12881
Proz	9599	23562	26531	29359.33333
Prp15	24011	25130	3014	37863
Prpf18	19134.66667	24279.33333	21209.33333	24624.66667
Prpf19	8323	5634	3512	5231

Prpf3	20313	20591	20990	21332
Prpf31	25232	8399	7071.5	10338.5
Prpf38a	27478	30235.5	25564.5	28503
Prpf38b	21711	39882	14729.5	13790
Prpf39	15797.8	20013.6	21600.6	14629
Prpf4	20294	21725	21407	22404
Prpf40a	16964.5	15644	12735	11742.5
Prpf40b	27088	30308	33183	36111
Prpf4b	6164.75	12237.75	10917.5	9506.25
Prpf6	15514.33333	13760.66667	17229.33333	19261
Prpf8	31405	23399	23746.5	22026.5
Prph	6117	6183	33746	40529
Prph2	36235	32759	14555	7725
Prpmp5	32054.5	34790	34938.5	36248.5
Prps1	20295.25	20206.75	19590.25	21879.25
Prps1l1	2395	2223	17075	8314
Prps2	27531.5	30750	23740.5	28126.5
Prpsap1	28204.66667	24623	26724	27217.66667
Prpsap2	13121	12854	11702	9348
Prr11	19454	16621.66667	23985.66667	22607
Prr12	17231.5	18463	19161.5	15970.5
Prr13	16716	18736	14531	22099
Prr14	24679	23419	24776	22157
Prr14l	10730	22011.5	30629	27580.5
Prr15	19114	20790.5	21439.5	23085.5
Prr15l	19829	23086	3306	23280.5
Prr16	22051	27681.33333	29144	29355.66667
Prr18	8612	34687	3358	36550
Prr22	18018	19971	23914	25347
Prr23a3	787	4607	6615	7628
Prr27	25626	25366	40726	27931
Prr29	5696	40128	10486	9236
Prr3	21036.5	24704.5	21914.5	22629.5
Prr30	23822.5	4672	11111.5	8760
Prr32	14604	19335	8755	17347
Prr36	12160	10326	13444	16180
Prr5l	21992	36156	14348	30330
Prr9	24541	25855	27040	26087
Prrc1	23070	22476.33333	22132.66667	23607.33333

Prrc2a	5664	5671	2710	40693
Prrc2b	4360.5	22963	4477.5	23284
Prrc2c	21832.25	17317.75	16715.75	16167.25
Prrg1	25360.75	26123.25	27416.75	27296.75
Prrg2	27104	26507.5	27933.5	24968.5
Prrg3	39724	9355	2042	10714
Prrg4	24168.5	21892.5	29073.5	26933.5
Prrt1	16265	18970	22756	25805
Prrt2	19023	18023	27164	24361
Prrt3	21933	23242.5	27267.5	29270
Prrx1	18814.85714	20545.57143	23857	25863.71429
Prrx2	32116	37145	34349	39538
Prrxl1	8848	9565	5766	2862
Prss12	2448	5444	12978	12668
Prss16	25766	30151.5	11548	12302.5
Prss2	21756	24721	27361	32776
Prss21	34322	33473	12109	32576
Prss22	21086	24707	25231	33461
Prss23	11134.5	11485.5	13821	21562
Prss27	18163	14222	19216	14455
Prss28	24392	35009	28140	39362
Prss29	16386	18166	17893	24432
Prss3	20411	24665	8344	8959
Prss30	8644	11229	12954	12104
Prss32	3905	1284	8493	7288
Prss33	13115	14536	22053	21530
Prss34	33436	36613	6672	5285
Prss35	34964	34851	33044	1378
Prss36	20983	21842	23039	22565
Prss37	16477	19385	20170	19933
Prss39	29451	30956	8754	6045
Prss40	15057.5	29722.5	17550	22534.5
Prss41	39349	40392	8787	6594
Prss42	6122.5	24638.5	12897	11776.5
Prss43	24024	37230	40382	39549
Prss44	30090	38927	1647	32127
Prss45	15239	17439	24407	22475
Prss46	11410	2896	12826	6634
Prss47	4082	1395	31418	29993

Prss50	28188	26974.5	30992.5	33561.5
Prss51	8838	12634	9778	10045
Prss52	34015	33521	14896	3673
Prss53	34841	35507	2918	40371
Prss54	632	2262	4369	5004
Prss55	6586	7450	17085	16406
Prss56	8060.5	13364	11450	9637.5
Prss57	24769	25909	6847	10405
Prss58	12079	20439	21239	21218
Prss8	24910	26288	35437	35173
Prtg	31046.33333	20812	11297.33333	22266
Prtn3	218	40801	2708	3888
Prune	38980	32345	37565	33424
Prune2	27823	17607.25	31438.5	27354.25
Prx	28348	26516	26663	27743
Psap	16608.5	2315.5	14665	11596
Psapl1	20805.5	22571.5	25569	5311
Psat1	13368	4147	10381	40499
Psca	37432	40222	2829	4033
Psd	12488.5	27401.5	16159	15494
Psd2	12800	30007	14396	4888
Psd3	25186	23109.8	30016.4	20317.2
Psd4	36917	36999	35162	34846
Psen1	39436	38942	37809	38680
Psen2	27663	28135	27154	34769
Psenen	8288	8847	13830	10189
Psg16	38504	32219	12511	6515
Psg17	11460	10026	20771	21287
Psg18	28396	35647	13409	4367
Psg19	10456	14757	22330	22582
Psg22	27039	28607	29744	28374
Psg23	27748	29095	31425	29919
Psip1	8825.666667	23573.33333	6828.333333	9007.333333
Pskh1	33131	32098	36874	32781
Psma1	17128	19629	15556	19605
Psma2	29552	35275	31867.5	35637
Psma3	6100	12343	3888	7454
Psma4	37421.5	20000.5	26801	37716.5
Psma5	22087	38375	31173	39965

Psma6	12210	22312	15835	19383
Psma7	21475	32330	25073	34045
Psma8	34390	38395	34286	35115
Psmb1	15260	23722	21556	22987
Psmb10	16885	31715	17233	27563
Psmb11	22261.5	22970	29505.5	27963.5
Psmb2	19581	29047	25216	33565
Psmb3	12674.5	16486.5	13566	18732
Psmb4	22237	23458	23378	28086
Psmb5	11054	19011	18283	26292
Psmb6	34879	3732	973	6626
Psmb7	36046	573	38831	2736
Psmb8	10114.5	21880	14184	24037
Psmb9	5748	13578	8534	13371
Psmc1	10019	14512	12914	16846
Psmc2	13526.5	16816.5	15744.5	20592
Psmc3	40526	2775	40828	4585
Psmc3ip	14796.5	23378.5	17826	23057
Psmc4	23210.5	6747	6797	10102.5
Psmc5	10974.5	15799.5	32089.5	18188
Psmc6	14023.5	17896.5	13366.5	18613
Psmd1	40680	2052	38357	2784
Psmd10	16035	39619	17646	39507
Psmd11	15567.4	21112.4	22567.6	23710.8
Psmd12	22186.33333	11973.33333	21370.66667	10714
Psmd13	12352	15868	11951	17221
Psmd14	6916.333333	15564.33333	14129.66667	22492.66667
Psmd2	15362.33333	19870.66667	12330.66667	13338.66667
Psmd3	24625	24155	31056	36054
Psmd4	12892	11638	10797	14197
Psmd5	28799	38917	25543	36391
Psmd6	8114	13229	13245	16647
Psmd7	20574	22742.66667	18097.33333	23304
Psmd8	17535	20700	19415	22532
Psmd9	9722.5	12456.5	13080	14900
Psme1	20381	29902	24600	36885
Psme2b	26461	6100	30295	7416.5
Psme3	15175	19437	20874	24145
Psme4	18125.6	15602.8	11321.4	16981.4

Psmf1	3220	2637.666667	26102.33333	28033.33333
Psmg1	14300	17482	15882	19540
Psmg2	12778.5	15777	11684.5	13222
Psmg3	22649	26617	24445	25853
Psmg4	23422	37690	33809	32141
Psors1c2	13633	15681	15772	17592
Pspc1	16900.5	19355	17971	18210
Psph	3821.5	5727.5	3668	21057
Pspn	24008	26074	30017	36404
Psrc1	19665	21656.33333	16704.66667	22384.33333
Pstk	19261	23237	22616	22460
Pstpip1	2550	37240	40012	36219
Pstpip2	12189	25653	12250	31805
Ptafr	11544.5	10132	11258	12844.5
Ptar1	18697	19563	16759	18915
Ptbp1	37335	30003	28496.5	30011.5
Ptbp2	35599.75	21707.5	29232.25	31338.5
Ptbp3	36775	26498	14425	34357
Ptcd1	19681	21312	20135	19326
Ptcd2	17502.5	18546	25099.5	11910
Ptcd3	23283.33333	20666	22082.66667	23547.33333
Ptch1	18360.25	24188.25	24327.5	26526.75
Ptch2	16077.5	16367.5	19696.5	17657.5
Ptchd1	15739	19394	13932	19065
Ptchd2	35040	36420	40151	40115
Ptchd3	2306	7158	8819	12023
Ptchd4	16090.5	19416	17911.5	8813.5
Ptcra	33490	36407	2383	2642
Ptdss1	30306	22937	23946	24592
Ptdss2	20082	21954	38142.5	20794
Pten	15245.8	25294.2	24399	22516.4
Pter	28878	28906	28509	25254
Ptf1a	1758	2057	37844	4982
Ptgdr	40066.5	31403	31530.5	30152
Ptgdr2	12736	17832	20886	20947
Ptgds	20413.5	22878	30703.5	29282
Ptger1	18079	15557	17500	14020
Ptger2	7487	2000	5017	3023
Ptger3	29893	21297.33333	20515.33333	29938.66667

Ptger4	26735	25953.66667	28620.66667	23276.66667
Ptges	22319	15297	30048	22680
Ptges2	15717	18386	14649	15945
Ptges3	22610.33333	9544.333333	17865	19053.66667
Ptges3l	38529	29517	31016	29322
Ptgfr	28725.5	31492.5	31253.5	16541
Ptgfrn	6357.5	11931.5	12127.5	14553.5
Ptgir	27453.5	23658.5	31260	3656
Ptgis	1551	22645	3110	25126
Ptgr1	13249	39087	17348	8364
Ptgr2	22721	23537	24595	23656
Ptgs1	16574	11232	10513	6864
Ptgs2	20647	10027	6671	31264.5
Pth	38989	41058	1191	11189
Pth1r	24968	23922	21475.5	6687.5
Pth2	12841.5	18435.5	21446.5	25557.5
Pth2r	19406.66667	30636.33333	25690.33333	25535.66667
Pthlh	18223.5	21574	24048.5	25054
Ptk2	32477	22427.66667	30854.33333	33450.33333
Ptk2b	29620.5	18658.5	23387.5	18947
Ptk6	30868	35833	2834	1694
Ptk7	6608	1073	10040	9405
Ptma	27692.5	29788	28109.5	26365
Ptms	13568.5	24823	15773	14145
Ptn	22236	22192	26460.5	23993
Ptov1	20661	17832	14489	13246
Ptp4a1	8454.25	17420	17926.5	15698.5
Ptp4a2	19848	14320.33333	16399.33333	13925.33333
Ptp4a3	10968.5	16630.5	16525	18127
Ptpdc1	24536.33333	16054	24848.33333	16377.66667
Ptpmt1	21558	4837.5	22953.5	21784.5
Ptpn1	33870	12599	30647.5	31484.5
Ptpn11	19999.66667	14729.33333	10074	20233.66667
Ptpn12	20391.66667	22809.33333	21236.66667	25875.33333
Ptpn13	22883.4	22667.4	18734	25400.6
Ptpn14	30423.75	21120	12155.75	21395
Ptpn18	23753	17807	22528	8776
Ptpn2	24386.5	27682	24621.5	28154.5
Ptpn20	32941	35109	35469	33564

Ptpn21	23587	18089	19840	17390.5
Ptpn22	17030	11541	11909	3850
Ptpn23	27618	33010	23686	25673
Ptpn3	6790	7113	17102	17325
Ptpn4	20806	21909	24475	28286.33333
Ptpn5	36249	39950	3970	6033
Ptpn6	21227	10104.33333	7865.333333	9168.333333
Ptpn7	16539.5	16715.5	18132	17108.5
Ptpn9	27024.5	26067	27003	27846.5
Ptpra	29699.5	22993	27033.5	23813.5
Ptprb	30393.5	22501.75	24737.5	28212
Ptprc	20650.2	23263.8	29009	30329.6
Ptprcap	39435	2370	3670	3394
Ptprd	24582.125	23775.5	25057.375	24047.375
Ptpre	17624	17107.5	15497.5	19727.5
Ptprf	14048	24320.25	22163.25	24963.5
Ptprg	20549.33333	26145.33333	28717.66667	25476.66667
Ptprh	34482	28744	123	42
Ptprj	5505.5	9156	3654.5	9724
Ptprk	25811.5	26625.2	30374.3	26481.7
Ptprm	22131.2	19354	22266.4	25425.6
Ptprn	19234	20054	2286	10103
Ptprn2	38057	20380	33824	33911
Ptpro	21366	24857.66667	33649.33333	34012.33333
Ptprq	31288	33582	34057	32292
Ptprr	21027.75	23773.75	25630	24289.5
Ptprs	15209	14312	15263	16175.5
Ptprt	25599.33333	30746.33333	31264.66667	29254
Ptpru	16474	18835	21113.5	20683
Ptprv	26667	26968.5	16533	10320
Ptprz1	19047.75	17620.75	12566.25	15547.75
Ptrf	15951	7751.25	17899.5	13534.25
Ptrh1	3515	7040	8332	10105
Ptrh2	16286.5	22648	22010	24512.5
Ptrhd1	23087	30093	25601	22360
Pts	34509	19564	32062.5	32563.5
Pttg1	2097	8128	3010	4184
Pttg1ip	9572	5176	6837	7804
Ptx3	5414	18432.5	11243.5	20840

Ptx4	8669	9961	13158	12884
Puf60	18646.66667	16471.66667	15848	15892
Pum1	9000	7855	9345	9368
Pum2	23835.66667	19480	21236.33333	22180.33333
Pum3	20520.4	22093	19887.6	16839.2
Pura	4530	1273	1288	37662
Purb	31875.33333	21940	22530.33333	21727
Purg	25609	23022.5	6329.5	24278
Pus1	11685	16135.5	18291	20869
Pus10	26199	9504	28522	28913
Pus3	36407.5	12067	27828	9952.5
Pus7	26780	10425.5	9837	10129
Pus7l	16115	14664	16568	31583.5
Pvalb	25652	26961	28740	32464
Pvrl1	36418	35032	41055	40828
Pvrl2	21247.57143	23187.42857	21085.14286	31112.42857
Pvrl3	22415.66667	13522.66667	17154.66667	26463.66667
Pvrl4	22729	21796.5	20773.5	38413.5
Pvt1	21116.66667	17862	21849.66667	23432.66667
Pwp1	22227	26816	25511	29721
Pwp2	17323.5	16095.5	17661	17596
Pwwp2a	27851.75	24004	24709.75	26271.5
Pxdc1	19936	21620	27488	35125
Pxdn	21527.5	23130	24240	25670.5
Pxk	27784	25788	36596	34839
Pxmp2	37669	37627	40428	37260
Pxmp4	36631	34376.5	25925	24945.5
Pxn	27817.66667	16913	15472.66667	15266.66667
Pxt1	3887	5438	7106	5892
Pxylp1	15902.5	16126.5	16354	15546.5
Pycard	17542	22632	20461	21083.5
Pycr1	39110	6140	3234	8157
Pycr2	421	3817	2485	3522
Pycrl	14286	17125.5	15253.5	15885
Pydc4	2645	13189	1085	14998
Pygb	24786	23884	20094	17929
Pygl	26644.5	16718	21345.5	20819.5
Pygm	4079	1247	37256	33298
Pygo1	222	37006	39881	37606

Pygo2	21487	18915	16005	16488
Pyhin1	25337	16389.66667	3857.333333	7771
Pym1	33429	35597	36605	39659
Pyroxd1	6746.5	6391.5	7970.5	8616
Pyroxd2	33347	24198.5	29951.5	25018.5
Pyy	9988.5	11887	14575	13204.5
Pzp	18268.5	18834.5	33959.5	24554
Qars	18375.5	14256	13466.5	11829
Qdpr	13980	12174	10020	9255
Qk	14750.5	18342.5	16369.5	14264.75
Qpct	37645	25316	26747	19099
Qpctl	40271	40090	40730	831
Qprt	34604	38240	807	40577
QRFP	39675	2347	1118	1406
Qrfpr	18694.5	21055	18034.5	33658
Qrich1	31200	30472	31023	32793
Qrich2	33095	40011	1809	6291
Qrsl1	37532	33972	27391	29835
Qser1	10990.75	33357.5	37752.5	27905.75
Qsox1	16586.5	13806.5	17046.5	17443
Qsox2	38549	28293	31786	33385
Qtrt1	27097.5	26670.5	25449.5	26635.5
Qtrtd1	6495	1558	2394	3325
R3hcc1	6478	6005	9347	6903
R3hcc1l	30780.75	26576.25	19536.5	20280.5
R3hdm1	22567.55556	22014.88889	19193.88889	19288.11111
R3hdm2	22686	20532	22024	20190
R3hdm4	23750	25177	25606	24756
R74862	2374	39376	6223	1639
Rab10	34356.25	8696.5	29961.75	10408.5
Rab11a	10999.33333	12173.66667	10948.33333	14431.66667
Rab11b	15539.75	9096.5	8464	6369.5
Rab11fip1	29367	19684	32455.5	23042.5
Rab11fip2	9370	7716	13738	11427
Rab11fip3	34841.33333	24411.66667	16248	16287.33333
Rab11fip4	31303	30474	29928.5	27837
Rab11fip5	18192	16789.5	19578.5	14449.5
Rab12	28428.75	13409.5	23507.25	22412.25
Rab13	31720.5	31568	31268	30836.5

Rab14	11247.66667	17114	5357.666667	17073
Rab15	17850	19707	25669	27123
Rab17	19774	20444	31908	31455
Rab18	12007.66667	15427.66667	16393.66667	16210.66667
Rab19	34900	16689	22313	3104
Rab1a	26025	21630	21295	20361
Rab1b	8404	5740	23989.5	22522
Rab20	13519	28985	15503	27925
Rab21	13619.5	18655.5	19600.5	20483
Rab22a	17599.66667	20500.33333	18088.66667	20650.33333
Rab23	35440	38213	37769	18480
Rab24	3167	2693	1968	3913
Rab25	35319	31992	40999	39874
Rab26	20878	15737	15439.5	17500.5
Rab27a	16037.5	18473.5	16693.5	13082
Rab27b	25814	27126.66667	28726	29276
Rab28	19248.33333	15179.66667	20180.66667	14907.33333
Rab29	20161	16898	22372	15639
Rab2a	9889	13227	12347	14446
Rab2b	19180.5	17978	17410	16365.5
Rab30	35330	16179	33124	17935
Rab31	26781.33333	35822.33333	14744.66667	24385
Rab32	40263	9412	36129	9035
Rab33a	10851	21438	19409	32519
Rab33b	34613	33830	35480	34344
Rab34	25446	25998	21916.33333	23190.33333
Rab35	17317	16785	17552	17087
Rab36	33267.5	33554.5	15213	19731
Rab37	16338	18062	21906.5	16391
Rab38	29367	37790	23108	18836
Rab39	37478	22106	154	33271
Rab39b	23510	24601	26392	25413
Rab3a	31818	26855	24051	22763
Rab3b	26704.5	25341.5	30406	29429
Rab3c	31448	29820	17733	32046
Rab3d	29701	28848.5	25454.5	23733.5
Rab3gap1	19823.5	15627.25	16760	15507
Rab3gap2	25498	20075.5	22798.25	21758
Rab3il1	21223.66667	20524.33333	15354.33333	26345

Rab3ip	30311.5	17131.5	28886	15970.5
Rab40b	35130.5	31861.5	16843	14675.5
Rab40c	7504	23999	27607.5	25461.5
Rab42	14509.5	16427	13991	13472.5
Rab43	23834.25	23261.75	21820.5	21893.5
Rab44	20163	16598	30116	33868
Rab4a	34073	19259	24296	20252
Rab4b	20191	21135	17680	19434
Rab5a	16658	19039.66667	20363.33333	21023.66667
Rab5b	3953	4687.666667	4331.333333	5378.333333
Rab5c	25212.5	22739	12208.5	26248.5
Rab6a	11401	13353	13979.5	14980.5
Rab6b	17082.5	12812.5	13342	9900
Rab7	13053	12737.5	12510	13948
Rab7b	5290	35441	4399	39335
Rab8a	22639.5	28606.5	27773	32627
Rab8b	11273.66667	24872	18057.66667	14835
Rab9	1266	718	1551	1212
Rab9b	14531	19385	6869.5	3464
Rabac1	31966	32601	38828	36842
Rabep1	15565	19189	13990	16405
Rabep2	39940	36472	38601	37284
Rabepk	12558	14016	11636	11330.5
Rabgap1	22206.8	21416.6	17950.8	20104.2
Rabgap1l	19964	19195.33333	20092.33333	19152.16667
Rabgef1	17494	21573	22228	23192
Rabggta	14702	12873.5	13533.5	13505
Rabggtb	19413.33333	32225.33333	21187	24984.33333
Rabif	8692	9168	3924	5163
Rabl2	9573	15589	13481	15585
Rabl3	30271	33163	29275.5	14867
Rabl6	15429.33333	9463.666667	18849	16159.66667
Rac1	13779.5	29126.5	31417.5	24769
Rac2	13373	11574	11852	13345.5
Rac3	1422	10498	8162	14559
Racgap1	11641	10201	9189	8564.5
Rad1	6075	7900	6848	4809
Rad17	32584.5	36403	33297	35216.5
Rad18	18760.5	20039	17517	20850.5

Rad21	10957	12181.5	16385.5	14433
Rad23a	13792	12608	8813	10543
Rad23b	19287	18713	18095	22203.33333
Rad50	28872	25557.5	26584	21944.5
Rad51	9916	3564	10313	7566
Rad51ap1	334	38497	3959	1104
Rad51b	10704.75	35238.25	19805	26567.75
Rad51c	17064	16662	19462	16303
Rad51d	24190.66667	21781.33333	20665.33333	24509.33333
Rad52	22098	22100.5	19512.5	22277
Rad54b	23939.5	28293.5	25279.5	27536
Rad54l	24050.33333	19471	8815	30597.66667
Rad54l2	33614	29146	35894	39592
Rad9a	37244	34070	36744	35763
Rad9b	31538.5	32542	28636.5	14439.5
Radil	27411.5	28950	33846.5	38860.5
Rae1	18745	15504	16177.5	19310
Raet1a	5754	9110	6391	7614.5
Raet1e	35197	38636	40187	40816
Raf1	17920.6	26833.2	12849.6	25191.2
Rag1	29015.66667	34122	33926.66667	23120.33333
Rag2	29776	36885	32718	38754
Rai1	15673.5	9899.5	10141.5	2448.5
Rai14	8297.6	15148.2	15700.2	17937
Rai2	37521	26055	8278	14979
Rala	12377.5	14217.5	14098.5	13786.5
Ralb	13670	15673	19961	16439
Ralbp1	38682	39658	1271	275
Ralgapa1	17313.6	17299.6	22939	24150
Ralgapa2	14805.66667	21708	22274.33333	13513.66667
Ralgapb	14794.25	11353	21746.5	6662
Ralgds	37011	18939	37599	20147
Ralgps1	35865.66667	18989	22026.66667	7731.666667
Ralgps2	15201.5	20553.75	21864.25	24045.75
Raly	33173	29086	23184	32113
Ralyl	26061	27379.5	29043	27921
Ramp1	24321	20245.66667	31248.66667	30911
Ramp2	8598	2955	11905	40455
Ramp3	18257	15644	19486	20474

Ran	23499.83333	16441.66667	16894.66667	21190.83333
Ranbp1	7853	17278	16748	17268
Ranbp10	19787	34843	15541.5	34866
Ranbp17	33104	33858.66667	15272	14157
Ranbp2	29500	23159.5	26539	26209.5
Ranbp3	15950.5	15369.5	14163	12568.5
Ranbp3l	26392	28168	28172	27120
Ranbp6	16180.5	9208	19212	12084
Ranbp9	33188	22412	24480	23403
Rangap1	20017.5	19116	17806	18925.5
Rangrf	8258	12472	10573	13092
Rap1a	26731	10448.5	26749	27117
Rap1b	8085	11061	12104	14038
Rap1gap	14123	16901	18047.5	16174
Rap1gap2	9866	24183.5	23576	18226
Rap1gds1	16497.2	15661	13978.8	17847.2
Rap2a	21293.5	4802.5	15053.5	8018
Rap2b	12993.33333	9795	16265.33333	13208
Rap2c	14601.5	8064	27330.5	26516.5
Rapgef1	28687	19172	31628.66667	21406
Rapgef2	12297.33333	20548.66667	9140	25150.33333
Rapgef3	14085	2647	16653	4926
Rapgef4	27563.5	26211.16667	18784.5	18146
Rapgef5	28240	16280.2	19600.6	12648.8
Rapgef6	21615	21386.71429	23278.14286	19825
Rapgefl1	30554	32612	36599	32443
Raph1	24064	22845	24030.5	24027
Rapsn	13204	7193	11323	5104
Rara	20591	17609	15269	20163
Rarb	17507.5	19213.5	18242.5	17632.5
Rarg	18113.5	20923.25	22006	24534.5
Rarres1	5183	39901	40082	32006
Rarres2	30754.5	2726.5	3427.5	10075
Rars	37898	40406	34883	3184
Rars2	9582	24910.5	24728	25347.5
Rasa1	13376	21498	6730	20865
Rasa2	33872.5	18678.5	18664.5	21945
Rasa3	38819.5	25596.5	35630	24240
Rasa4	17041	18113	15252	17529

Rasal1	32694	28995	37709	30639
Rasal2	23928	20491.4	21871.6	21402.8
Rasal3	6966	40865	40342	32612
Rasd1	8751	10071	4114	5685
Rasd2	40445	35272	40235	38054
Rasgef1a	8580	12231	10973	12548
Rasgef1b	35285	20073.5	29914	21088.5
Rasgef1c	30942	32568	2377	32384
Rasgrf1	24230.75	26104.5	28526.25	29141
Rasgrf2	12605	2187	16288	17051
Rasgrp1	10933	3930	26514	24871
Rasgrp2	25030.66667	21784.66667	19767	13707.33333
Rasgrp3	8141	15141	6287	17225
Rasgrp4	8780.5	22102	10499	24982.5
Rasip1	20057.5	18272.5	15572	17495.5
Rasl10a	36444	34502	40301	37939
Rasl10b	16232.5	15480.5	17420.5	21937.5
Rasl11a	6947	35171	9026	18309
Rasl11b	1152	7111	7168	14154
Rasl12	37472	40433	383	885
Rasl2-9	18837	21715	20598	25139
Rassf1	35592	35265	36610	38424
Rassf10	40672	39805	12259	11029
Rassf2	13664	17370	14443.5	17553
Rassf3	19118	15599	17201	15807
Rassf4	19397.33333	17486.66667	17043.33333	25838.5
Rassf5	4193	38507	1207	32744
Rassf6	22218.5	22285.5	26377	28891.5
Rassf7	39909	1618	3830	5589
Rassf8	22062.5	28774.5	17559.5	13793.5
Rassf9	1979	10982	6130	2154
Raver1	16226	19555	8093.333333	8183
Raver2	24886.75	18202.25	18522.25	18340
Rax	28049.5	8296	12029	10448.5
Rb1	20136.66667	20717.33333	27867	27709
Rb1cc1	18501	15916.5	14005.16667	13316.5
Rbak	34586.5	13349	13614	34612.5
Rbbp4	19352	17834	17619	17576
Rbbp5	12190	11596	12361	12723

Rbbp6	16851.25	13998.25	16741	14276.5
Rbbp7	16946	27020	12276.66667	17181
Rbbp8	21165	20950.33333	21816	25175.66667
Rbbp8nl	22347	24197	24889	23894
Rbbp9	24688.5	27699	24798.5	23040
Rbck1	38251	32342	31969	27566
Rbfa	16814	11234	7972	7294
Rbfox1	20028.2	21058.6	29711.6	23726
Rbfox2	27888	7232.5	12338	17603
Rbfox3	17928	19775.5	23212	24299.5
Rbks	19134	21272	17518	18971
Rbl1	9960	8069	11224	8694
Rbl2	12512	16886.5	14538.5	32622
Rbm10	34336	28739	31874	31704
Rbm11	22284	16157	29134	7017
Rbm12	9495	5070	5375	6886
Rbm12b1	31916.66667	8459.666667	17632.33333	17859.66667
Rbm14	1733	40890	4246	4527
Rbm15	10597	8044	11804	11461
Rbm15b	18382.5	21924	21264	21886
Rbm17	5150	6701	7269	6138
Rbm18	25166.66667	22752.66667	23107.33333	22006.33333
Rbm19	30796	25178	25412	28222
Rbm20	21957.5	24828.5	29339.5	26020
Rbm22	26753.5	25113	21278.5	22756.5
Rbm25	18231	19416.25	12138.75	11264
Rbm26	11067.4	14110	15542.4	15803.8
Rbm27	21739	14607.5	14048.5	13687.5
Rbm28	7809	17141	14683.75	15630.5
Rbm3	35387	40450	35878.5	36249
Rbm31y	18102	20399	22047	19726
Rbm33	24443	22579	22313.66667	25677
Rbm34	32541	36424	37825	38183
Rbm38	15477	18674	15470	17269
Rbm39	17398.66667	15530	15215	15744
Rbm4	18446.16667	22256.5	18146.5	18461.66667
Rbm41	15142.33333	33885	27540	27383.66667
Rbm42	3056	1721	2686	3389
Rbm43	35693	27770	24552	27154

Rbm45	10434	13101	16658	16253
Rbm46	36486	35777	30081	2020
Rbm47	26182.5	20336	28524.5	28300.5
Rbm48	9765.333333	15152	11122.66667	10034
Rbm5	7890	4107.666667	6931.333333	18816
Rbm6	34981.4	26091.6	21306.8	28531.4
Rbm7	13332.5	23007.5	13241	21886.5
Rbm8a	12288	18970.5	19141	20058
Rbms1	19736.33333	19221.33333	16796.5	13117.5
Rbms2	921	39570	4493	39760
Rbms3	25920.33333	22935	13185.33333	19912.66667
Rbmx	25790.66667	14994	26942.33333	13880.66667
Rbmx2	22056	25104	24903	25426
Rbmxl1	1467	1469	246	122
Rbmxl2	10946	5260	22452	16783
Rbmy	40725	39368	27798	26593
Rbp1	12094.5	11223	25194.5	25996.5
Rbp2	14264.5	15782	21230.5	19974.5
Rbp3	26017	27440	38870	2305
Rbp4	20598	15045	17575	15194
Rbp7	28998	34731.5	36531.5	15548
Rbpj	22416.625	22336.375	20324.875	21027.875
Rbpjl	37339	39286	2600	871
Rbpms	11978	25335	11729.33333	21829.33333
Rbpms2	35065	23922	2804	3429
Rbsn	21187	14540.66667	15617.66667	24837.66667
Rbx1	23689	28117	25603.66667	26559.33333
RC10	27911	29370	30854	29518
RC11	28028	35823	30979	31853
RC12	34805	31267	32680	29981
Rc3h1	29167.66667	23958.66667	24113.33333	21259.33333
Rc3h2	15979.5	16884.25	18094.25	15337
RC4	27958	35015	30839	29520
RC5	34861	37245	31726	31139
RC6	29002	30082	33562	31113
RC7	33493	38848	31415	30644
RC8	28493	32119	31226	29816
RC9	32805	32741	31281	29956
Rcan1	14548	18019.5	18036.5	18915

Rcan2	26120.33333	23669	25973.33333	27111.66667
Rcan3	18119.5	16592	16984	15180
Rcbtb1	21766.33333	29161.33333	17888	19191.33333
Rcbtb2	11828.6	9953.2	7781.4	19372.2
Rcc1	33284	36848	28949	34261
Rcc2	18445.5	24045	18710.5	22487
Rccd1	24439	22301.5	20709	20896.5
Rce1	10176	12639	12533.5	13139
Rchy1	26637.66667	25557	13607.33333	11046
Rcl1	7560.5	11262.5	26510.5	9669
Rcn1	4337	11257	10488	15094
Rcn2	18490	17095	13587	13558
Rcn3	22211.5	21260	27338	31990
Rcor1	11990.75	17865.25	23082.25	19551.5
Rcor2	1323	4041	11513	15443
Rcor3	9278.333333	30908.66667	24315.66667	23944
Rcsd1	23634	27097.5	20120	22873
Rcvrn	21808	28950	21098	31585
Rd3	23801	26200	33204.5	36627
Rdh1	2526	36069	6509	29991
Rdh10	14302	17627.5	15669.5	20562
Rdh11	23281.5	27561	22062.5	27113
Rdh12	40741	2400	3349	3632
Rdh13	14164	14554	10435.5	13671
Rdh14	13223.5	15434	13430.5	33245.5
Rdh16	28141	3929	1253	7179
Rdh18-ps	34452	34839	4628	5522
Rdh5	2267	1116	5569	5260
Rdh7	142	13698	1480	8578
Rdh9	18391.5	20181.5	23033	24622.5
Rdm1	3769	39918	4881	37524
Rdx	32618	25603.33333	24458.33333	22190.66667
Rec114	28863	25330.5	15360.5	22448.5
Rec8	17359.5	18677.5	22065	22713.5
Reck	17095.5	16085	26334.5	21881
Recql	39696	20916	37899.5	38346.5
Recql4	41	39226	37378	34417
Recql5	16497	13089	13779	12817
Reep1	20470	23196	35449	28856

Reep2	36228	40630	30955	29829
Reep3	19878.33333	18715.33333	15098.66667	15780.33333
Reep4	11252	10339	7484	7724
Reep5	29070	24380	22125	22311
Reep6	24210	33642	257	41146
Reg1	1226	39373	3469	2427
Reg2	4919	4292	3336	7562
Reg3a	23294	39066	1471	29577
Reg3b	28114	21999	33122	30714
Reg3d	35083	36646	26846	25692
Reg3g	25382	26477	28547	40794
Reg4	25626	26748	29350	28078
Rel	26808	28194	29983	32725
Rela	22019	6219	38307	1691
Relb	29461	13392	27360	9960
Rell1	15447	8819	16767	10112
Rell2	34329	35190	35911	34926
Reln	12175.25	17913	22717.5	16904
Relt	13155	12481	7245	7419
Rem1	2524	37526	32154	24408
Rem2	23013	20431	24457	26349
Ren1	18463.33333	23317.66667	15331	13780.33333
Renbp	19662	18621	18988	22151
Rep15	12230	10249	15104	15512
Repin1	3153	4458	4398	5029
Reps1	25276.33333	21481	15006.33333	3293
Reps2	12903	19059.5	22478.5	17487.5
Rer1	15526	15831	16546	16034
Rere	21922	20236	22214	25499
Rerg	33442.5	8434	18825	19208
Resp18	11023	12696	14840	9136
Rest	27251	28647.66667	18448.33333	22213
Ret	37869	2081	6964	10067
Retn	10173	15440.5	18525.5	21257
Retnla	27301	16039.5	15435	15478
Retnlb	23572	24638	26562	25531
Retnlg	38586	29707	37623	28842
Retsat	9415	7248	3646	40743
Rev1	32474.5	28321	28701	31282.5

Rev3l	16926	11726.6	19742.2	23286
Rex2	9027.5	12955.5	12506.5	13656.5
Rexo1	8732	27116	17770	17700.5
Rexo2	18553	26252.5	19473.5	24695.5
Rexo4	6231	8688	6897	7676
Rfc1	30336.5	18696.5	19989.5	15808.5
Rfc2	15394.5	13309	10975	28887
Rfc3	35793	38731	35664	35818
Rfc4	7726	10190	10095	8464
Rfc5	25715	23628	21387	20157
Rfesd	5146	5828	3696	3713
Rffl	11837	19697	14120.33333	21628.33333
Rfk	10592.33333	9958.333333	24053.66667	23584.66667
Rfng	2416	639	40843	39269
Rfpl3s	28593	25219	37439	26309
Rfpl4	11089	18732	18136	21091
Rft1	22291.5	15225	3719	2687
Rftn1	22467.33333	17984	7091	32859
Rftn2	19296	17717	19068.33333	16119
Rfwd2	21297.75	20529.5	19311.5	18484.5
Rfwd3	22138.5	30285	39352	21822.5
Rfx1	22937	22745	22403.5	20353.5
Rfx2	29805.5	11394	31228.5	33315.5
Rfx3	18531.5	22828.75	15862	16622
Rfx4	23106	27738.5	7761	11675.5
Rfx5	26582.5	17059.5	32726.5	19930.5
Rfx6	28421.5	30388	32592.5	15674.5
Rfx7	16937.5	15025	26522	26075.25
Rfxank	5843	7587	5646	5844
Rfxap	16440.5	16851.5	19597	17731
Rgag4	18598	11636	15069	8337
Rgcc	17467	34657	21458	34914
Rgl1	16598	11993.5	12974.5	14303
Rgl2	10913	4027	12547	5716
Rgl3	15292	17783	24964	25102
Rgma	24053	29102	25760	35945
Rgmb	11702	25927.75	23013	16161
Rgn	20845	22931	27282	34613
Rgp1	23970.5	20739	25843.5	24982.5

Rgr	31597	29035	30354	29217
Rgs1	14128	7721	20470	17664
Rgs10	22048	18528	25542	19357
Rgs11	6687	7591	12778	12676
Rgs12	30272	12428.5	14203.5	15341
Rgs13	39062	7622	9204	19200
Rgs14	20418	18778	19975	15815
Rgs16	1185	4419	2198	9898
Rgs17	436	29226	31755	2690
Rgs18	16653.5	20672	13082.5	28751.5
Rgs19	22233.5	21747	19201	18716
Rgs2	18491.5	7736	18011.5	25042
Rgs20	12780	7796	19301	20429
Rgs3	21545.6	19816.2	20469	26140.6
Rgs4	17240	17753	34738	29117.5
Rgs5	23352	25026	14351.5	10878
Rgs6	24975.5	31252.5	31939.5	18136
Rgs7	30880	1738	33689	3962
Rgs7bp	29061	30425.33333	33316	32093
Rgs8	20699.5	18742.5	10883	29893
Rgs9	5736	6405	13998	12903
Rgs9bp	14719	19947	5631	5130
Rgsl1	11032	22132	9171	23138
Rhag	7717	40592	10744	17671
Rhbdd1	26997.5	7737.5	27211	7306
Rhbdd2	21541.2	18891	20943.2	20702.6
Rhbdd3	38283	37172	38078	37301
Rhbdf1	2114	498	1317	3445
Rhbdf2	19732	21329.5	15370.5	22228.5
Rhbdl1	11513	11395	13744	14993
Rhbdl2	28418	12067	13021.5	13001
Rhbdl3	23667.66667	24221.66667	26586	26293.33333
Rhbg	10567	13175	19356	14839
Rhcg	1772	1604	7486	7217
Rhd	38931	25738	30995	4588
Rheb	22970	27073	28700	29691
Rhebl1	1968.5	8107.5	7577.5	8679
Rho	25478	35938	40480	1291
Rhoa	11323	11598.5	13234	33423.5

Rhob	16982	14983	15964	16060
Rhobtb1	19347.5	27539	19519.5	32231
Rhobtb2	17157.5	25546.5	29664	27167
Rhobtb3	16031.5	20887.5	23011.5	29366
Rhoc	12156	10035	12099	12553
Rhod	5305.666667	18987	21601.66667	20609.66667
Rhof	2738	39471	32777	9730
Rhog	9538	9390	5854	10539
Rhoh	16632	39779	15473	35856
Rhoj	15289.66667	31234	26265.33333	28449.66667
Rhoq	36811.5	5190.5	34220	5985.5
Rhot1	16993.2	20059.8	20626	21745.4
Rhot2	21401	20591	21925.5	21625.5
Rhou	37722	3589	40631	9082
Rhov	19767	13162	20590	17350
Rhox11	725	90	29959	6290
Rhox13	2207	36346	39085	1444
Rhox2a	1711	38101	37303	36240
Rhox3a	12327	14143	10319	17191
Rhox4e	33275	37808	2885	5051
Rhox5	34617	5033	8399	10904
Rhox6	15151	11716	34151	35662
Rhox9	24269	23906	37964	37371
Rhpn1	7146	8723	11742	16541
Rhpn2	15531.5	20278	19445	24251.5
Ribc1	13258	18694	20942	23203
Ribc2	16162	12910	23401	19072
Ric1	19560.66667	17919.33333	19652	31215
Ric3	29644.6	32952.2	27998.6	22159
Ric8	5026.5	4439	5493.5	5167
Ric8b	12998	13890	12988.5	16193
Rictor	21554.625	20733.125	24116.5	17763.25
Rif1	27526.5	21273.5	18009	18815
Riiad1	26007	9859	25225	20052
Rilp	25464	23670	23910	23627
Rilpl1	27746.33333	31177.33333	31601	31880
Rilpl2	36111	35740	37993	36158
Rimbp2	18917	20293	37685	36476
Rimkla	31258	23570	1005	39881

Rimklb	30237.5	223	21052	7882.5
Rims1	13908	18724.5	20888.5	22062
Rims2	24910.5	28309.5	20858.75	28332.75
Rims3	3074.5	18553	10818.5	11183.5
Rims4	15515	17367	17788	18267
Rin1	13812	16586	17469	18276
Rin2	99	36904	2409	38880
Rin3	30428	16660	23189	10324
Ring1	35416	36357	37465	36756
Rinl	14058.66667	12915.66667	13176.66667	12118
Rint1	35802	31248	28888	29393
Riok1	21708.5	19781.75	17524.5	16375.75
Riok3	11318	18881	11509.66667	8912
Ripk1	19741.33333	16474	15438	14781.33333
Ripk2	15711	19889	14326	19456
Ripk3	23098	23225	23270	25668
Ripk4	28179	28031	41003	27090
Ripply3	21622.5	19682.5	27994	22680
Rit1	15059	17473.5	15018.5	16428
Rit2	20892	22505.5	23932.5	23158
Rita1	7803	5627	1517	3431
Rlbp1	7862	37791	40544	29461
Rlf	18234.75	16297	9728.25	19609.25
Rlim	17536.5	28084.25	18825.25	29125.5
Rln1	3290	2801	401	12105
Rln3	14950	17170	17280	16255
Rmdn1	15762	20461.5	20333	19685
Rmdn2	28674.5	27601	22947.5	20511
Rmdn3	11370.33333	15422.33333	12920.66667	14369
Rmi1	17224	12912	16392	12220
Rmi2	10675	28667.5	17752	21276
Rmnd1	25291	21763.75	13789.75	18639.5
Rmnd5a	15705.6	14626	14612.4	15420.6
Rmnd5b	16366	14614	10887	9509
Rmrp	37820	4131	5465	2138
Rmst	27432	29200	29230	28015
Rnase1	30671.5	13609.5	18112	21100.5
Rnase10	17783	36072	25043	38837
Rnase2a	7620	40999	6482	1434

Rnase2b	33673	32927	33700	32896
Rnase4	6802.5	31592	23877	22177.5
Rnase6	15796	27452	7200	10787
Rnase9	34774	4050	13980	7171
Rnaseh1	21831	23596	25048	25860
Rnaseh2a	18039.5	21522.5	19943	20926
Rnaseh2b	28784.5	18351.5	32897.5	33527
Rnaseh2c	12730	17761	16062	18509
Rnasek	980	37741	39982	617
Rnasel	21402	23397	14870	21078
Rnaset2b	15872.25	18953.75	13808.75	14937.5
Rnd1	20191.33333	21324	15010	19998.33333
Rnd2	32002	23363	25662	22712
Rnd3	21849	19114.33333	27625.33333	7723
Rnf10	36540	31485	39954	37777
Rnf103	15094.5	14104.5	12448	11585
Rnf11	21325	24697	20763	25777
Rnf111	21387.33333	12104.33333	14833	13184
Rnf112	26138.5	1701	13134	12181
Rnf113a1	3918	9857	5638	9686
Rnf113a2	21849.8	20270.2	13964.8	19289.8
Rnf114	12605.5	11395.5	8070	12562.5
Rnf115	10275.5	16867.5	15366.5	20442.5
Rnf121	17483.33333	18933.33333	5429	7736.666667
Rnf122	4501	7696.5	18446	3474.5
Rnf123	9846	10700	22046	22588.33333
Rnf125	39929	27237	39934	33066
Rnf126	10537	10898	8168	9828
Rnf128	18397	14774	17683	17544
Rnf13	20537	8451	20618	20096.33333
Rnf130	24653.5	24273	23578	24061.5
Rnf133	4012	40701	8921	11443
Rnf135	4917	3744	3529	3071
Rnf138	17806.33333	13642	15167.33333	13809
Rnf139	23782.66667	25180.66667	18161	24688.66667
Rnf14	6673	11213	3961	10335
Rnf141	20649.25	25304.5	20447	26076.25
Rnf144a	13578.5	30836.5	6552	29147
Rnf144b	19214	15364	20362	35545.5

Rnf145	17734	19844	20472	19272
Rnf146	20654	9131.666667	9619.333333	10451.33333
Rnf148	38055	921	2805	5632
Rnf149	24230	22852.5	27565.5	23571
Rnf150	17185.5	11004.25	8523.75	16368.25
Rnf151	20060	21685	38100	38993
Rnf152	2892	15269.66667	17214.66667	9952.666667
Rnf157	19850	23008	25497	19282.4
Rnf165	30128.5	31969.5	32845.5	31474
Rnf166	6048	38935	31828	24989
Rnf167	8883	7719	10798.5	10734
Rnf168	17861.5	13005.5	8680.5	7089
Rnf169	21827	17721	20681	18542
Rnf17	27600	25004	11946.5	27405.5
Rnf170	11061.4	25355.2	22780.2	26159.6
Rnf180	10906.5	16143.5	26103	11301.5
Rnf181	35362	40053	30512	38584
Rnf182	35557	24093	40849	39105
Rnf183	37172	36670	1931	996
Rnf185	12294	13744	11641	14022
Rnf186	16906	15535	20840	24790
Rnf187	13791	9571	9301	6226
Rnf19a	31900.33333	20884.66667	32909.66667	24068.66667
Rnf19b	18183	28305	18378.5	28158
Rnf2	27928.5	9876.5	28465.5	10313.5
Rnf20	23603	23933	20631.66667	17628.33333
Rnf207	23699	24858	26415	27033
Rnf208	29585	39464	36293	36406
Rnf212	15891	17025	23064	22865
Rnf213	294	11080	2274	16002
Rnf214	12047.4	15953	13324.8	14105.4
Rnf215	35144	27021	27415	28684
Rnf216	19115.5	21618.75	12074.75	3964.25
Rnf217	24788.33333	31638.66667	24545	30019
Rnf219	27780	27086.5	28533.5	27977.5
Rnf220	13178.5	9485	9848	7998
Rnf222	7212	6834	14748	14398
Rnf225	4571	38160	8723	11018
Rnf24	17822	20985.66667	17348	23969.33333

Rnf25	20623.33333	26082.33333	25950.33333	25841.33333
Rnf26	33	2491	9286	2188
Rnf31	17404.5	17372.5	18039	19922
Rnf32	19913	21703	21444.33333	24613.33333
Rnf34	34556	28545	27304	27135
Rnf38	22499	22748	24923.66667	23563
Rnf4	23428.33333	18427	15415.66667	18247
Rnf40	6354	2535	803	64
Rnf41	16238	16002	15246.25	14496.5
Rnf43	31993.5	26491.5	27875	25895.5
Rnf44	26426	16771.5	26230	22641
Rnf5	36680.5	25290.5	29308.5	25990
Rnf6	20352	14619	12452.66667	11586
Rnf7	9179.5	13026	13658.5	11609.5
Rnf8	12627	9287.666667	8182.666667	10161.66667
Rnft1	28287	25775	27560.5	25804
Rnft2	18031	17267	10449	39567
Rngtt	19514	16533.5	17882.5	18209.5
Rnh1	37976	23657	32036	26246
Rnls	30812	36559	28706	29793
Rnmt	24872.16667	18891.16667	22892.33333	22358.33333
Rnmtl1	20141	23281	21650	23846
Rnpc3	10566.5	12334.5	11791	12319.5
Rnpep	4687	2678	5055	3871
Rnpepl1	22397	15183	20220	15946
Rnps1	3515	4890.5	2439.5	7122.5
Robo1	8852.5	13153	16373	18738
Robo2	24436.375	27480.375	19486.75	25427
Robo3	17933.5	20223.5	3320.5	2712.5
Robo4	30854	27180	28588	28847
Rock1	16012.33333	17742	18873	27366.66667
Rock2	10728	21120.5	19188	18073.5
Rogdi	20866	20678	16419	17319.5
Rom1	8118	7445	7784	7223
Romo1	22627	32547	26255	23781
Ropn1	38986	2048	35120	4787
Ropn1l	17583.5	15365.5	13751.5	13351.5
Ror1	25869	27313.5	18450	17375.5
Ror2	20605	20282.5	25317	13657

Rora	18856.5	18744.5	14595.75	12744
Rorb	31550.75	33045	35330	24158.75
Rorc	31309	33543	35195	2677
Ros1	26628	28029	29466	28467
Rp1	35945	36243	3168	21417.5
Rp1l1	5276.5	11423	11301	16521.5
Rp2	23345.5	18122.75	23532	20059.25
Rp9	8949	8841	8511	6252
Rpa1	15155	11665	15511	13905
Rpa2	18204.5	22394.5	15596	20521.5
Rpa3	37510	37979	37875	36238
Rpain	21090	23307	25244	24941
Rpap1	13744	7815	12209.5	8104.5
Rpap2	4989	5497	5169	5439
Rpap3	14744	16821	16141	17186
Rpe	26866.66667	16507	26479.66667	16669.66667
Rpe65	29506	31349	31547	30122
Rpf1	31227.33333	38322	37578.33333	13352
Rpf2	35301	38224	34305	37342
Rpgr	18044	19894.66667	17925	18192.66667
Rpgrip1	17600.83333	22017.33333	22845.66667	24107.16667
Rpgrip1l	21437.28571	26510.42857	22089.71429	26460.85714
Rph3a	24974	28375.5	32361.5	30030.5
Rph3al	8306	13608	18588	12309
Rpia	21884	22379	16540	19732
Rpl10	31613.5	24368.5	30867.5	30007
Rpl10a	15899	12776	18214	15828
Rpl11	16190.5	17635.5	19640	22529
Rpl12	6333	5561	13339	10364
Rpl13	16319.33333	13431	19311	17694
Rpl13a	31112	30427	32446.5	29209
Rpl14	5451	9224	9290	9931
Rpl15	30282	27404	28708	27827
Rpl17	14302.5	16678.5	23588	22365.5
Rpl18	24551	32569	33671	29684
Rpl18a	21391.5	20245	2995.5	1571.5
Rpl19	13301.5	21383.5	18336	18456
Rpl21	21749.5	22478.5	22677.5	22499.5
Rpl22	29189	30643	33225.5	31768

Rpl22l1	17465	17632	12507	11358
Rpl23	22983.5	23633.5	2434	1871.5
Rpl23a	21455	14225.33333	27157.66667	27138.66667
Rpl23a-ps1	20623	15613	23552	18727
Rpl24	7620.5	14548	8242.5	13326.5
Rpl26	39540	40662	3031	2180
Rpl27	5530	7424	9113	9702
Rpl27a	13	2875	40851	39629
Rpl28	2193	682	4294	39979
Rpl29	18259	12023.5	17743.75	14350.75
Rpl3	38463	33916	39492	39905
Rpl30	21841.5	24892	29248.5	30194.5
Rpl31	9960.5	11178	10550.5	9694
Rpl32	20657	2241.5	12985	11900
Rpl34	36840	17465.5	15041	12397.5
Rpl35	37451	40586	2944	1946
Rpl35a	19502	24116	27829	25338
Rpl36	27647	29718	33187.5	29502
Rpl36a	25854.66667	25493.66667	26905.33333	27224.66667
Rpl36al	12329	17133	18298	17562
Rpl37	18362.5	19871.5	25329.5	23317.5
Rpl37a	11784	9849	13434	12862
Rpl38	3249	8143	11652	10911
Rpl39	36678	37706	41111	40456
Rpl39l	8270.5	24846.5	18903.5	26102
Rpl3l	3667	12359	13629	9923
Rpl4	15434	19263	21596	22804
Rpl41	18769.5	17334.5	26385	22029
Rpl5	9859	10490	11906	11491
Rpl6	31235.5	31345	13561.5	13630
Rpl7	28344.33333	29831	5936.666667	18809.66667
Rpl7a	7928	10817.66667	11958	9410.666667
Rpl7l1	15384	17014.5	19911.5	21354
Rpl8	8217	9841	14504	13257
Rpl9	30119.5	11614.5	11708	13576
Rplp0	12123	13455	14425.66667	16105
Rplp1	15199	16757	19009	17382
Rplp1-ps1	22953	26197	5993	4448
Rplp2	23636	24275.5	23703.5	25175.5

Rpn1	16959.33333	7418.666667	17983.66667	6794.666667
Rpn2	35841.5	28685	26263	32048
Rpp14	8939	11962	7473	8067
Rpp21	10414	9393	11178	5328
Rpp25	13736	120	17925	11892
Rpp25l	35491	30991	34000.5	30645.5
Rpp30	15170	16394	13792	16327
Rpp38	4888	10666	10139	12476
Rpp40	23615.5	24320	20244	25753
Rprd1a	12270.75	22435.5	20029.25	23303
Rprd1b	20648.5	14837	15535.5	13802.5
Rprd2	16579.5	18195	22790.25	22737.375
Rprm	20020	14277	20910.5	12525.5
Rprml	22874	21833	24675	23438
Rps10	25290	30242	34295	34098
Rps11	16811	30534	19136.5	12124.5
Rps12	6050.5	6013	7385.5	6243.5
Rps13	11246.5	10633	14617	12458.5
Rps14	13522.5	19502	21327	22051.5
Rps15	6447	2194	11334	9568
Rps15a	32346	14517	12860	38383.5
Rps16	14747.5	16301.5	20818	19184
Rps17	18459	19143	19894	19327
Rps18	8732.666667	20171	8665.333333	18174
Rps19	23984	18531	19577	28663
Rps19bp1	20461.5	26163	25100.5	26810.5
Rps2	15998	16793	12966.8	13000.8
Rps20	10240.5	12106	12087	9515
Rps21	20871.33333	10530	12737.33333	21173.33333
Rps23	13981	14522	17165	17388
Rps24	26708.33333	24377	11276.33333	25906.66667
Rps25	1720	6603	10508.5	9567.5
Rps26	21911	18867	20476	20203
Rps27	5596	6470	11191	7322
Rps27a	15914.5	38131	37175.5	18278.5
Rps27l	15137	19669	19870	18347
Rps28	8171.333333	18254	19304.66667	14017.33333
Rps29	8237	8347	15400	9340
Rps3	2230.5	2075.5	22504	21362.5

Rps3a1	39061	21435	4216.5	3493.5
Rps4x	20824.5	22609.5	22370	23261
Rps4y2	16247	22482	21128	24249
Rps5	35243	40250	3516	41072
Rps6	33796.5	21479	39536.5	23924.5
Rps6ka1	28744.66667	27515	28074	28739.66667
Rps6ka2	570	11949	5655	11971
Rps6ka3	30058.75	28651.75	22655.75	32075
Rps6ka4	30848	35324	25958	31605
Rps6ka5	32405	33367.33333	21842	35320
Rps6ka6	26569.5	27833	30197.5	28812.5
Rps6kb1	17037.66667	26656.16667	33325.33333	17148.66667
Rps6kb2	15895	13400.33333	17046.66667	15438.33333
Rps6kc1	32692.66667	22002.66667	31529.66667	22176
Rps6kl1	28277	36672.5	34005	33676.5
Rps7	11769.66667	26487.66667	19661.66667	6167
Rps8	7741.333333	11692	13619.66667	11009.66667
Rps9	16009.5	17044.5	16367.5	16703.5
Rpsa	10748	17451.5	17231.5	16025.5
Rptn	30036	32752	32375	31015
Rptor	13572.5	18874	21267.25	20709
Rpusd1	38332	30441	26904	26210
Rpusd2	23679	24828	26148	34627
Rpusd3	5689	6586	5217	7715
Rpusd4	23430	21675	23571	22051
Rqcd1	37687	38677	31684	34889
Rrad	10101	36478	14856	36191
Rraga	2639	2349	5937	4370
Rragb	25299	26601	7295	7278
Rragc	16555	30022	12267.5	31834
Rragd	21366.66667	15950.33333	16052.33333	13871.66667
Rras	24607	37213	33376	39479
Rras2	13096.66667	13592.33333	17507.33333	14443
Rrbp1	29102.2	24408	25132.6	23634.4
Rreb1	18798	15169.33333	3751.333333	14903
Rrh	31310	32921	34422	32626
Rrm1	22863.5	23673	25772.5	24445.5
Rrm2	5039	4077	7365	8770
Rrm2b	34211	26303	27321	32598

Rrn3	13087.5	11800	10382.5	12160
Rrnad1	21051	21090.5	20889	21202
Rrp12	15886	14061	11761	16477
Rrp15	18291	19036.5	18933.5	19837.5
Rrp1b	15003.5	13927.5	14258.75	14270.75
RRP1B	1276	32925	28204	26414
Rrp36	32335	32008	34962	33784
Rrp7a	8169.5	8551.5	10795	11723.5
Rrp8	18562	18262	16570	16182
Rrp9	2614	5600	2272	4231
Rrs1	18979	33143	22102	33423
Rs1	26700	26944	28895	27689
Rsad1	2891	39102	37525	38713
Rsad2	24953	19272.66667	5954.333333	15840
Rsbn1	21566.66667	15745.66667	20695.66667	20974.66667
Rsbn1l	19742.66667	22630.66667	21878.66667	24795.66667
Rsc1a1	14897.5	27587	15870	8241.5
Rsf1	24769	24609.5	24948.25	34484
Rsg1	34006	40792	3218	12672
Rsl1	1402.5	6304	1567	5882
Rsl1d1	35395	171	40675	3768
Rsl24d1	2693	4504.5	2026.5	5958
Rsph1	20172	14780	22079	10871
Rsph10b	26873.5	15192.5	23442	23144.5
Rsph10b2	12881	9446	13860	18480
Rsph14	20375	21068	37898	34934
Rsph3a	19681.33333	16334.66667	18990.66667	13670.33333
Rsph4a	24784	26093	34538	26287
Rsph6a	21055.5	19957.5	23847.5	24072.5
Rsph9	20011	31180	20799	24826
Rspo1	36532	36329	984	483
Rspo2	28204.5	30246	31632	31510.5
Rspo3	20042.75	17939.5	16043.75	18739.5
Rspo4	8797.5	3070.5	11848.5	12134
Rspry1	8928.333333	8706.666667	5586.666667	8977.333333
Rsrc1	22051.4	21406.6	20920.2	20511.8
Rsrc2	37949	37758	40224	38651
Rsrp1	3081	37087	167	39250
Rsu1	7112	6977	9563	9309

Rtbdn	36885	39995	4348	5100
Rtca	19805	22239.5	18530	22091
Rtcb	36562	34847	26260	31486
Rtel1	18843	16778	16965	15427
Rtf1	12396.4	24425.2	26651.4	24984.8
Rtfdc1	23889.75	17918.75	16059.25	17751.5
Rtkn	18154	19963	21945	24837
Rtkn2	18991.33333	17962.66667	21331.33333	25516.66667
Rtn1	13616.5	25803	21269	13168.5
Rtn2	7687	23184	12481	27551
Rtn3	12865.25	20750.75	26904.5	18463.75
Rtn4	18577	16602.4	14221.8	14202.4
Rtn4ip1	31263	34803	27552	34024
Rtn4r	38554	6895	17679	27128
Rtn4rl1	6219	35035	14488	4280
Rtn4rl2	39816	1723	5439	4421
Rtp1	18387	19515	17798	20127
Rtp2	36685	5748	7031	6599
Rtp3	35432	38662.5	3169	20095
Rtp4	22863	12620	36830	16040
Rttn	17262.25	9249.5	18381.25	7131
Rufy1	19997.5	39561.5	20046	36363.5
Rufy2	23878	23093	5293	23305.5
Rufy3	11058	23882	27594.33333	21314.66667
Rundc1	8408	6477	5309	3601
Rundc3a	15356.66667	28308.33333	28263.33333	29620.33333
Rundc3b	33524	37238	32411	32228
Runx1	15820	13427	25657	26799.66667
Runx1t1	30599.5	22743.5	37609.5	25738
Runx2	25451.5	24457.16667	19251.5	20293.83333
Runx3	27145.5	28118.5	31267	32108
Rusc1	28148	20642	20256.5	26040.5
Rusc2	21058	20425.5	20665	20638.5
Ruvbl1	39976	1049	1024	3250
Ruvbl2	9373	11233	12372	13216
Rwdd1	23188	14575	17078.66667	14337.33333
Rwdd2a	18204	18448	10825	9682
Rwdd3	20514	21315.5	24917	26663.5
Rwdd4a	24191	26547.5	20729	25439.5

Rxfp1	27854.33333	26462.33333	28484.33333	28306
Rxfp2	26364.5	26638.5	37423	19719.5
Rxfp3	39056	31515	32919	35264
Rxfp4	6329	4258	10255	6780
Rxra	13432	9044	10920.5	28294.5
Rxrb	29667.5	28277.5	21834.5	21065
Rxrg	22177	22712	29402	34138
Rybp	31613	19980.66667	22273.66667	21065.33333
Ryk	37316	36415	21397.5	18834.5
Ryr1	14869	18001.66667	16100.66667	17342.66667
Ryr2	22262.6	19276.2	23567.2	16462
Ryr3	15326.85714	18128.71429	9887.857143	10539.28571
S100a1	12991	16773	15996	15835
S100a10	19710	16712	19824	19517
S100a11	10721	5329	4669	2189
S100a13	3334	6164	8891	2844
S100a14	24296.5	24396	26821.5	31343.5
S100a16	19175	13402	21324	25898
S100a3	34948	36732	877	1191
S100a4	34251.5	14060	15009.5	9804
S100a5	21589	24406	38900	41057
S100a6	1815	30404	3028	30149
S100a8	7482	24508	7802	23117
S100a9	40215	3971	10853	13600
S100b	33500	254	34587	35542
S100g	31524	25705	26982	26021
S100pbp	17563	17267.33333	19161	19046
S1pr1	35060	29745	41096	39794
S1pr2	28324	17974	25559	18669.5
S1pr3	21735	8493	12732	13917.5
S1pr4	14591	16382	14535	16602
S1pr5	32777	19097	39517	31607
Saa1	9902.333333	16020	8980	23426.33333
Saa3	16210	28595	25635	31498
Saa4	12177.5	10092	6475	11059.5
Saal1	15169.5	21292.5	35782.5	18795.5
Sac3d1	8281	12053	8506	9195
Sacm1l	18136.66667	22366	21796	24505.33333
Sacs	18131.25	13338.75	12818.25	13830.75

Sae1	4608	5739.5	3207.5	4826
Safb	14969	14695	14685	13432
Safb2	21317	18342	15192.5	17374
Sag	12579	5729	12191	16131
Sall1	923	915	5663	4919
Sall2	40637	7843	89	8026
Sall3	14290	18943	17590.33333	27152.33333
Sall4	17579	17413	21243.5	20111.5
Samd10	11919	14008	14381	15055
Samd11	24525	25859	34481	25803
Samd12	25904	40636	35005	1881
Samd13	6264	8036	19834	24031
Samd14	37460	40985	2780	5473
Samd3	32129	33414	435	34477
Samd4	9158	27146	12552.5	12342
Samd5	12107	9473	39308	11724
Samd7	20733	24484.5	27127.5	25762.5
Samd8	18089	15819.5	14753	17045.5
Samd9l	36392	22578	16291.5	1175.5
Samhd1	13357.5	8888	13078	12424.5
Samm50	14830.5	15443	14796.5	15656.5
Samsn1	25148	36335	23220	30537
Samt3	27657	29920	32228	373
Samt4	31753	33539	34942	34202
Sap130	19302	12050	14663	13758.5
Sap18	13061.75	12048.5	11456.25	11038.75
Sap30	33167	5211	2262	6806
Sap30bp	31150.5	24027.5	24187.5	24222
Sap30l	15218	14511	17534	15786
Sapcd1	8943.5	13791	16834.5	9416.5
Sapcd2	20317	24599	22288	24721
Sar1a	15162.5	20020.5	15236	18386
Sar1b	38612	3645	37924	39812
Saraf	22051	16845	17093	15841
Sardh	13897	2887	15335	7127
Sarm1	7207	9999	3753	18656
Sarnp	17611	25216.5	23613.5	24393.5
Sars	7577	7699	10771	11485
Sars2	1103	1574	40572	39239

Sart1	19592	17575	20365	19664.5
Sart3	6355	4636	9010	8181
Sash1	8967	13098.66667	16788.66667	18496
Sash3	2727	29930	34083	20421
Sass6	25214.66667	22346	16482	19571
Sat1	26808.5	17991.5	26398.5	15594.5
Sat2	16223	17499	18827	15351
Satb1	24121.5	19236	28597	22624
Satb2	24388.33333	22576	29634.33333	24782.33333
Satl1	34541	30191	3171	3370
Sav1	19773.66667	20385.33333	16330	24284.66667
Saxo1	23860	22950	29399	18861
Saxo2	29660.66667	28953	23159.66667	20665.33333
Saysd1	25739	30862.5	28064	27277.5
Sbds	20587	3830	21440.5	21756
Sbf1	35525	34595	27451	29191
Sbf2	21010.16667	14604	20357	17816
Sbk1	20062	18664	19810	19309
Sbno1	22795.4	18949	14783.4	18238.6
Sbno2	21493.5	27488	26049	29440
Sbp	17593.5	19281.5	23472.5	22302.5
Sbsn	23243	21169.5	25306	24156.5
Sbspon	23917	31427	2278	36965
Sc5d	9117	8591.5	5770.5	2878.5
Scaf1	19079	20725	21873	20845
Scaf11	23082.5	17903.5	15422	12905.5
Scaf4	13260	22716.5	29150	9340
Scaf8	28760.75	26197.25	28218.5	31063.5
Scai	23525.8	26109.2	22359.8	19875.4
Scamp1	16496	16338	27294.66667	4342
Scamp2	21833.66667	13919.66667	13043.33333	12542.33333
Scamp3	3641	998	41035	39906
Scamp4	30448.66667	29110.66667	28009	28238.33333
Scamp5	29199	21658	12363.5	26962
Scand1	24905	35075	30231	32942
Scap	16086	14247.5	15903.5	14563.5
SCAPER	13223.33333	19164	14875.66667	12696.33333
Scara3	10372	11248	19548	20816
Scara5	19077.5	20031	26518	25032.5

Scarb1	27815	20383	19075	18549
Scarb2	6317.5	9858	7169.5	11344
Scarf1	17847	35354	24145	37159
Scarf2	37427	34970	1548	3167
Sccpdh	22855	26932.5	26317.5	27855.5
Scd1	34687	882	26384	36595
Scd2	8535	10305	40694	4798
Scd3	4859	15301	13466	19217
Scd4	28782	27488	24380	25180.5
Scel	9780	17460	15524	10891
Scfd1	21714.75	18440.25	27409.5	28774.25
Scfd2	17298	14216	15276.5	14995.5
Scg2	29757	23095	40269	32982
Scg3	21959	20613	23324	19835
Scg5	25329	26870	27466	26383
Scgb1a1	3060	35463	23614	20260
Scgb1c1	31945	22343	33996	27384
Scgb3a1	9753	8457	13191	16241
Scgb3a2	9052	9651	21593	21449
Scgn	32743	34547	36213	34527
Schip1	18241	26428	24925	36363
Scin	9284	14139	21040	11053
Sclt1	24778.33333	24043.66667	22589	21048.33333
Scly	17969.5	37894.5	32324	33854
Scmh1	19371.66667	20247.66667	6859.666667	27336.66667
Scml2	14781	10633.66667	10778	11550.33333
Scml4	20113	21479	13577.5	25629.5
Scn10a	14825.33333	23794	14888.66667	16808.66667
Scn11a	27620	27592	29962	38849
Scn1a	20797.33333	31095.66667	33753	30649.33333
Scn1b	9091	17522.5	14354.5	17172.5
Scn2a1	33941.5	28509	30414.5	35183
Scn2b	19303	23123	5661	40819
Scn3a	30254	33356.66667	26233	29480
Scn3b	30381	31371.66667	33347.66667	18998
Scn4a	15559	18488	22670	22762
Scn5a	34420	37468	18889	2190
Scn7a	31052	33110	34329	32461
Scn8a	19070.5	8713	23462	8809

Scn9a	28821.5	30349	31987	34727
Scnm1	11432	17504	17463	20399
Scnn1a	34722.5	36023	37833.5	34544
Scnn1b	3285	38885	6636	13746
Scnn1g	3461	7640	8363	16218
Sco1	17910.5	20176	18570	20354.5
Scoc	22883	28828	21827.5	19059
Scp2	20300	22874.5	23839.5	20620
Scp2d1	36863.5	18612	3627	4547
Scpep1	10473.5	27125	10849.5	9803.5
Scrg1	11752	11034	16280	10479
Scrib	3161	37542.5	18003.5	35533.5
Scrn1	22573	16865.5	24924.5	29236.5
Scrn2	34512	31079	22761	23012
Scrn3	9495	21021	27909	24610
Scrt1	22004	27385	29161	30138.33333
Scrt2	17871	20004	22660	23464
Sct	26559	35619	2026	3048
Sctr	32499	33886	35832	33916
Scube1	17521.66667	19618.33333	11741.33333	12460.66667
Scube2	20301	24064	38545	34574
Scube3	39741	12994	6730	39630
Scx	22859	25179	28591	33469
Scyl1	7631	6411	9055	8951
Scyl2	12449	22985	24360	22178.66667
Scyl3	5363.5	4238.5	2607	1112
Sdad1	23425	27697.5	25389.5	29229.5
Sdc1	13144	21592	18263	21378
Sdc2	16483.5	18323.5	19823	24990.5
Sdc3	11571	18802.66667	11364.33333	20261
Sdc4	14252	29412.5	18837.5	29394
Sdcbp	18138.5	9547.5	19157.5	15843.5
Sdcbp2	6405	6322	4380	10511
Sdccag3	23911.5	23092	24230.5	24042.5
Sdccag8	29269.75	27674.5	24841	23318.5
Sde2	26898.5	23144	20312	20668.5
Sdf2	14434	15087	15513	15911
Sdf2l1	5723	13087	13512	13305
Sdf4	9541.333333	19994.66667	20752.66667	16731.33333

Sdha	33147.66667	30924.66667	26849.66667	27359.66667
Sdhaf1	21270	33098	21133	25294
Sdhaf2	14686.5	19556.5	19860.5	21816
Sdhaf3	30256	10997	12475.66667	14400
Sdhaf4	34814	37863	38309	32103
Sdhb	17160	20917	17824	19522
Sdhc	15095	16010.5	15139.5	13447.5
Sdhd	12738	17568	14780	16711
Sdk1	33047	34645	20240	34614
Sdk2	4320	11506	13680.5	7090
Sdpr	7892	4580	13554	15641
Sdr16c5	25430	26591	28925	27654
Sdr16c6	34631	34897	37398	36033
Sdr39u1	35614	3312	36464	760
Sdr42e1	17549	16293	19468	17336
Sdr9c7	22753.66667	20702.66667	14963	14867.33333
Sds	19430	2089	36316	8145
Sdsl	39979	38238	11649	11522
Sebox	12021	14456	22302	22701
Sec1	10244	25832	35743.5	20414.5
Sec11a	22333.66667	23770.66667	23853.33333	22080
Sec11c	36473	1416	929	2031
Sec13	22540.5	31179.5	22986.5	29295
Sec14l1	16506.66667	21740.33333	14817	17127
Sec14l2	23358	25270.5	10619	10077
Sec14l3	21817.5	22215.5	17462	17696.5
Sec14l4	34308	22211	33388	8462
Sec16a	28516.5	28653.5	27694	28495
Sec16b	12541.5	14090.5	22745.5	23148
Sec22a	18196	11823.16667	13373.33333	18900
Sec22b	18250.5	5834	18475.5	6441
Sec22c	19435	30697.33333	25742.33333	26857.66667
Sec23a	31086.5	15180.5	12820.5	15478
Sec23b	27664	24804.5	20812	22096.5
Sec23ip	32067.66667	20305.33333	31969.33333	22309.66667
Sec24a	15842.33333	22097	8706.666667	8549.666667
Sec24b	16663	10650.33333	26757.33333	26275.33333
Sec24c	3945	40132	37600	37114
Sec24d	14099	15606	14640	16838

Sec31a	36087.5	16871.5	29906	29986.5
Sec31b	20334	9107	22698	19821
Sec61a1	17056.66667	15120.66667	13867.66667	15015
Sec61a2	13551	15250	10191	13512
Sec61b	20033	27392	31542	29786
Sec61g	21838	23727	24258	26737
Sec62	24223.5	25488	26496	27480
Sec63	28169.25	21763.25	25530.5	27680.75
Secisbp2	6237	21690	19655	17968
Secisbp2l	25670	13395.33333	13282	25376
Sectm1a	25001	25672	30920	32061
Sectm1b	26565	27526	27137	26327
Seh1l	18068.33333	21008	17785.33333	8488.333333
Sel1l	17177.5	16871	16643.5	17438
Sel1l2	31794	36071	37284	33571
Sel1l3	33538.66667	28086.33333	26659.33333	20772.33333
Sele	30737	32397	33819	32551
Selenbp1	27291	19316	21000	13411
Selenbp2	25823	18139	19281	11483
Selk	34132	39078	40209	38989
Sell	8631	34060	5002	30349
Selm	15022	15266	17124.5	11872.5
Selo	19988	18617	15456	15608
Selp	14838.5	24516.5	19456.5	10832
Selplg	11703	19876.5	25334	17108
Selt	13903.33333	27597.33333	23162.66667	25097.33333
Sema3a	24456.25	25571.75	18132.75	20859.25
Sema3b	39773	28459	3450	8552
Sema3c	17216	16657.5	26243	20730
Sema3d	343	32945	32864	31415
Sema3e	23756.5	26735.5	29569	29150.25
Sema3f	17302	18724	22696.5	21197
Sema3g	4801	2037	7807	3561
Sema4a	33174	3315	21484	38593
Sema4b	3860	8756	156	4854
Sema4c	20621	20020	21970.5	22003.5
Sema4d	9511	13360.5	19314.5	26432
Sema4f	24414	36682	26860	27334
Sema4g	1131	37954	37515	2603

Sema5a	32739.5	33089	22906.5	22197.5
Sema5b	13675	19627.33333	12805	12284.66667
Sema6a	17750.4	24524	19584.4	12470
Sema6b	17098	14347	19400	16058
Sema6c	8732.5	23300.5	20527	20010
Sema6d	29344.66667	29263	35348.66667	34560.66667
Sema7a	15781	16107	24273	31101
Senp1	19776	9191	21023	20907.5
Senp2	16754	28661	27021.66667	27510
Senp3	19884	21438	22278	22425.5
Senp5	11742	10763	9352.5	10843.5
Senp6	16737.5	22040	19919.16667	11606.16667
Senp7	23253.6	22716.8	21001	18285.8
Senp8	9549	8065.333333	10607.33333	9138
Sep-02	28175	29586	32592	30940
Sep-09	29839	31321	33460	37816
Sep15	22039	22094	21112	23048
Sephs1	19120.5	18602.5	28558.5	31989
Sephs2	26895	30709	23121	27466
Sepn1	15821	11039	14210	9874
Sepp1	4763	9208	2771	12391
Sepsecs	19815.5	25725	23977.5	29041
Sept1	17057	20666	22401	30719
Sept10	12364.5	16538.5	18766.75	21424.5
Sept11	26492	32396	26094	25630
Sept12	17933	31952.5	24553	23211
Sept14	34231	27860	31475	37143
Sept2	26922.5	31482	31032	29687.5
Sept3	16017	39914.5	37901.5	19360
Sept4	2644	37467	28456	33573
Sept5	2736.5	7688.5	6468.5	12913.5
Sept6	15925.25	24070.25	19422.75	20279
Sept7	39843	3166	4427	5832
Sept8	15377	15231	19450	19303
Sept9	13432	7427	12707	8450
Sepw1	28361	26534	26177	26482
Serac1	15566	11413	12096	10338
Serbp1	8814.666667	17542	15492.66667	18674.66667
Serf1	14392	27465	16414.5	30133

Serf2	34447	38086	834	40513
Sergef	9936	8477	5976	4621
Serhl	14808.5	16072.5	15201	17617.5
Serinc1	22382	21542	23959	24533
Serinc2	30934	13301	3373	26160
Serinc3	30654	24635.66667	25474.33333	21679
Serinc4	19159	15433	17046	14405
Serinc5	26935.5	21086	24949.5	21216
Serp1	9531	9497	8655	10119.5
Serp2	22701	34686	18302	22181
Serpina10	14434.5	16724	18676.5	18525
Serpina11	23859	30180	27495	26730
Serpina12	40687	36723	2998	8895
Serpina1a	7339	10461	15074	14586
Serpina1b	28140	29840	37076	28727
Serpina1e	16291	19353	25250	22923
Serpina1f	31873	38718	34131	37756
Serpina3a	12449	13005	20600	15983
Serpina3b	16196	13688	22286	24848
Serpina3c	35218	39116	40322	36151
Serpina3f	2728.5	20917	27087	12698
Serpina3g	10711	24167	14088	25108
Serpina3j	35251	52	2911	3181
Serpina3k	20640	13610	19128.5	36344.5
Serpina3m	22870	25657	27797	28831
Serpina3n	30250	31825	33490	34264
Serpina4-ps1	20797	20059	26489	26177.5
Serpina5	29174	30759	32129.5	30812
Serpina6	11568	10156	13072	10010
Serpina7	33251	38476	37658	5926
Serpina9	36321	37257	39771	38740
Serpinb10	28698.5	23220.5	2288	12550
Serpinb11	14742	11875	16741	19291
Serpinb12	22903.5	27701	17646.5	25319
Serpinb13	27755	36952	40557	12278
Serpinb1a	19982	1728	38962	14047
Serpinb1b	33361.5	8344.5	1935.5	15694
Serpinb1c	16889.5	29645.5	12852.5	14389
Serpinb2	23919	37331	28902	13914

Serpinb3a	19860.5	32621.5	33114.5	35791.5
Serpinb3b	38568	323	8580	6919
Serpinb3c	14360.5	15337.5	20322.5	18666
Serpinb3d	25349	26223	33292	27478
Serpinb5	779	33122	587	7528
Serpinb6a	14745.33333	22330	23744	25858.66667
Serpinb6b	24504	21179	21110	22681
Serpinb6c	24978	20317	36773	32197
Serpinb7	3201	6971	10705	10481
Serpinb8	13857	18330	16533.5	22406
Serpinb9	15213	28122	12876	37979
Serpinb9b	32329	34124	36595	2522
Serpinb9c	26728	28290	32694	29169
Serpinb9d	36347	4678	10121	14523
Serpinb9e	40242	1742	9184	9463
Serpinb9g	37887.5	17444.5	1624	18453.5
Serpinc1	29749	11049	12553	12288
Serpind1	32123	33733	38347	37634
Serpine1	26371	21575	18012	29715
Serpine2	29043	23453	32018	30578.5
Serpine3	7344	13856	12948	16613
Serpinf1	20051	39727.5	8630	14210.5
Serpinf2	34048	36232	38893	36804
Serping1	13709	13970	17859	22336
Serpinh1	4255	11188	16697	24472
Serpini1	22914	28546	32928	39833
Serpini2	6692	8534	13350	14751
Sertad1	23529	17320.5	26857.5	24947.5
Sertad2	23737	16705.5	16991.5	14544
Sertad3	15894	12615	18839	14126
Sertad4	5051	9551	11907	21512
Sertm1	2798	8722	19173	11214
Sesn1	14388.33333	17239.66667	12993.66667	24938
Sesn2	37114	33579	28687	30202
Sesn3	14000.66667	12986.33333	25215.33333	24516
Sestd1	19854	20010	20215	22389
Set	20056.5	13177.5	13556.5	13949.75
Setbp1	23160.25	24298.25	27634.75	22162.75
Setd1a	13441.66667	9767.333333	10988.33333	23731

Setd1b	26104	20990	19977.5	12900
Setd2	6502	22816	23285	23204.5
Setd3	13524	26360.66667	14789	15637.66667
Setd4	24938	23287	25060	23150
Setd5	15385	19942.71429	19344.85714	25729.28571
Setd6	7572	28886.5	8407	28893.5
Setd7	19124.75	24750.5	18523	24042.5
Setd8	36347.66667	4180.333333	29267.33333	28639
Setdb1	22154.33333	22966.33333	24178.66667	24628
Setdb2	37787	1067	5086	7470
Setmar	40723	40707	37417	37109
Setx	23166.66667	20142.33333	20417.66667	18314
Sez6	31461.5	32675	16431.5	19887
Sez6l	18980.5	18204.5	21311.5	21882.5
Sez6l2	22150	22506	25562	22795
Sf1	17748.75	17828.5	19851.25	19926
Sf3a1	12204	8825.5	11775.5	29058
Sf3a2	9300	11047	10874	10685
Sf3a3	9187	9517	10090	10274
Sf3b1	22994	19772.5	19982.5	22593
Sf3b2	17080	13373	15907	13103
Sf3b3	12562.33333	11130	11432.66667	11373.33333
Sf3b4	33605	30352	28764	26599
Sf3b5	27533.5	30220.5	37624	38988.5
Sf3b6	14282.5	9368	11413.5	16307
Sfi1	1880	1464	1607.5	21233.5
Sfmbt1	18699.66667	15537.66667	17442.66667	16138.33333
Sfmbt2	30202.33333	28454	8241.333333	9278.666667
Sfn	10310.33333	15985.66667	15199.33333	18733
Sfpq	27684	21131	23269.33333	21971
Sfr1	39656	39914	43	1203
Sfrp1	30762	16414.5	17427	20812.5
Sfrp2	7041	14307	20306	21528
Sfrp4	16918	21678	22956	21089
Sfrp5	10474	12917	13130	13365
Sfswap	20364.8	25330.8	31777.8	32580.6
Sft2d1	16857	17251	15117	15151
Sft2d2	24755	22536.5	24013.5	21477
Sft2d3	15838.8	12592.8	33998.4	32851

Sftpa1	16473	18970	26399	26352.5
Sftpb	14965	17604	20906	23545
Sftpc	846	37018	1345	5901
Sftpd	35935	29810	12953	6109
Sfxn1	20821.33333	24399	22501.33333	26651.66667
Sfxn2	12427.5	28958	23404	21258.5
Sfxn3	37349	27731	40367	34805
Sfxn4	12980	13225.5	16012.5	14784.5
Sfxn5	17976.5	18629.5	16084	18505
Sgca	5427	12091	9823	4352
Sgcb	17637.33333	9275.666667	17469.66667	14238.66667
Sgcd	19631	21856	14386.5	16075.5
Sgce	12665	15190	26330	8997.5
Sgcg	19468	25162	21438	23059
Sgcz	7405	10595	14582	14661
Sgip1	22760.8	22888.4	28756.2	22419.2
Sgk1	15436	19979	18776.5	5347
Sgk2	20568	13892	20889	22306
Sgk3	12994.66667	24063.66667	30057	27539.33333
Sgms1	28039.5	21919.5	20102.5	23643
Sgms2	25352.5	12956	19878	12378
Sgol1	10446	9497	13520	13235
Sgol2a	25528.5	18467.5	15970.5	19851
Sgpl1	20294.5	23782	21130	28671.5
Sgpp1	23137	20816.5	22971	22169.5
Sgpp2	27858	29327.5	31646.5	17219
Sgsh	24154	14517	13536	26571.5
Sgsm1	7962	11093.5	15635	14500.5
Sgsm2	20194	37573.5	18078.5	14229
Sgsm3	23670	24420	20653	21828
Sgta	38277	36481.5	20534	21962
Sgtb	40233	37894	33278	33793
Sh2b1	27895	22248	26966	24930
Sh2b2	26903	29458	26168	31125
Sh2b3	15028	29696.5	28153	21421.5
Sh2d1a	28487.5	29675.5	32804.5	35030.5
Sh2d1b1	17279.5	22447	21103.5	28192.5
Sh2d2a	29579	25354	38557	30247
Sh2d3c	13120	9149	11705	5766

Sh2d4a	33026	34914	36059	36737
Sh2d4b	32574	24983	37119	35529
Sh2d5	12833.5	9407	27845.5	11320.5
Sh2d6	14835	17010	19168	19382
Sh2d7	19643	21231	34638	34277
Sh3bgr	15612	22012	22748.5	28938
Sh3bgrl	18012.75	20279.5	13535	14815
Sh3bgrl2	26661.5	7050.5	14404.5	10668.5
Sh3bgrl3	6452	4498	7491	6485
Sh3bp1	29328	29810	12148.5	10390.5
Sh3bp2	27894	19007	17964	16520
Sh3bp4	10821	23352	15029	24705
Sh3bp5	21781.75	24949.75	21192.75	21612.25
Sh3bp5l	9102	3063	1752	38138
Sh3d19	15868	15590	19651.66667	17148.66667
Sh3d21	11629	13059	13086	13943
Sh3d2c-ps1	34189	31657	37750	35988
Sh3gl1	27282	24141	25504	24642
Sh3gl2	24737.33333	23707	14264.66667	24934.33333
Sh3glb1	20621.25	18646.25	24326	16492.25
Sh3glb2	18728.5	18039.5	16871	15253
Sh3kbp1	15426	14076	16147.33333	13222.33333
Sh3pxd2a	21828.5	5987.5	9601.5	10230.5
Sh3pxd2b	20509	13668.5	18000	16346.5
Sh3rf1	23520.75	27032.75	23840	12413.75
Sh3rf2	31349	35312	31988	30933
Sh3rf3	30154	28222	33990	33182
Sh3tc1	6204	13016	8287	18843
Sh3tc2	21202	22705	38529	39332
Sh3yl1	23301.5	24258.5	25337.5	22786
Shank1	8178	5148	31965	36546
Shank2	40342	5073	18587	13307
Shank3	16527	17732	37047	32136
Sharpin	9292	7134	6494	7234
Shb	3984	4473	7809	1341
Shbg	6843	14081	1027	6366
Shc1	3749	9167	5536	4474
Shc2	6978.5	8746	13200	11011.5
Shc3	21417	23947	28536	34758

Shc4	4051	21749.33333	11681.33333	13634.66667
Shcbp1	18970	18608	19372	17794.5
Shcbp1l	1209	25397	31022	12046
Shd	36177	36483	3366	3960
She	5320	1095	25743	19093
Shf	7362	8296	13298	11772
Shfm1	1706	5394	4854	1571
Shh	25197.5	27901	32423.5	32770.5
Shisa2	29846	30945	30625	29280
Shisa3	28679	35363	24478	293
Shisa4	2213	4705	10201	11166
Shisa5	24545.33333	23243.33333	9680	11700.33333
Shisa7	40813	3414	7371	8091
Shisa8	21347	23513	29069	33120
Shisa9	16174.5	24643.5	21248	28261.5
Shkbp1	1341	38630	38536	35805
Shmt1	14092.5	17157	15033.5	14754.5
Shmt2	37273	37702	37093	36177
Shoc2	27108	10794	10941	31088
Shox2	34071	40593	28594	30177
Shpk	15036	15645	34498.5	32545.5
Shprh	13596	10054.16667	17708.16667	14542.33333
Shq1	28644.5	25652.5	25277	29258.75
Shroom1	5976	13631	14817	20612
Shroom3	24439	14889.66667	17486.66667	18724.66667
Shroom4	9526	11428	10803	10725
Shtn1	24473	25960.5	27864	27232.5
Siae	21077.33333	16838.33333	16812.33333	15310.66667
Siah1a	12897	12978	11772	15218
Siah1b	38227	18261	38662	18278
Siah2	3790	6078	2149	1009
Siah3	33765	35727	36839	37915
Sidt1	14024	14039	22479	22307
Sidt2	14939.5	12934	15249.5	14407.5
Sigirr	4804	5783	6970	6757
Siglec1	19319.5	27007	30436.5	26532
Siglec5	31661	8774	15756	28963
Siglece	36288	4929	36056	6955
Siglecg	342	4753	2214	4582

Siglech	23908	25062	26697	26977
Sigmar1	11429	9404	4750	3765
Sik1	31625	24414	36024	24919
Sik2	21307	13248	15046.5	19989
Sik3	16365.5	14987.75	15568.25	15898
Sike1	22911.33333	8681.666667	9914.333333	10885.66667
Sil1	12707	12300	13613	15204
Sim1	25128	26443	28020	26971
Sim2	27510	38486	36717	28950
Simc1	9299	7270	7604	8289
Sin3a	12158	13265	13672	14081
Sin3b	22997.66667	20318.33333	20092	24933.66667
Sipa1	17120.5	17072.5	14452	11563
Sipa1l1	16883.25	22074.25	17072.75	18295.75
Sipa1l2	19269	12207	17058	10494
Sipa1l3	27178	17144.5	16088.5	17382
Sirpa	13526	13864	12287	13163.33333
Sirpb1a	23325.33333	25504	22025.66667	10471.66667
Sirt1	16100	13201	12723	12933.5
Sirt2	26337.66667	23862	23410.66667	21564.66667
Sirt3	22995	22165	21101	20464
Sirt4	11479	12006	12021	12063
Sirt5	40438	720	202	39777
Sirt6	31968	38579	32542	36379
Sirt7	14114	8804	11681	9511
Sis	40129	986	4760	4030
Sit1	6465	10670	10012	11826
Siva1	27848	32264	26546	26215
Six1	19872	14626	12139.66667	23507.33333
Six2	14437	15362	18522	19083
Six3	13302	12583.5	18334.5	18634
Six3os1	3734	4380	374	11191
Six4	25065.5	28534.5	32868.5	30045
Six5	33492	38678	38703	3515
Six6	32780	34608	36115	34500
Ska1	26543	25002.5	25913.5	32998.5
Ska2	30024	14126	20469	9385.666667
Ska3	22987	17624	27643	30470
Skap1	10307.66667	25201.33333	20896.33333	22110.66667

Skap2	29506.5	27869.5	29012	29655.5
Ski	19609.33333	12159.66667	14258	22208
Skida1	28058.66667	29608.66667	35797.66667	24607.33333
Skil	2781	11120.5	6052.5	17235
Skint10	32504	33745	35860	33937
Skint11	24050	25098	27327	26180
Skint2	35642	33984	17256	20774.5
Skint3	16340	13327	7294	32337
Skint4	4700	7575	19277	17145
Skint7	28112	29892	35658	24160
Skint9	28692	38315	31630	33187
Skiv2l	18219	17733	16976	15237
Skiv2l2	23413.5	35955	35739	25942
Skor1	27023	29970	37765	38733
Skp1a	36805	37400	31713	37293
Skp2	24968	18377.5	20527.5	17045.5
Sla	19700.5	26970	19171	23193.5
Sla2	7136.5	8648	13891.5	17757
Slain1	2650.5	20527	33354	20440.5
Slain2	23246.75	23781.75	22814	24311.75
Slamf1	16857	21107.5	2270	12116.5
Slamf6	6218.333333	18527.33333	10615.33333	17800.66667
Slamf7	18024.5	38629.5	29886	20433
Slamf8	13661	18527	14570	20654
Slamf9	16884	7382	18791	6084
Slbp	29860	29820	27336	29778
Slc10a1	10532	6867	12880	12758
Slc10a2	31353.5	34012	29926	31389
Slc10a3	18063	17870	16092	15916
Slc10a4	35004	35052	18177	21801
Slc10a6	12465	19592	20868	32293
Slc10a7	37808.5	36321	20750.5	38276
Slc11a1	2895	1368	40149	4295
Slc11a2	10321	17998	12068	18174.66667
Slc12a1	21805.5	22336	22447.5	23055.5
Slc12a2	18749	20089.5	24280.5	28067.5
Slc12a3	5595	14188	15210	23662
Slc12a4	31927	32585	26987	34740
Slc12a5	21895	27725	35822	38276

Slc12a6	24353	10716.33333	20174	19764.66667
Slc12a7	18758	6455	15301	3716
Slc12a8	6460	40690	10285	10328
Slc12a9	37264	34429	40429	30832
Slc13a1	32688	33820.5	34373	37393
Slc13a2	40270	37848	855	9208
Slc13a3	2398	5771	40265	4667
Slc13a4	29570	32676	40448	40698
Slc13a5	40356	2710	1821	3956
Slc14a1	11679	18956	23014.5	20531
Slc14a2	22199	21747.75	17181	25822
Slc15a1	26646	28415	28382	27285
Slc15a2	9417.5	5818	18512.5	13713.5
Slc15a3	18376	14492	18289	18479
Slc15a4	8825	2207	2406	1010
Slc15a5	26764	28324	29372	28145
Slc16a1	9827.333333	12043.66667	11669	13008.66667
Slc16a10	24720.75	22384	26407.75	22554.25
Slc16a11	22786	21932	34902	34099
Slc16a12	29829	35638	39700	5798
Slc16a13	18503	18361	16342	16737
Slc16a14	23767	30760	36232	33276
Slc16a2	8775	12945	13150	15846
Slc16a3	1118	8532	7747	18589
Slc16a4	29678.5	31240.5	34985	31471.5
Slc16a5	24587	24430	4329	4525
Slc16a6	20215	30108.5	19489	19588.5
Slc16a7	17267	12497	16671	9957
Slc16a8	108	2329	5341	5488
Slc16a9	3152	18013	10881	29916
Slc17a1	32683	34368	36001	39702
Slc17a2	7457	8885	11404	9892
Slc17a3	29041	30900	32266	15885.5
Slc17a4	13323	10566	21129	22174
Slc17a5	20178	13989	19656	14725
Slc17a6	9930.5	15046	17782.5	35662
Slc17a7	25384	26480	32009	39752
Slc17a8	6649	27318.5	15211.5	11449.5
Slc17a9	7985	8178.5	7854.5	7468.5

Slc18a1	12187.5	7952	13103.5	6901
Slc18a2	26049.66667	27378.66667	26378	18999.33333
Slc18a3	10307	9307	7369	12869
Slc18b1	10216	5098	4757	36841
Slc19a1	7284.5	7037.5	8292.5	25984.5
Slc19a2	8415	7093	8857	8778
Slc19a3	25383	27667	30314.5	27921
Slc1a1	21134.5	17191.25	21027.5	27154.5
Slc1a2	20725.4	25148	27150	25874.6
Slc1a3	28102.66667	32615.66667	30561	29206
Slc1a4	10535	15482	13574.5	18734
Slc1a5	28756	36132	26659	37268
Slc1a6	37292	39391	3318	3793
Slc1a7	12157	14372	10855	15324
Slc20a1	27069	30009	26052.5	34601.5
Slc20a2	18818	21136	20879	21913.5
Slc22a1	35554	39348	36987	39691
Slc22a12	10423	12612	14271	14779
Slc22a13	31169	37264	366	38373
Slc22a14	6969	8551	5669	7380
Slc22a15	23280	20928.33333	23362.66667	20933.33333
Slc22a16	15865	12339	19183	15781
Slc22a17	23347	25263.5	29343	28080
Slc22a18	9879	27625	29993	24263.5
Slc22a19	33553	23088	1621	38635
Slc22a2	9126	700	14344	12086
Slc22a20	21428	21903	23630.5	30128
Slc22a21	16115	13127	14982	13448
Slc22a22	32962	4000	1275	36690
Slc22a23	28537.5	20145	21878.75	17771.5
Slc22a26	12926.5	13337.5	21968.5	21308
Slc22a29	32633	34138	35718	33830
Slc22a3	32195	33405	36015	34020
Slc22a30	24169.66667	14434.66667	18948	17432
Slc22a4	23116	39838	30688	4134
Slc22a5	22348	18899.5	20813	20245
Slc22a6	39058	29	5306	5460
Slc22a7	12495	14979	10387	20265
Slc22a8	23122.33333	23203.66667	23096.33333	24618.33333

Slc23a1	18389.5	20411.5	17094	22847.5
Slc23a2	14454	10184	15919	12761
Slc23a3	1578	1017	5608	5038
Slc24a1	29838	31814	31714	30340
Slc24a2	23533	26674	25842.33333	13450.33333
Slc24a3	23946	22204	26464	25737.5
Slc24a4	31556.5	32808.5	16956.5	19681.5
Slc24a5	38969	9613	5243	11591
Slc25a1	32399	29114	21154	24672
Slc25a10	29782	36736	24008	30934
Slc25a11	17677	20921	17903	19649
Slc25a12	23623.66667	25829.66667	31635.66667	35352
Slc25a13	15727	9328	13555	7258
Slc25a14	12260.66667	15726	19452.33333	19958.66667
Slc25a15	2010	5571	278	3586
Slc25a16	36138	35470	25851	30539
Slc25a17	28859	34922.5	32652.5	36564.5
Slc25a18	38665	4541	4615	7825
Slc25a19	22658	23157	23553	24149
Slc25a2	39306	36471	33353	37103
Slc25a20	9169	11024	12000	11513
Slc25a21	28940.5	28620	17654.5	40191.5
Slc25a22	35185	38680	29484	32207
Slc25a23	33753	38769	39133	40086
Slc25a24	27709	29633	31054.5	29801.5
Slc25a25	21166	10000.66667	17419.33333	19481.66667
Slc25a26	19169.33333	18949.33333	19836.66667	19239.66667
Slc25a27	28814.66667	19774.33333	26786.33333	23318
Slc25a28	33607	28188	33329	31800
Slc25a29	20305	19120.5	10737.5	16188
Slc25a3	21209	14302	16422.75	25618.5
Slc25a30	26098.66667	15005	14784.33333	14772
Slc25a31	37764	1914	7052	7704
Slc25a32	13421	17229	14644	17553
Slc25a33	13445.5	12370	17395.5	13355.5
Slc25a34	111	58	10847	11495
Slc25a35	15911.5	11829.5	24089	12345
Slc25a36	15416.5	16799	17357.5	17663
Slc25a37	11250.33333	32169.66667	20254.33333	25937

Slc25a38	12867	12149	9954	10732
Slc25a39	27751.5	26204	19449.5	20796
Slc25a4	14019.5	16401	15293.5	14958
Slc25a40	28256.8	26273	28404.2	27510.2
Slc25a41	7441	4390	11336	13529
Slc25a42	23054.5	27921	26278.5	12382.5
Slc25a43	16903	14764.5	28234.5	29625
Slc25a44	14264	16693	16876	19805
Slc25a45	21758	19331	18531	16208
Slc25a46	25351.5	16345	26064.75	14699
Slc25a47	2745	5825	2349	4098
Slc25a48	25740	27045	28937	33761
Slc25a5	15429	15143	11412.5	13768
Slc25a51	19709.5	14644	12790	14064.5
Slc26a1	18808	19740	25136.5	25988.5
Slc26a10	575	4156	40758	16011
Slc26a11	3450	22487	37057	20235
Slc26a2	36711	40440	34737	38413
Slc26a3	24751	24539.66667	17602.66667	26878
Slc26a4	17191	17506	20887	29838
Slc26a5	12944	13619	23465	18818
Slc26a6	2169.5	20527.5	38918	38468
Slc26a7	32472.5	28233	14679	28372.5
Slc26a8	34776	4817	27943	37486
Slc26a9	14187	16501	19485	19394
Slc27a1	37405	31400	39977	31746
Slc27a2	33939.33333	30649	22606.33333	26076.33333
Slc27a3	31617	24631	29407	32134
Slc27a4	22814.5	21048.5	20905.5	17658
Slc27a5	8550	3511	6950	16527
Slc27a6	658	3352	5179	38301
Slc28a2	16714.5	25139	28551.5	24355
Slc28a3	15672.5	18077	20643	19930
Slc29a1	10935	11796	10021	9667
Slc29a2	8933	11311	11587	10687
Slc29a3	11734.33333	14925.66667	15275.33333	14485.33333
Slc29a4	25402	28483	48	39188
Slc2a1	6248	10205	9868	15438
Slc2a10	7179	1475	14296	7255

Slc2a12	39622	31789	33831	37066
Slc2a13	27034	27951	34868.5	15611.5
Slc2a2	15777.5	18473	19591	20404
Slc2a3	14914.5	9159	16529.5	30025
Slc2a4	3802	40744	10732	6817
Slc2a5	29091	16152	20100	12510
Slc2a6	23648	13835	21182	9021
Slc2a8	34811.5	34689	31838.5	28092.5
Slc2a9	8795.5	18476.75	11246.5	25266
Slc30a1	4088	22091	4003.5	4037
Slc30a10	29379.5	35902.5	23706.5	28420.5
Slc30a2	2736	8606	40803	8838
Slc30a3	31528	33179	7090	9745
Slc30a4	22829	25409	21687	27580
Slc30a5	18149.66667	15290	13859.33333	10181
Slc30a6	22233	39470	20120	36189
Slc30a7	23021.25	22089	19417.75	19172.5
Slc30a8	6803	13900	15318	18529
Slc30a9	19469	17553.33333	18011.66667	19335.66667
Slc31a1	7300.5	15080.5	7788	13426
Slc31a2	15066.5	29206	15850	29012.5
Slc32a1	6869	5570	8752	5322
Slc33a1	18524.33333	3998.333333	876.6666667	13692.66667
Slc34a1	21968.33333	20251.33333	25177.33333	23061
Slc34a2	21074.5	22294	17495.5	36245.5
Slc34a3	16490	18822	25907	30880
Slc35a1	34117	35252	25984	30333
Slc35a2	29212.5	29515.5	30902.5	30349.5
Slc35a3	14260.66667	24129.33333	20210.66667	24040
Slc35a4	12657	9529	21359	2712
Slc35a5	8649.75	16047.75	4755.25	4358.5
Slc35b1	129	2964	32122	39902
Slc35b2	8116	7443.5	7369	6462.5
Slc35b3	11706	8703.5	7373.5	6177.5
Slc35b4	17719.66667	16590	27037	26266.33333
Slc35c1	15850	16727	20811	18408.5
Slc35c2	12271	9902	9593	5433
Slc35d1	25300	30800.33333	17152	16610.33333
Slc35d2	22329	6350	7104.5	6304.5

Slc35d3	40889	2462	11061	3539
Slc35e1	18713	17479	16692	15031.5
Slc35e2	6215	22782	1138	37318.5
Slc35e3	32949	27531	28587	28549
Slc35e4	38096	38073	3861	3476
Slc35f1	26489.5	33223.5	21760.5	21425.5
Slc35f2	39557	2726	40163	7168
Slc35f3	29800.5	12297.5	16065	18788
Slc35f4	22553	26199	25042	25286.66667
Slc35f5	18506	17959	17864	19020
Slc35f6	22220	14392.33333	19375	16807.33333
Slc35g1	15280.66667	19300.33333	24169.33333	24803.33333
Slc35g3	13868	12240	15349	16413
Slc36a1	18998	9366.5	13087	9020.5
Slc36a2	32785.5	24766.5	27734	23804.5
Slc36a3	23605.33333	24262	25224	26541.66667
Slc36a4	7721	4903	20402.5	23612
Slc37a1	40878	32324	38219	25171
Slc37a2	26045	27584	18692	25674
Slc37a3	20436	13250	13723	8095
Slc37a4	14650	13834	13418	13259
Slc38a1	14744	11907.66667	14476	24424.33333
Slc38a10	13961	13177.5	10757.5	10018.5
Slc38a11	16538.66667	20002	23784.33333	22766
Slc38a2	20499.25	36627	25759.5	27668.75
Slc38a3	35561	36813	926	40480
Slc38a4	24223.5	11284	28977	37000.5
Slc38a5	29077	39922	36726	3523
Slc38a7	9927	10794	9940	8786
Slc38a8	10964	17877	21180	23164
Slc38a9	14052	15625.75	21249.5	18893.25
Slc39a1	1737.5	3790.5	4987	5423.5
Slc39a10	10610	7477	10510	9475
Slc39a11	11496	6696	9932	5271
Slc39a12	34097	22852	33095	22227
Slc39a13	5809.5	5931.5	23250.5	22191.5
Slc39a14	7917.5	16648.5	9576.5	19992.5
Slc39a2	3750	9457	12154	14504
Slc39a3	6066	4443	5832	630

Slc39a4	2411	183	12277	6245
Slc39a5	25705	35622	5727	15824
Slc39a6	22891	23132	23229	23959
Slc39a7	21553	21215	23493	28617
Slc39a8	20634	15153	20523	24544.66667
Slc39a9	9985.5	7086.5	8123	8096.5
Slc3a1	22648	3551	14057.5	17840.5
Slc3a2	35729	13348	28098.5	36525
Slc40a1	18610	36515	15173	39730
Slc41a1	24792.5	24168	8569	8277.5
Slc41a2	19746	16588	17431	17845
Slc41a3	15347	11780	14168	10542
Slc43a1	14570	9714	18127	15921
Slc43a2	14977.5	8305	7872	7395
Slc43a3	15656	21559	13728	18779
Slc44a1	22938.33333	25210	18387.66667	24689
Slc44a2	17675.5	16224	14313	15005
Slc44a3	3488	34989	35414	668
Slc44a4	31850.5	16925	21382.5	5233
Slc44a5	1393	27177	39354	29872
Slc45a1	13244	15852	16416	17330
Slc45a2	15344	18906	22365	23962
Slc45a3	5745	38051	8366	1367
Slc45a4	21687.5	10409	13612	27363.5
Slc46a1	167	39473	7310	1107
Slc46a2	35941	35072	15164	13885
Slc46a3	13122	26436	6421	21495
Slc47a1	17145	17401	12408.5	23644.5
Slc48a1	14466	17397.5	9844	17245
Slc4a1	36132	617	3866	7596
Slc4a10	29562.33333	31124.33333	33678	21892.33333
Slc4a11	35500	570	4136	1710
Slc4a1ap	12500.5	13373	11585.5	13173
Slc4a2	17747	13956	13780	14029
Slc4a3	25426.5	10210.5	8838.5	10996.5
Slc4a4	25479.66667	27426.66667	29696.66667	29605.66667
Slc4a5	34818	20168	22269	4545.5
Slc4a7	8701.666667	17698.33333	18875.66667	24689.66667
Slc4a8	16792.33333	14916.66667	19811.66667	19894

Slc4a9	12431	14385	17449	17272
Slc50a1	17215	18062	17231	16850
Slc51a	20604	436.5	37804.5	37799.5
Slc51b	15348	15267	20513	19274
Slc52a2	10998	11535	8220	10329
Slc52a3	26273.5	15983.5	29238.5	19066
Slc5a1	18779	18922.5	33511	16641
Slc5a10	15240	16313	18979	18441
Slc5a11	20781	14123	8239	7904
Slc5a12	28821.5	30266.5	31908	30487.5
Slc5a2	12288	10823	14248	8150
Slc5a3	14142	11489	8143	11548
Slc5a4a	2341	28170	4051	29875
Slc5a4b	14174.33333	17210.66667	20336.33333	20118.66667
Slc5a5	14448	19840	16929	17110
Slc5a6	39804	4096	36766	2373
Slc5a7	26710	28042	29703	29515
Slc5a8	767	533	10086	15402
Slc5a9	30689	32562	33499	15756.5
Slc6a1	34926	5977	14121	9936
Slc6a11	15049	24599.5	19890.5	19634
Slc6a12	17693	2733	16305	38429
Slc6a13	30307.5	16032.5	32507	12071
Slc6a14	19924	20489.5	25094	25190.5
Slc6a15	23461.5	24525	26289	34320.5
Slc6a16	11795	14671	15877	16891
Slc6a17	17969.66667	22173	14527.33333	12373.66667
Slc6a18	11481	13065	24442.33333	11732.66667
Slc6a19	12336	35189	8187	17317
Slc6a2	12430	11753	18913	21436
Slc6a20a	28128.5	23130	21333.5	18497
Slc6a20b	17140	18480	25322	28062
Slc6a3	23197	28345	37780	31255
Slc6a4	35686	3999	3900	5379
Slc6a5	31102	32795	34378	32901
Slc6a6	19402	18835.5	31244.5	19811.5
Slc6a7	26811.5	25546.5	33380.5	33042.5
Slc6a8	27393	18315	35218	25874
Slc6a9	21176	17990	39870	38565

Slc7a1	25564	26330.5	26476	28331.5
Slc7a10	24899	26150	40980	32258
Slc7a11	19230.8	16599.4	21275.8	20877.6
Slc7a12	31704	797	37521	5436
Slc7a13	32616.5	38057.5	34188	20435
Slc7a14	18097.33333	18821	21739	23975.66667
Slc7a15	1181.5	37303	19914.5	22169
Slc7a2	19683	36497	25146	4862
Slc7a3	38121	28271	41011	38606
Slc7a4	36607	30952	25872	30664
Slc7a5	12940	20076	17853	22955
Slc7a6	19192	21516.33333	21161.33333	23472.33333
Slc7a6os	10856	10805	13160	13861
Slc7a7	2590	40768	436	37930
Slc7a8	39408	39871	839	3654
Slc7a9	2491	3020	5892	7071
Slc8a1	16502.5	16656.7	17757.6	17914.4
Slc8a2	12396	15147	18696	18899
Slc8a3	15918	17330.33333	30944	19725.66667
Slc8b1	37808	38472	4093	1225
Slc9a1	6633.5	26618.5	23077	22997.5
Slc9a2	32921.5	24708	36696	31484
Slc9a3	27380	24731	30781.5	31909.5
Slc9a3r1	37897	36976	38296	32694
Slc9a3r2	28193.66667	32496.33333	18028.66667	33434.66667
Slc9a4	9451.5	13440.5	7327.5	10921
Slc9a6	14474.33333	10838	25373.33333	23196.66667
Slc9a7	14764	19534	11837.5	15339.5
Slc9a8	17171.5	24915.5	17961	19677.25
Slc9a9	29565	26882.75	25424.5	25325.5
Slc9b1	18816.66667	27958.66667	22560.33333	26562
Slc9b2	17684	33006	40018	286
Slc9c1	23244	24300	26731	35157
Slco1a1	30141	32087.5	33213	31544
Slco1a4	35605	37632	809	40741
Slco1a5	30423.5	29052.5	20848	21297
Slco1a6	31317	33039	34482	33168
Slco1b2	27580	36146	40965	40676
Slco1c1	4049	2352	7261	8571

Slco2a1	21336.66667	20458.33333	18567.66667	18698
Slco2b1	21583	8865	18642	60
Slco3a1	34584	3461	30277	4318
Slco4a1	2674	4521	3728	9820
Slco4c1	31643.5	33392.5	37800.5	33419
Slco5a1	18339.5	19698	33951.5	32304
Slco6b1	28491	37282	30064	28782
Slco6c1	40926	33248	34766	33003
Slco6d1	32485	36618	5544	28426
Slf1	22134.6	28244.4	21500.8	27990.6
Slfn1	19785	34629	28351	11347
Slfn10-ps	22129	19307	20172	24261
Slfn2	4842	15664	6017	18511
Slfn3	20427.5	20093	21273	8271.5
Slfn4	13810	20579	18888	35973
Slfn5	2530	8610	39728	2286
Slfn8	33705	33385	23887	38622
Slfn9	29399	29333	23632	37622
Slfnl1	7589	10376	11030	8541
Slirp	20112	25861.5	18158.5	24381.5
Slit1	15462	15486	20111	19252
Slit2	28967.5	24663.5	20660.16667	24131.83333
Slit3	26902	16352	16888.33333	19087.33333
Slitrk1	13859	40315	16135	9024
Slitrk2	38698	11179	36394	10395
Slitrk3	37335	839	41000	37426
Slitrk4	24377	25454	27756	26555
Slitrk5	24691	25929	27577	26415
Slitrk6	39986	10500	6310	14147
Slk	13044.75	10854.75	10201.25	10510.5
Slmap	28022.33333	21740.66667	22170.66667	19949
Slmo1	17854.66667	18060.33333	19183.66667	18957
Slmo2	12576	19144	14359	16750
Sln	9181	11389.5	30764.5	12964
Slpi	26002	23560	26853	22412
Sltm	20273	19541.5	19322.75	24912.25
Slu7	11844	14473	11676	11578
Slurp1	18560	19163	22059	24968
Slx1b	25824.5	4916.5	26310	6467

Slx4	25056	21955	22750	22007
Slx4ip	21765	10834	17253.33333	12813
Slxl1	16318	14280	28285.5	27950
Sly	31452	29969	38843	37172
Smad1	32249	24119	29419.5	24922.5
Smad2	17334.5	30893	28131	32068
Smad3	29472.66667	29527.33333	35533.33333	36434.33333
Smad4	13519	9811.5	10898.5	9702.5
Smad5	21269	18613.66667	15365	17759.33333
Smad6	1398	17149	5144	18521
Smad7	40840	22978	36404	37077
Smad9	21181	16806	27181.5	3226
Smagp	17806	9665	23501	14838
Smap1	13969.33333	12540.33333	12153.33333	13360.33333
Smap2	25415	16690.5	22893	17632.5
Smarca1	10081.66667	12829	16937	17261.33333
Smarca2	21897.33333	11638	15699.33333	24547.66667
Smarca4	14083.33333	8447.666667	7381.333333	17722.33333
Smarca5	14926.2	13979.4	13500.4	13757.2
Smarcad1	10416	8880	9153	29099.5
Smarcal1	19175.33333	14075	12058.66667	23736
Smarcb1	5663	3718	5896	4646
Smarcc1	23454	19613.66667	12852.33333	23224.66667
Smarcc2	29926	23933.5	21633	18941
Smarcd1	10897	7649	8963	6912
Smarcd2	31525.5	33244.5	15634.5	34203.5
Smarcd3	6911	17352	14578	25656
Smarce1	23830.8	18153.4	20386.8	26553.4
Smc1a	15812.5	13464.5	13868	11796
Smc1b	12209	16587	17225	20685
Smc2	20452.75	12514.25	19371.75	12872
Smc3	12619	11827.5	11069.5	10703.5
Smc4	20688.5	14618.5	16415	12258.5
Smc5	13357.5	13693.5	15522	15953
Smc6	30693.66667	17546.66667	14919.33333	19490.33333
Smchd1	17373.8	27283	28679.2	27236.8
Smco1	32975	34016	35838	38546
Smco2	36224	38496	4230	4243
Smco4	26138	30514	31488	34781

Smcp	8699	37390	8207	7625
Smcr8	26803.75	23953.5	23570.25	26711.5
Smdt1	38461	39013	4099	1366
Smek1	12517.5	10730.5	11628.5	12730.75
Smek2	21758.6	13722.6	8427	15205.8
Smg1	15305.5	20436.33333	20346	21489.66667
Smg5	17882.33333	16270.33333	14372	18307.33333
Smg6	23040.28571	20865.42857	21808.14286	15035.85714
Smg7	9585.333333	7810.333333	9003.666667	9532.333333
Smg8	5500	5234	10312	11008
Smg9	21969	21943	24436	29600
Smgc	26202.5	27472.5	33495.5	35467
Smim1	23155	17023.5	28402.5	29535.5
Smim10l1	11392	16696	14001	18802
Smim11	23289	24323	31749	24720
Smim12	9097	9635	11556	10798
Smim13	19902	21751	19684.5	20307.5
Smim14	27008.5	27075	14956	8863
Smim15	38976.5	37660	37793	36928
Smim18	37167	29919	137	5918
Smim19	14461	14348	11058	10658
Smim20	37428	37768	37723	31413
Smim23	33970	30549	33573	38467
Smim24	15255	12469	12228	8669
Smim3	27562	35768	24842	36077
Smim4	30383	37143	28279	25135
Smim5	36741	36561	35127	13563
Smim7	13182	12502.5	12177.5	12103.5
Smim8	10365.5	11044	9023.5	7090
Smlr1	24260	25542	27056	33281
Smn1	18336	18839	18926	22408
Smndc1	28586.5	25353.5	27279	28545
Smo	22091.33333	23226.33333	27244.33333	28658.33333
Smoc1	1668	4684	9418	18617
Smoc2	12949.5	12128.5	17791.5	19431.5
Smok3a	33965.5	22378.5	20228.5	32221.5
Smok4a	223	31035	3812	502
Smox	10436.5	9354.5	7488.5	12713
Smpd1	5741	4021	6377	5600

Smpd2	39183	1195	36792	40157
Smpd3	9163.333333	8996	12229.66667	9921.333333
Smpd4	15367	12712	14077	14607
Smpdl3a	37880	25404	28617	26140
Smpdl3b	17182	15620	18168	10652
Smpx	8952	36768	981	40066
Smr2	39760	30034	33799	34281
Smr3a	29333	30903	34009	39069
Sms	21078.66667	14843.33333	19096.33333	17009.33333
Smtn	3724	1155	3093	39124
Smtnl1	35405	39913	32190	30655
Smtnl2	12408	24522	13567	23709
Smu1	27301.33333	26582	21420.33333	26912
Smug1	17599.75	14779.75	26960	26022
Smurf1	36254.5	4498	30606	23136.5
Smurf2	14199	22655.5	12091.75	20184
Smyd1	26267.5	33639.5	31026	34873.5
Smyd2	16600.5	16131	20353.5	26799
Smyd3	15889.66667	26262	25508	24679
Smyd4	16912	17048	16880	15161
Smyd5	22809	23033	20192	20933
Snai1	24279	25341	33351	6967
Snai2	5219	14067	15899	31974
Snai3	9466	10671	15170	18701
Snap23	22743.66667	25378.66667	27740	25421
Snap25	16754.66667	8710.333333	17106.33333	24690
Snap29	15384	17746	15323	17622.66667
Snap47	18083.5	19771.5	17640	17558.5
Snap91	27679.66667	32606	31079	30601.66667
Snapc1	22592	8112	5895.5	8817
Snapc2	520	3253	1457	3007
Snapc3	12863	10595	27460.5	6772
Snapc4	25244	23555	23843	25477
Snapc5	11338	16594	17222	14284
Snapin	23497	22569	23930	23348
Snca	16209.5	20141.5	19581.5	19941.5
Sncaip	21716.75	25116	26640.75	18047.25
Sncb	13176	9177	12471	10168
Sncg	14766	11324	14442	18573

Snd1	28842	32316	22550	24393
Sned1	17936.4	16066.4	26202.6	11379
Snf8	13210.5	14286.5	13761	17461.5
Snhg11	21891	17743.6	25537.4	22634.2
Snhg3	20705	21108	22246	21003
Snhg5	14206	14764	15808	15412
Snhg7	21740	19119	20618	20057
Snhg8	15171	16472	17047	14044
Snip1	13605	14708	12682	13817
Snn	17602.5	30170.5	19039	27191.5
Snora69	33310	27912	31845	30000
Snord22	8886	8082	13761	10971
Snord33	5523	4751	4824	7030
Snord35b	36580	35715	38589	38760
Snph	14339	10117	17606	11292
Snrk	22113.5	14825	14998.5	10280.5
Snrnp200	13458.33333	21569.33333	31845.66667	23117.66667
Snrnp25	12687.5	16028	15747.5	13431.5
Snrnp27	5083	11714	8965	11504
Snrnp35	34100	39815	37845	568
Snrnp40	25879	26166	27368	27366
Snrnp48	25162.5	6640.5	7182	9266.5
Snrnp70	20383	16779	19987.5	17990
Snrpa	16621.25	18955.5	16172.25	10249.25
Snrpa1	20527	22876	19382.5	25121.5
Snrpb	7249	7170	4143	7256
Snrpb2	8498.5	8366.5	5379.5	4577
Snrpc	8001	10194	8354	10244
Snrpd1	11908	12829	29077	14140
Snrpd2	30315	36803	33301	33647
Snrpd3	18232	10220	12737	12951.66667
Snrpe	14351	16938	13874	13839
Snrpf	38730.5	2899.5	4613	2199.5
Snrpg	8829	12819	13494	11235
Snrpn	10928	10574	16368.5	18313.5
Snta1	1756	4028	7546	6501
Sntb1	3183	8609	10990	12734
Sntb2	25235.66667	24017.66667	19173	24536
Sntg1	18798.5	25544	34002.75	27705

Sntg2	30582	32225	34000	32242
Sntn	31318	33268	33654	32112
Snupn	6944	9844	8496	10621
Snurf	18987	19598	19382	23427
Snw1	31359	14237	13739.5	15109
Snx1	30429	23757	26980	24744
Snx10	14522.5	17419.5	13758.5	19214.5
Snx11	37058	40050	27130	33687
Snx12	25993.4	20475.6	18515.8	13965.2
Snx13	27399	35268	27352	25543
Snx14	27914	24834	30396	26144
Snx15	26173	19237	11252.33333	14227
Snx16	21426	21035	20043	19945.5
Snx17	19652	18445	20224	19364
Snx18	12466.5	14493.5	12919.5	17896
Snx19	19472.5	18846.5	39463.5	19791
Snx2	14427	17132	19198	17483
Snx20	5327	11452	4383	9578
Snx21	26425.66667	23480.66667	22608.66667	23140.33333
Snx22	13168	13555	16833	15711
Snx24	11867	22907.5	10841.5	21325.5
Snx25	25138	34731	27132	34260
Snx27	21733.83333	21257	22280.16667	17116.66667
Snx29	18035.5	26780.5	9994.5	25387
Snx3	27490.5	22963	25052	23623
Snx30	17829	11578	9219	7051
Snx31	30913	36168	33885	32279
Snx32	22289	20528.5	17886	18356.5
Snx33	10163	6521	9053	4119
Snx4	34893	37650.66667	33378.33333	36593.66667
Snx5	14365	22570.66667	24262.33333	23380.33333
Snx6	25200.5	26693.5	23939.5	27647
Snx7	24782	32535	25325.33333	28758
Snx8	10599.5	5762	8850.5	4420
Snx9	12393.5	11066	11111.5	8383
Soat1	12698.25	20471.75	23514.5	22669.5
Soat2	21565	22269	19717	25361
Sobp	24567	23468.33333	19036.66667	17786.33333
Socs1	36686	2488	838	2108

Socs2	23959.5	16445.5	11229.5	17628
Socs3	26388	28207	26849	27817
Socs4	28047.5	34743.5	31689.5	36617
Socs5	37559	31906	32488	30884
Socs6	23230.33333	21376	35072.66667	21165.33333
Socs7	33840	35940	38343	38390
Sod1	28326	23663.66667	21715	23579.66667
Sod2	17652	11842	14744	26503
Sod3	36425	32791	34904	29020
sodium channel	37486	23672	31502	32282
Soga1	34385	24360.5	31459	26225.5
Soga3	36617	2242	23	34051
Sohlh2	7288	10122	13138	13109
Son	20650.25	6557.75	8170.25	17722.5
Sorbs1	6258	20962.5	12373.5	11546.5
Sorbs2	35472	21176.25	26232.25	24521.25
Sorbs3	20339	19952	16736	15225
Sorcs1	21487.33333	25879.33333	24655	25234
Sorcs2	33456.5	16321	17931	20758.5
Sorcs3	25487	26725	16711	33559.5
Sord	1051	39401	38454	31018
Sorl1	36272	18314	10608	5431
Sort1	14631.75	11987	10928.75	10397.5
Sos1	23909.66667	20216.33333	21368.66667	21280.66667
Sos2	19389	15782	16315	17395
Sost	6329	9265	15347.5	15608
Sostdc1	32346	33763	40528	5237
Sowaha	34561.5	32716.5	33314.5	31885
Sowahb	5173	4893	1567	1961
Sowahc	17107	28539	20372	15308
Sowahd	35282	29732	40878	34969
Sox1	1614	3270	5821	6149
Sox10	16869	18994.5	23726	27707
Sox11	25583.16667	26896.33333	28841.66667	24944.83333
Sox12	19119	17435	18141	19533
Sox13	13001	38654	12571	5494
Sox14	11330	27814	37457.5	36307
Sox15	22950	26114	27849	30849
Sox17	20385.75	27927	28427.75	28672.25

Sox18	23967	25159	12665	22294
Sox19	8349	8949	11453	10917
Sox2	5418	34147	34203	32479
Sox21	11260.66667	13045	15883	17685.33333
Sox2ot	24371.66667	25929.33333	17356	15529
Sox3	34801	40899	27739	26616
Sox30	30281	31955	33306	31434
Sox4	33991	23831	24388	24664
Sox5	21823.6	23844.2	25429.6	27116
Sox6	31521.8	22440	14850	10404.4
Sox7	26162.5	17093.5	29093.5	20810.5
Sox8	36372	18019	14912.5	20888.5
Sox9	16345	18522.75	21803.75	22259.25
Sp1	22614	15572	17182	12671
Sp100	18327.33333	8684.666667	7023	12123.33333
Sp100-rs	16218	18623	14028	21484
Sp110	3186	180	1089	37754
Sp2	22522	19732	20279	10323
Sp3	12177	13676	17649.33333	18473
Sp4	10477.5	869.5	5124.5	16697
Sp5	1106	712	2792	34812
Sp6	16070	19473	24624	27829
Sp7	3182	4820	11448	11006
Sp8	35871	136	27270	27910
Spa17	40733	122	1105	3035
Spaca1	34664.5	25673	29319	28668
Spaca3	6769	10963	24396	22031
Spaca4	17575	19151	22949	22942
Spaca5	4684	4628	20531	18702
Spaca7	39214	6918	3558	8198
Spag1	5232	5274	7577	8885
Spag11a	15064	16177	21669	23097
Spag16	30237	23416	20977	22865.66667
Spag17	1045	40652	4385	30695
Spag4	18931	12147.5	10597.5	16490.5
Spag5	20057.5	11953	17896	17007
Spag6	30805.5	32679.5	35460	14963
Spag6l	15737	13772	22759	20538
Spag7	36525	2869	5289	4989

Spag9	10059.85714	19091.14286	12032.71429	11343.28571
Spam1	2139	38852	15120	8872
Sparc	22539	18583.5	30430.5	17727.5
Sparcl1	22500	22768.5	10904.5	13110.5
Spast	30947	28834.33333	17502.33333	30308.33333
Spata1	12569.5	15358	30930.5	16121.5
Spata13	28875	16153	28620	17597
Spata16	18573.5	17115	21308	21208
Spata17	13302	3896.666667	29761	25201.33333
Spata18	16996.5	37577	38783.5	40229
Spata19	8060	979	10187	7845
Spata2	14868	13465	11982.5	11700
Spata20	16632	22845	8660	10227
Spata21	18927.5	37563.5	19333.5	17324
Spata24	18328	22480	22305	22602
Spata25	5875	3994	9128	5404
Spata2l	19997	16801	18945	16103
Spata3	7914	39667	12008	6683
Spata31	30313	31820	33407	31781
Spata31d1a	23633	27219	28766	31050
Spata31d1d	16371	18687	21496	21370
Spata32	7354	10639	14816	16420
Spata33	33172	33921.5	31096.5	32293.5
Spata4	33641	31862	37758.5	21211
Spata45	26152	27533	29114	27920
Spata5	20838.66667	14509	15566.66667	19875.66667
Spata5l1	37884	6845	37314	10681
Spata6	18416.33333	14835	16696	16654
Spata7	27468	16959.33333	18062	6366.333333
Spata9	26010.5	28015	27288.5	31952.5
Spatc1	33120	32958	38877	38029
Spatc1l	3716	4830	10832	7738
Spats1	21398	27581	25529.5	24001
Spats2	25814	10320.5	13503	16744
Spats2l	27751	4270	9621.333333	12124
Spc24	19850	23427	29262	35163
Spc25	23228	22071	23625	20848
Spcs1	18005	13950	11833	12456
Spcs2	16776	19606	21438	20785.5

Spcs3	18460.33333	8574.333333	18794.66667	6241.666667
Spdef	10898	15812	8794	8809
Spdl1	21056.5	26540	18988.5	26890.5
Spdya	25693	22360	14791	12600.5
Spdye4a	14195.5	15143	7092.5	25384
Spdye4b	23617	24738	26391	25307
Specc1	20464	16199	22162.5	20085
Specc1l	10074.5	23487.5	17898	18472
Speer2	37994	37326	4092.5	22145
Speer3	29736	36713	40336	40108
Speer4a	31543	33250	34310	3146
Speer4b	32576	3721	37425	35435
Speer4d	37856	39366	10612	38061
Speer4f	893	3668	4172	40839
Speer5-ps1	7917	14454	16381	17506
Speer6-ps1	32460	34717	34787	33021
Speer7-ps1	17440.5	16846.5	12877.5	18003.5
Speer8-ps1	29475	4792	33492	38531
Speer9-ps1	13945	18536	19830	20243
Spef1	18372	16125	14731	14729
Spef2	35938.5	22482.5	37136.5	25357.5
Speg	22854.5	21824	23931	24653.5
Spem1	17193	14193	24283	21045
Spen	33851	34709	32053	30687
Spert	16637	17983	22603	23557
Spesp1	15845	15603	29692	30593
Spg11	12665.2	16851.6	18695.2	9879.4
Spg20	21477	13296.5	20212	14330.5
Spg21	4440	3593	38730	39912
Spg7	15766.5	13403	14577.5	14243.5
Sphk1	31655	27881	25769	29395
Sphk2	38132	31643	33769	33632
Sphkap	23109.5	29202	25310	25829
Spi1	1343	2686	39230	51
Spib	30968	15490	23547	2660
Spic	16256.5	25674.5	12756	27561.5
Spice1	20065.66667	19650	16224.66667	32348
Spidr	19465	20915	18110	21633
Spin2c	30177	2427	33517	38107

Spin2d	27777	39856	30792	29327
Spin4	26066	16765.5	20160	22412
SPINK1	8174	8403	9850	8377
Spink10	26571	25576	27682	26500
Spink12	23969	34893	27177	26176
Spink2	8576	18923	6124	11493
Spink4	17297	14871	25218	22651
Spink5	31073	33320	33370	31713
Spink6	25847	39157	29221	28002
Spink8	1067	39769	33679	32111
Spinkl	26314	35618	29046	33334
Spint1	25951.5	27247	28771	30507.5
Spint2	28486	32062.5	35515	32525
Spint4	33070	35019	35885	33957
Spire1	19056.33333	14575	18417.33333	17555.33333
Spire2	20352	19931	21727	24381
Spn	16214	21241.66667	22725.33333	23861
Spns1	17217	14598	15289	15252
Spns2	6252	11567	5572	39346
Spns3	5342	16525	25931	31870
Spo11	27678	25424.5	23896	24831.5
Spock1	16491.5	22710.5	21711.5	13168.5
Spock2	25431.33333	25372	24816	28917
Spock3	32561.5	34059	36837.5	20575.5
Spon1	21313	23888	29580	26790.33333
Spon2	18601	37492	3810	20833.5
Spop	28350.33333	24745.33333	30354	28240.66667
Spopl	21497	23309	21619.5	22457.5
Spp1	8693	6373	9285	3918
Spp2	31257	32877	34383	32625
Sppl2a	20813.4	21206.8	14152.2	21166.8
Sppl2b	3370	38761	41095	40137
Sppl2c	12438	8789	12193	16788
Sppl3	13666.6	20808.8	21853.4	20797.2
Spr	16833	19382	13446	17032
Spred1	20258.33333	15925	9002.333333	18764.33333
Spred2	22028.33333	18934	21303	23571.33333
Spred3	28489	39978	34182	1829
Sprn	10828	13481.5	19327.5	19008.5

Sprr1a	40891	40584	8384	6987
Sprr1b	37607	36092	3536	37855
Sprr2a1	25690	25739	33941	35640
Sprr2b	9589	11330	12733	12874
Sprr2d	6734	6180	12542	18069
Sprr2e	39635	1270	2890	2050
Sprr2f	40322	6425	5736	2461
Sprr2g	37545	38231	201	40253
Sprr2h	32871	34635	36475	35721
Sprr2j-ps	22764	26061	26028	27745
Sprr2k	799	3906	8956	8968
Sprr3	4735	12047	10607	6444
Sprr4	13347	16205	21929	22613
Sprtn	17720.5	15052	21129	25930
Spry1	8662	9805	15106	15804
Spry2	22714.5	22490	8878	7231.5
Spry3	22865	18022	23444	26595
Spry4	29581	31330	32580.5	31075.5
Spryd3	19671	18052	18045	19775
Spryd4	35152	39954	34090	36428
Spryd7	5301	15224	10058	19833
Spsb1	24702	14269.33333	22366.66667	25685.33333
Spsb2	10109	6690	13059	11868
Spsb3	40525	39021	34185	37647
Spsb4	27963	28205.5	23920	30724.5
Spt1	34607	30754	32116	38316
Spta1	26484	30905	29447	29442
Sptan1	17284	21953	21286	22300.33333
Sptb	13227	15197.75	17392.5	11357.75
Sptbn1	19735.75	20279.5	19218.75	18958.75
Sptbn2	113	2489	6135	5991
Sptbn4	26284.5	5836.5	7286	5553
Sptlc1	19575	18871	17033	17941
Sptlc2	27064.5	30231.25	25330.75	16138.75
Sptlc3	34	40969	12574	8703
Sptssa	12938	13070	18270	13007
Sptssb	23257	24307	26316	25388
Spty2d1	17781	15974.5	16413	19354.5
Spx	28046	34012	37909	39103

Spz1	26550	27897	29442	30731
Sqle	7848	7538	4078	2402
Sqrdl	2763	13085	3422	15754
Sqstm1	734	9762	3706	17595
Sra1	27881	28955	29070.5	30072.5
Srbd1	29191	28395.5	28621.5	9411.5
Src	18121.5	21532.5	26755.5	4332.5
Srcap	22115.4	25616.6	25421	25682
SRCIN1	11774	13885	15748	18070
Srd5a1	23824.66667	15425.33333	21837.66667	14334.66667
Srd5a2	28571	30507	1885	34302
Srd5a3	3516.5	22791	18124	20342.5
Srebf1	10736	10691	9369	9018
Srebf2	28872	26173.33333	22165	20440.33333
Srek1	5174	20695.75	20211.75	20153.75
Srek1ip1	10958	12358	12844	10231
Srf	23935	16931	24519	19939
Srfbp1	7358.5	14157.5	9168	14521.5
Srgap1	18366.75	23100.5	21929.5	28752.75
Srgap2	22163.2	21999.2	15543.2	18459.8
Srgap3	13254.5	30038	16522	28728.5
Srgn	28299	25467	33733	32718
Sri	19240	22056	17485	19883
Srl	16041	11969	20047	11003
Srm	20986	21535	16662	17639
Srms	17177	18980	22496	19588
Srp14	23551	31085	26739	28676
Srp19	5115	10362	11243	10851
Srp54a	11205.5	20683.5	10304.5	14670.25
Srp68	16555	18089	17230	20263
Srp72	24872	23190	18786.33333	20738.66667
Srp9	19571	23100	20773.33333	19265.33333
Srpk1	8442.5	11071.5	9348.5	10320.5
Srpk2	12820.83333	20744.66667	22107.33333	26592
Srpk3	7497	3344	10927	12020
Srpr	13872.25	11731.75	13572.25	14826.25
Srprb	4905.666667	4879.666667	15842.33333	17888.33333
Srpx	5614	4230	10800	21771
Srpx2	13313	4151	14486	15476

Srr	14038.8	14226.6	18256.4	20227
Srrm1	18263.33333	26032	15496.33333	14250.33333
Srrm2	9240	3830	11666	11312
Srrm3	17621	14437.25	18475.5	16889
Srrm4	20891	32944	18300.66667	32336
Srrt	4096	4189	41125	40740
Srsf1	30298.5	37204	37863	39811
Srsf10	22478.5	15428.25	13203.75	13567.75
Srsf11	10579	20088.5	21768.25	24353.5
Srsf12	40151	370	15493	14561
Srsf2	28273.66667	26335.83333	24311.5	21827.33333
Srsf3	34710.5	33786	27316.5	29024
Srsf4	17209.5	15778	17001	15575.5
Srsf5	29098.5	27242.5	33806	34940.5
Srsf6	25179	21941.5	23984.5	25030
Srsf7	34526.66667	22599.66667	31779	20947.33333
Srsf9	15962	14547	18499	16876
Srst	7583	8213	12520	15581
Srxn1	24283.66667	6829	33634.66667	14342.33333
Sry	38483	6822	37186	5566
Ss18	11312	9218	8792	7834
Ss18l1	22298.5	22126	23489	26580.5
Ssb	22056.5	22827	23832.5	27153.5
Ssbp1	21206.66667	25286	19816.33333	20570.66667
Ssbp2	24071	17762	20537.4	19990.8
Ssbp3	5269.5	24103	10490	8467.5
Ssbp4	5808	2397	3840	40438
Ssc4d	7093	10966	12321	12855
Ssc5d	38607	40355	4782	5286
Ssfa2	18247	25693	21383	23935
Ssh1	9703.333333	11271.33333	11922.66667	9794
Ssh2	22301.5	18040.5	23356.5	19934.5
Ssh3	3836	3446	4650	41160
Ssmem1	25825	32766	3472	7736
Ssna1	1206	2868	3105	4207
Sspn	37915	39245	40718	2816
Sspo	17800	22803	31039	27808
Ssr1	22766	6593.666667	9997.333333	8709.666667
Ssr2	37684	2429	1259	5188

Ssr3	16491.66667	20942.33333	18992	20169
Ssr4	10264	12965.5	14773.5	14675.5
Ssrp1	13602	11423	14298	12568
Sssca1	38811	6273	3350	6670
Sst	28771	27815	38400	37872
Sstr1	39768	37352	40181	38809
Sstr2	28399	32299	26156	28924
Sstr3	34945	31766	31472	34215
Sstr4	7980	9612.5	12530	12647
Ssty1	20700	22979	25849	23591
Ssty2	24283	17029	28025	34362
Ssu2	18664.33333	9460.666667	18193.33333	31419.66667
Ssu72	23936	7990	27590.5	7508
Ssx2ip	21887	10296.5	24121	10738.5
Ssx9	33616	34021.5	37100	35124
Ssxb1	2059	4335	6241	40517
Ssxb2	33512	25129	27019	27703
St13	10590.66667	14596	16276	20074.33333
St14	21524.5	20440	23499.5	20908.5
St18	26864.66667	28390.66667	24939.33333	25393
St3gal1	29407	3591	30104	6202
St3gal2	19429	21139.5	19829.5	19769
St3gal3	18542.5	21158.5	15185	18178
St3gal4	9559.5	5610.5	9519	24394.5
St3gal5	7442.5	19649	10602	9632.5
St3gal6	27851	2776.5	8869	8706.5
St5	32384	17115	13239	20664
St6gal1	30393.66667	19396.33333	27216.66667	19786.66667
St6gal2	23338.5	26274	27220	30465.5
St6galnac1	29015	30476	35106	30756
St6galnac2	27823.66667	15085.66667	31273.66667	28737.66667
St6galnac3	18977	19894	8388.5	21931.5
St6galnac4	15405	12781	13959.5	12027
St6galnac5	20613.5	30834.5	30531.5	16792.5
St6galnac6	11601.33333	12744.66667	20000.66667	21731.66667
St7	19715	24619.6	20880.2	15404.2
St7l	23411.5	22891.5	24260	25277.5
St8sia1	8799.666667	11381	20781.33333	18248.66667
St8sia2	17638	21442	34019	35034

St8sia3	30443.66667	32234.66667	34417.66667	24792.66667
St8sia4	15473.5	4021.5	18941	9661.5
St8sia5	21336	4704	13561	9965.5
St8sia6	6422	13834.33333	14972.33333	6020
Stab1	22922.5	19476	23591.5	22215.5
Stab2	19691	13412	19813	16076
Stac	17281	19567	21150	16544
Stac2	8725	11461	9123	12368
Stac3	18755	21303	26394	26853
Stag1	16567.8	22310.8	21941	21358
Stag2	16398.66667	15207.66667	17131	16321.66667
Stag3	21020	8964	17122	14636
Stam	14124.66667	13880	14701	16535
Stam2	22182	24931.5	20646.5	25848.5
Stambp	21806	22263	21058	20850
Stambpl1	19222.5	10860	13481	8341
Stap1	17051	33424.5	15023	34198.5
Stap2	211	13002	3082	11121
Star	26898.5	19074.5	21950.5	23498.5
Stard10	24512.5	13541	10812.5	14631.5
Stard13	12706.5	3869	17693	13602.5
Stard3	12785	9470	11481	9777
Stard3nl	4628	5356	6353	7766
Stard4	26753.66667	20076.66667	24950	24419
Stard5	14096.66667	10192.66667	9825.666667	8150.333333
Stard6	7414	13428	8010	7814
Stard7	23388	23047	20728	21856
Stard8	27314	14330	22087	10461
Stard9	19264.2	21376.6	17015.6	20476.2
Stat1	21420.8	23777.2	14879.2	28663.6
Stat2	10586	11279	4855	11281
Stat3	17174.5	13058.5	14197.5	33693
Stat4	7239	6526	10359	7575
Stat5a	20819	22314	19637	21111
Stat5b	21340.66667	16767.66667	19235	19172
Stat6	9643.666667	21005	12389.33333	9148
Stau1	15004	9444.5	14075	14006.5
Stau2	28048.33333	27513	28261	31014.66667
Stbd1	24578.66667	25271.66667	7999.333333	6708.333333

Stc1	21812.66667	22933	28930	26518.33333
Stc2	38338	279	10011	20016
Steap1	14900	26118.5	10114	17839
Steap2	34538	36802	20301.5	10871.5
Steap3	11946	11618	18482.5	19078.5
Steap4	22932.5	7160	27274	11777
Stfa1	16855	27781.33333	18602.33333	15747.33333
Stfa2	27128	40459	28968	326
Stfa2l1	3237	4290	2848	8505
Stfa3	19799	38019	26536	35382
Stil	26591.66667	22641.66667	27061.33333	27997.66667
Stim1	15598.66667	22626.66667	23077	10316.66667
Stim2	16589.5	19452.5	12843	15580
Stip1	18121	23409	17218	20905.5
Stk10	32815.5	26534.5	25669	6367.5
Stk11	35370	29299	29528	33509
Stk11ip	24802	30841	22930	24290
Stk16	14805	32870	12566	31940
Stk17b	18059	18751	17170	18513
Stk19	10833.66667	10226	7763	10499.66667
Stk24	15739.5	36239.5	39980	21685.5
Stk25	6685	7388	9487	7678
Stk26	17823	17969	19105.5	15405.5
Stk3	16211	13091	13929.75	15823
Stk31	33198	33225	37170	33089
Stk32a	30888.5	23015	25288.75	23725
Stk32b	29566	31060	32700	31141
Stk32c	9037	9187	6011	29396
Stk33	22955	24038	24674	29925
Stk35	5038.5	8860.5	4913.5	9990.5
Stk36	9721.333333	7006.666667	13525.66667	13137.66667
Stk38	33379	26005	24078	27693
Stk38l	18161.5	28425	23936.5	12780
Stk39	8971	8727	8333	8361
Stk4	15935.5	9907.5	26692.25	26546.5
Stk40	7462	10341	3340	3971
Stkld1	24926	20516	13035	11627
Stmn1	32400.5	20445	9727	17609.5
Stmn2	20670	23401.5	20167.5	2483

Stmn3	2386	6312	7219	5019
Stmn4	3388	7981	7242	10314
Stom	9545	4120	7132	3763
Stoml1	22442	21101.5	18509.5	19656.5
Stoml2	12074	18262	16212	19853
Stoml3	17815	16583.5	37806.5	22401
Ston1	14471	17625	18712	17361
Ston2	16324.5	27681	34393.5	35440
Stox1	29020	30910	30962	29545
Stox2	14798.25	17096.75	24887.75	19270.75
Stpg1	33993	39534	1167	5768
Stpg2	30168	31672	33652	32033
Stra13	19627	21047	20919	18932
Stra6	34042	23296	19314	21816
Stra8	21598	11909	21663	16836
Strada	19623.5	25816.75	24550.5	26343.75
Stradb	32041.66667	29445.33333	26179.33333	30015.66667
Strap	4805	7942	7387	8551
Strbp	21289.5	15918.33333	23459.66667	11347
Strc	21111	24531	35740	35579
Strip1	34052	15159	15010.5	14585.5
Strip2	18493.5	12856.25	17195.5	19678.25
Strm	40125	4176	40396	36984
Strn	7749	8168.5	11481.5	11675
Strn3	29319.25	18840.75	16133.25	18353
Strn4	20383.5	20924.5	1871	3103.5
Sts	38535	38199	1080	36198
Stt3a	28960.33333	29504	24048	28034
Stt3b	15543	26861	19956	26456.33333
Stub1	10748	7657	8486	7832
Stx11	10226	21213	16523	24967
Stx12	38516	36386	35073	35482
Stx16	24766	22831	26211	28312
Stx17	19101.5	34488	18977	38274
Stx18	37062	38444	37358	40027
Stx19	37638	26701	38715	27326
Stx1a	34291	12644	15209	10317
Stx1b	31444	26557	28807	27620
Stx2	26169	28716	23054.5	25344

Stx3	13217	14007.5	16098.5	19524
Stx4a	14622	17063.5	22022	25526.5
Stx5a	23734	20917.5	20255	24590.5
Stx6	26965	27517	24445.5	23722
Stx7	17983	17590	15757	14665
Stx8	11469	16957.75	15846	18696.5
Stxbp1	6563	14304	9459	14566
Stxbp2	13707	15544	9202	13319
Stxbp3	11962.5	4631	5332	6470
Stxbp4	20721	11860.16667	13841.33333	14895.83333
Stxbp5	13613.16667	20412.16667	16779	15518.16667
Stxbp5l	29770	31436	28895.66667	22522.33333
Stxbp6	24198.5	23526	24570	21976
Styk1	29648	31107	32701	30990
Styx	10175	10162	23792	4100.5
Styxl1	8987	14813	20536	20434
Sub1	19983	20408.5	18699.5	18974.5
Sucla2	15367.66667	19711.33333	18627.33333	19803.33333
Suclg1	12391	13305	11376	13074
Suclg2	20358	20703.33333	15554.33333	20711.33333
Sucnr1	27475	28587	31029	29634
Suco	13298.66667	27989	10861.66667	19571
Suds3	21132.5	18759.5	19794.5	19071
Sufu	23703.66667	27100.66667	24802	26911.66667
Sugct	22605.75	22615.5	11722.25	21139
SUGCT	40636	35846	33525	35364
Sugp1	6182	3110	6590	6227
Sugp2	21366.25	14792.25	18810.75	19961
Sugt1	10769.5	12734	11928.5	13178
Sulf1	15620	16081	25647	36721
Sulf2	10459.33333	17818.66667	16750.33333	25542.33333
Sult1a1	17221	2937	18698	1726
Sult1b1	28373.5	35903.5	39456	15187
Sult1c1	10313	12157	15811	14837
Sult1c2	12448	17292	12869	20104
Sult1d1	35705	31470	1653	38252
Sult1e1	34528	36074	39343	38218
Sult2a2	34075	34577.5	31622	33386.5
Sult2a4	26533	40204	30001	28703

Sult2b1	30143	30832	15546.5	19505
Sult3a1	23592	40746	3435	40273
Sult4a1	35288.5	19268.5	8929.5	23550
Sult5a1	22447	24290	27832	31971
Sult6b1	34809	39777	29927	36489
Sumf1	6214	5652	4366	3446
Sumf2	23103.5	5552	24074	25603
Sumo1	24203.66667	33859.33333	27824.33333	30446
Sumo2	14386	18657	18007	18249
Sumo3	18249	22418.5	15823.5	18883
Sun1	39659	37024	40130	38148
Sun2	30687.5	22137.5	30656	25939.5
Sun3	34833	33448	36049	36810
Sun5	39517	35351	39301	3057
Suox	8224	7274	9001	5276
Supt16	5898	10329	11596	9389
Supt20	17107.75	21036.5	17530.75	18352.5
Supt3	35966	39136	34995	39996
Supt4a	12465	17501	13557	13388
Supt5	7949.5	24377	9291.5	10342.5
Supt6	36137	31796	36678	38233
Supt7l	23914.5	19874.5	18765.5	16747.5
Supv3l1	2566	5904	2521	4631
Surf1	33262	31458	29107	30181
Surf2	11614	13206	15292	14097
Surf4	12814	15692	12825.5	15437.5
Surf6	9517	8153	9888	9833
Susd1	16426	6031	11041	16093
Susd2	37017	1279	11147	12139
Susd3	36490	30025	35645.5	23910.5
Susd4	24758	36494	106	25790
Susd6	31422.66667	21713.33333	27788.66667	19090
Suv39h1	31820	23586	24287	22900
Suv39h2	21748	23400	26169.6	30580.4
Suv420h1	18783	12676.66667	12397	12742.33333
Suv420h2	25622	23483	28440	25431
Suz12	24368.5	16852	16643	17369
Sv2a	20638.5	17767	22885.5	20397
Sv2b	34108.66667	16831.66667	25961.33333	30357.66667

Sv2c	26711	28073.5	21002	34967
Sva	10501	16318	28308	30450
Sval1	23693	27634	24694	31501
Sval2	16378	15984	24603	25443
Svep1	28508.8	16609.6	23293.4	22219.4
Svil	16551.5	20848	18769.33333	19915.66667
Svip	6342.5	8442	25715	19867.5
Svop	7501.5	11149.5	19364	18424.5
Svopl	24222.5	25111	27899	26243.5
Svs1	5422	10130	11449	21178
Svs2	35970	3690	29885	1315
Svs3a	25313	26507	29358	40573
Svs4	23892	24953	27064	26002
Svs5	16262	16934	18961	17001
Svs6	31374	33712	36278	36505
Swap70	28849.66667	27178	19972.66667	17035.33333
Swi5	18582	19799.66667	23910	12960.33333
Swsap1	19939.5	23507	15290	18061.5
Swt1	21252.25	20147.5	20205.5	19865
Syap1	8182	10308	11897	11988
Sybu	21429	21969.6	26622.8	25581.8
Syce1	39751	1671	26271	28079
Syce1l	13628	9564	12793	16817
Syce2	11637	17180	16487	15152
Syce3	35973	29044	34663	4051
Sycn	17390	19219	21841	21800
Sycp1	5553	33220	9920	5893
Sycp1-ps1	23486	24549	26538	25470
Sycp2	23832.33333	30891	24436.33333	31768
Sycp3	35038	32891.5	33352.5	34585.5
Syde1	39772	2634	4573	9491
Syde2	33591.5	22020	35661	36065
Syf2	19535	21822	18211	21685
Syk	13669	14049	12786	13193
Sympk	23304	22792	23200	23558
Syn1	18042	21254.5	22092.5	14876
Syn2	20523.33333	10435.66667	14881.66667	15455.33333
Syn3	22430	23079.75	25864.25	25827.75
Synb	33658	4871	6497	184

Sync	9919.333333	15687	20425.33333	18700.66667
Syncrip	9834	8528	8320.5	11477.75
Syne1	27226.16667	29432.83333	31810.16667	25926.66667
Syne2	15946.75	19250.25	22251.5	20661.25
Syne3	39970	28275	40366	32717
Syne4	18731	20773	23528	23842
Syngr1	12711	22220.33333	12732	18967
Syngr2	38862	39303	28551	33752
Syngr3	6083	7594	18734	15656
Syngr4	34267	25783	320	40384
Synj1	24452.25	20564.75	14538.5	12721.75
Synj2	7079.75	5707.5	7858.5	6053.5
Synj2bp	13510.28571	17822.28571	22378.42857	22013.28571
Synm	14632.5	14265.5	18554.5	16742.5
Synpo	19927.75	26245.5	25523.25	18403.25
Synpo2	19150.75	29159.5	26793	35375
Synpo2l	13336	10954	22682	23620
Synpr	33875	31587	34227	19196
Synrg	21452	17935	17863.5	17322.5
Syp	21856	18319	24809	13733
Sypl	15453.66667	17196	12884.33333	15974
Sypl2	23042.5	22277	33843.5	31954.5
Sys1	23156.66667	20324	23571	21903
Syt1	16130.88889	22470.55556	18060.33333	24285
Syt10	27558	19574.5	18928.5	22598
Syt11	22537.6	21159.8	13796	13698
Syt12	27937	21557	29470	10868
Syt13	16405	18170.5	17838.5	31170.5
Syt14	31005	32368.5	34574	33003.5
Syt15	19096.5	22314	21780	20605
Syt16	13988	18657	14507.33333	26424
Syt17	15902	12079	12950	14883
Syt2	14127	10070	15838	15907
Syt3	34635	31126	27684	23231
Syt4	27634	29126	17217.5	31265.5
Syt5	15300	12998	11830	11733
Syt6	20226	2815	39924	13303
Syt7	24388.5	27445.75	20235.25	16049.25
Syt8	33597	25991	32189	36731

Syt9	22518	29532.5	28391.5	25901.5
Sytl1	11423	15660	19003	22346
Sytl2	26735	28000	30393	28981
Sytl3	9577	13024	2978	2261
Sytl4	32604	34037	36246	38966
Sytl5	20716	20597	28658	34475
Syvn1	17818	14524	17932	18442
Szrd1	20433	25009	25104	31272
Szt2	10222	8864	11744	11329
Taar1	39350	293	36859	6736
Taar2	701	3415	10289	9763
Taar4	849	40846	5018	6575
Taar9	1098	2671	8683	9095
Tab1	37484	27636	36851	29563
Tab2	19404.33333	10667	16392.33333	17681.33333
Tab3	10206	9022	9476	10531
Tac1	39782	3544	12233	14170.66667
Tac2	16862	21437	21838	21670
Tac4	36236	5492	5444	4557
Tacc1	31271.33333	16677.66667	17756.33333	27329.66667
Tacc2	20345	26441.66667	23435	19995.66667
Tacc3	22384	13144	16211	11572
Taco1	17022	20680	19042	21573
Tacr1	16199	17696.5	19763	18778.5
Tacr2	101	10846	4610	40100
Tacr3	31890.5	35743	35184	19681
Tacstd2	7139	7077	3059	8461
Tada1	16154	12126	12061	16594
Tada2a	14408.5	15498.5	13689.5	15612.5
Tada3	13368.5	11912	13301	9902
Taf1	24999.16667	23244.83333	21910.33333	19969.16667
Taf10	20485	18836	18557	20151
Taf11	27402.66667	19897	19345.33333	21858.66667
Taf12	19507	17290	15481	16805
Taf13	34098	35617	33944	31887
Taf15	12077.5	24122	24035.5	23480
Taf1a	28276	26914	27118	29978.5
Taf1b	35777	2687	39336	40754
Taf1c	6675	4059	5006	757

Taf1d	30460.75	2193.75	21262.75	21358.5
Taf2	11651	7303	1808	36855
Taf3	22890.8	23319.8	23058	16967.8
Taf4a	23407	22519.5	24739	23088
Taf4b	25689	25977.5	34970	33774.5
Taf5	14080.25	16130.75	14331	14851
Taf5l	18305.5	17181	34988.5	34332
Taf6	13987	15596	10829	11984
Taf6l	19475.75	19478	28380.5	28066.75
Taf7	23821	26548	22869.33333	26905.66667
Taf7l	35408	17248	32817	36368.5
Taf8	12307	13823	16776	16944
Taf9	25023	28939	29156.5	28502.25
Taf9b	12510	11875	27814.5	26593
Tagap1	10369	10970	5814	6118
Tagln	26468.5	28437	31143	4952
Tagln2	37970	247	39786	126
Tagln3	19650	13335	23059	17520.5
Tal1	15915	13045	32056	25308
Tal2	10574	34405	10097	5727
Taldo1	5783	9357	9359	14944
Tamm41	27334	30931	25248	22590
Tanc1	5283.666667	6313	21466.33333	11360
Tanc2	21225.33333	19671.33333	21404.66667	19835.33333
Tango2	40164.5	36689	38580	38582
Tango6	40546	365	7272	6416
Tank	10530.25	29109	27599.75	18482.5
Taok1	14947.25	8812.25	9061.75	17266.75
Taok2	27028	22291.66667	27431.33333	24687.33333
Taok3	10923.33333	28112	17190.33333	14421.66667
Tap1	10286	14221	2967	13129
Tap2	14771	16157	12518	15697
Tapbp	28480.5	20680.5	24307.5	19316
Tapbpl	17681	26133	14915	23060
Tapt1	8887	15839	7861	16454
Tarbp2	17329	16515	17369	18450
Tardbp	22468.33333	11528.66667	21257.66667	25845.33333
Tarm1	38861	34956	633	26869
Tars	3754	9042.5	4769.5	8041

Tars2	13783.5	13922.5	10894	14010
Tarsl2	11706.5	10704	10233.5	6343.5
Tas1r1	20119	31887	36955	38974
Tas1r2	28719	31792	39458	37685
Tas1r3	9353	11155	11145	12258
Tas2r102	24729	37934	27815	26638
Tas2r105	25899	14946	32767	33421
Tas2r107	40886	11062	8419	7451
Tas2r108	29541	35472	39618	35193
Tas2r110	26966	28303	30390	28965
Tas2r116	5362	3562	6741	10680
Tas2r118	9760	12595	17633	17408
Tas2r119	32035	37994	36385	38895
Tas2r130	31642	24815	35070	37846
Tas2r135	35241	35204	39886	39468
Tas2r136	31051	35887	3679	6902
Tas2r139	32044	34241	34499	32789
Tas2r140	35452	1396	5143	4469
Tasp1	25563	25201	18078	22924
Tat	10982	12827	21160	21898
Tatdn1	38285.5	19240	31467	18905.5
Tatdn2	21879	21709	23346	24856
Tatdn3	14561	14277	8237	12338
Tax1bp1	15028	14765.5	15606.75	16019
Tax1bp3	3212	39949	35494	35948
Taz	20991.5	35748.5	30089.5	30669.5
Tbata	18502	22162	25650	30821.5
Tbc1d1	13690	8141	13146.5	6522
Tbc1d10a	20580	20451	18365	20604
Tbc1d10b	17498	16282	19109	17754
Tbc1d10c	39234	9	4676	5251
Tbc1d12	22615	24766	25316.5	7071.5
Tbc1d13	16182	19254	15633	18723
Tbc1d14	623.5	32107.5	36958.5	33074
Tbc1d15	21099.33333	23306.33333	22665.66667	24037.33333
Tbc1d16	16845	4013	3974	25841
Tbc1d17	18512.5	19564.5	17384.5	17351
Tbc1d19	4132	7092	4300	5524
Tbc1d2	13594	8944.5	15362	9835

Tbc1d20	20042.66667	18394	21088.66667	28137.33333
Tbc1d21	36162	33131	36565	26259
Tbc1d22a	9230	2135	4589	1600
Tbc1d22b	29614	25235	25132	24669
Tbc1d23	21299	22378.6	24840.8	24704.2
Tbc1d24	10948	12033.5	10335	13736.5
Tbc1d25	36893	35062	37963	34906
Tbc1d2b	12045	16357.5	11064	14952
Tbc1d30	26626	31030.5	11613.5	15308.5
Tbc1d31	22398.5	23039	3405	2961
Tbc1d32	27804.5	18278.5	16790.5	17251.5
Tbc1d4	25430.6	19534.2	18410.4	30801.6
Tbc1d5	20006.4	16651	23388.2	22028.4
Tbc1d7	1940	1777	38610	1063
Tbc1d8	7209	9629	3724	6872
Tbc1d8b	25321.5	23156.5	29673.5	21428
Tbc1d9	12201.33333	20868.33333	11433	10982.33333
Tbc1d9b	20744.66667	20819.66667	17915.33333	18834
Tbca	24270.5	24821.5	23833.5	28016.5
Tbcb	21677	29182	25921	31048
Tbcc	10193	10261	14416	13249
Tbccd1	38922.5	34687	33707	38279.5
Tbcd	28485	25923	23492	27760
Tbce	2107	7383	3221	8192
Tbcel	22683.33333	20596	16650	23200.33333
Tbck	16971.66667	11096.33333	11122.33333	11061.33333
Tbk1	23648	12290	6691	13310.5
Tbkbp1	28506	27445	13468.5	10546
Tbl1x	20422	34122.5	33735	33987.5
Tbl1xr1	13498	27832.5	12445.5	7965.5
Tbl2	17344.33333	24566.33333	28555.66667	31322
Tbl3	21201	22742	19224	22069
Tbp	24744.5	21352	19724	23751
Tbpl1	17453.8	19891.4	19699.6	26738.6
Tbpl2	16504	17927	22905	22248
Tbr1	30639	31819	33647	31963
Tbrg1	5457	7655	7456	7831
Tbrg3	33969.5	27405	28621	26336.5
Tbrg4	20476	20937.5	20371.5	22837.5

Tbx1	23965	25096	26856	25720
Tbx10	39706	37443	30422	39587
Tbx15	32526.66667	33734.33333	3218	14092.33333
Tbx18	25541	27039.5	28213	30355
Tbx19	17094	9932	23788	22407
Tbx2	8889	7414	10309	10318
Tbx20	26962	28340.25	30205	29451.25
Tbx21	22450	26776	27763	34823
Tbx22	31612	34021.33333	34664	23807
Tbx3	15983.5	25394	10557.5	14377
Tbx4	13742.5	12941.5	16246	13509.5
Tbx5	10161	16403.5	11861	14418.5
Tbx6	13733	9848	15787	15643
Tbxa2r	30047	33108	40204	32908
Tbxas1	11368.66667	17482.33333	7031.333333	29897
TC1410399	21503	18154	18658	17103
TC1410439	22510	22518	22280	25651
TC1410447	20224	19852	19454	20545
TC1410512	14389	9429	7694	7770
TC1410573	35999	30886	27957	34946
TC1410591	6116	4696	328	39704
TC1410604	11980	12079	19283	23668
TC1410642	13437	11785	14436	15442
TC1410667	35280	38673	39691	41135
TC1410711	20055	18012	19222	18405
TC1410815	20838	16361	17446	14290
TC1410863	18367	18211	20851	21041
TC1411223	34715	36858	24766	32252
TC1411627	37912	27084	37381	32093
TC1411637	19918	18951	20014	25499
TC1411675	21008	23772	36568	37707
TC1412123	9288	6450	5941	4444
TC1412363	11848	9950	11418	13387
TC1412375	5433	8645	3718	3948
TC1412899	17237	19186	16345	19243
TC1412967	346	4209	32541	39558
TC1413093	8621	10792	596	8011
TC1413394	11194	14103	13616	15944
TC1413629	31795	33567	40183	4000

TC1413663	15857	17988	21987	23266
TC1413911	3847	35650	34772	39229
TC1414063	33752	40812	483	3775
TC1414138	37279	25092	41009	35237
TC1414310	8607	4664	12698	18148
TC1414328	5627	11884	1412	23159
TC1414359	40022	21726	22888	19834
TC1414388	33810	32096	31038	34856
TC1414410	3040	38813	37032	37183
TC1414786	39361	31510	40816	33530
TC1414823	18901	23269	24735	25791
TC1414896	33406	34841	19522	20307
TC1415016	21939	20895	17324	17189
TC1415210	1314	39991	38662	40243
TC1415308	17367	39081	15168	28713
TC1415393	41012	37950	3574	39298
TC1415448	12616	9043	12867	12981
TC1415656	9999	8416	10260	12596
TC1415719	28315	24790	25715	24236
TC1416031	39231	147	37738	7034
TC1416052	35799	28665	36522	33801
TC1416253	23090	22497	32252	34464
TC1416257	580	2217	2950	2225
TC1416353	32578	17160	21339	17054
TC1416391	30954	36864	34240	35147
TC1416410	29616	30498	34216	34958
TC1416446	18050	23643	22572	24383
TC1416461	10681	3429	17693	20905
TC1416647	29235	30819	6142	31200
TC1416670	7315	1961	14852	7839
TC1416737	38688	3121	15696	12483
TC1416851	21933	22025	28936	25615
TC1417120	17948	16089	20970	17304
TC1417206	3735	285	40586	37279
TC1417235	28110	9377	30890	34712
TC1417337	33767	32840	40384	40488
TC1417421	2460	40058	37662	33231
TC1417543	3718	3632	5972	5175
TC1417713	39855	38998	40696	40401

TC1418013	899	7487	8751	13578
TC1418187	24952	28181	27356	27213
TC1418474	19013	16751	12589	18638
TC1418480	6908	3775	4009	7664
TC1418521	21562	20455	18054	16042
TC1418772	23876	25054	26976	34379
TC1418971	5143	4828	8940	10304
TC1419200	4170	13902	6015	17201
TC1419210	7298	2472	7910	8940
TC1419321	3392	39756	1450	1068
TC1419390	6743	4098	6820	6699
TC1419452	29950	7252	20781	20803
TC1419784	17976	23705	27226	22288
TC1419971	23821	15817	14391	8849
TC1420198	34425	36980	40650	37957
TC1420422	3474	11474	17520	22412
TC1420464	35305	29464	39941	38704
TC1420598	31835	33490	35375	33579
TC1420722	5368	6136	12027	15149
TC1420765	1464	38216	38883	38127
TC1421031	17007	17557	16610	22110
TC1421055	16287	16416	7101	24151
TC1421239	30200	17709	21176	20090
TC1421903	20077	23043	23885	20565
TC1422158	17414	9207	16730	13645
TC1422581	10507	12290	18244	16903
TC1423273	35721	38660	40337	4714
TC1424914	1089	1974	7961	6007
TC1425332	7697	35349	30757	23604
TC1425352	6039	10320	6986	4050
TC1425548	2661	36868	6556	964
TC1425983	35611	8963	6778	12120
TC1426818	36149	32955	34261	33706
TC1426983	20904	23439	20560	19359
TC1427508	2222	36504	41028	37542
TC1427712	18919	14765	16283	8319
TC1427873	27523	25834	36975	36919
TC1428425	35894	37124	39253	37192
TC1428572	2420	266	25657	25116

TC1428732	35114	40476	35271	361
TC1428970	1863	40429	38916	40107
TC1429179	28303	30444	35288	32427
TC1429743	29143	21534	21148	22456
TC1429757	6736	7211	3749	3854
TC1429758	4754	13530	6067	10585
TC1429763	15465	14282	17965	18606
TC1429894	19374	21600	26701	28714
TC1429930	9223	10067	8694	12408
TC1429987	10865	6876	11529	13021
TC1430032	22211	20607	18576	24039
TC1430143	13825	12110	10123	11298
TC1430357	13159	11137	11856	11505
TC1430370	11782	5778	8958	13406
TC1430551	3331	27434	23718	15460
TC1430634	1926	1249	38638	40525
TC1431008	17049	18616	18579	20831
TC1431058	22449	25889	36690	36832
TC1431252	33213	34780	37740	6075
TC1431333	38531	35536	24180	32023
TC1431409	13876	12277	6959	11797
TC1431411	9440	5331	2128	38352
TC1431636	34136	29951	32249	31883
TC1431688	3996	586	5842	3646
TC1431967	23005	23735	36787	39533
TC1432382	20657	21256	26867	28801
TC1432518	9344	2518	40911	35989
TC1432613	4620	7302	9739	9310
TC1433083	31125	38050	38898	40302
TC1433510	21215	19557	19608	25279
TC1433704	27710	24045	37724	36542
TC1433768	28161	29638	31201	29751
TC1433866	32615	34347	36295	34179
TC1433903	34670	37578	7557	9939
TC1434027	20639	21057	26768	35834
TC1434196	11314	7286	14180	18277
TC1434341	19214	9810	18705	10771
TC1434594	2764	30139	26507	27144
TC1434979	29490	30981	33466	31814

TC1435025	30101	20501	17574	20767
TC1435115	40739	3231	4310	5532
TC1435215	8996	126	7239	8540
TC1435238	24811	9853	12498	25228
TC1435352	20257	11345	19571	18994
TC1435479	19585	39929	14807	9096
TC1435793	35656	28337	25083	25394
TC1435794	20780	22603	21313	23746
TC1435904	21611	21117	25287	24325
TC1436068	40284	20732	26890	25229
TC1436226	14652	13082	19472	20963
TC1436275	32779	16981	22343	15801
TC1436551	20383	17762	19359	15299
TC1436553	13489	15207	19076	18650
TC1436661	22115	25608	25639	35155
TC1437558	40991	31218	27341	33999
TC1437743	37967	37595	2433	3834
TC1438120	34294	33930	5267	4865
TC1438297	6396	7231	10114	8409
TC1438414	3908	4883	12946	10972
TC1438513	51	39128	4921	6569
TC1438641	33402	24542	22845	27564
TC1438809	25488	25764	39098	38525
TC1438815	9972	4529	2591	377
TC1439005	11736	10648	30582	28747
TC1440113	29966	14999	17496	17326
TC1440174	36896	37570	4698	1922
TC1440579	10346	8342	10188	7887
TC1440691	23377	21608	20553	21481
TC1442221	11359	8578	19065	27432
TC1442897	7991	7519	8560	8169
TC1443778	32103	36007	38891	37234
TC1444368	30442	31973	33963	32324
TC1445114	11487	7049	8377	4476
TC1445297	7692	4255	15734	15292
TC1445692	22841	22819	21060	20208
TC1445742	22349	25127	24739	21930
TC1445810	38093	37811	2356	678
TC1445851	17167	20823	17645	18749

TC1446286	11195	5789	10353	7191
TC1446314	25309	33345	1356	2757
TC1446374	9659	11052	16296	15988
TC1446509	37821	21959	24228	26687
TC1446827	6210	2008	13526	13251
TC1447060	6381	39428	3568	695
TC1447641	27308	20770	18967	16221
TC1447684	1082	12526	6325	16588
TC1447988	19132	22407	20366	23290
TC1448255	7226	9048	19082	19079
TC1448287	14181	9789	7977	4769
TC1448313	12293	7809	6564	8249
TC1448320	1041	36861	109	11729
TC1448370	15440	22122	18248	22105
TC1448906	40505	2379	39182	40850
TC1449044	10396	4340	4630	12442
TC1449235	35677	31960	32039	31947
TC1449385	13321	21527	41076	10645
TC1449405	7470	7408	9670	14051
TC1449546	18533	17398	24214	21708
TC1449652	17648	16910	14749	15468
TC1449811	19605	17325	21703	18897
TC1450063	8480	9369	15609	16215
TC1450333	33949	10493	2303	405
TC1450334	32173	33629	35967	34026
TC1450448	6886	7761	10658	11833
TC1450987	1151	3760	7681	7638
TC1451873	15634	18594	11848	5815
TC1452430	38173	47	2421	2935
TC1452538	34288	36408	6094	5774
TC1452905	29766	34120	37483	40554
TC1453066	13855	18700	22975	25549
TC1453087	220	26747	35256	29487
TC1453715	28297	3224	30721	29313
TC1453818	18293	18090	14910	20054
TC1454581	16152	13724	18398	15953
TC1454619	23979	25421	39203	37868
TC1454733	39469	19245	22008	15675
TC1454811	40380	4790	7814	6518

TC1454836	57	2927	11177	13459
TC1455097	10591	10738	14264	12625
TC1455119	4583	5451	14345	12872
TC1457046	32297	22623	29676	22953
TC1457252	19906	21132	18648	21390
TC1458227	11415	5063	11067	7990
TC1459209	3092	37017	8369	6649
TC1459687	23454	14549	20793	17731
TC1459708	7816	40041	2654	39304
TC1459789	18661	15455	23769	35354
TC1459838	16380	8146	11459	12180
TC1459893	247	4793	1679	7471
TC1459984	4537	4880	7011	5471
TC1460002	38922	36056	38839	37928
TC1460407	30517	2772	39580	15
TC1460566	33821	32469	26431	25467
TC1461004	6725	23332	9916	23487
TC1461328	20167	26984	13821	19043
TC1461420	11379	9040	8572	8329
TC1461435	28993	24168	25491	33314
TC1461516	34518	23702	31815	32851
TC1461913	21103	18171	19633	15797
TC1461965	19151	27247	18441	21270
TC1462044	30510	25415	23088	21129
TC1462119	14594	14827	37329	30457
TC1462323	7708	6564	8245	8415
TC1462346	8745	10784	1424	13037
TC1462411	20421	14214	13414	11958
TC1462473	33740	24296	26315	34450
TC1462517	4970	8769	8949	8453
TC1462530	7681	10813	16974	17922
TC1462532	9710	14602	23789	24946
TC1462623	7516	10238	5291	7353
TC1463051	16385.5	12201	14493	12571.5
TC1463173	6327	10894	3869	9302
TC1463225	14879	7052	12974	14357
TC1463322	39370	12068	39295	16123
TC1463399	24948	30290	36107	36081
TC1463498	508	70	34444	38668

TC1463522	34284	37175	4235	9175
TC1463652	21266	19558	24269	21319
TC1463723	34519	33575	26366	34777
TC1463803	20234	19206	20538	20314
TC1463995	16202	20761	14294	19319
TC1464191	1836	5212	9505	7334
TC1464310	40308	32106	33043	24687
TC1464371	9158	4393	7026	8913
TC1464462	24208	34737	522	41028
TC1464637	27358	23358	26414	28005
TC1464713	34278	32435	37959	34289
TC1464871	11823	7729	3432	1817
TC1465218	29997	26957	24449	23112
TC1465377	3062	37259	40867	38471
TC1465400	190	40889	1434	4869
TC1465503	14823	20592	11676	18253
TC1465518	10659	8049	10041	14374
TC1465597	30203	22505	19768	21594
TC1465806	13662	12682	12394	8845
TC1465854	17651	8678	23805	21616
TC1466019	32149	34633	35129	33153
TC1466459	33623	23334	24501	22793
TC1466492	6190	4581	5012	4899
TC1466632	27397	25042	26221	26959
TC1466661	13763	16158	19845	22084
TC1466953	14504	7535	16986	10028
TC1466985	9612	37363	38550	34465
TC1467194	36861	32859	34647	38506
TC1467208	7520	8091	6921	8446
TC1467241	20512	16827	13863	15542
TC1467337	22748	16254	22661	22944
TC1467473	5353	9722	10471	12155
TC1467536	18170	15581	25766	29256
TC1467970	17480	15962	35905	39205
TC1468120	5884	8372	15758	16091
TC1468432	36958	5235	27234	26832
TC1468445	33556	27355	36163	34692
TC1468837	5005	8764	10444	12091
TC1469191	29556	31206	37961	36834

TC1469196	6850	6555	8531	7295
TC1469276	11935	17675	17651	19344
TC1469302	38126	27757	36869	33342
TC1469328	35749	40032	3768	4110
TC1469347	39184	437	15548	13811
TC1469392	39442	39281	38754	39840
TC1469469	23744	24907	26319	524
TC1469536	11995	6827	15535	9859
TC1469938	40471	41020	37277	6949
TC1470100	26085	27543	31033	3602
TC1471543	6814	10438	22774	19868
TC1471783	13404	13238	9435	12319
TC1472363	2921	37782	4307	1686
TC1473012	14786	12507	15344	14834
TC1473353	13288	8110	8893	6933
TC1473482	23997	22629	23334	20889
TC1473513	8761	11140	15968	15603
TC1473695	13169	20585	15878	12348
TC1473718	17031	15570	21747	22008
TC1474038	26222	31888	32022	36938
TC1474507	14729	18869	26400	31170
TC1474853	39812	723	3710	1656
TC1474992	37595	35722	217	5160
TC1475242	24268	23285	39186	35120
TC1475295	23037	21287	19592	20712
TC1475345	24561	25725	27466	35631
TC1475563	37959	32785	5820	6041
TC1476099	6833	8341	15896	16158
TC1476343	24078	21624	29192	26783
TC1476642	9303	9114	12837	13585
TC1476722	13460	8258	20417	16322
TC1476831	26121	32357	31132	33286
TC1477069	38506	41050	9839	11949
TC1477109	34707	38666	10761	8052
TC1477251	6084	36229	33144	30440
TC1477702	20267	21585	24450	32771
TC1477839	9649	920	1852	40624
TC1477917	29187	19580	17639	13464
TC1478118	13534	17423	15537	19600

TC1478260	39409	35824	25486	33846
TC1478383	4897	3804	4030	3916
TC1478474	37724	32015	32689	38388
TC1478864	13688	16779	15631	18369
TC1479009	39728	39410	39294	35939
TC1479186	2552	7236	11089	12809
TC1479224	29767	21367	16458	15077
TC1479236	7178	6841	40397	1012
TC1479238	19944	24783	24778	34476
TC1479310	5675	10046	8498	7711
TC1479736	16489	9250	22780	21722
TC1479877	15426	4332	1164	7740
TC1479923	14651	18662	17279	21149
TC1479950	10360	5547	7785	7693
TC1480175	34096	38455	38233	38787
TC1480345	12997	6655	11462	4516
TC1480428	38293	36696	2573	39834
TC1480463	28826	29228	27071	32133
TC1480513	400	36992	41052	37697
TC1480539	20797	6946	23187	12099
TC1480545	33237	40848	29032	39034
TC1480729	33271	36740	35651	37231
TC1481001	13715	9466	11771	14119
TC1481003	25053	15379	21225	11146
TC1481131	16129	15458	17728	19352
TC1481270	3565	39965	40354	37368
TC1481553	11988	4008	10897	10048
TC1481563	18233.5	7276	12922.5	5749.5
TC1481637	2073	1014	33201	39661
TC1481729	40753	24046	39205	20042
TC1482326	9360	19438	14387	18153
TC1482443	26362	20026	19035	18893
TC1482477	2162	1097	1677	1707
TC1482479	15298	10689	11933	13631
TC1482838	7864	10953	7956	10512
TC1482865	37382	36953	34166	35037
TC1483082	30394	31272	38752	37618
TC1483241	40013	36975	34418	36405
TC1483381	22771	21240	23409	36830

TC1483755	31544	22226	22598	22470
TC1483763	2014	12329	28758	11883
TC1483835	15472	10742	13315	10832
TC1484357	33780	22241	23056	21728
TC1484422	4108	13292	36764	18653
TC1484648	5944	7672	10442	8828
TC1484664	36087	31569	29560	27666
TC1484701	6863	14967	17580	18265
TC1484949	39965	34890	39778	38377
TC1484973	11745	9609	9378	8351
TC1485047	28616	31813	28674	37227
TC1485330	37283	39059	10094	4649
TC1485374	30277	23920	25731	27512
TC1485599	21571	23792	24604	21471
TC1485946	26552	41080	29575	1499
TC1486015	39447	35007	28986	29732
TC1486081	17	38492	38871	38340
TC1486091	34944	16622	994	30498
TC1486204	13042	15384	19759	21205
TC1486351	8856	3083	16533	16598
TC1486438	24485	35452	34548	33460
TC1486453	31622	38233	35493	38805
TC1486657	5806	3520	37910	36025
TC1486688	5763	11088	7466	4235
TC1486743	34681	37586	33876	31684
TC1486771	3853	6894	13172	3068
TC1486873	28107	40067	2263	29938
TC1487147	31628	33344	35135	33467
TC1487280	38774	27585	40469	37988
TC1487490	8326	10448	14214	14550
TC1487634	26113	29937	37369	37062
TC1487745	10738	10007	6978	9764
TC1487975	22798	23247	20726	21366
TC1488060	31711	34183	37246	33028
TC1488278	28375	31130	34621	36831
TC1488746	27870	29080	35383	33713
TC1489649	16243	2440	6285	38279
TC1489773	37262	35623	36698	36579
TC1489795	24314	25916	35006	33218

TC1489857	14989	7908	9774	20006
TC1490033	14539	9749	3164	14943
TC1490206	27892	34062	36621	39349
TC1490224	21106	20896	35529	33869
TC1490928	13965	5714	5677	36969
TC1491116	33203	40037	13607	40002
TC1491281	25161	30763	37874	34516
TC1491311	19693	34488	18966	30351
TC1492305	16977	6598	1380	9826
TC1493562	19391	19891	24607	21084
TC1493753	20100	24400	25438	25446
TC1493944	25037	20907	12606	17888
TC1494175	31316	38304	25751	34001
TC1494189	39737	31942	38229	31109
TC1494321	33594	28465	34611	37359
TC1494344	10406	8730	10856	16402
TC1494641	2666	4629	8614	11630
TC1495082	26906	28338	29525	28304
TC1495093	5040	7632	11873	12659
TC1495159	32375	33476	35999	35315
TC1495415	7536	4063	15109	10109
TC1495809	27577	37442	37897	37789
TC1495954	40577	11267	12001	14462
TC1496191	10132	12470	8215	14859
TC1496338	36257	36161	27217	37509
TC1496405	32990	22573	24305	21761
TC1496499	18945	23864	23342	24002
TC1496587	20293	18476	18197	17066
TC1497053	17902	16494	18598	17856
TC1497099	36743	32457	28137	28212
TC1497215	22919	35423	2176	2830
TC1497558	9747	1801	3662	41061
TC1497602	27080	28279	36657	38903
TC1497699	24343	38223	24093	35791
TC1497792	11613	9189	9905	9967
TC1498005	27658	24191	24100	23642
TC1498165	4158	9990	13198	13813
TC1498205	2380	38283	33168	36452
TC1498646	17525	5061	9235	6682

TC1498661	12787	13541	22951	39680
TC1498676	20895	32767	18352	30764
TC1499238	38491	40369	26807	26297
TC1499331	2826	7447	90	3346
TC1499427	22926	27304	39339	39209
TC1499589	13845	19387	18473	23418
TC1499591	37683	566	5777	3384
TC1499760	9915	10768	2243	4490
TC1499901	22128	16070	17519	17839
TC1500074	38004	34198	33725	35249
TC1500171	5105	39967	943	32122
TC1500358	12884	16460	14507	15539
TC1500485	24375	25554	27230	26114
TC1500555	23841	19139	18405	17969
TC1500867	1598	39755	39933	38618
TC1500960	16624	8701	17813	11843
TC1500988	22728	36948	28108	25787
TC1501160	22316	21097	29662	24332
TC1501319	8077	2312	3315	5659
TC1501443	6552	5257	38934	5373
TC1501515	5205	6375	5730	8652
TC1501700	18488	20025	23081	22557
TC1501770	4072	36215	32403	28120
TC1501833	27049	20268	22733	22073
TC1501906	28684	28114	3020	39147
TC1501990	19847	40115	11498	38758
TC1502079	17613	17215	20646	17965
TC1502119	37122	40821	4069	1170
TC1502356	7798	40833	1300	1950
TC1502406	30887	33073	32991	31518
TC1502647	14134	8240	8863	8788
TC1502884	23374	24506	2887	38853
TC1503077	18207	21267	27382	25852
TC1503336	16762	14545	20229	21238
TC1503622	22306	22169	27144	32199
TC1503683	18403	19117	22058	23346
TC1503921	38819	22846	26091	23375
TC1504053	6224	21671	17738	36041
TC1504103	22279	16551	23403	26001

TC1504141	838	2915	3559	4446
TC1504545	8814	3336	5801	7085
TC1504555	10833	10163	8585	11157
TC1504633	35356	36321	7859	4790
TC1504758	13452	14311	14828	15761
TC1504814	27360	34960	36540	37457
TC1505025	24506	19160	28568	25710
TC1505134	19012	11757	9632	1915
TC1505292	40364	37445	3440	38194
TC1505839	6622	37858	1461	34636
TC1505847	15313	17213	22508	19827
TC1507087	40497	35420	39710	33108
TC1507360	40086	29696	28316	28880
TC1507585	32451	34335	35727	34249
TC1507774	25019	26104	28346	32768
TC1507823	13904	15850	21736	22772
TC1507911	38289	26669	1251	39009
TC1507956	35579	20521	35873	34273
TC1508253	29241	30746	32235	30723
TC1508466	2211	4181	7086	6408
TC1509035	6289	9770	12344	13534
TC1510315	23324	23610	34429	33612
TC1510329	35996	22126	20328	15741
TC1510514	28958	34196	36831	3238
TC1510693	2326	5751	9690	9607
TC1511137	23362	23255	37987	37366
TC1511291	12228	18178	19232	19662
TC1511483	20662	22589	35768	26675
TC1511722	37854	31371	17066	20462
TC1511760	37186	24345	26309	4222
TC1511972	39265	29910	38923	33756
TC1512204	17385	7269	18931	5645
TC1512541	32030	31083	3504	837
TC1513547	2188	6063	9121	10844
TC1513595	15491	6478	32699	36002
TC1513887	22854	19158	25703	36789
TC1514005	33106.5	29421	12072.5	11791
TC1514012	27050.5	29552	30544.5	31406.5
TC1514428	21899	18988	20764	20591

TC1514537	21817	21549	20102	20728
TC1514546	19578	18766	26005	24429
TC1514978	38673	41048	33209	38027
TC1514992	4853	11610	7398	15223
TC1515090	26415	29326	26523	25528
TC1515274	933	24396	26040	29027
TC1515419	20814	19617	23335	26413
TC1515471	21685	15718	16417	16359
TC1515730	31430	22007	25578	12575
TC1515832	38505	34714	36003	29933
TC1515953	19680	21708	22310	23155
TC1516201	34856	29693	2167	31726
TC1516250	34573	31355	30689	38545
TC1516267	5643	8028	40057	1739
TC1516362	17132.66667	8835.666667	14540	34927.33333
TC1516383	19692	21260	23474	24107
TC1516393	7028	2814	3081	3187
TC1516600	25907	1718	9157	7778
TC1516608	27150	27737	29242	22453
TC1516668	11686	20132	21695	33689
TC1516845	20955	19039	18404	17446
TC1516904	9853	8584	9925	9510
TC1516974	10142	16097	29042	15336.5
TC1517038	18643	18118	18135	18991
TC1517240	35638	33726	32038	29306
TC1517350	11210	10940	18321	18414
TC1517425	7491	714	2327	39478
TC1517524	18446	16453	18747	19458
TC1517582	6652	1659	3841	1811
TC1517658	16819	14365	14399	15575
TC1518060	29004	3905	30342	31447
TC1518072	13513	14552	18674	15963
TC1518278	27597	22389	25256	24346
TC1518438	39702	33295	38835	37350
TC1518753	12437	10891	10812	9352
TC1518766	14597	13573	14917	11065
TC1518832	16951	11977	12080	14049
TC1518976	26288	12196	14461	13773
TC1519260	20378	21100	26666	26050

TC1519646	28762	25441	26863	23704
TC1519762	12197	16983	12170	20693
TC1519807	3846	36328	4280	28671
TC1520063	16703	20986	24202	22810
TC1520091	27806	9657	18094	17951
TC1520131	35477	32719	36633	36636
TC1520140	12351	2534	33639	38081
TC1520423	5997	3880	4950	5453
TC1520483	5315	1053	3860	30875
TC1520600	1241	4660	3308	6639
TC1520674	30974	32616	34232	32601
TC1520710	16978	13462	16158	14999
TC1520898	34371	39164	4809	5381
TC1520969	40594	1021	6337	4068
TC1521029	35132	766	36935	36204
TC1521156	30863	37667	38414	40078
TC1521263	40676	35364	29219	23691
TC1521271	40536	40390	5687	38498
TC1521417	40152	38332	40332	39227
TC1521474	30446	21271	14820	12534
TC1521512	14707	13328	21368	20689
TC1521521	19858	23071	23122	19566
TC1521571	15829	19479	18896	20877
TC1521712	38950	38407	33198	37471
TC1521782	8346	38431	7833	6035
TC1521889	11055	12886	23414	25679
TC1521899	40186	19850	21506	25138
TC1522038	20043	33467	15715	34105
TC1522119	35399	1767	37189	731
TC1522598	39085	24825	30376	21580
TC1522631	11825	8930	11798	7560
TC1522673	21996	19370	18504	19230
TC1522927	24234	26956	25112	19644
TC1523060	3789	40713	9852	6099
TC1523491	19176	2437	5857	39281
TC1523645	36287	35598	39581	39291
TC1523778	20318	17780	25270	23129
TC1523833	40579	36716	490	39738
TC1525324	36384	35451	29122	27850

TC1525352	13722	11849	10303	6059
TC1525769	38213	21134	23654	22627
TC1525814	25178	38464	30477	1094
TC1526069	1640	17639	12691	13981
TC1526893	31790	32935	36240	34647
TC1527591	11596	16175	22609	21496
TC1529036	22007	8821	11955	9620
TC1529376	39871	4566	13288	14375
TC1529825	2234	36557	3060	6268
TC1529876	11656	14863	15491	14579
TC1529953	3622	8060	7873	12031
TC1530097	840	31397	2756	1433
TC1530172	39152	26571	31668	24985
TC1530268	38297	4921	9024	5198
TC1530331	1164	12795	9993	16694
TC1530514	2361	4114	6390	6803
TC1530636	33577	22525	23427	25871
TC1530779	16719	12642	14403	10113
TC1530870	36033	21972	28611	25608
TC1531561	1167	1684	4886	3282
TC1531662	35092	35510	30493	35395
TC1531686	7813	8275	4577	12774
TC1531727	9429	7906	4199	7816
TC1531749	24672	20870	24686	22705
TC1531841	6193	4501	4677	8102
TC1532349	745	23261	24916	21083
TC1532550	35494	33183	36434	35314
TC1532585	23194	24253	26227	25148
TC1532651	23220	32428	22591	24786
TC1532654	21492	20308	19470	21223
TC1532719	16685	17811	17730	16530
TC1532732	2593	8367	2022	7651
TC1533174	28767	14629	27323	37637
TC1533197	21272	21613	20498	23456
TC1533274	38285	826	16155	16132
TC1533357	12511	14684	11309	11637
TC1533506	13658	14224	16767	17176
TC1533758	34463	22186	24293	25662
TC1533832	18014	17028	17154	18418

TC1533927	38468	38725	28100	37131
TC1534658	16304	20573	24310	29387
TC1534793	23741	17913	18672	20312
TC1534798	16669	19240	24476	23030
TC1535087	6215	9326	5843	14791
TC1535284	18287	12338	15135	8674
TC1535306	16627	23163	9855	20922
TC1535961	17994	21806	15498	21514
TC1536100	38713	686	47	5008
TC1536164	16348	18622	10222	18947
TC1536354	39627	38914	1129	40320
TC1536584	6293	36973	40962	37501
TC1536622	22436	23150	25150	25792
TC1536702	39347	12195	1667	12726
TC1536703	2026	38228	40253	37399
TC1536721	38929	1043	39862	31799
TC1536731	18605	4915	17204	20027
TC1536734	17144	15867	9421	13377
TC1536826	19086	12241	14625	12722
TC1536898	30861	1850	40404	35611
TC1536985	28342	35643	40549	4998
TC1537300	33352	14649	21260	19042
TC1537769	10849	9086	15019	8665
TC1538551	23125	28260	39422	40803
TC1538868	9568	34260	8100	34316
TC1538885	33243	24157	25724	27267
TC1539049	32833	37122	6070	30877
TC1539197	17520	15476	14980	14897
TC1539342	36744	532	40882	2873
TC1539507	20059	13986	15176	11517
TC1539813	15340	20977	21044	34096
TC1539980	8491	16111	11503	16532
TC1539994	28178	29938	30102	28811
TC1540057	17866	17474	17186	18820
TC1540204	6098.5	10310.5	8201	10181
TC1540687	16301	31914	5214	10929
TC1540715	34907	39067	5554	4876
TC1541044	8146	9682	14413	12921
TC1541384	12292	18119	25681	22773

TC1541591	22624	16614	38082	24874
TC1541724	32625	34041	35806	33897
TC1541749	21097	23765	30065	32075
TC1541797	12697	7162	10133	10291
TC1541923	11665	7812	9535	9023
TC1542265	24864	21205	25719	25457
TC1542400	36587	26118	28732	279
TC1542481	4244	36630	1050	3142
TC1542709	3946	38083	17130	15222
TC1542722	2574	37340	33224	40006
TC1542750	147	38026	40651	38988
TC1542803	6678	11804	19274	17955
TC1543118	1433	37437	458	38925
TC1543130	8564	11013	10756	10819
TC1543265	7115	531	40969	3005
TC1543649	30012	31557	33054	31468
TC1544213	20528	20213	27333	23956
TC1544275	37230	40162	32359	11819
TC1544307	11540	15419	21222	20974
TC1545132	36790	36262	30622	29279
TC1545135	19855	22197	23877	24131
TC1546192	24103	25362	38325	39815
TC1546511	15844	15736	15197	16263
TC1547448	17168	17291	19668	19132
TC1547579	14467	691	6889	4846
TC1547759	15785	11761	12241	12071
TC1548420	34306	2717	36380	35370
TC1548551	21057	18461	23531	20893
TC1549319	21657	26278	29110	27705
TC1549841	23181	23069	19089	19203
TC1550015	12030	37847	39681	9041
TC1561961	14834	17469	18408	18963
Tc2n	27340	18306	37210.5	19345
Tcaf1	28306	15554.66667	20086.33333	21000.33333
Tcaf2	39233	37189	759	37946
Tcaim	24012	21045	21847	25305
Tcam1	10593	11299.5	30239	14734.5
Tcap	33386	35016	37767	37935
Tcea1	21154.5	19884	23818.25	19928

Tcea2	38618	39248	33432	39102
Tcea3	19828.5	18356.5	25044	23799
Tceal1	16521	15532	17597	11020
Tceal3	13021	14295	25631	25746
Tceal6	28971	32243	35675	36927
Tceal7	38156	22888	35699	19597
Tceal8	38714	4374	9469	6177
Tceanc	28818	31281	33232	33701
Tceanc2	24951.33333	21764	22673	24456.66667
Tceb1	13318.5	18007	14277	16269
Tceb2	19890.5	26388.5	25152.5	26942.5
Tceb3	16406.33333	15828.66667	14070.66667	11603
Tcerg1	40280	40671	712	41131
Tcerg1l	22677.5	24220	18462.5	9221
Tcf12	15840.875	25528.75	18289	23204.125
Tcf15	6058	3372	30060	5298
Tcf19	11808	13035	15778	17815
Tcf20	29571.66667	33112.66667	20597	20691.33333
Tcf21	4931	1869	16891	7844
Tcf23	30160	18962	23961.5	10225
Tcf25	19620.66667	18305.33333	20107.33333	20178.33333
Tcf3	27807	28155.66667	16630	26217.33333
Tcf4	18645.57143	17112.28571	18892.28571	18584.85714
Tcf7	27015.5	26762	16936.5	16153.5
Tcf7l2	20915.25	22690.5	19628.75	14460
Tcfl5	39887	4848	40565	35956
Tchhl1	33269	40347	37433	39995
Tchp	7427	4368	7081	4301
Tcirg1	32421	18038	19987	17875
Tcl1	10464	12530	17358	17936
Tcl1b1	25654	24947	27869	27988
Tcl1b2	18948	19853	24953	24624
Tcl1b3	10004	12613	15607	15668
Tcl1b4	13854	14239	18513	18194
Tcl1b5	17355	21284	16863	18826
Tcn2	21648	19211	20695	19819
Tcof1	24842	20038	21632	17435
Tcp1	9399	12238	6529	13439
Tcp10a	33574	9003	23634	4120

Tcp10c	17881	2049	23422	3979
Tcp11	13877.5	18844.5	18681.5	26058.5
Tcp11l1	3368	10689	8806.5	13228
Tcp11l2	11234	13887.66667	16949.66667	20138.66667
Tcp11x2	32813	35059	4038	33240
Tcrb-J	13461	14125.5	22602	18281.5
Tcrg	18757.25	22459	19158.75	30612.5
Tcrg-C	16993	20958.5	20204.5	21260.5
Tcstv1	23956.66667	13506.33333	20829.66667	17066.33333
Tcstv3	35200.5	36188	1131.5	1868.5
Tcta	35183	36669	33845	34251
Tcte1	32135	33724	35853	36221
Tcte2	18458.5	16141.5	22485.5	22180
Tcte3	28759.5	18130.5	23244.5	3635.5
Tctex1d1	35187	31726	28272	27124
Tctex1d2	10453	17427	15736	14207
Tctex1d4	19478	17548	22684	22206
Tctn1	40042	1933	2296	3939
Tctn2	21331	15942	24730	24534
Tctn3	26526.66667	24690.33333	26793.33333	28261.66667
Tdg	3085	6707.5	23705.5	5632.5
Tdgf1	8910	17247	28798	22753
Tdh	34901	2668	37496	37879
Tdo2	24801	26408.5	20128.5	15747.5
Tdp1	6039	17482	23786.5	4746.5
Tdp2	12871.5	13378.5	15669.5	16169
Tdpoz1	25729.33333	25414	30451.66667	17192.33333
Tdrd1	36575	17099.5	20354.5	3624.5
Tdrd12	7538	8883	24881.5	12396
Tdrd3	24155.75	22756	19454.5	21729.5
Tdrd5	23403.5	23433.5	26451	29665
Tdrd6	33835	35492	31870	34376
Tdrd7	3873	1541	2594	3150
Tdrd9	24572	25751	27635	26526
Tdrkh	19048	13261.66667	29220.33333	17555.33333
Tdrp	38959	39333	40892	30180
Tead1	11451.5	19034.75	23268	15938.75
Tead2	26111	26933	14578.5	16000.5
Tead3	25931	31734	38960	36732

Tead4	28734	3159	39340	2506
Tec	23768.66667	25507	29112.33333	23769
Tecpr1	22993	26950.5	19589	27626
Tecpr2	27883.75	14615.25	19453.25	13045.75
Tecr	34694	35832	24719	29055
Tecrl	853	4812	37426	7019
Tecta	36823	29031	6107	5073
Tectb	31878	33523	3371	8065
Teddm1a	40198	35132	34747	34535
Tef	15895.75	21926	20705	21352.5
Tefm	36114.5	6077	18139	20442
Tek	13434.5	15257	18451	17428
Tekt1	21834.5	3251	17688	22346
Tekt2	8245	7683	11694	14947
Tekt3	1473	38428	1	224
Tekt4	8891	10829	13595	12713
Tekt5	19756	18807	15900	22258
Telo2	1550	2841	1915	2384
Ten1	12902	17086	11117	11954
Tenm1	24232.16667	23120.08333	28481.91667	23629.16667
Tenm2	24455.28571	23239.85714	26268.28571	26785.28571
Tenm4	19977.75	10526.25	16177	16921.25
Tep1	8039.5	19740.5	26427.5	17999.5
Tepp	40565	2265	4550	6278
Terc	22538.5	17236	23956	9046.5
Terf1	39638	1200	2497	1967
Terf2	14399.75	12731	12689.25	28959.25
Terf2ip	10764	11255	12139.66667	10079
Tert	7762	11115	14844	14822
Tes	23523.5	27752	29673.5	13130.5
Tesc	25551	19648.5	24874.5	23134
Tesk1	9967	2788	10245	1671
Tesk2	35401	31886	26436	24363
Tespa1	32885.66667	21819	34902	15687.66667
Tet1	21012	27664	27669	12017.5
Tet2	14676.33333	17812.33333	17848.66667	17990
Tet3	14785	13601	12987.5	9617.75
Tex10	21962.33333	17767	16226.66667	16186.66667
Tex101	18463	18289	24001	24605

Tex11	25068	26268	28250	27043
Tex12	26611.33333	22645.33333	27093.66667	22348.33333
Tex13	29352	30599	32672	40739
Tex13a	31073.5	31436.5	32658	16602
Tex14	28931	26215	36804	30962
Tex15	11673	11422	12225	17053
Tex16	11376	37879	6197	33708
Tex18	30508	32144	34175	32441
Tex19.1	24482	23448	29948	24891
Tex19.2	27114	35984	24135	35656
Tex2	19333.5	35059.25	18034.25	25797.25
Tex21	24342	24011	36375	36195
Tex22	26034	21792	41081	37184
Tex26	5526	25177.5	6649.5	10986
Tex261	13503	10379.66667	9949.666667	8347.333333
Tex264	8577	7334	13743	13613.5
Tex29	33738	27094	29277	29527
Tex30	20170	24524.5	23047.5	22697
Tex33	26535	25120	37471	33476
Tex35	32939	34498	5683	34818
Tex36	20931	21829	20957	22414
Tex37	23768	24931	38189	39151
Tex38	13700.5	20121	23974.5	23728.5
Tex40	18447.5	21623.5	3379	7121.5
Tex43	21682	27264	25640	26264
Tex9	26645.66667	29543.66667	27718.66667	22082.66667
Tfam	18031	19539.66667	18028.66667	19747.33333
Tfap2a	3547	7051	9957.5	25430
Tfap2b	19195.33333	21390.66667	25487	21978.66667
Tfap2c	17362	12665	15745	11689
Tfap2d	11773	13953	23036	15350
Tfap2e	30556	32778.5	22755	33656.5
Tfap4	13961.5	15346.5	16712	18239
Tfb1m	11550	15928	20044.5	20054
Tfb2m	38155.5	1568	39753	20384.5
Tfcp2	24570.33333	10415	14059	28189
Tfcp2l1	12267	15773	10228.33333	11587.33333
Tfdp1	17495.5	19290.5	15755.5	15545
Tfdp2	16923.66667	22547.66667	22939.66667	16000

Tfe3	23875.66667	21062	18373.33333	19440
Tfeb	38663	30587	25998	24312
Tfec	20392	30716.5	22045	31536.5
Tff1	7313	9738	13997	13405
Tff2	9023	11353	12553	15730
Tff3	3529	39693	1172	5764
Tfg	29328	25382.5	24087	26942
Tfip11	10022	8415.5	10273.5	8620.5
Tfpi	26483.4	27629.8	24137.6	21373.2
Tfpi2	30240	37254	33357	40670
Tfpt	34730	40029	40475	659
Tfr2	9340	36038	17451	1067
Tfrc	292	41051	38066	38246
Tg	19544.5	22508	12769.5	19945.5
Tgds	3228	4195	3808	8242
Tgfa	20172.5	20026.5	23717	23810.5
Tgfb1	38853	35322	36432	34317
Tgfb1i1	19310	26441	12888.33333	18124.33333
Tgfb2	17118	5265	36451	20144
Tgfb3	12470	9619	18001	18101
Tgfbi	34018	14435	12183	40541
Tgfbr1	18758.5	38332	16161.5	17683
Tgfbr2	14885.25	14457	14121.5	11118
Tgfbr3	19013	21542.33333	23475.66667	23082.66667
Tgfbrap1	18949.66667	15959.66667	28342.33333	26834.66667
Tgif1	21454	22751	24801	26216
Tgif2	20428.5	20031	18226.5	21649
Tgif2lx1	31140	32773	34449	32691
Tgm1	34652	22523	38320	25914
Tgm2	18095.33333	11340	22768.66667	12525.33333
Tgm3	148	2530	5118	5242
Tgm4	34010	22310	35564	24016
Tgm5	11650	13536	24936	10392
Tgm6	20277	20908	36007	30459
Tgoln1	28139	14211	29371	13114.5
Tgs1	14086.2	15327.4	14254.4	19346.2
Tgtp1	25615	21620	12858	7098
Th	4015	5016	8559	7389
Tha1	5294	29948	1371	25517

Thada	22802	27929.33333	29223	17883.33333
Thap1	11150	18503	19236	19596
Thap11	6273	4342	2870	615
Thap2	15212.66667	9030	13132.66667	9489.666667
Thap3	6225	5244	6969	3271
Thap4	21649	23360	21283	23462
Thap6	34145	19860	25721	23685
Thap7	22723	23159	22072	22442
Thbd	23012	7959	23085	8771
Thbs1	11369.33333	9501.333333	13072.33333	11647.66667
Thbs2	18860	14943	26231	29618
Thbs3	35739	29589	36045	33645
Thbs4	19253	25506.5	12257	15097
Theg	17823.5	9665.5	8396.5	13630.5
Thegl	25855	22734	27081	29449
Them4	17922	12334	11823	13016
Them5	25998	27342	29686	37655
Them6	12443	9971	8014	12875
Themis	20301	20958	26423.75	23949.75
Themis2	17187	2260	13611	370
Thg1l	18263	29772	23903	31784
Thnsl1	29585.5	30024	26660	28011.5
Thnsl2	1861	38423	36844	30810
Thoc1	24931	24449.75	25197	17777.25
Thoc2	19169.375	19633.25	16307.125	19116.625
Thoc3	15118	13730	12339	12108
Thoc5	21898	22220.5	20304.5	20529
Thoc6	29472	34086	27917	35023
Thoc7	27269.33333	20491.33333	12317.66667	15455.33333
Thop1	27492	26212	22596	23071
Thpo	23953	27715	27016	27517.66667
Thra	24923.5	18945.5	28209	22303.5
Thrap3	14849	22381.66667	35375	31191.33333
Thrb	12003.5	14433.75	10386.75	24383.5
Thrsp	12909	15509	11396	11825
Thsd1	32852.5	25146.5	20820.5	13437
Thsd4	23491	28688	40166	1334
Thsd7a	18572	19215	22901	20547
Thsd7b	22985.375	14836.625	28063.25	26298.5

Thtpa	9644	9315	8976	7534
Thumpd1	20930	30666	28816	27540
Thumpd2	35834	36533	36609	33797
Thumpd3	21758.33333	26025	12419	13366.33333
Thy1	19202.5	6217	26067	27516.5
Thyn1	21405.5	16119.5	14857	12213
Tia1	17097.5	10187.25	12573	20058.75
Tiaf2	3700	5507	9379	12386
Tial1	17024.5	16186	17850.5	19291.5
Tiam1	28358	5891	14871	11536.5
Tiam2	20480	9896.666667	27868	33251
Ticam1	8276.5	5034.5	5736.5	2792
Ticam2	12702.5	13309	28741	31770
Ticrr	15586	27126.66667	24476	24930
Tie1	29345	35542	30109	35622
Tifa	39012	4470	38083	2288
Tifab	16563	10574	17232	8423
Tigar	18385.66667	14243	19648.33333	12038
Tigd2	13881	13539	10586	11159
Tigd3	11671	14720	17382	19162
Tigd4	16199	19300	22114	21641
Tigd5	21791	18369	22224	20638
Timd2	13493	15099	19129	18017.5
Timd4	25639	21820	31876	24175
Timeless	9690.5	24583.5	4567	20324.5
Timm10	15440.5	14600.5	15253.5	18757
Timm10b	7790	8857	8813.333333	19166.33333
Timm13	22695.5	29782.5	27792	32563
Timm17a	20158	2353.5	33393	39308.5
Timm17b	17191.25	18917	17427.75	15742
Timm21	12513	16115	15384	15688
Timm22	21536	21099	20205	19757.5
Timm23	13892	20015	15925	20562
Timm44	38623	40311	40451	39839
Timm50	15853	17501	15945	17617
Timm8a1	25266	38790	31688	37477
Timm8a2	21581	18835	28325	20622
Timm8b	7238	15535.5	11349.5	14262
Timm9	10124.5	12130	14398.5	14559.5

Timmdc1	16801	16250	15918	16001
Timp1	38438.66667	26877.66667	5589	12894.33333
Timp2	4368	20575	38105	19092
Timp3	5505	4833	13760	22851
Timp4	26762	37509	4657	3495
Tinag	24771	26570	39729	26038
Tinagl1	14091	12534	16489.5	16723
Tinf2	12201.5	8345.5	9487	5272.5
Tiparp	33172	28398	26750	34779
Tipin	14379	15281	14521	15760
Tiprl	23806.5	27104	24479	25846
Tirap	8877.5	5319	7669.5	4723
TISP22	25425	26658	29008	37822
Tjap1	11689.5	32616	37669	38747
Tjp1	10205	16465	20840	24146.5
Tjp2	21555.75	23896	30302.25	23034
Tjp3	20674	17216	18172	12049
Tk1	17481	20467	19670	22757
Tk2	23129	16731	20782	14521
Tkfc	18946	20151	21478	21531
Tkt	23709	20462.33333	32170.66667	22626.66667
Tktl1	25728	27196	30256	36119
Tktl2	33500.5	16229.5	38685	34976.5
Tlcd1	24970.5	27408.5	8123	8479
Tlcd2	21822	21507	22128	23168
Tldc1	9878	8058	8565	6801
Tle1	7296.333333	19772	29090.33333	25917.66667
Tle2	1441	6563	31026	149
Tle3	27939	25862.5	14295.5	12971.5
Tle4	9519	2739	8185	2133
Tle6	5380	6548	36750	36977
Tlk1	17683.5	7324.5	26894	18780
Tlk2	29903	12382	25998.33333	11375.33333
Tll1	34177	28192	32147	26715
Tll2	26707	27864	30978	29505
Tlm	12591	19291	13246	14847
Tln1	19945.33333	14366	19454	13085.33333
Tln2	12813.66667	23066.33333	20552.66667	24455.33333
Tlr1	26214	36251	18786	28502

Tlr2	22720	17097	19852	16377
Tlr3	22741	32349.5	33168.5	30415.5
Tlr4	24949.5	23516.5	25004	25388
Tlr5	22928	35503	21397	23289
Tlr6	19105	20913	15807	19465
Tlr7	38193	23842	24584	26258
Tlr8	37286	19934	29904	15842
Tlr9	15595	15194	19110	18246
Tlx1	37107	2867	31798	687
Tlx2	12633	11860	13857	14300
Tlx3	38315	35949	1923	36687
Tm2d1	39331	7320	6288	8511
Tm2d2	29004	32637	25291.5	29830.5
Tm2d3	8015	8486	3803	8214
Tm4sf1	19863	26481	24299	36459
Tm4sf20	29688	35113	36554	32062
Tm4sf4	12696	13583	20625	19399
Tm4sf5	9769	14002	17391	20982
Tm6sf1	33821.5	15514.5	21999.5	11649
Tm6sf2	35645.5	36564.5	20200.5	39175.5
Tm7sf2	18976	20286	11832	11322
Tm7sf3	37107	32050.5	24574	22716
Tm9sf1	5337.5	8047.5	5677	9624
Tm9sf2	24774	20331	20378.5	18415.5
Tm9sf3	30245.5	11419	11104	12645.5
Tm9sf4	16780	18644.33333	20287.66667	19852
Tma16	15461.33333	17205.33333	21131.66667	19919.33333
Tma7	27006	10846	29364.5	28727.5
Tmbim1	20086.5	16987	17752	20768.5
Tmbim4	35174	20859.5	35816	36522
Tmbim6	23341	22375	22016	21269
Tmc1	25189	27167.5	29578.5	29424.5
Tmc2	22168.5	24327	29415	26578
Tmc3	21526.66667	23678.66667	27038	26367.33333
Tmc4	26627.33333	39827	14011.33333	13152
Tmc5	12539	15240	16777	20759
Tmc6	35926	32986	14600	14723
Tmc7	21723	22530.33333	16838.66667	16167
Tmc8	40340	1002	509	40128

Tmcc1	21640.5	21529	21187.5	22446.5
Tmcc2	3963	4597	4871	1783
Tmcc3	4759	18882	17696.5	31372.5
Tmco1	4611.5	13644.5	15675.5	6529.5
Tmco2	24574	25787	28576	26611
Tmco3	9596	11004.5	7468.5	12156
Tmco4	17948	23056.66667	18723.66667	18834
Tmco5	12776	17743	29188	28025
Tmco5b	40282	39975	8739	7
Tmco6	30518	39082	35298	39765
Tmed1	27003	35358	31851	37743
Tmed10	32289.5	29603	31547	30523.5
Tmed11	25636	27038	28755	27511
Tmed2	19082.66667	25932.33333	23696.33333	28476.33333
Tmed3	28378.5	29176.5	34879	29571.5
Tmed4	21717	22853	19969	22941
Tmed5	36781	35937	38977	39737
Tmed6	29645	30845	32246	30908
Tmed7	23186.33333	10504.66667	16871.33333	17194.33333
Tmed8	18317.5	21373	16062	21879
Tmed9	36700	40549	2860	3968
Tmeff1	24551.5	25639.5	13870.5	16769.5
Tmeff2	24071.75	22841.25	26484.5	27508
Tmem100	9667.5	25785	11775.5	13440.5
Tmem101	11394	11079	11801	11705
Tmem102	25959	27335	28894	29100
Tmem106a	7971	18857.5	23924.5	16701
Tmem106b	7918	25153.66667	24483	25246
Tmem106c	22696	26437.5	19104.5	21993
Tmem107	6801	8219	8663	7582
Tmem108	9363.5	11963	15156	17080
Tmem109	33823	19739	24032	21177
Tmem11	8523.5	10051.5	9929.5	10349.5
Tmem110	19540	31716.66667	19928	27790.66667
Tmem114	40669	29569	210	28274
Tmem115	16939	17139	19695	18889
Tmem116	6564.5	6445.5	8624.5	11245.5
Tmem117	21148.25	20470.5	27725	27472.5
Tmem119	7934	19682	13645	24483

Tmem120a	6847	5038	8769	7961
Tmem120b	34243	33424	24246	26637
Tmem121	35726	37422	40846	3008
Tmem123	4208.5	23848	19886	39335
Tmem125	24913	26264	27546	28075
Tmem126a	22323	35894	29991	34446
Tmem126b	9012	12043	12555	13023
Tmem127	16531.5	13454.5	18107.5	16922.5
Tmem128	35214.5	21851.5	17678.5	17758.5
Tmem129	21578	20764	19421	19943
Tmem130	16635	20228	25117	27210
Tmem131	14870.5	11310	11775.5	11090.5
Tmem132a	11356	10705	16828	14813
Tmem132b	27565.5	33512	18573.5	18290
Tmem132c	32904	34602	36296	34600
Tmem132d	28836	30238	32931	31325
Tmem132e	41022	13290	36072	11606
Tmem134	18389.5	18646.5	19549.5	19433.5
Tmem135	23896.25	14622.75	22263	24575.5
Tmem138	17932	20419.66667	22396	23805.66667
Tmem139	19876	23164	19921	22033
Tmem140	21707	22959	27070.5	25164
Tmem141	9247	2505	11950	7134
Tmem143	14465	9847	6692	4173
Tmem144	23758.33333	23730.33333	33191.33333	23693
Tmem145	6642	13906	14048	19879
Tmem147	34388	32413	28994	33620
Tmem14a	28141	8555.5	8872.5	24794.5
Tmem14c	7713	12435	9182	13231
Tmem150a	30537	38350	24057	34754
Tmem150b	11670.5	17884	3765.5	39053
Tmem150c	16371	18799.5	34731	17921
Tmem151a	28334	35091	35420	37160
Tmem154	12369	417	4820	26161
Tmem156	24212	25405	33703	29477
Tmem158	21490	16878	24738	19706
Tmem159	26825	27412	27221	26764
Tmem160	14581	18663	16683	15311
Tmem161a	18963.5	17931.5	19214.5	18259

Tmem161b	23827	6058	24491.33333	8542.666667
Tmem163	13383	10135	20313	20097
Tmem164	34719.5	25209.5	24909.5	23191
Tmem165	29938.5	20013	24048.5	21745.5
Tmem167	14528	24438.5	10569	20799.75
Tmem167b	16757	17812	19514	19086
Tmem168	22898.5	22871.5	22024.5	22770
Tmem169	31440	33118	34878	33190
Tmem17	27060	28390.5	10106.5	7825
Tmem170	40397	1786	4475	6340
Tmem171	22269	17181	28533	18560
Tmem173	22486	23229	17759	15527
Tmem174	28985	38401	37760	86
Tmem175	8447.5	24184	6655.5	23640
Tmem176a	937	15498	38033	9797.5
Tmem176b	23764	5560	21126	36271
Tmem177	21721	27028	17708	22572
Tmem178	28075	39628	23925	24714
Tmem179	29089	26273	34006	36180
Tmem18	23083.5	18703.5	19837.5	17492
Tmem180	27988	29629	23622	24026
Tmem181a	28713	22614	22346	22352
Tmem182	38125	2537	3544	10834
Tmem183a	10898	14509.5	10189.5	12154.5
Tmem184a	6119	8312	15467	15132
Tmem184b	36368	29368	34011	35554
Tmem184c	36400	33850	35284	34221
Tmem185b	11541	13598	7906	8897
Tmem186	20806.75	23151.25	22303.5	23516.25
Tmem189	12217	9633.5	11467	11577
Tmem19	40558	26813	37453	28918
Tmem190	9578	9219	17402	19475
Tmem191c	27639.5	22646	26453.5	20354.5
Tmem192	20290	18113	15398.5	16045
Tmem194	12573	10686	12640.5	11728
Tmem194b	18325	12076	17328.5	12882.5
Tmem198	11565	13280	16209.5	17387
Tmem198b	27444.5	25033.5	26224	23985
Tmem199	11834.5	10952	23509.5	29992

Tmem2	21308	19906	37344.5	18267.5
Tmem200a	10512	14920.5	15248.5	20325
Tmem201	19706	14899	16555	15796
Tmem202	26835.5	28387.5	12463.5	27704.5
Tmem203	19233	20141	19889	19506
Tmem204	23926	29233	26363.5	26828
Tmem205	15350	17634	20025.5	37700
Tmem206	10448	22120.5	5728.5	19612.5
Tmem207	30563	32108	33647	32009
Tmem208	6737	12363	8697	9466
Tmem209	23063.5	24985.5	21831	25477.75
Tmem210	24018.5	25464.5	32210.5	31616.5
Tmem212	8394	16528	19103	22600
Tmem213	39161	934	2995	11099
Tmem214	21302	19537.5	18952	18420.5
Tmem215	28847	27374	699	38681
Tmem216	18944.5	14031	32257	19192
Tmem217	12065	16716	16408	16382
Tmem218	11819	13756	11275	10286
Tmem219	11803	13014	9762	12288.5
Tmem220	11305	11972	9755	12006
Tmem221	10819	16629	10324	14932
Tmem222	23134	21815.5	28319.5	10446
Tmem225	20952	22649	27458	29852
Tmem229a	16878.5	19775.5	22234	26935
Tmem229b	2702	37335	40876	34159
Tmem230	41050	38685	747	40057
Tmem231	4448	8192	9864	8891
Tmem232	23902	25001	26841	25882
Tmem234	4409	1794	39456	37891
Tmem237	16187	19801	17667	18972
Tmem238	33530	39426	27000	35207
Tmem239	5154	5177	11606	10272
Tmem241	24952.66667	26042.33333	26651.66667	25981.66667
Tmem242	33987.5	34339.5	19258.5	33275
Tmem243	24952.5	12276	9087	11634
Tmem245	8756.2	11006.6	7849.2	9328
Tmem246	33242	22524	37648	34633
Tmem247	1986	7719	12967	14752

Tmem248	17041	14793.66667	28010.33333	13951.66667
Tmem25	34610	29727	39005	40050
Tmem251	8586	11389	13263	11766
Tmem252	34392	36747	499	5
Tmem254a	7186	4544	40860	36599
Tmem255a	24268.5	25081.5	31019	9992
Tmem256	3237	10780.5	10199.5	8511.5
Tmem258	11940	11890	8171	9189.5
Tmem26	8426	40085	36454	28024
Tmem260	32907.75	26755.75	24666	22543.25
Tmem261	21667	22093.5	24262.5	18453.5
Tmem263	14759	17363	7557	11693.5
Tmem27	26835	28267	29581	28611
Tmem28	23328.5	24151.5	29841.75	30664.5
Tmem29	25800	27320	21094	22090
Tmem30a	23461.5	24586	21970.5	3662
Tmem30b	18485.5	21149	27283.5	31588
Tmem30c	24941.66667	22750.66667	20810	11330.66667
Tmem33	24166.66667	14377.66667	23237.33333	13181.33333
Tmem35	17859	14498	18748	24614
Tmem37	15172.5	19198.5	11075.5	34101.5
Tmem38a	23673.5	22596.5	22630	23256.5
Tmem38b	16837	23122	13848	15872
Tmem39a	1063	3534	38068	38824
Tmem39b	34379	34287	30913	35670
Tmem40	34228	4269	21192	10500
Tmem41a	16168	14481	15262	12915
Tmem41b	22083.66667	25150.66667	17233.66667	20601.66667
Tmem42	19665.5	22815	20457.5	21094
Tmem43	6988	4861	3169	8096
Tmem44	6634.75	17190.75	19343.25	19669.25
Tmem45a	36567	40010	1521	4969
Tmem45a2	32451.5	34476.5	19413	35698.5
Tmem45b	33009	34741	36061	1939
Tmem47	30158	31729	32725	35291
Tmem5	18629	29400	19785	28933
Tmem50a	36620	37669	40232	38904
Tmem50b	20565.5	20691	32658.5	37063
Tmem51	16255	12746	15617	11921

Tmem52	16567	21718	22815	23252
Tmem52b	6501	3496	9528	2889
Tmem53	11886	11850	11755	10671
Tmem54	10829	9203	12434	8657
Tmem55a	28462	22378	21953	18892
Tmem55b	38006	34934	24709	25697
Tmem56	24405.5	25844.5	28601	29575
Tmem57	10784.75	20755.25	19124.5	21470.75
Tmem59	14454.33333	15568	14945.33333	15934
Tmem59l	20545	21425	29600	29220
Tmem60	30603	35478	29373	35509
Tmem62	19715	22206.5	14672.5	21308
Tmem63a	25497	24162	23330	27283
Tmem63b	4424	10225	12973	14383
Tmem63c	25767.5	22707	25679.5	22631
Tmem64	30096	19099	20894.5	18359
Tmem65	16941.5	20179	24712.5	18684
Tmem67	27110	25590.33333	29003.66667	30565.33333
Tmem68	21672	21837	19627	20170
Tmem69	21123	29421	20583	12517
Tmem70	17556	18438	15084	17570
Tmem71	39303	24114.5	35616	23650.5
Tmem72	26191	8122	30183	13033
Tmem74	34014.5	31019.5	20684	33388
Tmem79	23620	25040	29174	30513
Tmem8	25278.5	17521	19192	17437
Tmem80	6654	4423	7009	6832
Tmem81	36292	35260	40918	35024
Tmem82	2164	522	3541	40463
Tmem86a	39385	28656	27925	24584
Tmem86b	15833.66667	17352.66667	12231.33333	13951.33333
Tmem87a	8841.5	7930	8813	8727.5
Tmem87b	26196.5	16892.5	18537.5	18795.5
Tmem88	33324	27366	27837	33992
Tmem88b	26629	37070	37828	28946
Tmem89	5229	12393	10440	10212
Tmem8b	33211	34938	2425	1211
Tmem8c	22692	23344	28345	31304
Tmem9	25546.5	27406.5	25012.5	25690

Tmem91	28601	27261	23706	26696
Tmem97	19316	24833	17945	21806
Tmem98	34527	22907	32503	37046
Tmem9b	17325	17085	14916	15181
Tmf1	40727	2314	40229	40663
Tmie	36219	24205	2103	32833
Tmigd1	16953	35166	38636	35131
Tmlhe	22857.33333	9380	11175.66667	22913.66667
Tmod1	27097.5	20364	29250	27780.5
Tmod2	29582.66667	34073.66667	24398	24756.33333
Tmod3	14727.25	11847.5	15896.75	18117.5
Tmod4	9400	8536	4406	8650
Tmpo	32591	32983.75	34200.25	32325
Tmprss11a	10029	13439	30020	34625
Tmprss11b	32727	34083	35799	33902
Tmprss11d	32726	34341	35552	33710
Tmprss11e	30136	31673	32630	31243
Tmprss11f	27913.5	37071	30659.5	34730.5
Tmprss11g	25356.5	28701	27112.5	26256.5
Tmprss12	27606	29043	30431	29459
Tmprss13	4513	9051	10437	14493
Tmprss15	22179	10154	37272.5	38597
Tmprss2	35868.5	1897.5	6795	8405.5
Tmprss3	33785	39189	1568	2843
Tmprss4	23021	10619	25188	1857
Tmprss5	28547	30079	31449	30328
Tmprss6	4589	2774	11165	9961
Tmprss7	22038	21656	23595	21425
Tmsb10	25645.5	29352	13967	11664
Tmsb15a	16385	19386	25267	23074
Tmsb15l	20036	24767	23181	21865
Tmsb4x	9613	10532	12928	14879
Tmtc1	32166	33939	35805	34101
Tmtc2	27889.57143	24679.28571	26619.28571	14222.71429
Tmtc3	31207	31695.5	33404.5	32276
Tmtc4	17849.5	35709	31105	24660
Tmub1	7872	6030	543	728
Tmub2	13092.5	11945	11605.5	11063.5
Tmx1	35932.5	19667	17423.5	22275.5

Tmx2	14975	15414	31478	31740.5
Tmx3	24651.5	34176.75	22218.75	31252.25
Tmx4	27998	22306.75	23174.5	21307.75
Tnc	22786.8	21516.8	14450.6	16316
Tnf	7435	25155	5470	22269
Tnfaip1	3696	5045	7114	8110
Tnfaip2	26312	23466	24689	25119
Tnfaip3	39781	9206	40496	12048
Tnfaip6	23474	38668	8542	18711
Tnfaip8	3033.5	15125.5	35862.5	34158
Tnfaip8l1	37554	9339	33727	3899
Tnfaip8l2	5330	36409	39358	30042
Tnfaip8l3	31736	29567.5	40575	22690
Tnfrsf10b	19883.66667	31359.66667	24176.33333	31701.33333
Tnfrsf11a	30342	23224	10159	17963
Tnfrsf11b	32776	38588	2787	4112
Tnfrsf12a	30170	31762	4295	39011
Tnfrsf13b	18325.5	12379	12654.5	10560.5
Tnfrsf13c	1648	13734	8344	5257
Tnfrsf14	13638.5	30803.5	9225.5	24339
Tnfrsf17	39430	11030	34417	11439
Tnfrsf18	40142	39597	1224	40075
Tnfrsf19	24092	21405.66667	25262	10992.33333
Tnfrsf1a	13128	6152	10027	9995
Tnfrsf1b	25152	28613	21599	28676
Tnfrsf21	23830.66667	15418	19543.33333	16213.66667
Tnfrsf22	13806	14954	18198	19293
Tnfrsf23	20918	20290	25233	25769
Tnfrsf25	14194.5	13912	21210	20249.5
Tnfrsf26	12552	14053	14320	15425
Tnfrsf4	28736	25671	33859	34149
Tnfrsf8	24095	23667	26218	23328
Tnfrsf9	24548.75	28676.25	30725.75	35413.75
Tnfsf10	2906	3671	7867	9823
Tnfsf11	24995	26060.5	28485.5	14824.5
Tnfsf12	30938	22713	40059	28152
Tnfsf13	18248	12975	25281	16983
Tnfsf13b	22479.5	15534.5	21786	13347.5
Tnfsf14	25084	27870	30002	32715

Tnfsf15	38632	10141	6286	9152
Tnfsf18	21616	11902	1001	18491
Tnfsf4	36789	4474	12125	12753
Tnfsf8	23854	21567	25836	31804
Tnfsf9	23589	5902	29000	9713
Tnfsg5	34741	37637.5	20963.5	21129.5
Tnik	20945.2	20413.4	21209.8	14706
Tnip1	5776	10298	5112	14053
Tnip2	34792	30293	31613	32515
Tnip3	5101	29489	10801	24099
Tnk1	22939	7509	10211	19559
Tnk2	6026	9989	6740	12794
Tnks	18105	10333.66667	11671	9117
Tnks1bp1	18387.5	18083	18909.5	17650.5
Tnks2	24350.66667	18057.33333	21353.66667	19527.66667
Tnmd	33191	34925	36728	34963
Tnn	32661	34239	36683	34762
Tnnc1	25667	34543	38781	2813
Tnnc2	3649	5612	12689	12485
Tnni1	16339.5	16233	19541	20227
Tnni2	25229	17779	24506	11715
Tnni3	4743.5	9066	7964.5	12426
Tnni3k	4241	6522	4712	6553
Tnnt1	13887	16893	19935	17759
Tnnt2	30487	11890	15648.5	20698.5
Tnnt3	14095.5	12550.5	22014.5	15637
Tnp1	11914	14641	18116	18006
Tnp2	36398	37060	398	40487
Tnpo1	36259	30884.5	34353.5	32191.5
Tnpo2	2851	4248	3718.5	5436.5
Tnpo3	17471.83333	22278.33333	30463	23231.83333
Tnr	1093	32558	38947	5124
Tnrc18	10401	165	33777	22259
Tnrc6a	8686.666667	15541.66667	19153	18803.33333
Tnrc6b	20965.57143	19176.14286	17887.57143	24696.57143
Tnrc6c	6921	1717	7231	2647
Tns1	17132.75	21049.25	20977.25	15414
Tns2	18104	15276	19456	11993
Tns3	14444.66667	7038.333333	12179	8885.666667

Tns4	35207	38788	32412	34530
Tnxb	7955	6874	20080	26921
Tob1	9933	8373	7871	2345
Tob2	13124.66667	21344.66667	21655.66667	18282
Toe1	20322.66667	21956.33333	19353.33333	21028
Tollip	9134.666667	8716.666667	9612.333333	11514
Tom1	20150.5	21100.5	20683	21352
Tom1l1	25828	23777.5	16992	28796.5
Tom1l2	9129.666667	7084.666667	7849.333333	8554
Tomm20	18430	24594	21854	22570
Tomm20l	395	40775	35804	20108
Tomm22	7196	11167	12325	13343
Tomm34	24744.5	23410.5	21554.5	23372
Tomm40	11080	12119	32286.5	16655.5
Tomm40l	8799	8408	9787	8605
Tomm6	22520.5	35596	13974.5	31397.5
Tomm7	9713	9575	9796	8589
Tomm70a	15528.75	15400.75	17002	16335.25
Tonsl	23916.66667	33035.33333	24625.33333	31423
Top1	18113	14790.5	18559	19298.5
Top1mt	1620	8124	5930	10836
Top2a	14961.66667	23120.33333	37363.66667	25947
Top2b	15262.5	14600	5556.5	3351
Top3a	17387	16576	16089	17987
Top3b	32157	29116	31692	34038
Topbp1	9128	6154	9083	4147
Topors	14992	13648	31445	32291.5
Tor1a	8736	11266	6236	8321
Tor1aip1	17126	19752	17635	17627
Tor1aip2	19809	19731.8	19311	22695.4
Tor1b	5382	37506	24868	31719
Tor2a	39168	38830.5	36078.5	36291.5
Tor3a	9535	14397	8071	15046
Tor4a	30157	29754	25980	25349
Tox	25678.66667	26879	28703.66667	32688
Tox2	8900	34627	11530	13649
Tox3	25487	27020	27819	39544
Tox4	13608.5	11517.5	13008.5	13599
Tpbg	14451.33333	16864	25640.66667	27612

Tpbpa	30214	31747	40055	32047
Tpbpb	38807	18720	6827	29918
Tpcn1	18966.33333	17771.33333	14248	26811.66667
Tpcn2	14138.5	24510.5	27162	25538
Tpd52	21795.5	14754.5	20804.5	16535
Tpd52l1	29164	4717	31511	409
Tpd52l2	29801	36416	34437	37040
Tpgs1	37327	41098	25730	29650
Tpgs2	19365	14501.5	36928	20587
Tph1	23433.5	25660.5	30826	31497.5
Tph2	24303	25447	27343	27623
Tpi1	22599	15274.5	15611	19379
Tpk1	10601	29520	23191.5	28146.5
Tpm1	20276	14775.66667	17046.66667	22319
Tpm2	10692.5	5436	25518	22219.5
Tpm3	19809.66667	13052.33333	16254.66667	16444
Tpm4	30949	36810	37419	708
Tpmt	18817	30579	22393	25644
Tpo	7185	12264	41061	4571
Tpp1	22379	31374.5	33678.5	30687
Tpp2	18935.33333	26642.33333	27464.33333	28627
Tppp	38894	4152	1623	5734
Tppp2	30826	32502	2161	32358
Tppp3	19577	12991	15437	22774
Tpr	13337	29832.5	28267	24847.5
Tpra1	35893	23027	23500	20941
Tprg	33850	18229	25728	8987
Tprgl	39725	36381	37670	36051
Tprkb	34090	34871.5	29062	36432
Tprn	2179	3688	6519	3478
Tpsab1	27270	35988	9252	39393
Tpsb2	21652	22010	30014	26852
Tpsg1	18515	22728	21707	20663
Tpst1	21171	20124	22970	20325
Tpst2	27890	21132.5	23098.5	19504
Tpt1	37003	29933	2427	39435
Tpte	13493	17333	19701	21671
Tpx2	32138	31787	36528	35149
Tra2a	24090	20990.66667	29508.33333	31171.66667

Trabd	20376	22488.5	19888.5	20259
Tradd	4727	6496	8346	8254
Traf1	828	36058	5903	41139
Traf2	18131	29529	21466	34599
Traf3	14349	22172	13031	22101
Traf3ip1	23624	21821.5	23417.5	22559
Traf3ip2	19633	15922	18077	14552
Traf3ip3	11609	8975	7447	38598
Traf4	34473.5	35683	20648.5	24008
Traf5	22722	37841	20807	37958
Traf6	23098.33333	20381.66667	18363	21046.33333
Traf7	17974	13400	13337	13020
Trafd1	13282	11024.5	8261	8130
Traip	15399	12860	14204	15326
Trak1	13732.33333	12703.33333	13250	11776.66667
Trak2	23819	18219.5	14553.5	15442.5
Tram1	28843.33333	31993.33333	30873.66667	29607.33333
Tram1l1	33980	26195	3594	5330
Tram2	13718	15308	15784	16440
Trank1	6942	11238	13236	13660
Trap1	29623.5	10371	6950.5	9689
Trap1a	10854	9485	17755	16309
Trappc1	40806	1922	37680	38031
Trappc10	10336	13805	15972.5	16551
Trappc11	26460.5	6464.5	26796.5	7938
Trappc12	18013.2	21384.8	14066.4	21453
Trappc13	21052.5	23431.5	17263.5	18609.5
Trappc2	9019	12646	9109	10238
Trappc2l	6004	12976	11109	12222
Trappc3	5291.5	9208	5196	8186
Trappc4	10470	11732	10393.5	8567
Trappc5	21901	25133.5	21129.5	26503.5
Trappc6a	35215	33532.5	33803	35670.5
Trappc6b	25325.33333	28166.66667	11686.33333	12079
Trappc8	30177.33333	27991.66667	28203	29746.66667
Trappc9	16592	17280.66667	18030	18162
Trat1	11758	8441.5	12586	29139
Trav1	8524	5277	10831	13959
Trav14d-3-dv8	31400	26297	34039	9894

Trav3-4	7800	31203	9683	4778
Trav5-4	13523	16462	18502	18138
Trav9n-4	25157	29685	39644	27238
Trbv1	14734	9680	16102	17077
Trbv14	18243.75	19766	22063.25	20330.25
Trdmt1	21419	28203	19357	23808
Trdn	29942.5	23774.75	25302.75	26643.5
Trdv2-2	28823	37003	37490	33247
Treh	28430	30321	30428	32914
Trem1	17610	22081	7319	4299.5
Trem2	9600	18477.5	11015	23787
Trem3	33075	22192	330	20763
Treml1	14994	40957	15893	4718
Treml4	16133	24000	19733	26714
Trerf1	11343.75	21772.25	11564.25	21748.75
Trex1	13228	24309	10515	25574
Trex2	34027	2420	2784	9088
Trf	19527	2650	11920	38513
Trh	39684	4868	2997	40586
Trhde	31911.33333	34273	35528.33333	34313.33333
Trhr	20891.5	25304.5	17271	17428.5
Trhr2	32303	35972	33304	32932
Triap1	2072	39806	40488	39194
Trib1	26766	16349	23139	19717
Trib2	7929	6822	9613.5	12981
Trib3	13756.5	16378.5	18087.5	20586
Tril	20628.33333	21940.66667	25483.33333	24311
Trim10	22636	23137	30599	33119
Trim11	17283.5	16092.5	15278	14679
Trim12a	34478	19425	34718	20150.5
Trim12c	35440	37100	27299	40184
Trim13	11796	23285	12976.5	27213
Trim14	27092	23464	24185	23163
Trim15	4419	38801	31170	4012
Trim16	19277.33333	14826.66667	15643.66667	12964.33333
Trim17	35177	36332	4202	1971
Trim2	21661	24377.66667	24540.33333	16232.33333
Trim21	722	3588	39232	3093
Trim23	15903	19227.25	18236.5	22055.75

Trim24	27540.66667	25867	29206	27916
Trim25	21817	16402.66667	21104.66667	18083
Trim26	20109	18559.66667	19200.66667	29706.66667
Trim27	10812	13548	10880.5	31191
Trim28	27129	22401	23555	22332
Trim29	2235	8199	35963	1943
Trim3	20623.5	20199	19267.5	20695.5
Trim30a	18305.5	22340.5	14861.5	21617
Trim30b	19437	18832	23055	17122
Trim30d	12548	17271	14591	22668
Trim31	9763	4386	8153	7465
Trim32	8006	28123	28097	27313.5
Trim33	34321.5	32965.5	30393	25100.5
Trim34a	12203.66667	20094.33333	16479	23094
Trim35	25908.66667	21664.33333	18360.66667	18059
Trim36	22693	14492.5	12172	9940
Trim37	18159	16388	9059.666667	7918.666667
Trim39	22443.25	25280.5	25642.5	25781.75
Trim41	18006	11427.5	14789	11224
Trim42	28782	30066	35521	2709
Trim44	26598.33333	28396	22730.66667	23972
Trim45	22674.5	23017	27339.5	28062
Trim46	10504	12869	15128	14840
Trim47	34503	23897	38020	28071
Trim5	21421	23769	25294	29695
Trim50	9173	12769	39947	8969
Trim52	21973.5	34426	34750	33697.5
Trim54	34645	29843	38546	39108
Trim55	22995.5	27234.5	22074	35875.5
Trim56	13564	12832	11273	12279
Trim59	5952	20443.5	16061.5	20709
Trim6	11999.8	17509.2	9207.4	16725.2
Trim60	19554	23888	28383	28498
Trim61	25441	26628	28907	27630
Trim62	16070	15945	18168.5	17961
Trim63	18325.5	19937.5	19326.5	18917.5
Trim65	23055	17714.5	20041	22410.5
Trim66	24527.66667	28074.66667	29487.66667	26830.33333
Trim67	31520.5	34430	3726	21261.5

Trim68	12470	28782	6783	9996
Trim69	16666	14566	19720	23766
Trim7	23507.66667	12733.33333	14120	17053.66667
Trim8	13959.33333	28044	13262.66667	14564
Trim9	26392	14589.75	19319.75	15334
Triml1	23433	29216.5	25677.5	27719.5
Trio	26891.5	20485.5	25054.75	23420.25
Triobp	7216	7909	4115	2528
Trip10	14043	17688	15892.5	22130.5
Trip11	14890.85714	22281.42857	17241.71429	16051.71429
Trip12	13576	22008.25	9704.25	19132.75
Trip13	5215	39650	2246	31244
Trip4	12720	18043.6	18927.6	12814.6
Trip6	10193.5	11540	17032	18438
Triqk	38780	32630	34072	20337
Trit1	36660	40191	39523	41064
Trmt1	25430	19757	17023.5	16856
Trmt10a	8546	16103.5	14365	21174
Trmt10b	3921	6014	7507	7520
Trmt10c	10810	15275	15078	17657
Trmt11	37599.5	17702.5	40186	39695
Trmt112	2892	2464	1019	3321
Trmt12	6630	25327.5	17770	18633.5
Trmt13	36422	36707	38879	39863
Trmt1l	40839	40993	2788	2121
Trmt2a	6143.5	5026	3525.5	6914.5
Trmt2b	23664	22806.5	13940.5	13957.5
Trmt44	18422	13267.5	27590	13433.5
Trmt5	5421	7085.5	6787.5	8445.5
Trmt6	22852.5	21622	17598	17495
Trmt61a	8557	8741	11514	14595
Trmt61b	22146	28930	25977	28430
Trmu	20664.33333	14019	23339.66667	14368.66667
Trnau1ap	20278.5	22667.5	22867.5	20093.5
Trnp1	1232	2117	6522	1004
Trnt1	22428.66667	25008	22497.33333	23884.66667
Tro	9398.25	13104	14094.25	25381.75
Troap	14507.5	13595	15577.5	16659
Trove2	12088	19428	8351.333333	7544.666667

Trp53	20384	18599	16245	21337
Trp53bp1	4496	25800	3897.333333	12750.33333
Trp53bp2	5140.5	19310	21299.5	19826
Trp53i11	40602	4940	36014	359
Trp53i13	27241	22396	25158	25488
Trp53inp1	13530	24366.5	21893.5	20292.5
Trp53inp2	7301	1271	3962	1265
Trp53rkb	9160.333333	15802.66667	23327	28396.33333
Trp53tg5	39428	1907	40607	6335
Trp63	33850.5	34840.5	35218	34516.75
Trp73	28597	28489.5	31981	31049
Trpa1	17468	18911	23727	19405
Trpc1	22001.33333	23296.66667	26959	26273.33333
Trpc2	27182	22877	23901	24127.5
Trpc3	31865	38312	9607	13606
Trpc4	2336	3713	11907.5	11699.5
Trpc4ap	19539.8	16561.4	21823.6	22571.6
Trpc5	33618	31526.5	33237	37058.5
Trpc6	23242	21098.5	21744.5	21796.5
Trpc7	29022	30431	33089	31467
Trpd52l3	30083	31592	33493	31874
Trpm1	10773	4488.5	35329.5	21666.5
Trpm2	31469	23587	12870	9544
Trpm3	20744.14286	26725.71429	24123.57143	26357.71429
Trpm4	15728	13274.5	18206.5	21104
Trpm5	1075	703	16831	13283
Trpm6	19197.5	36394	23245.5	19744
Trpm7	22071	17736	19258	18473
Trpm8	23575	24661	35088	25355
Trps1	7576	18877.25	24235	7732.5
Trpt1	12419	16150	16259	16895
Trpv2	12001	9413	10808	9523
Trpv3	22950	23315.5	24842.5	24535
Trpv4	19751	14887	19818	16249
Trpv5	33592	33591	28197	35597
Trpv6	3238	37252	9245	144
Trrap	24251.25	14242	23691.25	21830.75
Trub1	16983.66667	15893.66667	14034	14733
Trub2	10944.5	8965.5	6106	8017

Try5	7164	11762	9221	11088
Tsacc	12064	12261	13806.5	12555
Tsc1	19733.75	14453	16181.5	15846.25
Tsc2	4890	789	1822	245
Tsc22d1	13790	11500	8637	1845
Tsc22d2	5260	2723.5	21395	4287
Tsc22d3	4641.333333	15699	9858.333333	19072.66667
Tsc22d4	12130.25	27856	26396	23779.75
Tsen15	22574	29070	28261	28605
Tsen2	13336	10538.66667	12534	15345.66667
Tsen34	2298	3944	5307	3855
Tsen54	35020	36806	36767	36901
Tsfm	13483	17268	14575	17413
Tsg101	22629.5	27017.75	20853.25	21471.75
Tsga10	5152	3427	13420	11611
Tsga10ip	18271	28217	21953	21486
Tsga13	31720	37947	38612	3722
Tsga8	11412	12517	20283	17379
Tshb	25432	24326.5	28662.5	27097
Tshr	20939	16484	6675.5	8479.5
Tshz1	21573	31337.5	19150	33199.5
Tshz2	30278	20032.25	30869	22425
Tshz3	5654	1480	16715	19748
Tsix	30287	32224	38712	31362
Tsks	4706	7281	7946	6262
Tsku	15848.5	20794.5	25278.5	24651
Tslp	36868	34356	35446	33970
Tsn	13661.25	22500.75	19315.25	17714.5
Tsnax	24225	23465	21573	23833
Tsnaxip1	2433	3008	35004	40139
Tspan1	21212	25651.5	8683.5	11224.5
Tspan10	3189	20206	15702	15150
Tspan11	10290	28812.5	31980	28361.5
Tspan12	5697	9625	39926	31470
Tspan13	2856	34680	3642	37098
Tspan14	16770	8972	13750	8713
Tspan15	33581	224	39319	3635
Tspan17	15660	13728	18451	14901
Tspan18	21288.5	15776	17525.75	21589.25

Tspan2	559.5	2261.5	17489	2314
Tspan3	2033	6350.5	21102.5	3856.5
Tspan31	13473	13371	15118	15457
Tspan32	16873	22958	14882.5	37718
Tspan33	11773.5	22464.5	4083.5	26796.5
Tspan4	31812	21862	25897	22072
Tspan5	39134	32075	27842	24991
Tspan6	38393.5	23220	6743	8792.5
Tspan7	35804	20742	3972	33409
Tspan8	21592	21059	23394	24976
Tspan9	22255	22520	26170.5	25304.5
Tspo	10730.5	14296.5	9302.5	12268
Tspo2	40844	1208	7260	8347
Tspyl1	40609	40093	2261	2906
Tspyl2	15441	16108	16222.5	16324.5
Tspyl3	12014	10549	8276	6128
Tspyl4	29936.66667	23911	29981	24792.66667
Tspyl5	25755	12705.66667	19747.66667	15068
Tsr1	2982	2711.5	40195	2262
Tsr2	11393	8604	11085	10403
Tsr3	17082	16721	16461	19301
Tssc1	7404	24986.66667	16581	7130.666667
Tssc4	32823	36036	36541	35657
Tssk1	27481.5	28285	16009	14831.5
Tssk2	6721	17276	12632	22909
Tssk3	6607	15225	13185	18873
Tssk4	22918.5	23521.5	32219	33780.5
Tssk5	14244	16713	19455	19142
Tssk6	19792	18114	22158	21303
Tst	34895	528	629	2622
Tsta3	14119	14932	11962	13967
Tstd2	17037	19406	17357	18999
Tsx	34587	33084	35339	33344
Ttbk1	12793	9898	18484	12216
Ttbk2	23027.25	20402.75	21784	21459
Ttc1	24528.5	9923	26980	10669
Ttc12	18958.5	25404.5	21359.5	9909
Ttc13	14655	15285	10605	12376
Ttc14	18053	17578.66667	29691.33333	30990.66667

Ttc16	10153	10081	9737	8323
Ttc17	16779	8291.666667	9018.666667	8215
Ttc19	9180	9894.666667	7311.333333	11398.66667
Ttc21a	7611	7524	18963	21643
Ttc21b	13621.66667	28372	30935.66667	26033.66667
Ttc22	8687	12384	15261	15271
Ttc23	29656.66667	32894.33333	33757	20452.66667
Ttc23l	28793	30401	31171	29826
Ttc24	8063	8202	7220	7171
Ttc25	23674	23330	24664	27874
Ttc26	39111	22138.25	20156.75	11993
Ttc27	7933	9303	4801	4952
Ttc28	28151	28035.4	20278.4	26919.8
Ttc29	27977	29492	36341.5	39424.5
Ttc3	12659	10599	32131	36501
Ttc30a1	29257	30234.5	29762	28388.5
Ttc30b	27790.5	24114.5	23781	22336
Ttc32	11124	15931	9556	9416
Ttc33	29013	33573	27725	32183
Ttc34	5898	8518	10538.5	8360.5
Ttc36	539	1854	16304	8179
Ttc37	11988	27364.5	27074	28314.5
Ttc38	17969	16848	14364	12506
Ttc39a	27024	20582	36721	23643
Ttc39b	35563	30101.33333	31884.66667	30990.66667
Ttc39c	24034	11351	16049.66667	10973
Ttc39d	24764	32340	28212	35540
Ttc4	35709	30412	25440	28162
Ttc5	15738	17467	19812.5	18366.5
Ttc6	25581	36166	39529	35227
Ttc7	14578.5	13280.5	11753	9416.5
Ttc7b	39174	36956	36635	31664
Ttc8	17316	16794	15401	14906
Ttc9	30745.5	37319	36932.5	18907.5
Ttc9b	4891	8232	3489	7494
Ttc9c	37831	37949	30618	32430
Ttf1	29293.5	26914.5	21020	19018.5
Ttf2	26185	25712.5	21998	24839.5
Tti1	37753	37069	33013	31394

Tti2	36616	37954	39767.5	39807.5
Ttk	621	6689	14335	11553
Ttl	13088.5	20897	15529	14612
Ttll1	22081.5	22081	23260.5	25219
Ttll10	32640.5	29125	32566	29370
Ttll11	33620	25286	37346	37223
Ttll12	7918	5697	5697	4696
Ttll13	28372	17834	28940.5	25522.5
Ttll3	11142.5	5672	4345	21216
Ttll4	24362	12259.5	15993.75	19778.75
Ttll5	22228.57143	21536.85714	21299.85714	17062.42857
Ttll6	29819	30479	39777	289
Ttll7	5329	14670.33333	15782	17440.33333
Ttll8	8753	13088	12684	11353
Ttll9	20772	18590	15571	18409
Ttn	34086.09091	31534.45455	27285	27048
Ttpa	22932	18832	28161	25841.5
Ttpal	15822.66667	17049.66667	15260.66667	18452.66667
Ttr	32180	33527	38243	34582
Ttyh1	15900.4	17313.4	23919.6	21999.2
Ttyh2	31860	24724	18237	17694
Ttyh3	7493	39085	7629	36196
Tub	23088.66667	28473.66667	25798	26862.33333
Tuba1a	5443	11289	9863	12211
Tuba1b	8643	12450	11882	14761
Tuba1c	4051	4679	4633	9365
Tuba3a	24678	8283.5	6441.5	10152.5
Tuba4a	21662	26038.66667	18531.33333	27560
Tuba8	15995	17130	22629	16854
Tuba-rs1	33268	37432	33764	38570
Tubb2a	27521.33333	19835.66667	25725.66667	26042.33333
Tubb2b	8002	12404	11548	16580
Tubb3	24217	22087	4391	40316
Tubb4a	17517	19689	15766	19693
Tubb4b	16636	22477	18725	24070
Tubb5	21363.5	22523	22965.5	27827
Tubb6	19114	17692.5	19715.5	21616
Tubd1	9306	8706	9895	8426
Tube1	26743.5	26499.5	10251.5	10757

Tubg1	10541	13249	5869	14110
Tubg2	3758	9190	1523	10628
Tubgcp2	4823	3066	520	40104
Tubgcp3	14848	15857	18056	16683
Tubgcp4	23721	8244.5	16181.5	6638
Tubgcp5	6982	16131	28793.66667	4943.666667
Tubgcp6	21360.33333	20534	25056	25505
Tufm	3804	3647	2620	2664
Tuft1	5579	2985	7726	3256
Tug1	19643	7206.666667	9366.666667	13393.66667
Tulp1	30865	22366	27045	24731
Tulp2	26676	28105	36271	28623
Tulp3	30714	30376	25433	26787.5
Tulp4	2348	15093	5724.333333	5058
Tusc1	8401	5798	9765	5859
Tusc2	25142.5	25109.5	24279	22502
Tusc3	9734	11702	10397	29519.5
Tusc5	12695	16385	29468	8983
Tut1	19619	18097	19461	19751
Tvp23b	5363	7083	3880	5546
Twf1	23731.25	19370.5	21196.25	23927.25
Twf2	11669.5	10116.5	8948.5	9591
Twist1	37315	4602	16332	19729
Twist2	10450	14974	22865	22420
Twistnb	24261.5	22031.5	17975.5	19503.5
Twsg1	38868	17045.33333	24020.66667	7351.666667
Txk	25662.5	30483	21652.5	31972.5
Txlna	24341.5	21665	20095.5	17408.5
Txlnb	36265	17924	5158	17107
Txlng	13238.5	6691.5	11555	13570
Txn1	36717	37043	34199	41122
Txn2	19249.5	19424	15965.5	15171
Txndc11	25462.5	26081	23503.5	25590.5
Txndc12	24227	28015	23728	23979
Txndc15	14480.5	10833	9343	5602.5
Txndc16	18087.66667	10827.33333	12483.33333	13566
Txndc17	32225	36844	39073	34776
Txndc2	24450.5	25638.5	17428	11903.5
Txndc5	23712.5	28025	27258	27461.5

Txndc8	7932	13505	13301	12320
Txndc9	11639.66667	14954.33333	12109.66667	14360
Txnip	5998.5	37130.5	5010.5	38755
Txnl1	13868.5	21883.5	17310	24019
Txnl4a	18631.66667	19783	17436	19799
Txnl4b	21571.5	25822.5	18479.5	22561
Txnrd1	20181	38108	23775	4656
Txnrd2	20715	15422.66667	16420.33333	28006
Txnrd3	12008	14220	11610	14868
Tyk2	18417	19470	11049	11771
Tymp	11176	10882	7067	7293
Tyms	22296.5	14497.5	5730.5	13923.5
Tyms-ps	18635	23114	21273	20929
Tyr	37124	39854	6122	5366
Tyro3	15414.66667	12365	20042	19499.66667
Tyrobp	10475	7067	14725	10448
Tyrp1	35200	36819	38477	39776
Tysnd1	36330	35002	34760	35026
Tyw1	21008	21670	22955	22721
Tyw3	37422	594	40131	446
Tyw5	2177	7051	2635	4406
U2af1	19505.5	4158.5	23371	6920
U2af1l4	12407	14480	13427	15501
U2af2	19139.5	20637.5	17404	17838
U2surp	11103.33333	5960	29236.33333	30675.66667
U55872	34355	33091	23832	21782
U90926	39630	1990	8285	8358
Uaca	7314	5507.5	30595.5	11249.5
Uap1	9595	11980	11519	11752
Uap1l1	26374	21576	19680.5	21693.5
Uba1	17086	18034.33333	15215.66667	18616
Uba2	3755	3035	4799	2002
Uba3	29998	36760	31772	37573
Uba5	22417	15804	18692.5	3846.5
Uba52	12152.33333	13664.33333	15729.66667	15707.66667
Uba6	25441	23324.6	18153.6	19228
Uba7	27647	31520	23090	34126
Ubac1	30310	31059	25918	25843
Ubac2	9619.5	11898.5	23457	26776.5

Ubald1	20030	13686	19903	14066
Ubald2	19176.5	23159.5	20179.5	24891.5
Ubap1	34842.5	10934.5	30314	14356
Ubap2	18994	24823.5	11643	16002.5
Ubap2l	20625.5	28445.33333	16062.83333	27083.66667
Ubash3a	15681.5	17166	18828.5	16566.5
Ubash3b	609	36382	39656	37201
Ubb	7543	9812	10341	14165
Ubc	21726	18884	5303	23738
Ubd	4140	14416	10351	26247
Ube1y1	29471	30940	32711	31151
Ube2a	29578	12087	29661.5	30228.5
Ube2b	22921.2	16916.4	20582.2	19565.2
Ube2c	16571.5	20578	20461	23833.5
Ube2cbp	33832.5	28950.5	28342.5	28436.5
Ube2d1	21179	20542	19883.5	20284.5
Ube2d2a	25979.33333	25362	22397.33333	24318
Ube2d2b	20863	28328	29541	29163
Ube2d3	7254	16204.2	13353.8	16318.2
Ube2d4	19167	30084	29969	35671
Ube2dnl1	31521	32871	36025	33813
Ube2dnl2	1581	6557	7700	7348
Ube2e1	36657	40413	431	40206
Ube2e2	26862	18526.33333	18662.66667	24418
Ube2e3	19557.75	22462	24546	22300.25
Ube2f	25878	20866	25073.33333	23068.66667
Ube2g1	18296.66667	20117.33333	17130	20948.66667
Ube2g2	16790.33333	4532	16383.66667	17127.33333
Ube2h	15277.25	16024.75	17411	15272.25
Ube2i	18426.14286	12869.57143	18287.42857	17799.71429
Ube2j1	34857	53	35219	36555
Ube2j2	30766.5	25228	26100	25881.25
Ube2k	12183.2	17760	15106.4	18553
Ube2l3	25903.5	28775.5	21784.5	26046.5
Ube2l6	40945	12697	39111	10110
Ube2m	21508.5	22738	21575.5	3764
Ube2n	25933.66667	13368.33333	27410.33333	24820.33333
Ube2o	8949.5	27326.5	23233.5	23883.5
Ube2q1	13981.5	34356	33296	34206.5

Ube2q2	25446.2	25961.4	20365	12447.6
Ube2ql1	30067	31625	32647	807
Ube2r2	17934.4	15297.2	22973.6	23567.2
Ube2s	6464	9703.5	7631.5	10517
Ube2t	30037.5	29133.5	11866	12325
Ube2u	28662	24208	28416	39885
Ube2v1	26099.75	24295	22873.5	23821
Ube2v2	17140.25	16373.75	14682	14839.5
Ube2w	27205.75	25130	23093.75	25665
Ube2z	25279.5	25025.5	23391.5	24824
Ube3a	23774.4	22913.8	24330.6	15702
Ube3b	5856	976	1690	39377
Ube3c	4622	3997	1697.5	4351
Ube4a	9649	5772.666667	18041.66667	6373.333333
Ube4b	22225.5	4075.5	2370.5	3567.5
Ubfd1	9670	11082.5	12511	14710.25
Ubiad1	15513	16240	16190	16189
Ubl3	10778	10673	10347.5	9710.5
Ubl4a	12988.5	15916.5	13081.5	13824.5
Ubl4b	23913	37826	32369	37666
Ubl5	9487	7594	19535	8922
Ubl7	16751.5	18465	18632	18896.5
Ublcp1	24738	34462	29580	36541
Ubn1	23971.33333	21801	19415.33333	30360.33333
Ubn2	9186.333333	27882.33333	18283.33333	18850.33333
Ubox5	5604.5	4392.5	3684	4783
Ubp1	28679	33696.5	32488.5	35972
Ubqln1	20428.5	24622	21151	24966.5
Ubqln2	5243	35571	36	1437
Ubqln3	37207	35858	38053	33722
Ubqln4	38236	36968	34697	31655
Ubqlnl	35468	37705	2540	40283
Ubr1	25519.71429	24319.28571	24044.14286	25510.71429
Ubr2	16352.16667	24715.33333	23254	22036.83333
Ubr3	26320.6	21172	24533.6	26353.2
Ubr4	10028.5	8922.5	9659.75	9251.25
Ubr5	15821.25	22109.25	23233.25	23256.75
Ubr7	18148.5	17741.5	14920	16985.5
Ubtd1	2784	41116	2330	39126

Ubtd2	27680	28563	27975.5	30542.5
Ubtf	30548	28877	36758.5	30935
Ubxn1	35061.5	19085	21101.5	23505
Ubxn10	4844	7277	21306	18478
Ubxn11	34854	3136	36835	6741
Ubxn2a	28032	31108	22014	26745
Ubxn2b	27753	21790.33333	8027	8821
Ubxn4	18302.6	25561.8	18824.8	10560.2
Ubxn6	15766	18007	17872	18133
Ubxn7	10961	12771	15089.33333	16751.66667
Ubxn8	17876	22618	16810	19983
Uchl1	20127	16670.5	1261.5	25727
Uchl3	24182.5	25276	24990	24460
Uchl4	10854.5	14290	14328	13821.5
Uchl5	5562	5157	5075	5032
Uck1	23583	21321.5	17686.5	18956
Uck2	1787.5	10650.5	35235.5	4699.5
Uckl1	20910	17041	18114	17047
Ucma	24749	25361	31067	25741
Ucn	11114	10232	21263	17431
Ucn2	2768	5932	7902	14577
Ucn3	2480	31499	32612	22
Ucp1	11267	14436	17554	19350
Ucp2	24468.4	23965.2	18321.2	14287.8
Ucp3	26460	27851	29564	28240
Uevld	31347.66667	27798.66667	23686.66667	15023.66667
Ufc1	14609	13916	15424	13714
Ufd1l	23508.5	23095	18299	24242
Ufl1	19106.33333	18417.66667	18737	17755.33333
Ufm1	16089.5	17801	14490	13542.5
Ufsp1	19504	25433	19678	23365
Ufsp2	3546.5	5001	6492	9452
Ugcg	19486	21480.33333	14864.66667	28325.33333
Ugdh	11607	12118	16220	17666
Uggt1	12055	16396	33997.33333	33740.66667
Uggt2	32020.85714	28575.57143	30526.57143	28903.57143
Ugp2	6263.5	7324.5	12878	13743.5
Ugt1a1	40008	450	2240	7049
Ugt1a6b	18168	21878	17386.5	26763

Ugt1a9	34633	35530	17592	7935
Ugt2a1	18699	19916.66667	19905.33333	22124
Ugt2a3	23995	25209	4597	2098
Ugt2b1	24886	26028	28161	26948
Ugt2b34	40413	14817	2778	7291
Ugt2b35	6571	16642	18927	18667
Ugt2b37	18740	21258.5	25236	23948
Ugt2b38	540	40054	3381	4638
Ugt2b5	25407	35207	38700	27368
Ugt3a1	12829	15398	24378	17443
Ugt3a2	16799	16595.5	21246	8716.5
Ugt8a	30797	20034.33333	23401	25774.66667
Uhmk1	27754	24300	22873	22384.5
Uhrf1	15862.5	18322	16290	19236
Uhrf1bp1	13420	13286.5	17570	17715.5
Uhrf1bp1l	17227	8816.25	11685.75	8548.25
Uhrf2	23835.75	15602.25	21715.5	17287.5
Uimc1	19525.33333	24500.33333	12860.66667	13375
Ulbp1	21409	22095.5	19539	20072
Ulk1	8536	27482	6757.5	26158.5
Ulk2	28568.25	24572.25	26661.75	24856.25
Ulk3	18885	19375	24624	23381
Ulk4	14199.5	27970.5	12189.5	12597.5
Umod	15249	17458	24990	28813
Umodl1	24027	35097	26972	35139
Umps	26283.33333	23671	22521	20050.33333
Unc119	24503	20322	18434	14540
Unc119b	612	39438	36534	35828
Unc13a	14400	13529	19873.5	16190.5
Unc13b	12972.66667	10857.66667	17062.33333	13105.66667
Unc13c	4770.5	7352	29282.5	25495
Unc13d	14318	14955	16368	14637
Unc45a	27900.5	29079.5	30143	32499
Unc45b	2389	36303	7899	12670
Unc50	13579	14392	13730	11154
Unc5a	21518	26860	25841	24460
Unc5b	10759	17006	9177	21067
Unc5c	23400.66667	27084	35369.66667	35289.66667
Unc5cl	28242	29624	31991	31676

Unc5d	25694	20464	25271.5	22598.5
Unc79	28525.66667	23767	36926	21793
Unc80	23926	17535.66667	8432.333333	21605.66667
Unc93a	13627	12459	20938	17440
Unc93b1	28748.5	22041	24016	22131
Uncx	9454	26039.5	14465.5	30096
Ung	17313	20400	20342	22236
Unk	22152	23426	20554	19998
Unkl	21811	23498.5	7220.5	6667
unknown	20467.6	21715.2	19589.4	24331
Uox	10810	14217	15097	15046
Upb1	6027	16373	11062	19183
Upf1	4276	2263	8809	5625
Upf2	11812.75	12920.5	13807.75	24859.5
Upf3a	17365	16842.5	17979.5	17808
Upf3b	13774.5	22495.25	20219.5	17996.75
Upk1a	14757.5	13584	16931.5	16602
Upk1b	27479	10419	25741	20215
Upk2	18128	17772	26157	33046
Upk3a	40396	38033	35829	38310
Upk3b	17760	39412	16625	1536
Upk3bl	6865	11272	5984.5	10592.5
Upp1	21021	19604	21384	22683
Upp2	32875	34505	35821	36707
Uqcc1	11688.66667	10964.33333	10360.33333	9640.333333
Uqcc2	7352	17859	17695	18847
Uqcc3	14087	17782	20082	17681
Uqcr10	22073	33732	25930	30262
Uqcr11	21568	30871	28500	27720
Uqcrb	6557	11100	10087	9162
Uqcrc1	9700.5	8737	9292	10226.5
Uqcrc2	13694	15648	14395	17056
Uqcrfs1	6758	10286	5283	5332
Uqcrh	30117	31811.5	13559.5	32056.5
Uqcrq	21323.5	28501.5	25263	26798.5
Urad	11060	12315	23812	20791
Urb1	26791	16196	19407.66667	22019.66667
Urb2	36755	37613	35528	34836
Urgcp	24285.5	24423.5	24777	23729.5

Uri1	28599.33333	27388	24890.66667	27283.33333
Urm1	23882	24103.66667	24792.66667	25699
Uroc1	14055	18669	31252	34543
Urod	3324	3989	3107	4664
Uros	20028	23711	17861	20856
Usb1	10375	12326	3062	7082
Use1	40307	36897.5	39022.5	38157
Usf1	7552	7864	10337	8926
Usf2	32194	23322.5	22411.5	19123
Ush1c	2529	3856	7196	7307
Ush1g	35913	37323	39213	39495
Ush2a	16754.33333	23633	24161	28090.33333
Ushbp1	35556	4082	31791	27092
Usmg2	23501	24617	26352	25264
Usmg5	12359	19202	15328	17393
Uso1	19308.5	22676	20696.5	24388.5
Usp1	22889	24448	25015	28837
Usp10	18903	20039	17947	18503
Usp11	24623	25589	26745.5	25396.5
Usp12	38433.5	38394.5	31953	33361.5
Usp13	11286	7149.5	17249.5	13757
Usp14	13474.66667	15697.66667	15234	19742
Usp15	16878.44444	19617	19925.55556	20593.44444
Usp16	28748	34062	25332.5	32429.5
Usp17la	18027	17426	36567	36248
Usp17lb	28569	30342	31727	38528
Usp17lc	38732	1518	29845	1492
Usp17le	32938.5	21935.5	21107.5	5804.5
Usp18	9412	21140	15842	32435
Usp19	15313	14135.5	12934	13311
Usp2	39063	30677.5	25950	30079
Usp20	12380	14203	14297.5	14535.5
Usp21	2605	496	38647	34882
Usp22	25798.5	26382	31960.5	29867.5
Usp24	24658	18427.66667	17835.33333	18472.66667
Usp25	24046.66667	22034	19893.66667	23832
Usp26	26953.5	32342.5	34789	28516
Usp27x	20201	39952	5547.5	6367
Usp28	9322	6844	7831	7240

Usp29	17819.4	21719	20752.6	23952.2
Usp3	10272.5	12572.5	7810	12631
Usp30	33883.5	35697.5	33073.5	36191
Usp31	25669.5	18989	23073	24618
Usp32	14062.5	11701	14535	13002
Usp33	18919.16667	23137.33333	23782.66667	19296
Usp34	20307.5	14862.75	13522.75	21828.25
Usp36	18907.5	14867.5	9336	8687
Usp37	11690.5	12486.5	13510	13596
Usp38	24702.66667	21558	21792.33333	22748.33333
Usp39	10960	14118	11016	13191
Usp4	14347.33333	35455	23762.66667	20021.33333
Usp40	22854	17434.5	14977.5	18752.5
Usp42	4800	39202.5	25855	23480.5
Usp43	19980	22563	26825	27424
Usp44	15469	17109	31735	35188
Usp45	27789.5	23263.5	27291.5	27109.5
Usp46	33972.66667	21030.66667	22572	24795
Usp47	27447.71429	24167.42857	23178.14286	23085.71429
Usp48	16632.33333	10425.33333	24888	22281
Usp49	24720.5	23135	7129.5	19692
Usp5	23341	22117	18798	18738
Usp50	26042	27174	29903	28576
Usp53	16263.2	24100	20550.4	20379.4
Usp54	19193.5	16269	22073	23187.5
Usp6nl	21444	19919	22832	23568
Usp7	9971	4513	8411	7979
Usp8	14190	9853	30933.5	27925
Usp9x	15850.75	20202.75	22264.75	16494.5
Usp9y	32428	34302	38451	34161
Uspl1	29865	29888.5	14047	33049
Ust	21361	5229.5	7810	11691
Utf1	225	40264	41006	39120
Utp11l	3083	6255	6467	6574
Utp14a	11533	22152.66667	18624.33333	20100.33333
Utp14b	26949	37620	26795	37009
Utp15	38427	32960	32911.5	37074
Utp18	24458	11809	14604	11200
Utp20	19736.5	21016	20130	22125

Utp23	1308.5	4533.5	3594.5	6231.5
Utp3	22774.5	24144.5	5529.5	5289.5
Utp6	20147.33333	23008	21722.66667	23643.33333
Utrn	16784.28571	16763.85714	18933	19245.14286
Uts2	13053	15926	18757	14927
Uts2b	3115	7296	14529	15098
Uts2r	18006	18967	25343	26325
Uty	21806	27109.5	29177.5	28149
Uvrag	17864	18728.25	22744.25	20214.25
Uvssa	25395.5	21998.5	25876	22824
Uxs1	24978.5	25692	28155.5	25773
Uxt	27812	32102	32706	35123.5
V1rd19	4148	18247	31057	32696
V1rg10	34433	864	7790	6357
Vac14	21399.5	21712	19975.5	20047
Vamp1	21225	21160	22118	22511
Vamp2	11006.33333	10066	8271	20843
Vamp3	25027.5	23127	19835	24563.5
Vamp4	14892.66667	16347.66667	14218.66667	14964.66667
Vamp5	13509.5	19335.5	15442	22282
Vamp7	23823	36862	38030	34521
Vamp8	9808	19213.5	14395	18947
Vangl1	4210.5	7712.5	13719.5	13398
Vangl2	12873.75	15344	6550.75	17590.5
Vapa	18596.5	21787.5	20588.5	23105.5
Vapb	32961.33333	28406.66667	30509.33333	28362
Vars2	10770.75	9466.75	29449.5	27137.5
Vash1	15135	17514	21183	21813
Vash2	12083.5	8518.5	25036	24672
Vasn	16435	22958	20345	34310
Vasp	12432	11354	14068.5	27003.5
Vat1	20572	17806	21970	19048
Vat1l	18288.33333	18767	7756	19080.33333
Vav1	39209	20770.5	33573	38943
Vav2	20974.5	18209	20942	16369.5
Vav3	22353.33333	26377.33333	34095	27882.66667
Vax1	9318	10928	8489	10722
Vax2	29159	31136	31903	3138
Vbp1	33371	33639.5	31268	34124.5

Vcam1	13804.33333	28203.33333	19886.33333	22835.66667
Vcan	18323	20298.6	27777.4	24730.6
Vcl	21592	20293.5	24323	23680
Vcp	9166	10013.66667	22560.33333	13501
Vcpip1	26727.5	23324.5	22510.5	11270.5
Vcpkmt	10729	15619	13605	15693
Vdac1	16660.66667	14434	15928.66667	15303.33333
Vdac2	21455	21109	19909	17109
Vdac3	18846	19292.5	18679.5	20863
Vdr	35485	5051	32363	873
Vegfa	16482.66667	18110.33333	14863.66667	26144
Vegfb	11119.5	8301	26650.5	22692
Vegfc	11627	16589	20168	32740
Veph1	27110	19796	31623	24259
Vezf1	26559.5	22087.5	24708.5	24191.5
Vezt	14996.66667	20824	31025.33333	22452
Vgll1	17155	20557	14218	18351
Vgll2	36929	35319	36533	46
Vgll3	19483.5	17254	36047	17143.5
Vgll4	10466	2876	37411	40015
Vhl	3306	7082	6162	9566
Vil1	21284	21378.33333	15330.33333	29184
Vill	19472.5	15797	15750	14264.5
Vim	6448	19182.5	24265.5	2630.5
Vimp	15833	17670	17125	18240
Vip	30230	31848	36700	2056
Vipas39	16696.5	21486	19809.25	14611.5
Vipr1	3842	9778	13097	13856
Vipr2	16528	20113.5	31612	30220
Vit	33232.5	32636.5	21652	8529.5
Vkorc1	3972	3899	1599	38411
Vkorc1l1	20286.75	14536.5	18903.75	15123.75
Vldlr	7691	25658	24356.5	13257
Vma21	28082	26072.5	28005.5	21524
Vmac	19396	17748	16021	15547
Vmn1r10	36551	721	7278	9821
Vmn1r11	16608	17379	32917	24520
Vmn1r13	38545	4414	13864	11530
Vmn1r14	36640	4875	2300	39079

Vmn1r148	40893	65	883	6563
Vmn1r15	31475	33515	33839	32276
Vmn1r16	32907	35087	40016	33691
Vmn1r17	31831	32866	70	35440
Vmn1r171	16599	18128	25059	21892
Vmn1r172	37704	36330	39852	39918
Vmn1r18	14632	19721	23752	27683
Vmn1r181	15261	19418	14226	17737
Vmn1r183	35582	28968	31312	471
Vmn1r185	15525	17457	22473	21717
Vmn1r188	22326	25490.5	26704	27003
Vmn1r189	37046	32387	34248	40234
Vmn1r19	35781	32519	33794	32301
Vmn1r190-ps	15286	18093	20629	17256
Vmn1r192	10843	8953	15705	12731
Vmn1r193	2807	10640	7597	17981
Vmn1r195	8882	12138	14709	15085
Vmn1r196	24319	31845	39929	39947
Vmn1r198	30495	32118	34334	38673
Vmn1r199	304	3943	4941	3197
Vmn1r200	40367	40280	31771	5367
Vmn1r201	12694	15586	17725	18407
Vmn1r202	7707	18163	17890	18058
Vmn1r203	23896	25083	26566	25594
Vmn1r205	32828	35606	10562	10744
Vmn1r206	34717	518	37169	29764
Vmn1r208	2137	16786	9288	10362
Vmn1r21	15404	15505	16955	14951
Vmn1r210	2944	34113	35331	33510
Vmn1r211	29662	30926	129	31539
Vmn1r212	34471	23600	33947	38829
Vmn1r214	31928	37015	27668	26722
Vmn1r215	32438	34186	35511	36414
Vmn1r216	39895	743	6896	8232
Vmn1r217	23398	24502	26281	25300
Vmn1r218	3875	14867	17674	17452
Vmn1r219	30748	32370	33771	32565
Vmn1r22	14587	19352	22448	19725
Vmn1r220	23797	24975	26429	25368

Vmn1r225	22846	23206	29586	32728
Vmn1r226	3372	1743	4970	6201
Vmn1r227	5149	7138	10480	11377
Vmn1r228	13896	12442	14926	9268
Vmn1r229	14801	14738	22255	20175
Vmn1r23	3985	35218	2634	38785
Vmn1r230	34589	39662	2446	1035
Vmn1r231	28409	30806	1600	2838
Vmn1r233	29711	31252	32851	34791
Vmn1r234	38317	30949	39666	883
Vmn1r235	5520	2608	5789	8190
Vmn1r236	29301	27598	29798	35001
Vmn1r237	26266	32581	37927	39740
Vmn1r24	26655	27959	29680	28407
Vmn1r25	14816	17500	20398	21156
Vmn1r26	4067	4983	3288	2219
Vmn1r27	12253	16512	22530	21584
Vmn1r28	38718	1131	40787	756
Vmn1r29	32809	34220	37046	35137
Vmn1r30	27235	29029	29072	30869
Vmn1r32	31293	33538	34204	32391
Vmn1r33	34060	36708	33145	31444
Vmn1r35	25792	37718	29383	38473
Vmn1r37	2747	10036	13922	11360
Vmn1r38	24855	25942	28322	27131
Vmn1r40	10335	10343	14039	15566
Vmn1r41	39606	286	4564	4735
Vmn1r42	95	3386	6153	5456
Vmn1r43	1531	4486	8996	9167
Vmn1r44	31231	33249	35931	34838
Vmn1r45	40088	40354	965	35148
Vmn1r46	6772	7983	11866	10925
Vmn1r47	14623	17261	21208	21279
Vmn1r49	29638	31182	33404	1222
Vmn1r5	5598	39688	1347	11206
Vmn1r50	26179	27724	29611	1006
Vmn1r51	33212	32662	795	2821
Vmn1r52	5148	1714	1051	6769
Vmn1r53	8300	14116	14779	23539

Vmn1r54	22734	25593	23519	36985
Vmn1r56	9367	13837	20373	24616
Vmn1r58	36839.5	20528.5	4858.5	4699
Vmn1r6	1697	10330	11928	11015
Vmn1r62	30805	36060	1711	372
Vmn1r63	38879	6178	3155	14454
Vmn1r65	26197	38366	37368	36535
Vmn1r66	12603	13191	19730	13151
Vmn1r67	31578	33349	495	1259
Vmn1r69	29103	30675	8656	34792
Vmn1r70	26498	27622	30356	29011
Vmn1r71	31746	33446	35297	33559
Vmn1r72	11266	14393	9028	11063
Vmn1r73	26106	27465	29243	28105
Vmn1r74	31643	33378	34886	33111
Vmn1r75	12261	19287	19898	17376
Vmn1r76	21976	33237	37585	39717
Vmn1r78	9194	16007	24588	24106
Vmn1r8	32027	412	3006	35379
Vmn1r80	26495	35645	39190	39270
Vmn1r81	33754	38812	40692	37381
Vmn1r82	15174	22596	35176	33099
Vmn1r83	26558	27717	31093	34654
Vmn1r84	31904	33814	35448	39498
Vmn1r85	5095	3882	9091	8876
Vmn1r87	34021	5958	9111	8747
Vmn1r89	38783	1183	2631	3387
Vmn1r9	31015	39197	39730	2465
Vmn1r90	19045	17519	26178.5	3718.5
Vmn1r-ps103	1842	8000	11875	13069
Vmn1r-ps8	16954	18462	34626	34147
Vmn2r1	37375	13395	14111	11238
Vmn2r10	32647	34951	35108	33227
Vmn2r107	28540	15150.5	22642.5	19505
Vmn2r14	37050	491	3734	3084
Vmn2r16	35476	8868	11361	4528
Vmn2r26	26975	8150	30953	10377
Vmn2r29	26874	31951.5	29832.5	28509
Vmn2r30	15614	11638	21998	16998

Vmn2r37	40863	8145	13795	14449
Vmn2r42	12672	12140	20754	20161
Vmn2r43	40804	39567	14373	4496
Vmn2r53	15351	14691	24155	22828
Vmn2r57	21124	21162	34792	37799
Vmn2r58	10053	14725	16429	13662
Vmn2r7	31840	33676	11892	35857
Vmn2r81	22324	21014	36573	22878
Vmn2r89	23178.5	12166	14335	15696.5
Vmn2r-ps105	28490	30260	30229	29092
Vmn2r-ps11	17547	20653	26929	27751
Vmn2r-ps54	3691	6746.5	10212.5	10247.5
Vmn2r-ps88	36554	2962	12515	12857
Vmo1	6260	1189	35507	3937
Vmp1	20038.25	19898.25	30433.25	21637
Vnn1	20919	23622	23900	24774
Vnn3	17857	802	17531	37425
Vopp1	12250	22160.5	10383	21986.5
Vprbp	24099.66667	25630	23917.33333	30489.33333
Vpreb1	2757	38937	1925	27695
Vpreb2	843	1457	3000	3244
Vpreb3	22832.5	24571	11830.5	12066
Vps11	25664.33333	22989.66667	18561.33333	20356
Vps13a	25599.2	20204.6	22453	24801.8
Vps13b	18247.41667	26799.91667	22779.25	28060.41667
Vps13c	15206.6	20991.8	11901.2	16264.6
Vps13d	21308.25	24830.25	17998.75	19978.75
Vps16	33656	30457.5	30001.5	12750.5
Vps18	12836.33333	10928.33333	11765	11266.66667
Vps25	13346	14574	11995	14627
Vps26a	40470	1233	367	744
Vps26b	10793.33333	21966.66667	10974.66667	20701.66667
Vps29	16571.25	14734.75	12448.75	21852.5
Vps33a	9605.333333	14107.33333	13495	11829.66667
Vps33b	24960	20935	23410	22547
Vps35	20119.33333	32495.66667	29486	17336.66667
Vps36	22177.5	20501.5	17660.5	16615
Vps37a	14991	16551	19856	21935
Vps37b	21854	21036	20401	21261

Vps37c	5784	4969.5	2376	5243.5
Vps37d	34719	32862	1241	3041
Vps39	28341	19356.5	21404.5	16956.5
Vps41	38519	37146	37075	37193
Vps45	11884	12740	12291	12491
Vps4a	18250	18081	16530	18460
Vps4b	22516.66667	24440.33333	22581	24906.33333
Vps51	13725.5	16206.5	17009.5	38295.5
Vps52	19525	20845	20251	20021
Vps53	11712.25	21544.25	13345.5	21364.5
Vps54	14597.5	23437.25	15026.5	16781.5
Vps72	12142	12438	8141	9983
Vps8	38005	37623	29978	35238
Vps9d1	31751	22909	27865	24816
Vrk1	6030.5	7013	6354	3931
Vrk2	6522.5	4820.5	18321	9340
Vrk3	1125	38583	30744	35461
Vsig1	26311	35535	37642	29539
Vsig10	22350	21590	26491	24303
Vsig2	5651	6072.5	10049.5	9764
Vsig4	10664	33518	23086	9364
Vsig8	25557	25259	8785	11430
Vsnl1	21814	27987	12538	11861
Vstm2a	24371.66667	24500.33333	29321.33333	25789.66667
Vstm2b	34411	34161	3789	35013
Vstm2l	24982	32346	3137	39746
Vstm4	26170	27240	25178	24791
Vstm5	15873	18268	22278	24167
Vsx1	30009	32091	33165	30368
Vsx2	3557	9506	3034	5504
Vta1	22949	3867	23802	21100
Vtcn1	29968	31660	32221	30885
Vti1a	15394.4	15231.4	12006.8	16303
Vti1b	23599	27705	23786	24796
Vtn	40240	7676	40841	7593
Vwa1	17038	25480	16765	22254
Vwa2	26787	543	8474	40502
Vwa3a	33829	29082	40106	29418
Vwa3b	4092.5	7104.5	27978.5	12484.5

Vwa5a	36124	30116	36819	37386
Vwa5b1	23646	31006	40731	37732
Vwa5b2	21249	26295	25344	28129
Vwa7	40544	209	27757	1692
Vwa8	16420.5	20819.5	15424.25	12747.25
Vwa9	17534	17036	15672	15580
Vwc2	30015.5	34160.5	36184.5	34178
Vwc2l	12700	12418	18155	20825.5
Vwce	27230	41067	6408	11362
Vwde	8433	11553	19908	17729
Vwf	23799.66667	16635.66667	19906	16400
Wac	19340.4	16403.2	14606.8	15681.4
Wap	25602.5	26615	13900.5	11873.5
Wapl	25631.33333	22863.66667	24011.33333	22202.66667
Wars	29953	31336.5	23276	31335
Wars2	21589.8	17628.6	19543.4	18933
Was	21691.5	12983.5	15555	32327.5
Wasf1	15180	3470	14484	8177
Wasf2	22888.33333	14513.66667	18012.33333	12193.66667
Wasf3	40425	134	2905	2360
Wasl	14846	26451.5	25616.5	25525.5
Wbp1	40979	228	39738	38004
Wbp11	25651	21575	23066	22869
Wbp1l	23676	23296	22951.5	23641
Wbp2	7126	2791	40162	197
Wbp2nl	34956	38146	5795	7841
Wbp4	16041	31860	15578	17587.5
Wbp5	11239	10899	20020	12462
Wbscr16	6140	2332	1557	2526
Wbscr17	21709.5	22159.25	22958.25	23620.75
Wbscr22	24196	30298	28646	30649
Wbscr25	39593	1890	28182	1059
Wbscr27	17990	19698.5	20936	25760
Wbscr28	19466	20796	35599	30144
Wdfy1	12269.5	13897.5	7752.5	8703
Wdfy2	482	36937	40067	40005
Wdfy3	22583	20270.4	21865.4	20588.8
Wdhd1	28329	27972.5	31200.5	28816.25
Wdpcp	14708	21529	25486.66667	21156.66667

Wdr1	40394	37167	426	39323
Wdr11	8840.5	4895.5	6541.5	2568.5
Wdr12	10720.66667	14990.33333	11258	13799.66667
Wdr13	17500	14173.5	14517	14400.5
Wdr17	29916	31528	32336.5	30856
Wdr18	7905	22541	18714	17438.33333
Wdr19	23384.25	20457.5	25333.5	22674
Wdr20	27699.4	20811.4	15703.6	14644
Wdr24	13093	10958	9907	8734
Wdr25	26575	22550	26301.5	23228.5
Wdr26	17859.5	29147.5	15765.5	12491
Wdr27	15317	21409.5	25226.5	25306.5
Wdr3	23707	24189	25255	27740
Wdr31	17374	19924	18183	23711
Wdr33	11159.33333	15835.16667	20068.33333	14259.16667
Wdr34	38311	38656	33056	31300
Wdr35	12844	14447	14766	14730
Wdr36	24253	20718.5	16273.5	20676.5
Wdr37	9666.142857	13510.28571	18905.85714	11577.85714
Wdr38	21742	22178	25217.5	22620
Wdr4	11453.5	10742	28327.5	27768
Wdr41	38173.5	17862	16156	16585
Wdr43	8532.5	6956.5	5294	7256
Wdr44	35310	25778.5	29333	27576.5
Wdr45	13160	14288	13076	14079
Wdr45b	21294.33333	20498.66667	19437.66667	21257
Wdr46	878	1343	2800	4657
Wdr47	13695	7583	7311.5	7238
Wdr48	9090	12027	10065	11974
Wdr5	23764	25762.5	21954.5	26229
Wdr53	24908	24169	24121	24854
Wdr54	17138	11855.5	21940.5	25830.5
Wdr55	31101	35125	31188	34745
Wdr59	16660.66667	13260	17494.33333	17706.33333
Wdr5b	26711	25169	28869	29501
Wdr6	36645	40282	28175	32975
Wdr60	20248.66667	27742.33333	16184	10063.33333
Wdr61	20623.5	19500	18585.5	18058
Wdr62	28020	27193	26286	25515.5

Wdr63	8917	3411	9789.5	22334
Wdr64	32122	33720	6726	401
Wdr66	17057	23730	20783.33333	20899.33333
Wdr7	16385.66667	11863.33333	11024	10582.66667
Wdr70	21855	22943	25412.5	24806.5
Wdr72	29072	40176	27659	26563
Wdr73	22570	23851	21480	23419
Wdr74	18672	17526	13078	17405
Wdr75	18303	18360	16070	19138
Wdr76	1705	40381	6990	3369
Wdr77	17059.5	15476	9939.5	13968.5
Wdr78	15272.5	15966.5	3978	7532
Wdr81	27812.5	22589	24962.5	22257
Wdr82	24454.5	22505	25716.5	26041
Wdr83	9837.5	11694	9359.5	8634
Wdr83os	23484.5	19920.5	18651	17163
Wdr86	19672	21922	21164	22946
Wdr90	21321	19082	18333	16619
Wdr91	38702	302	32753	37113
Wdr92	28644.5	12676.5	27282	9917.5
Wdr93	23139	30627	1700	5365
Wdr95	7435	9240	6245	8756
Wdsub1	13597	14059	14839	15094.5
Wdtc1	6753	12881.5	11413.5	15655
Wdyhv1	24374	26954	25116	26363
Wee1	38124	35264	40050	36099
Wee2	10922	13669	14815	17492
Wfdc1	17949	16409	20259	19703
Wfdc12	22938	22290	28519	33243
Wfdc15a	17083	17373	18679	17423
Wfdc15b	18044	21386	31860	31984
Wfdc2	21514	23207	27954	25896
Wfdc3	7754	14189.5	11045.5	9962.5
Wfdc5	18766.5	19725.5	23349.5	24763
Wfdc6a	23101	23202	22897	31169
Wfikkn1	9334	6650	15126	7214
Wfikkn2	15734.66667	16490.66667	17219.66667	19295.66667
Wfs1	22593	13921	18671	14817
Whamm	17432.5	18232.5	6478.5	22190.5

Whrn	25020.5	11424	16044.5	23731.5
Whsc1	14912.8	19719.4	29116.4	13256.2
Whsc1l1	12630	20949.25	3134.25	20304.75
Wif1	38918	19745	5738	17738
Wipf1	21142	27357.5	20489	32245
Wipf2	16746.5	12747.5	16189.5	15068.5
Wipf3	32865.5	32894	18956	18580
Wipi1	30829	30025.5	24806	29981
Wipi2	19015	9383.75	8939	9722.25
Wisp1	22738	20673	39567	6882
Wisp2	4101	7309	8554	7308
Wisp3	5943	36357	5262	582
Wiz	23547.5	20164	19796.5	19380
Wls	35099.66667	22830.33333	24721.66667	19630.66667
Wnk1	19618.66667	13681.33333	16750	19035.33333
Wnk2	14174	22733	17562	25837
Wnk3	28852.33333	21373.33333	31741.66667	24860
Wnk4	9652	8379	4920	13629
Wnt1	10611	11935	16137	15845
Wnt10a	13814	12030	12809	13650
Wnt10b	2432	31843	2704	32930
Wnt11	6958	8746	7766	8827
Wnt16	4800	35444	7992	7390
Wnt2	19051	15621	38964	28411
Wnt2b	28419	38640	39838	1924
Wnt3	30741.5	32392	34250.5	32522.5
Wnt3a	41013	41010	1899	39601
Wnt4	34195	15033	26086	21199
Wnt5a	8432	5137	34846	33045
Wnt5b	9524	11448	14129.5	14531
Wnt6	17607	21508.5	22338	24371
Wnt7a	11999	16161	21123	19222
Wnt7b	8984	12278	14242	16448
Wnt8a	28130	29493	32378	30788
Wnt8b	7511	6949	18233	10381
Wnt9a	20247	5506	6568	8327.5
Wnt9b	9057	12130	13260	15009
Wrap53	11213	10743	13453	13667
Wrap73	9667.5	10105.5	6081.5	6431

Wrb	24191.66667	33317.66667	21504	19942.66667
Wrn	20377	19670	19657.25	19352.25
Wrnip1	21714.66667	22434.66667	21203	20564.66667
Wsb1	24785	28092.5	29999.5	28398
Wsb2	16944.66667	24654.66667	22996.33333	15104.33333
Wscd1	30799	20040	6181.5	22404.5
Wscd2	17090	6903.666667	8484.333333	10517.66667
Wt1	32516	21238	28916	36007
Wtap	19531.6	20970.2	18586.6	23380
Wtip	20302	20078	26074	24268
Wwc1	3777	33556	5665	34719
Wwc2	29808	22322.5	17143	15608.5
Wwox	14549.2	18527.2	15620	18094
Wwp1	19174	22296.6	24696.4	25335.4
Wwp2	16185.4	20433.6	21434.2	20292.4
Wwtr1	17565	35441	34366	38562
X99300	35090	4073	7854	9292
Xab2	20019	18065	20601	20091
Xbp1	1726	4092	3937	4099
Xcl1	35055	32230	33846	34025
Xcr1	21247.5	23888.5	28301	28662
Xdh	24835	16293.5	24054.5	17375
Xiap	26628.33333	19756	19417	18101.33333
Xirp1	29731	35038	40413	939
Xirp2	26670	26574.66667	29349	27951.66667
Xist	19482.5	27995.5	28486	29639.75
Xk	28179.25	30548.75	23735.25	18537.5
Xkr5	13563.5	21307.5	26066	6634.5
Xkr6	39412	5878	1524	37914
Xkr8	14815	22503	20380	24301
Xkrx	25532	26643	29063	27870
Xlr	34514	38974	3815	3977
Xlr3c	22297	23346	13805	12472
Xlr4a	39244	40718	40380	39330
Xlr4b	27812.66667	29762.33333	22546.66667	24529.66667
Xlr5c	28115	36824	38951	6125
Xlr5d-ps	6749	11664	14140	22563
XM_620320	32718	34562	35941	34398
XM_622288	31539	2448	5676	38492

XM_622779	17713	22076	18285	17851
XM_622813	389	8608	37011	2540
XM_622871	2725	38996	5720	671
Xpa	11796	12775	17161	15363
Xpc	27615	28646	9714	27920
Xpnpep1	11433	13435	4502	8582
Xpnpep2	38304	40125	36552	28004
Xpnpep3	10002	19552.66667	19250	20309
Xpo1	19554.66667	15432	19358	20721.33333
Xpo4	22931.5	15647	9736	13039.5
Xpo5	30969.25	31156.5	28966	28242.5
Xpo6	4842	19274.5	16418	35546
Xpo7	14254.33333	24018.66667	21064.66667	20237
Xpot	19207	20087	19766.5	21004
Xpr1	10667.66667	19968.33333	10851.66667	21911.66667
Xrcc1	3034	39749	38337	36080
Xrcc2	19126.5	14893.75	18651.5	10189.75
Xrcc3	3679	35538	3133	36941
Xrcc4	19215	28411.5	20985.5	21771.25
Xrcc5	19771	17883	23492	18458.5
Xrcc6	31875	32223	28868	26275
Xrcc6bp1	17977.5	15442	16748.5	9825.5
Xrn1	8304.666667	21071.66667	16884	17722.33333
Xrn2	9318	28305	28766	29525.5
Xrra1	21278	24806	27179	24621
Xxylt1	22595	25809	20875	21245
Xylb	17000.5	17202	14455.5	15098.5
Xylt1	35097	38022	40592	1128
Xylt2	22293	34262	22653	19009.5
Yae1d1	9675	10514.33333	20980.33333	19828.33333
Yaf2	23790	37423	25829	29893
Yap1	40922	554	9889	12645
Yars	12739	12914	10382	13138
Yars2	14747	16734.5	13947.5	17943.5
Ybey	16960	17250	18694	17577
Ybx1	24235.66667	17944	23750.83333	17684.5
Ybx2	35089	40066	2908	2848
Ybx3	28774	34975	30592.5	36574
Ydjc	20247.5	25021.5	6707.5	6171.5

Yeats2	11006	7999	3357.5	9364
Yeats4	35694	35170	33650	31735
Yes1	32643	34487.5	20708	24438.5
Yif1a	22967	25554	22289.5	25902
Yif1b	8931	11143	10546	11490
Yipf1	24663.5	26756	2984	6546.5
Yipf2	38909.33333	13997	2348.666667	2375
Yipf3	17152	16067.5	13801.5	14873
Yipf4	12634	17343	15560	17724
Yipf5	17235.5	19225.5	13770	19288.5
Yipf6	21822.25	26016.75	17470.25	26249
Yipf7	23168	13538	23299.5	21284
Yjefn3	13808	18979	17077	16470
Ykt6	23177.66667	26597	26551	28034
Ylpm1	17300.25	21211.75	14567.25	12364.5
Yme1l1	15557	11063.75	19387.75	23474.25
Yod1	22459	19793	19705	20008
Ypel1	25527.66667	23078	24180	14383.66667
Ypel2	38750	35101	22321	24546
Ypel3	19304	16446	20336	15535
Ypel4	4139	6426	16959	15736
Ypel5	21061.5	25593	20348.5	24426
Yrdc	10871.5	15426.5	12383.5	15834.5
Ythdc1	36289	36297	39674	37398
Ythdc2	19405	21586	21587	20535
Ythdf1	11280.5	9267	7849.5	9019
Ythdf2	16233.5	17333	18751.5	18194.5
Ythdf3	15514	15704.5	19266.5	18166
Ywhab	16993.5	14568.5	14403.5	18008
Ywhae	18286.66667	17574.66667	14603	28766
Ywhag	16296	13695	19024	16620
Ywhah	94	32124	393	36755
Ywhaq	22889.66667	24654.66667	23061.33333	24226.33333
Ywhaz	28715.66667	27299.66667	28465.66667	29338.66667
Yy1	16294	15953	16029	15385
Zadh2	21051	35491	25215	38247
Zak	24798.66667	20577.66667	25601.33333	24541.66667
Zan	7776.5	8377	10913	14420.5
Zap70	33409	30526	327	2166

Zar1	10022	5890	14251	11024
Zbbx	8404	14573	17291	14625
Zbed3	1288	20940.5	29522.5	29417
Zbed4	19422	35456.66667	28992.33333	28740
Zbed5	20626	28421	26004	36973
Zbp1	4816	24005	11616	36802
Zbtb1	11254	14673.66667	21545.33333	12785.66667
Zbtb10	15607	5848.666667	21548.66667	15244.66667
Zbtb11	21517.33333	19568	18866	22792
Zbtb12	17341	20471	15149	18519
Zbtb14	10028.5	10221	11097.5	29661
Zbtb17	27031	28299	23212	24808
Zbtb18	30897	22187	36304	31611
Zbtb20	8966	23981.6	18889.6	16747.2
Zbtb21	27847	26999	12945.5	28007.5
Zbtb22	12500	14402	15561	14288
Zbtb24	24415.33333	29832.33333	26818	27975
Zbtb25	12325	13599	11861	10387
Zbtb26	33174.5	31604	30998.5	33269
Zbtb3	28682	30458	25976	28987
Zbtb32	15009	19981	16399	13345
Zbtb33	11199.5	5947.5	9119	10100
Zbtb34	25230	19243	25547	22318
Zbtb37	27439.5	2301.5	6993.5	5539
Zbtb38	20517	12915	7711	38392
Zbtb39	16851	11205	10865	11040
Zbtb4	28291.5	22667	30008.5	27882
Zbtb40	30145.66667	25256.66667	33012.33333	29833.33333
Zbtb41	11625	8748	9310	9632
Zbtb42	38221	37111	27958	32498
Zbtb43	25351	21335.5	39879.5	40756.5
Zbtb44	21048.5	17450	4434	22432
Zbtb45	12540	11180	9147	6404
Zbtb46	24290.4	19302.4	19108.4	23106.8
Zbtb48	21153	18810	16001	17711
Zbtb49	25446	19510	26752.33333	24610.33333
Zbtb5	5941.5	24568	18512	20032.5
Zbtb6	7860.5	7176.5	11309.5	7126
Zbtb7a	15264.33333	23316.66667	11416.66667	20104.33333

Zbtb7b	18379	19930	21510	22290
Zbtb7c	18474.5	16924	22134.5	25067
Zbtb8a	21199	19634	23104	19995
Zbtb8b	3059	35744	6016	8738
Zbtb8os	14930	15684.5	16296	15204.5
Zbtb9	34095	38311	36020	31990
Zc2hc1a	21363	20159	33873.5	38921
Zc2hc1b	39015	35515	36350	30672
Zc2hc1c	20219	20545.33333	34247.33333	14147.33333
Zc3h10	16443	14500	14208	15360
Zc3h11a	14909.25	9766	10291.5	8879
Zc3h12a	264	1897	40090	3350
Zc3h12b	31313	33022	35244	39174
Zc3h12c	19456	38784	19926	40804
Zc3h12d	40707	26219	4252	35623
Zc3h13	24662.33333	24998	17670.33333	19373.33333
Zc3h14	25666	26942	25154	27330
Zc3h15	28812.66667	28531.33333	27469.33333	27106
Zc3h18	21560	20342	19576	17138
Zc3h3	26035.5	23246	28557.5	29805
Zc3h4	16680	13874	14341.5	13352
Zc3h6	14585.75	29650.25	10286.25	26311.75
Zc3h7a	4732.5	4367.5	5193.5	7936
Zc3h8	14925	14521	9612	8596
Zc3hav1	9198.5	11205	9162.25	12273.25
Zc3hav1l	12490	16224	15628	18390
Zc3hc1	8677	10657	9488	12559
Zc4h2	21615.5	19320	23219	21438.5
Zcchc10	14011	17950	16682	17012
Zcchc11	25495.2	13983.2	18562.4	21639.2
Zcchc12	32534	34020	35330	33503
Zcchc13	30720	25135	39824	34565
Zcchc14	24688.5	17373.5	29518	28279.5
Zcchc17	26897.5	25720	24562.5	23518
Zcchc18	15428	18208	27429	3848
Zcchc2	36165	21773	39245.5	6111.5
Zcchc24	38131	24646	39012	28800
Zcchc3	6724	9599	10638	10697
Zcchc4	34191	36529	31079	36517

Zcchc5	28010	29382	31537	30022
Zcchc6	25700	12671.33333	13000.33333	14169.33333
Zcchc7	18515.6	22458.4	22992.8	20811.6
Zcchc8	15906.66667	25078.66667	22957	23877.33333
Zcchc9	20655	22039	21219	22801
Zcrb1	9725.5	9172	4464	22876
Zcwpw1	12836	13020	14835	13588
Zcwpw2	33176.5	16739	17686	17747
Zdbf2	14482.66667	17730.33333	21433.66667	21732
Zdhhc1	3067	2836	2341	40686
Zdhhc11	27148	38651	30558	32705
Zdhhc12	14294	20578	15307	23108
Zdhhc13	13131	14481	9576.5	11364
Zdhhc14	15992.5	26433.5	17310.5	24087.5
Zdhhc15	29735.66667	31201	33327	35084
Zdhhc16	2302	40551	37092	37537
Zdhhc17	16776	15267.33333	13974	13707
Zdhhc19	14886	20599	25864	25002
Zdhhc2	24486.25	20510.25	29629.5	23853.75
Zdhhc20	23383.25	24289.5	22661.25	24301.5
Zdhhc21	28814.5	8251	25532	24665.5
Zdhhc24	16362	14286.2	21743.2	14546.6
Zdhhc25	16028	18015	21125	23819
Zdhhc3	17783	17214	16105	15419.5
Zdhhc4	9132	7652	2696	1918
Zdhhc5	29506	28926.5	10784	30163.5
Zdhhc6	31450.5	31453.5	30856.5	37410
Zdhhc7	35676.5	28040	24645	23293.5
Zdhhc8	21781	19616.66667	9389.666667	19868.33333
Zdhhc9	17578.5	35659	19170	35889.5
Zeb1	7112	17439.5	10860.5	3516
Zeb2	17849.625	17074.375	20315.5	23383.125
Zer1	29787.5	24479.5	20067	17348
Zfa	26872	31711	28471	27463
Zfand1	22697	22760	22097	19452
Zfand2a	18484	11423.5	19858.5	14263
Zfand2b	40514	2741	1510	2946
Zfand3	7144	14861.33333	28426.33333	17878.66667
Zfand4	20857.5	2957.5	21743	39301.5

Zfand5	16270.75	12019.75	11972.75	13417.5
Zfand6	24622	34042	26055	29723
Zfat	26380	26142	21825	29165
Zfc3h1	20997.16667	13253	15082.83333	12927.16667
Zfhx2	27995	21732	30343	32036
Zfhx3	13969	10418	18806.5	18421.75
Zfhx4	21653	30586	32489.66667	32626.33333
Zfp1	22561	29547	24402	26108
Zfp101	18230	13169	17422	14268
Zfp105	41091	2973	397	1418
Zfp106	16355.25	15838.5	15248.75	16164.25
Zfp108	24615.5	23331.5	22812.5	20883
Zfp109	40365	28405	30773	29302
Zfp11	14985	15925	14984	15650
Zfp110	16566	8982.5	27195.5	27984
Zfp111	24289	23883	23859	23869
Zfp112	22195	23098	25867	25450
Zfp113	21769	14852	23746	20676
Zfp114	7017	9652	14647.5	13015
Zfp119a	22428.33333	18071.66667	15545	4079.333333
Zfp119b	9008	8169	6680	6323
Zfp12	17061	15622	16503	14988
Zfp120	14531	9835	10665	9565
Zfp125	1996	34794	36400	35180
Zfp128	15729	7046	9225	7166
Zfp13	11269	16796	16031	21055
Zfp131	34769	31981	28960	33560
Zfp133-ps	30438	20407	33154	31559
Zfp14	7421	3245	1884	40799
Zfp141	2427	2168	687	2386
Zfp142	15173.5	13558	13712	14963
Zfp143	20015	21695.5	21976.5	4254.5
Zfp146	14719	7275	4510	3992.666667
Zfp148	19243.8	18848	12977.6	18879.4
Zfp157	17837	21075.75	14656.5	13290.75
Zfp160	31088	22806	24398	26096
Zfp169	8456.5	27153	9174	28789
Zfp174	14433	9463	7216	3681
Zfp180	11268.5	13368.5	12793.5	13254

Zfp182	12507	8264	13225	10706
Zfp184	39402	7213	39698	5189
Zfp185	30375.5	28246	38619.5	16794.5
Zfp189	13934	11370	8875	8945
Zfp191	13312.33333	22285.66667	21785	22565.66667
Zfp2	21758	22171.75	24969.25	26997.75
Zfp202	23340	20126	23505	20713
Zfp207	22310	19567.25	21649.75	27500.5
Zfp212	19007	17269	16676	15513
Zfp217	11420	7506	7312	8519
Zfp219	34610.5	22842	26105.5	21824.5
Zfp229	35266	32762	26307	25975
Zfp235	16928	18197	18252	14886
Zfp236	26970.66667	23702.33333	21405.33333	24991.33333
Zfp239	5135	6227	5472	1533
Zfp248	12557.5	16708.5	15071.5	14775
Zfp251	19190	20444.5	21331.5	19171.5
Zfp26	17158	15196.375	22381.75	22271.625
Zfp260	20354.5	38580	19649.5	20832.5
Zfp263	10780.33333	13768.66667	13320	15497
Zfp266	14547	20731.33333	15491	12358.33333
Zfp27	15632	16718.5	16749	16490
Zfp273	22542	25403	20083.66667	21355
Zfp275	38936	31092	30011	34755
Zfp276	15198	13205.5	14653	14830
Zfp277	15607.33333	15212.33333	15738.66667	14537
Zfp28	33583	29833.5	32109	30716.5
Zfp280b	20487	20817	20359	19599
Zfp280c	24444	16345.5	22940	21487
Zfp280d	24690.8	24471.4	31836.2	31088.2
Zfp281	17581	11530	16338	12195
Zfp282	16363	31953.66667	27191.33333	28614.33333
Zfp286	6284	6599.5	6176.5	2897.5
Zfp287	24592.25	23627.25	26718.25	16544.75
Zfp292	19312.8	22756	18898.8	19505.6
Zfp296	20369	24160.5	22022.5	22393.5
Zfp3	13089.5	17389.5	13940	16406.5
Zfp30	17704.5	15388	12481	9419.5
Zfp300	35261	3923	3919	3437

Zfp316	27661	23482	22609.5	26878
Zfp317	23855.5	22061	14821.25	23477.5
Zfp318	8725.2	32444	17935.2	29835.6
Zfp319	17784.5	16586.5	37627.5	35570
Zfp322a	19216	14711	14921	13457
Zfp324	21400	18908	17181.5	13108.5
Zfp326	20675.25	24342.25	19375.25	27025.25
Zfp329	8102	7748.333333	22475.33333	10296.33333
Zfp330	13075	16819	16830.5	18189
Zfp334	23635	24727	4075	25125
Zfp335	7672	4619.5	4158	8366.5
Zfp33b	27023	26164.66667	15890.33333	16078.66667
Zfp341	35099	34871	31617	34162
Zfp346	19292	15122	15824	14546
Zfp35	23163.5	23507.5	22869	25345.75
Zfp352	23697.5	32343	28103	27437.5
Zfp354a	17468.5	15308	9596	27577.5
Zfp354b	33638	3335	10218	38193
Zfp354c	789	36205	11831	40707
Zfp358	4077	3483	39818	38375
Zfp36	16132.5	10346.5	12410	8557.5
Zfp362	33012.5	11435	31473	29613
Zfp365	7222	11606	13305	10534
Zfp367	22084.25	23036.75	22184.75	22687.75
Zfp369	24960.25	30280.25	5271.75	12474
Zfp36l1	24639.5	3032	22634	3853.5
Zfp36l2	18730	18042	19071	15774
Zfp36l3	4748	1252	6807	3818
Zfp37	15210.5	16030	18605	22427
Zfp383	27052	14434	13994	21127
Zfp384	18992.66667	17688	19451	17952.33333
Zfp385a	33872.66667	26524.33333	27971.66667	24265
Zfp385b	26770.75	20066.5	25511.25	30224.5
Zfp385c	20196	16435	18205	19733
Zfp386	16566.83333	20146.5	18736	18650.16667
Zfp39	9903	8273	12056	6705
Zfp395	1767	7505	3945	2281
Zfp397	22095	17172.5	18166	32843.5
Zfp398	19074	29321.33333	22141.33333	24185.33333

Zfp40	12674	6705	7333	6477
Zfp407	9445	22691.5	21668	16511.25
Zfp41	30556	33885	27124	31604
Zfp410	17184	18434	18516	18816
Zfp414	36012.5	29654.5	30088.5	28626.5
Zfp418	14367	14715	14330	15866
Zfp42	32084.5	16908.5	8041	21490
Zfp420	23628	22220	22469	22367
Zfp422	1945	6684.5	21338.5	2426.5
Zfp422-rs1	24265.83333	23334.5	22124.16667	21088.66667
Zfp423	25929	29846.5	28179.5	30134
Zfp426	15951.66667	23643.66667	23422	24490.33333
Zfp428	71	4285	5103	5941
Zfp429	14481	17143	12563	16067
Zfp433	647	39143	37597	38288
Zfp438	2071	2266	1953	40649
Zfp442	16526.5	31137	32016.5	29331
Zfp444	16998.5	13919	14392	14661.5
Zfp445	10523	19234.66667	27223.33333	27436
Zfp446	5721	8328	7151	5756
Zfp449	17009.5	13695.5	21556.5	19283
Zfp451	11207.75	9907.5	8673.75	12584.25
Zfp454	38328	36192	940	37745
Zfp455	40976	2159	39276	1711
Zfp458	17864.66667	30013.66667	20183	34198.66667
Zfp459	25720	26839	29467	28191
Zfp46	19429	16479	17627	16947
Zfp462	15288	12068.5	27780.5	28833
Zfp467	31134	19322	21464	11754
Zfp472	27256.33333	25287.33333	23287.33333	21577
Zfp473	23849	23786	27781	25873
Zfp474	39954	6673	35808	3427
Zfp493	20805	26258	23585	25153
Zfp503	5637	9780	10599	11336
Zfp507	11055.5	14525.5	14915	18578
Zfp51	8797	8942	6034	7486.5
Zfp511	20127	23343	23443	24108
Zfp512	40027	39648	40431	38591
Zfp513	16431	16429	17448	15947

Zfp516	23669.66667	23028.33333	20208	26205.66667
Zfp518a	11694.5	22558.5	8031	5492.5
Zfp518b	9541	4954	6834	4590
Zfp52	29068	29080	29010	29153.5
Zfp521	18550.5	36499	20118	18258
Zfp523	13013	18179	12088	17689
Zfp524	23863	23837	23707	23040
Zfp526	25213.5	23865	6816.5	24080.5
Zfp53	8568.666667	11269.66667	24638.66667	19175.66667
Zfp532	28171	23974.66667	23716	22709
Zfp536	27874.66667	30976.66667	33361.66667	34194.66667
Zfp54	19486.5	21111	19199.5	19344.5
Zfp553	30292.33333	26091.66667	26566.33333	26193.33333
Zfp558	10244.5	13337	11002	14841.5
Zfp560	22708	20114.33333	19798.33333	18137
Zfp563	5807	5733	4570	4250
Zfp566	6125	7355	6716	7577
Zfp57	1456	11807	15531	12269
Zfp572	15698	18877	27529	29215
Zfp574	11007	12552	12669	11188
Zfp579	14324	8435	13800	10573
Zfp58	5106	13296	18039	21099
Zfp580	36976	40020	27758	29189
Zfp583	4576	6382	4268	1572
Zfp59	1143	5065	40176	2432
Zfp592	23376.5	18786.5	20373.5	18716.5
Zfp593	34970	21362.5	38851.5	456.5
Zfp595	13744	10294	9270	9795
Zfp597	35959	26395	24977	24728
Zfp598	26829	22642	22013	23457
Zfp599	29013.5	22425.5	11587	12540
Zfp60	26458.5	20242	24541.5	24077
Zfp605	35219	20188	17145	13238
Zfp606	4766.5	14179	15667.5	13356
Zfp607	29146	36476	24500	36654
Zfp608	17935.33333	15125	14679.66667	16699.33333
Zfp609	14670	11639.33333	24651	24632
Zfp61	21726.5	22722	19860.5	20680
Zfp617	3858.5	23413.5	17989.5	20753.5

Zfp618	15850.33333	17409.66667	16000	22581.33333
Zfp62	13314.33333	14581	14600	15059
Zfp622	33915	35400	34236	35372
Zfp623	7760	7599	5054	8048
Zfp626	12460	14107.5	14227	13712.5
Zfp628	11946	9765	13875	11914
Zfp629	26871	17755.5	16105	14623.5
Zfp637	38990	40097	37556	34010
Zfp638	15950	23107.6	18038.2	24890.8
Zfp639	36116	35831	33977	33504
Zfp64	9738.666667	12089	13585	14306.33333
Zfp640	38030	16430	13458	10990
Zfp641	33201	30505	31871	30779
Zfp644	28049.5	24721.5	33848.25	35152.75
Zfp646	28778.5	28699	29892.5	27891
Zfp647	17422	16171	16584	13004
Zfp651	2376	2284	9834	8244
Zfp652	13345.33333	20906.33333	22195.66667	22277.66667
Zfp653	5975	9286	6053	6359
Zfp654	17071	16736.5	18022	21251.5
Zfp655	9449.25	9735.75	11808.75	12484
Zfp658	8331	11823	6485	13322
Zfp661	20802	16471	20961	18787
Zfp663	34477	8562	8961	11918
Zfp664	20016	34805.5	30655	28232
Zfp667	9376.5	27108.5	23927	21691
Zfp668	14061.5	26223.5	23894	21529
Zfp672	17166.5	18209.5	17869.5	15359
Zfp677	35298	28008	25483	30835
Zfp68	18335	21124.5	18039.5	23141.5
Zfp687	15836	12835	18794	15887
Zfp688	15327	15690.5	16576	17227.5
Zfp689	3012	4441	5905	7736.5
Zfp69	15866	21003	18825	23794
Zfp691	6881	10919	13592	14046
Zfp692	4481	41027	1389	37558
Zfp697	29550.5	29347.5	3569	18776
Zfp7	21701.6	23095.8	18916	20736.6
Zfp704	13086.8	13569.4	19518.8	22832.4

Zfp706	28819.66667	14421.66667	21498.66667	20957
Zfp707	35882	17497	36970.5	18111.5
Zfp708	19491	19291.5	40472	19294.5
Zfp709	28497	24143	21221	23285
Zfp710	15527	12116.5	17088.5	12784.5
Zfp711	23656	30713.5	31567.5	30264.75
Zfp712	38605	38972	38890	36886
Zfp715	16958.25	18210	20644.25	19775.5
Zfp71-rs1	20066	19664	20313	18204
Zfp729a	14017.33333	24571.66667	23014.33333	21698
Zfp729b	16229.5	30935	28951	30017.5
Zfp735	31103	32731	34150	11094
Zfp738	24773.5	7172	6131	6393
Zfp74	8393.25	7548.25	9572	8883.5
Zfp740	31456	29701	31586	32876
Zfp747	12570	11944	11102	11110
Zfp748	19102.66667	9327	10182.66667	9985
Zfp750	38839	3265	32142	40483
Zfp758	20214.5	19015.5	15064	18000.5
Zfp759	27245	25193	23020	31069
Zfp760	1680	38	11468	16411
Zfp763	16274	14507	15416	12725
Zfp764	21140	20734	21526	20618
Zfp768	31582	25223	30369	30014
Zfp770	25356.25	21749.5	19540.75	20465
Zfp771	35411	39370	37083	35590
Zfp772	36256	37462	38055	37693
Zfp773	14176	16561	15106	15327
Zfp775	20040	23200	28256	32959
Zfp777	28221	27181.5	29044.5	27690.5
Zfp780b	14541	10832	11520	11282
Zfp783	7246	4171	9437	6071
Zfp784	19606	17530	17322	16288
Zfp786	30380	31975	33462	32401
Zfp787	12925.5	15779	17955	16155.5
Zfp788	17670.66667	17274.33333	20128.33333	20925.66667
Zfp790	17142	14213	12859	12928
Zfp799	36305	33247	25798	26493
Zfp800	24528.5	22754.5	17501.5	20541

Zfp804a	29141	30778	34986	36252
Zfp804b	9942	12850	6691	11095
Zfp809	25177.5	22412.5	20142.5	19224.5
Zfp81	11905.25	10324.5	12591	12544
Zfp810	24259	20492	19337	16759
Zfp811	33630	37635	30799	30151
Zfp819	40545	4680	13960	14198
Zfp82	30723	11687.5	15679.5	36969.5
Zfp820	22201.33333	3719.333333	21019.33333	21631.33333
Zfp821	1939	40669	979	38918
Zfp825	24388.5	24235	22561	23000.5
Zfp826	23928	16956.33333	17646.33333	17873
Zfp827	23258.33333	32508.33333	28145.33333	13040
Zfp830	21745.5	6697	5705	6241
Zfp839	19279	18789	22358	19312
Zfp84	11829	11962	13949.33333	12717
Zfp846	19798.25	18004	16332	16008
Zfp85	37171	1261	39948	1129
Zfp850	16327	12590	2836	13540
Zfp862	10685.5	27073.5	26468	26421
Zfp865	29564.5	27309	26673.5	23499
Zfp866	20462	17932	21761.5	23397.5
Zfp867	9575	11728	11922	12632
Zfp868	13692.75	11543.75	11173.25	11389
Zfp869	36934	39239	1799	946
Zfp87	21560.4	24657.8	27026.8	33368.8
Zfp870	14767	16370.5	15541.5	15802
Zfp871	24835.5	20242.5	11730	12076
Zfp874a	39530	39374	38952	2341
Zfp874b	21381	24032	25381	31742
Zfp879	35386	39217	38562	39206
Zfp9	11671.33333	16595.33333	19388	20452
Zfp90	20599	10755	17190	7083
Zfp91	29181.2	18235.6	26342.6	24321.8
Zfp92	8194	28622	30129	28871
Zfp93	12332	15536	11901	12252
Zfp932	7379	6520	5233	6462
Zfp933	26666.33333	29364	22775.66667	28305.66667
Zfp934	17451.5	36212	32257.5	37880

Zfp935	26735.8	19851.6	19417	19169.6
Zfp938	20744.33333	9670.333333	13272	14521.33333
Zfp939	37995	21149	25939	23225
Zfp94	7811	6413	7282	6866
Zfp940	40490	561	34601	39844
Zfp941	34709	28344	40390	35825
Zfp942	16702	18485	13766	17181
Zfp943	21813	7108	7911.5	23103
Zfp944	35564.66667	22006.66667	9784.666667	9101.333333
Zfp945	19726.75	18777.5	10936.25	9357.75
Zfp946	14617	16618	10080	14532
Zfp947	1642	11398	41080	14080
Zfp948	29591	28695	31307	30842
Zfp949	29183	35146	40189	40846
Zfp952	24323.5	16160	18824.5	36778.5
Zfp954	17513	17638	16481	16469
Zfp956	11592	8734	8521	9673
Zfp957	14061	18900	19018	20780
Zfp958	33569	31474	26619	31608
Zfp959	26062	24048	24023	24572.5
Zfp961	35667.25	24042.75	26447.75	25741.5
Zfp97	13354.66667	24635	12618.66667	26204.33333
Zfpl1	14400	14127	16009	16352
Zfpm1	13344	14947	20753	22036
Zfpm2	25828	26128	26459.8	21888.6
Zfr	20879.5	9877.5	9769.75	11001.5
Zfr2	16793	8001	18062	18488
Zfx	20287	17586.5	16573.5	14240.5
Zfy1	22743.5	4060	9280	8669
Zfy2	18405	22756.5	6450	24665.5
Zfyve1	3421	5010	5714	3844
Zfyve16	11239.5	7171.5	6776.5	5656.5
Zfyve19	19469	18758	20371	19503
Zfyve21	20467	20602	20747	18928
Zfyve26	17002.6	13663.2	21756.4	19878.6
Zfyve27	30989	16291.33333	16276.33333	29286.33333
Zfyve28	18216	37738	22406	4881
Zfyve9	9690.666667	8040.333333	20711	17530
Zg16	28210	35074	39402	39801

Zgpat	11743	11897	12627	12580
Zgrf1	7911	38205	3330.5	32871.5
Zhx1	29143.33333	19037.33333	18241	31585.33333
Zhx2	9307	13809	5889	15825
Zhx3	9991.8	23165.8	19079.4	9164.2
Zic1	17739.5	11193.5	7447.5	6240.5
Zic2	40452	9645	13597	16119
Zic3	26455.5	27824.5	29727	34677
Zic4	33778	21085.5	19606.5	29619
Zic5	31828	33500	35389	33616
Zik1	13862.5	24861.5	15006	14705
Zim1	24872	26287	32319	26416
Zim2	27191	28758	30062	28575
Zkscan1	13188	14998.6	15948	16467.8
Zkscan14	3520	4732	4612	2931
Zkscan16	30882	781	40056	32049
Zkscan17	32232	32421	25712	31229
Zkscan2	23128.66667	24566	31635.33333	31320.66667
Zkscan3	7654	3865.5	3635.5	3744
Zkscan5	20223.66667	19842.33333	20984.33333	19697.33333
Zkscan8	18262	19593.33333	19356.33333	24330.66667
Zmat1	24906.5	18053	17137	12924.5
Zmat2	7639	8892	11136	7751
Zmat3	40652	28558	29414	25005
Zmat4	32444	31896.5	34201	30420.5
Zmat5	6996	11231	12222	11072
Zmiz1	10577	9006.5	5292	4465.5
Zmiz2	16169	20239	9220	16950
Zmpste24	32920	14078.5	32177.5	14996
Zmym1	40021	580	101	40201
Zmym2	16202	14858	18683.66667	19675.66667
Zmym3	12350	34157	37131	34375
Zmym4	17657.5	32474.5	31213.25	30244.5
Zmym5	11264.66667	22514.33333	11101.66667	24276
Zmym6	16296	32303	13591	15459
Zmynd10	17599	20448	38029	36466
Zmynd11	23778.33333	22921.33333	16172.66667	19033.33333
Zmynd12	10538	14928	12621.66667	16578.66667
Zmynd15	37059	20252	7600	30218

Zmynd19	17614.5	18356.5	14245	16494.5
Zmynd8	19790.75	17519.75	21824	18515.25
ZNF18	1699	18365	19371	37599.5
ZNF23	16276.5	30555	16825.5	38125.5
Znf512b	22186	18697	22427	19915
Znfx1	31496	38373.5	14557	39046.5
Znhit1	18883.5	24868	22647	26436
Znhit2	30388	29664	26021	26440
Znhit3	37921	2697	3980	4375
Znhit6	11932	16309	15724.5	18565
Znrd1	19697.5	20308.5	20113.5	19158
Znrd1as	30354	35309	34354	35106
Znrf1	18582.33333	20958.33333	19219	22168
Znrf2	6754.5	25747.5	3526.5	19547
Znrf3	23015.5	12008	19368	17230
Znrf4	1017	1617	5546	3111
Zp1	22637	23895	37292	38040
Zp2	35412	29098.5	30191.5	17562
Zp3	17899.5	18646.5	21610	21945
Zp3r	28948	38953	27923	1675
Zpbp	27766	39339	30885	28709
Zpbp2	37522.5	27626	2197.5	18720
Zpld1	10292	16985	16631	16911
Zpr1	18047	21638.5	19555.5	23700
Zranb1	27638	28497	27264	31653
Zranb2	461	38419	23382	25725
Zranb3	6637	39008.5	10430.5	5585
Zrsr1	27396	14709.5	9350.5	26218.5
Zrsr2	3999.5	3021.5	5220	5704
Zscan12	20325.5	20522	20419.5	16432
Zscan2	11127	10481	10310	12288
Zscan20	18954	18702.5	20275	20762.5
ZSCAN21	28078	25954	25758	25524
Zscan22	20129	21654	19605	24078
ZSCAN29	4755	8483	3443	5800
Zscan30	1197	29839	2524	39019
Zscan4c	23475	25706.5	12295	30377
Zscan5b	32397	33953	4447	38049
Zswim1	6592	3075	34908	40566

Zswim2	4198	4553	1831	2293
Zswim3	37287	36224	38165	34869
Zswim4	1828	9574	20245	11350
Zswim5	23991.66667	15471	14738.33333	9217
Zswim6	13401	19651.33333	11456	8885.666667
Zswim7	19755	35209	26121	36858
Zswim8	14102.33333	22723.33333	9404.666667	20915.33333
Zufsp	11972.5	11295	6581.5	8709.5
Zw10	15706	15357	16567	18374
Zwilch	17432	14431	17346	15341
Zwint	14253.66667	19326.66667	16241.66667	24171.33333
Zxda	9443	4488	8793	8593
Zxdb	8594	9298	9645	7723
Zxdc	18397	34897.5	30727	30453
Zyg11a	28331	29794	31337	30165
Zyg11b	22935	19001	22558	18642
Zyx	22413	10124	22454	14574
Zzef1	20323.33333	14953.66667	17218.66667	16000.66667
Zzz3	15766.5	11918	11417	11480.5
