# Supplementary material for: Inhibitors of Apoptosis Protein Antagonists (Smac Mimetic Compounds) Control Polarization of Macrophages during Microbial Challenge and Sterile Inflammatory Responses
Source: Front Immunol. 2018 Jan 9;8:1792. doi: 10.3389/fimmu.2017.01792 (PMC5767188; doi:10.3389/fimmu.2017.01792)
Supplement: Figure S1 — (A–E) cIAP1 proteins regulate TNF-mediated immune response in macrophages. mRNA from cIAP-1 WT and KO macrophages stimulated with TNF for 24 h duration was analyzed by whole mouse genome 44 k microarray Kit, Agilent technology (Cat No—G4122F, Chip ID no. 014868). Pathway-Express (PE) was used to map differentially expressed genes and Gene Spring 12.6.1 software (Agilent Technologies, Santa Clara, CA, USA) was used to prepare corresponding heat map. Pathways that were up/downregulated during TNF induction and or CIAP-KO were identified using previously developed method on Gene-Set enrichment analysis. Genes Sets for mouse were downloaded from Bader lab and were obtained by translation of human counterparts using orthologous relationship from homologene database. (A) The representation of up- and downregulated total number of genes by cIAP-1 in macrophages in the form of Venn diagram while (B) are the gene sets from Reactome_3 pathways, (C) from BP_3.5 pathways, and (D) from NCL_2 pathways and gene ontology process from Mouse Genome Database (MGD). [file Data_Sheet_1.zip › Figure Captions.PDF]

Legend to Supplementary figures

Suppl. Figure 1A-E.

cIAP1 proteins regulate TNF mediated immune response in macrophages.

mRNA from cIAP-1 WT and KO macrophages stimulated with TNF for 24fh duration was analyzed by whole mouse genome 44k microarray Kit, Agilent technology Cat No – G4122F, Chip ID no- 014868) Pathway-Express (PE) was used to map differentially expressed genes and Gene Spring 12.6.1 software (Agilent Technologies, Santa Clara, CA, USA) was used to prepare corresponding heat map. Pathways that were up/down regulated during TNF induction and or CIAP-KO were identified using previously developed method on Gene-Set enrichment analysis**.** Genes Sets for mouse were downloaded from Bader lab and were obtained by translation of human counterparts using orthologous relationship from homologene database. **A** is the representation of up and down regulated total number of genes by cIAP-1 in macrophages in the form of Venn diagram while **B** are the gene sets from Reactome_3 pathways, **C** from BP_3.5 pathways and **D** from NCL_2 pathways and gene ontology process from Mouse Genome Database (MGD).

Suppl. Figure 2.

C. pneumonia **infection induced sTNFR1 shedding in TNF stimulated HeLa.**

cIap1 KO upon infection with *C. pneumoniae* and further stimulation with TNF was quantified at defined time intervals. Normal, cIAP1 shRNA transfected and vector control stable HeLa cells clones were infected with *C. pneumoniae* for one infection cycle and soluble/shed TNFR1 were quantified in their culture supernatant by sandwich ELISA method (Bio-Sources). Data is represented as pg/ml of sTNFR1 released ± S.E. from 2 independent experiments. Statistical analysis was conducted using 2way ANOVA followed by Bonferroni post-test (*$p<0.05$; **$p<0.01$; ***$p< 0.001$).

Suppl. Figure 3.

C. pneumonia **interfere with Iron mediate nitric oxide generation in macrophages**

CD11b+/Gr-1- peritoneal macrophages from WT, cIAP KO mice (A) and xIAP KO mice (B) were infected with *C. pneumonia* and stimulated with SNP (sodium nitroprusside) which release NO (nitric oxide) in Iron dependence manner. NO titers were quantified in their culture supernatant at indicated time interval. Data represented as mean of μM of NO ±S.E. and statistical analysis was conducted using 2way ANOVA followed by Bonferroni post-test (***$p<0.001$).

Suppl. Figure 4.

IAP deficiency promotes *C. pneumonia* mediated interference of macrophage stimulation and their metabolic activity

(A) WT, cIAP KO and xIAP KO macrophages were infected with *C. pn* and the fate of altered metabolic activity in these macrophages at specified time points was analyzed by MTT assay. In similar lines, cpn infection mediated altercations in metabolic activity of IFNγ (B), TNF (C) and SNP

(D) stimulated macrophages were also quantified. Statistical analysis was conducted using 2way ANOVA followed by Bonferroni post-test (***p<0.001).

Suppl. Figure 5.

Smac mimicry enhances M. smegmatis/ E. coli infection induced NO production macrophages.

RAW 264.7 macrophages were infected with M. smegmatis / E. coli and after 1h post infection extra cellular bacteria washed off and cells co-stimulated either with IFNγ or BP and combination of IFNγ +BP. NO production by these macrophages was quantified in cell supernatant at 24h (A) and 48h (B) post infection. Bacterial counts (CFU/ml) were measured in macrophage cell lysates at 24h (C & D) and 48h post infection (E & F). Data are represented as mean ±S.E. of three independent experiments in triplicates and statistical analysis was conducted using Student t- test (*p ≤0.05, ** p ≤0.01, *** p≤0.001).

Suppl. Figure 6.

***M. smegmatis and E. coli* do not interfere with IFNγ induced iNOS production in macrophages.**

RAW 264.7 macrophages were infected with *M. smegmatis* and *E. Coli* and stimulated either with/without IFNγ and/or BP and expression of iNOS and IAPs in these whole cell lysates were analyzed by Immunoblotting at 24h (A & B) and 48h (C & D) post infection. Data shown is the representative of three independent repeats and the densitometry analysis of the representative blots was done using ImageJ and presented as ratio of proteins with actin.

Suppl. Figure 7. *Leishmania donovani* skew M2 phenotype in macrophages and interfere with IFNγ stimulated generation of NO in macrophages

RAW 264.7 macrophages were infected with Leishmania donoveni (MOI-5) and after 4 h the extracellular parasites were washed off. Macrophages were stimulated with IFNγ and treated with BP while maintaining untreated cells as controls. NO titres were quantified in cell culture supernatant at 24h and 48h post infection. Data presented is a mean of three independent experiments done in triplicates and statistical analysis was conducted using Student t- test (*p<0.05; **p<0.01; ***p<0.001).

Suppl. Figure 8.

IAP regulates hypoxic mediated sterile inflammatory responses in iNOS+ macrophages

RAW macrophages were cultured in presence of CoCl2 and the expression of iNOS and HIF1 proteins was analyzed by immunofluorescence **(A)** for confirming M1 polarization of these macrophages. RAW264.7A murine MΦ were stimulated with Th1 stimuli viz LPS or IFNγ and both LPA and IFNγ with and without CoCl2 and birinapath and cultured for indicated time points. **(B)** NO titer was quantified cell culture supernatants and the data was represented as a mean µM of NO± SEM. Statistical analysis was conducted using one way ANOVA followed by Bonferroni post-test (*p<0.05; **p<0.01; ***p<0.001) **(C)** The cultures mentioned under **B** were lysed and analyzed for various M1 and M2 effector proteins, IAPs and signaling markers by western blotting. **(D)** Densitometry quantification of western blots from three independent repeats were analyzed by Image

J and the data was plotted as mean of protein/actin ratio **E** To monitor the intracellular signaling, important metabolic signaling component activation was observed using PathScan Intracellular Signaling Array Kit from Cell Signaling Technology. Images were analyzed by using ImageJ software and mean densitometry values were plotted in terms of relative expression. Statistical analysis was conducted using 2way ANOVA followed by Bonferroni post-test (*p<0.05; **p<0.01; ***p< 0.001).

Supp. Figure 9.

IAP regulates angiogenic programming in iNOS+ macrophages

RAW264.7A murine MΦ were stimulated with Th1 stimuli viz LPS or IFNγ and both LPS and IFNγ with and without VEGF and birinapath and cultured for indicated time points. **(A)** NO titer was quantified in the cell culture supernatant and the data was represented as the mean μM of NO ± SEM. Statistical analysis was conducted using 1way ANOVA followed by Bonferroni post-test (*p<0.05; **p<0.01; ***p<0.001). **(B)** The cultures mentioned under **A** were lysed and analyzed for various M1 and M2 effector proteins, IAPs and signaling markers by western blotting. **(C)** Densitometry quantification of western blots from three independent repeats were analyzed by Image J and the data was plotted as mean of protein/actin ratio **(D)** To monitor the intracellular signaling, important metabolic signaling component activation was observed using PathScan Intracellular Signaling Array Kit from Cell Signaling Technology. Images were analyzed by using ImageJ software and mean densitometry values were plotted in terms of relative expression. Statistical analysis was conducted using 2way ANOVA followed by Bonferroni post-test (*p<0.05; **p<0.01; ***p< 0.001).

Supp. Figure 10. IAP control hypoxia induce polarization in Th1 committed CD11b+ mouse peripheral macrophages

CD11b+ peritoneal macrophages were purified by MACS based method and cultured o/n and stimulated with VEGF and CoCl2 in presence of Brinapant. NO titres were quantified in both naïve **(A)** and IFNγ stimulated (**B**) macrophages. Shown here is the mean μM of NO ± SEM from three independent experiments.

Supp. Figure 11. IAP proteins re-program metabolic activity in both naive and INOS+ macrophages during macrophage polarization.

RAW264.7A murine MΦ either naïve **(A)** or stimulated with Th1 effector cytokine IFNγ **(B)**; were treated with metformin in both normal and high glucose condition and in presence and absence of birinapath and cultured for indicated time points. NO titer was quantified and the data represent the mean μM of NO ± SEM. Statistical analysis was conducted using 2way ANOVA followed by Bonferroni post-test (*p<0.05; **p<0.01; ***p<0.001).
